# Characterization of paralogous protein families in rice

**DOI:** 10.1186/1471-2229-8-18

**Published:** 2008-02-19

**Authors:** Haining Lin, Shu Ouyang, Rain Simons, Kan Nobuta, Brian J Haas, Wei Zhu, Xun Gu, Joana C Silva, Blake C Meyers, C Robin Buell

**Affiliations:** 1 The Institute for Genomic Research, 9712 Medical Center Dr., Rockville, MD 20850, USA and J.Craig Venter Institute, 9704 Medical Center Dr., Rockville, MD 20850, USA; 2 Department of Plant and Soil Sciences & Delaware Biotechnology Institute, University of Delaware, Newark, DE 19711, USA; 3 Department of Genetics, Development, and Cell Biology, Iowa State University, Ames, IA 50011, USA; 4 Center for Bioinformatics and Biological Statistics, Iowa State University, Ames, IA 50011, USA; 5 The Broad Institute, 7 Cambridge Center, Cambridge, MA 02142, USA; 6 Institute for Genome Sciences & Department of Microbiology & Immunology, University of Maryland, Baltimore, School of Medicine, HSF-II, Rm S-445, 20 Penn St., Baltimore, MD 21201, USA; 7 Department of Plant Biology, Michigan State University, 166 Plant Biology Building, East Lansing, MI 48824, USA

## Abstract

**Background:**

High gene numbers in plant genomes reflect polyploidy and major gene duplication events. *Oryza sativa*, cultivated rice, is a diploid monocotyledonous species with a ~390 Mb genome that has undergone segmental duplication of a substantial portion of its genome. This, coupled with other genetic events such as tandem duplications, has resulted in a substantial number of its genes, and resulting proteins, occurring in paralogous families.

**Results:**

Using a computational pipeline that utilizes Pfam and novel protein domains, we characterized paralogous families in rice and compared these with paralogous families in the model dicotyledonous diploid species, *Arabidopsis thaliana*. Arabidopsis, which has undergone genome duplication as well, has a substantially smaller genome (~120 Mb) and gene complement compared to rice. Overall, 53% and 68% of the non-transposable element-related rice and Arabidopsis proteins could be classified into paralogous protein families, respectively. Singleton and paralogous family genes differed substantially in their likelihood of encoding a protein of known or putative function; 26% and 66% of singleton genes compared to 73% and 96% of the paralogous family genes encode a known or putative protein in rice and Arabidopsis, respectively. Furthermore, a major skew in the distribution of specific gene function was observed; a total of 17 Gene Ontology categories in both rice and Arabidopsis were statistically significant in their differential distribution between paralogous family and singleton proteins. In contrast to mammalian organisms, we found that duplicated genes in rice and Arabidopsis tend to have more alternative splice forms. Using data from Massively Parallel Signature Sequencing, we show that a significant portion of the duplicated genes in rice show divergent expression although a correlation between sequence divergence and correlation of expression could be seen in very young genes.

**Conclusion:**

Collectively, these data suggest that while co-regulation and conserved function are present in some paralogous protein family members, evolutionary pressures have resulted in functional divergence with differential expression patterns.

## Background

Gene duplication is a major contributor to genetic novelty and proteomic complexity. Evolutionary pressures on duplicated genes differ from single copy (singleton) genes and several models have been proposed for the evolutionary fate of duplicated genes. In the non/neofunctionalization model, one of the duplicated genes becomes a pseudogene through the accumulation of deleterious mutations although on a rare occasion, it may acquire a new function [1]. In the subfunctionalization model [2-4], duplicated genes adopt a subset of functions of the ancestral gene. Functional redundancy of duplicated genes has been shown to increase the robustness of biological systems [5].

Gene duplication occurs frequently in plants, either in the form of segmental duplication, tandem duplication, and at the level of whole genome duplication [6-14]. Genome duplication has been reported in rice (*Oryza sativa*), an important agricultural species and model species for the grass family (Poaceae) [15-19]. Depending on the methods, parameters, and genome assemblies used, 15% to 62% [15-19] of the rice genome underwent one round of large-scale segmental duplication that occurred approximately 70 Million Years Ago (MYA) [15,16,18]. A more recent duplication, on the short arms of chromosomes 11 and 12, occurred approximately 5 ~8 MYA [15,20]. With respect to tandem duplications, depending on the parameters utilized, 14–29% of rice genes occur in tandem [21]. Paralogous families, composed of tandemly and segmentally duplicated genes, have been studied to a limited extent in rice, typically in a comparative context with the finished genome of the dicotyledonous plant species, *Arabidopsis thaliana *[22-27]. To date, only limited genome-wide analyses of paralogous protein families have been reported in rice [28,29]. In Horan *et al*. [28], Arabidopsis and rice proteins were co-clustered using Pfam domain-based or BLASTP-based similarity clustering which allowed for the clustering of proteins into families common between these two model species and for the identification of proteins that were species-specific.

In this study, we classified proteins from the predicted rice proteome into paralogous protein families using a computational pipeline that utilizes both Pfam and BLASTP-based novel domains [30]. While the focus in our study was analysis of the rice paralogous families, for comparative purposes, we performed a similar classification with the predicted Arabidopsis proteome to compare and contrast paralogous family composition and features in two model species which represent two major divisions of the angiosperms, monocots and dicots. In rice, we characterized alternative splicing, functional classification of paralogous family proteins, expression patterns, and duplication age and compared these data to those observed in single copy proteins. A parallel analysis of alternative splicing and functional domain composition of paralogous family proteins was performed with Arabidopsis to compare and contrast with the findings in rice. To highlight our observations, we examined in depth two rice protein families, prolamin and Bowman-Birk inhibitor. This study provides a comprehensive analysis of rice paralogous families in parallel with a comparative analysis in Arabidopsis thereby providing novel insight into paralogous gene family evolution in these two model plant species.

## Results and Discussion

### Classification of paralogous protein families in rice and Arabidopsis

**Figure 1:**
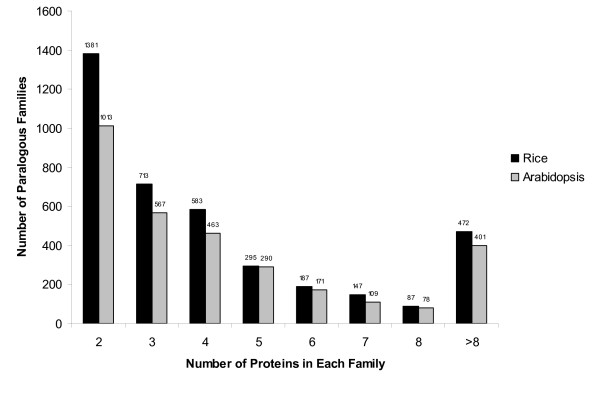
Size distribution of paralogous protein families in rice and Arabidopsis. The exact number of families is listed above the bars.

A total of 3,865 paralogous protein families containing 21,998 proteins were identified [see Additional file 1] from the 42,653 total non-transposable element (TE)-related proteins predicted in the rice genome, leaving 20,655 putative singleton proteins encoded by single copy genes. On average, a rice family contained six family members, ranging in size from two to 214 family members (Fig. 1). A total of 11 paralogous protein families with more than one hundred member proteins were identified in rice which encoded proteins such as zinc finger proteins, protein kinases, Myb-like proteins, and transducins [see Additional file 2], similar to the largest protein families reported in Arabidopsis [30]. Paralogous protein family genes of rice were distributed throughout the genome and within chromosomes in a pattern similar to the singleton genes [see Additional file 3A]. Although paralogous protein family genes were more frequently located in the euchromatic regions, this was consistent with previous reports that non-TE-related genes are found more prevalently in euchromatic regions. A comparison of segmentally duplicated genes with the paralogous protein family genes suggested that our classification pipeline was robust. Of the 2,403 segmentally duplicated gene pairs within 163 segmentally duplicated blocks, 1,570 duplicated gene pairs (65%) were classified in the same paralogous protein family. For the remainder of the segmentally duplicated genes, 175 pairs (7%) were classified in different paralogous protein families and 268 (11%) had one gene classified in a paralogous protein family and the other gene classified as a singleton. We observed that 390 segmentally duplicated gene pairs (16%) were not included in any paralogous protein family. Note that in our computational pipeline, four or more members were required to define a BLASTP-based domain. Consequently, a single pair of segmentally duplicated genes alone is insufficient to define a BLASTP-based domain. The lack of 100% correspondence between segmental duplication and paralogous family classification may be due to the acquisition of new domain(s) or loss of existing domain(s) within one of the duplicated genes as in our computational pipeline, only proteins with the identical domain composition were classified into the same paralogous protein family. Alternatively, the difference could be due to the different classification methods employed in each method. For example, LOC_Os08g37350 and LOC_Os09g28940 are segmentally duplicated genes from chromosomes 8 and 9, respectively. These two protein sequences had a 56% identity over 70% of the length of the longer sequence and were within a segmentally duplicated block of 43 collinear gene pairs. LOC_Os08g37350 has two Pfam domains (PF00443: Ubiquitin carboxyl-terminal hydrolase; PF01753: MYND finger) while LOC_Os09g28940 has only one Pfam domain (PF00443: Ubiquitin carboxyl-terminal hydrolase). As a consequence, these loci were classified in two different paralogous families (LOC_Os08g37350 is classified in Family 1545; LOC_Os09g28940 is in Family 3650). In a second example, LOC_Os11g03210 and LOC_Os12g02960 are from a segmental duplication event involving chromosomes 11 and 12 which includes 160 collinear gene pairs. LOC_Os11g03210 has a single Pfam domain (PF02798: Glutathione S-transferase, N-terminal domain) and thus is classified in Family 3362 while LOC_Os12g02960 is classified as a singleton as although it has two Pfam domains (PF02798: Glutathione S-transferase, N-terminal domain; PF00043: Glutathione S-transferase, C-terminal domain) no other protein has exactly the same domain profile. Note that in our computational pipeline, a paralogous family must have at least two members with identical domain profiles. In a third example, segmentally duplicated genes LOC_Os01g41900 and LOC_Os05g51160 are from chromosomes 1 and 5. These two genes were derived from full length cDNAs (FLcDNAs) and had a 59% identity over approximately three-quarters of the longer protein sequence. LOC_Os01g41900 has two Pfam domains (PF00249: Myb-like DNA-binding domain and PF00098: Zinc knuckle) while LOC_Os05g51160 has only one single Pfam domain (PF00249: Myb-like DNA-binding domain). As a consequence, they were classified in different families, Family 1452 and Family 3863, respectively. Manual inspection of these three sets of loci revealed that they were correctly annotated and that the lack of clustering into a single paralogous family could not be attributed to incorrect structural annotation which is another potential cause for lack of 100% correspondence between segmentally duplicated genes and paralogous families.

A parallel construction of paralogous protein families in Arabidopsis identified 3,092 paralogous protein families (18,183 proteins) and 8,636 single copy genes from a total of 26,819 protein coding genes from TAIR7 release [31]. A similar size distribution of Arabidopsis protein families was observed, ranging from two to 182 (Fig. 1). In Arabidopsis, the largest families encode Myb-like proteins, zinc finger proteins, and protein kinases, consistent with what has been reported previously [30]. Arabidopsis paralogous protein family genes distributed similarly to singleton genes and were more frequently located in the euchromatic regions [see Additional file 3B].

### Function of paralogous protein families in rice and Arabidopsis

**Figure 2:**
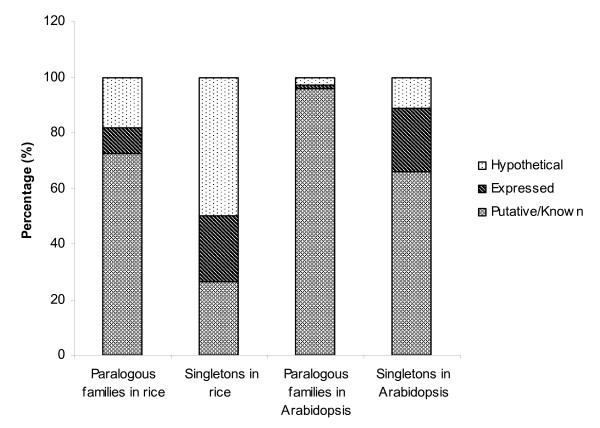
Functional classification of paralogous family and singleton proteins in rice and Arabidopsis.

We examined the functional annotation of paralogous family and singleton proteins. A total of 21,403 and 23,081 genes were annotated as encoding known or putative proteins in rice and Arabidopsis, respectively, due to strong similarity with proteins with a known function or the presence of Pfam domains above the trusted cutoff. Genes with no known or putative function can be supported by experimental transcript evidence (i.e., encode an "expressed protein") or are predicted solely by an *ab initio *gene finder and lack expression support as well as sequence similarity to known proteins with the exception of other hypothetical proteins (i.e., encode a "hypothetical protein"). In rice, a total of 6,913 genes encode expressed proteins as shown by experimental transcript evidence from Expressed Sequence Tags (ESTs), FLcDNAs, Massively Parallel Signature Sequencing [32], Serial Analysis of Gene Expression, and/or proteomic data [33]. In Arabidopsis, 2,270 genes encode expressed proteins as shown by experimental transcript in the form of ESTs and/or cDNA evidence (see Methods). The remaining 14,337 rice genes [33] and 1,468 Arabidopsis genes (see Methods) encode hypothetical proteins. A majority of rice paralogous family genes (73%) encode either a known or putative protein (Fig. 2). The remaining rice paralogous family genes encode expressed proteins (9%) and hypothetical proteins (18%). In contrast, rice singletons had a larger portion of hypothetical genes (50%) and a smaller portion of genes with a known or putative function (26%). Even though Arabidopsis overall has a smaller number of genes with unknown function than rice, a similar bias of genes with a known or putative function in paralogous family genes was observed in a parallel analysis in Arabidopsis (Fig. 2).

**Figure 3:**
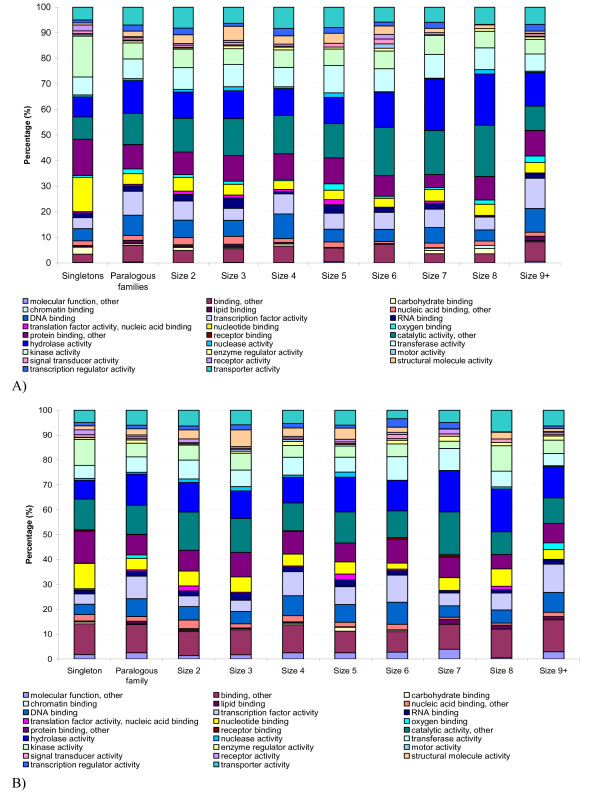
GOSlim assignment of A) rice paralogous families and singletons, B) Arabidopsis paralogous families and singletons. The paralogous protein families are further classified by family size.

Using Plant GOSlim annotations [34], we compared the function of the proteins within rice paralogous families to that in the singletons. Within the 26 molecular function GOSlim categories identified in our analyses, rice paralogous protein families showed different patterns from singletons in a number of GOSlim categories (Fig. 3A). Although, the relative abundance of each GOSlim category varied with the size of the rice paralogous family, no obvious correlation was observed (Fig. 3A). For each category, a two-tailed two-sample binomial test was performed by comparing the abundance of that category in rice paralogous families with that in the singletons. Multiple testing was corrected using the Benjamini and Hochberg false discovery rate control at a level of 0.05 [35]. The statistical test revealed a substantial enrichment of 12 categories in rice paralogous family proteins including transcription factor activity, hydrolase activity, DNA binding, and transporter activity while a substantial reduction was seen in five categories including receptor activity, nucleotide binding and carbohydrate binding (Table 1). A similar skew in GOSlim categories was observed in a parallel analysis in Arabidopsis (Table 2 & Fig. 3B), consistent with a previous report in Arabidopsis [36] that non-random loss and retention of paralogous genes with different functions occurred after gene duplication.

**Table 1:** Two-sample binomial tests for GOSlim assignments of paralogous family and singleton proteins in rice

GOSlim assignment^a^	Singletons (%)	Paralogous genes (%)	P-value^d^
Binding, other^b^	3.3	6.5	<1e-5
Carbohydrate binding^c^	2.7	0.6	<1e-5
DNA binding^b^	4.8	8.0	<1e-5
Hydrolase activity^b^	7.8	12.7	<1e-5
Kinase activity^c^	16.0	6.2	<1e-5
Nucleotide binding^c^	13.4	4.2	<1e-5
Protein binding, other^c^	14.2	9.5	<1e-5
Receptor activity^c^	2.3	0.4	<1e-5
Transcription factor activity^b^	4.3	9.3	<1e-5
Catalytic activity, other^b^	8.7	12.2	<1e-5
Structural molecule activity^b^	0.8	2.2	<1e-5
Oxygen binding^b^	0.7	1.9	<1e-5
Transcription regulator activity^b^	1.1	2.3	<1e-5
Transporter activity^b^	5.0	7.0	<1e-5
Lipid binding^b^	0.4	1.1	<1e-5
Molecular function, other^b^	0.1	0.4	0.001
Enzyme regulator activity^b^	0.5	0.9	0.008
Motor activity	0.5	0.3	0.051
Transferase activity	7.0	7.7	0.095
Receptor binding	0.0	0.1	0.137
RNA binding	1.8	2.1	0.369
Translation factor activity, nucleic acid binding	0.5	0.7	0.353
Signal transducer activity	1.0	0.9	0.43
Chromatin binding	0.3	0.2	0.465
Nucleic acid binding, other	1.8	1.9	0.882
Nuclease activity	0.8	0.8	0.888

^a ^GoSlim assignment classifications were performed as described in the Materials and Methods.

^b ^Enrichment of GOSlim annotations in paralogous protein families compared to singletons.

^c ^Reduction of GOSlim annotations in paralogous protein families compared to singletons.

^d ^Benjamini and Hochberg correction for multiple testing.

**Table 2:** Two-sample binomial tests for GOSlim assignments of paralogous family and singleton proteins in Arabidopsis

GOSlim assignment^a^	Singletons (%)	Paralogous genes (%)	P-value^d^
Hydrolase activity^b^	7.5	12.6	<1e-5
Kinase activity^c^	10.4	5.5	<1e-5
Nucleotide binding^c^	10.2	4.6	<1e-5
Protein binding, other^c^	12.9	8.2	<1e-5
Transcription factor activity^b^	4.2	9.0	<1e-5
Receptor activity^c^	1.9	0.7	<1e-5
DNA binding^b^	4.1	7.2	<1e-5
Oxygen binding^b^	0.1	1.4	<1e-5
Receptor binding^c^	0.5	0.1	<1e-5
Carbohydrate binding^c^	0.7	0.3	<1e-3
Lipid binding^b^	0.3	0.8	0.001
Structural molecule activity^b^	1.6	2.5	0.002
Enzyme regulator activity^b^	0.7	1.4	0.005
Molecular function, other^b^	1.8	2.5	0.011
Transporter activity^b^	5.0	6.0	0.019
Nucleic acid binding, other^c^	2.6	2.0	0.027
Motor activity^b^	0.2	0.5	0.03
Transferase activity	5.3	6.1	0.053
RNA binding	1.5	1.9	0.099
Binding, other	12.3	11.3	0.102
Signal transducer activity	1.0	0.8	0.132
Catalytic activity, other	12.4	11.7	0.244
Transcription regulator activity	1.3	1.5	0.743
Chromatin binding	0.2	0.1	0.803
Translation factor activity, nucleic acid binding	0.6	0.6	1
Nuclease activity	0.7	0.8	1

^a ^GoSlim assignment classifications were performed as described in the Materials and Methods.

^b ^Enrichment of GOSlim annotations in paralogous protein families compared to singletons.

^c ^Reduction of GOSlim annotations in paralogous protein families compared to singletons.

^d ^Benjamini and Hochberg correction for multiple testing.

### Paralogous protein family genes tend to have more alternative isoforms than singletons

Alternative splicing has been regarded as a mechanism to increase genetic novelty. In the rice genome, 6,253 non-TE-related genes have evidence of alternative splicing (see Methods) and we used this set of genes to examine alternative splicing in singleton versus paralogous protein family genes. The percentage of alternative splicing in single copy genes is 2,094/20,655 = 10.1%, while that in paralogous family genes is 4,159/21,998 = 18.9%; a statistically significant difference (*χ*^2 ^test, P < 1e-5). To remove any bias due to genes that lack transcript evidence, we restricted our analysis to genes with EST and/or FLcDNA evidence. The percentage of alternative splicing in singletons is 2,094/8,619 = 24.3%, while that in paralogous protein family genes is 4,159/14,072 = 29.6%; a statistically significant difference (*χ*^2 ^test, P < 1e-5). We further restricted our analysis to high confidence genes whose structures were completely supported by ESTs and/or FLcDNAs. The percentage of alternative splicing in singletons increases to 1,826/5,964 = 30.6%, while that in paralogous protein family genes increases to 3,765/11,235 = 33.5%; a statistically significant difference (*χ*^2 ^test, P < 1e-3).

To confirm that our observation was not restricted to rice, we performed a parallel analysis with Arabidopsis. Using data on alternative splicing as provided with the TAIR7 release (see Methods), the percentage of alternative splicing in Arabidopsis single copy genes is 943/8,636 = 9.8%, while that in paralogous protein family genes is 2,856/18,183 = 15.7%. This difference is also statistically significant (*χ*^2 ^test, P < 1e-5), similar to that observed in rice. Restricting the analysis to only those Arabidopsis genes with EST and/or cDNA support as provided in the TAIR7 release revealed that the percentage of alternative splicing in singletons is 942/6,663 = 14.1%, while that in paralogous family genes is 2,852/15,369 = 18.6%; a statistically significant difference (*χ*^2 ^test, P < 1e-5). Our findings are contradictory to previous reports in model animal species in which duplicated genes tend to have fewer alternative spliced isoforms thereby supporting the 'function-sharing model' that alternative splicing and gene duplication are two mechanisms that are complementary with respect to proteomic function diversity [37,38]. Our results suggested that plants may employ multiple mechanisms for proteomic complexity, gene duplication and alternative splicing.

### Age of paralogous protein families in rice

While there are previous reports on gene duplication in rice [15-19], they utilized alternative assemblies and annotation datasets of the rice genome. To provide information on the age of paralogous families identified in this study, we estimated the age of a paralogous family from the maximum value of the distribution of pairwise *d*_S _calculated among all members of that protein family (see Methods). We found that the origin of most paralogous families dates back to over 115 Million Years (MY), the point at which synonymous sites are saturated and dating becomes unreliable (*d*_S _~1.5) [see Additional file 4A]. Among protein families for which the maximum pairwise *d*_S _value is less than 1.5, the distribution of maximum *d*_S _is fairly flat, with the exception of a recent peak at *d*_S _between 0 and 0.1 [see Additional file 4B]. This suggests that paralogous families have been arising at a relatively constant pace within the past 115 MY, but that a burst of duplication took place within the last 7.5 MY. Alternatively, paralogous families arise at a rate similar to that observed for the first few million years, but about 2/3 of them revert to single-gene status soon thereafter, accounting for the quick decline after the first 7.5 MY. The fairly constant number of older paralogous families can be due to selective constraints maintaining the elevated copy number or if the loss of paralogs is dependent on sequence similarity, such that after ~10% sequence divergence, paralog loss is negligible. Finally, for each family we identified the largest peak below 1.5 (if there was one) in the distribution of all pairwise *d*_S _values. The distribution of this peak value across all families is bimodal [see Additional file 5], and it confirms the presence of a large number of recently duplicated genes (0 ≤ *d*_S _< 0.1). In addition, the peak at 0.7 ≤ *d*_S _≤ 1 most likely results from the large-scale segmental duplication event that occurred ~70 MYA.

### Expression of paralogous protein families in rice

We further examined the expression patterns of the paralogous families using MPSS data from 18 libraries [32]. MPSS tags were searched against our release 4 pseudomolecules and cDNA sequences of all annotated gene models to ensure that all MPSS tags would be identified even if they spanned the intron(s). We found 11,619 genes within the paralogous protein families that were associated with unique, reliable, and significant MPSS tags, which were referred as MPSS-qualifying genes.

**Figure 4:**
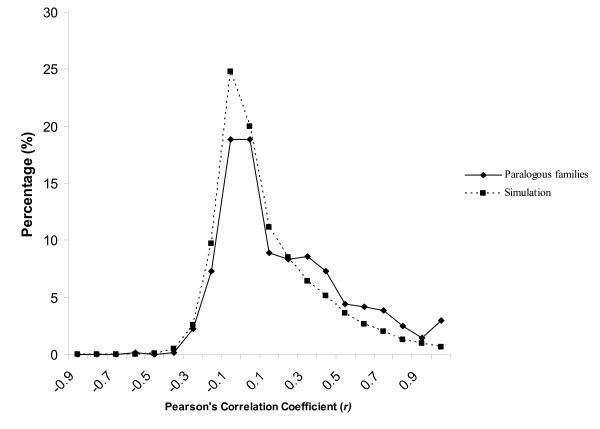
Histogram of Pearson's Correlation Coefficients of expression (*r*) of rice paralogous protein families with exactly two MPSS-qualifying genes.

Suitable summary statistics of correlation for expression divergence of a gene family can be found in Gu [39] and Gu *et al*. [40], though microarray data were the primary focus in these studies. To be concise, we restricted our analysis of expression correlation in the libraries and tissues to paralogous families with exactly two MPSS-qualifying genes (674 protein families). To measure the expression correlation, the Pearson's Correlation Coefficient (*r*) of their expression was computed for each pair of MPSS-qualifying genes from each of the 674 protein families across all 18 MPSS libraries. It is important to note that we excluded MPSS tags which mapped to multiple locations, as most of these are likely to match to closely-related paralogs and could have confounded our analyses. We employed the method used by Blanc and Wolfe [36] to determine a minimum cutoff value for Pearson's Correlation Coefficient (*r*) to classify two duplicated genes as having divergent expression. Basically, a total of 10,000 gene pairs were generated by random shuffling of the singleton genes and the Pearson's Correlation Coefficient (*r*) was calculated similarly for each pair. Ninety five percent of the random shuffled gene pairs had a correlation value *r *< 0.59. As random shuffled gene pairs should have divergent function and expression patterns, we utilized *r *< 0.59 as an indicator of divergent expression. Our results show that the expression correlation value (*r*) of the paralogous protein family genes ranged from -0.6 to 1.0 although the majority of the gene pairs had little correlation with *r *peaking at -0.2 ~0, similar to that observed with the singletons (Fig. 4). Using the correlation cutoff (*r *= 0.59), a total of 598 (89%) paralogous protein families with two-qualifying MPSS genes exhibited divergent expression patterns, consistent with what has been reported in Arabidopsis [36] and in yeast in which more than 80% of the older duplicated gene pairs (*ds *> 1.5) showed divergence in expression [41].

To gain a better understanding of the expression patterns of paralogous protein family members in different organs/tissues, we classified the 18 MPSS libraries [32] into four groups by organs/tissues: roots, leaves, reproductive organs/tissues, and "other tissues". Within the 674 paralogous families with exactly two MPSS-qualifying genes, 239, 168, 223, and 200 paralogous families had only a single member of the pair expressed in roots, leaves, reproductive organs/tissues, and "other tissues", respectively, which demonstrated their diverged expression patterns, and possible tissue-specific expression. To further examine the tissue-specific or stress-induced expression patterns of paralogous protein family members, we calculated the Preferential Expression Measure (PEM) for each of the 1,348 genes from the 674 paralogous families (see Methods) in the 18 MPSS libraries. The PEM shows the base-10 log of ratio of the observed expression level in a given tissue/treatment to the expected expression level assuming uniform expression across all tissues/treatments. A PEM value of 1 means the observed expression level in a given tissue/treatment is 10 times that of expected and indicates strong tissue specific expression. For each gene, tissue(s) with a stringent cutoff of PEM ≥ 1 were compared with the other member of the duplicated gene pair. A total of 375 (375/674 = 55.6%) of the paralogous families showed little tissue-specific expression as none of the associated PEMs had a value equal to or greater than 1. Two hundred ninety-nine families showed strong tissue specific expression patterns; 19 families were preferentially expressed in the same tissue or treatment, 49 families were preferentially expressed in different tissues or treatments, and 231 families had only one of the duplicated genes with preferential tissue-specific expression.

We further examined the correlation between expression divergence and sequence divergence. For each family, we calculated the Pearson's Correlation Coefficient (*r*) for all possible pairs of the MPSS-qualifying genes to measure expression divergence. We then used *ds *as a proxy of divergence time for each gene pair. We restricted our analysis to *d*_S _≤ 1.5 so that the synonymous sites are not saturated. The Pearson's Correlation Coefficient (*r*) values were plotted against the *d*s values for each interval of 0.1 to gain better resolution. That is, we plotted for gene pairs with 0 <*d*_S _≤ 0.1, 0.1 <*d*_S _≤ 0.2, 0.2 <*d*_S _≤ 0.3, and so on. We found no correlation between *d*_S _and correlation of expression except for gene pairs with 0 <*d*_S _≤ 0.1 (R = 0.33, P < 1e-4) where duplicated genes were relatively young [see Additional file 6]. The number of non-synonymous substitutions per site (*dN*) was also calculated for each gene pair and plotted against correlation of expression. No correlation was observed between *dN *and correlation of expression (data not shown). This is consistent with reports in Arabidopsis in which expression divergence is not strictly coupled with sequence divergence as shown by no appreciable change for the majority of gene duplicates with highly diverged amino acid sequences in expression pattern in developing roots [42].

Positive correlation of expression patterns among paralogous protein family members would suggest that similar transcriptional regulation was retained in both members and possibly, similar functions. However, we observed a large number of gene pairs with little expression correlation which could be an indication of subfunctionalization or neofunctionalization after gene duplication. The duplication-degeneration-complementarity (DDC) model proposed by Force et al. [3] and Lynch and Force [4] suggests that subfunctionalization is a major mechanism for retention of duplicated genes as a result of differential expression caused by accumulation of mutations in regulatory regions rather than protein coding regions. The 49 families with preferential expression in two different tissues or treatments, along with the 231 families having only one member of the paralogous pair preferentially expressed, is a strong indicator of subfunctionalization. As our paralogous protein family classification required that each family member have the same domain profile, the differential expression may be attributable to mutations in regulatory regions rather than gene coding regions, consistent with the DDC model.

### Case studies of rice paralogous protein families

#### Prolamin protein family

Prolamin is one of the major endosperm storage proteins in cereal grains such as wheat, barley, rye, maize, and sorghum [43-46]. It was named prolamin due to its high content of proline and glutamine. In rice, prolamin contributes 35% of the total seed protein [47]. Three classes of prolamins have been identified in *Oryza *by their molecular weights: 10, 13, and 16 kDa [48]. The major prolamin families in rice are Family 3722 (20 members) and Family 3193 (seven members). Members of both families have a BLASTP-based domain. Members of Family 3193 have a Pfam domain (PF00234; Protease inhibitor/seed storage/LTP family) in addition to the common BLASTP-based domain and thus were not clustered within Family 3722 as the exact same domain profile is required for each family member in our computational pipeline [see Additional file 7]. All of the prolamin genes were single-exon genes as reported previously [49] with the exception of four genes that contained a single intron which were further examined and found that based on the EST alignments they were single-exon genes that had not been properly annotated (data not shown). The length of the deduced amino acids of the prolamin proteins (excluding the four inaccurate genes) varied from 101 to 156 bp with two peaks at 101~110 and 145~160 bp, consistent with what had been reported in rice prolamin proteins [49,50].

Only five prolamin family members (LOC_Os05g26720.1, LOC_Os05g26770.1, LOC_Os06g31070.1, LOC_Os12g16880.1, LOC_Os12g16890.1) were associated with unique, reliable, and significant MPSS tags, which, as expected, were exclusively expressed in 3-day germinating seeds with relatively high abundances (198, 562, 1042, 148, and 670 Transcripts Per Million (TPM), respectively) [see Additional file 8]. We also examined the expression of the two prolamin families with that of Family 3856 (123 members) which contained the same Pfam domain (PF00234) that was in prolamin family 3193 [see Additional file 7]. A total of 54 genes from Family 3856 were associated with unique, reliable, and significant MPSS tags. However, the expression pattern observed in Family 3856 substantially differed from that of the prolamin families (Family 3722 and Family 3193) in that most of the genes were expressed in multiple organs/tissues [see Additional file 9].

Interestingly, we observed that genes encoding the prolamin protein family seemed to localize closely on the chromosomes. A total of 16 prolamin protein family genes were located together on chromosome 5 with a large number of TE-related genes inserted between the family members [see Additional file 10]. Other prolamin protein family genes were located on chromosome 6 (two genes in tandem), chromosome 7 (in two gene clusters), and chromosome 12 (three genes with TE-related genes inserted between them), suggestive of tandem duplication(s) of the prolamin protein family genes followed by insertion of transposable elements throughout the course of evolution. This is consistent with previous report on the compact expansion of *α*-zein gene family of maize [13].

#### Bowman-Birk Inhibitor (BBI) type protein family

BBI is a cysteine-rich protein which has trypsin and chymotrypsin inhibitory activities [51]. It was first characterized in soybean [52,53] and later found widely distributed in monocot and dicot species [54-58]. It has been extensively studied due to its possible role in plant defense [51,54,58] and its potential application in cancer chemoprevention [59-61]. The major BBI type protein families in rice are Family 3328 (eight members) and Family 1493 (three members). While both families have the Pfam domain PF00228 (Bowman-Birk serine protease inhibitor family), Family 3328 also has a second domain identified via BLASTP [see Additional file 11]. Amino acid composition analysis showed that 31% and 47% of the conserved residues of Family 3288 and Family 1493, respectively, was cysteine suggesting that this amino acid has an important role in the protease inhibitory activity of BBI. These composition data also revealed subtle differences between the two BBI type protein families. The phylogenetic tree generated by MEGA version 3.1 [62] for family 3328 [see Additional file 12] suggests that after the original duplication event, only one of the paralogs underwent further rounds of duplication, consistent with the physical clustering of this set of BBI genes on chromosome 1 [see Additional file 13].

MPSS analysis showed that the BBI genes were differentially expressed in a wide range of tissues and organs, consistent with previously reported expression patterns [58]. Seven genes of Family 3328 were associated with unique, reliable, and significant MPSS tags with the pairwise Pearson's Correlation Coefficient values ranging from -0.35 to 0.71. Two genes within Family 1493 were associated with unique, reliable, and significant MPSS tags, which showed little correlation in expression (*r *= -0.12). It would be interesting to determine expression levels of the BBI genes following wounding, as seven proteins of the Family 3328 were annotated as Bowman-Birk type bran trypsin inhibitor precursors, a type which was reported to play an important role in plant defense [54,58], and two members of the Family 1493 were annotated as wound-induced BBI type WIP1 precursors [33].

## Conclusion

We demonstrated that even relatively small plant genomes such as rice and Arabidopsis have a significant portion of their proteomes in paralogous families, resulting in a partially redundant proteome. The origin of most paralogous gene families in the rice genome seems to be very old, but duplicates have continued to arise at a fairly steady pace, with a peak in duplication being coincident with a major segmental duplication that took place at ~70 MYA. While conservation of protein domains was clearly observed within rice and Arabidopsis paralogous families, we did observe a major skew in types of proteins and protein domains within paralogous families versus singleton proteins, suggesting an impact of selection occurred during genome evolution and gene duplication. Another level of potential functionality in paralogous family proteins could also occur through alternative splicing which was statistically more frequent in paralogous family proteins compared to singletons in both rice as well as Arabidopsis. In rice, while some paralogous family members were transcriptionally co-regulated, divergence in expression patterns was clearly evident, thereby allowing an expanded range of functionality for the protein. These data suggested that multiple mechanisms are present in plant genomes to generate protein diversity and that these two model plant species share at least a subset of these mechanisms.

## Methods

### Construction of paralogous protein families

In release 4 of the TIGR Rice Genome Annotation [33], a total of 55,890 genes were annotated, of which 13,237 were related to TE. The TE-related genes were excluded from all further analyses. As alternative splicing occurs in the rice genome and some genes have multiple splice forms, the largest peptide sequence was used whenever alternative isoforms existed. Short protein sequences (<50 amino acids) were excluded from this analysis. A total of 42,653 rice protein sequences were used to classify paralogous protein families using protein domain compositions as described in Haas *et al*. [30]. The basic approach for generating the protein families involved identification of the domains followed by organization of the families based on domains. Two different types of domains were used for the generation of paralogous families: Pfam/HMM domains and BLASTP-based domains. For the Pfam/HMM domains, the predicted rice proteome was searched against the Pfam HMM domain database [63] using HMMER2 [64] and proteins with scores above the trusted cutoff value were retained. For the BLASTP-based domain, peptide regions that were not covered by the Pfam HMM profiles were then clustered based on homology derived from an all versus all BLASTP search [65]. Links were made if two peptides had an >45% identity over >75 amino acids with an E-value <0.001. To prevent multi-domain proteins that are not related from artificially clustering due to single linkages, the Jaccard coefficient of community [66], also known as link score, was used in the clustering process. As described in Haas *et al*. [30], a link score was calculated for the pairs of linked peptide sequences *a *and *b *as follows:

$$J_{a,b}=\frac{\#distinct\;sequences\;matching\;a\;and\;b\;including(a,b)}{\#distinct\;sequences\;matching\;either\;a\;or\;b}$$

Peptides with a link score above the cut-off value (0.66) were selected to generate single linkage clusters. Clustered peptides were then aligned using CLUSTALW [67,68] and used to develop BLASTP-based domains, which were used to build the families if the domain alignments contained four or more members. Protein families were then organized based on the domain composition that refers to the type and number of the domains, which included both Pfam HMM domains and BLASTP-based domains. Proteins with identical domain composition were then classified into putative protein families. Paralogous protein families in Arabidopsis were constructed similarly with a total of 26,819 protein coding genes from the TAIR7 release of the predicted proteome [31].

### Identification of segmentally duplicated genes

Segmentally duplicated genes in the rice genome were defined in Release 4 as described previously [69]. In brief, similar gene pairs were identified by all versus all BLASTP search (WU-BLASTP, parameters "V = 5 B = 5 E = 1e-10 -filter seg") [65], which were then used to define segmentally duplicated blocks by running DAGchainer [70] with parameters "-s -I -D 100000".

### Functional classification of Arabidopsis proteome

A total of 26,819 Arabidopsis protein coding genes were downloaded from the TAIR7 release of the predicted proteome [31] and searched against an in-house non-redundant amino acid database that contains all publicly available protein sequences (e.g. GenBank, Swissprot, etc.) using BLASTP [65] and the Pfam HMM domain database [63] using HMMER2 [64]. BLASTP matches to Arabidopsis sequences were excluded unless they were from Swissprot. BLASTP matches to conserved hypothetical or hypothetical proteins were excluded as well. Arabidopsis proteins with a BLASTP match (< 1e-10 and > 30% identity over 50% coverage) or Pfam domains with scores above the trusted cutoff value were classified as known or putative proteins. The remaining Arabidopsis genes were classified as expressed genes or hypothetical genes according to the gene set downloaded from TAIR7 release [31] which had at least one supporting cDNA and/or EST.

### GOSlim assignment

To assign Gene Ontologies (GO) [71], the predicted rice proteome was searched against the predicted Arabidopsis proteome (TAIR6 Genome Release) [31] using BLASTP. Using an E-value cutoff of 1e-10, plant GOSlim annotations [34] were transitively annotated using the GO terms from Arabidopsis. Hypothetical/expressed proteins, TE-related proteins, and proteins assigned with GO terms with "unknown" definitions were excluded from this analysis. The GOSlim assignment of Arabidopsis proteins was obtained form TAIR7 release [31].

### Identification of alternatively spliced genes

Approximately 780,000 rice EST sequences were released subsequent to the generation of the Release 4 gene models [33]. Thus, we utilized the PASA program [72] to re-annotate the gene models and comprehensively identify alternatively spliced genes with the latest set of rice transcript data. Alternative splicing information on Arabidopsis was obtained from TAIR7 release [31].

### Estimation of the age of the paralogous protein families

A multiple protein sequence alignment was obtained for each family using CLUSTALW with default parameter settings [67,68]. From each protein family of size *n*, all (*n*^2^-*n*)/2 pairwise alignments were extracted from the global family alignment, maintaining the position and length of all gaps. A maximum likelihood estimate of the number of synonymous substitutions per synonymous site (*d*_S_) was obtained for all pairwise alignments. All calculations were performed using the codon-based substitution model of Goodman and Yang [73] implemented in *codeml*, of the PAML package, version 3.15 [74], running in pairwise mode (runmode = -2), with codon equilibrium frequencies estimated from average nucleotide frequencies at each codon position (codonFreq = 2).

The age of a paralogous protein family is defined by the duplication that gave rise to its second member, and can be approximated by the divergence between the most distantly-related pair of genes in the family. Given the rate of synonymous substitutions in grasses, estimated to be ~6.5 × 10^-9 ^per site per year [75], the number of synonymous substitutions per site (*d*_S_) between the most divergent gene pair in a family can be converted into a divergence time, provided synonymous sites are not saturated (*d*_S _< ~1). In addition, peaks in the distribution of intra-family pairwise *d*_S _values suggest periods of family diversification. For each family, the distribution of pariwise *d*_S _values was determined, plotted within the range of 0 to 1.5, with bin size of 0.1. Both the modal bin of each distribution (usually resulting from the most ancient split in the family tree) and the largest modal value of *d*_S _< 1.5 (reflecting a burst in diversification within the last 100 MY) were recorded.

### Massively parallel signature sequencing data and mapping

A total of 106,521 significant (>3 TPM) and reliable (observed in more than one sequencing run) MPSS [32] tags were obtained from the Rice MPSS Project [32,76]. These MPSS tags are derived from nine treated or untreated organs/tissues including callus, leaf, seed, crown vegetative meristematic tissue, ovary, stigma, pollen, panicle and stem. To reduce background noise, the method of Haberer *et al*. [77] was used to remove tags if the total minimal abundance across all libraries was ≤ 10 TPM or if the tag was not detected at ≥ 5 TPM in at least a single library, resulting in a total of 74,748 tags for subsequent analyses. The final set of MPSS tags were searched against TIGR rice pseudomolecules [33] using the Vmatch program [78]. As tags can span an intron(s), MPSS tags were also searched against all the cDNA sequences of the annotated genes. MPSS tags that mapped to the anti-sense sequence of the annotated genes or that mapped to multiple locations of the genome were excluded, which is important to minimize false correlations among closely related paralogs. If a gene was associated with multiple MPSS tags, only the most 3' tag was used for the expression analysis. Paralogous genes that were associated with unique, reliable, and significant MPSS tags were analyzed. Pearson's Correlation Coefficient (*r*) was calculated for each gene pair to determine the expression correlation using the following formula [79]:

$$r=\frac{n\sum_{i=1}^{n}x_{i}y_{i}-\sum_{i=1}^{n}x_{i}\sum_{i=1}^{n}y_{i}}{\sqrt{[n\sum_{i=1}^{n}x_{i}^{2}-{\left(\sum_{i=1}^{n}x_{i}\right)}^{2}][n\sum_{i=1}^{n}y_{i}^{2}-{\left(\sum_{i=1}^{n}y_{i}\right)}^{2}]}},$$

Where *n *is the number of DNA libraries. ***X***_*i *_and ***Y***_*i *_represent the expression level of the gene pair in the *i*-th library.

### Tissue specific expression analysis

To determine if a gene was preferentially expressed in a specific tissue, we employed the PEM devised by Huminiecki *et al *[80]. PEM is defined as log_10_(*O*/*E*). Basically, it compares the observed (O) expression level in a given tissue with that of expected (E) level, assuming uniform expression across all tissues. The PEM value of the *i*-th gene in the *j*-th tissue was calculated as followed:

$$PEM_{i,j}=\log_{10}(x_{i,j}/(\sum_{k=1}^{m}x_{k,j}\sum_{l=1}^{n}x_{i,l}/\sum_{k=1}^{m}\sum_{l=1}^{n}x_{k,l}))$$

Where *m *and *n *represent the total number of MPSS-qualifying genes and tissues, respectively.*x*_*i*, *j *_is the expression level of the i-th gene in the j-th tissue.

## Abbreviations

BBI: Bowman-Birk Inhibitor; EST: Expressed Sequence Tag;FLcDNA: Full Length cDNA; MPSS: Massively Parallel Signature Sequencing; MY: Million Years; MYA: Million Years Ago; PEM: Preferential Expression Measure; TE: Transposable Element; TPM: Transcripts Per Million;

## Authors' contributions

HL designed the study, performed the analyses, and drafted the manuscript. SO participated in the analysis of GOSlim and made Additional file 3. KN and BM provided rice MPSS data. RS and JS carried out the age analysis of paralogous families. BH identified alternative splicing isoforms in rice. WZ identified the high confidence gene set in rice. XG participated in the analysis of alternative splicing. RB designed the study and drafted the manuscript. All authors read and approved the final manuscript.

## Supplementary Material

Additional File 1 Putative paralogous protein families within the rice genome. Click here for file

Additional File 2 Rice paralogous protein families with more than one hundred member proteins. Click here for file

Additional File 3 Distribution of non-transposable element-related genes in rice and Arabidopsis. In panel A, the 12 rice chromosomes are shown with paralogous gene family members plotted in blue while single copy genes are plotted in red. Segmental duplicated blocks are indicated in green and centromeres are denoted by a white box. In panel B, the five Arabidopsis chromosomes are shown with paralogous gene family members plotted in blue while single copy genes are plotted in red. Click here for file

Additional File 4 The age distribution of rice paralogous protein families. **A) **an expanded view of the age distribution. **B) **the enlarged distribution of rice paralogous protein families with largest *d*s ≤ 1.5. Click here for file

Additional File 5 Distribution of modal values under *d*_S _≤ 1.5 across rice paralogous protein families. Of all 3,865 paralogous protein families, 2,388 showed a peak under 1.5 in the distribution of all pairwise *d*_S _values and are plotted. Click here for file

Additional File 6 Pearson's correlation coefficient (*r*) versus *ds *values. **A) **0 <*d*_S _≤ 0.1; **B) **0.4 <*d*_S _≤ 0.5; **C) **1.0 <*d*_S _≤ 1.1; **D) **1.4 <*d*_S _≤ 1.5. Click here for file

Additional File 7 Schematic illustration of the domain composition of three related rice paralogous protein families: Family 3722, Family 3193, and Family 3856. Click here for file

Additional File 8 Expression abundance of the rice prolamin genes from Family 3722 and Family 3193 in 18 libraries which were associated with unique, reliable, and significant MPSS tags. Click here for file

Additional File 9 Expression abundance of genes from rice paralogous protein family Family 3856 (contained PF00234) in 18 libraries which were associated with unique, reliable, and significant MPSS tags. Click here for file

Additional File 10 Genome Browser view of the genes encoding rice prolamin proteins with TE-related genes inserted between putative tandem duplications. Click here for file

Additional File 11 Schematic illustration of the domain composition of two rice BBI-related paralogous protein families which have Pfam domain PF00228: Family 3328 and Family 1493. Click here for file

Additional File 12 Neighbor-Joining tree of the rice Bowman-Birk inhibitor protein family Family 3328. Click here for file

Additional File 13 Genome Browser view of the rice genes encoding BBI proteins on chromosome 1. Click here for file
