# Supplementary material for: Characterization of paralogous protein families in rice
Source: BMC Plant Biol. 2008 Feb 19;8:18. doi: 10.1186/1471-2229-8-18 (PMC2275729; doi:10.1186/1471-2229-8-18)
Supplement: Additional File 1 — Putative paralogous protein families within the rice genome. [file 1471-2229-8-18-S1.pdf]

Additional file 1. Putative paralogous protein families within the rice genome

Family_ID	Pub_locus	Chr
1	LOC_Os03g19350.1	3
1	LOC_Os07g48960.1	7
2	LOC_Os03g05730.1	3
2	LOC_Os10g30580.1	10
3	LOC_Os01g66330.1	1
3	LOC_Os05g45750.1	5
4	LOC_Os02g19150.1	2
4	LOC_Os02g35630.1	2
5	LOC_Os01g45450.1	1
5	LOC_Os03g38800.1	3
6	LOC_Os02g10640.1	2
6	LOC_Os02g11050.1	2
7	LOC_Os03g18690.1	3
7	LOC_Os07g49150.1	7
8	LOC_Os02g54340.1	2
8	LOC_Os06g09290.1	6
9	LOC_Os02g18180.1	2
9	LOC_Os11g34350.1	11
10	LOC_Os06g03770.1	6
10	LOC_Os07g28090.1	7
11	LOC_Os05g10730.1	5
11	LOC_Os06g06440.1	6
12	LOC_Os02g32690.1	2
12	LOC_Os09g16380.1	9
13	LOC_Os04g44610.1	4
13	LOC_Os10g35180.1	10
14	LOC_Os03g64200.1	3
14	LOC_Os12g22110.1	12
15	LOC_Os08g43120.1	8
15	LOC_Os12g13720.1	12
16	LOC_Os01g08260.1	1
16	LOC_Os07g33780.1	7
17	LOC_Os09g16330.1	9
17	LOC_Os09g16458.1	9
18	LOC_Os05g35320.1	5
18	LOC_Os09g08880.1	9
19	LOC_Os01g54910.1	1
19	LOC_Os02g18450.1	2
20	LOC_Os02g33850.1	2
20	LOC_Os03g44780.1	3
21	LOC_Os01g70310.1	1
21	LOC_Os03g04310.1	3
22	LOC_Os10g39750.1	10
22	LOC_Os11g32100.1	11
23	LOC_Os08g38210.1	8
23	LOC_Os09g29930.1	9
24	LOC_Os04g47040.1	4
24	LOC_Os11g15210.1	11
25	LOC_Os04g23550.1	4
25	LOC_Os09g24490.1	9

26	LOC_Os04g28280.1	4
26	LOC_Os09g33580.1	9
27	LOC_Os03g58330.1	3
27	LOC_Os06g08500.1	6
28	LOC_Os02g52150.1	2
28	LOC_Os06g11610.1	6
29	LOC_Os02g13130.1	2
29	LOC_Os08g01930.1	8
30	LOC_Os01g45990.1	1
30	LOC_Os07g07910.1	7
31	LOC_Os12g43940.1	12
31	LOC_Os12g43840.1	12
32	LOC_Os11g04600.1	11
32	LOC_Os12g04410.1	12
33	LOC_Os11g01720.1	11
33	LOC_Os11g11240.1	11
34	LOC_Os09g11120.1	9
34	LOC_Os09g15190.1	9
35	LOC_Os09g30414.1	9
35	LOC_Os09g30434.1	9
36	LOC_Os02g48900.1	2
36	LOC_Os06g20040.1	6
37	LOC_Os04g55080.1	4
37	LOC_Os12g28260.1	12
38	LOC_Os03g44440.1	3
38	LOC_Os09g38580.1	9
39	LOC_Os01g55200.1	1
39	LOC_Os02g14840.1	2
40	LOC_Os01g52070.1	1
40	LOC_Os06g14310.1	6
41	LOC_Os01g57370.1	1
41	LOC_Os05g42250.1	5
42	LOC_Os03g02970.1	3
42	LOC_Os03g38740.1	3
43	LOC_Os05g18850.1	5
43	LOC_Os06g25250.1	6
44	LOC_Os02g03410.1	2
44	LOC_Os07g22710.1	7
45	LOC_Os01g61590.1	1
45	LOC_Os05g39090.1	5
46	LOC_Os03g56300.1	3
46	LOC_Os05g06510.1	5
47	LOC_Os01g53020.1	1
47	LOC_Os05g45350.1	5
48	LOC_Os05g05310.1	5
48	LOC_Os12g34850.1	12
49	LOC_Os02g12800.1	2
49	LOC_Os06g37440.1	6
50	LOC_Os10g38340.1	10
50	LOC_Os10g38489.1	10
51	LOC_Os08g43680.1	8
51	LOC_Os09g37240.1	9

52	LOC_Os02g05450.1	2
52	LOC_Os06g12400.1	6
53	LOC_Os01g57890.1	1
53	LOC_Os08g19590.1	8
54	LOC_Os06g08900.1	6
54	LOC_Os06g09120.1	6
55	LOC_Os02g57190.1	2
55	LOC_Os03g53660.1	3
56	LOC_Os07g23570.1	7
56	LOC_Os07g44140.1	7
57	LOC_Os02g11020.1	2
57	LOC_Os05g33600.1	5
58	LOC_Os01g43710.1	1
58	LOC_Os01g43844.1	1
59	LOC_Os01g24780.1	1
59	LOC_Os03g25480.1	3
60	LOC_Os03g25490.1	3
60	LOC_Os07g44130.1	7
61	LOC_Os02g32770.1	2
61	LOC_Os07g11870.1	7
62	LOC_Os02g09260.1	2
62	LOC_Os02g38930.1	2
63	LOC_Os08g03090.1	8
63	LOC_Os08g03160.1	8
64	LOC_Os07g03780.1	7
64	LOC_Os09g09500.1	9
65	LOC_Os07g03950.1	7
65	LOC_Os07g18230.1	7
66	LOC_Os08g03100.1	8
66	LOC_Os08g03150.1	8
67	LOC_Os04g05560.1	4
67	LOC_Os04g06244.1	4
68	LOC_Os03g56180.1	3
68	LOC_Os09g09510.1	9
69	LOC_Os02g26160.1	2
69	LOC_Os05g03450.1	5
70	LOC_Os02g48210.1	2
70	LOC_Os08g40280.1	8
71	LOC_Os11g10640.1	11
71	LOC_Os11g10710.1	11
72	LOC_Os03g21620.1	3
72	LOC_Os07g48290.1	7
73	LOC_Os11g01200.1	11
73	LOC_Os12g01200.1	12
74	LOC_Os11g01140.1	11
74	LOC_Os12g01140.1	12
75	LOC_Os02g52850.1	2
75	LOC_Os09g28180.1	9
76	LOC_Os04g12600.1	4
76	LOC_Os04g39930.1	4
77	LOC_Os01g36500.1	1
77	LOC_Os10g10540.1	10

78	LOC_Os07g30410.1	7
78	LOC_Os07g30510.1	7
79	LOC_Os12g41490.1	12
79	LOC_Os12g41530.1	12
80	LOC_Os01g38900.1	1
80	LOC_Os05g03920.1	5
81	LOC_Os01g36790.1	1
81	LOC_Os10g04730.1	10
82	LOC_Os06g35160.1	6
82	LOC_Os12g41090.1	12
83	LOC_Os01g10870.1	1
83	LOC_Os01g55440.1	1
84	LOC_Os03g31000.1	3
84	LOC_Os03g31070.1	3
85	LOC_Os01g43350.1	1
85	LOC_Os05g50830.1	5
86	LOC_Os02g41480.1	2
86	LOC_Os04g30270.1	4
87	LOC_Os01g44110.1	1
87	LOC_Os01g47470.1	1
88	LOC_Os06g03610.1	6
88	LOC_Os06g22810.1	6
89	LOC_Os09g17970.1	9
89	LOC_Os09g19510.1	9
90	LOC_Os11g47110.1	11
90	LOC_Os11g47140.1	11
91	LOC_Os05g20150.1	5
91	LOC_Os05g25450.1	5
92	LOC_Os01g02580.1	1
92	LOC_Os01g02430.1	1
93	LOC_Os01g02800.1	1
93	LOC_Os01g02830.1	1
94	LOC_Os01g04460.1	1
94	LOC_Os01g04570.1	1
95	LOC_Os11g44430.1	11
95	LOC_Os11g44660.1	11
96	LOC_Os11g44250.1	11
96	LOC_Os11g44860.1	11
97	LOC_Os01g50370.1	1
97	LOC_Os02g21700.1	2
98	LOC_Os01g70970.1	1
98	LOC_Os04g30060.1	4
99	LOC_Os02g34600.1	2
99	LOC_Os10g41490.1	10
100	LOC_Os01g72790.1	1
100	LOC_Os11g13860.1	11
101	LOC_Os02g40860.1	2
101	LOC_Os10g33650.1	10
102	LOC_Os11g08950.1	11
102	LOC_Os12g08050.1	12
103	LOC_Os01g38950.1	1
103	LOC_Os05g51560.1	5

104	LOC_Os01g64970.1	1
104	LOC_Os04g59450.1	4
105	LOC_Os01g61410.1	1
105	LOC_Os07g37730.1	7
106	LOC_Os06g08450.1	6
106	LOC_Os06g44410.1	6
107	LOC_Os06g45900.1	6
107	LOC_Os06g45910.1	6
108	LOC_Os07g19560.1	7
108	LOC_Os09g12800.1	9
109	LOC_Os02g03890.1	2
109	LOC_Os04g30430.1	4
110	LOC_Os02g39700.1	2
110	LOC_Os04g42010.1	4
111	LOC_Os01g16090.1	1
111	LOC_Os04g54440.1	4
112	LOC_Os02g16940.1	2
112	LOC_Os04g03100.1	4
113	LOC_Os01g23740.1	1
113	LOC_Os05g06430.1	5
114	LOC_Os01g45320.1	1
114	LOC_Os03g28990.1	3
115	LOC_Os06g38040.1	6
115	LOC_Os11g45990.1	11
116	LOC_Os06g40960.1	6
116	LOC_Os08g39390.1	8
117	LOC_Os10g28330.1	10
117	LOC_Os12g07280.1	12
118	LOC_Os07g39960.1	7
118	LOC_Os12g39400.1	12
119	LOC_Os03g41390.1	3
119	LOC_Os11g25610.1	11
120	LOC_Os01g68104.1	1
120	LOC_Os01g68160.1	1
121	LOC_Os04g43300.1	4
121	LOC_Os05g43610.1	5
122	LOC_Os02g40810.1	2
122	LOC_Os09g33740.1	9
123	LOC_Os02g19804.1	2
123	LOC_Os06g21390.1	6
124	LOC_Os02g09060.1	2
124	LOC_Os04g55480.1	4
125	LOC_Os04g22240.1	4
125	LOC_Os05g01230.1	5
126	LOC_Os03g07130.1	3
126	LOC_Os08g01360.1	8
127	LOC_Os05g19480.1	5
127	LOC_Os07g41800.1	7
128	LOC_Os01g67850.1	1
128	LOC_Os07g46700.1	7
129	LOC_Os01g53500.1	1
129	LOC_Os05g45060.1	5

130	LOC_Os03g05570.1	3
130	LOC_Os03g28080.1	3
131	LOC_Os03g15000.1	3
131	LOC_Os10g13670.1	10
132	LOC_Os02g05660.1	2
132	LOC_Os06g48210.1	6
133	LOC_Os01g65220.1	1
133	LOC_Os03g58060.1	3
134	LOC_Os03g38100.1	3
134	LOC_Os05g28250.1	5
135	LOC_Os04g16890.1	4
135	LOC_Os11g15140.1	11
136	LOC_Os04g19060.1	4
136	LOC_Os08g21390.1	8
137	LOC_Os04g18310.1	4
137	LOC_Os07g15070.1	7
138	LOC_Os02g25670.1	2
138	LOC_Os12g09954.1	12
139	LOC_Os09g06290.1	9
139	LOC_Os12g08410.1	12
140	LOC_Os12g16390.1	12
140	LOC_Os12g27450.1	12
141	LOC_Os08g40670.1	8
141	LOC_Os11g24250.1	11
142	LOC_Os10g10520.1	10
142	LOC_Os11g39980.1	11
143	LOC_Os08g10200.1	8
143	LOC_Os08g30290.1	8
144	LOC_Os05g16020.1	5
144	LOC_Os06g34930.1	6
145	LOC_Os07g33150.1	7
145	LOC_Os12g13330.1	12
146	LOC_Os05g08880.1	5
146	LOC_Os06g25560.1	6
147	LOC_Os05g23240.1	5
147	LOC_Os09g01640.1	9
148	LOC_Os04g25300.1	4
148	LOC_Os04g26170.1	4
149	LOC_Os02g49670.1	2
149	LOC_Os11g44890.1	11
150	LOC_Os02g25310.1	2
150	LOC_Os06g28300.1	6
151	LOC_Os11g07850.1	11
151	LOC_Os12g07590.1	12
152	LOC_Os04g53800.1	4
152	LOC_Os04g53920.1	4
153	LOC_Os03g16220.1	3
153	LOC_Os07g37420.1	7
154	LOC_Os11g04490.1	11
154	LOC_Os12g04260.1	12
155	LOC_Os04g52400.1	4
155	LOC_Os05g04870.1	5

156	LOC_Os02g30060.1	2
156	LOC_Os03g16230.1	3
157	LOC_Os04g20070.1	4
157	LOC_Os11g25220.1	11
158	LOC_Os11g01154.1	11
158	LOC_Os12g01160.1	12
159	LOC_Os05g24550.1	5
159	LOC_Os12g25680.1	12
160	LOC_Os01g11830.1	1
160	LOC_Os01g24560.1	1
161	LOC_Os09g39070.1	9
161	LOC_Os09g39100.1	9
162	LOC_Os02g08010.1	2
162	LOC_Os03g52090.1	3
163	LOC_Os05g33390.1	5
163	LOC_Os06g47550.1	6
164	LOC_Os03g01670.1	3
164	LOC_Os11g29750.1	11
165	LOC_Os07g42030.1	7
165	LOC_Os07g42070.1	7
166	LOC_Os03g52460.1	3
166	LOC_Os07g13980.1	7
167	LOC_Os07g26170.1	7
167	LOC_Os09g16650.1	9
168	LOC_Os07g06190.1	7
168	LOC_Os07g06300.1	7
169	LOC_Os07g44480.1	7
169	LOC_Os07g44499.1	7
170	LOC_Os04g54340.1	4
170	LOC_Os08g08030.1	8
171	LOC_Os01g70360.1	1
171	LOC_Os11g31640.1	11
172	LOC_Os01g72240.1	1
172	LOC_Os01g72250.1	1
173	LOC_Os01g65260.1	1
173	LOC_Os05g35580.1	5
174	LOC_Os03g05980.1	3
174	LOC_Os11g38949.1	11
175	LOC_Os06g11990.1	6
175	LOC_Os11g08090.1	11
176	LOC_Os02g32160.1	2
176	LOC_Os05g30970.1	5
177	LOC_Os02g22130.1	2
177	LOC_Os07g01770.1	7
178	LOC_Os02g25060.1	2
178	LOC_Os09g36770.1	9
179	LOC_Os07g22640.1	7
179	LOC_Os08g20544.1	8
180	LOC_Os02g50550.1	2
180	LOC_Os06g13820.1	6
181	LOC_Os03g24180.1	3
181	LOC_Os07g46450.1	7

182	LOC_Os10g40590.1	10
182	LOC_Os12g18770.1	12
183	LOC_Os02g01270.1	2
183	LOC_Os10g31770.1	10
184	LOC_Os03g20650.1	3
184	LOC_Os07g48660.1	7
185	LOC_Os01g59760.1	1
185	LOC_Os02g52780.1	2
186	LOC_Os02g14910.1	2
186	LOC_Os03g21800.1	3
187	LOC_Os03g20310.1	3
187	LOC_Os05g41280.1	5
188	LOC_Os01g62900.1	1
188	LOC_Os05g38150.1	5
189	LOC_Os08g41830.1	8
189	LOC_Os12g25630.1	12
190	LOC_Os01g26872.1	1
190	LOC_Os01g27040.1	1
191	LOC_Os01g65850.1	1
191	LOC_Os06g08480.1	6
192	LOC_Os01g01060.1	1
192	LOC_Os11g29190.1	11
193	LOC_Os05g48380.1	5
193	LOC_Os09g12310.1	9
194	LOC_Os02g47590.1	2
194	LOC_Os08g15030.1	8
195	LOC_Os11g29390.1	11
195	LOC_Os12g26060.1	12
196	LOC_Os04g09390.1	4
196	LOC_Os04g41640.1	4
197	LOC_Os04g16780.1	4
197	LOC_Os12g34130.1	12
198	LOC_Os03g38000.1	3
198	LOC_Os07g41750.1	7
199	LOC_Os02g31040.1	2
199	LOC_Os04g32330.1	4
200	LOC_Os02g36840.1	2
200	LOC_Os04g25440.1	4
201	LOC_Os07g30469.1	7
201	LOC_Os07g30369.1	7
202	LOC_Os04g24850.1	4
202	LOC_Os04g25370.1	4
203	LOC_Os02g36830.1	2
203	LOC_Os04g25980.1	4
204	LOC_Os02g14570.1	2
204	LOC_Os05g45080.1	5
205	LOC_Os07g32020.1	7
205	LOC_Os07g32630.1	7
206	LOC_Os01g53420.1	1
206	LOC_Os01g53460.1	1
207	LOC_Os03g58430.1	3
207	LOC_Os07g08660.1	7

208	LOC_Os01g13310.1	1
208	LOC_Os02g47150.1	2
209	LOC_Os11g01530.1	11
209	LOC_Os12g01530.1	12
210	LOC_Os01g54080.1	1
210	LOC_Os05g44560.1	5
211	LOC_Os03g05820.1	3
211	LOC_Os06g04560.1	6
212	LOC_Os01g74580.1	1
212	LOC_Os02g10180.1	2
213	LOC_Os08g36980.1	8
213	LOC_Os09g28590.1	9
214	LOC_Os06g34440.1	6
214	LOC_Os11g36960.1	11
215	LOC_Os07g32880.1	7
215	LOC_Os10g17280.1	10
216	LOC_Os01g22490.1	1
216	LOC_Os05g06770.1	5
217	LOC_Os02g02710.1	2
217	LOC_Os06g05240.1	6
218	LOC_Os01g09760.1	1
218	LOC_Os03g43800.1	3
219	LOC_Os11g41610.1	11
219	LOC_Os12g32380.1	12
220	LOC_Os01g38180.1	1
220	LOC_Os01g38229.1	1
221	LOC_Os03g29460.1	3
221	LOC_Os07g42170.1	7
222	LOC_Os01g58070.1	1
222	LOC_Os04g53260.1	4
223	LOC_Os01g58100.1	1
223	LOC_Os04g53300.1	4
224	LOC_Os06g03720.1	6
224	LOC_Os06g14620.1	6
225	LOC_Os06g34420.1	6
225	LOC_Os07g41570.1	7
226	LOC_Os04g35420.1	4
226	LOC_Os07g48360.1	7
227	LOC_Os01g24690.1	1
227	LOC_Os04g42270.1	4
228	LOC_Os05g22940.1	5
228	LOC_Os10g21910.1	10
229	LOC_Os03g18310.1	3
229	LOC_Os05g07130.1	5
230	LOC_Os03g08220.1	3
230	LOC_Os04g37430.1	4
231	LOC_Os08g39840.1	8
231	LOC_Os08g39850.1	8
232	LOC_Os04g54940.1	4
232	LOC_Os10g35580.1	10
233	LOC_Os03g18130.1	3
233	LOC_Os06g15420.1	6

234	LOC_Os04g16720.1	4
234	LOC_Os10g21340.1	10
235	LOC_Os02g56100.1	2
235	LOC_Os06g07210.1	6
236	LOC_Os03g08440.1	3
236	LOC_Os07g42450.1	7
237	LOC_Os02g05510.1	2
237	LOC_Os03g47970.1	3
238	LOC_Os01g47360.1	1
238	LOC_Os05g49280.1	5
239	LOC_Os06g11180.1	6
239	LOC_Os06g11190.1	6
240	LOC_Os01g63270.1	1
240	LOC_Os03g55090.1	3
241	LOC_Os05g33020.1	5
241	LOC_Os08g15460.1	8
242	LOC_Os01g54420.1	1
242	LOC_Os12g07880.1	12
243	LOC_Os02g32120.1	2
243	LOC_Os04g32660.1	4
244	LOC_Os02g08130.1	2
244	LOC_Os04g55410.1	4
245	LOC_Os07g40660.1	7
245	LOC_Os08g34570.1	8
246	LOC_Os06g44460.1	6
246	LOC_Os08g34720.1	8
247	LOC_Os07g31380.1	7
247	LOC_Os10g35960.1	10
248	LOC_Os03g31750.1	3
248	LOC_Os05g33570.1	5
249	LOC_Os06g02144.1	6
249	LOC_Os11g29400.1	11
250	LOC_Os11g01730.1	11
250	LOC_Os12g01730.1	12
251	LOC_Os04g52870.1	4
251	LOC_Os11g07970.1	11
252	LOC_Os02g11830.1	2
252	LOC_Os06g05180.1	6
253	LOC_Os06g44030.1	6
253	LOC_Os07g10520.1	7
254	LOC_Os10g01540.1	10
254	LOC_Os11g38170.1	11
255	LOC_Os02g33630.1	2
255	LOC_Os04g34080.1	4
256	LOC_Os03g02440.1	3
256	LOC_Os04g48010.1	4
257	LOC_Os02g49090.1	2
257	LOC_Os06g19660.1	6
258	LOC_Os01g09252.1	1
258	LOC_Os03g05210.1	3
259	LOC_Os01g58000.1	1
259	LOC_Os12g19430.1	12

260	LOC_Os11g20790.1	11
260	LOC_Os12g13380.1	12
261	LOC_Os01g61210.1	1
261	LOC_Os02g13330.1	2
262	LOC_Os01g72320.1	1
262	LOC_Os05g31920.1	5
263	LOC_Os03g61120.1	3
263	LOC_Os03g15780.1	3
264	LOC_Os01g10940.1	1
264	LOC_Os07g29440.1	7
265	LOC_Os05g07090.1	5
265	LOC_Os05g03480.1	5
266	LOC_Os08g41630.1	8
266	LOC_Os09g32740.1	9
267	LOC_Os01g72800.1	1
267	LOC_Os08g37444.1	8
268	LOC_Os01g53280.1	1
268	LOC_Os05g45220.1	5
269	LOC_Os01g16470.1	1
269	LOC_Os11g10420.1	11
270	LOC_Os05g08810.1	5
270	LOC_Os08g21590.1	8
271	LOC_Os04g19740.1	4
271	LOC_Os06g04270.1	6
272	LOC_Os06g12030.1	6
272	LOC_Os10g34170.1	10
273	LOC_Os02g18380.1	2
273	LOC_Os10g41470.1	10
274	LOC_Os01g39010.1	1
274	LOC_Os01g59060.1	1
275	LOC_Os08g07840.1	8
275	LOC_Os08g07850.1	8
276	LOC_Os01g06630.1	1
276	LOC_Os05g28210.1	5
277	LOC_Os03g59530.1	3
277	LOC_Os07g11010.1	7
278	LOC_Os01g45274.1	1
278	LOC_Os09g28910.1	9
279	LOC_Os05g41880.1	5
279	LOC_Os10g36530.1	10
280	LOC_Os05g19270.1	5
280	LOC_Os09g24220.1	9
281	LOC_Os02g27060.1	2
281	LOC_Os09g37250.1	9
282	LOC_Os01g08970.1	1
282	LOC_Os05g08970.1	5
283	LOC_Os03g49500.1	3
283	LOC_Os05g06320.1	5
284	LOC_Os02g06530.1	2
284	LOC_Os03g60780.1	3
285	LOC_Os01g06470.1	1
285	LOC_Os07g48880.1	7

286	LOC_Os02g49520.1	2
286	LOC_Os06g16410.1	6
287	LOC_Os01g60860.1	1
287	LOC_Os02g13960.1	2
288	LOC_Os03g02580.1	3
288	LOC_Os07g37350.1	7
289	LOC_Os05g49810.1	5
289	LOC_Os10g41360.1	10
290	LOC_Os09g03890.1	9
290	LOC_Os09g38390.1	9
291	LOC_Os02g43920.1	2
291	LOC_Os06g46540.1	6
292	LOC_Os06g06490.1	6
292	LOC_Os06g06470.1	6
293	LOC_Os04g35690.1	4
293	LOC_Os07g06710.1	7
294	LOC_Os02g43020.1	2
294	LOC_Os04g45480.1	4
295	LOC_Os01g42960.1	1
295	LOC_Os02g51730.1	2
296	LOC_Os06g12450.1	6
296	LOC_Os10g30156.1	10
297	LOC_Os03g60939.1	3
297	LOC_Os03g60700.1	3
298	LOC_Os02g51060.1	2
298	LOC_Os10g26630.1	10
299	LOC_Os01g56130.1	1
299	LOC_Os05g43530.1	5
300	LOC_Os04g33220.1	4
300	LOC_Os04g30910.1	4
301	LOC_Os04g18090.1	4
301	LOC_Os06g04020.1	6
302	LOC_Os05g51850.1	5
302	LOC_Os07g08710.1	7
303	LOC_Os01g36620.1	1
303	LOC_Os06g38520.1	6
304	LOC_Os01g19450.1	1
304	LOC_Os07g38970.1	7
305	LOC_Os02g13140.1	2
305	LOC_Os12g37640.1	12
306	LOC_Os01g63420.1	1
306	LOC_Os05g37690.1	5
307	LOC_Os11g01780.1	11
307	LOC_Os12g01760.1	12
308	LOC_Os01g58530.1	1
308	LOC_Os11g29090.1	11
309	LOC_Os01g57310.1	1
309	LOC_Os01g57340.1	1
310	LOC_Os09g10054.1	9
310	LOC_Os09g14060.1	9
311	LOC_Os04g39460.1	4
311	LOC_Os11g36410.1	11

312	LOC_Os12g29690.1	12
312	LOC_Os12g29710.1	12
313	LOC_Os09g15390.1	9
313	LOC_Os12g30760.1	12
314	LOC_Os11g39190.1	11
314	LOC_Os11g39330.1	11
315	LOC_Os04g22090.1	4
315	LOC_Os06g39360.1	6
316	LOC_Os05g12140.1	5
316	LOC_Os12g05930.1	12
317	LOC_Os04g08370.1	4
317	LOC_Os04g08390.1	4
318	LOC_Os02g40260.1	2
318	LOC_Os04g57430.1	4
319	LOC_Os03g53090.1	3
319	LOC_Os12g06110.1	12
320	LOC_Os06g37820.1	6
320	LOC_Os11g43770.1	11
321	LOC_Os04g31240.1	4
321	LOC_Os12g02589.1	12
322	LOC_Os02g38050.1	2
322	LOC_Os04g40090.1	4
323	LOC_Os11g25780.1	11
323	LOC_Os11g25800.1	11
324	LOC_Os02g32350.1	2
324	LOC_Os04g32960.1	4
325	LOC_Os03g06190.1	3
325	LOC_Os10g30100.1	10
326	LOC_Os03g18510.1	3
326	LOC_Os07g49220.1	7
327	LOC_Os03g31690.1	3
327	LOC_Os07g39690.1	7
328	LOC_Os02g03850.1	2
328	LOC_Os03g03450.1	3
329	LOC_Os01g47450.1	1
329	LOC_Os06g21380.1	6
330	LOC_Os01g66170.1	1
330	LOC_Os07g44020.1	7
331	LOC_Os02g48780.1	2
331	LOC_Os11g31060.1	11
332	LOC_Os01g20860.1	1
332	LOC_Os06g44060.1	6
333	LOC_Os02g35070.1	2
333	LOC_Os04g54350.1	4
334	LOC_Os11g04930.1	11
334	LOC_Os12g04930.1	12
335	LOC_Os03g20460.1	3
335	LOC_Os12g33100.1	12
336	LOC_Os07g46690.1	7
336	LOC_Os11g05130.1	11
337	LOC_Os03g19020.1	3
337	LOC_Os06g17280.1	6

338	LOC_Os11g16580.1	11
338	LOC_Os12g10850.1	12
339	LOC_Os11g01380.1	11
339	LOC_Os12g01390.1	12
340	LOC_Os01g53650.1	1
340	LOC_Os05g10670.1	5
341	LOC_Os05g48960.1	5
341	LOC_Os09g31482.1	9
342	LOC_Os01g09620.1	1
342	LOC_Os05g45020.1	5
343	LOC_Os02g05700.1	2
343	LOC_Os06g47890.1	6
344	LOC_Os03g07160.1	3
344	LOC_Os10g26990.1	10
345	LOC_Os03g27250.1	3
345	LOC_Os11g36450.1	11
346	LOC_Os04g25140.1	4
346	LOC_Os04g26240.1	4
347	LOC_Os01g40240.1	1
347	LOC_Os05g40450.1	5
348	LOC_Os01g56750.1	1
348	LOC_Os05g46050.1	5
349	LOC_Os08g06710.1	8
349	LOC_Os11g38140.1	11
350	LOC_Os12g05709.1	12
350	LOC_Os12g05609.1	12
351	LOC_Os06g05620.1	6
351	LOC_Os06g05580.1	6
352	LOC_Os11g36350.1	11
352	LOC_Os11g38180.1	11
353	LOC_Os11g38100.1	11
353	LOC_Os11g38120.1	11
354	LOC_Os07g48940.1	7
354	LOC_Os08g34890.1	8
355	LOC_Os01g47050.1	1
355	LOC_Os02g15950.1	2
356	LOC_Os04g19920.1	4
356	LOC_Os11g42310.1	11
357	LOC_Os07g13900.1	7
357	LOC_Os07g18510.1	7
358	LOC_Os08g35930.1	8
358	LOC_Os08g35880.1	8
359	LOC_Os01g41340.1	1
359	LOC_Os06g29700.1	6
360	LOC_Os11g33180.1	11
360	LOC_Os11g33210.1	11
361	LOC_Os01g37260.1	1
361	LOC_Os07g26000.1	7
362	LOC_Os11g09970.1	11
362	LOC_Os11g15620.1	11
363	LOC_Os11g10340.1	11
363	LOC_Os11g10240.1	11

364	LOC_Os07g03100.1	7
364	LOC_Os11g37340.1	11
365	LOC_Os01g32640.1	1
365	LOC_Os07g33400.1	7
366	LOC_Os04g13170.1	4
366	LOC_Os04g13150.1	4
367	LOC_Os09g08550.1	9
367	LOC_Os09g08620.1	9
368	LOC_Os03g12200.1	3
368	LOC_Os03g12190.1	3
369	LOC_Os07g04790.1	7
369	LOC_Os07g31680.1	7
370	LOC_Os09g15560.1	9
370	LOC_Os10g10410.1	10
371	LOC_Os05g25580.1	5
371	LOC_Os10g04780.1	10
372	LOC_Os10g03910.1	10
372	LOC_Os10g03930.1	10
373	LOC_Os10g03730.1	10
373	LOC_Os10g03750.1	10
374	LOC_Os04g32460.1	4
374	LOC_Os11g31620.1	11
375	LOC_Os04g11450.1	4
375	LOC_Os10g25210.1	10
376	LOC_Os02g48300.1	2
376	LOC_Os04g40760.1	4
377	LOC_Os01g40160.1	1
377	LOC_Os04g40780.1	4
378	LOC_Os09g39000.1	9
378	LOC_Os10g36310.1	10
379	LOC_Os10g34300.1	10
379	LOC_Os10g34340.1	10
380	LOC_Os01g14270.1	1
380	LOC_Os05g01630.1	5
381	LOC_Os07g02930.1	7
381	LOC_Os10g05000.1	10
382	LOC_Os02g16760.1	2
382	LOC_Os09g39050.1	9
383	LOC_Os07g02290.1	7
383	LOC_Os07g12560.1	7
384	LOC_Os12g33830.1	12
384	LOC_Os12g34240.1	12
385	LOC_Os04g35990.1	4
385	LOC_Os04g36000.1	4
386	LOC_Os02g06470.1	2
386	LOC_Os08g31690.1	8
387	LOC_Os01g70670.1	1
387	LOC_Os04g56460.1	4
388	LOC_Os07g46160.1	7
388	LOC_Os08g03530.1	8
389	LOC_Os03g57854.1	3
389	LOC_Os07g07270.1	7

390	LOC_Os08g03510.1	8
390	LOC_Os08g25240.1	8
391	LOC_Os08g31420.1	8
391	LOC_Os10g29410.1	10
392	LOC_Os10g29810.1	10
392	LOC_Os10g29950.1	10
393	LOC_Os01g66890.1	1
393	LOC_Os04g40630.1	4
394	LOC_Os04g53430.1	4
394	LOC_Os10g29120.1	10
395	LOC_Os04g33700.1	4
395	LOC_Os05g04600.1	5
396	LOC_Os02g52390.1	2
396	LOC_Os06g41990.1	6
397	LOC_Os01g09560.1	1
397	LOC_Os01g73550.1	1
398	LOC_Os04g32020.1	4
398	LOC_Os07g49520.1	7
399	LOC_Os08g25390.1	8
399	LOC_Os09g12290.1	9
400	LOC_Os03g63330.1	3
400	LOC_Os07g20544.1	7
401	LOC_Os04g18200.1	4
401	LOC_Os04g48540.1	4
402	LOC_Os01g72340.1	1
402	LOC_Os05g32530.1	5
403	LOC_Os06g15380.1	6
403	LOC_Os06g15390.1	6
404	LOC_Os03g07840.1	3
404	LOC_Os03g49220.1	3
405	LOC_Os05g37410.1	5
405	LOC_Os05g37350.1	5
406	LOC_Os11g03470.1	11
406	LOC_Os12g03190.1	12
407	LOC_Os07g02130.1	7
407	LOC_Os10g40570.1	10
408	LOC_Os01g16520.1	1
408	LOC_Os10g22380.1	10
409	LOC_Os01g09000.1	1
409	LOC_Os05g08990.1	5
410	LOC_Os01g06510.1	1
410	LOC_Os05g07030.1	5
411	LOC_Os05g29760.1	5
411	LOC_Os09g12560.1	9
412	LOC_Os11g19770.1	11
412	LOC_Os12g13320.1	12
413	LOC_Os11g11070.1	11
413	LOC_Os12g09120.1	12
414	LOC_Os05g50660.1	5
414	LOC_Os11g06040.1	11
415	LOC_Os04g57520.1	4
415	LOC_Os08g43300.1	8

416	LOC_Os04g39040.1	4
416	LOC_Os06g42810.1	6
417	LOC_Os02g11890.1	2
417	LOC_Os03g52650.1	3
418	LOC_Os03g06160.1	3
418	LOC_Os10g30190.1	10
419	LOC_Os04g20800.1	4
419	LOC_Os09g08730.1	9
420	LOC_Os01g55300.1	1
420	LOC_Os03g42010.1	3
421	LOC_Os03g40180.1	3
421	LOC_Os05g19370.1	5
422	LOC_Os02g15900.1	2
422	LOC_Os05g48410.1	5
423	LOC_Os05g01110.1	5
423	LOC_Os07g36090.1	7
424	LOC_Os03g01900.1	3
424	LOC_Os10g27190.1	10
425	LOC_Os03g07300.1	3
425	LOC_Os09g32810.1	9
426	LOC_Os02g40070.1	2
426	LOC_Os04g42570.1	4
427	LOC_Os01g67410.1	1
427	LOC_Os11g19060.1	11
428	LOC_Os03g05806.1	3
428	LOC_Os06g50360.1	6
429	LOC_Os04g39030.1	4
429	LOC_Os06g15370.1	6
430	LOC_Os02g46460.1	2
430	LOC_Os10g40600.1	10
431	LOC_Os01g65190.1	1
431	LOC_Os10g33170.1	10
432	LOC_Os01g01360.1	1
432	LOC_Os07g41250.1	7
433	LOC_Os06g49250.1	6
433	LOC_Os10g02210.1	10
434	LOC_Os01g55600.1	1
434	LOC_Os01g55610.1	1
435	LOC_Os02g47090.1	2
435	LOC_Os05g27304.1	5
436	LOC_Os01g65140.1	1
436	LOC_Os05g35650.1	5
437	LOC_Os01g67630.1	1
437	LOC_Os11g18110.1	11
438	LOC_Os05g38810.1	5
438	LOC_Os07g49380.1	7
439	LOC_Os02g40770.1	2
439	LOC_Os02g47900.1	2
440	LOC_Os05g26840.1	5
440	LOC_Os11g24060.1	11
441	LOC_Os01g67134.1	1
441	LOC_Os03g61260.1	3

442	LOC_Os02g55140.1	2
442	LOC_Os12g24650.1	12
443	LOC_Os08g40610.1	8
443	LOC_Os09g32270.1	9
444	LOC_Os04g40830.1	4
444	LOC_Os05g10580.1	5
445	LOC_Os02g04170.1	2
445	LOC_Os07g04240.1	7
446	LOC_Os03g49940.1	3
446	LOC_Os10g35140.1	10
447	LOC_Os01g07730.1	1
447	LOC_Os05g07870.1	5
448	LOC_Os08g25624.1	8
448	LOC_Os09g12600.1	9
449	LOC_Os05g01580.1	5
449	LOC_Os06g49500.1	6
450	LOC_Os01g10970.1	1
450	LOC_Os01g10990.1	1
451	LOC_Os06g01972.1	6
451	LOC_Os06g01966.1	6
452	LOC_Os04g34530.1	4
452	LOC_Os11g04140.1	11
453	LOC_Os01g16540.1	1
453	LOC_Os07g39780.1	7
454	LOC_Os04g35480.1	4
454	LOC_Os11g38030.1	11
455	LOC_Os01g70170.1	1
455	LOC_Os08g05830.1	8
456	LOC_Os02g36340.1	2
456	LOC_Os05g38570.1	5
457	LOC_Os12g10340.1	12
457	LOC_Os12g10400.1	12
458	LOC_Os07g02660.1	7
458	LOC_Os11g06210.1	11
459	LOC_Os10g04570.1	10
459	LOC_Os11g06220.1	11
460	LOC_Os12g10360.1	12
460	LOC_Os12g10330.1	12
461	LOC_Os11g30110.1	11
461	LOC_Os11g30050.1	11
462	LOC_Os11g19160.1	11
462	LOC_Os11g19210.1	11
463	LOC_Os01g19220.1	1
463	LOC_Os11g47350.1	11
464	LOC_Os06g44230.1	6
464	LOC_Os09g07120.1	9
465	LOC_Os01g11260.1	1
465	LOC_Os07g46640.1	7
466	LOC_Os03g57760.1	3
466	LOC_Os06g09850.1	6
467	LOC_Os04g58620.1	4
467	LOC_Os12g42300.1	12

468	LOC_Os01g60140.1	1
468	LOC_Os02g58660.1	2
469	LOC_Os03g18410.1	3
469	LOC_Os09g02284.1	9
470	LOC_Os03g61920.1	3
470	LOC_Os04g10400.1	4
471	LOC_Os01g05820.1	1
471	LOC_Os01g05810.1	1
472	LOC_Os04g48820.1	4
472	LOC_Os07g06440.1	7
473	LOC_Os01g21820.1	1
473	LOC_Os11g08980.1	11
474	LOC_Os08g09210.1	8
474	LOC_Os12g09540.1	12
475	LOC_Os04g53810.1	4
475	LOC_Os04g53850.1	4
476	LOC_Os03g36540.1	3
476	LOC_Os03g59640.1	3
477	LOC_Os01g16930.1	1
477	LOC_Os03g40700.1	3
478	LOC_Os03g27260.1	3
478	LOC_Os07g42950.1	7
479	LOC_Os06g08880.1	6
479	LOC_Os09g25940.1	9
480	LOC_Os03g60130.1	3
480	LOC_Os07g12630.1	7
481	LOC_Os04g46370.1	4
481	LOC_Os04g58600.1	4
482	LOC_Os03g42110.1	3
482	LOC_Os03g55280.1	3
483	LOC_Os01g72880.1	1
483	LOC_Os02g37920.1	2
484	LOC_Os03g06900.1	3
484	LOC_Os09g32450.1	9
485	LOC_Os04g40540.1	4
485	LOC_Os08g44280.1	8
486	LOC_Os01g56620.1	1
486	LOC_Os05g43370.1	5
487	LOC_Os06g43610.1	6
487	LOC_Os08g21330.1	8
488	LOC_Os03g04750.1	3
488	LOC_Os10g32820.1	10
489	LOC_Os01g62350.1	1
489	LOC_Os05g38520.1	5
490	LOC_Os02g37862.1	2
490	LOC_Os04g39700.1	4
491	LOC_Os01g66520.1	1
491	LOC_Os07g06820.1	7
492	LOC_Os02g35060.1	2
492	LOC_Os06g04150.1	6
493	LOC_Os02g16670.1	2
493	LOC_Os02g52470.1	2

494	LOC_Os02g19770.1	2
494	LOC_Os06g23440.1	6
495	LOC_Os06g23114.1	6
495	LOC_Os06g23140.1	6
496	LOC_Os04g04950.1	4
496	LOC_Os04g40040.1	4
497	LOC_Os02g50350.1	2
497	LOC_Os04g57950.1	4
498	LOC_Os03g51250.1	3
498	LOC_Os08g34780.1	8
499	LOC_Os07g31280.1	7
499	LOC_Os10g41520.1	10
500	LOC_Os01g42650.1	1
500	LOC_Os06g05080.1	6
501	LOC_Os03g16690.1	3
501	LOC_Os08g40590.1	8
502	LOC_Os03g49770.1	3
502	LOC_Os08g05870.1	8
503	LOC_Os07g05480.1	7
503	LOC_Os09g30340.1	9
504	LOC_Os03g22460.1	3
504	LOC_Os03g46490.1	3
505	LOC_Os03g62630.1	3
505	LOC_Os12g37610.1	12
506	LOC_Os03g12910.1	3
506	LOC_Os03g12900.1	3
507	LOC_Os01g39960.1	1
507	LOC_Os02g09750.1	2
508	LOC_Os02g57590.1	2
508	LOC_Os05g08360.1	5
509	LOC_Os06g20430.1	6
509	LOC_Os09g30474.1	9
510	LOC_Os03g21940.1	3
510	LOC_Os03g38260.1	3
511	LOC_Os02g57670.1	2
511	LOC_Os05g45270.1	5
512	LOC_Os01g60790.1	1
512	LOC_Os05g39960.1	5
513	LOC_Os03g37970.1	3
513	LOC_Os06g02510.1	6
514	LOC_Os03g10450.1	3
514	LOC_Os08g41910.1	8
515	LOC_Os05g46200.1	5
515	LOC_Os12g24184.1	12
516	LOC_Os08g43570.1	8
516	LOC_Os09g36810.1	9
517	LOC_Os01g39830.1	1
517	LOC_Os03g06940.1	3
518	LOC_Os02g12730.1	2
518	LOC_Os06g37560.1	6
519	LOC_Os02g53680.1	2
519	LOC_Os03g11540.1	3

520	LOC_Os01g13700.1	1
520	LOC_Os06g01370.1	6
521	LOC_Os05g18280.1	5
521	LOC_Os11g08830.1	11
522	LOC_Os01g19480.1	1
522	LOC_Os03g61950.1	3
523	LOC_Os01g65400.1	1
523	LOC_Os03g12610.1	3
524	LOC_Os04g28620.1	4
524	LOC_Os08g20200.1	8
525	LOC_Os02g58790.1	2
525	LOC_Os04g53830.1	4
526	LOC_Os06g39240.1	6
526	LOC_Os08g27850.1	8
527	LOC_Os05g07050.1	5
527	LOC_Os06g07080.1	6
528	LOC_Os02g03080.1	2
528	LOC_Os07g03230.1	7
529	LOC_Os02g38810.1	2
529	LOC_Os04g40850.1	4
530	LOC_Os10g26130.1	10
530	LOC_Os12g34860.1	12
531	LOC_Os06g13660.1	6
531	LOC_Os10g10244.1	10
532	LOC_Os01g50010.1	1
532	LOC_Os06g45050.1	6
533	LOC_Os04g26320.1	4
533	LOC_Os12g12720.1	12
534	LOC_Os12g09700.1	12
534	LOC_Os12g14440.1	12
535	LOC_Os01g46380.1	1
535	LOC_Os05g49800.1	5
536	LOC_Os01g61020.1	1
536	LOC_Os09g02410.1	9
537	LOC_Os01g03650.1	1
537	LOC_Os01g61400.1	1
538	LOC_Os01g54390.1	1
538	LOC_Os04g56670.1	4
539	LOC_Os08g35060.1	8
539	LOC_Os09g25260.1	9
540	LOC_Os08g42740.1	8
540	LOC_Os09g38640.1	9
541	LOC_Os02g46720.1	2
541	LOC_Os04g50740.1	4
542	LOC_Os03g05880.1	3
542	LOC_Os07g30960.1	7
543	LOC_Os03g21250.1	3
543	LOC_Os12g02910.1	12
544	LOC_Os06g14750.1	6
544	LOC_Os09g10650.1	9
545	LOC_Os11g04840.1	11
545	LOC_Os12g04700.1	12

546	LOC_Os03g15550.1	3
546	LOC_Os03g24170.1	3
547	LOC_Os04g49560.1	4
547	LOC_Os09g38970.1	9
548	LOC_Os01g25270.1	1
548	LOC_Os09g07780.1	9
549	LOC_Os07g39910.1	7
549	LOC_Os11g37330.1	11
550	LOC_Os03g04390.1	3
550	LOC_Os03g62400.1	3
551	LOC_Os08g25280.1	8
551	LOC_Os10g40920.1	10
552	LOC_Os01g68610.1	1
552	LOC_Os08g37520.1	8
553	LOC_Os01g62220.1	1
553	LOC_Os10g26070.1	10
554	LOC_Os08g13990.1	8
554	LOC_Os11g17530.1	11
555	LOC_Os03g11700.1	3
555	LOC_Os07g44170.1	7
556	LOC_Os02g40750.1	2
556	LOC_Os07g19400.1	7
557	LOC_Os04g38930.1	4
557	LOC_Os06g09880.1	6
558	LOC_Os01g19380.1	1
558	LOC_Os04g57940.1	4
559	LOC_Os05g45760.1	5
559	LOC_Os10g10170.1	10
560	LOC_Os05g30240.1	5
560	LOC_Os11g24570.1	11
561	LOC_Os04g46010.1	4
561	LOC_Os07g40750.1	7
562	LOC_Os01g19490.1	1
562	LOC_Os01g19548.1	1
563	LOC_Os04g25410.1	4
563	LOC_Os10g35730.1	10
564	LOC_Os11g01210.1	11
564	LOC_Os12g01210.1	12
565	LOC_Os04g36840.1	4
565	LOC_Os06g30420.1	6
566	LOC_Os05g47510.1	5
566	LOC_Os08g41380.1	8
567	LOC_Os03g52620.1	3
567	LOC_Os06g03530.1	6
568	LOC_Os01g07340.1	1
568	LOC_Os02g02950.1	2
569	LOC_Os01g63580.1	1
569	LOC_Os05g38350.1	5
570	LOC_Os10g13940.1	10
570	LOC_Os12g01580.1	12
571	LOC_Os02g10310.1	2
571	LOC_Os03g61330.1	3

572	LOC_Os07g06890.1	7
572	LOC_Os07g08950.1	7
573	LOC_Os03g49400.1	3
573	LOC_Os07g06130.1	7
574	LOC_Os04g53230.1	4
574	LOC_Os06g04380.1	6
575	LOC_Os07g38560.1	7
575	LOC_Os11g41650.1	11
576	LOC_Os01g60660.1	1
576	LOC_Os04g23820.1	4
577	LOC_Os01g47340.1	1
577	LOC_Os05g49300.1	5
578	LOC_Os01g17430.1	1
578	LOC_Os11g07280.1	11
579	LOC_Os05g28860.1	5
579	LOC_Os07g32890.1	7
580	LOC_Os06g43900.1	6
580	LOC_Os10g42860.1	10
581	LOC_Os01g08760.1	1
581	LOC_Os09g26700.1	9
582	LOC_Os01g51920.1	1
582	LOC_Os05g45880.1	5
583	LOC_Os01g31760.1	1
583	LOC_Os09g32760.1	9
584	LOC_Os03g16960.1	3
584	LOC_Os03g16950.1	3
585	LOC_Os01g56840.1	1
585	LOC_Os04g12220.1	4
586	LOC_Os03g09250.1	3
586	LOC_Os10g22450.1	10
587	LOC_Os07g01020.1	7
587	LOC_Os10g01080.1	10
588	LOC_Os09g29420.1	9
588	LOC_Os10g05220.1	10
589	LOC_Os01g65350.1	1
589	LOC_Os05g35520.1	5
590	LOC_Os01g12530.1	1
590	LOC_Os06g09890.1	6
591	LOC_Os01g34700.1	1
591	LOC_Os05g35470.1	5
592	LOC_Os03g53230.1	3
592	LOC_Os04g02050.1	4
593	LOC_Os01g39650.1	1
593	LOC_Os03g18830.1	3
594	LOC_Os05g47880.1	5
594	LOC_Os09g26300.1	9
595	LOC_Os01g20840.1	1
595	LOC_Os02g54010.1	2
596	LOC_Os06g14490.1	6
596	LOC_Os09g39580.1	9
597	LOC_Os01g46240.1	1
597	LOC_Os05g49830.1	5

598	LOC_Os02g43700.1	2
598	LOC_Os08g04800.1	8
599	LOC_Os02g42170.1	2
599	LOC_Os11g04940.1	11
600	LOC_Os04g41300.1	4
600	LOC_Os07g38300.1	7
601	LOC_Os03g22340.1	3
601	LOC_Os07g47710.1	7
602	LOC_Os01g19840.1	1
602	LOC_Os05g28750.1	5
603	LOC_Os01g48770.1	1
603	LOC_Os05g48320.1	5
604	LOC_Os03g07290.1	3
604	LOC_Os10g26690.1	10
605	LOC_Os10g32400.1	10
605	LOC_Os10g32348.1	10
606	LOC_Os04g46890.1	4
606	LOC_Os07g04850.1	7
607	LOC_Os03g22730.1	3
607	LOC_Os03g22740.1	3
608	LOC_Os02g15970.1	2
608	LOC_Os11g47930.1	11
609	LOC_Os07g39070.1	7
609	LOC_Os12g07020.1	12
610	LOC_Os02g01070.1	2
610	LOC_Os03g61050.1	3
611	LOC_Os02g34990.1	2
611	LOC_Os08g02500.1	8
612	LOC_Os08g42600.1	8
612	LOC_Os11g32900.1	11
613	LOC_Os02g57050.1	2
613	LOC_Os11g16430.1	11
614	LOC_Os07g05380.1	7
614	LOC_Os09g04800.1	9
615	LOC_Os03g21550.1	3
615	LOC_Os07g48300.1	7
616	LOC_Os06g48350.1	6
616	LOC_Os09g15770.1	9
617	LOC_Os05g24580.1	5
617	LOC_Os08g20270.1	8
618	LOC_Os02g09460.1	2
618	LOC_Os06g43140.1	6
619	LOC_Os01g48210.1	1
619	LOC_Os06g45800.1	6
620	LOC_Os02g02970.1	2
620	LOC_Os10g11034.1	10
621	LOC_Os03g11240.1	3
621	LOC_Os12g18530.1	12
622	LOC_Os01g17330.1	1
622	LOC_Os07g44620.1	7
623	LOC_Os03g52970.1	3
623	LOC_Os09g25370.1	9

624	LOC_Os05g28940.1	5
624	LOC_Os07g43470.1	7
625	LOC_Os02g35130.1	2
625	LOC_Os04g36030.1	4
626	LOC_Os03g56840.1	3
626	LOC_Os08g09940.1	8
627	LOC_Os06g09570.1	6
627	LOC_Os07g01920.1	7
628	LOC_Os02g40880.1	2
628	LOC_Os04g43540.1	4
629	LOC_Os05g42010.1	5
629	LOC_Os08g43230.1	8
630	LOC_Os06g33980.1	6
630	LOC_Os11g38930.1	11
631	LOC_Os01g51280.1	1
631	LOC_Os07g10460.1	7
632	LOC_Os01g72350.1	1
632	LOC_Os12g28270.1	12
633	LOC_Os04g55860.1	4
633	LOC_Os05g44390.1	5
634	LOC_Os05g47850.1	5
634	LOC_Os09g19850.1	9
635	LOC_Os01g16100.1	1
635	LOC_Os10g30640.1	10
636	LOC_Os03g57280.1	3
636	LOC_Os10g21360.1	10
637	LOC_Os12g34450.1	12
637	LOC_Os12g34460.1	12
638	LOC_Os03g01150.1	3
638	LOC_Os10g41340.1	10
639	LOC_Os01g63250.1	1
639	LOC_Os03g15850.1	3
640	LOC_Os01g53720.1	1
640	LOC_Os02g13360.1	2
641	LOC_Os04g43150.1	4
641	LOC_Os08g42000.1	8
642	LOC_Os02g53700.1	2
642	LOC_Os03g05200.1	3
643	LOC_Os04g20270.1	4
643	LOC_Os12g07950.1	12
644	LOC_Os03g01230.1	3
644	LOC_Os12g37370.1	12
645	LOC_Os06g35050.1	6
645	LOC_Os06g49520.1	6
646	LOC_Os01g04900.1	1
646	LOC_Os03g57840.1	3
647	LOC_Os01g70960.1	1
647	LOC_Os05g23620.1	5
648	LOC_Os03g47830.1	3
648	LOC_Os03g57560.1	3
649	LOC_Os02g39920.1	2
649	LOC_Os04g42320.1	4

650	LOC_Os06g33250.1	6
650	LOC_Os10g18150.1	10
651	LOC_Os03g62580.1	3
651	LOC_Os03g15650.1	3
652	LOC_Os01g05500.1	1
652	LOC_Os06g33810.1	6
653	LOC_Os03g57420.1	3
653	LOC_Os07g06590.1	7
654	LOC_Os01g50960.1	1
654	LOC_Os02g34840.1	2
655	LOC_Os03g04490.1	3
655	LOC_Os10g33310.1	10
656	LOC_Os01g73890.1	1
656	LOC_Os05g01710.1	5
657	LOC_Os01g56550.1	1
657	LOC_Os05g43450.1	5
658	LOC_Os03g29470.1	3
658	LOC_Os07g42150.1	7
659	LOC_Os02g43660.1	2
659	LOC_Os06g11490.1	6
660	LOC_Os01g07170.1	1
660	LOC_Os12g41350.1	12
661	LOC_Os03g43400.1	3
661	LOC_Os05g09480.1	5
662	LOC_Os11g32110.1	11
662	LOC_Os12g29520.1	12
663	LOC_Os01g70270.1	1
663	LOC_Os04g56850.1	4
664	LOC_Os02g33730.1	2
664	LOC_Os11g06340.1	11
665	LOC_Os01g51410.1	1
665	LOC_Os06g40940.1	6
666	LOC_Os02g29220.1	2
666	LOC_Os04g35220.1	4
667	LOC_Os06g09660.1	6
667	LOC_Os08g40900.1	8
668	LOC_Os02g41800.1	2
668	LOC_Os04g43910.1	4
669	LOC_Os06g47150.1	6
669	LOC_Os10g33940.1	10
670	LOC_Os01g48060.1	1
670	LOC_Os01g54990.1	1
671	LOC_Os07g37610.1	7
671	LOC_Os07g48200.1	7
672	LOC_Os10g25620.1	10
672	LOC_Os10g26270.1	10
673	LOC_Os03g61249.1	3
673	LOC_Os03g61319.1	3
674	LOC_Os01g60020.1	1
674	LOC_Os11g03300.1	11
675	LOC_Os03g60080.1	3
675	LOC_Os07g12340.1	7

676	LOC_Os05g10770.1	5
676	LOC_Os05g23670.1	5
677	LOC_Os02g51100.1	2
677	LOC_Os09g34970.1	9
678	LOC_Os03g52170.1	3
678	LOC_Os03g52180.1	3
679	LOC_Os03g56370.1	3
679	LOC_Os07g14350.1	7
680	LOC_Os09g32030.1	9
680	LOC_Os12g13824.1	12
681	LOC_Os03g43430.1	3
681	LOC_Os11g37130.1	11
682	LOC_Os11g02440.1	11
682	LOC_Os12g02370.1	12
683	LOC_Os05g51790.1	5
683	LOC_Os12g44390.1	12
684	LOC_Os08g23180.1	8
684	LOC_Os09g07350.1	9
685	LOC_Os01g29409.1	1
685	LOC_Os02g39370.1	2
686	LOC_Os05g49050.1	5
686	LOC_Os07g40404.1	7
687	LOC_Os09g28060.1	9
687	LOC_Os09g38330.1	9
688	LOC_Os03g20700.1	3
688	LOC_Os07g46310.1	7
689	LOC_Os04g57890.1	4
689	LOC_Os05g44360.1	5
690	LOC_Os01g12560.1	1
690	LOC_Os01g12570.1	1
691	LOC_Os01g16146.1	1
691	LOC_Os03g32040.1	3
692	LOC_Os04g58970.1	4
692	LOC_Os07g19470.1	7
693	LOC_Os01g61700.1	1
693	LOC_Os08g33540.1	8
694	LOC_Os02g58450.1	2
694	LOC_Os04g01780.1	4
695	LOC_Os02g06480.1	2
695	LOC_Os09g20430.1	9
696	LOC_Os01g40620.1	1
696	LOC_Os01g68390.1	1
697	LOC_Os11g04500.1	11
697	LOC_Os12g04270.1	12
698	LOC_Os06g15910.1	6
698	LOC_Os06g42030.1	6
699	LOC_Os03g51620.1	3
699	LOC_Os12g39320.1	12
700	LOC_Os11g03430.1	11
700	LOC_Os12g03130.1	12
701	LOC_Os02g52250.1	2
701	LOC_Os06g11420.1	6

702	LOC_Os01g71960.1	1
702	LOC_Os03g01100.1	3
703	LOC_Os01g58020.1	1
703	LOC_Os05g35330.1	5
704	LOC_Os08g01660.1	8
704	LOC_Os12g18880.1	12
705	LOC_Os02g51660.1	2
705	LOC_Os03g09180.1	3
706	LOC_Os08g17680.1	8
706	LOC_Os08g34190.1	8
707	LOC_Os03g03460.1	3
707	LOC_Os10g38900.1	10
708	LOC_Os03g49430.1	3
708	LOC_Os07g38420.1	7
709	LOC_Os02g09180.1	2
709	LOC_Os06g43550.1	6
710	LOC_Os02g39840.1	2
710	LOC_Os04g42140.1	4
711	LOC_Os02g42040.1	2
711	LOC_Os03g22570.1	3
712	LOC_Os10g40130.1	10
712	LOC_Os12g42870.1	12
713	LOC_Os06g06870.1	6
713	LOC_Os07g25890.1	7
714	LOC_Os08g09800.1	8
714	LOC_Os08g09810.1	8
715	LOC_Os02g15520.1	2
715	LOC_Os12g10260.1	12
716	LOC_Os03g14600.1	3
716	LOC_Os06g10740.1	6
717	LOC_Os10g02910.1	10
717	LOC_Os11g40360.1	11
718	LOC_Os01g01560.1	1
718	LOC_Os11g05040.1	11
719	LOC_Os02g39020.1	2
719	LOC_Os11g42780.1	11
720	LOC_Os04g48780.1	4
720	LOC_Os07g17780.1	7
721	LOC_Os09g21420.1	9
721	LOC_Os09g21430.1	9
722	LOC_Os02g15510.1	2
722	LOC_Os02g15390.1	2
723	LOC_Os04g01110.1	4
723	LOC_Os07g23210.1	7
724	LOC_Os01g65680.1	1
724	LOC_Os01g65690.1	1
725	LOC_Os09g08450.1	9
725	LOC_Os11g12500.1	11
726	LOC_Os04g30860.1	4
726	LOC_Os08g33280.1	8
727	LOC_Os02g42570.1	2
727	LOC_Os04g44650.1	4

728	LOC_Os10g34520.1	10
728	LOC_Os11g19220.1	11
729	LOC_Os02g05310.1	2
729	LOC_Os02g05410.1	2
730	LOC_Os06g12830.1	6
730	LOC_Os06g15560.1	6
731	LOC_Os07g46230.1	7
731	LOC_Os12g36750.1	12
732	LOC_Os03g18110.1	3
732	LOC_Os06g12860.1	6
733	LOC_Os03g18140.1	3
733	LOC_Os05g28830.1	5
734	LOC_Os06g12800.1	6
734	LOC_Os06g12820.1	6
735	LOC_Os07g42570.1	7
735	LOC_Os11g07690.1	11
736	LOC_Os01g64750.1	1
736	LOC_Os04g04254.1	4
737	LOC_Os08g28240.1	8
737	LOC_Os09g15240.1	9
738	LOC_Os03g19390.1	3
738	LOC_Os08g45160.1	8
739	LOC_Os02g05620.1	2
739	LOC_Os06g48330.1	6
740	LOC_Os06g51010.1	6
740	LOC_Os10g36670.1	10
741	LOC_Os03g08800.1	3
741	LOC_Os10g23204.1	10
742	LOC_Os03g15040.1	3
742	LOC_Os04g04400.1	4
743	LOC_Os03g44810.1	3
743	LOC_Os09g34990.1	9
744	LOC_Os03g29250.1	3
744	LOC_Os07g42330.1	7
745	LOC_Os04g21950.1	4
745	LOC_Os04g51560.1	4
746	LOC_Os02g47060.1	2
746	LOC_Os04g50920.1	4
747	LOC_Os01g56780.1	1
747	LOC_Os12g35030.1	12
748	LOC_Os12g06620.1	12
748	LOC_Os12g43100.1	12
749	LOC_Os01g03420.1	1
749	LOC_Os01g55640.1	1
750	LOC_Os01g54580.1	1
750	LOC_Os06g05980.1	6
751	LOC_Os02g46320.1	2
751	LOC_Os07g07010.1	7
752	LOC_Os06g07914.1	6
752	LOC_Os06g08014.1	6
753	LOC_Os06g07932.1	6
753	LOC_Os06g08032.1	6

754	LOC_Os06g08041.1	6
754	LOC_Os06g07941.1	6
755	LOC_Os06g14390.1	6
755	LOC_Os06g14400.1	6
756	LOC_Os01g35230.1	1
756	LOC_Os08g30150.1	8
757	LOC_Os02g47320.1	2
757	LOC_Os04g51270.1	4
758	LOC_Os01g45910.1	1
758	LOC_Os02g11740.1	2
759	LOC_Os01g61240.1	1
759	LOC_Os03g18454.1	3
760	LOC_Os01g04210.1	1
760	LOC_Os01g04220.1	1
761	LOC_Os02g22160.1	2
761	LOC_Os03g52120.1	3
762	LOC_Os01g65490.1	1
762	LOC_Os10g08280.1	10
763	LOC_Os03g44580.1	3
763	LOC_Os07g06490.1	7
764	LOC_Os02g54000.1	2
764	LOC_Os08g08200.1	8
765	LOC_Os01g72430.1	1
765	LOC_Os01g72460.1	1
766	LOC_Os03g18600.1	3
766	LOC_Os05g39580.1	5
767	LOC_Os03g27350.1	3
767	LOC_Os04g58560.1	4
768	LOC_Os02g54254.1	2
768	LOC_Os07g40620.1	7
769	LOC_Os01g53910.1	1
769	LOC_Os06g46372.1	6
770	LOC_Os06g45860.1	6
770	LOC_Os09g38777.1	9
771	LOC_Os11g38250.1	11
771	LOC_Os11g38190.1	11
772	LOC_Os03g56781.1	3
772	LOC_Os11g24940.1	11
773	LOC_Os02g38770.1	2
773	LOC_Os03g56770.1	3
774	LOC_Os04g08110.1	4
774	LOC_Os04g08240.1	4
775	LOC_Os12g23460.1	12
775	LOC_Os12g23670.1	12
776	LOC_Os02g38508.1	2
776	LOC_Os02g38598.1	2
777	LOC_Os03g56720.1	3
777	LOC_Os03g56760.1	3
778	LOC_Os04g40920.1	4
778	LOC_Os08g27540.1	8
779	LOC_Os05g49510.1	5
779	LOC_Os12g23450.1	12

780	LOC_Os06g13490.1	6
780	LOC_Os06g13520.1	6
781	LOC_Os04g57070.1	4
781	LOC_Os06g21760.1	6
782	LOC_Os02g44370.1	2
782	LOC_Os10g40390.1	10
783	LOC_Os01g45860.1	1
783	LOC_Os03g49990.1	3
784	LOC_Os11g04590.1	11
784	LOC_Os12g04380.1	12
785	LOC_Os04g46860.1	4
785	LOC_Os06g01620.1	6
786	LOC_Os11g04570.1	11
786	LOC_Os12g04370.1	12
787	LOC_Os01g65900.1	1
787	LOC_Os07g36170.1	7
788	LOC_Os11g04400.1	11
788	LOC_Os12g04200.1	12
789	LOC_Os04g50060.1	4
789	LOC_Os07g38030.1	7
790	LOC_Os11g47900.1	11
790	LOC_Os12g38490.1	12
791	LOC_Os11g47890.1	11
791	LOC_Os11g47870.1	11
792	LOC_Os01g62460.1	1
792	LOC_Os11g47920.1	11
793	LOC_Os03g14520.1	3
793	LOC_Os10g08879.1	10
794	LOC_Os05g08370.1	5
794	LOC_Os09g25490.1	9
795	LOC_Os01g73850.1	1
795	LOC_Os05g05870.1	5
796	LOC_Os01g43460.1	1
796	LOC_Os05g50770.1	5
797	LOC_Os03g05390.1	3
797	LOC_Os10g39980.1	10
798	LOC_Os05g05980.1	5
798	LOC_Os07g01450.1	7
799	LOC_Os06g10520.1	6
799	LOC_Os09g36270.1	9
800	LOC_Os06g22670.1	6
800	LOC_Os12g41210.1	12
801	LOC_Os11g41130.1	11
801	LOC_Os12g31640.1	12
802	LOC_Os01g55900.1	1
802	LOC_Os10g40650.1	10
803	LOC_Os01g73020.1	1
803	LOC_Os10g33910.1	10
804	LOC_Os04g56760.1	4
804	LOC_Os05g30410.1	5
805	LOC_Os03g63700.1	3
805	LOC_Os11g04420.1	11

806	LOC_Os10g35280.1	10
806	LOC_Os12g06610.1	12
807	LOC_Os04g56440.1	4
807	LOC_Os08g39970.1	8
808	LOC_Os02g52450.1	2
808	LOC_Os02g52430.1	2
809	LOC_Os11g03580.1	11
809	LOC_Os12g03350.1	12
810	LOC_Os03g47042.1	3
810	LOC_Os03g47022.1	3
811	LOC_Os03g38010.1	3
811	LOC_Os08g06600.1	8
812	LOC_Os02g14290.1	2
812	LOC_Os04g51080.1	4
813	LOC_Os03g06090.1	3
813	LOC_Os06g02580.1	6
814	LOC_Os01g63940.1	1
814	LOC_Os01g62820.1	1
815	LOC_Os03g62870.1	3
815	LOC_Os06g48230.1	6
816	LOC_Os05g06490.1	5
816	LOC_Os07g29420.1	7
817	LOC_Os04g08320.1	4
817	LOC_Os06g41590.1	6
818	LOC_Os03g22620.1	3
818	LOC_Os03g24650.1	3
819	LOC_Os04g37710.1	4
819	LOC_Os07g48770.1	7
820	LOC_Os07g43020.1	7
820	LOC_Os10g30300.1	10
821	LOC_Os03g63500.1	3
821	LOC_Os08g16480.1	8
822	LOC_Os02g48350.1	2
822	LOC_Os06g22080.1	6
823	LOC_Os01g10290.1	1
823	LOC_Os10g41110.1	10
824	LOC_Os04g41990.1	4
824	LOC_Os12g32210.1	12
825	LOC_Os07g01880.1	7
825	LOC_Os12g38970.1	12
826	LOC_Os05g06840.1	5
826	LOC_Os08g36330.1	8
827	LOC_Os08g25070.1	8
827	LOC_Os11g03750.1	11
828	LOC_Os03g43820.1	3
828	LOC_Os10g10630.1	10
829	LOC_Os03g01240.1	3
829	LOC_Os10g40780.1	10
830	LOC_Os02g15870.1	2
830	LOC_Os12g32230.1	12
831	LOC_Os03g03650.1	3
831	LOC_Os12g13950.1	12

832	LOC_Os02g06320.1	2
832	LOC_Os09g36140.1	9
833	LOC_Os05g13330.1	5
833	LOC_Os10g38030.1	10
834	LOC_Os03g62110.1	3
834	LOC_Os04g35060.1	4
835	LOC_Os01g33020.1	1
835	LOC_Os07g27150.1	7
836	LOC_Os05g03820.1	5
836	LOC_Os07g27790.1	7
837	LOC_Os03g14380.1	3
837	LOC_Os10g07994.1	10
838	LOC_Os02g36550.1	2
838	LOC_Os08g07880.1	8
839	LOC_Os03g30460.1	3
839	LOC_Os10g34680.1	10
840	LOC_Os01g73570.1	1
840	LOC_Os02g55980.1	2
841	LOC_Os09g36020.1	9
841	LOC_Os11g44790.1	11
842	LOC_Os11g04520.1	11
842	LOC_Os12g04290.1	12
843	LOC_Os06g11020.1	6
843	LOC_Os07g19030.1	7
844	LOC_Os07g22700.1	7
844	LOC_Os07g45300.1	7
845	LOC_Os01g11590.1	1
845	LOC_Os03g24830.1	3
846	LOC_Os08g06050.1	8
846	LOC_Os12g42760.1	12
847	LOC_Os08g25140.1	8
847	LOC_Os09g07320.1	9
848	LOC_Os08g38040.1	8
848	LOC_Os12g20420.1	12
849	LOC_Os02g51330.1	2
849	LOC_Os07g48720.1	7
850	LOC_Os03g25260.1	3
850	LOC_Os10g37640.1	10
851	LOC_Os03g59680.1	3
851	LOC_Os12g37430.1	12
852	LOC_Os04g41150.1	4
852	LOC_Os09g11520.1	9
853	LOC_Os04g44740.1	4
853	LOC_Os07g37990.1	7
854	LOC_Os02g53150.1	2
854	LOC_Os08g36450.1	8
855	LOC_Os03g63520.1	3
855	LOC_Os05g09550.1	5
856	LOC_Os03g09900.1	3
856	LOC_Os08g41280.1	8
857	LOC_Os03g09850.1	3
857	LOC_Os09g32470.1	9

858	LOC_Os02g12760.1	2
858	LOC_Os11g42940.1	11
859	LOC_Os04g35680.1	4
859	LOC_Os05g36360.1	5
860	LOC_Os01g04030.1	1
860	LOC_Os09g38370.1	9
861	LOC_Os06g02850.1	6
861	LOC_Os06g02960.1	6
862	LOC_Os04g05360.1	4
862	LOC_Os04g06500.1	4
863	LOC_Os01g05020.1	1
863	LOC_Os01g05040.1	1
864	LOC_Os03g58740.1	3
864	LOC_Os05g43050.1	5
865	LOC_Os08g06730.1	8
865	LOC_Os08g06740.1	8
866	LOC_Os01g65720.1	1
866	LOC_Os12g21789.1	12
867	LOC_Os02g40000.1	2
867	LOC_Os04g42500.1	4
868	LOC_Os02g10000.1	2
868	LOC_Os02g44260.1	2
869	LOC_Os01g46020.1	1
869	LOC_Os05g28010.1	5
870	LOC_Os04g33440.1	4
870	LOC_Os04g33420.1	4
871	LOC_Os02g48670.1	2
871	LOC_Os09g31190.1	9
872	LOC_Os01g49150.1	1
872	LOC_Os05g48030.1	5
873	LOC_Os04g52270.1	4
873	LOC_Os04g55200.1	4
874	LOC_Os02g26700.1	2
874	LOC_Os04g37580.1	4
875	LOC_Os01g31580.1	1
875	LOC_Os09g35620.1	9
876	LOC_Os02g05900.1	2
876	LOC_Os06g49060.1	6
877	LOC_Os03g55890.1	3
877	LOC_Os07g03140.1	7
878	LOC_Os01g23590.1	1
878	LOC_Os05g06470.1	5
879	LOC_Os04g33690.1	4
879	LOC_Os05g37120.1	5
880	LOC_Os03g18910.1	3
880	LOC_Os07g49080.1	7
881	LOC_Os01g54180.1	1
881	LOC_Os05g27120.1	5
882	LOC_Os01g51980.1	1
882	LOC_Os01g52010.1	1
883	LOC_Os01g07500.1	1
883	LOC_Os05g07720.1	5

884	LOC_Os07g16950.1	7
884	LOC_Os07g17040.1	7
885	LOC_Os02g26850.1	2
885	LOC_Os06g06410.1	6
886	LOC_Os03g19920.1	3
886	LOC_Os04g49870.1	4
887	LOC_Os03g53610.1	3
887	LOC_Os06g23350.1	6
888	LOC_Os03g06360.1	3
888	LOC_Os03g53620.1	3
889	LOC_Os01g50730.1	1
889	LOC_Os07g48890.1	7
890	LOC_Os06g44800.1	6
890	LOC_Os11g39660.1	11
891	LOC_Os08g20290.1	8
891	LOC_Os12g26020.1	12
892	LOC_Os02g32650.1	2
892	LOC_Os04g33450.1	4
893	LOC_Os04g08764.1	4
893	LOC_Os10g27090.1	10
894	LOC_Os03g33590.1	3
894	LOC_Os07g39900.1	7
895	LOC_Os04g57790.1	4
895	LOC_Os05g09650.1	5
896	LOC_Os05g34290.1	5
896	LOC_Os06g01260.1	6
897	LOC_Os03g10930.1	3
897	LOC_Os04g24520.1	4
898	LOC_Os01g26896.1	1
898	LOC_Os01g27100.1	1
899	LOC_Os01g66580.1	1
899	LOC_Os04g32350.1	4
900	LOC_Os04g44730.1	4
900	LOC_Os10g35480.1	10
901	LOC_Os03g60920.1	3
901	LOC_Os12g41830.1	12
902	LOC_Os02g01110.1	2
902	LOC_Os02g55120.1	2
903	LOC_Os02g43750.1	2
903	LOC_Os03g32590.1	3
904	LOC_Os03g02170.1	3
904	LOC_Os10g36830.1	10
905	LOC_Os01g63310.1	1
905	LOC_Os05g37800.1	5
906	LOC_Os02g50360.1	2
906	LOC_Os08g01180.1	8
907	LOC_Os03g50870.1	3
907	LOC_Os04g50860.1	4
908	LOC_Os03g55850.1	3
908	LOC_Os05g49170.1	5
909	LOC_Os05g49470.1	5
909	LOC_Os08g06360.1	8

910	LOC_Os03g02380.1	3
910	LOC_Os10g37520.1	10
911	LOC_Os02g11000.1	2
911	LOC_Os06g39900.1	6
912	LOC_Os07g07020.1	7
912	LOC_Os10g41450.1	10
913	LOC_Os01g54670.1	1
913	LOC_Os05g13440.1	5
914	LOC_Os09g04280.1	9
914	LOC_Os12g16130.1	12
915	LOC_Os01g18650.1	1
915	LOC_Os05g12780.1	5
916	LOC_Os01g14190.1	1
916	LOC_Os04g42810.1	4
917	LOC_Os04g45850.1	4
917	LOC_Os08g30270.1	8
918	LOC_Os05g28270.1	5
918	LOC_Os12g16490.1	12
919	LOC_Os04g15910.1	4
919	LOC_Os06g02090.1	6
920	LOC_Os04g01130.1	4
920	LOC_Os11g34300.1	11
921	LOC_Os03g50040.1	3
921	LOC_Os05g39650.1	5
922	LOC_Os03g62670.1	3
922	LOC_Os06g06040.1	6
923	LOC_Os04g47680.1	4
923	LOC_Os09g15480.1	9
924	LOC_Os01g01307.1	1
924	LOC_Os05g01262.1	5
925	LOC_Os07g41439.1	7
925	LOC_Os07g41448.1	7
926	LOC_Os05g05940.1	5
926	LOC_Os07g47510.1	7
927	LOC_Os07g36080.1	7
927	LOC_Os07g01480.1	7
928	LOC_Os03g14720.1	3
928	LOC_Os10g10990.1	10
929	LOC_Os01g22870.1	1
929	LOC_Os05g06700.1	5
930	LOC_Os08g34120.1	8
930	LOC_Os09g24970.1	9
931	LOC_Os11g03760.1	11
931	LOC_Os12g03500.1	12
932	LOC_Os07g23540.1	7
932	LOC_Os12g06230.1	12
933	LOC_Os03g31560.1	3
933	LOC_Os07g02230.1	7
934	LOC_Os02g19280.1	2
934	LOC_Os12g32690.1	12
935	LOC_Os02g22280.1	2
935	LOC_Os12g23030.1	12

936	LOC_Os01g44200.1	1
936	LOC_Os02g24780.1	2
937	LOC_Os04g03750.1	4
937	LOC_Os08g41070.1	8
938	LOC_Os06g29550.1	6
938	LOC_Os08g20520.1	8
939	LOC_Os04g24140.1	4
939	LOC_Os07g08030.1	7
940	LOC_Os08g36750.1	8
940	LOC_Os09g28220.1	9
941	LOC_Os01g62040.1	1
941	LOC_Os07g08170.1	7
942	LOC_Os02g04800.1	2
942	LOC_Os06g48960.1	6
943	LOC_Os01g04110.1	1
943	LOC_Os08g37480.1	8
944	LOC_Os01g37832.1	1
944	LOC_Os03g58130.1	3
945	LOC_Os01g07780.1	1
945	LOC_Os05g07890.1	5
946	LOC_Os03g03980.1	3
946	LOC_Os04g47370.1	4
947	LOC_Os02g33230.1	2
947	LOC_Os04g33770.1	4
948	LOC_Os05g10980.1	5
948	LOC_Os07g38600.1	7
949	LOC_Os03g56610.1	3
949	LOC_Os04g42340.1	4
950	LOC_Os06g07480.1	6
950	LOC_Os11g28350.1	11
951	LOC_Os10g25040.1	10
951	LOC_Os10g25030.1	10
952	LOC_Os03g22370.1	3
952	LOC_Os07g47640.1	7
953	LOC_Os02g27110.1	2
953	LOC_Os08g02350.1	8
954	LOC_Os08g05200.1	8
954	LOC_Os12g13490.1	12
955	LOC_Os03g29830.1	3
955	LOC_Os07g41660.1	7
956	LOC_Os01g64990.1	1
956	LOC_Os02g46350.1	2
957	LOC_Os03g32490.1	3
957	LOC_Os05g46250.1	5
958	LOC_Os12g01570.1	12
958	LOC_Os12g44180.1	12
959	LOC_Os04g31924.1	4
959	LOC_Os12g29950.1	12
960	LOC_Os05g07930.1	5
960	LOC_Os10g38770.1	10
961	LOC_Os04g33940.1	4
961	LOC_Os09g36982.1	9

962	LOC_Os05g04120.1	5
962	LOC_Os06g36450.1	6
963	LOC_Os03g50070.1	3
963	LOC_Os04g01510.1	4
964	LOC_Os04g53310.1	4
964	LOC_Os08g09230.1	8
965	LOC_Os04g33280.1	4
965	LOC_Os09g26580.1	9
966	LOC_Os01g56680.1	1
966	LOC_Os05g43310.1	5
967	LOC_Os01g24480.1	1
967	LOC_Os11g40590.1	11
968	LOC_Os03g43240.1	3
968	LOC_Os08g20885.1	8
969	LOC_Os03g37580.1	3
969	LOC_Os03g08770.1	3
970	LOC_Os01g55050.1	1
970	LOC_Os05g07730.1	5
971	LOC_Os01g48220.1	1
971	LOC_Os05g48760.1	5
972	LOC_Os04g22860.1	4
972	LOC_Os05g18774.1	5
973	LOC_Os03g40650.1	3
973	LOC_Os12g38620.1	12
974	LOC_Os11g03590.1	11
974	LOC_Os12g03360.1	12
975	LOC_Os07g41920.1	7
975	LOC_Os09g20150.1	9
976	LOC_Os07g41910.1	7
976	LOC_Os07g42040.1	7
977	LOC_Os04g10260.1	4
977	LOC_Os09g34880.1	9
978	LOC_Os01g04620.1	1
978	LOC_Os05g44130.1	5
979	LOC_Os07g17520.1	7
979	LOC_Os07g18670.1	7
980	LOC_Os02g52230.1	2
980	LOC_Os03g15880.1	3
981	LOC_Os01g36900.1	1
981	LOC_Os11g28440.1	11
982	LOC_Os04g55190.1	4
982	LOC_Os06g39480.1	6
983	LOC_Os01g14170.1	1
983	LOC_Os01g14180.1	1
984	LOC_Os01g13810.1	1
984	LOC_Os06g20310.1	6
985	LOC_Os12g34440.1	12
985	LOC_Os12g39710.1	12
986	LOC_Os01g09340.1	1
986	LOC_Os01g09420.1	1
987	LOC_Os03g40040.1	3
987	LOC_Os12g07440.1	12

988	LOC_Os02g39550.1	2
988	LOC_Os04g41950.1	4
989	LOC_Os04g05070.1	4
989	LOC_Os05g02450.1	5
990	LOC_Os03g44520.1	3
990	LOC_Os07g06460.1	7
991	LOC_Os11g04950.1	11
991	LOC_Os12g04940.1	12
992	LOC_Os03g19090.1	3
992	LOC_Os11g44920.1	11
993	LOC_Os01g72190.1	1
993	LOC_Os05g31690.1	5
994	LOC_Os05g33730.1	5
994	LOC_Os07g44850.1	7
995	LOC_Os07g41590.1	7
995	LOC_Os07g44890.1	7
996	LOC_Os08g37040.1	8
996	LOC_Os09g28640.1	9
997	LOC_Os01g06220.1	1
997	LOC_Os03g57640.1	3
998	LOC_Os07g34370.1	7
998	LOC_Os09g28650.1	9
999	LOC_Os01g36090.1	1
999	LOC_Os07g31270.1	7
1000	LOC_Os01g02180.1	1
1000	LOC_Os05g08980.1	5
1001	LOC_Os02g01710.1	2
1001	LOC_Os12g18760.1	12
1002	LOC_Os01g16970.1	1
1002	LOC_Os06g43710.1	6
1003	LOC_Os01g42830.1	1
1003	LOC_Os10g33630.1	10
1004	LOC_Os05g36000.1	5
1004	LOC_Os08g12840.1	8
1005	LOC_Os02g17700.1	2
1005	LOC_Os06g11140.1	6
1006	LOC_Os01g08850.1	1
1006	LOC_Os01g25240.1	1
1007	LOC_Os01g46060.1	1
1007	LOC_Os12g43830.1	12
1008	LOC_Os06g25200.1	6
1008	LOC_Os07g15840.1	7
1009	LOC_Os07g22970.1	7
1009	LOC_Os12g28320.1	12
1010	LOC_Os10g16410.1	10
1010	LOC_Os12g13260.1	12
1011	LOC_Os02g24690.1	2
1011	LOC_Os07g10010.1	7
1012	LOC_Os04g28800.1	4
1012	LOC_Os05g40100.1	5
1013	LOC_Os06g19100.1	6
1013	LOC_Os10g26530.1	10

1014	LOC_Os03g54010.1	3
1014	LOC_Os07g04880.1	7
1015	LOC_Os02g39250.1	2
1015	LOC_Os03g07460.1	3
1016	LOC_Os06g19820.1	6
1016	LOC_Os11g02650.1	11
1017	LOC_Os01g10240.1	1
1017	LOC_Os07g37454.1	7
1018	LOC_Os03g20970.1	3
1018	LOC_Os03g20958.1	3
1019	LOC_Os11g09680.1	11
1019	LOC_Os11g10250.1	11
1020	LOC_Os04g02550.1	4
1020	LOC_Os04g22910.1	4
1021	LOC_Os04g22730.1	4
1021	LOC_Os11g25230.1	11
1022	LOC_Os02g45580.1	2
1022	LOC_Os10g24090.1	10
1023	LOC_Os06g20470.1	6
1023	LOC_Os06g20530.1	6
1024	LOC_Os11g22480.1	11
1024	LOC_Os12g33630.1	12
1025	LOC_Os04g26260.1	4
1025	LOC_Os08g19680.1	8
1026	LOC_Os09g28240.1	9
1026	LOC_Os09g31442.1	9
1027	LOC_Os04g25390.1	4
1027	LOC_Os04g25960.1	4
1028	LOC_Os01g49140.1	1
1028	LOC_Os04g21010.1	4
1029	LOC_Os10g12812.1	10
1029	LOC_Os11g33462.1	11
1030	LOC_Os03g33920.1	3
1030	LOC_Os09g38590.1	9
1031	LOC_Os06g36630.1	6
1031	LOC_Os08g12100.1	8
1032	LOC_Os10g11550.1	10
1032	LOC_Os12g27560.1	12
1033	LOC_Os05g16869.1	5
1033	LOC_Os05g16889.1	5
1034	LOC_Os01g57294.1	1
1034	LOC_Os01g57324.1	1
1035	LOC_Os02g19600.1	2
1035	LOC_Os02g24980.1	2
1036	LOC_Os10g22920.1	10
1036	LOC_Os11g35790.1	11
1037	LOC_Os04g40750.1	4
1037	LOC_Os06g42210.1	6
1038	LOC_Os03g44330.1	3
1038	LOC_Os08g03460.1	8
1039	LOC_Os04g33580.1	4
1039	LOC_Os12g44110.1	12

1040	LOC_Os02g14330.1	2
1040	LOC_Os02g14350.1	2
1041	LOC_Os12g33859.1	12
1041	LOC_Os12g33879.1	12
1042	LOC_Os05g17210.1	5
1042	LOC_Os05g17230.1	5
1043	LOC_Os06g17629.1	6
1043	LOC_Os06g17659.1	6
1044	LOC_Os03g36070.1	3
1044	LOC_Os08g08080.1	8
1045	LOC_Os01g16730.1	1
1045	LOC_Os05g03080.1	5
1046	LOC_Os11g25080.1	11
1046	LOC_Os12g08250.1	12
1047	LOC_Os05g44690.1	5
1047	LOC_Os05g44710.1	5
1048	LOC_Os10g42202.1	10
1048	LOC_Os11g17280.1	11
1049	LOC_Os04g02040.1	4
1049	LOC_Os04g02480.1	4
1050	LOC_Os05g03500.1	5
1050	LOC_Os10g07340.1	10
1051	LOC_Os03g19260.1	3
1051	LOC_Os05g47960.1	5
1052	LOC_Os08g07200.1	8
1052	LOC_Os10g07970.1	10
1053	LOC_Os01g08440.1	1
1053	LOC_Os02g10880.1	2
1054	LOC_Os04g12970.1	4
1054	LOC_Os04g12980.1	4
1055	LOC_Os01g41950.1	1
1055	LOC_Os01g41970.1	1
1056	LOC_Os03g12090.1	3
1056	LOC_Os03g45970.1	3
1057	LOC_Os06g29880.1	6
1057	LOC_Os10g16500.1	10
1058	LOC_Os10g05170.1	10
1058	LOC_Os11g47490.1	11
1059	LOC_Os11g12020.1	11
1059	LOC_Os11g12350.1	11
1060	LOC_Os05g27050.1	5
1060	LOC_Os06g49230.1	6
1061	LOC_Os06g28540.1	6
1061	LOC_Os07g24410.1	7
1062	LOC_Os08g27410.1	8
1062	LOC_Os08g40320.1	8
1063	LOC_Os07g37050.1	7
1063	LOC_Os09g02320.1	9
1064	LOC_Os08g26170.1	8
1064	LOC_Os08g30300.1	8
1065	LOC_Os12g34980.1	12
1065	LOC_Os12g34920.1	12

1066	LOC_Os12g07620.1	12
1066	LOC_Os12g09230.1	12
1067	LOC_Os11g09940.1	11
1067	LOC_Os12g09190.1	12
1068	LOC_Os01g65700.1	1
1068	LOC_Os01g65710.1	1
1069	LOC_Os01g37180.1	1
1069	LOC_Os06g14580.1	6
1070	LOC_Os02g51650.1	2
1070	LOC_Os11g31440.1	11
1071	LOC_Os01g27850.1	1
1071	LOC_Os11g25740.1	11
1072	LOC_Os08g28200.1	8
1072	LOC_Os09g36690.1	9
1073	LOC_Os06g02270.1	6
1073	LOC_Os06g02320.1	6
1074	LOC_Os05g30780.1	5
1074	LOC_Os11g30030.1	11
1075	LOC_Os10g37700.1	10
1075	LOC_Os12g05630.1	12
1076	LOC_Os09g26460.1	9
1076	LOC_Os11g38710.1	11
1077	LOC_Os01g34520.1	1
1077	LOC_Os02g17560.1	2
1078	LOC_Os07g35160.1	7
1078	LOC_Os07g35190.1	7
1079	LOC_Os05g04900.1	5
1079	LOC_Os07g46050.1	7
1080	LOC_Os07g46030.1	7
1080	LOC_Os07g46080.1	7
1081	LOC_Os07g45460.1	7
1081	LOC_Os07g45780.1	7
1082	LOC_Os03g42860.1	3
1082	LOC_Os06g29290.1	6
1083	LOC_Os11g06560.1	11
1083	LOC_Os11g06610.1	11
1084	LOC_Os04g49370.1	4
1084	LOC_Os09g32330.1	9
1085	LOC_Os05g14280.1	5
1085	LOC_Os12g34030.1	12
1086	LOC_Os07g02170.1	7
1086	LOC_Os11g11510.1	11
1087	LOC_Os01g02500.1	1
1087	LOC_Os04g10569.1	4
1088	LOC_Os01g34900.1	1
1088	LOC_Os03g03620.1	3
1089	LOC_Os11g34220.1	11
1089	LOC_Os11g34230.1	11
1090	LOC_Os05g01860.1	5
1090	LOC_Os05g01890.1	5
1091	LOC_Os04g18940.1	4
1091	LOC_Os10g24120.1	10

1092	LOC_Os09g03070.1	9
1092	LOC_Os10g16510.1	10
1093	LOC_Os04g05090.1	4
1093	LOC_Os04g06050.1	4
1094	LOC_Os02g14610.1	2
1094	LOC_Os06g37870.1	6
1095	LOC_Os05g37610.1	5
1095	LOC_Os05g41960.1	5
1096	LOC_Os06g38080.1	6
1096	LOC_Os11g46000.1	11
1097	LOC_Os09g13290.1	9
1097	LOC_Os10g16740.1	10
1098	LOC_Os03g40240.1	3
1098	LOC_Os06g10090.1	6
1099	LOC_Os04g19160.1	4
1099	LOC_Os05g09510.1	5
1100	LOC_Os01g24160.1	1
1100	LOC_Os03g44990.1	3
1101	LOC_Os03g64269.1	3
1101	LOC_Os11g26956.1	11
1102	LOC_Os02g38990.1	2
1102	LOC_Os03g13579.1	3
1103	LOC_Os03g16320.1	3
1103	LOC_Os08g36660.1	8
1104	LOC_Os03g30770.1	3
1104	LOC_Os09g18400.1	9
1105	LOC_Os08g16650.1	8
1105	LOC_Os08g16690.1	8
1106	LOC_Os08g16740.1	8
1106	LOC_Os08g35939.1	8
1107	LOC_Os02g47490.1	2
1107	LOC_Os04g43450.1	4
1108	LOC_Os07g13728.1	7
1108	LOC_Os08g33980.1	8
1109	LOC_Os07g06157.1	7
1109	LOC_Os07g06335.1	7
1110	LOC_Os03g59090.1	3
1110	LOC_Os03g59152.1	3
1111	LOC_Os06g36990.1	6
1111	LOC_Os10g27220.1	10
1112	LOC_Os01g01020.1	1
1112	LOC_Os02g19430.1	2
1113	LOC_Os01g04430.1	1
1113	LOC_Os01g04560.1	1
1114	LOC_Os01g04470.1	1
1114	LOC_Os01g04530.1	1
1115	LOC_Os02g17770.1	2
1115	LOC_Os10g26100.1	10
1116	LOC_Os03g11170.1	3
1116	LOC_Os09g08100.1	9
1117	LOC_Os03g24730.1	3
1117	LOC_Os03g24820.1	3

1118	LOC_Os01g51460.1	1
1118	LOC_Os01g51510.1	1
1119	LOC_Os02g40560.1	2
1119	LOC_Os02g40610.1	2
1120	LOC_Os07g28110.1	7
1120	LOC_Os07g28140.1	7
1121	LOC_Os04g24830.1	4
1121	LOC_Os04g25830.1	4
1122	LOC_Os08g22200.1	8
1122	LOC_Os09g03090.1	9
1123	LOC_Os01g05580.1	1
1123	LOC_Os10g33850.1	10
1124	LOC_Os11g16710.1	11
1124	LOC_Os11g22800.1	11
1125	LOC_Os10g05329.1	10
1125	LOC_Os10g05309.1	10
1126	LOC_Os06g26050.1	6
1126	LOC_Os11g23000.1	11
1127	LOC_Os02g44280.1	2
1127	LOC_Os06g46910.1	6
1128	LOC_Os03g39190.1	3
1128	LOC_Os03g56170.1	3
1129	LOC_Os06g16600.1	6
1129	LOC_Os11g03360.1	11
1130	LOC_Os03g62050.1	3
1130	LOC_Os04g41180.1	4
1131	LOC_Os06g04260.1	6
1131	LOC_Os08g38690.1	8
1132	LOC_Os09g27220.1	9
1132	LOC_Os12g40930.1	12
1133	LOC_Os01g46370.1	1
1133	LOC_Os11g27980.1	11
1134	LOC_Os11g24180.1	11
1134	LOC_Os11g24240.1	11
1135	LOC_Os04g22960.1	4
1135	LOC_Os08g16200.1	8
1136	LOC_Os05g48880.1	5
1136	LOC_Os08g15950.1	8
1137	LOC_Os04g17360.1	4
1137	LOC_Os09g06590.1	9
1138	LOC_Os01g41110.1	1
1138	LOC_Os08g34560.1	8
1139	LOC_Os01g03464.1	1
1139	LOC_Os01g03429.1	1
1140	LOC_Os05g06060.1	5
1140	LOC_Os05g06100.1	5
1141	LOC_Os02g21220.1	2
1141	LOC_Os02g21310.1	2
1142	LOC_Os06g50620.1	6
1142	LOC_Os06g50770.1	6
1143	LOC_Os11g24300.1	11
1143	LOC_Os12g16230.1	12

1144	LOC_Os01g26270.1	1
1144	LOC_Os10g17890.1	10
1145	LOC_Os01g49600.1	1
1145	LOC_Os01g49560.1	1
1146	LOC_Os06g29830.1	6
1146	LOC_Os10g38170.1	10
1147	LOC_Os06g13410.1	6
1147	LOC_Os06g13420.1	6
1148	LOC_Os08g09830.1	8
1148	LOC_Os08g13020.1	8
1149	LOC_Os02g50670.1	2
1149	LOC_Os10g39489.1	10
1150	LOC_Os06g20380.1	6
1150	LOC_Os12g43564.1	12
1151	LOC_Os06g36430.1	6
1151	LOC_Os07g30540.1	7
1152	LOC_Os03g63650.1	3
1152	LOC_Os08g36020.1	8
1153	LOC_Os12g18210.1	12
1153	LOC_Os12g18130.1	12
1154	LOC_Os01g38650.1	1
1154	LOC_Os06g15430.1	6
1155	LOC_Os11g45320.1	11
1155	LOC_Os11g45290.1	11
1156	LOC_Os01g50510.1	1
1156	LOC_Os08g10280.1	8
1157	LOC_Os05g12590.1	5
1157	LOC_Os11g01910.1	11
1158	LOC_Os07g25610.1	7
1158	LOC_Os09g10520.1	9
1159	LOC_Os01g39890.1	1
1159	LOC_Os05g09380.1	5
1160	LOC_Os08g03560.1	8
1160	LOC_Os12g01449.1	12
1161	LOC_Os05g12000.1	5
1161	LOC_Os11g39050.1	11
1162	LOC_Os02g53530.1	2
1162	LOC_Os08g36110.1	8
1163	LOC_Os03g11680.1	3
1163	LOC_Os09g27320.1	9
1164	LOC_Os05g29100.1	5
1164	LOC_Os06g47170.1	6
1165	LOC_Os03g55230.1	3
1165	LOC_Os10g21060.1	10
1166	LOC_Os07g45490.1	7
1166	LOC_Os07g45560.1	7
1167	LOC_Os12g07200.1	12
1167	LOC_Os12g28860.1	12
1168	LOC_Os06g50724.1	6
1168	LOC_Os06g50688.1	6
1169	LOC_Os08g03770.1	8
1169	LOC_Os08g04100.1	8

1170	LOC_Os05g01900.1	5
1170	LOC_Os08g44160.1	8
1171	LOC_Os01g01820.1	1
1171	LOC_Os10g34140.1	10
1172	LOC_Os01g61870.1	1
1172	LOC_Os12g15930.1	12
1173	LOC_Os05g07960.1	5
1173	LOC_Os07g35070.1	7
1174	LOC_Os10g03860.1	10
1174	LOC_Os12g08550.1	12
1175	LOC_Os06g30330.1	6
1175	LOC_Os12g08290.1	12
1176	LOC_Os07g45570.1	7
1176	LOC_Os07g46130.1	7
1177	LOC_Os07g45510.1	7
1177	LOC_Os07g46040.1	7
1178	LOC_Os11g17210.1	11
1178	LOC_Os11g17340.1	11
1179	LOC_Os03g05860.1	3
1179	LOC_Os03g05990.1	3
1180	LOC_Os06g17090.1	6
1180	LOC_Os06g17220.1	6
1181	LOC_Os07g12670.1	7
1181	LOC_Os11g16490.1	11
1182	LOC_Os11g11420.1	11
1182	LOC_Os11g11430.1	11
1183	LOC_Os03g56780.1	3
1183	LOC_Os03g56783.1	3
1184	LOC_Os06g34970.1	6
1184	LOC_Os06g47780.1	6
1185	LOC_Os04g05060.1	4
1185	LOC_Os10g36320.1	10
1186	LOC_Os04g04240.1	4
1186	LOC_Os07g23750.1	7
1187	LOC_Os02g19790.1	2
1187	LOC_Os06g04420.1	6
1188	LOC_Os01g36840.1	1
1188	LOC_Os04g24550.1	4
1189	LOC_Os02g18050.1	2
1189	LOC_Os08g05700.1	8
1190	LOC_Os07g11700.1	7
1190	LOC_Os11g33946.1	11
1191	LOC_Os09g16740.1	9
1191	LOC_Os10g09080.1	10
1192	LOC_Os03g10700.1	3
1192	LOC_Os12g06140.1	12
1193	LOC_Os04g12690.1	4
1193	LOC_Os04g12950.1	4
1194	LOC_Os01g41190.1	1
1194	LOC_Os01g68720.1	1
1195	LOC_Os01g11580.1	1
1195	LOC_Os04g53070.1	4

1196	LOC_Os05g14850.1	5
1196	LOC_Os12g10940.1	12
1197	LOC_Os12g15440.1	12
1197	LOC_Os12g28110.1	12
1198	LOC_Os11g47431.1	11
1198	LOC_Os11g47437.1	11
1199	LOC_Os04g53520.1	4
1199	LOC_Os08g43840.1	8
1200	LOC_Os02g05840.1	2
1200	LOC_Os08g12430.1	8
1201	LOC_Os10g33550.1	10
1201	LOC_Os10g33530.1	10
1202	LOC_Os07g32370.1	7
1202	LOC_Os12g08490.1	12
1203	LOC_Os04g39940.1	4
1203	LOC_Os12g09080.1	12
1204	LOC_Os04g29810.1	4
1204	LOC_Os05g18800.1	5
1205	LOC_Os04g14750.1	4
1205	LOC_Os07g27290.1	7
1206	LOC_Os06g07700.1	6
1206	LOC_Os06g07750.1	6
1207	LOC_Os03g26250.1	3
1207	LOC_Os07g31810.1	7
1208	LOC_Os03g51060.1	3
1208	LOC_Os05g38010.1	5
1209	LOC_Os06g11400.1	6
1209	LOC_Os09g29080.1	9
1210	LOC_Os02g06830.1	2
1210	LOC_Os06g01740.1	6
1211	LOC_Os03g44950.1	3
1211	LOC_Os03g57540.1	3
1212	LOC_Os03g27340.1	3
1212	LOC_Os11g24500.1	11
1213	LOC_Os01g56900.1	1
1213	LOC_Os06g29450.1	6
1214	LOC_Os02g09970.1	2
1214	LOC_Os06g41940.1	6
1215	LOC_Os07g30950.1	7
1215	LOC_Os07g33600.1	7
1216	LOC_Os04g01730.1	4
1216	LOC_Os09g02750.1	9
1217	LOC_Os08g10910.1	8
1217	LOC_Os10g17390.1	10
1218	LOC_Os01g65470.1	1
1218	LOC_Os02g05760.1	2
1219	LOC_Os05g43070.1	5
1219	LOC_Os08g38790.1	8
1220	LOC_Os11g15040.1	11
1220	LOC_Os11g15410.1	11
1221	LOC_Os01g39560.1	1
1221	LOC_Os01g39430.1	1

1222	LOC_Os06g49570.1	6
1222	LOC_Os07g12370.1	7
1223	LOC_Os08g25050.1	8
1223	LOC_Os11g24050.1	11
1224	LOC_Os01g59740.1	1
1224	LOC_Os12g18630.1	12
1225	LOC_Os07g25550.1	7
1225	LOC_Os12g08960.1	12
1226	LOC_Os01g24590.1	1
1226	LOC_Os08g35660.1	8
1227	LOC_Os06g28860.1	6
1227	LOC_Os06g47980.1	6
1228	LOC_Os08g28400.1	8
1228	LOC_Os08g28430.1	8
1229	LOC_Os06g14220.1	6
1229	LOC_Os07g41560.1	7
1230	LOC_Os02g46810.1	2
1230	LOC_Os08g32070.1	8
1231	LOC_Os01g67790.1	1
1231	LOC_Os05g02540.1	5
1232	LOC_Os06g21830.1	6
1232	LOC_Os06g21720.1	6
1233	LOC_Os03g21130.1	3
1233	LOC_Os07g04050.1	7
1234	LOC_Os10g25740.1	10
1234	LOC_Os11g13650.1	11
1235	LOC_Os01g51520.1	1
1235	LOC_Os01g51470.1	1
1236	LOC_Os04g05870.1	4
1236	LOC_Os04g18430.1	4
1237	LOC_Os01g53680.1	1
1237	LOC_Os04g39420.1	4
1238	LOC_Os06g11120.1	6
1238	LOC_Os06g23180.1	6
1239	LOC_Os03g42120.1	3
1239	LOC_Os03g62440.1	3
1240	LOC_Os01g14840.1	1
1240	LOC_Os05g19970.1	5
1241	LOC_Os03g25710.1	3
1241	LOC_Os03g26310.1	3
1242	LOC_Os02g08510.1	2
1242	LOC_Os07g40300.1	7
1243	LOC_Os09g26100.1	9
1243	LOC_Os09g38610.1	9
1244	LOC_Os03g41110.1	3
1244	LOC_Os12g39220.1	12
1245	LOC_Os05g13480.1	5
1245	LOC_Os09g24600.1	9
1246	LOC_Os11g44470.1	11
1246	LOC_Os11g44490.1	11
1247	LOC_Os02g31980.1	2
1247	LOC_Os09g16150.1	9

1248	LOC_Os01g08680.1	1
1248	LOC_Os07g44030.1	7
1249	LOC_Os12g14830.1	12
1249	LOC_Os12g34990.1	12
1250	LOC_Os11g31660.1	11
1250	LOC_Os11g31710.1	11
1251	LOC_Os04g38390.1	4
1251	LOC_Os06g11810.1	6
1252	LOC_Os03g27510.1	3
1252	LOC_Os03g27530.1	3
1253	LOC_Os12g06440.1	12
1253	LOC_Os12g06480.1	12
1254	LOC_Os03g09940.1	3
1254	LOC_Os07g18700.1	7
1255	LOC_Os06g08980.1	6
1255	LOC_Os06g09010.1	6
1256	LOC_Os05g40940.1	5
1256	LOC_Os09g01130.1	9
1257	LOC_Os03g40900.1	3
1257	LOC_Os08g12230.1	8
1258	LOC_Os05g30770.1	5
1258	LOC_Os11g30040.1	11
1259	LOC_Os01g35300.1	1
1259	LOC_Os11g10620.1	11
1260	LOC_Os02g14030.1	2
1260	LOC_Os08g33410.1	8
1261	LOC_Os12g38150.1	12
1261	LOC_Os12g38170.1	12
1262	LOC_Os01g24420.1	1
1262	LOC_Os06g19510.1	6
1263	LOC_Os01g52180.1	1
1263	LOC_Os01g54810.1	1
1264	LOC_Os07g28250.1	7
1264	LOC_Os07g34440.1	7
1265	LOC_Os03g58190.1	3
1265	LOC_Os07g08220.1	7
1266	LOC_Os01g65610.1	1
1266	LOC_Os02g47040.1	2
1267	LOC_Os03g33380.1	3
1267	LOC_Os11g27670.1	11
1268	LOC_Os07g47130.1	7
1268	LOC_Os12g01510.1	12
1269	LOC_Os12g43080.1	12
1269	LOC_Os12g43300.1	12
1270	LOC_Os09g33720.1	9
1270	LOC_Os12g43070.1	12
1271	LOC_Os01g39120.1	1
1271	LOC_Os10g32780.1	10
1272	LOC_Os05g45700.1	5
1272	LOC_Os11g28470.1	11
1273	LOC_Os08g29900.1	8
1273	LOC_Os08g29950.1	8

1274	LOC_Os04g19030.1	4
1274	LOC_Os12g28390.1	12
1275	LOC_Os10g21670.1	10
1275	LOC_Os10g21790.1	10
1276	LOC_Os03g10730.1	3
1276	LOC_Os07g45050.1	7
1277	LOC_Os04g16160.1	4
1277	LOC_Os11g33250.1	11
1278	LOC_Os07g32360.1	7
1278	LOC_Os08g19050.1	8
1279	LOC_Os04g30700.1	4
1279	LOC_Os06g18164.1	6
1280	LOC_Os03g33800.1	3
1280	LOC_Os10g08500.1	10
1281	LOC_Os01g46740.1	1
1281	LOC_Os01g46670.1	1
1282	LOC_Os11g01690.1	11
1282	LOC_Os11g11250.1	11
1283	LOC_Os08g17560.1	8
1283	LOC_Os08g30470.1	8
1284	LOC_Os01g09330.1	1
1284	LOC_Os03g30510.1	3
1285	LOC_Os03g53180.1	3
1285	LOC_Os05g37650.1	5
1286	LOC_Os02g36130.1	2
1286	LOC_Os06g49330.1	6
1287	LOC_Os06g13710.1	6
1287	LOC_Os08g34680.1	8
1288	LOC_Os08g13850.1	8
1288	LOC_Os10g23840.1	10
1289	LOC_Os04g28850.1	4
1289	LOC_Os04g31460.1	4
1290	LOC_Os06g29950.1	6
1290	LOC_Os10g20380.1	10
1291	LOC_Os03g33040.1	3
1291	LOC_Os04g14640.1	4
1292	LOC_Os01g40170.1	1
1292	LOC_Os12g31880.1	12
1293	LOC_Os03g39970.1	3
1293	LOC_Os06g02790.1	6
1294	LOC_Os08g24910.1	8
1294	LOC_Os09g34080.1	9
1295	LOC_Os01g13350.1	1
1295	LOC_Os08g21580.1	8
1296	LOC_Os11g47433.1	11
1296	LOC_Os11g47439.1	11
1297	LOC_Os02g17840.1	2
1297	LOC_Os04g32390.1	4
1298	LOC_Os01g66620.1	1
1298	LOC_Os04g52510.1	4
1299	LOC_Os01g40370.1	1
1299	LOC_Os01g40390.1	1

1300	LOC_Os03g32260.1	3
1300	LOC_Os11g33150.1	11
1301	LOC_Os07g07380.1	7
1301	LOC_Os09g20750.1	9
1302	LOC_Os09g18390.1	9
1302	LOC_Os10g40990.1	10
1303	LOC_Os01g17300.1	1
1303	LOC_Os01g64290.1	1
1304	LOC_Os07g04980.1	7
1304	LOC_Os07g36400.1	7
1305	LOC_Os04g30750.1	4
1305	LOC_Os09g37110.1	9
1306	LOC_Os02g19290.1	2
1306	LOC_Os05g28260.1	5
1307	LOC_Os01g08580.1	1
1307	LOC_Os05g12390.1	5
1308	LOC_Os01g63620.1	1
1308	LOC_Os10g35800.1	10
1309	LOC_Os08g14060.1	8
1309	LOC_Os08g18860.1	8
1310	LOC_Os01g22570.1	1
1310	LOC_Os03g07060.1	3
1311	LOC_Os06g01879.1	6
1311	LOC_Os06g01899.1	6
1312	LOC_Os03g36380.1	3
1312	LOC_Os04g20710.1	4
1313	LOC_Os05g14150.1	5
1313	LOC_Os06g16810.1	6
1314	LOC_Os01g14080.1	1
1314	LOC_Os01g47610.1	1
1315	LOC_Os02g17480.1	2
1315	LOC_Os09g17146.1	9
1316	LOC_Os05g09160.1	5
1316	LOC_Os06g40610.1	6
1317	LOC_Os06g29070.1	6
1317	LOC_Os12g18230.1	12
1318	LOC_Os01g24270.1	1
1318	LOC_Os04g08812.1	4
1319	LOC_Os11g42360.1	11
1319	LOC_Os12g26720.1	12
1320	LOC_Os03g27570.1	3
1320	LOC_Os03g27580.1	3
1321	LOC_Os07g41600.1	7
1321	LOC_Os07g48390.1	7
1322	LOC_Os03g02370.1	3
1322	LOC_Os10g17710.1	10
1323	LOC_Os06g19230.1	6
1323	LOC_Os08g41140.1	8
1324	LOC_Os02g48430.1	2
1324	LOC_Os11g18160.1	11
1325	LOC_Os08g40100.1	8
1325	LOC_Os09g31466.1	9

1326	LOC_Os08g04670.1	8
1326	LOC_Os08g04690.1	8
1327	LOC_Os03g33490.1	3
1327	LOC_Os07g25250.1	7
1328	LOC_Os12g33270.1	12
1328	LOC_Os12g33780.1	12
1329	LOC_Os03g11490.1	3
1329	LOC_Os03g30740.1	3
1330	LOC_Os03g18990.1	3
1330	LOC_Os04g50212.1	4
1331	LOC_Os02g06960.1	2
1331	LOC_Os12g41660.1	12
1332	LOC_Os04g52800.1	4
1332	LOC_Os06g46250.1	6
1333	LOC_Os07g36950.1	7
1333	LOC_Os07g36960.1	7
1334	LOC_Os08g05470.1	8
1334	LOC_Os08g05350.1	8
1335	LOC_Os04g31550.1	4
1335	LOC_Os04g31410.1	4
1336	LOC_Os06g31500.1	6
1336	LOC_Os06g31580.1	6
1337	LOC_Os01g50520.1	1
1337	LOC_Os01g50570.1	1
1338	LOC_Os07g08090.1	7
1338	LOC_Os10g06670.1	10
1339	LOC_Os01g03220.1	1
1339	LOC_Os01g03210.1	1
1340	LOC_Os05g24480.1	5
1340	LOC_Os11g25550.1	11
1341	LOC_Os04g11730.1	4
1341	LOC_Os04g11530.1	4
1342	LOC_Os02g17870.1	2
1342	LOC_Os08g14460.1	8
1343	LOC_Os05g06070.1	5
1343	LOC_Os05g06080.1	5
1344	LOC_Os03g31120.1	3
1344	LOC_Os05g48580.1	5
1345	LOC_Os04g49710.1	4
1345	LOC_Os06g39420.1	6
1346	LOC_Os02g39750.1	2
1346	LOC_Os06g21930.1	6
1347	LOC_Os01g49740.1	1
1347	LOC_Os03g11650.1	3
1348	LOC_Os01g09110.1	1
1348	LOC_Os02g27700.1	2
1349	LOC_Os08g04650.1	8
1349	LOC_Os08g04660.1	8
1350	LOC_Os05g31830.1	5
1350	LOC_Os05g31890.1	5
1351	LOC_Os07g34400.1	7
1351	LOC_Os07g34420.1	7

1352	LOC_Os08g25720.1	8
1352	LOC_Os09g12650.1	9
1353	LOC_Os05g14390.1	5
1353	LOC_Os06g29560.1	6
1354	LOC_Os01g40140.1	1
1354	LOC_Os10g22700.1	10
1355	LOC_Os03g38410.1	3
1355	LOC_Os11g25890.1	11
1356	LOC_Os01g15100.1	1
1356	LOC_Os03g15080.1	3
1357	LOC_Os01g16360.1	1
1357	LOC_Os11g39280.1	11
1358	LOC_Os11g03220.1	11
1358	LOC_Os12g02970.1	12
1359	LOC_Os05g09680.1	5
1359	LOC_Os05g09732.1	5
1360	LOC_Os04g53250.1	4
1360	LOC_Os04g53290.1	4
1361	LOC_Os06g41270.1	6
1361	LOC_Os10g15140.1	10
1362	LOC_Os01g49460.1	1
1362	LOC_Os12g24620.1	12
1363	LOC_Os05g26070.1	5
1363	LOC_Os05g26020.1	5
1364	LOC_Os12g23710.1	12
1364	LOC_Os12g23750.1	12
1365	LOC_Os01g18230.1	1
1365	LOC_Os05g21140.1	5
1366	LOC_Os01g73670.1	1
1366	LOC_Os01g73860.1	1
1367	LOC_Os11g08360.1	11
1367	LOC_Os11g08420.1	11
1368	LOC_Os01g26350.1	1
1368	LOC_Os07g23870.1	7
1369	LOC_Os07g13350.1	7
1369	LOC_Os12g39730.1	12
1370	LOC_Os02g07470.1	2
1370	LOC_Os11g38840.1	11
1371	LOC_Os04g21490.1	4
1371	LOC_Os12g19910.1	12
1372	LOC_Os08g29350.1	8
1372	LOC_Os11g22450.1	11
1373	LOC_Os01g58194.1	1
1373	LOC_Os11g11440.1	11
1374	LOC_Os01g54920.1	1
1374	LOC_Os05g43970.1	5
1375	LOC_Os06g34670.1	6
1375	LOC_Os11g37580.1	11
1376	LOC_Os05g10380.1	5
1376	LOC_Os11g40110.1	11
1377	LOC_Os05g33420.1	5
1377	LOC_Os05g33430.1	5

1378	LOC_Os04g53502.1	4
1378	LOC_Os10g29870.1	10
1379	LOC_Os12g39720.1	12
1379	LOC_Os12g39780.1	12
1380	LOC_Os05g09000.1	5
1380	LOC_Os11g41410.1	11
1381	LOC_Os03g19220.1	3
1381	LOC_Os12g24580.1	12
1382	LOC_Os01g39250.1	1
1382	LOC_Os01g39260.1	1
1382	LOC_Os01g43150.1	1
1383	LOC_Os01g04840.1	1
1383	LOC_Os01g04814.1	1
1383	LOC_Os02g06490.1	2
1384	LOC_Os12g28137.1	12
1384	LOC_Os12g28177.1	12
1384	LOC_Os12g44190.1	12
1385	LOC_Os01g19260.1	1
1385	LOC_Os07g09470.1	7
1385	LOC_Os09g27340.1	9
1386	LOC_Os12g28550.1	12
1386	LOC_Os12g28460.1	12
1386	LOC_Os12g44210.1	12
1387	LOC_Os02g56000.1	2
1387	LOC_Os06g07630.1	6
1387	LOC_Os09g38730.1	9
1388	LOC_Os09g29670.1	9
1388	LOC_Os09g29660.1	9
1388	LOC_Os09g03939.1	9
1389	LOC_Os06g51460.1	6
1389	LOC_Os10g30610.1	10
1389	LOC_Os11g22350.1	11
1390	LOC_Os09g34010.1	9
1390	LOC_Os12g07740.1	12
1390	LOC_Os12g41400.1	12
1391	LOC_Os01g11910.1	1
1391	LOC_Os01g06640.1	1
1391	LOC_Os03g59670.1	3
1392	LOC_Os01g39580.1	1
1392	LOC_Os04g47080.1	4
1392	LOC_Os07g11020.1	7
1393	LOC_Os03g15440.1	3
1393	LOC_Os08g37290.1	8
1393	LOC_Os09g28900.1	9
1394	LOC_Os02g10080.1	2
1394	LOC_Os04g57010.1	4
1394	LOC_Os06g41390.1	6
1395	LOC_Os03g25970.1	3
1395	LOC_Os08g10030.1	8
1395	LOC_Os09g32300.1	9
1396	LOC_Os02g42134.1	2
1396	LOC_Os08g42530.1	8

1396	LOC_Os09g33600.1	9
1397	LOC_Os09g17130.1	9
1397	LOC_Os11g14260.1	11
1397	LOC_Os11g15460.1	11
1398	LOC_Os11g24850.1	11
1398	LOC_Os11g24690.1	11
1398	LOC_Os11g24770.1	11
1399	LOC_Os07g29860.1	7
1399	LOC_Os09g16050.1	9
1399	LOC_Os11g24670.1	11
1400	LOC_Os01g66180.1	1
1400	LOC_Os05g34770.1	5
1400	LOC_Os07g38000.1	7
1401	LOC_Os05g26660.1	5
1401	LOC_Os06g47000.1	6
1401	LOC_Os08g04630.1	8
1402	LOC_Os02g06340.1	2
1402	LOC_Os04g57350.1	4
1402	LOC_Os06g47330.1	6
1403	LOC_Os05g06920.1	5
1403	LOC_Os05g06940.1	5
1403	LOC_Os05g06890.1	5
1404	LOC_Os01g72080.1	1
1404	LOC_Os11g04560.1	11
1404	LOC_Os12g04360.1	12
1405	LOC_Os03g04220.1	3
1405	LOC_Os03g04260.1	3
1405	LOC_Os10g39740.1	10
1406	LOC_Os02g34080.1	2
1406	LOC_Os02g53740.1	2
1406	LOC_Os06g29350.1	6
1407	LOC_Os01g43700.1	1
1407	LOC_Os07g45290.1	7
1407	LOC_Os12g02640.1	12
1408	LOC_Os01g29150.1	1
1408	LOC_Os06g39880.1	6
1408	LOC_Os08g33300.1	8
1409	LOC_Os02g21810.1	2
1409	LOC_Os05g12040.1	5
1409	LOC_Os11g32240.1	11
1410	LOC_Os01g41800.1	1
1410	LOC_Os07g23710.1	7
1410	LOC_Os07g48330.1	7
1411	LOC_Os07g33480.1	7
1411	LOC_Os07g33620.1	7
1411	LOC_Os09g21260.1	9
1412	LOC_Os02g19630.1	2
1412	LOC_Os07g03840.1	7
1412	LOC_Os07g38810.1	7
1413	LOC_Os02g48200.1	2
1413	LOC_Os06g22290.1	6
1413	LOC_Os08g40270.1	8

1414	LOC_Os01g39970.1	1
1414	LOC_Os02g12660.1	2
1414	LOC_Os02g54590.1	2
1415	LOC_Os03g17980.1	3
1415	LOC_Os05g45420.1	5
1415	LOC_Os08g37800.1	8
1416	LOC_Os07g35650.1	7
1416	LOC_Os07g35300.1	7
1416	LOC_Os07g35580.1	7
1417	LOC_Os01g18800.1	1
1417	LOC_Os05g04550.1	5
1417	LOC_Os07g44290.1	7
1418	LOC_Os03g01160.1	3
1418	LOC_Os06g04880.1	6
1418	LOC_Os10g41220.1	10
1419	LOC_Os02g57700.1	2
1419	LOC_Os09g39640.1	9
1419	LOC_Os10g01060.1	10
1420	LOC_Os01g26300.1	1
1420	LOC_Os04g21820.1	4
1420	LOC_Os04g24290.1	4
1421	LOC_Os01g71000.1	1
1421	LOC_Os02g30900.1	2
1421	LOC_Os05g30870.1	5
1422	LOC_Os04g35890.1	4
1422	LOC_Os08g01830.1	8
1422	LOC_Os11g11490.1	11
1423	LOC_Os10g35450.1	10
1423	LOC_Os11g01740.1	11
1423	LOC_Os12g01740.1	12
1424	LOC_Os01g21960.1	1
1424	LOC_Os03g03410.1	3
1424	LOC_Os05g36050.1	5
1425	LOC_Os06g05830.1	6
1425	LOC_Os08g28710.1	8
1425	LOC_Os11g44500.1	11
1426	LOC_Os01g51200.1	1
1426	LOC_Os02g56560.1	2
1426	LOC_Os04g43490.1	4
1427	LOC_Os03g13080.1	3
1427	LOC_Os06g41960.1	6
1427	LOC_Os11g35330.1	11
1428	LOC_Os03g21160.1	3
1428	LOC_Os03g21140.1	3
1428	LOC_Os07g48410.1	7
1429	LOC_Os02g29480.1	2
1429	LOC_Os03g30550.1	3
1429	LOC_Os07g41180.1	7
1430	LOC_Os01g11120.1	1
1430	LOC_Os02g14760.1	2
1430	LOC_Os09g14550.1	9
1431	LOC_Os01g56000.1	1

1431	LOC_Os09g12730.1	9
1431	LOC_Os09g34070.1	9
1432	LOC_Os02g15310.1	2
1432	LOC_Os03g22380.1	3
1432	LOC_Os07g47630.1	7
1433	LOC_Os10g06130.1	10
1433	LOC_Os11g04390.1	11
1433	LOC_Os12g04180.1	12
1434	LOC_Os02g12850.1	2
1434	LOC_Os08g23120.1	8
1434	LOC_Os12g43600.1	12
1435	LOC_Os07g39560.1	7
1435	LOC_Os10g33230.1	10
1435	LOC_Os11g41890.1	11
1436	LOC_Os05g25850.1	5
1436	LOC_Os06g02500.1	6
1436	LOC_Os06g05110.1	6
1437	LOC_Os01g22710.1	1
1437	LOC_Os07g37530.1	7
1437	LOC_Os07g37510.1	7
1438	LOC_Os04g38640.1	4
1438	LOC_Os05g49380.1	5
1438	LOC_Os11g14170.1	11
1439	LOC_Os03g29350.1	3
1439	LOC_Os07g42280.1	7
1439	LOC_Os11g43030.1	11
1440	LOC_Os02g56280.1	2
1440	LOC_Os03g04890.1	3
1440	LOC_Os04g42980.1	4
1441	LOC_Os08g03260.1	8
1441	LOC_Os08g03270.1	8
1441	LOC_Os11g45390.1	11
1442	LOC_Os01g70870.1	1
1442	LOC_Os08g36390.1	8
1442	LOC_Os09g27650.1	9
1443	LOC_Os03g13600.1	3
1443	LOC_Os04g50070.1	4
1443	LOC_Os05g20930.1	5
1444	LOC_Os04g46670.1	4
1444	LOC_Os05g14130.1	5
1444	LOC_Os12g38960.1	12
1445	LOC_Os03g32220.1	3
1445	LOC_Os03g32230.1	3
1445	LOC_Os07g40080.1	7
1446	LOC_Os01g74040.1	1
1446	LOC_Os03g16480.1	3
1446	LOC_Os05g01940.1	5
1447	LOC_Os01g20930.1	1
1447	LOC_Os05g29676.1	5
1447	LOC_Os05g29710.1	5
1448	LOC_Os01g03100.1	1
1448	LOC_Os05g47900.1	5

1448	LOC_Os11g38800.1	11
1449	LOC_Os02g50290.1	2
1449	LOC_Os04g48050.1	4
1449	LOC_Os07g27950.1	7
1450	LOC_Os01g58400.1	1
1450	LOC_Os05g41800.1	5
1450	LOC_Os11g36560.1	11
1451	LOC_Os06g34620.1	6
1451	LOC_Os06g34860.1	6
1451	LOC_Os06g34870.1	6
1452	LOC_Os01g09640.1	1
1452	LOC_Os01g41900.1	1
1452	LOC_Os05g10690.1	5
1453	LOC_Os05g25380.1	5
1453	LOC_Os10g14930.1	10
1453	LOC_Os11g23940.1	11
1454	LOC_Os05g20920.1	5
1454	LOC_Os05g20730.1	5
1454	LOC_Os08g26400.1	8
1455	LOC_Os08g18820.1	8
1455	LOC_Os08g18740.1	8
1455	LOC_Os08g18830.1	8
1456	LOC_Os04g08784.1	4
1456	LOC_Os04g17300.1	4
1456	LOC_Os12g15580.1	12
1457	LOC_Os05g18720.1	5
1457	LOC_Os09g09720.1	9
1457	LOC_Os12g24730.1	12
1458	LOC_Os05g01180.1	5
1458	LOC_Os08g37160.1	8
1458	LOC_Os11g15640.1	11
1459	LOC_Os01g51340.1	1
1459	LOC_Os04g48590.1	4
1459	LOC_Os05g24100.1	5
1460	LOC_Os02g08420.1	2
1460	LOC_Os02g56700.1	2
1460	LOC_Os03g60380.1	3
1461	LOC_Os01g45200.1	1
1461	LOC_Os05g50250.1	5
1461	LOC_Os09g31510.1	9
1462	LOC_Os07g46830.1	7
1462	LOC_Os07g46840.1	7
1462	LOC_Os07g46940.1	7
1463	LOC_Os03g53690.1	3
1463	LOC_Os06g08600.1	6
1463	LOC_Os10g31780.1	10
1464	LOC_Os01g11840.1	1
1464	LOC_Os01g24550.1	1
1464	LOC_Os01g24600.1	1
1465	LOC_Os01g05490.1	1
1465	LOC_Os01g62420.1	1
1465	LOC_Os09g36450.1	9

1466	LOC_Os03g17310.1	3
1466	LOC_Os03g42020.1	3
1466	LOC_Os10g28240.1	10
1467	LOC_Os03g31180.1	3
1467	LOC_Os12g12260.1	12
1467	LOC_Os12g38780.1	12
1468	LOC_Os02g55680.1	2
1468	LOC_Os02g55720.1	2
1468	LOC_Os02g55780.1	2
1469	LOC_Os02g04010.1	2
1469	LOC_Os02g24190.1	2
1469	LOC_Os12g30020.1	12
1470	LOC_Os01g64820.1	1
1470	LOC_Os02g30800.1	2
1470	LOC_Os11g08330.1	11
1471	LOC_Os08g24670.1	8
1471	LOC_Os08g24770.1	8
1471	LOC_Os10g03950.1	10
1472	LOC_Os03g44500.1	3
1472	LOC_Os05g05240.1	5
1472	LOC_Os12g42310.1	12
1473	LOC_Os06g43640.1	6
1473	LOC_Os11g05410.1	11
1473	LOC_Os12g44020.1	12
1474	LOC_Os04g33530.1	4
1474	LOC_Os11g05400.1	11
1474	LOC_Os12g05540.1	12
1475	LOC_Os01g67510.1	1
1475	LOC_Os03g43850.1	3
1475	LOC_Os11g19720.1	11
1476	LOC_Os02g17320.1	2
1476	LOC_Os09g32820.1	9
1476	LOC_Os11g16370.1	11
1477	LOC_Os01g61820.1	1
1477	LOC_Os03g60400.1	3
1477	LOC_Os12g33930.1	12
1478	LOC_Os05g03610.1	5
1478	LOC_Os07g49330.1	7
1478	LOC_Os12g37560.1	12
1479	LOC_Os01g72230.1	1
1479	LOC_Os02g10480.1	2
1479	LOC_Os05g31720.1	5
1480	LOC_Os07g31720.1	7
1480	LOC_Os07g31830.1	7
1480	LOC_Os07g01780.1	7
1481	LOC_Os03g14700.1	3
1481	LOC_Os04g55220.1	4
1481	LOC_Os06g47130.1	6
1482	LOC_Os01g63800.1	1
1482	LOC_Os03g46340.1	3
1482	LOC_Os07g04550.1	7
1483	LOC_Os11g05640.1	11

1483	LOC_Os11g11100.1	11
1483	LOC_Os12g43790.1	12
1484	LOC_Os02g53130.1	2
1484	LOC_Os08g36480.1	8
1484	LOC_Os08g36500.1	8
1485	LOC_Os06g14440.1	6
1485	LOC_Os07g25390.1	7
1485	LOC_Os08g14610.1	8
1486	LOC_Os02g02400.1	2
1486	LOC_Os03g03910.1	3
1486	LOC_Os06g51150.1	6
1487	LOC_Os01g41430.1	1
1487	LOC_Os01g45140.1	1
1487	LOC_Os01g45110.1	1
1488	LOC_Os03g19280.1	3
1488	LOC_Os03g19930.1	3
1488	LOC_Os03g21950.1	3
1489	LOC_Os01g37760.1	1
1489	LOC_Os02g43470.1	2
1489	LOC_Os03g58040.1	3
1490	LOC_Os02g51470.1	2
1490	LOC_Os03g52660.1	3
1490	LOC_Os06g43850.1	6
1491	LOC_Os04g39270.1	4
1491	LOC_Os08g23150.1	8
1491	LOC_Os09g08130.1	9
1492	LOC_Os02g28850.1	2
1492	LOC_Os03g39020.1	3
1492	LOC_Os04g28260.1	4
1493	LOC_Os01g04040.1	1
1493	LOC_Os01g04050.1	1
1493	LOC_Os03g60840.1	3
1494	LOC_Os06g05880.1	6
1494	LOC_Os10g17660.1	10
1494	LOC_Os10g17680.1	10
1495	LOC_Os01g42980.1	1
1495	LOC_Os08g41810.1	8
1495	LOC_Os09g08430.1	9
1496	LOC_Os02g10510.1	2
1496	LOC_Os03g03920.1	3
1496	LOC_Os10g39620.1	10
1497	LOC_Os02g08300.1	2
1497	LOC_Os06g15360.1	6
1497	LOC_Os08g33340.1	8
1498	LOC_Os01g61500.1	1
1498	LOC_Os06g03640.1	6
1498	LOC_Os09g35630.1	9
1499	LOC_Os01g27470.1	1
1499	LOC_Os01g68940.1	1
1499	LOC_Os01g68950.1	1
1500	LOC_Os01g40670.1	1
1500	LOC_Os01g51154.1	1

1500	LOC_Os12g41920.1	12
1501	LOC_Os06g07640.1	6
1501	LOC_Os06g07650.1	6
1501	LOC_Os06g07740.1	6
1502	LOC_Os01g06320.1	1
1502	LOC_Os02g45670.1	2
1502	LOC_Os06g01670.1	6
1503	LOC_Os01g57570.1	1
1503	LOC_Os05g42190.1	5
1503	LOC_Os08g04460.1	8
1504	LOC_Os02g01740.1	2
1504	LOC_Os02g40450.1	2
1504	LOC_Os03g53220.1	3
1505	LOC_Os01g49680.1	1
1505	LOC_Os07g45360.1	7
1505	LOC_Os11g07870.1	11
1506	LOC_Os02g06500.1	2
1506	LOC_Os02g50560.1	2
1506	LOC_Os11g07500.1	11
1507	LOC_Os03g59050.1	3
1507	LOC_Os06g40020.1	6
1507	LOC_Os11g38670.1	11
1508	LOC_Os02g10070.1	2
1508	LOC_Os02g13840.1	2
1508	LOC_Os11g33240.1	11
1509	LOC_Os08g06530.1	8
1509	LOC_Os08g23410.1	8
1509	LOC_Os11g32500.1	11
1510	LOC_Os01g33080.1	1
1510	LOC_Os02g37580.1	2
1510	LOC_Os02g48740.1	2
1511	LOC_Os07g38540.1	7
1511	LOC_Os08g02410.1	8
1511	LOC_Os08g02400.1	8
1512	LOC_Os06g30810.1	6
1512	LOC_Os06g30830.1	6
1512	LOC_Os12g21850.1	12
1513	LOC_Os02g47210.1	2
1513	LOC_Os03g25920.1	3
1513	LOC_Os03g37984.1	3
1514	LOC_Os01g01830.1	1
1514	LOC_Os04g47360.1	4
1514	LOC_Os06g51410.1	6
1515	LOC_Os04g51630.1	4
1515	LOC_Os08g13690.1	8
1515	LOC_Os08g42920.1	8
1516	LOC_Os01g56980.1	1
1516	LOC_Os03g48300.1	3
1516	LOC_Os03g60370.1	3
1517	LOC_Os03g04410.1	3
1517	LOC_Os06g19960.1	6
1517	LOC_Os08g09200.1	8

1518	LOC_Os03g45410.1	3
1518	LOC_Os10g29660.1	10
1518	LOC_Os12g39070.1	12
1519	LOC_Os03g19590.1	3
1519	LOC_Os03g51030.1	3
1519	LOC_Os03g54084.1	3
1520	LOC_Os02g48360.1	2
1520	LOC_Os06g13810.1	6
1520	LOC_Os06g22060.1	6
1521	LOC_Os02g48330.1	2
1521	LOC_Os08g40180.1	8
1521	LOC_Os09g31970.1	9
1522	LOC_Os03g10070.1	3
1522	LOC_Os05g23940.1	5
1522	LOC_Os07g32120.1	7
1523	LOC_Os06g37080.1	6
1523	LOC_Os09g20090.1	9
1523	LOC_Os09g32952.1	9
1524	LOC_Os02g03050.1	2
1524	LOC_Os07g32550.1	7
1524	LOC_Os10g10434.1	10
1525	LOC_Os03g50340.1	3
1525	LOC_Os03g50350.1	3
1525	LOC_Os09g04110.1	9
1526	LOC_Os01g08190.1	1
1526	LOC_Os01g42260.1	1
1526	LOC_Os01g42270.1	1
1527	LOC_Os01g52640.1	1
1527	LOC_Os02g02380.1	2
1527	LOC_Os05g49590.1	5
1528	LOC_Os02g42590.1	2
1528	LOC_Os04g58180.1	4
1528	LOC_Os11g08400.1	11
1529	LOC_Os01g15020.1	1
1529	LOC_Os03g14980.1	3
1529	LOC_Os08g06480.1	8
1530	LOC_Os02g19210.1	2
1530	LOC_Os06g43690.1	6
1530	LOC_Os10g32770.1	10
1531	LOC_Os03g42710.1	3
1531	LOC_Os03g52870.1	3
1531	LOC_Os12g40260.1	12
1532	LOC_Os01g57210.1	1
1532	LOC_Os04g58130.1	4
1532	LOC_Os10g35200.1	10
1533	LOC_Os01g07400.1	1
1533	LOC_Os01g70780.1	1
1533	LOC_Os05g07710.1	5
1534	LOC_Os03g50480.1	3
1534	LOC_Os07g26610.1	7
1534	LOC_Os10g11140.1	10
1535	LOC_Os02g15610.1	2

1535	LOC_Os02g27760.1	2
1535	LOC_Os07g10720.1	7
1536	LOC_Os03g42820.1	3
1536	LOC_Os06g08400.1	6
1536	LOC_Os12g40490.1	12
1537	LOC_Os02g50730.1	2
1537	LOC_Os06g13180.1	6
1537	LOC_Os10g40830.1	10
1538	LOC_Os01g52630.1	1
1538	LOC_Os01g62810.1	1
1538	LOC_Os05g38270.1	5
1539	LOC_Os01g73410.1	1
1539	LOC_Os04g58960.1	4
1539	LOC_Os05g01610.1	5
1540	LOC_Os08g03640.1	8
1540	LOC_Os11g04070.1	11
1540	LOC_Os12g03880.1	12
1541	LOC_Os01g53600.1	1
1541	LOC_Os01g05680.1	1
1541	LOC_Os08g40140.1	8
1542	LOC_Os01g18860.1	1
1542	LOC_Os01g22010.1	1
1542	LOC_Os05g04510.1	5
1543	LOC_Os06g01390.1	6
1543	LOC_Os06g24704.1	6
1543	LOC_Os11g39220.1	11
1544	LOC_Os01g56490.1	1
1544	LOC_Os07g06950.1	7
1544	LOC_Os12g30540.1	12
1545	LOC_Os02g14730.1	2
1545	LOC_Os06g44380.1	6
1545	LOC_Os08g37350.1	8
1546	LOC_Os01g56600.1	1
1546	LOC_Os05g43390.1	5
1546	LOC_Os11g05556.1	11
1547	LOC_Os03g27480.1	3
1547	LOC_Os03g27590.1	3
1547	LOC_Os08g44640.1	8
1548	LOC_Os05g14550.1	5
1548	LOC_Os06g50910.1	6
1548	LOC_Os07g45070.1	7
1549	LOC_Os03g17580.1	3
1549	LOC_Os11g01420.1	11
1549	LOC_Os12g01430.1	12
1550	LOC_Os01g25610.1	1
1550	LOC_Os02g01560.1	2
1550	LOC_Os05g30530.1	5
1551	LOC_Os01g14950.1	1
1551	LOC_Os01g24060.1	1
1551	LOC_Os05g06350.1	5
1552	LOC_Os02g51070.1	2
1552	LOC_Os06g04200.1	6

1552	LOC_Os06g06560.1	6
1553	LOC_Os01g64030.1	1
1553	LOC_Os10g25680.1	10
1553	LOC_Os11g16280.1	11
1554	LOC_Os03g10910.1	3
1554	LOC_Os03g14900.1	3
1554	LOC_Os04g43440.1	4
1555	LOC_Os11g30060.1	11
1555	LOC_Os11g29990.1	11
1555	LOC_Os11g30210.1	11
1556	LOC_Os04g40080.1	4
1556	LOC_Os04g51580.1	4
1556	LOC_Os08g40090.1	8
1557	LOC_Os04g21160.1	4
1557	LOC_Os06g42860.1	6
1557	LOC_Os08g41780.1	8
1558	LOC_Os02g48880.1	2
1558	LOC_Os08g38980.1	8
1558	LOC_Os12g25200.1	12
1559	LOC_Os03g53630.1	3
1559	LOC_Os04g59510.1	4
1559	LOC_Os07g49290.1	7
1560	LOC_Os01g66500.1	1
1560	LOC_Os03g61600.1	3
1560	LOC_Os05g01440.1	5
1561	LOC_Os01g37837.1	1
1561	LOC_Os03g10190.1	3
1561	LOC_Os11g39670.1	11
1562	LOC_Os01g44050.1	1
1562	LOC_Os03g23970.1	3
1562	LOC_Os10g27450.1	10
1563	LOC_Os01g09470.1	1
1563	LOC_Os01g53980.1	1
1563	LOC_Os05g09520.1	5
1564	LOC_Os03g25760.1	3
1564	LOC_Os07g43970.1	7
1564	LOC_Os10g27170.1	10
1565	LOC_Os01g73460.1	1
1565	LOC_Os02g03030.1	2
1565	LOC_Os09g38440.1	9
1566	LOC_Os04g02830.1	4
1566	LOC_Os04g56800.1	4
1566	LOC_Os09g24540.1	9
1567	LOC_Os01g15350.1	1
1567	LOC_Os01g15460.1	1
1567	LOC_Os01g68860.1	1
1568	LOC_Os02g51350.1	2
1568	LOC_Os03g07530.1	3
1568	LOC_Os04g52830.1	4
1569	LOC_Os01g69270.1	1
1569	LOC_Os04g31610.1	4
1569	LOC_Os11g29710.1	11

1570	LOC_Os01g22400.1	1
1570	LOC_Os03g51090.1	3
1570	LOC_Os11g37060.1	11
1571	LOC_Os07g23890.1	7
1571	LOC_Os08g03320.1	8
1571	LOC_Os08g03300.1	8
1572	LOC_Os10g05540.1	10
1572	LOC_Os10g06700.1	10
1572	LOC_Os10g05530.1	10
1573	LOC_Os07g03110.1	7
1573	LOC_Os07g23900.1	7
1573	LOC_Os08g20492.1	8
1574	LOC_Os01g37670.1	1
1574	LOC_Os07g02890.1	7
1574	LOC_Os07g02910.1	7
1575	LOC_Os11g09590.1	11
1575	LOC_Os11g10400.1	11
1575	LOC_Os11g10370.1	11
1576	LOC_Os02g18630.1	2
1576	LOC_Os02g18640.1	2
1576	LOC_Os02g19200.1	2
1577	LOC_Os06g02100.1	6
1577	LOC_Os06g02110.1	6
1577	LOC_Os06g02340.1	6
1578	LOC_Os08g09460.1	8
1578	LOC_Os08g09380.1	8
1578	LOC_Os08g09390.1	8
1579	LOC_Os01g39670.1	1
1579	LOC_Os01g41260.1	1
1579	LOC_Os04g13160.1	4
1580	LOC_Os02g35560.1	2
1580	LOC_Os07g16420.1	7
1580	LOC_Os07g16800.1	7
1581	LOC_Os02g56760.1	2
1581	LOC_Os12g03594.1	12
1581	LOC_Os12g03740.1	12
1582	LOC_Os12g34310.1	12
1582	LOC_Os12g34290.1	12
1582	LOC_Os12g34300.1	12
1583	LOC_Os02g17210.1	2
1583	LOC_Os10g04020.1	10
1583	LOC_Os10g04370.1	10
1584	LOC_Os01g34270.1	1
1584	LOC_Os04g08470.1	4
1584	LOC_Os12g40140.1	12
1585	LOC_Os04g11660.1	4
1585	LOC_Os04g11890.1	4
1585	LOC_Os10g03600.1	10
1586	LOC_Os01g07160.1	1
1586	LOC_Os02g10600.1	2
1586	LOC_Os05g02550.1	5
1587	LOC_Os03g25640.1	3

1587	LOC_Os03g25650.1	3
1587	LOC_Os10g25660.1	10
1588	LOC_Os04g12990.1	4
1588	LOC_Os04g13040.1	4
1588	LOC_Os04g35930.1	4
1589	LOC_Os07g12610.1	7
1589	LOC_Os07g12590.1	7
1589	LOC_Os07g36520.1	7
1590	LOC_Os01g65380.1	1
1590	LOC_Os05g35460.1	5
1590	LOC_Os05g27820.1	5
1591	LOC_Os02g20720.1	2
1591	LOC_Os04g53400.1	4
1591	LOC_Os10g29050.1	10
1592	LOC_Os08g03500.1	8
1592	LOC_Os10g29840.1	10
1592	LOC_Os10g29750.1	10
1593	LOC_Os08g13250.1	8
1593	LOC_Os10g29260.1	10
1593	LOC_Os10g29440.1	10
1594	LOC_Os08g41120.1	8
1594	LOC_Os08g41240.1	8
1594	LOC_Os08g41180.1	8
1595	LOC_Os02g20600.1	2
1595	LOC_Os09g16870.1	9
1595	LOC_Os10g28980.1	10
1596	LOC_Os03g02610.1	3
1596	LOC_Os04g53950.1	4
1596	LOC_Os07g34580.1	7
1597	LOC_Os02g40840.1	2
1597	LOC_Os10g33520.1	10
1597	LOC_Os10g33540.1	10
1598	LOC_Os03g29260.1	3
1598	LOC_Os07g46750.1	7
1598	LOC_Os07g42300.1	7
1599	LOC_Os02g02850.1	2
1599	LOC_Os05g05830.1	5
1599	LOC_Os09g15810.1	9
1600	LOC_Os01g62440.1	1
1600	LOC_Os02g51780.1	2
1600	LOC_Os03g03710.1	3
1601	LOC_Os01g05080.1	1
1601	LOC_Os02g42960.1	2
1601	LOC_Os10g35810.1	10
1602	LOC_Os03g09300.1	3
1602	LOC_Os04g20774.1	4
1602	LOC_Os10g25110.1	10
1603	LOC_Os01g62650.1	1
1603	LOC_Os01g74030.1	1
1603	LOC_Os05g01910.1	5
1604	LOC_Os03g09150.1	3
1604	LOC_Os11g37090.1	11

1604	LOC_Os12g31000.1	12
1605	LOC_Os04g12480.1	4
1605	LOC_Os09g32210.1	9
1605	LOC_Os12g30520.1	12
1606	LOC_Os01g19140.1	1
1606	LOC_Os05g10630.1	5
1606	LOC_Os12g29990.1	12
1607	LOC_Os03g21470.1	3
1607	LOC_Os05g07700.1	5
1607	LOC_Os11g11390.1	11
1608	LOC_Os03g22180.1	3
1608	LOC_Os05g06310.1	5
1608	LOC_Os07g47780.1	7
1609	LOC_Os02g30050.1	2
1609	LOC_Os02g51790.1	2
1609	LOC_Os04g30730.1	4
1610	LOC_Os02g55370.1	2
1610	LOC_Os02g55390.1	2
1610	LOC_Os06g08320.1	6
1611	LOC_Os01g40400.1	1
1611	LOC_Os11g09370.1	11
1611	LOC_Os11g09440.1	11
1612	LOC_Os01g59620.1	1
1612	LOC_Os05g41172.1	5
1612	LOC_Os11g03700.1	11
1613	LOC_Os04g18830.1	4
1613	LOC_Os11g41230.1	11
1613	LOC_Os11g41240.1	11
1614	LOC_Os01g08020.1	1
1614	LOC_Os05g08430.1	5
1614	LOC_Os12g37840.1	12
1615	LOC_Os05g28180.1	5
1615	LOC_Os07g46630.1	7
1615	LOC_Os07g49270.1	7
1616	LOC_Os02g18550.1	2
1616	LOC_Os03g10340.1	3
1616	LOC_Os12g21798.1	12
1617	LOC_Os03g58540.1	3
1617	LOC_Os07g47300.1	7
1617	LOC_Os11g47800.1	11
1618	LOC_Os01g12940.1	1
1618	LOC_Os05g13970.1	5
1618	LOC_Os06g02220.1	6
1619	LOC_Os02g57990.1	2
1619	LOC_Os03g17230.1	3
1619	LOC_Os08g03570.1	8
1620	LOC_Os01g01650.1	1
1620	LOC_Os01g13610.1	1
1620	LOC_Os06g27770.1	6
1621	LOC_Os02g35039.1	2
1621	LOC_Os04g33060.1	4
1621	LOC_Os09g34090.1	9

1622	LOC_Os03g29150.1	3
1622	LOC_Os10g28200.1	10
1622	LOC_Os11g37890.1	11
1623	LOC_Os06g13650.1	6
1623	LOC_Os10g05069.1	10
1623	LOC_Os11g32260.1	11
1624	LOC_Os01g25484.1	1
1624	LOC_Os02g52730.1	2
1624	LOC_Os05g42350.1	5
1625	LOC_Os03g59160.1	3
1625	LOC_Os11g02450.1	11
1625	LOC_Os12g02380.1	12
1626	LOC_Os02g34570.1	2
1626	LOC_Os09g07820.1	9
1626	LOC_Os11g20689.1	11
1627	LOC_Os03g02710.1	3
1627	LOC_Os08g43170.1	8
1627	LOC_Os09g34960.1	9
1628	LOC_Os06g48600.1	6
1628	LOC_Os11g01600.1	11
1628	LOC_Os12g01680.1	12
1629	LOC_Os03g38960.1	3
1629	LOC_Os07g27930.1	7
1629	LOC_Os08g32990.1	8
1630	LOC_Os02g49180.1	2
1630	LOC_Os07g07610.1	7
1630	LOC_Os12g09280.1	12
1631	LOC_Os01g62210.1	1
1631	LOC_Os03g60100.1	3
1631	LOC_Os05g38580.1	5
1632	LOC_Os02g48660.1	2
1632	LOC_Os06g21480.1	6
1632	LOC_Os08g39500.1	8
1633	LOC_Os08g06040.1	8
1633	LOC_Os08g33920.1	8
1633	LOC_Os09g24690.1	9
1634	LOC_Os02g28810.1	2
1634	LOC_Os04g28180.1	4
1634	LOC_Os07g47580.1	7
1635	LOC_Os04g21000.1	4
1635	LOC_Os04g44890.1	4
1635	LOC_Os06g49870.1	6
1636	LOC_Os01g43390.1	1
1636	LOC_Os03g21900.1	3
1636	LOC_Os03g22060.1	3
1637	LOC_Os01g50030.1	1
1637	LOC_Os05g47540.1	5
1637	LOC_Os07g10600.1	7
1638	LOC_Os01g25189.1	1
1638	LOC_Os02g26650.1	2
1638	LOC_Os09g39220.1	9
1639	LOC_Os01g03710.1	1

1639	LOC_Os09g22090.1	9
1639	LOC_Os11g38810.1	11
1640	LOC_Os02g11030.1	2
1640	LOC_Os02g32400.1	2
1640	LOC_Os02g43600.1	2
1641	LOC_Os02g54470.1	2
1641	LOC_Os05g48220.1	5
1641	LOC_Os05g48310.1	5
1642	LOC_Os03g18570.1	3
1642	LOC_Os03g18580.1	3
1642	LOC_Os05g27940.1	5
1643	LOC_Os01g63830.1	1
1643	LOC_Os06g07120.1	6
1643	LOC_Os07g41680.1	7
1644	LOC_Os01g52490.1	1
1644	LOC_Os02g13530.1	2
1644	LOC_Os06g36160.1	6
1645	LOC_Os03g15050.1	3
1645	LOC_Os04g50208.1	4
1645	LOC_Os10g13700.1	10
1646	LOC_Os01g37510.1	1
1646	LOC_Os01g44980.1	1
1646	LOC_Os01g45070.1	1
1647	LOC_Os01g65910.1	1
1647	LOC_Os08g15280.1	8
1647	LOC_Os10g21290.1	10
1648	LOC_Os01g07140.1	1
1648	LOC_Os05g07220.1	5
1648	LOC_Os07g35610.1	7
1649	LOC_Os02g45540.1	2
1649	LOC_Os06g44260.1	6
1649	LOC_Os06g44270.1	6
1650	LOC_Os03g04960.1	3
1650	LOC_Os09g38420.1	9
1650	LOC_Os10g32570.1	10
1651	LOC_Os05g33550.1	5
1651	LOC_Os06g48870.1	6
1651	LOC_Os09g32090.1	9
1652	LOC_Os04g11640.1	4
1652	LOC_Os04g19684.1	4
1652	LOC_Os04g20560.1	4
1653	LOC_Os01g67590.1	1
1653	LOC_Os02g58340.1	2
1653	LOC_Os06g47210.1	6
1654	LOC_Os03g60460.1	3
1654	LOC_Os08g44860.1	8
1654	LOC_Os09g19800.1	9
1655	LOC_Os02g12650.1	2
1655	LOC_Os08g30810.1	8
1655	LOC_Os09g19790.1	9
1656	LOC_Os01g73910.1	1
1656	LOC_Os02g45650.1	2

1656	LOC_Os02g50230.1	2
1657	LOC_Os02g13950.1	2
1657	LOC_Os06g15820.1	6
1657	LOC_Os08g31970.1	8
1658	LOC_Os03g25270.1	3
1658	LOC_Os07g44610.1	7
1658	LOC_Os09g23280.1	9
1659	LOC_Os02g51710.1	2
1659	LOC_Os04g38360.1	4
1659	LOC_Os04g38370.1	4
1660	LOC_Os03g42780.1	3
1660	LOC_Os03g42790.1	3
1660	LOC_Os03g42760.1	3
1661	LOC_Os01g61780.1	1
1661	LOC_Os03g14690.1	3
1661	LOC_Os10g10500.1	10
1662	LOC_Os05g40720.1	5
1662	LOC_Os07g48370.1	7
1662	LOC_Os11g03160.1	11
1663	LOC_Os04g59540.1	4
1663	LOC_Os08g01390.1	8
1663	LOC_Os12g13440.1	12
1664	LOC_Os04g48700.1	4
1664	LOC_Os05g01750.1	5
1664	LOC_Os11g38600.1	11
1665	LOC_Os03g11110.1	3
1665	LOC_Os07g40480.1	7
1665	LOC_Os10g19180.1	10
1666	LOC_Os07g11280.1	7
1666	LOC_Os08g09270.1	8
1666	LOC_Os08g21570.1	8
1667	LOC_Os01g08120.1	1
1667	LOC_Os01g59890.1	1
1667	LOC_Os06g08660.1	6
1668	LOC_Os01g66160.1	1
1668	LOC_Os03g13830.1	3
1668	LOC_Os03g56400.1	3
1669	LOC_Os08g01640.1	8
1669	LOC_Os08g01870.1	8
1669	LOC_Os10g35240.1	10
1670	LOC_Os02g55310.1	2
1670	LOC_Os10g35750.1	10
1670	LOC_Os12g34340.1	12
1671	LOC_Os10g35090.1	10
1671	LOC_Os10g35230.1	10
1671	LOC_Os10g35260.1	10
1672	LOC_Os02g46520.1	2
1672	LOC_Os06g45480.1	6
1672	LOC_Os07g14600.1	7
1673	LOC_Os10g25780.1	10
1673	LOC_Os11g04740.1	11
1673	LOC_Os12g04520.1	12

1674	LOC_Os08g06190.1	8
1674	LOC_Os08g06170.1	8
1674	LOC_Os08g25490.1	8
1675	LOC_Os02g07410.1	2
1675	LOC_Os06g45670.1	6
1675	LOC_Os10g37180.1	10
1676	LOC_Os03g02150.1	3
1676	LOC_Os03g23950.1	3
1676	LOC_Os06g07090.1	6
1677	LOC_Os03g02640.1	3
1677	LOC_Os04g57400.1	4
1677	LOC_Os04g57410.1	4
1678	LOC_Os03g24600.1	3
1678	LOC_Os05g33510.1	5
1678	LOC_Os06g27760.1	6
1679	LOC_Os08g14490.1	8
1679	LOC_Os11g04160.1	11
1679	LOC_Os12g03960.1	12
1680	LOC_Os01g73880.1	1
1680	LOC_Os03g15590.1	3
1680	LOC_Os10g32970.1	10
1681	LOC_Os02g50200.1	2
1681	LOC_Os04g45460.1	4
1681	LOC_Os06g14280.1	6
1682	LOC_Os03g05660.1	3
1682	LOC_Os03g21830.1	3
1682	LOC_Os10g30750.1	10
1683	LOC_Os01g60190.1	1
1683	LOC_Os03g21260.1	3
1683	LOC_Os05g40420.1	5
1684	LOC_Os01g64680.1	1
1684	LOC_Os03g11990.1	3
1684	LOC_Os06g28970.1	6
1685	LOC_Os07g37100.1	7
1685	LOC_Os07g37110.1	7
1685	LOC_Os08g10450.1	8
1686	LOC_Os03g03930.1	3
1686	LOC_Os04g32490.1	4
1686	LOC_Os12g07350.1	12
1687	LOC_Os03g49730.1	3
1687	LOC_Os04g53700.1	4
1687	LOC_Os07g29770.1	7
1688	LOC_Os01g46250.1	1
1688	LOC_Os01g46290.1	1
1688	LOC_Os01g51360.1	1
1689	LOC_Os12g41970.1	12
1689	LOC_Os12g41980.1	12
1689	LOC_Os12g42010.1	12
1690	LOC_Os03g61540.1	3
1690	LOC_Os11g43760.1	11
1690	LOC_Os12g36770.1	12
1691	LOC_Os01g47660.1	1

1691	LOC_Os01g54870.1	1
1691	LOC_Os05g49030.1	5
1692	LOC_Os01g51020.1	1
1692	LOC_Os02g57540.1	2
1692	LOC_Os05g46430.1	5
1693	LOC_Os04g36720.1	4
1693	LOC_Os04g48930.1	4
1693	LOC_Os08g35210.1	8
1694	LOC_Os01g50320.1	1
1694	LOC_Os06g29400.1	6
1694	LOC_Os06g46880.1	6
1695	LOC_Os01g54510.1	1
1695	LOC_Os01g72860.1	1
1695	LOC_Os07g07194.1	7
1696	LOC_Os03g07370.1	3
1696	LOC_Os07g07480.1	7
1696	LOC_Os10g18890.1	10
1697	LOC_Os03g01910.1	3
1697	LOC_Os03g63400.1	3
1697	LOC_Os10g34180.1	10
1698	LOC_Os01g10320.1	1
1698	LOC_Os06g50510.1	6
1698	LOC_Os06g50560.1	6
1699	LOC_Os11g01074.1	11
1699	LOC_Os12g01060.1	12
1699	LOC_Os12g07680.1	12
1700	LOC_Os02g02130.1	2
1700	LOC_Os02g56990.1	2
1700	LOC_Os08g03450.1	8
1701	LOC_Os02g13400.1	2
1701	LOC_Os06g21470.1	6
1701	LOC_Os06g36360.1	6
1702	LOC_Os08g29760.1	8
1702	LOC_Os08g30020.1	8
1702	LOC_Os09g17600.1	9
1703	LOC_Os01g08290.1	1
1703	LOC_Os01g32280.1	1
1703	LOC_Os01g52864.1	1
1704	LOC_Os07g42160.1	7
1704	LOC_Os08g35620.1	8
1704	LOC_Os09g27050.1	9
1705	LOC_Os01g54370.1	1
1705	LOC_Os01g59340.1	1
1705	LOC_Os04g58390.1	4
1706	LOC_Os01g72820.1	1
1706	LOC_Os04g41550.1	4
1706	LOC_Os05g05300.1	5
1707	LOC_Os01g12260.1	1
1707	LOC_Os01g46980.1	1
1707	LOC_Os05g40230.1	5
1708	LOC_Os01g68540.1	1
1708	LOC_Os02g48730.1	2

1708	LOC_Os06g21340.1	6
1709	LOC_Os01g55880.1	1
1709	LOC_Os08g07540.1	8
1709	LOC_Os08g40450.1	8
1710	LOC_Os06g06100.1	6
1710	LOC_Os08g44210.1	8
1710	LOC_Os09g38759.1	9
1711	LOC_Os02g40280.1	2
1711	LOC_Os03g14790.1	3
1711	LOC_Os07g16224.1	7
1712	LOC_Os08g30870.1	8
1712	LOC_Os08g30900.1	8
1712	LOC_Os08g30850.1	8
1713	LOC_Os01g54040.1	1
1713	LOC_Os03g29540.1	3
1713	LOC_Os07g32560.1	7
1714	LOC_Os01g69990.1	1
1714	LOC_Os05g34790.1	5
1714	LOC_Os07g04530.1	7
1715	LOC_Os02g39910.1	2
1715	LOC_Os04g42310.1	4
1715	LOC_Os07g23720.1	7
1716	LOC_Os02g52480.1	2
1716	LOC_Os06g11050.1	6
1716	LOC_Os11g40030.1	11
1717	LOC_Os03g59220.1	3
1717	LOC_Os07g10500.1	7
1717	LOC_Os08g14860.1	8
1718	LOC_Os03g27290.1	3
1718	LOC_Os04g42060.1	4
1718	LOC_Os07g42910.1	7
1719	LOC_Os03g10570.1	3
1719	LOC_Os04g40940.1	4
1719	LOC_Os09g32930.1	9
1720	LOC_Os01g73540.1	1
1720	LOC_Os03g08080.1	3
1720	LOC_Os07g08770.1	7
1721	LOC_Os04g57610.1	4
1721	LOC_Os06g46410.1	6
1721	LOC_Os12g41950.1	12
1722	LOC_Os02g58560.1	2
1722	LOC_Os06g08380.1	6
1722	LOC_Os06g51270.1	6
1723	LOC_Os01g55040.1	1
1723	LOC_Os02g14900.1	2
1723	LOC_Os03g03610.1	3
1724	LOC_Os01g36630.1	1
1724	LOC_Os02g46930.1	2
1724	LOC_Os09g31050.1	9
1725	LOC_Os02g01940.1	2
1725	LOC_Os03g22540.1	3
1725	LOC_Os09g22540.1	9

1726	LOC_Os01g20160.1	1
1726	LOC_Os02g07830.1	2
1726	LOC_Os04g51830.1	4
1727	LOC_Os01g35170.1	1
1727	LOC_Os06g12990.1	6
1727	LOC_Os10g30550.1	10
1728	LOC_Os02g56910.1	2
1728	LOC_Os04g52500.1	4
1728	LOC_Os09g27210.1	9
1729	LOC_Os01g71800.1	1
1729	LOC_Os03g52010.1	3
1729	LOC_Os03g13030.1	3
1730	LOC_Os02g04040.1	2
1730	LOC_Os02g04060.1	2
1730	LOC_Os05g41750.1	5
1731	LOC_Os02g55630.1	2
1731	LOC_Os04g51100.1	4
1731	LOC_Os07g40790.1	7
1732	LOC_Os01g01960.1	1
1732	LOC_Os05g01020.1	5
1732	LOC_Os08g25200.1	8
1733	LOC_Os01g19740.1	1
1733	LOC_Os03g19380.1	3
1733	LOC_Os08g40240.1	8
1734	LOC_Os03g62790.1	3
1734	LOC_Os08g27720.1	8
1734	LOC_Os09g31120.1	9
1735	LOC_Os03g10750.1	3
1735	LOC_Os06g49200.1	6
1735	LOC_Os10g16440.1	10
1736	LOC_Os04g03000.1	4
1736	LOC_Os07g34230.1	7
1736	LOC_Os10g14230.1	10
1737	LOC_Os02g08340.1	2
1737	LOC_Os02g55620.1	2
1737	LOC_Os05g50640.1	5
1738	LOC_Os02g14250.1	2
1738	LOC_Os02g32170.1	2
1738	LOC_Os10g30680.1	10
1739	LOC_Os03g24960.1	3
1739	LOC_Os03g24990.1	3
1739	LOC_Os04g49394.1	4
1740	LOC_Os02g20310.1	2
1740	LOC_Os08g42189.1	8
1740	LOC_Os08g42268.1	8
1741	LOC_Os07g40460.1	7
1741	LOC_Os10g34670.1	10
1741	LOC_Os12g37590.1	12
1742	LOC_Os01g46400.1	1
1742	LOC_Os02g49660.1	2
1742	LOC_Os06g16250.1	6
1743	LOC_Os01g02890.1	1

1743	LOC_Os01g49020.1	1
1743	LOC_Os05g48060.1	5
1744	LOC_Os03g19436.1	3
1744	LOC_Os03g19420.1	3
1744	LOC_Os07g48980.1	7
1745	LOC_Os01g37000.1	1
1745	LOC_Os01g74160.1	1
1745	LOC_Os07g38590.1	7
1746	LOC_Os02g54960.1	2
1746	LOC_Os02g55230.1	2
1746	LOC_Os05g27500.1	5
1747	LOC_Os01g28600.1	1
1747	LOC_Os06g14450.1	6
1747	LOC_Os08g13570.1	8
1748	LOC_Os03g04040.1	3
1748	LOC_Os06g50980.1	6
1748	LOC_Os10g39660.1	10
1749	LOC_Os01g09670.1	1
1749	LOC_Os05g10740.1	5
1749	LOC_Os06g36740.1	6
1750	LOC_Os03g20560.1	3
1750	LOC_Os08g33810.1	8
1750	LOC_Os12g40450.1	12
1751	LOC_Os01g02000.1	1
1751	LOC_Os02g56510.1	2
1751	LOC_Os06g29790.1	6
1752	LOC_Os02g45520.1	2
1752	LOC_Os04g48390.1	4
1752	LOC_Os06g03860.1	6
1753	LOC_Os02g10780.1	2
1753	LOC_Os06g40120.1	6
1753	LOC_Os10g25310.1	10
1754	LOC_Os01g61080.1	1
1754	LOC_Os05g39720.1	5
1754	LOC_Os05g27730.1	5
1755	LOC_Os02g56200.1	2
1755	LOC_Os02g57160.1	2
1755	LOC_Os11g11000.1	11
1756	LOC_Os04g54790.1	4
1756	LOC_Os07g27480.1	7
1756	LOC_Os09g07660.1	9
1757	LOC_Os02g36570.1	2
1757	LOC_Os05g25840.1	5
1757	LOC_Os06g48770.1	6
1758	LOC_Os01g09300.1	1
1758	LOC_Os09g07020.1	9
1758	LOC_Os10g37880.1	10
1759	LOC_Os05g50090.1	5
1759	LOC_Os08g32170.1	8
1759	LOC_Os08g32160.1	8
1760	LOC_Os02g04480.1	2
1760	LOC_Os05g51480.1	5

1760	LOC_Os07g10390.1	7
1761	LOC_Os02g39300.1	2
1761	LOC_Os03g62640.1	3
1761	LOC_Os07g27300.1	7
1762	LOC_Os11g42350.1	11
1762	LOC_Os12g16200.1	12
1762	LOC_Os12g34380.1	12
1763	LOC_Os03g40270.1	3
1763	LOC_Os04g56520.1	4
1763	LOC_Os07g41360.1	7
1764	LOC_Os01g01640.1	1
1764	LOC_Os06g49460.1	6
1764	LOC_Os08g21760.1	8
1765	LOC_Os04g20420.1	4
1765	LOC_Os08g01890.1	8
1765	LOC_Os12g43880.1	12
1766	LOC_Os08g44480.1	8
1766	LOC_Os09g39540.1	9
1766	LOC_Os11g05562.1	11
1767	LOC_Os09g17070.1	9
1767	LOC_Os09g21660.1	9
1767	LOC_Os09g21670.1	9
1768	LOC_Os06g35080.1	6
1768	LOC_Os10g37450.1	10
1768	LOC_Os10g37600.1	10
1769	LOC_Os01g07250.1	1
1769	LOC_Os05g26954.1	5
1769	LOC_Os11g02150.1	11
1770	LOC_Os05g01140.1	5
1770	LOC_Os06g21020.1	6
1770	LOC_Os11g15310.1	11
1771	LOC_Os01g73160.1	1
1771	LOC_Os02g34460.1	2
1771	LOC_Os04g35090.1	4
1772	LOC_Os03g51330.1	3
1772	LOC_Os11g03110.1	11
1772	LOC_Os12g02870.1	12
1773	LOC_Os04g49110.1	4
1773	LOC_Os05g49930.1	5
1773	LOC_Os07g40020.1	7
1774	LOC_Os06g12690.1	6
1774	LOC_Os07g43720.1	7
1774	LOC_Os08g34070.1	8
1775	LOC_Os04g26994.1	4
1775	LOC_Os08g17810.1	8
1775	LOC_Os09g09310.1	9
1776	LOC_Os01g47770.1	1
1776	LOC_Os02g51000.1	2
1776	LOC_Os06g12530.1	6
1777	LOC_Os01g14520.1	1
1777	LOC_Os04g48530.1	4
1777	LOC_Os05g18670.1	5

1778	LOC_Os01g28840.1	1
1778	LOC_Os05g13320.1	5
1778	LOC_Os07g08350.1	7
1779	LOC_Os03g59360.1	3
1779	LOC_Os07g10780.1	7
1779	LOC_Os07g38170.1	7
1780	LOC_Os04g08170.1	4
1780	LOC_Os05g39890.1	5
1780	LOC_Os08g19410.1	8
1781	LOC_Os03g03370.1	3
1781	LOC_Os04g48880.1	4
1781	LOC_Os10g38940.1	10
1782	LOC_Os04g49580.1	4
1782	LOC_Os06g30320.1	6
1782	LOC_Os09g02810.1	9
1783	LOC_Os02g45180.1	2
1783	LOC_Os04g47970.1	4
1783	LOC_Os07g26940.1	7
1784	LOC_Os01g24950.1	1
1784	LOC_Os03g06760.1	3
1784	LOC_Os10g27990.1	10
1785	LOC_Os03g07580.1	3
1785	LOC_Os12g06870.1	12
1785	LOC_Os12g06890.1	12
1786	LOC_Os01g48920.1	1
1786	LOC_Os03g15290.1	3
1786	LOC_Os03g44200.1	3
1787	LOC_Os01g07800.1	1
1787	LOC_Os04g57310.1	4
1787	LOC_Os06g13010.1	6
1788	LOC_Os01g57220.1	1
1788	LOC_Os03g38600.1	3
1788	LOC_Os05g42330.1	5
1789	LOC_Os02g47010.1	2
1789	LOC_Os04g50890.1	4
1789	LOC_Os07g37740.1	7
1790	LOC_Os04g51940.1	4
1790	LOC_Os07g07490.1	7
1790	LOC_Os08g12760.1	8
1791	LOC_Os01g48790.1	1
1791	LOC_Os03g06240.1	3
1791	LOC_Os03g53670.1	3
1792	LOC_Os01g16170.1	1
1792	LOC_Os07g29610.1	7
1792	LOC_Os12g18110.1	12
1793	LOC_Os02g02530.1	2
1793	LOC_Os05g36070.1	5
1793	LOC_Os10g25360.1	10
1794	LOC_Os01g25440.1	1
1794	LOC_Os01g70580.1	1
1794	LOC_Os05g30454.1	5
1795	LOC_Os10g34910.1	10

1795	LOC_Os10g34920.1	10
1795	LOC_Os10g34930.1	10
1796	LOC_Os01g40340.1	1
1796	LOC_Os04g52940.1	4
1796	LOC_Os05g51490.1	5
1797	LOC_Os01g40600.1	1
1797	LOC_Os01g40610.1	1
1797	LOC_Os02g21880.1	2
1798	LOC_Os03g09880.1	3
1798	LOC_Os08g24790.1	8
1798	LOC_Os08g41290.1	8
1799	LOC_Os05g05950.1	5
1799	LOC_Os10g40110.1	10
1799	LOC_Os12g09570.1	12
1800	LOC_Os01g24240.1	1
1800	LOC_Os01g23370.1	1
1800	LOC_Os01g23440.1	1
1801	LOC_Os04g05400.1	4
1801	LOC_Os04g06510.1	4
1801	LOC_Os10g11310.1	10
1802	LOC_Os01g58500.1	1
1802	LOC_Os02g14700.1	2
1802	LOC_Os04g05700.1	4
1803	LOC_Os11g40260.1	11
1803	LOC_Os11g40420.1	11
1803	LOC_Os11g40390.1	11
1804	LOC_Os05g02460.1	5
1804	LOC_Os06g06710.1	6
1804	LOC_Os08g06790.1	8
1805	LOC_Os03g16334.1	3
1805	LOC_Os08g04300.1	8
1805	LOC_Os10g37260.1	10
1806	LOC_Os01g01720.1	1
1806	LOC_Os05g01090.1	5
1806	LOC_Os05g50220.1	5
1807	LOC_Os01g41610.1	1
1807	LOC_Os01g52200.1	1
1807	LOC_Os05g45740.1	5
1808	LOC_Os02g38140.1	2
1808	LOC_Os02g38160.1	2
1808	LOC_Os04g40150.1	4
1809	LOC_Os03g20340.1	3
1809	LOC_Os05g41220.1	5
1809	LOC_Os07g48790.1	7
1810	LOC_Os08g40420.1	8
1810	LOC_Os09g32010.1	9
1810	LOC_Os10g41040.1	10
1811	LOC_Os03g07120.1	3
1811	LOC_Os05g16300.1	5
1811	LOC_Os10g27110.1	10
1812	LOC_Os02g52160.1	2
1812	LOC_Os06g03760.1	6

1812	LOC_Os06g11520.1	6
1813	LOC_Os05g32110.1	5
1813	LOC_Os07g41310.1	7
1813	LOC_Os10g35460.1	10
1814	LOC_Os02g34560.1	2
1814	LOC_Os04g35280.1	4
1814	LOC_Os11g07440.1	11
1815	LOC_Os01g14540.1	1
1815	LOC_Os05g18860.1	5
1815	LOC_Os05g18940.1	5
1816	LOC_Os05g34240.1	5
1816	LOC_Os06g33420.1	6
1816	LOC_Os10g40240.1	10
1817	LOC_Os01g12280.1	1
1817	LOC_Os03g60800.1	3
1817	LOC_Os09g32380.1	9
1818	LOC_Os05g24790.1	5
1818	LOC_Os06g49050.1	6
1818	LOC_Os10g03220.1	10
1819	LOC_Os02g34650.1	2
1819	LOC_Os05g35530.1	5
1819	LOC_Os06g50220.1	6
1820	LOC_Os03g52320.1	3
1820	LOC_Os11g40100.1	11
1820	LOC_Os12g31350.1	12
1821	LOC_Os03g05440.1	3
1821	LOC_Os03g10240.1	3
1821	LOC_Os10g21540.1	10
1822	LOC_Os06g06080.1	6
1822	LOC_Os11g06690.1	11
1822	LOC_Os12g06800.1	12
1823	LOC_Os01g16180.1	1
1823	LOC_Os02g45110.1	2
1823	LOC_Os03g05420.1	3
1824	LOC_Os01g27880.1	1
1824	LOC_Os02g05030.1	2
1824	LOC_Os05g05270.1	5
1825	LOC_Os02g04160.1	2
1825	LOC_Os06g06270.1	6
1825	LOC_Os07g43730.1	7
1826	LOC_Os01g42050.1	1
1826	LOC_Os02g57430.1	2
1826	LOC_Os06g50870.1	6
1827	LOC_Os02g55640.1	2
1827	LOC_Os03g38720.1	3
1827	LOC_Os09g14590.1	9
1828	LOC_Os01g55170.1	1
1828	LOC_Os07g01260.1	7
1828	LOC_Os08g42980.1	8
1829	LOC_Os03g05520.1	3
1829	LOC_Os04g10240.1	4
1829	LOC_Os10g30860.1	10

1830	LOC_Os02g21650.1	2
1830	LOC_Os03g63110.1	3
1830	LOC_Os05g19040.1	5
1831	LOC_Os01g51430.1	1
1831	LOC_Os03g58520.1	3
1831	LOC_Os05g46240.1	5
1832	LOC_Os04g25520.1	4
1832	LOC_Os08g31210.1	8
1832	LOC_Os08g34690.1	8
1833	LOC_Os03g22040.1	3
1833	LOC_Os05g39840.1	5
1833	LOC_Os07g48080.1	7
1834	LOC_Os01g37770.1	1
1834	LOC_Os04g50730.1	4
1834	LOC_Os11g35870.1	11
1835	LOC_Os02g35080.1	2
1835	LOC_Os04g35840.1	4
1835	LOC_Os04g35860.1	4
1836	LOC_Os01g39310.1	1
1836	LOC_Os11g37260.1	11
1836	LOC_Os12g41170.1	12
1837	LOC_Os03g55130.1	3
1837	LOC_Os10g40580.1	10
1837	LOC_Os12g38870.1	12
1838	LOC_Os04g04390.1	4
1838	LOC_Os10g12174.1	10
1838	LOC_Os12g26850.1	12
1839	LOC_Os10g35880.1	10
1839	LOC_Os11g28660.1	11
1839	LOC_Os12g35400.1	12
1840	LOC_Os04g23360.1	4
1840	LOC_Os05g04260.1	5
1840	LOC_Os05g19050.1	5
1841	LOC_Os04g28820.1	4
1841	LOC_Os10g10730.1	10
1841	LOC_Os10g11620.1	10
1842	LOC_Os04g01520.1	4
1842	LOC_Os12g37920.1	12
1842	LOC_Os12g37939.1	12
1843	LOC_Os08g44020.1	8
1843	LOC_Os08g44040.1	8
1843	LOC_Os12g03790.1	12
1844	LOC_Os07g14580.1	7
1844	LOC_Os11g05650.1	11
1844	LOC_Os12g06020.1	12
1845	LOC_Os03g04930.1	3
1845	LOC_Os03g51150.1	3
1845	LOC_Os07g38840.1	7
1846	LOC_Os02g36870.1	2
1846	LOC_Os08g33680.1	8
1846	LOC_Os10g26700.1	10
1847	LOC_Os01g53070.1	1

1847	LOC_Os05g45340.1	5
1847	LOC_Os08g02670.1	8
1848	LOC_Os06g09820.1	6
1848	LOC_Os06g09830.1	6
1848	LOC_Os08g35480.1	8
1849	LOC_Os03g28940.1	3
1849	LOC_Os07g42370.1	7
1849	LOC_Os10g25290.1	10
1850	LOC_Os02g38220.1	2
1850	LOC_Os02g24330.1	2
1850	LOC_Os04g40400.1	4
1851	LOC_Os06g06430.1	6
1851	LOC_Os11g02630.1	11
1851	LOC_Os12g02550.1	12
1852	LOC_Os01g40980.1	1
1852	LOC_Os05g05260.1	5
1852	LOC_Os09g37920.1	9
1853	LOC_Os01g73730.1	1
1853	LOC_Os04g33520.1	4
1853	LOC_Os07g23600.1	7
1854	LOC_Os05g18960.1	5
1854	LOC_Os10g31360.1	10
1854	LOC_Os12g08530.1	12
1855	LOC_Os01g52830.1	1
1855	LOC_Os01g54520.1	1
1855	LOC_Os05g49440.1	5
1856	LOC_Os03g11400.1	3
1856	LOC_Os07g32390.1	7
1856	LOC_Os12g38790.1	12
1857	LOC_Os01g41660.1	1
1857	LOC_Os01g52230.1	1
1857	LOC_Os02g13290.1	2
1858	LOC_Os01g52480.1	1
1858	LOC_Os03g13840.1	3
1858	LOC_Os06g50330.1	6
1859	LOC_Os03g18530.1	3
1859	LOC_Os11g06730.1	11
1859	LOC_Os12g06970.1	12
1860	LOC_Os02g55910.1	2
1860	LOC_Os08g20420.1	8
1860	LOC_Os09g25580.1	9
1861	LOC_Os11g03780.1	11
1861	LOC_Os12g03480.1	12
1861	LOC_Os12g03530.1	12
1862	LOC_Os01g14020.1	1
1862	LOC_Os01g54950.1	1
1862	LOC_Os02g20844.1	2
1863	LOC_Os02g50320.1	2
1863	LOC_Os06g14080.1	6
1863	LOC_Os12g44340.1	12
1864	LOC_Os03g18770.1	3
1864	LOC_Os05g27580.1	5

1864	LOC_Os05g27590.1	5
1865	LOC_Os12g31850.1	12
1865	LOC_Os12g31890.1	12
1865	LOC_Os12g31860.1	12
1866	LOC_Os03g48920.1	3
1866	LOC_Os08g02560.1	8
1866	LOC_Os08g02570.1	8
1867	LOC_Os01g57440.1	1
1867	LOC_Os01g65890.1	1
1867	LOC_Os08g01630.1	8
1868	LOC_Os01g65860.1	1
1868	LOC_Os08g36410.1	8
1868	LOC_Os11g14000.1	11
1869	LOC_Os03g19990.1	3
1869	LOC_Os03g28980.1	3
1869	LOC_Os07g44410.1	7
1870	LOC_Os01g42060.1	1
1870	LOC_Os07g01460.1	7
1870	LOC_Os08g24170.1	8
1871	LOC_Os03g07970.1	3
1871	LOC_Os03g08160.1	3
1871	LOC_Os09g38860.1	9
1872	LOC_Os05g16150.1	5
1872	LOC_Os05g39160.1	5
1872	LOC_Os05g39170.1	5
1873	LOC_Os11g07350.1	11
1873	LOC_Os11g07420.1	11
1873	LOC_Os11g36170.1	11
1874	LOC_Os01g44040.1	1
1874	LOC_Os03g19970.1	3
1874	LOC_Os05g50510.1	5
1875	LOC_Os03g38540.1	3
1875	LOC_Os06g02610.1	6
1875	LOC_Os06g03740.1	6
1876	LOC_Os05g33940.1	5
1876	LOC_Os11g04360.1	11
1876	LOC_Os12g04150.1	12
1877	LOC_Os01g03870.1	1
1877	LOC_Os08g40820.1	8
1877	LOC_Os09g24700.1	9
1878	LOC_Os03g11550.1	3
1878	LOC_Os03g30570.1	3
1878	LOC_Os07g41160.1	7
1879	LOC_Os03g58070.1	3
1879	LOC_Os07g07680.1	7
1879	LOC_Os09g07360.1	9
1880	LOC_Os02g26170.1	2
1880	LOC_Os02g52770.1	2
1880	LOC_Os04g21340.1	4
1881	LOC_Os02g10190.1	2
1881	LOC_Os06g41020.1	6
1881	LOC_Os06g41030.1	6

1882	LOC_Os01g42080.1	1
1882	LOC_Os03g57160.1	3
1882	LOC_Os07g05610.1	7
1883	LOC_Os03g20520.1	3
1883	LOC_Os04g38340.1	4
1883	LOC_Os05g41120.1	5
1884	LOC_Os04g49920.1	4
1884	LOC_Os04g49930.1	4
1884	LOC_Os05g02690.1	5
1885	LOC_Os02g10130.1	2
1885	LOC_Os02g35110.1	2
1885	LOC_Os06g41110.1	6
1886	LOC_Os04g28600.1	4
1886	LOC_Os04g28680.1	4
1886	LOC_Os07g23340.1	7
1887	LOC_Os06g04350.1	6
1887	LOC_Os06g04360.1	6
1887	LOC_Os10g35210.1	10
1888	LOC_Os06g04330.1	6
1888	LOC_Os06g04340.1	6
1888	LOC_Os06g04470.1	6
1889	LOC_Os03g34102.1	3
1889	LOC_Os07g20750.1	7
1889	LOC_Os10g11530.1	10
1890	LOC_Os05g08180.1	5
1890	LOC_Os05g15730.1	5
1890	LOC_Os08g28260.1	8
1891	LOC_Os04g07810.1	4
1891	LOC_Os08g14520.1	8
1891	LOC_Os12g33490.1	12
1892	LOC_Os01g34960.1	1
1892	LOC_Os02g23850.1	2
1892	LOC_Os10g03060.1	10
1893	LOC_Os03g44100.1	3
1893	LOC_Os12g42100.1	12
1893	LOC_Os12g42110.1	12
1894	LOC_Os04g32810.1	4
1894	LOC_Os04g32820.1	4
1894	LOC_Os10g14050.1	10
1895	LOC_Os06g22380.1	6
1895	LOC_Os07g44770.1	7
1895	LOC_Os10g11780.1	10
1896	LOC_Os07g09250.1	7
1896	LOC_Os10g10740.1	10
1896	LOC_Os10g11610.1	10
1897	LOC_Os02g47370.1	2
1897	LOC_Os04g51320.1	4
1897	LOC_Os05g03740.1	5
1898	LOC_Os02g40210.1	2
1898	LOC_Os05g04130.1	5
1898	LOC_Os10g35320.1	10
1899	LOC_Os03g07600.1	3

1899	LOC_Os07g39630.1	7
1899	LOC_Os07g45280.1	7
1900	LOC_Os03g18280.1	3
1900	LOC_Os07g28070.1	7
1900	LOC_Os09g17860.1	9
1901	LOC_Os04g33910.1	4
1901	LOC_Os05g16090.1	5
1901	LOC_Os11g19670.1	11
1902	LOC_Os03g45310.1	3
1902	LOC_Os04g07630.1	4
1902	LOC_Os12g14850.1	12
1903	LOC_Os01g04880.1	1
1903	LOC_Os11g02090.1	11
1903	LOC_Os12g02050.1	12
1904	LOC_Os01g33580.1	1
1904	LOC_Os08g20040.1	8
1904	LOC_Os09g11150.1	9
1905	LOC_Os05g18250.1	5
1905	LOC_Os05g28060.1	5
1905	LOC_Os07g25070.1	7
1906	LOC_Os05g18419.1	5
1906	LOC_Os05g18437.1	5
1906	LOC_Os05g18455.1	5
1907	LOC_Os02g58420.1	2
1907	LOC_Os03g31870.1	3
1907	LOC_Os09g32790.1	9
1908	LOC_Os03g08980.1	3
1908	LOC_Os04g37790.1	4
1908	LOC_Os08g37500.1	8
1909	LOC_Os01g03080.1	1
1909	LOC_Os01g49210.1	1
1909	LOC_Os07g03120.1	7
1910	LOC_Os04g12900.1	4
1910	LOC_Os06g18790.1	6
1910	LOC_Os10g18480.1	10
1911	LOC_Os05g37870.1	5
1911	LOC_Os08g19770.1	8
1911	LOC_Os08g25680.1	8
1912	LOC_Os01g35960.1	1
1912	LOC_Os02g21540.1	2
1912	LOC_Os03g56740.1	3
1913	LOC_Os07g41060.1	7
1913	LOC_Os09g09270.1	9
1913	LOC_Os09g31522.1	9
1914	LOC_Os03g27920.1	3
1914	LOC_Os06g25330.1	6
1914	LOC_Os09g18580.1	9
1915	LOC_Os03g22290.1	3
1915	LOC_Os03g25570.1	3
1915	LOC_Os11g19600.1	11
1916	LOC_Os03g43550.1	3
1916	LOC_Os05g32470.1	5

1916	LOC_Os08g30550.1	8
1917	LOC_Os03g45690.1	3
1917	LOC_Os06g19830.1	6
1917	LOC_Os09g03910.1	9
1918	LOC_Os07g30280.1	7
1918	LOC_Os11g35430.1	11
1918	LOC_Os12g07500.1	12
1919	LOC_Os02g37710.1	2
1919	LOC_Os02g37760.1	2
1919	LOC_Os02g37770.1	2
1920	LOC_Os02g32270.1	2
1920	LOC_Os02g32310.1	2
1920	LOC_Os02g42800.1	2
1921	LOC_Os11g01400.1	11
1921	LOC_Os11g09820.1	11
1921	LOC_Os12g01410.1	12
1922	LOC_Os01g66230.1	1
1922	LOC_Os10g34602.1	10
1922	LOC_Os10g34614.1	10
1923	LOC_Os09g09010.1	9
1923	LOC_Os09g09030.1	9
1923	LOC_Os09g12450.1	9
1924	LOC_Os02g57620.1	2
1924	LOC_Os03g01700.1	3
1924	LOC_Os10g31040.1	10
1925	LOC_Os01g45260.1	1
1925	LOC_Os06g15490.1	6
1925	LOC_Os11g38330.1	11
1926	LOC_Os02g29170.1	2
1926	LOC_Os10g22770.1	10
1926	LOC_Os12g18850.1	12
1927	LOC_Os09g08520.1	9
1927	LOC_Os09g08570.1	9
1927	LOC_Os09g08580.1	9
1928	LOC_Os02g47560.1	2
1928	LOC_Os03g38990.1	3
1928	LOC_Os09g04440.1	9
1929	LOC_Os02g41940.1	2
1929	LOC_Os05g27760.1	5
1929	LOC_Os06g46960.1	6
1930	LOC_Os01g53730.1	1
1930	LOC_Os02g45930.1	2
1930	LOC_Os05g44900.1	5
1931	LOC_Os02g02080.1	2
1931	LOC_Os05g02230.1	5
1931	LOC_Os08g26210.1	8
1932	LOC_Os11g31420.1	11
1932	LOC_Os11g31450.1	11
1932	LOC_Os11g31800.1	11
1933	LOC_Os08g43850.1	8
1933	LOC_Os08g43780.1	8
1933	LOC_Os08g43950.1	8

1934	LOC_Os01g62780.1	1
1934	LOC_Os08g44560.1	8
1934	LOC_Os09g39700.1	9
1935	LOC_Os03g31290.1	3
1935	LOC_Os04g32270.1	4
1935	LOC_Os06g12020.1	6
1936	LOC_Os11g42950.1	11
1936	LOC_Os12g35230.1	12
1936	LOC_Os12g35260.1	12
1937	LOC_Os02g23960.1	2
1937	LOC_Os04g28450.1	4
1937	LOC_Os06g33790.1	6
1938	LOC_Os11g40480.1	11
1938	LOC_Os11g41250.1	11
1938	LOC_Os12g34820.1	12
1939	LOC_Os09g17270.1	9
1939	LOC_Os09g22370.1	9
1939	LOC_Os12g15370.1	12
1940	LOC_Os05g37670.1	5
1940	LOC_Os06g24000.1	6
1940	LOC_Os12g18090.1	12
1941	LOC_Os04g30000.1	4
1941	LOC_Os07g17200.1	7
1941	LOC_Os09g04910.1	9
1942	LOC_Os03g28170.1	3
1942	LOC_Os04g22900.1	4
1942	LOC_Os12g05020.1	12
1943	LOC_Os02g26280.1	2
1943	LOC_Os05g12700.1	5
1943	LOC_Os12g26490.1	12
1944	LOC_Os01g38200.1	1
1944	LOC_Os04g26820.1	4
1944	LOC_Os10g20740.1	10
1945	LOC_Os01g16940.1	1
1945	LOC_Os04g34180.1	4
1945	LOC_Os08g03400.1	8
1946	LOC_Os01g03280.1	1
1946	LOC_Os02g36290.1	2
1946	LOC_Os12g06940.1	12
1947	LOC_Os07g03328.1	7
1947	LOC_Os07g03508.1	7
1947	LOC_Os07g03418.1	7
1948	LOC_Os01g57700.1	1
1948	LOC_Os01g57710.1	1
1948	LOC_Os03g51650.1	3
1949	LOC_Os04g42480.1	4
1949	LOC_Os09g17890.1	9
1949	LOC_Os09g22300.1	9
1950	LOC_Os01g48850.1	1
1950	LOC_Os01g67030.1	1
1950	LOC_Os05g48270.1	5
1951	LOC_Os11g44230.1	11

1951	LOC_Os11g44300.1	11
1951	LOC_Os11g44850.1	11
1952	LOC_Os11g44380.1	11
1952	LOC_Os11g43990.1	11
1952	LOC_Os12g14520.1	12
1953	LOC_Os08g12160.1	8
1953	LOC_Os08g12600.1	8
1953	LOC_Os08g12520.1	8
1954	LOC_Os01g40220.1	1
1954	LOC_Os06g22880.1	6
1954	LOC_Os10g37290.1	10
1955	LOC_Os01g40360.1	1
1955	LOC_Os04g38670.1	4
1955	LOC_Os05g30150.1	5
1956	LOC_Os04g19510.1	4
1956	LOC_Os09g13120.1	9
1956	LOC_Os09g27020.1	9
1957	LOC_Os03g17460.1	3
1957	LOC_Os03g17470.1	3
1957	LOC_Os03g17480.1	3
1958	LOC_Os01g35610.1	1
1958	LOC_Os05g14210.1	5
1958	LOC_Os09g17500.1	9
1959	LOC_Os03g44870.1	3
1959	LOC_Os09g02420.1	9
1959	LOC_Os11g31140.1	11
1960	LOC_Os01g04000.1	1
1960	LOC_Os01g39060.1	1
1960	LOC_Os03g42180.1	3
1961	LOC_Os02g02270.1	2
1961	LOC_Os02g02240.1	2
1961	LOC_Os10g32020.1	10
1962	LOC_Os04g04140.1	4
1962	LOC_Os10g08510.1	10
1962	LOC_Os11g43170.1	11
1963	LOC_Os01g39280.1	1
1963	LOC_Os10g08960.1	10
1963	LOC_Os10g11520.1	10
1964	LOC_Os01g32610.1	1
1964	LOC_Os03g19770.1	3
1964	LOC_Os04g49140.1	4
1965	LOC_Os01g44100.1	1
1965	LOC_Os04g37890.1	4
1965	LOC_Os06g41680.1	6
1966	LOC_Os01g13840.1	1
1966	LOC_Os01g52020.1	1
1966	LOC_Os12g44280.1	12
1967	LOC_Os06g32750.1	6
1967	LOC_Os08g23900.1	8
1967	LOC_Os08g26080.1	8
1968	LOC_Os01g53970.1	1
1968	LOC_Os02g43540.1	2

1968	LOC_Os12g13980.1	12
1969	LOC_Os10g04910.1	10
1969	LOC_Os10g04930.1	10
1969	LOC_Os10g04940.1	10
1970	LOC_Os03g62560.1	3
1970	LOC_Os08g29220.1	8
1970	LOC_Os09g35640.1	9
1971	LOC_Os06g41800.1	6
1971	LOC_Os09g31518.1	9
1971	LOC_Os09g31506.1	9
1972	LOC_Os05g06530.1	5
1972	LOC_Os08g05340.1	8
1972	LOC_Os08g05030.1	8
1973	LOC_Os01g23710.1	1
1973	LOC_Os10g25630.1	10
1973	LOC_Os10g26250.1	10
1974	LOC_Os01g66300.1	1
1974	LOC_Os02g49080.1	2
1974	LOC_Os05g34670.1	5
1975	LOC_Os08g09130.1	8
1975	LOC_Os10g35080.1	10
1975	LOC_Os11g27990.1	11
1976	LOC_Os03g49680.1	3
1976	LOC_Os05g01830.1	5
1976	LOC_Os08g03790.1	8
1977	LOC_Os01g15290.1	1
1977	LOC_Os03g48840.1	3
1977	LOC_Os09g37740.1	9
1978	LOC_Os01g54770.1	1
1978	LOC_Os05g02700.1	5
1978	LOC_Os05g36980.1	5
1979	LOC_Os01g57840.1	1
1979	LOC_Os03g19800.1	3
1979	LOC_Os10g15390.1	10
1980	LOC_Os01g68670.1	1
1980	LOC_Os03g11160.1	3
1980	LOC_Os04g28250.1	4
1981	LOC_Os01g08140.1	1
1981	LOC_Os01g71558.1	1
1981	LOC_Os01g71608.1	1
1982	LOC_Os02g04690.1	2
1982	LOC_Os05g14800.1	5
1982	LOC_Os06g28820.1	6
1983	LOC_Os08g17760.1	8
1983	LOC_Os08g35650.1	8
1983	LOC_Os08g35680.1	8
1984	LOC_Os02g03640.1	2
1984	LOC_Os07g11150.1	7
1984	LOC_Os08g28010.1	8
1985	LOC_Os06g40520.1	6
1985	LOC_Os11g15280.1	11
1985	LOC_Os11g41720.1	11

1986	LOC_Os11g44420.1	11
1986	LOC_Os11g44870.1	11
1986	LOC_Os12g31540.1	12
1987	LOC_Os05g37640.1	5
1987	LOC_Os12g23900.1	12
1987	LOC_Os12g43140.1	12
1988	LOC_Os01g34990.1	1
1988	LOC_Os02g06710.1	2
1988	LOC_Os02g28970.1	2
1989	LOC_Os02g42640.1	2
1989	LOC_Os08g41740.1	8
1989	LOC_Os10g35630.1	10
1990	LOC_Os01g15250.1	1
1990	LOC_Os01g40020.1	1
1990	LOC_Os10g04550.1	10
1991	LOC_Os08g41550.1	8
1991	LOC_Os08g41560.1	8
1991	LOC_Os08g41580.1	8
1992	LOC_Os03g05410.1	3
1992	LOC_Os11g05710.1	11
1992	LOC_Os12g43290.1	12
1993	LOC_Os07g40850.1	7
1993	LOC_Os07g40860.1	7
1993	LOC_Os09g36490.1	9
1994	LOC_Os03g41940.1	3
1994	LOC_Os04g17200.1	4
1994	LOC_Os08g35980.1	8
1995	LOC_Os01g15680.1	1
1995	LOC_Os07g14120.1	7
1995	LOC_Os08g06220.1	8
1996	LOC_Os06g14970.1	6
1996	LOC_Os08g15740.1	8
1996	LOC_Os08g16210.1	8
1997	LOC_Os06g24430.1	6
1997	LOC_Os07g15960.1	7
1997	LOC_Os09g09040.1	9
1998	LOC_Os02g01530.1	2
1998	LOC_Os03g11874.1	3
1998	LOC_Os11g32410.1	11
1999	LOC_Os04g07280.1	4
1999	LOC_Os04g07600.1	4
1999	LOC_Os04g07890.1	4
2000	LOC_Os01g22730.1	1
2000	LOC_Os05g32000.1	5
2000	LOC_Os09g09540.1	9
2001	LOC_Os01g07990.1	1
2001	LOC_Os03g08400.1	3
2001	LOC_Os08g02000.1	8
2002	LOC_Os04g05800.1	4
2002	LOC_Os10g12330.1	10
2002	LOC_Os10g35890.1	10
2003	LOC_Os02g51260.1	2

2003	LOC_Os04g45780.1	4
2003	LOC_Os05g37100.1	5
2004	LOC_Os01g28220.1	1
2004	LOC_Os07g29930.1	7
2004	LOC_Os09g16060.1	9
2005	LOC_Os01g72710.1	1
2005	LOC_Os04g44290.1	4
2005	LOC_Os05g32230.1	5
2006	LOC_Os01g65620.1	1
2006	LOC_Os05g13690.1	5
2006	LOC_Os05g35240.1	5
2007	LOC_Os03g31740.1	3
2007	LOC_Os07g39720.1	7
2007	LOC_Os10g09680.1	10
2008	LOC_Os01g05220.1	1
2008	LOC_Os01g05260.1	1
2008	LOC_Os01g05230.1	1
2009	LOC_Os05g20910.1	5
2009	LOC_Os07g17430.1	7
2009	LOC_Os11g15720.1	11
2010	LOC_Os03g09960.1	3
2010	LOC_Os03g60509.1	3
2010	LOC_Os10g29040.1	10
2011	LOC_Os01g14360.1	1
2011	LOC_Os06g09380.1	6
2011	LOC_Os10g22356.1	10
2012	LOC_Os01g58850.1	1
2012	LOC_Os03g31310.1	3
2012	LOC_Os07g40710.1	7
2013	LOC_Os03g10960.1	3
2013	LOC_Os04g12550.1	4
2013	LOC_Os12g16760.1	12
2014	LOC_Os01g05450.1	1
2014	LOC_Os01g70020.1	1
2014	LOC_Os09g38400.1	9
2015	LOC_Os05g45140.1	5
2015	LOC_Os05g45170.1	5
2015	LOC_Os09g30510.1	9
2016	LOC_Os08g24470.1	8
2016	LOC_Os11g13900.1	11
2016	LOC_Os11g40900.1	11
2017	LOC_Os06g30870.1	6
2017	LOC_Os10g34320.1	10
2017	LOC_Os10g41050.1	10
2018	LOC_Os01g36820.1	1
2018	LOC_Os06g09140.1	6
2018	LOC_Os09g12060.1	9
2019	LOC_Os04g27250.1	4
2019	LOC_Os05g11260.1	5
2019	LOC_Os10g20270.1	10
2020	LOC_Os05g20660.1	5
2020	LOC_Os08g17020.1	8

2020	LOC_Os10g11240.1	10
2021	LOC_Os04g15880.1	4
2021	LOC_Os04g43860.1	4
2021	LOC_Os06g13250.1	6
2022	LOC_Os11g16970.1	11
2022	LOC_Os11g16990.1	11
2022	LOC_Os11g17580.1	11
2023	LOC_Os08g15610.1	8
2023	LOC_Os08g15710.1	8
2023	LOC_Os08g15780.1	8
2024	LOC_Os05g25620.1	5
2024	LOC_Os07g42790.1	7
2024	LOC_Os09g15680.1	9
2025	LOC_Os02g05420.1	2
2025	LOC_Os04g06350.1	4
2025	LOC_Os12g39350.1	12
2026	LOC_Os01g34670.1	1
2026	LOC_Os01g53660.1	1
2026	LOC_Os02g37950.1	2
2027	LOC_Os02g34310.1	2
2027	LOC_Os06g10450.1	6
2027	LOC_Os11g27380.1	11
2028	LOC_Os04g13590.1	4
2028	LOC_Os08g04490.1	8
2028	LOC_Os10g05300.1	10
2029	LOC_Os07g06220.1	7
2029	LOC_Os09g10680.1	9
2029	LOC_Os11g15540.1	11
2030	LOC_Os08g35330.1	8
2030	LOC_Os08g35400.1	8
2030	LOC_Os09g09670.1	9
2031	LOC_Os01g39520.1	1
2031	LOC_Os04g12050.1	4
2031	LOC_Os10g18980.1	10
2032	LOC_Os05g16060.1	5
2032	LOC_Os05g16070.1	5
2032	LOC_Os09g06380.1	9
2033	LOC_Os01g07670.1	1
2033	LOC_Os01g52080.1	1
2033	LOC_Os01g52440.1	1
2034	LOC_Os01g25990.1	1
2034	LOC_Os01g26039.1	1
2034	LOC_Os09g33530.1	9
2035	LOC_Os06g19570.1	6
2035	LOC_Os09g29880.1	9
2035	LOC_Os09g35820.1	9
2036	LOC_Os06g09160.1	6
2036	LOC_Os08g25040.1	8
2036	LOC_Os10g32280.1	10
2037	LOC_Os02g35460.1	2
2037	LOC_Os07g05900.1	7
2037	LOC_Os09g26200.1	9

2038	LOC_Os02g28560.1	2
2038	LOC_Os07g36860.1	7
2038	LOC_Os12g35910.1	12
2039	LOC_Os04g08836.1	4
2039	LOC_Os06g10260.1	6
2039	LOC_Os10g12080.1	10
2040	LOC_Os01g31990.1	1
2040	LOC_Os02g14620.1	2
2040	LOC_Os10g16610.1	10
2041	LOC_Os09g16500.1	9
2041	LOC_Os11g42400.1	11
2041	LOC_Os11g43650.1	11
2042	LOC_Os02g09590.1	2
2042	LOC_Os08g02800.1	8
2042	LOC_Os08g02840.1	8
2043	LOC_Os01g06650.1	1
2043	LOC_Os02g44030.1	2
2043	LOC_Os11g45890.1	11
2044	LOC_Os03g37790.1	3
2044	LOC_Os06g40680.1	6
2044	LOC_Os09g12410.1	9
2045	LOC_Os05g48470.1	5
2045	LOC_Os10g40220.1	10
2045	LOC_Os10g40230.1	10
2046	LOC_Os04g48960.1	4
2046	LOC_Os06g13620.1	6
2046	LOC_Os11g15000.1	11
2047	LOC_Os03g49560.1	3
2047	LOC_Os05g29800.1	5
2047	LOC_Os06g23810.1	6
2048	LOC_Os04g18540.1	4
2048	LOC_Os07g28150.1	7
2048	LOC_Os11g25900.1	11
2049	LOC_Os02g18100.1	2
2049	LOC_Os02g26070.1	2
2049	LOC_Os03g36710.1	3
2050	LOC_Os01g46340.1	1
2050	LOC_Os03g18300.1	3
2050	LOC_Os05g49820.1	5
2051	LOC_Os01g06940.1	1
2051	LOC_Os01g06980.1	1
2051	LOC_Os01g07000.1	1
2052	LOC_Os03g20240.1	3
2052	LOC_Os08g09540.1	8
2052	LOC_Os10g18330.1	10
2053	LOC_Os03g32870.1	3
2053	LOC_Os04g22550.1	4
2053	LOC_Os09g17210.1	9
2054	LOC_Os12g16090.1	12
2054	LOC_Os12g36310.1	12
2054	LOC_Os12g36350.1	12
2055	LOC_Os01g33120.1	1

2055	LOC_Os02g23824.1	2
2055	LOC_Os08g19280.1	8
2056	LOC_Os03g07480.1	3
2056	LOC_Os10g26470.1	10
2056	LOC_Os12g44380.1	12
2057	LOC_Os09g21790.1	9
2057	LOC_Os09g22520.1	9
2057	LOC_Os09g22420.1	9
2058	LOC_Os04g23140.1	4
2058	LOC_Os07g04120.1	7
2058	LOC_Os09g28820.1	9
2059	LOC_Os06g09050.1	6
2059	LOC_Os06g13730.1	6
2059	LOC_Os09g31160.1	9
2060	LOC_Os11g34690.1	11
2060	LOC_Os11g34700.1	11
2060	LOC_Os11g38150.1	11
2061	LOC_Os09g25800.1	9
2061	LOC_Os10g12400.1	10
2061	LOC_Os12g03920.1	12
2062	LOC_Os07g42000.1	7
2062	LOC_Os08g36710.1	8
2062	LOC_Os09g01970.1	9
2063	LOC_Os03g24630.1	3
2063	LOC_Os10g37560.1	10
2063	LOC_Os11g14590.1	11
2064	LOC_Os01g58150.1	1
2064	LOC_Os01g58140.1	1
2064	LOC_Os05g41920.1	5
2065	LOC_Os02g52490.1	2
2065	LOC_Os08g36950.1	8
2065	LOC_Os09g28520.1	9
2066	LOC_Os08g16810.1	8
2066	LOC_Os08g16820.1	8
2066	LOC_Os08g16830.1	8
2067	LOC_Os12g37750.1	12
2067	LOC_Os12g39970.1	12
2067	LOC_Os12g39870.1	12
2068	LOC_Os03g06210.1	3
2068	LOC_Os04g46270.1	4
2068	LOC_Os12g37620.1	12
2069	LOC_Os04g36710.1	4
2069	LOC_Os06g27980.1	6
2069	LOC_Os08g02150.1	8
2070	LOC_Os02g55170.1	2
2070	LOC_Os06g08540.1	6
2070	LOC_Os07g40520.1	7
2071	LOC_Os01g09090.1	1
2071	LOC_Os02g24200.1	2
2071	LOC_Os03g11430.1	3
2072	LOC_Os03g12440.1	3
2072	LOC_Os09g02790.1	9

2072	LOC_Os09g03080.1	9
2073	LOC_Os06g27530.1	6
2073	LOC_Os06g33780.1	6
2073	LOC_Os11g26640.1	11
2074	LOC_Os07g08310.1	7
2074	LOC_Os11g04150.1	11
2074	LOC_Os12g03950.1	12
2075	LOC_Os01g36740.1	1
2075	LOC_Os02g29610.1	2
2075	LOC_Os04g22950.1	4
2076	LOC_Os04g48360.1	4
2076	LOC_Os08g01200.1	8
2076	LOC_Os09g13080.1	9
2077	LOC_Os04g31140.1	4
2077	LOC_Os04g31880.1	4
2077	LOC_Os04g38230.1	4
2078	LOC_Os03g02010.1	3
2078	LOC_Os05g04330.1	5
2078	LOC_Os11g01810.1	11
2079	LOC_Os02g51850.1	2
2079	LOC_Os03g59170.1	3
2079	LOC_Os06g11740.1	6
2080	LOC_Os02g20950.1	2
2080	LOC_Os03g50420.1	3
2080	LOC_Os05g23530.1	5
2081	LOC_Os08g38540.1	8
2081	LOC_Os09g30170.1	9
2081	LOC_Os12g30950.1	12
2082	LOC_Os01g23360.1	1
2082	LOC_Os02g17630.1	2
2082	LOC_Os11g24460.1	11
2083	LOC_Os02g12520.1	2
2083	LOC_Os05g15380.1	5
2083	LOC_Os11g42730.1	11
2084	LOC_Os02g49930.1	2
2084	LOC_Os04g18300.1	4
2084	LOC_Os06g47090.1	6
2085	LOC_Os01g55030.1	1
2085	LOC_Os05g01050.1	5
2085	LOC_Os05g15520.1	5
2086	LOC_Os03g19660.1	3
2086	LOC_Os03g42740.1	3
2086	LOC_Os03g45910.1	3
2087	LOC_Os03g53380.1	3
2087	LOC_Os04g33350.1	4
2087	LOC_Os09g39370.1	9
2088	LOC_Os01g16920.1	1
2088	LOC_Os01g55330.1	1
2088	LOC_Os04g32580.1	4
2089	LOC_Os11g42570.1	11
2089	LOC_Os11g42680.1	11
2089	LOC_Os11g42700.1	11

2090	LOC_Os03g28230.1	3
2090	LOC_Os06g39250.1	6
2090	LOC_Os10g18880.1	10
2091	LOC_Os11g30120.1	11
2091	LOC_Os11g30220.1	11
2091	LOC_Os11g30230.1	11
2092	LOC_Os02g43990.1	2
2092	LOC_Os03g03050.1	3
2092	LOC_Os07g39580.1	7
2093	LOC_Os02g30570.1	2
2093	LOC_Os02g51580.1	2
2093	LOC_Os04g31530.1	4
2094	LOC_Os04g09750.1	4
2094	LOC_Os04g28730.1	4
2094	LOC_Os10g19090.1	10
2095	LOC_Os03g58790.1	3
2095	LOC_Os06g48250.1	6
2095	LOC_Os07g09490.1	7
2095	LOC_Os07g09420.1	7
2096	LOC_Os04g42110.1	4
2096	LOC_Os04g52690.1	4
2096	LOC_Os05g31220.1	5
2096	LOC_Os06g01980.1	6
2097	LOC_Os01g07870.1	1
2097	LOC_Os04g13220.1	4
2097	LOC_Os06g36650.1	6
2097	LOC_Os11g05700.1	11
2098	LOC_Os02g09720.1	2
2098	LOC_Os02g46680.1	2
2098	LOC_Os04g38570.1	4
2098	LOC_Os08g45030.1	8
2099	LOC_Os01g25386.1	1
2099	LOC_Os01g67580.1	1
2099	LOC_Os03g04920.1	3
2099	LOC_Os04g13210.1	4
2100	LOC_Os02g18670.1	2
2100	LOC_Os02g18700.1	2
2100	LOC_Os04g49900.1	4
2100	LOC_Os04g52900.1	4
2101	LOC_Os01g61940.1	1
2101	LOC_Os03g17350.1	3
2101	LOC_Os03g17370.1	3
2101	LOC_Os05g02870.1	5
2102	LOC_Os01g67480.1	1
2102	LOC_Os04g41229.1	4
2102	LOC_Os08g39630.1	8
2102	LOC_Os09g31300.1	9
2103	LOC_Os02g02480.1	2
2103	LOC_Os05g38140.1	5
2103	LOC_Os07g35870.1	7
2103	LOC_Os08g04390.1	8
2104	LOC_Os01g57580.1	1

2104	LOC_Os03g55220.1	3
2104	LOC_Os05g46370.1	5
2104	LOC_Os08g36740.1	8
2105	LOC_Os01g60260.1	1
2105	LOC_Os03g60110.1	3
2105	LOC_Os05g40290.1	5
2105	LOC_Os07g12490.1	7
2106	LOC_Os03g16000.1	3
2106	LOC_Os04g45670.1	4
2106	LOC_Os07g32460.1	7
2106	LOC_Os10g36870.1	10
2107	LOC_Os01g54480.1	1
2107	LOC_Os01g66860.1	1
2107	LOC_Os02g39560.1	2
2107	LOC_Os07g43900.1	7
2108	LOC_Os01g69910.1	1
2108	LOC_Os03g09100.1	3
2108	LOC_Os07g43030.1	7
2108	LOC_Os10g22950.1	10
2109	LOC_Os03g49170.1	3
2109	LOC_Os05g03760.1	5
2109	LOC_Os07g38090.1	7
2109	LOC_Os12g33090.1	12
2110	LOC_Os01g71590.1	1
2110	LOC_Os01g71540.1	1
2110	LOC_Os02g15790.1	2
2110	LOC_Os05g03320.1	5
2111	LOC_Os02g01240.1	2
2111	LOC_Os03g55330.1	3
2111	LOC_Os07g46500.1	7
2111	LOC_Os09g33810.1	9
2112	LOC_Os01g42320.1	1
2112	LOC_Os02g48860.1	2
2112	LOC_Os02g48870.1	2
2112	LOC_Os06g20190.1	6
2113	LOC_Os01g08330.1	1
2113	LOC_Os02g27360.1	2
2113	LOC_Os05g48330.1	5
2113	LOC_Os09g27940.1	9
2114	LOC_Os02g21040.1	2
2114	LOC_Os08g16660.1	8
2114	LOC_Os08g36530.1	8
2114	LOC_Os09g27910.1	9
2115	LOC_Os02g41710.1	2
2115	LOC_Os02g53340.1	2
2115	LOC_Os06g10580.1	6
2115	LOC_Os12g06570.1	12
2116	LOC_Os02g10740.1	2
2116	LOC_Os06g40200.1	6
2116	LOC_Os11g01270.1	11
2116	LOC_Os12g01240.1	12
2117	LOC_Os03g42840.1	3

2117	LOC_Os05g45810.1	5
2117	LOC_Os12g40510.1	12
2117	LOC_Os12g06510.1	12
2118	LOC_Os03g01890.1	3
2118	LOC_Os03g43930.1	3
2118	LOC_Os10g33960.1	10
2118	LOC_Os12g41860.1	12
2119	LOC_Os01g19694.1	1
2119	LOC_Os03g51690.1	3
2119	LOC_Os03g56110.1	3
2119	LOC_Os07g03770.1	7
2120	LOC_Os01g62920.1	1
2120	LOC_Os03g06930.1	3
2120	LOC_Os03g47740.1	3
2120	LOC_Os05g38120.1	5
2121	LOC_Os06g08910.1	6
2121	LOC_Os06g08930.1	6
2121	LOC_Os06g09090.1	6
2121	LOC_Os09g25930.1	9
2122	LOC_Os01g36350.1	1
2122	LOC_Os08g01480.1	8
2122	LOC_Os10g08230.1	10
2122	LOC_Os10g09110.1	10
2123	LOC_Os08g02996.1	8
2123	LOC_Os08g03020.1	8
2123	LOC_Os08g03170.1	8
2123	LOC_Os08g03240.1	8
2124	LOC_Os01g09200.1	1
2124	LOC_Os05g43570.1	5
2124	LOC_Os10g33640.1	10
2124	LOC_Os12g19290.1	12
2125	LOC_Os04g30040.1	4
2125	LOC_Os11g39490.1	11
2125	LOC_Os11g39530.1	11
2125	LOC_Os11g39420.1	11
2126	LOC_Os01g56330.1	1
2126	LOC_Os03g55210.1	3
2126	LOC_Os04g52860.1	4
2126	LOC_Os07g05370.1	7
2127	LOC_Os01g12720.1	1
2127	LOC_Os05g12680.1	5
2127	LOC_Os05g44030.1	5
2127	LOC_Os07g04520.1	7
2128	LOC_Os02g57420.1	2
2128	LOC_Os03g07430.1	3
2128	LOC_Os04g47620.1	4
2128	LOC_Os10g26520.1	10
2129	LOC_Os02g07790.1	2
2129	LOC_Os04g51950.1	4
2129	LOC_Os06g45300.1	6
2129	LOC_Os08g12750.1	8
2130	LOC_Os01g02420.1	1

2130	LOC_Os01g02600.1	1
2130	LOC_Os01g02770.1	1
2130	LOC_Os01g02400.1	1
2131	LOC_Os03g25070.1	3
2131	LOC_Os06g50030.1	6
2131	LOC_Os07g44710.1	7
2131	LOC_Os10g36710.1	10
2132	LOC_Os03g18370.1	3
2132	LOC_Os05g28520.1	5
2132	LOC_Os05g48660.1	5
2132	LOC_Os07g49240.1	7
2133	LOC_Os06g33720.1	6
2133	LOC_Os07g02120.1	7
2133	LOC_Os07g02140.1	7
2133	LOC_Os07g02100.1	7
2134	LOC_Os02g56850.1	2
2134	LOC_Os03g06740.1	3
2134	LOC_Os05g06750.1	5
2134	LOC_Os10g28000.1	10
2135	LOC_Os01g69920.1	1
2135	LOC_Os02g50480.1	2
2135	LOC_Os03g50860.1	3
2135	LOC_Os10g21810.1	10
2136	LOC_Os01g06290.1	1
2136	LOC_Os02g39720.1	2
2136	LOC_Os03g17710.1	3
2136	LOC_Os05g02880.1	5
2137	LOC_Os04g45930.1	4
2137	LOC_Os06g48310.1	6
2137	LOC_Os07g08320.1	7
2137	LOC_Os07g27110.1	7
2138	LOC_Os03g11960.1	3
2138	LOC_Os03g22810.1	3
2138	LOC_Os07g46990.1	7
2138	LOC_Os08g44770.1	8
2139	LOC_Os04g51970.1	4
2139	LOC_Os07g03910.1	7
2139	LOC_Os11g42430.1	11
2139	LOC_Os12g04640.1	12
2140	LOC_Os02g50880.1	2
2140	LOC_Os05g05480.1	5
2140	LOC_Os05g34460.1	5
2140	LOC_Os06g12780.1	6
2141	LOC_Os02g45390.1	2
2141	LOC_Os04g48310.1	4
2141	LOC_Os11g05300.1	11
2141	LOC_Os12g05370.1	12
2142	LOC_Os02g06920.1	2
2142	LOC_Os04g01490.1	4
2142	LOC_Os06g23274.1	6
2142	LOC_Os06g46366.1	6
2143	LOC_Os01g19800.1	1

2143	LOC_Os02g36740.1	2
2143	LOC_Os08g33860.1	8
2143	LOC_Os09g24650.1	9
2144	LOC_Os02g50930.1	2
2144	LOC_Os06g07100.1	6
2144	LOC_Os06g08820.1	6
2144	LOC_Os06g12680.1	6
2145	LOC_Os01g08340.1	1
2145	LOC_Os02g45240.1	2
2145	LOC_Os02g54624.1	2
2145	LOC_Os05g08610.1	5
2146	LOC_Os06g12560.1	6
2146	LOC_Os06g50370.1	6
2146	LOC_Os07g29600.1	7
2146	LOC_Os10g39770.1	10
2147	LOC_Os02g46600.1	2
2147	LOC_Os02g57460.1	2
2147	LOC_Os04g50100.1	4
2147	LOC_Os09g29370.1	9
2148	LOC_Os04g48260.1	4
2148	LOC_Os06g03580.1	6
2148	LOC_Os09g35690.1	9
2148	LOC_Os09g36460.1	9
2149	LOC_Os01g42500.1	1
2149	LOC_Os02g03950.1	2
2149	LOC_Os08g29590.1	8
2149	LOC_Os11g39130.1	11
2150	LOC_Os04g18190.1	4
2150	LOC_Os04g23950.1	4
2150	LOC_Os10g09650.1	10
2150	LOC_Os11g11800.1	11
2151	LOC_Os01g42170.1	1
2151	LOC_Os10g16870.1	10
2151	LOC_Os11g47432.1	11
2151	LOC_Os11g47438.1	11
2152	LOC_Os04g20840.1	4
2152	LOC_Os05g44870.1	5
2152	LOC_Os06g02740.1	6
2152	LOC_Os12g22180.1	12
2153	LOC_Os02g05830.1	2
2153	LOC_Os12g17600.1	12
2153	LOC_Os12g19470.1	12
2153	LOC_Os12g19394.1	12
2154	LOC_Os02g54890.1	2
2154	LOC_Os03g14540.1	3
2154	LOC_Os08g41440.1	8
2154	LOC_Os09g32670.1	9
2155	LOC_Os06g41810.1	6
2155	LOC_Os06g41840.1	6
2155	LOC_Os09g31490.1	9
2155	LOC_Os09g31514.1	9
2156	LOC_Os07g40250.1	7

2156	LOC_Os07g46970.1	7
2156	LOC_Os07g46980.1	7
2156	LOC_Os11g32030.1	11
2157	LOC_Os09g04730.1	9
2157	LOC_Os11g25700.1	11
2157	LOC_Os11g43200.1	11
2157	LOC_Os11g43360.1	11
2158	LOC_Os01g05840.1	1
2158	LOC_Os04g58200.1	4
2158	LOC_Os10g35370.1	10
2158	LOC_Os11g07930.1	11
2159	LOC_Os01g02020.1	1
2159	LOC_Os02g57260.1	2
2159	LOC_Os09g07830.1	9
2159	LOC_Os10g31950.1	10
2160	LOC_Os02g10320.1	2
2160	LOC_Os04g36800.1	4
2160	LOC_Os06g09630.1	6
2160	LOC_Os07g42420.1	7
2161	LOC_Os02g50240.1	2
2161	LOC_Os03g12290.1	3
2161	LOC_Os03g50490.1	3
2161	LOC_Os04g56400.1	4
2162	LOC_Os02g07630.1	2
2162	LOC_Os02g10290.1	2
2162	LOC_Os03g08070.1	3
2162	LOC_Os06g45500.1	6
2163	LOC_Os01g62840.1	1
2163	LOC_Os03g11050.1	3
2163	LOC_Os03g16150.1	3
2163	LOC_Os08g13930.1	8
2164	LOC_Os03g02100.1	3
2164	LOC_Os03g21740.1	3
2164	LOC_Os03g48850.1	3
2164	LOC_Os10g36210.1	10
2165	LOC_Os01g13229.1	1
2165	LOC_Os01g13260.1	1
2165	LOC_Os05g14730.1	5
2165	LOC_Os12g20324.1	12
2166	LOC_Os02g39260.1	2
2166	LOC_Os02g39240.1	2
2166	LOC_Os02g39420.1	2
2166	LOC_Os02g39430.1	2
2167	LOC_Os04g46660.1	4
2167	LOC_Os04g53680.1	4
2167	LOC_Os05g33040.1	5
2167	LOC_Os10g41430.1	10
2168	LOC_Os05g15510.1	5
2168	LOC_Os08g37750.1	8
2168	LOC_Os10g22520.1	10
2168	LOC_Os10g22570.1	10
2169	LOC_Os02g38260.1	2

2169	LOC_Os04g40490.1	4
2169	LOC_Os04g40500.1	4
2169	LOC_Os04g40510.1	4
2170	LOC_Os01g09700.1	1
2170	LOC_Os02g20360.1	2
2170	LOC_Os04g43650.1	4
2170	LOC_Os06g23684.1	6
2171	LOC_Os01g58610.1	1
2171	LOC_Os02g07260.1	2
2171	LOC_Os05g41640.1	5
2171	LOC_Os06g45710.1	6
2172	LOC_Os01g64020.1	1
2172	LOC_Os05g37170.1	5
2172	LOC_Os11g05480.1	11
2172	LOC_Os12g05680.1	12
2173	LOC_Os03g03550.1	3
2173	LOC_Os08g43090.1	8
2173	LOC_Os10g38820.1	10
2173	LOC_Os11g06170.1	11
2174	LOC_Os04g41820.1	4
2174	LOC_Os07g48180.1	7
2174	LOC_Os09g34060.1	9
2174	LOC_Os12g06520.1	12
2175	LOC_Os01g53250.1	1
2175	LOC_Os04g55960.1	4
2175	LOC_Os08g14570.1	8
2175	LOC_Os09g38620.1	9
2176	LOC_Os01g07910.1	1
2176	LOC_Os01g59930.1	1
2176	LOC_Os02g22260.1	2
2176	LOC_Os05g40990.1	5
2177	LOC_Os02g01340.1	2
2177	LOC_Os03g57120.1	3
2177	LOC_Os06g01850.1	6
2177	LOC_Os07g05400.1	7
2178	LOC_Os01g03520.1	1
2178	LOC_Os01g13280.1	1
2178	LOC_Os05g06120.1	5
2178	LOC_Os05g48390.1	5
2179	LOC_Os02g02210.1	2
2179	LOC_Os04g52440.1	4
2179	LOC_Os04g52450.1	4
2179	LOC_Os08g10510.1	8
2180	LOC_Os04g16760.1	4
2180	LOC_Os10g21250.1	10
2180	LOC_Os10g38229.1	10
2180	LOC_Os10g38248.1	10
2181	LOC_Os02g35000.1	2
2181	LOC_Os05g46620.1	5
2181	LOC_Os08g41110.1	8
2181	LOC_Os09g32050.1	9
2182	LOC_Os01g69930.1	1

2182	LOC_Os02g30620.1	2
2182	LOC_Os03g28310.1	3
2182	LOC_Os04g31940.1	4
2183	LOC_Os01g13360.1	1
2183	LOC_Os02g18840.1	2
2183	LOC_Os05g51230.1	5
2183	LOC_Os06g23290.1	6
2184	LOC_Os02g10060.1	2
2184	LOC_Os04g40420.1	4
2184	LOC_Os11g08080.1	11
2184	LOC_Os12g07730.1	12
2185	LOC_Os03g21240.1	3
2185	LOC_Os07g25710.1	7
2185	LOC_Os08g25820.1	8
2185	LOC_Os08g33750.1	8
2186	LOC_Os01g67054.1	1
2186	LOC_Os04g32950.1	4
2186	LOC_Os05g43170.1	5
2186	LOC_Os07g14270.1	7
2187	LOC_Os01g43130.1	1
2187	LOC_Os01g43120.1	1
2187	LOC_Os02g57980.1	2
2187	LOC_Os06g33520.1	6
2188	LOC_Os03g13300.1	3
2188	LOC_Os03g51080.1	3
2188	LOC_Os04g37460.1	4
2188	LOC_Os04g37500.1	4
2189	LOC_Os01g14830.1	1
2189	LOC_Os02g04460.1	2
2189	LOC_Os11g06750.1	11
2189	LOC_Os12g07010.1	12
2190	LOC_Os02g47140.1	2
2190	LOC_Os03g03020.1	3
2190	LOC_Os04g50990.1	4
2190	LOC_Os10g32870.1	10
2191	LOC_Os01g74440.1	1
2191	LOC_Os02g06860.1	2
2191	LOC_Os09g02830.1	9
2191	LOC_Os09g02780.1	9
2192	LOC_Os02g01230.1	2
2192	LOC_Os03g34040.1	3
2192	LOC_Os03g59310.1	3
2192	LOC_Os07g10660.1	7
2193	LOC_Os03g10060.1	3
2193	LOC_Os03g14530.1	3
2193	LOC_Os06g04290.1	6
2193	LOC_Os10g08930.1	10
2194	LOC_Os03g56460.1	3
2194	LOC_Os06g14510.1	6
2194	LOC_Os08g37380.1	8
2194	LOC_Os09g29070.1	9
2195	LOC_Os03g05310.1	3

2195	LOC_Os03g59100.1	3
2195	LOC_Os03g59110.1	3
2195	LOC_Os03g59120.1	3
2196	LOC_Os08g34050.1	8
2196	LOC_Os09g30240.1	9
2196	LOC_Os09g24910.1	9
2196	LOC_Os10g26570.1	10
2197	LOC_Os01g09570.1	1
2197	LOC_Os05g10650.1	5
2197	LOC_Os05g44922.1	5
2197	LOC_Os06g05860.1	6
2198	LOC_Os01g24680.1	1
2198	LOC_Os02g17390.1	2
2198	LOC_Os05g06300.1	5
2198	LOC_Os05g29880.1	5
2199	LOC_Os03g55930.1	3
2199	LOC_Os07g46440.1	7
2199	LOC_Os11g03400.1	11
2199	LOC_Os12g03090.1	12
2200	LOC_Os03g09820.1	3
2200	LOC_Os05g47480.1	5
2200	LOC_Os07g32080.1	7
2200	LOC_Os09g36440.1	9
2201	LOC_Os01g09320.1	1
2201	LOC_Os01g52500.1	1
2201	LOC_Os01g54030.1	1
2201	LOC_Os05g09440.1	5
2202	LOC_Os01g03530.1	1
2202	LOC_Os01g03620.1	1
2202	LOC_Os01g03630.1	1
2202	LOC_Os01g03640.1	1
2203	LOC_Os01g68760.1	1
2203	LOC_Os06g19470.1	6
2203	LOC_Os11g18940.1	11
2203	LOC_Os12g13100.1	12
2204	LOC_Os04g16790.1	4
2204	LOC_Os06g39700.1	6
2204	LOC_Os08g15250.1	8
2204	LOC_Os10g21330.1	10
2205	LOC_Os03g49710.1	3
2205	LOC_Os03g58050.1	3
2205	LOC_Os07g07709.1	7
2205	LOC_Os07g07770.1	7
2206	LOC_Os02g04730.1	2
2206	LOC_Os02g04750.1	2
2206	LOC_Os11g18366.1	11
2206	LOC_Os11g35710.1	11
2207	LOC_Os02g04710.1	2
2207	LOC_Os08g12730.1	8
2207	LOC_Os11g18194.1	11
2207	LOC_Os11g08569.1	11
2208	LOC_Os02g15220.1	2

2208	LOC_Os04g53130.1	4
2208	LOC_Os04g53170.1	4
2208	LOC_Os08g09340.1	8
2209	LOC_Os08g41530.1	8
2209	LOC_Os08g41540.1	8
2209	LOC_Os08g41620.1	8
2209	LOC_Os08g41640.1	8
2210	LOC_Os03g06630.1	3
2210	LOC_Os03g58160.1	3
2210	LOC_Os07g08140.1	7
2210	LOC_Os10g28340.1	10
2211	LOC_Os04g57300.1	4
2211	LOC_Os06g17290.1	6
2211	LOC_Os08g38200.1	8
2211	LOC_Os09g29890.1	9
2212	LOC_Os03g55150.1	3
2212	LOC_Os07g02210.1	7
2212	LOC_Os07g40580.1	7
2212	LOC_Os12g32240.1	12
2213	LOC_Os03g19300.1	3
2213	LOC_Os05g19630.1	5
2213	LOC_Os07g36250.1	7
2213	LOC_Os11g43600.1	11
2214	LOC_Os03g57220.1	3
2214	LOC_Os04g53210.1	4
2214	LOC_Os07g05820.1	7
2214	LOC_Os07g42440.1	7
2215	LOC_Os02g38840.1	2
2215	LOC_Os03g20300.1	3
2215	LOC_Os03g29950.1	3
2215	LOC_Os07g22350.1	7
2216	LOC_Os01g44220.1	1
2216	LOC_Os05g50380.1	5
2216	LOC_Os08g25734.1	8
2216	LOC_Os09g12660.1	9
2217	LOC_Os01g46750.1	1
2217	LOC_Os01g48910.1	1
2217	LOC_Os05g04170.1	5
2217	LOC_Os11g35400.1	11
2218	LOC_Os03g14090.1	3
2218	LOC_Os08g43500.1	8
2218	LOC_Os09g36550.1	9
2218	LOC_Os10g05730.1	10
2219	LOC_Os03g06970.1	3
2219	LOC_Os05g43040.1	5
2219	LOC_Os08g03620.1	8
2219	LOC_Os09g36740.1	9
2220	LOC_Os03g04500.1	3
2220	LOC_Os10g33290.1	10
2220	LOC_Os11g46230.1	11
2220	LOC_Os12g37780.1	12
2221	LOC_Os02g55270.1	2

2221	LOC_Os02g55280.1	2
2221	LOC_Os05g32430.1	5
2221	LOC_Os11g31890.1	11
2222	LOC_Os02g09930.1	2
2222	LOC_Os03g26044.1	3
2222	LOC_Os06g42020.1	6
2222	LOC_Os07g43710.1	7
2223	LOC_Os03g63320.1	3
2223	LOC_Os04g39090.1	4
2223	LOC_Os08g41480.1	8
2223	LOC_Os12g29480.1	12
2224	LOC_Os02g12300.1	2
2224	LOC_Os06g05260.1	6
2224	LOC_Os06g05209.1	6
2224	LOC_Os06g38510.1	6
2225	LOC_Os04g05050.1	4
2225	LOC_Os05g22800.1	5
2225	LOC_Os06g05272.1	6
2225	LOC_Os10g31910.1	10
2226	LOC_Os06g45360.1	6
2226	LOC_Os07g10540.1	7
2226	LOC_Os11g33330.1	11
2226	LOC_Os11g33340.1	11
2227	LOC_Os03g04270.1	3
2227	LOC_Os03g43390.1	3
2227	LOC_Os08g39060.1	8
2227	LOC_Os12g40860.1	12
2228	LOC_Os01g58520.1	1
2228	LOC_Os01g72410.1	1
2228	LOC_Os04g41380.1	4
2228	LOC_Os11g42100.1	11
2229	LOC_Os03g08110.1	3
2229	LOC_Os03g08100.1	3
2229	LOC_Os03g27110.1	3
2229	LOC_Os07g42140.1	7
2230	LOC_Os03g28960.1	3
2230	LOC_Os03g44484.1	3
2230	LOC_Os04g54840.1	4
2230	LOC_Os08g07480.1	8
2231	LOC_Os01g69900.1	1
2231	LOC_Os01g73040.1	1
2231	LOC_Os11g06930.1	11
2231	LOC_Os12g07190.1	12
2232	LOC_Os01g65500.1	1
2232	LOC_Os02g35190.1	2
2232	LOC_Os04g55210.1	4
2232	LOC_Os08g20570.1	8
2233	LOC_Os03g03430.1	3
2233	LOC_Os03g39640.1	3
2233	LOC_Os03g47120.1	3
2233	LOC_Os05g32850.1	5
2234	LOC_Os01g54540.1	1

2234	LOC_Os03g54890.1	3
2234	LOC_Os05g15370.1	5
2234	LOC_Os07g01870.1	7
2235	LOC_Os03g15870.1	3
2235	LOC_Os07g08330.1	7
2235	LOC_Os11g37510.1	11
2235	LOC_Os12g30490.1	12
2236	LOC_Os01g57420.1	1
2236	LOC_Os02g54650.1	2
2236	LOC_Os04g54200.1	4
2236	LOC_Os08g08110.1	8
2237	LOC_Os01g38980.1	1
2237	LOC_Os03g07110.1	3
2237	LOC_Os05g10840.1	5
2237	LOC_Os12g05420.1	12
2238	LOC_Os03g24220.1	3
2238	LOC_Os04g51440.1	4
2238	LOC_Os06g44890.1	6
2238	LOC_Os08g14230.1	8
2239	LOC_Os08g36994.1	8
2239	LOC_Os09g28610.1	9
2239	LOC_Os11g24560.1	11
2239	LOC_Os11g29200.1	11
2240	LOC_Os03g58530.1	3
2240	LOC_Os07g08880.1	7
2240	LOC_Os08g32620.1	8
2240	LOC_Os09g21770.1	9
2241	LOC_Os04g35430.1	4
2241	LOC_Os06g01170.1	6
2241	LOC_Os06g51450.1	6
2241	LOC_Os07g07690.1	7
2242	LOC_Os01g37050.1	1
2242	LOC_Os01g59510.1	1
2242	LOC_Os05g25400.1	5
2242	LOC_Os11g13420.1	11
2243	LOC_Os03g18180.1	3
2243	LOC_Os03g46480.1	3
2243	LOC_Os05g28190.1	5
2243	LOC_Os05g28290.1	5
2244	LOC_Os02g11790.1	2
2244	LOC_Os02g35530.1	2
2244	LOC_Os06g39370.1	6
2244	LOC_Os11g14140.1	11
2245	LOC_Os02g21240.1	2
2245	LOC_Os02g21260.1	2
2245	LOC_Os08g09450.1	8
2245	LOC_Os08g09640.1	8
2246	LOC_Os06g02054.1	6
2246	LOC_Os06g43740.1	6
2246	LOC_Os06g47400.1	6
2246	LOC_Os06g47680.1	6
2247	LOC_Os02g33240.1	2

2247	LOC_Os04g33820.1	4
2247	LOC_Os11g05660.1	11
2247	LOC_Os12g06030.1	12
2248	LOC_Os10g10420.1	10
2248	LOC_Os10g12200.1	10
2248	LOC_Os10g17930.1	10
2248	LOC_Os10g17940.1	10
2249	LOC_Os03g25190.1	3
2249	LOC_Os03g25220.1	3
2249	LOC_Os03g25240.1	3
2249	LOC_Os03g25250.1	3
2250	LOC_Os02g33840.1	2
2250	LOC_Os04g11790.1	4
2250	LOC_Os11g03680.1	11
2250	LOC_Os12g03440.1	12
2251	LOC_Os02g03910.1	2
2251	LOC_Os02g03980.1	2
2251	LOC_Os02g04000.1	2
2251	LOC_Os03g22750.1	3
2252	LOC_Os12g27750.1	12
2252	LOC_Os12g27790.1	12
2252	LOC_Os12g27810.1	12
2252	LOC_Os12g27760.1	12
2253	LOC_Os01g50616.1	1
2253	LOC_Os02g04030.1	2
2253	LOC_Os05g46720.1	5
2253	LOC_Os10g03400.1	10
2254	LOC_Os02g48990.1	2
2254	LOC_Os08g25310.1	8
2254	LOC_Os08g38850.1	8
2254	LOC_Os09g30330.1	9
2255	LOC_Os06g14060.1	6
2255	LOC_Os06g45730.1	6
2255	LOC_Os08g13070.1	8
2255	LOC_Os11g40490.1	11
2256	LOC_Os01g51390.1	1
2256	LOC_Os01g53700.1	1
2256	LOC_Os03g11410.1	3
2256	LOC_Os05g44916.1	5
2257	LOC_Os01g57082.1	1
2257	LOC_Os07g38280.1	7
2257	LOC_Os07g38260.1	7
2257	LOC_Os07g38270.1	7
2258	LOC_Os02g50620.1	2
2258	LOC_Os04g02900.1	4
2258	LOC_Os06g13720.1	6
2258	LOC_Os12g08260.1	12
2259	LOC_Os04g29000.1	4
2259	LOC_Os07g41460.1	7
2259	LOC_Os10g11270.1	10
2259	LOC_Os11g15370.1	11
2260	LOC_Os01g63810.1	1

2260	LOC_Os05g37450.1	5
2260	LOC_Os08g23430.1	8
2260	LOC_Os12g20150.1	12
2261	LOC_Os01g17010.1	1
2261	LOC_Os03g21680.1	3
2261	LOC_Os05g01030.1	5
2261	LOC_Os08g29150.1	8
2262	LOC_Os05g49250.1	5
2262	LOC_Os05g50290.1	5
2262	LOC_Os06g13070.1	6
2262	LOC_Os09g01290.1	9
2263	LOC_Os02g33610.1	2
2263	LOC_Os03g63590.1	3
2263	LOC_Os09g23380.1	9
2263	LOC_Os09g39590.1	9
2264	LOC_Os01g12030.1	1
2264	LOC_Os01g12070.1	1
2264	LOC_Os04g57860.1	4
2264	LOC_Os05g12150.1	5
2265	LOC_Os09g23690.1	9
2265	LOC_Os09g35710.1	9
2265	LOC_Os10g37630.1	10
2265	LOC_Os11g18880.1	11
2266	LOC_Os04g30800.1	4
2266	LOC_Os05g45310.1	5
2266	LOC_Os08g43470.1	8
2266	LOC_Os11g28340.1	11
2267	LOC_Os01g04800.1	1
2267	LOC_Os01g04750.1	1
2267	LOC_Os01g49830.1	1
2267	LOC_Os05g47650.1	5
2268	LOC_Os03g64260.1	3
2268	LOC_Os07g22770.1	7
2268	LOC_Os07g22730.1	7
2268	LOC_Os09g39850.1	9
2269	LOC_Os01g73770.1	1
2269	LOC_Os02g45420.1	2
2269	LOC_Os03g02650.1	3
2269	LOC_Os04g36640.1	4
2270	LOC_Os02g12350.1	2
2270	LOC_Os02g12380.1	2
2270	LOC_Os04g33480.1	4
2270	LOC_Os08g25570.1	8
2271	LOC_Os01g67640.1	1
2271	LOC_Os04g41410.1	4
2271	LOC_Os06g49240.1	6
2271	LOC_Os08g41590.1	8
2272	LOC_Os02g17620.1	2
2272	LOC_Os02g17640.1	2
2272	LOC_Os02g39400.1	2
2272	LOC_Os04g44420.1	4
2273	LOC_Os03g14000.1	3

2273	LOC_Os03g37960.1	3
2273	LOC_Os06g02490.1	6
2273	LOC_Os08g06550.1	8
2274	LOC_Os01g02870.1	1
2274	LOC_Os01g10980.1	1
2274	LOC_Os02g02310.1	2
2274	LOC_Os08g44750.1	8
2275	LOC_Os04g38430.1	4
2275	LOC_Os08g31130.1	8
2275	LOC_Os09g25810.1	9
2275	LOC_Os10g12500.1	10
2276	LOC_Os03g18380.1	3
2276	LOC_Os07g49230.1	7
2276	LOC_Os11g01510.1	11
2276	LOC_Os12g01520.1	12
2277	LOC_Os02g44770.1	2
2277	LOC_Os02g45690.1	2
2277	LOC_Os04g47320.1	4
2277	LOC_Os04g48940.1	4
2278	LOC_Os01g61710.1	1
2278	LOC_Os05g24594.1	5
2278	LOC_Os08g28080.1	8
2278	LOC_Os12g34370.1	12
2279	LOC_Os01g52330.1	1
2279	LOC_Os01g52360.1	1
2279	LOC_Os02g41760.1	2
2279	LOC_Os07g02570.1	7
2280	LOC_Os02g10550.1	2
2280	LOC_Os07g02560.1	7
2280	LOC_Os07g02620.1	7
2280	LOC_Os12g25170.1	12
2281	LOC_Os11g37940.1	11
2281	LOC_Os11g37970.1	11
2281	LOC_Os11g37950.1	11
2281	LOC_Os11g37960.1	11
2282	LOC_Os01g53000.1	1
2282	LOC_Os01g54560.1	1
2282	LOC_Os05g44100.1	5
2282	LOC_Os09g23350.1	9
2283	LOC_Os03g16900.1	3
2283	LOC_Os05g23860.1	5
2283	LOC_Os05g34540.1	5
2283	LOC_Os07g16970.1	7
2284	LOC_Os01g69950.1	1
2284	LOC_Os04g25540.1	4
2284	LOC_Os08g31219.1	8
2284	LOC_Os08g31228.1	8
2285	LOC_Os02g39870.1	2
2285	LOC_Os04g35180.1	4
2285	LOC_Os08g25090.1	8
2285	LOC_Os09g11250.1	9
2286	LOC_Os02g24020.1	2

2286	LOC_Os03g02570.1	3
2286	LOC_Os03g02690.1	3
2286	LOC_Os03g14120.1	3
2287	LOC_Os01g63890.1	1
2287	LOC_Os04g55290.1	4
2287	LOC_Os05g37390.1	5
2287	LOC_Os12g05410.1	12
2288	LOC_Os03g62720.1	3
2288	LOC_Os03g63720.1	3
2288	LOC_Os08g02360.1	8
2288	LOC_Os12g21500.1	12
2289	LOC_Os03g30300.1	3
2289	LOC_Os07g41280.1	7
2289	LOC_Os08g43370.1	8
2289	LOC_Os09g35970.1	9
2290	LOC_Os01g59140.1	1
2290	LOC_Os04g41040.1	4
2290	LOC_Os11g04730.1	11
2290	LOC_Os12g04510.1	12
2291	LOC_Os01g04130.1	1
2291	LOC_Os01g49900.1	1
2291	LOC_Os03g22610.1	3
2291	LOC_Os05g47630.1	5
2292	LOC_Os01g01302.1	1
2292	LOC_Os02g51410.1	2
2292	LOC_Os04g54800.1	4
2292	LOC_Os06g12150.1	6
2293	LOC_Os01g09890.1	1
2293	LOC_Os01g58220.1	1
2293	LOC_Os05g41900.1	5
2293	LOC_Os07g34589.1	7
2294	LOC_Os02g27200.1	2
2294	LOC_Os02g35320.1	2
2294	LOC_Os03g05900.1	3
2294	LOC_Os03g05910.1	3
2295	LOC_Os02g51880.1	2
2295	LOC_Os04g47270.1	4
2295	LOC_Os08g04780.1	8
2295	LOC_Os10g38850.1	10
2296	LOC_Os02g13890.1	2
2296	LOC_Os06g35910.1	6
2296	LOC_Os07g17130.1	7
2296	LOC_Os07g42500.1	7
2297	LOC_Os02g22090.1	2
2297	LOC_Os03g14260.1	3
2297	LOC_Os03g46330.1	3
2297	LOC_Os04g02690.1	4
2298	LOC_Os01g01660.1	1
2298	LOC_Os05g01970.1	5
2298	LOC_Os12g16410.1	12
2298	LOC_Os12g16220.1	12
2299	LOC_Os03g24710.1	3

2299	LOC_Os03g31430.1	3
2299	LOC_Os05g24500.1	5
2299	LOC_Os11g28500.1	11
2300	LOC_Os02g17780.1	2
2300	LOC_Os02g36210.1	2
2300	LOC_Os04g09900.1	4
2300	LOC_Os04g10060.1	4
2301	LOC_Os01g53670.1	1
2301	LOC_Os01g62370.1	1
2301	LOC_Os04g51120.1	4
2301	LOC_Os08g45230.1	8
2302	LOC_Os02g07900.1	2
2302	LOC_Os05g47550.1	5
2302	LOC_Os06g07830.1	6
2302	LOC_Os06g48630.1	6
2303	LOC_Os01g34330.1	1
2303	LOC_Os01g42460.1	1
2303	LOC_Os02g50840.1	2
2303	LOC_Os02g50850.1	2
2304	LOC_Os04g11510.1	4
2304	LOC_Os04g52380.1	4
2304	LOC_Os08g10580.1	8
2304	LOC_Os12g42550.1	12
2305	LOC_Os03g27230.1	3
2305	LOC_Os07g42960.1	7
2305	LOC_Os08g37790.1	8
2305	LOC_Os10g41480.1	10
2306	LOC_Os08g35070.1	8
2306	LOC_Os09g11170.1	9
2306	LOC_Os09g25190.1	9
2306	LOC_Os09g25220.1	9
2307	LOC_Os03g03070.1	3
2307	LOC_Os04g38780.1	4
2307	LOC_Os06g23980.1	6
2307	LOC_Os12g31748.1	12
2308	LOC_Os01g27780.1	1
2308	LOC_Os01g27750.1	1
2308	LOC_Os10g35840.1	10
2308	LOC_Os10g38930.1	10
2309	LOC_Os01g67550.1	1
2309	LOC_Os03g62260.1	3
2309	LOC_Os04g34580.1	4
2309	LOC_Os06g39660.1	6
2310	LOC_Os06g49810.1	6
2310	LOC_Os06g51160.1	6
2310	LOC_Os09g36190.1	9
2310	LOC_Os10g21890.1	10
2311	LOC_Os01g65780.1	1
2311	LOC_Os02g35020.1	2
2311	LOC_Os03g08600.1	3
2311	LOC_Os05g35200.1	5
2312	LOC_Os01g72690.1	1

2312	LOC_Os05g32210.1	5
2312	LOC_Os09g17680.1	9
2312	LOC_Os11g08670.1	11
2313	LOC_Os01g01610.1	1
2313	LOC_Os06g05400.1	6
2313	LOC_Os08g28230.1	8
2313	LOC_Os12g30030.1	12
2314	LOC_Os01g62620.1	1
2314	LOC_Os02g57370.1	2
2314	LOC_Os05g38360.1	5
2314	LOC_Os07g28460.1	7
2315	LOC_Os01g56670.1	1
2315	LOC_Os02g50780.1	2
2315	LOC_Os04g51690.1	4
2315	LOC_Os05g11850.1	5
2316	LOC_Os03g38580.1	3
2316	LOC_Os04g14130.1	4
2316	LOC_Os05g30710.1	5
2316	LOC_Os05g23960.1	5
2317	LOC_Os02g18810.1	2
2317	LOC_Os03g25380.1	3
2317	LOC_Os06g29800.1	6
2317	LOC_Os09g24680.1	9
2318	LOC_Os03g11670.1	3
2318	LOC_Os06g07550.1	6
2318	LOC_Os07g30930.1	7
2318	LOC_Os11g27240.1	11
2319	LOC_Os02g39790.1	2
2319	LOC_Os04g42090.1	4
2319	LOC_Os05g04990.1	5
2319	LOC_Os09g25620.1	9
2320	LOC_Os06g10770.1	6
2320	LOC_Os07g14610.1	7
2320	LOC_Os08g40110.1	8
2320	LOC_Os12g40550.1	12
2321	LOC_Os03g07000.1	3
2321	LOC_Os03g58880.1	3
2321	LOC_Os07g09630.1	7
2321	LOC_Os10g27340.1	10
2322	LOC_Os02g45380.1	2
2322	LOC_Os06g36330.1	6
2322	LOC_Os09g35600.1	9
2322	LOC_Os12g36660.1	12
2323	LOC_Os02g14190.1	2
2323	LOC_Os02g15550.1	2
2323	LOC_Os06g33710.1	6
2323	LOC_Os07g22600.1	7
2324	LOC_Os02g03900.1	2
2324	LOC_Os06g46310.1	6
2324	LOC_Os07g15370.1	7
2324	LOC_Os07g15460.1	7
2325	LOC_Os02g47570.1	2

2325	LOC_Os02g44290.1	2
2325	LOC_Os03g17940.1	3
2325	LOC_Os03g59070.1	3
2326	LOC_Os01g41670.1	1
2326	LOC_Os04g42460.1	4
2326	LOC_Os04g43324.1	4
2326	LOC_Os12g42140.1	12
2327	LOC_Os04g40600.1	4
2327	LOC_Os04g40620.1	4
2327	LOC_Os06g04650.1	6
2327	LOC_Os10g41400.1	10
2328	LOC_Os01g54050.1	1
2328	LOC_Os05g44570.1	5
2328	LOC_Os08g44350.1	8
2328	LOC_Os09g39400.1	9
2329	LOC_Os02g43090.1	2
2329	LOC_Os06g05130.1	6
2329	LOC_Os06g39520.1	6
2329	LOC_Os11g43820.1	11
2330	LOC_Os08g41300.1	8
2330	LOC_Os09g32520.1	9
2330	LOC_Os09g32500.1	9
2330	LOC_Os09g32532.1	9
2331	LOC_Os04g25060.1	4
2331	LOC_Os04g25650.1	4
2331	LOC_Os04g56430.1	4
2331	LOC_Os07g35320.1	7
2332	LOC_Os03g60750.1	3
2332	LOC_Os05g49230.1	5
2332	LOC_Os06g49140.1	6
2332	LOC_Os09g27270.1	9
2333	LOC_Os01g55650.1	1
2333	LOC_Os03g59620.1	3
2333	LOC_Os11g34370.1	11
2333	LOC_Os11g40009.1	11
2334	LOC_Os01g71010.1	1
2334	LOC_Os05g30900.1	5
2334	LOC_Os07g47250.1	7
2334	LOC_Os11g43510.1	11
2335	LOC_Os01g67430.1	1
2335	LOC_Os01g67450.1	1
2335	LOC_Os11g19290.1	11
2335	LOC_Os11g19340.1	11
2336	LOC_Os07g04840.1	7
2336	LOC_Os07g17390.1	7
2336	LOC_Os08g25900.1	8
2336	LOC_Os12g37710.1	12
2337	LOC_Os02g27480.1	2
2337	LOC_Os02g50340.1	2
2337	LOC_Os05g48360.1	5
2337	LOC_Os06g14050.1	6
2338	LOC_Os01g50360.1	1

2338	LOC_Os01g71230.1	1
2338	LOC_Os03g02960.1	3
2338	LOC_Os05g31000.1	5
2339	LOC_Os01g45390.1	1
2339	LOC_Os01g01920.1	1
2339	LOC_Os02g08260.1	2
2339	LOC_Os10g34470.1	10
2340	LOC_Os03g20420.1	3
2340	LOC_Os07g48750.1	7
2340	LOC_Os11g03730.1	11
2340	LOC_Os12g03470.1	12
2341	LOC_Os03g26630.1	3
2341	LOC_Os11g29340.1	11
2341	LOC_Os12g06980.1	12
2341	LOC_Os12g25640.1	12
2342	LOC_Os07g26900.1	7
2342	LOC_Os07g48160.1	7
2342	LOC_Os10g35070.1	10
2342	LOC_Os10g35110.1	10
2343	LOC_Os01g05800.1	1
2343	LOC_Os03g02480.1	3
2343	LOC_Os03g62750.1	3
2343	LOC_Os10g37690.1	10
2344	LOC_Os01g61890.1	1
2344	LOC_Os03g15580.1	3
2344	LOC_Os04g53460.1	4
2344	LOC_Os07g27140.1	7
2345	LOC_Os01g68620.1	1
2345	LOC_Os02g57710.1	2
2345	LOC_Os06g51430.1	6
2345	LOC_Os11g24540.1	11
2346	LOC_Os02g20410.1	2
2346	LOC_Os03g58240.1	3
2346	LOC_Os05g48860.1	5
2346	LOC_Os07g08400.1	7
2347	LOC_Os01g03740.1	1
2347	LOC_Os01g03730.1	1
2347	LOC_Os04g54390.1	4
2347	LOC_Os04g55850.1	4
2348	LOC_Os02g07210.1	2
2348	LOC_Os03g64219.1	3
2348	LOC_Os04g32970.1	4
2348	LOC_Os06g45850.1	6
2349	LOC_Os04g59430.1	4
2349	LOC_Os07g08530.1	7
2349	LOC_Os07g08540.1	7
2349	LOC_Os07g08600.1	7
2350	LOC_Os11g31330.1	11
2350	LOC_Os11g31340.1	11
2350	LOC_Os11g31360.1	11
2350	LOC_Os11g31380.1	11
2351	LOC_Os01g34850.1	1

2351	LOC_Os04g51820.1	4
2351	LOC_Os06g48800.1	6
2351	LOC_Os06g48810.1	6
2352	LOC_Os02g37654.1	2
2352	LOC_Os02g37700.1	2
2352	LOC_Os02g37800.1	2
2352	LOC_Os10g08026.1	10
2353	LOC_Os01g62380.1	1
2353	LOC_Os03g03600.1	3
2353	LOC_Os04g48490.1	4
2353	LOC_Os05g38500.1	5
2354	LOC_Os03g25400.1	3
2354	LOC_Os07g44330.1	7
2354	LOC_Os09g10770.1	9
2354	LOC_Os09g37930.1	9
2355	LOC_Os02g27000.1	2
2355	LOC_Os06g28060.1	6
2355	LOC_Os10g10190.1	10
2355	LOC_Os11g26830.1	11
2356	LOC_Os03g51479.1	3
2356	LOC_Os03g12810.1	3
2356	LOC_Os11g34850.1	11
2356	LOC_Os11g34770.1	11
2357	LOC_Os01g56610.1	1
2357	LOC_Os03g12110.1	3
2357	LOC_Os10g28630.1	10
2357	LOC_Os12g41390.1	12
2358	LOC_Os01g66280.1	1
2358	LOC_Os05g34720.1	5
2358	LOC_Os10g18370.1	10
2358	LOC_Os10g34390.1	10
2359	LOC_Os01g15900.1	1
2359	LOC_Os01g17000.1	1
2359	LOC_Os03g07360.1	3
2359	LOC_Os07g48570.1	7
2360	LOC_Os07g02030.1	7
2360	LOC_Os09g20530.1	9
2360	LOC_Os10g33980.1	10
2360	LOC_Os11g06520.1	11
2361	LOC_Os01g48180.1	1
2361	LOC_Os02g35100.1	2
2361	LOC_Os04g35880.1	4
2361	LOC_Os07g42750.1	7
2362	LOC_Os03g12180.1	3
2362	LOC_Os04g40660.1	4
2362	LOC_Os05g05450.1	5
2362	LOC_Os08g02690.1	8
2363	LOC_Os08g10780.1	8
2363	LOC_Os12g38990.1	12
2363	LOC_Os12g39020.1	12
2363	LOC_Os12g39030.1	12
2364	LOC_Os01g05030.1	1

2364	LOC_Os01g15380.1	1
2364	LOC_Os06g07440.1	6
2364	LOC_Os06g12480.1	6
2365	LOC_Os02g55830.1	2
2365	LOC_Os03g13880.1	3
2365	LOC_Os07g17290.1	7
2365	LOC_Os07g28650.1	7
2366	LOC_Os05g40090.1	5
2366	LOC_Os06g07370.1	6
2366	LOC_Os07g17850.1	7
2366	LOC_Os12g27980.1	12
2367	LOC_Os02g50640.1	2
2367	LOC_Os06g13600.1	6
2367	LOC_Os07g33630.1	7
2367	LOC_Os09g38710.1	9
2368	LOC_Os01g50910.1	1
2368	LOC_Os02g15250.1	2
2368	LOC_Os04g52110.1	4
2368	LOC_Os05g46480.1	5
2369	LOC_Os03g07140.1	3
2369	LOC_Os07g30600.1	7
2369	LOC_Os08g44360.1	8
2369	LOC_Os09g39410.1	9
2370	LOC_Os03g05110.1	3
2370	LOC_Os10g32080.1	10
2370	LOC_Os10g32160.1	10
2370	LOC_Os10g32170.1	10
2371	LOC_Os02g34950.1	2
2371	LOC_Os03g19870.1	3
2371	LOC_Os03g21914.1	3
2371	LOC_Os03g50620.1	3
2372	LOC_Os03g06620.1	3
2372	LOC_Os04g27830.1	4
2372	LOC_Os10g28350.1	10
2372	LOC_Os10g28360.1	10
2373	LOC_Os02g55210.1	2
2373	LOC_Os07g24050.1	7
2373	LOC_Os08g31740.1	8
2373	LOC_Os11g10920.1	11
2374	LOC_Os03g58080.1	3
2374	LOC_Os04g42130.1	4
2374	LOC_Os06g08750.1	6
2374	LOC_Os07g24230.1	7
2375	LOC_Os01g14100.1	1
2375	LOC_Os05g32320.1	5
2375	LOC_Os07g07654.1	7
2375	LOC_Os09g25560.1	9
2376	LOC_Os02g26430.1	2
2376	LOC_Os03g53050.1	3
2376	LOC_Os08g13840.1	8
2376	LOC_Os12g40570.1	12
2377	LOC_Os02g49460.1	2

2377	LOC_Os03g21090.1	3
2377	LOC_Os06g17390.1	6
2377	LOC_Os09g29940.1	9
2378	LOC_Os01g05120.1	1
2378	LOC_Os01g35040.1	1
2378	LOC_Os02g08480.1	2
2378	LOC_Os04g48770.1	4
2379	LOC_Os02g53160.1	2
2379	LOC_Os06g10650.1	6
2379	LOC_Os09g05020.1	9
2379	LOC_Os12g23190.1	12
2380	LOC_Os01g12580.1	1
2380	LOC_Os01g43530.1	1
2380	LOC_Os03g62620.1	3
2380	LOC_Os05g50710.1	5
2381	LOC_Os01g39860.1	1
2381	LOC_Os02g53180.1	2
2381	LOC_Os06g37590.1	6
2381	LOC_Os09g27820.1	9
2382	LOC_Os02g07880.1	2
2382	LOC_Os06g45110.1	6
2382	LOC_Os06g48700.1	6
2382	LOC_Os08g12890.1	8
2383	LOC_Os01g08460.1	1
2383	LOC_Os02g54990.1	2
2383	LOC_Os03g34300.1	3
2383	LOC_Os04g42720.1	4
2384	LOC_Os01g22510.1	1
2384	LOC_Os01g56560.1	1
2384	LOC_Os01g74010.1	1
2384	LOC_Os03g62170.1	3
2385	LOC_Os01g61930.1	1
2385	LOC_Os03g22870.1	3
2385	LOC_Os05g38730.1	5
2385	LOC_Os07g45160.1	7
2386	LOC_Os01g39270.1	1
2386	LOC_Os02g35500.1	2
2386	LOC_Os05g49070.1	5
2386	LOC_Os06g14740.1	6
2387	LOC_Os01g05470.1	1
2387	LOC_Os01g44230.1	1
2387	LOC_Os02g19130.1	2
2387	LOC_Os05g06130.1	5
2388	LOC_Os01g05440.1	1
2388	LOC_Os03g39910.1	3
2388	LOC_Os04g21730.1	4
2388	LOC_Os12g38440.1	12
2389	LOC_Os05g40570.1	5
2389	LOC_Os05g40580.1	5
2389	LOC_Os05g40590.1	5
2389	LOC_Os05g40510.1	5
2390	LOC_Os04g57050.1	4

2390	LOC_Os04g57080.1	4
2390	LOC_Os04g57090.1	4
2390	LOC_Os06g21820.1	6
2391	LOC_Os04g16440.1	4
2391	LOC_Os05g28220.1	5
2391	LOC_Os08g11330.1	8
2391	LOC_Os09g09290.1	9
2392	LOC_Os03g29340.1	3
2392	LOC_Os03g61210.1	3
2392	LOC_Os07g14090.1	7
2392	LOC_Os07g32230.1	7
2393	LOC_Os02g44910.1	2
2393	LOC_Os04g47530.1	4
2393	LOC_Os05g44080.1	5
2393	LOC_Os06g51100.1	6
2394	LOC_Os03g64030.1	3
2394	LOC_Os03g64050.1	3
2394	LOC_Os04g01980.1	4
2394	LOC_Os04g20810.1	4
2395	LOC_Os03g29570.1	3
2395	LOC_Os03g38020.1	3
2395	LOC_Os08g06610.1	8
2395	LOC_Os10g25674.1	10
2396	LOC_Os04g49980.1	4
2396	LOC_Os06g02040.1	6
2396	LOC_Os06g21910.1	6
2396	LOC_Os08g23870.1	8
2397	LOC_Os02g02500.1	2
2397	LOC_Os03g02840.1	3
2397	LOC_Os04g52920.1	4
2397	LOC_Os12g41940.1	12
2398	LOC_Os02g37080.1	2
2398	LOC_Os02g49590.1	2
2398	LOC_Os03g15750.1	3
2398	LOC_Os09g20630.1	9
2399	LOC_Os01g13430.1	1
2399	LOC_Os03g42050.1	3
2399	LOC_Os10g33770.1	10
2399	LOC_Os11g47130.1	11
2400	LOC_Os01g11070.1	1
2400	LOC_Os01g39810.1	1
2400	LOC_Os02g02110.1	2
2400	LOC_Os04g46050.1	4
2401	LOC_Os01g56100.1	1
2401	LOC_Os03g52000.1	3
2401	LOC_Os05g43520.1	5
2401	LOC_Os06g07820.1	6
2402	LOC_Os01g38510.1	1
2402	LOC_Os11g01300.1	11
2402	LOC_Os12g01260.1	12
2402	LOC_Os12g07490.1	12
2403	LOC_Os02g01160.1	2

2403	LOC_Os07g43220.1	7
2403	LOC_Os07g43230.1	7
2403	LOC_Os11g48030.1	11
2404	LOC_Os03g37100.1	3
2404	LOC_Os06g23504.1	6
2404	LOC_Os11g04850.1	11
2404	LOC_Os12g04860.1	12
2405	LOC_Os01g12800.1	1
2405	LOC_Os01g53060.1	1
2405	LOC_Os03g38730.1	3
2405	LOC_Os12g32330.1	12
2406	LOC_Os02g13270.1	2
2406	LOC_Os08g45210.1	8
2406	LOC_Os11g03670.1	11
2406	LOC_Os12g03430.1	12
2407	LOC_Os01g22630.1	1
2407	LOC_Os03g20180.1	3
2407	LOC_Os04g04000.1	4
2407	LOC_Os05g01520.1	5
2408	LOC_Os02g54120.1	2
2408	LOC_Os03g44900.1	3
2408	LOC_Os03g52594.1	3
2408	LOC_Os05g40790.1	5
2409	LOC_Os02g39070.1	2
2409	LOC_Os03g11100.1	3
2409	LOC_Os07g40320.1	7
2409	LOC_Os10g19190.1	10
2410	LOC_Os02g09420.1	2
2410	LOC_Os06g43180.1	6
2410	LOC_Os08g23100.1	8
2410	LOC_Os09g02270.1	9
2411	LOC_Os02g16880.1	2
2411	LOC_Os05g36110.1	5
2411	LOC_Os05g45280.1	5
2411	LOC_Os12g29750.1	12
2412	LOC_Os06g50450.1	6
2412	LOC_Os06g50630.1	6
2412	LOC_Os06g50780.1	6
2412	LOC_Os08g41570.1	8
2413	LOC_Os01g20760.1	1
2413	LOC_Os01g40910.1	1
2413	LOC_Os06g11570.1	6
2413	LOC_Os11g22190.1	11
2414	LOC_Os03g38480.1	3
2414	LOC_Os08g34320.1	8
2414	LOC_Os09g25500.1	9
2414	LOC_Os10g40620.1	10
2415	LOC_Os03g10820.1	3
2415	LOC_Os03g55470.1	3
2415	LOC_Os07g02470.1	7
2415	LOC_Os10g20510.1	10
2416	LOC_Os01g14720.1	1

2416	LOC_Os03g25430.1	3
2416	LOC_Os03g50110.1	3
2416	LOC_Os07g44200.1	7
2417	LOC_Os03g31400.1	3
2417	LOC_Os04g30470.1	4
2417	LOC_Os04g49500.1	4
2417	LOC_Os08g13780.1	8
2418	LOC_Os01g56790.1	1
2418	LOC_Os03g15410.1	3
2418	LOC_Os03g50100.1	3
2418	LOC_Os10g27480.1	10
2419	LOC_Os01g72380.1	1
2419	LOC_Os07g26560.1	7
2419	LOC_Os11g42080.1	11
2419	LOC_Os11g42030.1	11
2420	LOC_Os06g02680.1	6
2420	LOC_Os06g02830.1	6
2420	LOC_Os06g03050.1	6
2420	LOC_Os06g03380.1	6
2421	LOC_Os02g45700.1	2
2421	LOC_Os03g03270.1	3
2421	LOC_Os04g48950.1	4
2421	LOC_Os10g39020.1	10
2422	LOC_Os07g26210.1	7
2422	LOC_Os08g01940.1	8
2422	LOC_Os10g33420.1	10
2422	LOC_Os11g13810.1	11
2423	LOC_Os03g16060.1	3
2423	LOC_Os03g40930.1	3
2423	LOC_Os07g40150.1	7
2423	LOC_Os12g39160.1	12
2424	LOC_Os04g54420.1	4
2424	LOC_Os09g39270.1	9
2424	LOC_Os11g03570.1	11
2424	LOC_Os12g03340.1	12
2425	LOC_Os01g67250.1	1
2425	LOC_Os04g41110.1	4
2425	LOC_Os05g50410.1	5
2425	LOC_Os08g16610.1	8
2426	LOC_Os03g30250.1	3
2426	LOC_Os03g30260.1	3
2426	LOC_Os06g47110.1	6
2426	LOC_Os07g41320.1	7
2427	LOC_Os01g64410.1	1
2427	LOC_Os04g58820.1	4
2427	LOC_Os05g36990.1	5
2427	LOC_Os08g01190.1	8
2428	LOC_Os01g14260.1	1
2428	LOC_Os05g09570.1	5
2428	LOC_Os07g22270.1	7
2428	LOC_Os07g47610.1	7
2429	LOC_Os05g36910.1	5

2429	LOC_Os05g24560.1	5
2429	LOC_Os08g43350.1	8
2429	LOC_Os09g35810.1	9
2430	LOC_Os01g10140.1	1
2430	LOC_Os01g34350.1	1
2430	LOC_Os02g50330.1	2
2430	LOC_Os04g39160.1	4
2431	LOC_Os01g68260.1	1
2431	LOC_Os01g73970.1	1
2431	LOC_Os02g19510.1	2
2431	LOC_Os05g01760.1	5
2432	LOC_Os03g22270.1	3
2432	LOC_Os08g35190.1	8
2432	LOC_Os09g26620.1	9
2432	LOC_Os11g44810.1	11
2433	LOC_Os01g48190.1	1
2433	LOC_Os01g73960.1	1
2433	LOC_Os05g01730.1	5
2433	LOC_Os05g48800.1	5
2434	LOC_Os02g01930.1	2
2434	LOC_Os04g59140.1	4
2434	LOC_Os11g06440.1	11
2434	LOC_Os12g06780.1	12
2435	LOC_Os02g50010.1	2
2435	LOC_Os04g52520.1	4
2435	LOC_Os04g53612.1	4
2435	LOC_Os07g40920.1	7
2436	LOC_Os01g10610.1	1
2436	LOC_Os02g13900.1	2
2436	LOC_Os06g35900.1	6
2436	LOC_Os07g39220.1	7
2437	LOC_Os03g63000.1	3
2437	LOC_Os07g41670.1	7
2437	LOC_Os11g38420.1	11
2437	LOC_Os12g42940.1	12
2438	LOC_Os02g14150.1	2
2438	LOC_Os10g28180.1	10
2438	LOC_Os11g41110.1	11
2438	LOC_Os12g37830.1	12
2439	LOC_Os02g04110.1	2
2439	LOC_Os06g11040.1	6
2439	LOC_Os06g51390.1	6
2439	LOC_Os11g40070.1	11
2440	LOC_Os01g14810.1	1
2440	LOC_Os03g56310.1	3
2440	LOC_Os09g25640.1	9
2440	LOC_Os12g08000.1	12
2441	LOC_Os04g11850.1	4
2441	LOC_Os05g23690.1	5
2441	LOC_Os07g15160.1	7
2441	LOC_Os09g04980.1	9
2442	LOC_Os01g08890.1	1

2442	LOC_Os08g24450.1	8
2442	LOC_Os11g13920.1	11
2442	LOC_Os11g40920.1	11
2443	LOC_Os01g43980.1	1
2443	LOC_Os08g18080.1	8
2443	LOC_Os10g31990.1	10
2443	LOC_Os11g38300.1	11
2444	LOC_Os02g44350.1	2
2444	LOC_Os05g14380.1	5
2444	LOC_Os06g09490.1	6
2444	LOC_Os06g22500.1	6
2445	LOC_Os06g04010.1	6
2445	LOC_Os10g02509.1	10
2445	LOC_Os10g02584.1	10
2445	LOC_Os10g02620.1	10
2446	LOC_Os01g53790.1	1
2446	LOC_Os03g56220.1	3
2446	LOC_Os07g04150.1	7
2446	LOC_Os08g06640.1	8
2447	LOC_Os01g61010.1	1
2447	LOC_Os06g08110.1	6
2447	LOC_Os08g15450.1	8
2447	LOC_Os09g36600.1	9
2448	LOC_Os01g61970.1	1
2448	LOC_Os02g40550.1	2
2448	LOC_Os04g42960.1	4
2448	LOC_Os05g38720.1	5
2449	LOC_Os02g10690.1	2
2449	LOC_Os06g40450.1	6
2449	LOC_Os07g08790.1	7
2449	LOC_Os11g38010.1	11
2450	LOC_Os01g47430.1	1
2450	LOC_Os05g49180.1	5
2450	LOC_Os06g10430.1	6
2450	LOC_Os09g08670.1	9
2451	LOC_Os01g48874.1	1
2451	LOC_Os01g56360.1	1
2451	LOC_Os01g56370.1	1
2451	LOC_Os05g48260.1	5
2452	LOC_Os01g73000.1	1
2452	LOC_Os01g68060.1	1
2452	LOC_Os08g38600.1	8
2452	LOC_Os12g19030.1	12
2453	LOC_Os03g29680.1	3
2453	LOC_Os08g27860.1	8
2453	LOC_Os08g27870.1	8
2453	LOC_Os11g40610.1	11
2454	LOC_Os03g52910.1	3
2454	LOC_Os06g49640.1	6
2454	LOC_Os07g46320.1	7
2454	LOC_Os07g46330.1	7
2455	LOC_Os06g21210.1	6

2455	LOC_Os06g21220.1	6
2455	LOC_Os06g21230.1	6
2455	LOC_Os06g21240.1	6
2456	LOC_Os01g48540.1	1
2456	LOC_Os03g15320.1	3
2456	LOC_Os05g48520.1	5
2456	LOC_Os11g06870.1	11
2457	LOC_Os03g22580.1	3
2457	LOC_Os05g30790.1	5
2457	LOC_Os06g41710.1	6
2457	LOC_Os07g47360.1	7
2458	LOC_Os01g16220.1	1
2458	LOC_Os01g41600.1	1
2458	LOC_Os01g65520.1	1
2458	LOC_Os05g18770.1	5
2459	LOC_Os03g23900.1	3
2459	LOC_Os03g23920.1	3
2459	LOC_Os03g54190.1	3
2459	LOC_Os03g58610.1	3
2460	LOC_Os01g14220.1	1
2460	LOC_Os01g14310.1	1
2460	LOC_Os01g14320.1	1
2460	LOC_Os01g14340.1	1
2461	LOC_Os11g07360.1	11
2461	LOC_Os11g07380.1	11
2461	LOC_Os11g07320.1	11
2461	LOC_Os11g07330.1	11
2462	LOC_Os01g11980.1	1
2462	LOC_Os02g35640.1	2
2462	LOC_Os06g19620.1	6
2462	LOC_Os06g40630.1	6
2463	LOC_Os01g14690.1	1
2463	LOC_Os01g19940.1	1
2463	LOC_Os01g47490.1	1
2463	LOC_Os05g49160.1	5
2464	LOC_Os04g37680.1	4
2464	LOC_Os07g34170.1	7
2464	LOC_Os09g26490.1	9
2464	LOC_Os09g28720.1	9
2465	LOC_Os03g02020.1	3
2465	LOC_Os07g41810.1	7
2465	LOC_Os07g41820.1	7
2465	LOC_Os11g05290.1	11
2466	LOC_Os03g28120.1	3
2466	LOC_Os03g54100.1	3
2466	LOC_Os07g01810.1	7
2466	LOC_Os09g12790.1	9
2467	LOC_Os11g03770.1	11
2467	LOC_Os11g03740.1	11
2467	LOC_Os12g03490.1	12
2467	LOC_Os12g03510.1	12
2468	LOC_Os01g44310.1	1

2468	LOC_Os03g10180.1	3
2468	LOC_Os05g50370.1	5
2468	LOC_Os11g43950.1	11
2469	LOC_Os01g31940.1	1
2469	LOC_Os01g48800.1	1
2469	LOC_Os05g48300.1	5
2469	LOC_Os09g29239.1	9
2470	LOC_Os01g28100.1	1
2470	LOC_Os01g28120.1	1
2470	LOC_Os07g37660.1	7
2470	LOC_Os09g04370.1	9
2471	LOC_Os02g28580.1	2
2471	LOC_Os02g28660.1	2
2471	LOC_Os02g28680.1	2
2471	LOC_Os10g30890.1	10
2472	LOC_Os02g06010.1	2
2472	LOC_Os03g11590.1	3
2472	LOC_Os08g10350.1	8
2472	LOC_Os09g14520.1	9
2473	LOC_Os04g32380.1	4
2473	LOC_Os04g42230.1	4
2473	LOC_Os08g42790.1	8
2473	LOC_Os08g42880.1	8
2474	LOC_Os01g18020.1	1
2474	LOC_Os05g17660.1	5
2474	LOC_Os11g40310.1	11
2474	LOC_Os12g18680.1	12
2475	LOC_Os02g18220.1	2
2475	LOC_Os02g18250.1	2
2475	LOC_Os04g13940.1	4
2475	LOC_Os09g15910.1	9
2476	LOC_Os01g68780.1	1
2476	LOC_Os04g44140.1	4
2476	LOC_Os09g37940.1	9
2476	LOC_Os11g10530.1	11
2477	LOC_Os04g50188.1	4
2477	LOC_Os04g56420.1	4
2477	LOC_Os07g48590.1	7
2477	LOC_Os08g09980.1	8
2478	LOC_Os01g68930.1	1
2478	LOC_Os02g53280.1	2
2478	LOC_Os08g36380.1	8
2478	LOC_Os09g27590.1	9
2479	LOC_Os02g53440.1	2
2479	LOC_Os02g53480.1	2
2479	LOC_Os07g43490.1	7
2479	LOC_Os07g43500.1	7
2480	LOC_Os02g37750.1	2
2480	LOC_Os02g37780.1	2
2480	LOC_Os10g08170.1	10
2480	LOC_Os10g08050.1	10
2481	LOC_Os05g05330.1	5

2481	LOC_Os05g05530.1	5
2481	LOC_Os05g05354.1	5
2481	LOC_Os05g05390.1	5
2482	LOC_Os05g09720.1	5
2482	LOC_Os08g34920.1	8
2482	LOC_Os08g36830.1	8
2482	LOC_Os11g09050.1	11
2483	LOC_Os03g24100.1	3
2483	LOC_Os05g31970.1	5
2483	LOC_Os07g46510.1	7
2483	LOC_Os11g33050.1	11
2484	LOC_Os02g52744.1	2
2484	LOC_Os04g48370.1	4
2484	LOC_Os08g21700.1	8
2484	LOC_Os09g02440.1	9
2485	LOC_Os01g21590.1	1
2485	LOC_Os02g33770.1	2
2485	LOC_Os11g06410.1	11
2485	LOC_Os12g06640.1	12
2486	LOC_Os02g20880.1	2
2486	LOC_Os03g03110.1	3
2486	LOC_Os03g28070.1	3
2486	LOC_Os05g26120.1	5
2487	LOC_Os01g14550.1	1
2487	LOC_Os01g14590.1	1
2487	LOC_Os01g53090.1	1
2487	LOC_Os01g53110.1	1
2488	LOC_Os03g04830.1	3
2488	LOC_Os08g03840.1	8
2488	LOC_Os08g03990.1	8
2488	LOC_Os08g04090.1	8
2489	LOC_Os02g20330.1	2
2489	LOC_Os08g42110.1	8
2489	LOC_Os08g42210.1	8
2489	LOC_Os09g10000.1	9
2490	LOC_Os06g02630.1	6
2490	LOC_Os06g02800.1	6
2490	LOC_Os06g03059.1	6
2490	LOC_Os06g03099.1	6
2491	LOC_Os01g16630.1	1
2491	LOC_Os01g18490.1	1
2491	LOC_Os01g18530.1	1
2491	LOC_Os02g37070.1	2
2492	LOC_Os03g43309.1	3
2492	LOC_Os06g17080.1	6
2492	LOC_Os07g34100.1	7
2492	LOC_Os10g31610.1	10
2493	LOC_Os03g04710.1	3
2493	LOC_Os04g43160.1	4
2493	LOC_Os05g37520.1	5
2493	LOC_Os10g42610.1	10
2494	LOC_Os02g30974.1	2

2494	LOC_Os02g31830.1	2
2494	LOC_Os02g32280.1	2
2494	LOC_Os02g32290.1	2
2495	LOC_Os01g44030.1	1
2495	LOC_Os01g62740.1	1
2495	LOC_Os03g63880.1	3
2495	LOC_Os12g35470.1	12
2496	LOC_Os01g59950.1	1
2496	LOC_Os03g10160.1	3
2496	LOC_Os05g32670.1	5
2496	LOC_Os05g40950.1	5
2497	LOC_Os02g11870.1	2
2497	LOC_Os02g50940.1	2
2497	LOC_Os06g12660.1	6
2497	LOC_Os08g19670.1	8
2498	LOC_Os03g18470.1	3
2498	LOC_Os05g15600.1	5
2498	LOC_Os06g47630.1	6
2498	LOC_Os06g47870.1	6
2499	LOC_Os01g01810.1	1
2499	LOC_Os04g21610.1	4
2499	LOC_Os06g42040.1	6
2499	LOC_Os12g25810.1	12
2500	LOC_Os01g22289.1	1
2500	LOC_Os01g22309.1	1
2500	LOC_Os04g02940.1	4
2500	LOC_Os08g24090.1	8
2501	LOC_Os04g58320.1	4
2501	LOC_Os07g17400.1	7
2501	LOC_Os10g30850.1	10
2501	LOC_Os10g35670.1	10
2502	LOC_Os03g29370.1	3
2502	LOC_Os06g49600.1	6
2502	LOC_Os09g03000.1	9
2502	LOC_Os10g21150.1	10
2503	LOC_Os05g23060.1	5
2503	LOC_Os11g13950.1	11
2503	LOC_Os11g36330.1	11
2503	LOC_Os12g28010.1	12
2504	LOC_Os03g56690.1	3
2504	LOC_Os03g57360.1	3
2504	LOC_Os03g57400.1	3
2504	LOC_Os03g59880.1	3
2505	LOC_Os04g24319.1	4
2505	LOC_Os04g24328.1	4
2505	LOC_Os04g24478.1	4
2505	LOC_Os04g24469.1	4
2506	LOC_Os01g28820.1	1
2506	LOC_Os04g19010.1	4
2506	LOC_Os09g12440.1	9
2506	LOC_Os12g18430.1	12
2507	LOC_Os08g27430.1	8

2507	LOC_Os09g39880.1	9
2507	LOC_Os10g01840.1	10
2507	LOC_Os10g09180.1	10
2508	LOC_Os01g26852.1	1
2508	LOC_Os01g26832.1	1
2508	LOC_Os01g26920.1	1
2508	LOC_Os01g26970.1	1
2509	LOC_Os02g43550.1	2
2509	LOC_Os04g46000.1	4
2509	LOC_Os05g25950.1	5
2509	LOC_Os07g12780.1	7
2510	LOC_Os02g42020.1	2
2510	LOC_Os04g57700.1	4
2510	LOC_Os05g24000.1	5
2510	LOC_Os08g01080.1	8
2511	LOC_Os01g61760.1	1
2511	LOC_Os05g08380.1	5
2511	LOC_Os05g43870.1	5
2511	LOC_Os12g35080.1	12
2512	LOC_Os03g25310.1	3
2512	LOC_Os05g10750.1	5
2512	LOC_Os06g23960.1	6
2512	LOC_Os10g20300.1	10
2513	LOC_Os01g71020.1	1
2513	LOC_Os02g41530.1	2
2513	LOC_Os05g32690.1	5
2513	LOC_Os09g02200.1	9
2514	LOC_Os07g13380.1	7
2514	LOC_Os07g13440.1	7
2514	LOC_Os07g13450.1	7
2514	LOC_Os07g13310.1	7
2515	LOC_Os03g08360.1	3
2515	LOC_Os09g19650.1	9
2515	LOC_Os09g34930.1	9
2515	LOC_Os10g33370.1	10
2516	LOC_Os02g07860.1	2
2516	LOC_Os02g32050.1	2
2516	LOC_Os05g50030.1	5
2516	LOC_Os08g09440.1	8
2517	LOC_Os01g12100.1	1
2517	LOC_Os02g43960.1	2
2517	LOC_Os05g16220.1	5
2517	LOC_Os08g39330.1	8
2518	LOC_Os03g44600.1	3
2518	LOC_Os05g41050.1	5
2518	LOC_Os07g39450.1	7
2518	LOC_Os08g34100.1	8
2519	LOC_Os03g53270.1	3
2519	LOC_Os03g58170.1	3
2519	LOC_Os11g05050.1	11
2519	LOC_Os12g05050.1	12
2520	LOC_Os01g55870.1	1

2520	LOC_Os02g08410.1	2
2520	LOC_Os08g34290.1	8
2520	LOC_Os12g38900.1	12
2521	LOC_Os02g40170.1	2
2521	LOC_Os02g40150.1	2
2521	LOC_Os06g02990.1	6
2521	LOC_Os06g03010.1	6
2522	LOC_Os03g49070.1	3
2522	LOC_Os05g49630.1	5
2522	LOC_Os05g49640.1	5
2522	LOC_Os07g09850.1	7
2523	LOC_Os03g57610.1	3
2523	LOC_Os04g43100.1	4
2523	LOC_Os07g45430.1	7
2523	LOC_Os08g27490.1	8
2524	LOC_Os05g31090.1	5
2524	LOC_Os05g22890.1	5
2524	LOC_Os05g48180.1	5
2524	LOC_Os11g40330.1	11
2525	LOC_Os02g57750.1	2
2525	LOC_Os07g31650.1	7
2525	LOC_Os07g45350.1	7
2525	LOC_Os09g39690.1	9
2526	LOC_Os02g26320.1	2
2526	LOC_Os02g49420.1	2
2526	LOC_Os04g21570.1	4
2526	LOC_Os06g44660.1	6
2527	LOC_Os06g04220.1	6
2527	LOC_Os06g04230.1	6
2527	LOC_Os06g04210.1	6
2527	LOC_Os06g04240.1	6
2528	LOC_Os04g52420.1	4
2528	LOC_Os04g52660.1	4
2528	LOC_Os07g10120.1	7
2528	LOC_Os07g10030.1	7
2529	LOC_Os03g47480.1	3
2529	LOC_Os05g19980.1	5
2529	LOC_Os11g18740.1	11
2529	LOC_Os11g24680.1	11
2530	LOC_Os04g27620.1	4
2530	LOC_Os07g26420.1	7
2530	LOC_Os11g22660.1	11
2530	LOC_Os12g09390.1	12
2531	LOC_Os02g45000.1	2
2531	LOC_Os07g13830.1	7
2531	LOC_Os07g40160.1	7
2531	LOC_Os10g37580.1	10
2532	LOC_Os01g43110.1	1
2532	LOC_Os05g14900.1	5
2532	LOC_Os11g09100.1	11
2532	LOC_Os12g36270.1	12
2533	LOC_Os12g18729.1	12

2533	LOC_Os12g26410.1	12
2533	LOC_Os12g36340.1	12
2533	LOC_Os12g36420.1	12
2534	LOC_Os01g12150.1	1
2534	LOC_Os04g24130.1	4
2534	LOC_Os06g19250.1	6
2534	LOC_Os08g29320.1	8
2535	LOC_Os01g59480.1	1
2535	LOC_Os01g59710.1	1
2535	LOC_Os01g59720.1	1
2535	LOC_Os08g18110.1	8
2536	LOC_Os01g60760.1	1
2536	LOC_Os06g01190.1	6
2536	LOC_Os06g41650.1	6
2536	LOC_Os06g42840.1	6
2537	LOC_Os06g30250.1	6
2537	LOC_Os09g01610.1	9
2537	LOC_Os09g01470.1	9
2537	LOC_Os09g03990.1	9
2538	LOC_Os01g70480.1	1
2538	LOC_Os07g22760.1	7
2538	LOC_Os12g31630.1	12
2538	LOC_Os12g33140.1	12
2539	LOC_Os01g61680.1	1
2539	LOC_Os03g04370.1	3
2539	LOC_Os05g03590.1	5
2539	LOC_Os05g39060.1	5
2540	LOC_Os02g36500.1	2
2540	LOC_Os03g27010.1	3
2540	LOC_Os04g38310.1	4
2540	LOC_Os07g43070.1	7
2541	LOC_Os05g15190.1	5
2541	LOC_Os06g34564.1	6
2541	LOC_Os09g13420.1	9
2541	LOC_Os12g25840.1	12
2542	LOC_Os01g65970.1	1
2542	LOC_Os01g06560.1	1
2542	LOC_Os05g48650.1	5
2542	LOC_Os10g41640.1	10
2543	LOC_Os03g62160.1	3
2543	LOC_Os07g10810.1	7
2543	LOC_Os07g15280.1	7
2543	LOC_Os07g28940.1	7
2544	LOC_Os01g55710.1	1
2544	LOC_Os03g21570.1	3
2544	LOC_Os03g21700.1	3
2544	LOC_Os03g45730.1	3
2545	LOC_Os03g43560.1	3
2545	LOC_Os04g40690.1	4
2545	LOC_Os05g03700.1	5
2545	LOC_Os12g24010.1	12
2546	LOC_Os02g56260.1	2

2546	LOC_Os03g50830.1	3
2546	LOC_Os09g26440.1	9
2546	LOC_Os12g08300.1	12
2547	LOC_Os03g52360.1	3
2547	LOC_Os03g52370.1	3
2547	LOC_Os03g52380.1	3
2547	LOC_Os03g52390.1	3
2548	LOC_Os01g18220.1	1
2548	LOC_Os05g04830.1	5
2548	LOC_Os11g17930.1	11
2548	LOC_Os12g12880.1	12
2549	LOC_Os01g52380.1	1
2549	LOC_Os07g02610.1	7
2549	LOC_Os07g02630.1	7
2549	LOC_Os07g02590.1	7
2550	LOC_Os12g33939.1	12
2550	LOC_Os12g34079.1	12
2550	LOC_Os12g34149.1	12
2550	LOC_Os12g34080.1	12
2551	LOC_Os07g39550.1	7
2551	LOC_Os08g38950.1	8
2551	LOC_Os08g45090.1	8
2551	LOC_Os11g11310.1	11
2552	LOC_Os02g12700.1	2
2552	LOC_Os02g12710.1	2
2552	LOC_Os02g12720.1	2
2552	LOC_Os08g38340.1	8
2553	LOC_Os04g24804.1	4
2553	LOC_Os08g24850.1	8
2553	LOC_Os10g09260.1	10
2553	LOC_Os10g33190.1	10
2554	LOC_Os01g22039.1	1
2554	LOC_Os01g22209.1	1
2554	LOC_Os03g62522.1	3
2554	LOC_Os03g62539.1	3
2555	LOC_Os02g10730.1	2
2555	LOC_Os06g40210.1	6
2555	LOC_Os12g33180.1	12
2555	LOC_Os12g36740.1	12
2556	LOC_Os02g09980.1	2
2556	LOC_Os02g09990.1	2
2556	LOC_Os03g52940.1	3
2556	LOC_Os09g32000.1	9
2557	LOC_Os01g05150.1	1
2557	LOC_Os01g05180.1	1
2557	LOC_Os01g05360.1	1
2557	LOC_Os01g08940.1	1
2558	LOC_Os03g22230.1	3
2558	LOC_Os03g22210.1	3
2558	LOC_Os07g47750.1	7
2558	LOC_Os07g47760.1	7
2559	LOC_Os08g39000.1	8

2559	LOC_Os09g27000.1	9
2559	LOC_Os10g18070.1	10
2559	LOC_Os11g38380.1	11
2560	LOC_Os01g64420.1	1
2560	LOC_Os02g05990.1	2
2560	LOC_Os08g33450.1	8
2560	LOC_Os09g23670.1	9
2561	LOC_Os03g25840.1	3
2561	LOC_Os03g25869.1	3
2561	LOC_Os08g41370.1	8
2561	LOC_Os12g39080.1	12
2562	LOC_Os01g50259.1	1
2562	LOC_Os01g50280.1	1
2562	LOC_Os01g50290.1	1
2562	LOC_Os01g50300.1	1
2563	LOC_Os01g38750.1	1
2563	LOC_Os03g19030.1	3
2563	LOC_Os05g37810.1	5
2563	LOC_Os09g16280.1	9
2564	LOC_Os03g44130.1	3
2564	LOC_Os07g02500.1	7
2564	LOC_Os07g10950.1	7
2564	LOC_Os10g33030.1	10
2565	LOC_Os03g21970.1	3
2565	LOC_Os05g27090.1	5
2565	LOC_Os07g47960.1	7
2565	LOC_Os11g02054.1	11
2566	LOC_Os01g13670.1	1
2566	LOC_Os03g55880.1	3
2566	LOC_Os06g14770.1	6
2566	LOC_Os09g31990.1	9
2567	LOC_Os02g32390.1	2
2567	LOC_Os07g45140.1	7
2567	LOC_Os08g23350.1	8
2567	LOC_Os10g38680.1	10
2568	LOC_Os01g55370.1	1
2568	LOC_Os03g18660.1	3
2568	LOC_Os05g07240.1	5
2568	LOC_Os05g39710.1	5
2569	LOC_Os03g22320.1	3
2569	LOC_Os04g40430.1	4
2569	LOC_Os04g54430.1	4
2569	LOC_Os08g05540.1	8
2570	LOC_Os02g35830.1	2
2570	LOC_Os04g37520.1	4
2570	LOC_Os06g42850.1	6
2570	LOC_Os08g03430.1	8
2571	LOC_Os01g49730.1	1
2571	LOC_Os08g11580.1	8
2571	LOC_Os08g12030.1	8
2571	LOC_Os09g24630.1	9
2572	LOC_Os08g38530.1	8

2572	LOC_Os10g08460.1	10
2572	LOC_Os11g29470.1	11
2572	LOC_Os11g30820.1	11
2573	LOC_Os02g33330.1	2
2573	LOC_Os04g33860.1	4
2573	LOC_Os05g37030.1	5
2573	LOC_Os12g06130.1	12
2574	LOC_Os03g21850.1	3
2574	LOC_Os07g48170.1	7
2574	LOC_Os11g02320.1	11
2574	LOC_Os12g02260.1	12
2575	LOC_Os01g57550.1	1
2575	LOC_Os03g24910.1	3
2575	LOC_Os03g27760.1	3
2575	LOC_Os07g44960.1	7
2576	LOC_Os02g17030.1	2
2576	LOC_Os02g49910.1	2
2576	LOC_Os11g38370.1	11
2576	LOC_Os12g37680.1	12
2577	LOC_Os04g06840.1	4
2577	LOC_Os04g07040.1	4
2577	LOC_Os04g06810.1	4
2577	LOC_Os04g06930.1	4
2578	LOC_Os02g16220.1	2
2578	LOC_Os06g13750.1	6
2578	LOC_Os06g13890.1	6
2578	LOC_Os09g01790.1	9
2579	LOC_Os02g05290.1	2
2579	LOC_Os02g15420.1	2
2579	LOC_Os08g15390.1	8
2579	LOC_Os09g10700.1	9
2580	LOC_Os02g06390.1	2
2580	LOC_Os03g53970.1	3
2580	LOC_Os05g46920.1	5
2580	LOC_Os07g02720.1	7
2581	LOC_Os07g17980.1	7
2581	LOC_Os10g15290.1	10
2581	LOC_Os10g17220.1	10
2581	LOC_Os10g24410.1	10
2582	LOC_Os02g34120.1	2
2582	LOC_Os07g11770.1	7
2582	LOC_Os09g16780.1	9
2582	LOC_Os12g18450.1	12
2583	LOC_Os07g45130.1	7
2583	LOC_Os07g45234.1	7
2583	LOC_Os12g38540.1	12
2583	LOC_Os12g38550.1	12
2584	LOC_Os08g13010.1	8
2584	LOC_Os08g30030.1	8
2584	LOC_Os10g10400.1	10
2584	LOC_Os12g03380.1	12
2585	LOC_Os02g22670.1	2

2585	LOC_Os03g33012.1	3
2585	LOC_Os10g10010.1	10
2585	LOC_Os12g25670.1	12
2586	LOC_Os02g09330.1	2
2586	LOC_Os03g40610.1	3
2586	LOC_Os03g53060.1	3
2586	LOC_Os10g09100.1	10
2587	LOC_Os01g07890.1	1
2587	LOC_Os01g50870.1	1
2587	LOC_Os05g08390.1	5
2587	LOC_Os05g46500.1	5
2588	LOC_Os02g44890.1	2
2588	LOC_Os04g59340.1	4
2588	LOC_Os07g15270.1	7
2588	LOC_Os09g02400.1	9
2589	LOC_Os02g49960.1	2
2589	LOC_Os06g14780.1	6
2589	LOC_Os08g33210.1	8
2589	LOC_Os09g23780.1	9
2590	LOC_Os01g42130.1	1
2590	LOC_Os03g27850.1	3
2590	LOC_Os08g36820.1	8
2590	LOC_Os09g28340.1	9
2591	LOC_Os07g10360.1	7
2591	LOC_Os07g10310.1	7
2591	LOC_Os07g10330.1	7
2591	LOC_Os07g10370.1	7
2592	LOC_Os01g12000.1	1
2592	LOC_Os01g48950.1	1
2592	LOC_Os02g44720.1	2
2592	LOC_Os05g12120.1	5
2593	LOC_Os01g05000.1	1
2593	LOC_Os05g40340.1	5
2593	LOC_Os05g40350.1	5
2593	LOC_Os05g40360.1	5
2594	LOC_Os06g39290.1	6
2594	LOC_Os07g40370.1	7
2594	LOC_Os11g16650.1	11
2594	LOC_Os12g12630.1	12
2595	LOC_Os01g60200.1	1
2595	LOC_Os02g54090.1	2
2595	LOC_Os05g40410.1	5
2595	LOC_Os12g31440.1	12
2596	LOC_Os01g43330.1	1
2596	LOC_Os02g15820.1	2
2596	LOC_Os05g50910.1	5
2596	LOC_Os06g33320.1	6
2597	LOC_Os01g72630.1	1
2597	LOC_Os01g73080.1	1
2597	LOC_Os01g73800.1	1
2597	LOC_Os05g08090.1	5
2598	LOC_Os01g24150.1	1

2598	LOC_Os04g47920.1	4
2598	LOC_Os11g26700.1	11
2598	LOC_Os12g26930.1	12
2599	LOC_Os09g21080.1	9
2599	LOC_Os09g21090.1	9
2599	LOC_Os09g25270.1	9
2599	LOC_Os11g10590.1	11
2600	LOC_Os01g19320.1	1
2600	LOC_Os04g47950.1	4
2600	LOC_Os07g41210.1	7
2600	LOC_Os09g26600.1	9
2601	LOC_Os10g03770.1	10
2601	LOC_Os10g28110.1	10
2601	LOC_Os11g11040.1	11
2601	LOC_Os12g15430.1	12
2602	LOC_Os01g46650.1	1
2602	LOC_Os07g14480.1	7
2602	LOC_Os08g36590.1	8
2602	LOC_Os09g21270.1	9
2603	LOC_Os04g07050.1	4
2603	LOC_Os04g07130.1	4
2603	LOC_Os04g07260.1	4
2603	LOC_Os04g07920.1	4
2604	LOC_Os06g38740.1	6
2604	LOC_Os11g07530.1	11
2604	LOC_Os11g07540.1	11
2604	LOC_Os11g07550.1	11
2605	LOC_Os02g03710.1	2
2605	LOC_Os08g32370.1	8
2605	LOC_Os10g36610.1	10
2605	LOC_Os10g36580.1	10
2606	LOC_Os06g28890.1	6
2606	LOC_Os08g28370.1	8
2606	LOC_Os11g45120.1	11
2606	LOC_Os12g22660.1	12
2607	LOC_Os01g13050.1	1
2607	LOC_Os01g64230.1	1
2607	LOC_Os03g19070.1	3
2607	LOC_Os11g04580.1	11
2608	LOC_Os02g48640.1	2
2608	LOC_Os08g24434.1	8
2608	LOC_Os09g10950.1	9
2608	LOC_Os09g31360.1	9
2609	LOC_Os02g03230.1	2
2609	LOC_Os02g26860.1	2
2609	LOC_Os04g02910.1	4
2609	LOC_Os07g08760.1	7
2610	LOC_Os02g17450.1	2
2610	LOC_Os04g58430.1	4
2610	LOC_Os05g48920.1	5
2610	LOC_Os08g16490.1	8
2611	LOC_Os01g29570.1	1

2611	LOC_Os08g42330.1	8
2611	LOC_Os08g42340.1	8
2611	LOC_Os11g02810.1	11
2612	LOC_Os01g58230.1	1
2612	LOC_Os07g06166.1	7
2612	LOC_Os07g06250.1	7
2612	LOC_Os07g06345.1	7
2613	LOC_Os08g07630.1	8
2613	LOC_Os08g07640.1	8
2613	LOC_Os08g07660.1	8
2613	LOC_Os08g07670.1	8
2614	LOC_Os02g29850.1	2
2614	LOC_Os06g26190.1	6
2614	LOC_Os12g22530.1	12
2614	LOC_Os12g28210.1	12
2615	LOC_Os03g52410.1	3
2615	LOC_Os03g58510.1	3
2615	LOC_Os10g28160.1	10
2615	LOC_Os10g28170.1	10
2616	LOC_Os01g71220.1	1
2616	LOC_Os02g52910.1	2
2616	LOC_Os05g30990.1	5
2616	LOC_Os09g28110.1	9
2617	LOC_Os02g22340.1	2
2617	LOC_Os04g14070.1	4
2617	LOC_Os04g20150.1	4
2617	LOC_Os12g19510.1	12
2618	LOC_Os01g48610.1	1
2618	LOC_Os01g48620.1	1
2618	LOC_Os01g48640.1	1
2618	LOC_Os01g48660.1	1
2619	LOC_Os01g52900.1	1
2619	LOC_Os11g10430.1	11
2619	LOC_Os12g08850.1	12
2619	LOC_Os12g08900.1	12
2620	LOC_Os07g24820.1	7
2620	LOC_Os07g24830.1	7
2620	LOC_Os07g25050.1	7
2620	LOC_Os07g25060.1	7
2621	LOC_Os05g05340.1	5
2621	LOC_Os05g05440.1	5
2621	LOC_Os05g05370.1	5
2621	LOC_Os08g05080.1	8
2622	LOC_Os04g03860.1	4
2622	LOC_Os08g45080.1	8
2622	LOC_Os12g14140.1	12
2622	LOC_Os12g14150.1	12
2623	LOC_Os03g55450.1	3
2623	LOC_Os04g21650.1	4
2623	LOC_Os04g56300.1	4
2623	LOC_Os12g39090.1	12
2624	LOC_Os08g04710.1	8

2624	LOC_Os08g04740.1	8
2624	LOC_Os08g04640.1	8
2624	LOC_Os08g04680.1	8
2625	LOC_Os02g43560.1	2
2625	LOC_Os05g45230.1	5
2625	LOC_Os06g36380.1	6
2625	LOC_Os11g18750.1	11
2626	LOC_Os02g36000.1	2
2626	LOC_Os02g44090.1	2
2626	LOC_Os03g08840.1	3
2626	LOC_Os04g46600.1	4
2627	LOC_Os01g13930.1	1
2627	LOC_Os02g38860.1	2
2627	LOC_Os04g52880.1	4
2627	LOC_Os06g35770.1	6
2628	LOC_Os02g44420.1	2
2628	LOC_Os05g38780.1	5
2628	LOC_Os09g27120.1	9
2628	LOC_Os09g33840.1	9
2629	LOC_Os01g12080.1	1
2629	LOC_Os02g04450.1	2
2629	LOC_Os05g12170.1	5
2629	LOC_Os06g49180.1	6
2630	LOC_Os05g40730.1	5
2630	LOC_Os05g41360.1	5
2630	LOC_Os07g42104.1	7
2630	LOC_Os07g42130.1	7
2631	LOC_Os03g56350.1	3
2631	LOC_Os10g09890.1	10
2631	LOC_Os12g28128.1	12
2631	LOC_Os12g28168.1	12
2632	LOC_Os06g03030.1	6
2632	LOC_Os06g02940.1	6
2632	LOC_Os06g03070.1	6
2632	LOC_Os06g03110.1	6
2633	LOC_Os07g05020.1	7
2633	LOC_Os08g16870.1	8
2633	LOC_Os09g34260.1	9
2633	LOC_Os11g22650.1	11
2634	LOC_Os03g37360.1	3
2634	LOC_Os07g24140.1	7
2634	LOC_Os07g26050.1	7
2634	LOC_Os11g46110.1	11
2635	LOC_Os02g32190.1	2
2635	LOC_Os02g50710.1	2
2635	LOC_Os03g11780.1	3
2635	LOC_Os06g13190.1	6
2636	LOC_Os02g38430.1	2
2636	LOC_Os04g37670.1	4
2636	LOC_Os04g40700.1	4
2636	LOC_Os06g19630.1	6
2637	LOC_Os11g03180.1	11

2637	LOC_Os11g03190.1	11
2637	LOC_Os12g02930.1	12
2637	LOC_Os12g02940.1	12
2638	LOC_Os07g44760.1	7
2638	LOC_Os09g38460.1	9
2638	LOC_Os09g38470.1	9
2638	LOC_Os09g38490.1	9
2639	LOC_Os11g08680.1	11
2639	LOC_Os11g08930.1	11
2639	LOC_Os11g08860.1	11
2639	LOC_Os11g36230.1	11
2640	LOC_Os03g21270.1	3
2640	LOC_Os03g64320.1	3
2640	LOC_Os07g42410.1	7
2640	LOC_Os07g26440.1	7
2641	LOC_Os04g02470.1	4
2641	LOC_Os04g53840.1	4
2641	LOC_Os06g08250.1	6
2641	LOC_Os09g20100.1	9
2642	LOC_Os01g04510.1	1
2642	LOC_Os01g16560.1	1
2642	LOC_Os03g25410.1	3
2642	LOC_Os07g11560.1	7
2643	LOC_Os04g03884.1	4
2643	LOC_Os07g15910.1	7
2643	LOC_Os07g33300.1	7
2643	LOC_Os09g27630.1	9
2644	LOC_Os05g16420.1	5
2644	LOC_Os05g16920.1	5
2644	LOC_Os05g17800.1	5
2644	LOC_Os08g10130.1	8
2645	LOC_Os11g31430.1	11
2645	LOC_Os11g31470.1	11
2645	LOC_Os11g31770.1	11
2645	LOC_Os11g31820.1	11
2646	LOC_Os08g27240.1	8
2646	LOC_Os08g35050.1	8
2646	LOC_Os08g35000.1	8
2646	LOC_Os08g35010.1	8
2647	LOC_Os03g24090.1	3
2647	LOC_Os04g48470.1	4
2647	LOC_Os11g34330.1	11
2647	LOC_Os12g08680.1	12
2648	LOC_Os03g38680.1	3
2648	LOC_Os07g01900.1	7
2648	LOC_Os07g02250.1	7
2648	LOC_Os07g01990.1	7
2649	LOC_Os08g29610.1	8
2649	LOC_Os09g02910.1	9
2649	LOC_Os10g10140.1	10
2649	LOC_Os12g34660.1	12
2650	LOC_Os02g15210.1	2

2650	LOC_Os04g03010.1	4
2650	LOC_Os09g14130.1	9
2650	LOC_Os11g05250.1	11
2651	LOC_Os03g38700.1	3
2651	LOC_Os04g22660.1	4
2651	LOC_Os09g14570.1	9
2651	LOC_Os11g25100.1	11
2652	LOC_Os03g06610.1	3
2652	LOC_Os04g38470.1	4
2652	LOC_Os05g39880.1	5
2652	LOC_Os11g37190.1	11
2653	LOC_Os02g55540.1	2
2653	LOC_Os04g30180.1	4
2653	LOC_Os04g30230.1	4
2653	LOC_Os04g30320.1	4
2654	LOC_Os09g36050.1	9
2654	LOC_Os09g36380.1	9
2654	LOC_Os09g36430.1	9
2654	LOC_Os09g37750.1	9
2655	LOC_Os01g08690.1	1
2655	LOC_Os01g40690.1	1
2655	LOC_Os08g30730.1	8
2655	LOC_Os09g19640.1	9
2656	LOC_Os01g27710.1	1
2656	LOC_Os04g44660.1	4
2656	LOC_Os10g02270.1	10
2656	LOC_Os11g26150.1	11
2657	LOC_Os04g05460.1	4
2657	LOC_Os06g18970.1	6
2657	LOC_Os09g29180.1	9
2657	LOC_Os10g27100.1	10
2658	LOC_Os02g01380.1	2
2658	LOC_Os02g43300.1	2
2658	LOC_Os04g45750.1	4
2658	LOC_Os10g37240.1	10
2659	LOC_Os01g23430.1	1
2659	LOC_Os02g05270.1	2
2659	LOC_Os06g25570.1	6
2659	LOC_Os08g24960.1	8
2660	LOC_Os02g24290.1	2
2660	LOC_Os05g32030.1	5
2660	LOC_Os07g44980.1	7
2660	LOC_Os09g22400.1	9
2661	LOC_Os01g64560.1	1
2661	LOC_Os02g45170.1	2
2661	LOC_Os11g06010.1	11
2661	LOC_Os12g06330.1	12
2662	LOC_Os01g14330.1	1
2662	LOC_Os01g18810.1	1
2662	LOC_Os05g04540.1	5
2662	LOC_Os05g19620.1	5
2663	LOC_Os01g56300.1	1

2663	LOC_Os02g19220.1	2
2663	LOC_Os02g19330.1	2
2663	LOC_Os02g31220.1	2
2664	LOC_Os01g08620.1	1
2664	LOC_Os02g15720.1	2
2664	LOC_Os03g40380.1	3
2664	LOC_Os04g19310.1	4
2665	LOC_Os04g31350.1	4
2665	LOC_Os08g42630.1	8
2665	LOC_Os09g33640.1	9
2665	LOC_Os09g33650.1	9
2666	LOC_Os01g63050.1	1
2666	LOC_Os05g37930.1	5
2666	LOC_Os06g47430.1	6
2666	LOC_Os10g29700.1	10
2667	LOC_Os06g38790.1	6
2667	LOC_Os06g46860.1	6
2667	LOC_Os06g47020.1	6
2667	LOC_Os11g11620.1	11
2668	LOC_Os01g20170.1	1
2668	LOC_Os05g29160.1	5
2668	LOC_Os07g26160.1	7
2668	LOC_Os10g02640.1	10
2669	LOC_Os01g64740.1	1
2669	LOC_Os03g13060.1	3
2669	LOC_Os04g33130.1	4
2669	LOC_Os06g10060.1	6
2670	LOC_Os08g03820.1	8
2670	LOC_Os08g03760.1	8
2670	LOC_Os08g04030.1	8
2670	LOC_Os08g03900.1	8
2671	LOC_Os01g61950.1	1
2671	LOC_Os04g39540.1	4
2671	LOC_Os08g38890.1	8
2671	LOC_Os09g30350.1	9
2672	LOC_Os03g04760.1	3
2672	LOC_Os05g02720.1	5
2672	LOC_Os05g11370.1	5
2672	LOC_Os06g12170.1	6
2673	LOC_Os01g06690.1	1
2673	LOC_Os02g58760.1	2
2673	LOC_Os09g27290.1	9
2673	LOC_Os10g09140.1	10
2674	LOC_Os03g07540.1	3
2674	LOC_Os04g54900.1	4
2674	LOC_Os10g26460.1	10
2674	LOC_Os10g26410.1	10
2675	LOC_Os04g37760.1	4
2675	LOC_Os08g37720.1	8
2675	LOC_Os09g29380.1	9
2675	LOC_Os10g23060.1	10
2676	LOC_Os03g36760.1	3

2676	LOC_Os03g36830.1	3
2676	LOC_Os10g11820.1	10
2676	LOC_Os12g15450.1	12
2677	LOC_Os03g30430.1	3
2677	LOC_Os04g48870.1	4
2677	LOC_Os11g41150.1	11
2677	LOC_Os12g31780.1	12
2678	LOC_Os01g42380.1	1
2678	LOC_Os01g42410.1	1
2678	LOC_Os01g42350.1	1
2678	LOC_Os01g42370.1	1
2678	LOC_Os11g37700.1	11
2679	LOC_Os02g11960.1	2
2679	LOC_Os06g38950.1	6
2679	LOC_Os08g30770.1	8
2679	LOC_Os08g30780.1	8
2679	LOC_Os09g19680.1	9
2680	LOC_Os02g58020.1	2
2680	LOC_Os03g32630.1	3
2680	LOC_Os08g45010.1	8
2680	LOC_Os09g39910.1	9
2680	LOC_Os11g39020.1	11
2681	LOC_Os01g72370.1	1
2681	LOC_Os02g51320.1	2
2681	LOC_Os05g01256.1	5
2681	LOC_Os06g12210.1	6
2681	LOC_Os11g39000.1	11
2682	LOC_Os01g18630.1	1
2682	LOC_Os01g44130.1	1
2682	LOC_Os01g47410.1	1
2682	LOC_Os05g04630.1	5
2682	LOC_Os05g49200.1	5
2683	LOC_Os01g16430.1	1
2683	LOC_Os01g58890.1	1
2683	LOC_Os01g68660.1	1
2683	LOC_Os05g41460.1	5
2683	LOC_Os05g33880.1	5
2684	LOC_Os03g13140.1	3
2684	LOC_Os03g13150.1	3
2684	LOC_Os03g13160.1	3
2684	LOC_Os03g12510.1	3
2684	LOC_Os05g44140.1	5
2685	LOC_Os01g41810.1	1
2685	LOC_Os01g43750.1	1
2685	LOC_Os01g43774.1	1
2685	LOC_Os01g52790.1	1
2685	LOC_Os01g41820.1	1
2686	LOC_Os01g12760.1	1
2686	LOC_Os02g30080.1	2
2686	LOC_Os06g43320.1	6
2686	LOC_Os10g08540.1	10
2686	LOC_Os10g30380.1	10

2687	LOC_Os01g72760.1	1
2687	LOC_Os02g09190.1	2
2687	LOC_Os04g09920.1	4
2687	LOC_Os04g10160.1	4
2687	LOC_Os06g43420.1	6
2688	LOC_Os03g15250.1	3
2688	LOC_Os03g60810.1	3
2688	LOC_Os06g10790.1	6
2688	LOC_Os10g38960.1	10
2688	LOC_Os10g30530.1	10
2689	LOC_Os02g54510.1	2
2689	LOC_Os03g28300.1	3
2689	LOC_Os06g08280.1	6
2689	LOC_Os06g43840.1	6
2689	LOC_Os07g25680.1	7
2690	LOC_Os02g02780.1	2
2690	LOC_Os06g50920.1	6
2690	LOC_Os08g01270.1	8
2690	LOC_Os09g37230.1	9
2690	LOC_Os12g06670.1	12
2691	LOC_Os02g08240.1	2
2691	LOC_Os02g54900.1	2
2691	LOC_Os03g02320.1	3
2691	LOC_Os06g29120.1	6
2691	LOC_Os10g37480.1	10
2692	LOC_Os01g70410.1	1
2692	LOC_Os05g30820.1	5
2692	LOC_Os10g02360.1	10
2692	LOC_Os10g05160.1	10
2692	LOC_Os11g35260.1	11
2693	LOC_Os03g27280.1	3
2693	LOC_Os03g41460.1	3
2693	LOC_Os03g55600.1	3
2693	LOC_Os07g42940.1	7
2693	LOC_Os12g39630.1	12
2694	LOC_Os02g43740.1	2
2694	LOC_Os04g46180.1	4
2694	LOC_Os08g38320.1	8
2694	LOC_Os09g08420.1	9
2694	LOC_Os09g30150.1	9
2695	LOC_Os02g48290.1	2
2695	LOC_Os02g05680.1	2
2695	LOC_Os05g48200.1	5
2695	LOC_Os06g22140.1	6
2695	LOC_Os11g05970.1	11
2696	LOC_Os02g47790.1	2
2696	LOC_Os02g47800.1	2
2696	LOC_Os08g44340.1	8
2696	LOC_Os08g05570.1	8
2696	LOC_Os09g39380.1	9
2697	LOC_Os02g40510.1	2
2697	LOC_Os03g17570.1	3

2697	LOC_Os07g49460.1	7
2697	LOC_Os09g36220.1	9
2697	LOC_Os11g05930.1	11
2698	LOC_Os03g61110.1	3
2698	LOC_Os04g32340.1	4
2698	LOC_Os06g07350.1	6
2698	LOC_Os07g18050.1	7
2698	LOC_Os08g03310.1	8
2699	LOC_Os03g17030.1	3
2699	LOC_Os04g42600.1	4
2699	LOC_Os06g38980.1	6
2699	LOC_Os08g22354.1	8
2699	LOC_Os09g02700.1	9
2700	LOC_Os01g68000.1	1
2700	LOC_Os02g21430.1	2
2700	LOC_Os02g31290.1	2
2700	LOC_Os02g48790.1	2
2700	LOC_Os05g01240.1	5
2701	LOC_Os01g43170.1	1
2701	LOC_Os01g64770.1	1
2701	LOC_Os03g25980.1	3
2701	LOC_Os05g36120.1	5
2701	LOC_Os08g33830.1	8
2702	LOC_Os03g50560.1	3
2702	LOC_Os03g53770.1	3
2702	LOC_Os04g53330.1	4
2702	LOC_Os06g02240.1	6
2702	LOC_Os11g34680.1	11
2703	LOC_Os01g57650.1	1
2703	LOC_Os03g15790.1	3
2703	LOC_Os04g02510.1	4
2703	LOC_Os04g59380.1	4
2703	LOC_Os11g06840.1	11
2704	LOC_Os02g44130.1	2
2704	LOC_Os02g57790.1	2
2704	LOC_Os03g55540.1	3
2704	LOC_Os04g08060.1	4
2704	LOC_Os04g46680.1	4
2705	LOC_Os01g52110.1	1
2705	LOC_Os03g22680.1	3
2705	LOC_Os03g05270.1	3
2705	LOC_Os10g31850.1	10
2705	LOC_Os12g35320.1	12
2706	LOC_Os02g36300.1	2
2706	LOC_Os02g52210.1	2
2706	LOC_Os06g11450.1	6
2706	LOC_Os08g37760.1	8
2706	LOC_Os09g29310.1	9
2707	LOC_Os01g56070.1	1
2707	LOC_Os07g42610.1	7
2707	LOC_Os08g38060.1	8
2707	LOC_Os09g06770.1	9

2707	LOC_Os12g02210.1	12
2708	LOC_Os04g56880.1	4
2708	LOC_Os05g02270.1	5
2708	LOC_Os08g16760.1	8
2708	LOC_Os10g22230.1	10
2708	LOC_Os12g29900.1	12
2709	LOC_Os01g18110.1	1
2709	LOC_Os02g56460.1	2
2709	LOC_Os02g56680.1	2
2709	LOC_Os02g56720.1	2
2709	LOC_Os09g04050.1	9
2710	LOC_Os02g56690.1	2
2710	LOC_Os03g22780.1	3
2710	LOC_Os08g17500.1	8
2710	LOC_Os08g34280.1	8
2710	LOC_Os09g25150.1	9
2711	LOC_Os02g30690.1	2
2711	LOC_Os02g30714.1	2
2711	LOC_Os04g32070.1	4
2711	LOC_Os11g30560.1	11
2711	LOC_Os12g27830.1	12
2712	LOC_Os01g54940.1	1
2712	LOC_Os04g28990.1	4
2712	LOC_Os04g29030.1	4
2712	LOC_Os05g24880.1	5
2712	LOC_Os08g29170.1	8
2713	LOC_Os03g31550.1	3
2713	LOC_Os03g57680.1	3
2713	LOC_Os03g57690.1	3
2713	LOC_Os07g18120.1	7
2713	LOC_Os10g04860.1	10
2714	LOC_Os01g42780.1	1
2714	LOC_Os01g67980.1	1
2714	LOC_Os04g01710.1	4
2714	LOC_Os06g38450.1	6
2714	LOC_Os09g21370.1	9
2715	LOC_Os02g48450.1	2
2715	LOC_Os05g01810.1	5
2715	LOC_Os09g39060.1	9
2715	LOC_Os09g39170.1	9
2715	LOC_Os09g39110.1	9
2716	LOC_Os03g14450.1	3
2716	LOC_Os03g15950.1	3
2716	LOC_Os06g04510.1	6
2716	LOC_Os09g20820.1	9
2716	LOC_Os10g08550.1	10
2717	LOC_Os01g18070.1	1
2717	LOC_Os01g52400.1	1
2717	LOC_Os07g04200.1	7
2717	LOC_Os07g44840.1	7
2717	LOC_Os12g07220.1	12
2718	LOC_Os03g10650.1	3

2718	LOC_Os03g52750.1	3
2718	LOC_Os09g02360.1	9
2718	LOC_Os09g21450.1	9
2718	LOC_Os09g32680.1	9
2719	LOC_Os01g42630.1	1
2719	LOC_Os03g58400.1	3
2719	LOC_Os05g13780.1	5
2719	LOC_Os07g08500.1	7
2719	LOC_Os10g01570.1	10
2720	LOC_Os03g47460.1	3
2720	LOC_Os03g47896.1	3
2720	LOC_Os03g48200.1	3
2720	LOC_Os10g42060.1	10
2720	LOC_Os11g06490.1	11
2721	LOC_Os02g10630.1	2
2721	LOC_Os04g40070.1	4
2721	LOC_Os06g19400.1	6
2721	LOC_Os06g40570.1	6
2721	LOC_Os08g38440.1	8
2722	LOC_Os01g64000.1	1
2722	LOC_Os05g41070.1	5
2722	LOC_Os06g10880.1	6
2722	LOC_Os08g36790.1	8
2722	LOC_Os09g28310.1	9
2723	LOC_Os01g57950.1	1
2723	LOC_Os02g56960.1	2
2723	LOC_Os03g04590.1	3
2723	LOC_Os10g32920.1	10
2723	LOC_Os12g42180.1	12
2724	LOC_Os03g13170.1	3
2724	LOC_Os03g15370.1	3
2724	LOC_Os07g30640.1	7
2724	LOC_Os09g27930.1	9
2724	LOC_Os09g39500.1	9
2725	LOC_Os02g07770.1	2
2725	LOC_Os03g20900.1	3
2725	LOC_Os05g40960.1	5
2725	LOC_Os07g48596.1	7
2725	LOC_Os08g25799.1	8
2726	LOC_Os02g28980.1	2
2726	LOC_Os04g28420.1	4
2726	LOC_Os08g41390.1	8
2726	LOC_Os11g05090.1	11
2726	LOC_Os12g05090.1	12
2727	LOC_Os02g44500.1	2
2727	LOC_Os03g24380.1	3
2727	LOC_Os04g46960.1	4
2727	LOC_Os06g08670.1	6
2727	LOC_Os11g18170.1	11
2728	LOC_Os01g18660.1	1
2728	LOC_Os01g18640.1	1
2728	LOC_Os03g06200.1	3

2728	LOC_Os08g39300.1	8
2728	LOC_Os12g18900.1	12
2729	LOC_Os02g24354.1	2
2729	LOC_Os04g01690.1	4
2729	LOC_Os04g04980.1	4
2729	LOC_Os06g04070.1	6
2729	LOC_Os09g37120.1	9
2730	LOC_Os01g10820.1	1
2730	LOC_Os02g14059.1	2
2730	LOC_Os03g03360.1	3
2730	LOC_Os05g11710.1	5
2730	LOC_Os06g35730.1	6
2731	LOC_Os03g58290.1	3
2731	LOC_Os03g58300.1	3
2731	LOC_Os03g58320.1	3
2731	LOC_Os03g58260.1	3
2731	LOC_Os07g08430.1	7
2732	LOC_Os02g55940.1	2
2732	LOC_Os06g07896.1	6
2732	LOC_Os06g07996.1	6
2732	LOC_Os08g15240.1	8
2732	LOC_Os12g34550.1	12
2733	LOC_Os01g64660.1	1
2733	LOC_Os03g16050.1	3
2733	LOC_Os04g16680.1	4
2733	LOC_Os05g36270.1	5
2733	LOC_Os06g45370.1	6
2734	LOC_Os01g18420.1	1
2734	LOC_Os01g18440.1	1
2734	LOC_Os03g37670.1	3
2734	LOC_Os03g38610.1	3
2734	LOC_Os04g25870.1	4
2735	LOC_Os02g35700.1	2
2735	LOC_Os05g51700.1	5
2735	LOC_Os07g30970.1	7
2735	LOC_Os10g41410.1	10
2735	LOC_Os12g36194.1	12
2736	LOC_Os03g11440.1	3
2736	LOC_Os03g48590.1	3
2736	LOC_Os09g17830.1	9
2736	LOC_Os09g17840.1	9
2736	LOC_Os12g41840.1	12
2737	LOC_Os02g01332.1	2
2737	LOC_Os03g24020.1	3
2737	LOC_Os03g51510.1	3
2737	LOC_Os03g54040.1	3
2737	LOC_Os09g31180.1	9
2738	LOC_Os01g22390.1	1
2738	LOC_Os02g06720.1	2
2738	LOC_Os02g44380.1	2
2738	LOC_Os03g53280.1	3
2738	LOC_Os06g46480.1	6

2739	LOC_Os02g48964.1	2
2739	LOC_Os03g26870.1	3
2739	LOC_Os06g07540.1	6
2739	LOC_Os08g31560.1	8
2739	LOC_Os08g38880.1	8
2740	LOC_Os06g39710.1	6
2740	LOC_Os08g15260.1	8
2740	LOC_Os09g04680.1	9
2740	LOC_Os10g21310.1	10
2740	LOC_Os12g10600.1	12
2741	LOC_Os01g45530.1	1
2741	LOC_Os01g72049.1	1
2741	LOC_Os03g43420.1	3
2741	LOC_Os03g41530.1	3
2741	LOC_Os05g43440.1	5
2742	LOC_Os01g65410.1	1
2742	LOC_Os03g52840.1	3
2742	LOC_Os05g35440.1	5
2742	LOC_Os11g26860.1	11
2742	LOC_Os12g22030.1	12
2743	LOC_Os02g47020.1	2
2743	LOC_Os04g21110.1	4
2743	LOC_Os04g50880.1	4
2743	LOC_Os08g41790.1	8
2743	LOC_Os12g40880.1	12
2744	LOC_Os03g59040.1	3
2744	LOC_Os06g51290.1	6
2744	LOC_Os07g10130.1	7
2744	LOC_Os09g38320.1	9
2744	LOC_Os12g43130.1	12
2745	LOC_Os01g24030.1	1
2745	LOC_Os03g04120.1	3
2745	LOC_Os03g04130.1	3
2745	LOC_Os03g19240.1	3
2745	LOC_Os03g19250.1	3
2746	LOC_Os05g25310.1	5
2746	LOC_Os06g06350.1	6
2746	LOC_Os11g04980.1	11
2746	LOC_Os12g04990.1	12
2746	LOC_Os12g07110.1	12
2747	LOC_Os05g26890.1	5
2747	LOC_Os06g02130.1	6
2747	LOC_Os10g02814.1	10
2747	LOC_Os11g10050.1	11
2747	LOC_Os12g40190.1	12
2748	LOC_Os01g69030.1	1
2748	LOC_Os02g09170.1	2
2748	LOC_Os06g43630.1	6
2748	LOC_Os08g20660.1	8
2748	LOC_Os11g12810.1	11
2749	LOC_Os01g49330.1	1
2749	LOC_Os03g01222.1	3

2749	LOC_Os05g18790.1	5
2749	LOC_Os06g22560.1	6
2749	LOC_Os08g39160.1	8
2750	LOC_Os01g73560.1	1
2750	LOC_Os03g09070.1	3
2750	LOC_Os05g42220.1	5
2750	LOC_Os07g01550.1	7
2750	LOC_Os10g22980.1	10
2751	LOC_Os01g13540.1	1
2751	LOC_Os03g03900.1	3
2751	LOC_Os04g41850.1	4
2751	LOC_Os09g37710.1	9
2751	LOC_Os11g16290.1	11
2752	LOC_Os01g07200.1	1
2752	LOC_Os01g07212.1	1
2752	LOC_Os11g01830.1	11
2752	LOC_Os12g01830.1	12
2752	LOC_Os12g15314.1	12
2753	LOC_Os02g08400.1	2
2753	LOC_Os03g10810.1	3
2753	LOC_Os03g55530.1	3
2753	LOC_Os06g44100.1	6
2753	LOC_Os07g02540.1	7
2754	LOC_Os02g51830.1	2
2754	LOC_Os04g32650.1	4
2754	LOC_Os05g05840.1	5
2754	LOC_Os08g42560.1	8
2754	LOC_Os12g25710.1	12
2755	LOC_Os02g01170.1	2
2755	LOC_Os05g03100.1	5
2755	LOC_Os05g06690.1	5
2755	LOC_Os05g38830.1	5
2755	LOC_Os12g24080.1	12
2756	LOC_Os01g71050.1	1
2756	LOC_Os05g30950.1	5
2756	LOC_Os07g37270.1	7
2756	LOC_Os08g04620.1	8
2756	LOC_Os10g36350.1	10
2757	LOC_Os03g12820.1	3
2757	LOC_Os03g63770.1	3
2757	LOC_Os04g57640.1	4
2757	LOC_Os06g13860.1	6
2757	LOC_Os10g42710.1	10
2758	LOC_Os01g56740.1	1
2758	LOC_Os02g54330.1	2
2758	LOC_Os05g40430.1	5
2758	LOC_Os05g40500.1	5
2758	LOC_Os05g46150.1	5
2759	LOC_Os01g22430.1	1
2759	LOC_Os01g22480.1	1
2759	LOC_Os11g37390.1	11
2759	LOC_Os11g39559.1	11

2759	LOC_Os11g39609.1	11
2760	LOC_Os03g63850.1	3
2760	LOC_Os06g05610.1	6
2760	LOC_Os06g49340.1	6
2760	LOC_Os10g37540.1	10
2760	LOC_Os10g37570.1	10
2761	LOC_Os01g56390.1	1
2761	LOC_Os01g59690.1	1
2761	LOC_Os03g20500.1	3
2761	LOC_Os05g41130.1	5
2761	LOC_Os05g43490.1	5
2762	LOC_Os02g45320.1	2
2762	LOC_Os03g02550.1	3
2762	LOC_Os04g48270.1	4
2762	LOC_Os09g35680.1	9
2762	LOC_Os10g37830.1	10
2763	LOC_Os02g21630.1	2
2763	LOC_Os03g11950.1	3
2763	LOC_Os03g51430.1	3
2763	LOC_Os03g60610.1	3
2763	LOC_Os05g18294.1	5
2764	LOC_Os02g20590.1	2
2764	LOC_Os04g35310.1	4
2764	LOC_Os10g29290.1	10
2764	LOC_Os10g29230.1	10
2764	LOC_Os10g29330.1	10
2765	LOC_Os02g09610.1	2
2765	LOC_Os02g09620.1	2
2765	LOC_Os06g05630.1	6
2765	LOC_Os06g43044.1	6
2765	LOC_Os09g04624.1	9
2766	LOC_Os01g16350.1	1
2766	LOC_Os11g04210.1	11
2766	LOC_Os11g04670.1	11
2766	LOC_Os12g04440.1	12
2766	LOC_Os12g04020.1	12
2767	LOC_Os07g05460.1	7
2767	LOC_Os08g14180.1	8
2767	LOC_Os08g14190.1	8
2767	LOC_Os08g20130.1	8
2767	LOC_Os12g17160.1	12
2768	LOC_Os01g55890.1	1
2768	LOC_Os01g73450.1	1
2768	LOC_Os02g44000.1	2
2768	LOC_Os04g33300.1	4
2768	LOC_Os04g46460.1	4
2769	LOC_Os01g66700.1	1
2769	LOC_Os03g11980.1	3
2769	LOC_Os05g02510.1	5
2769	LOC_Os05g34320.1	5
2769	LOC_Os07g38790.1	7
2770	LOC_Os01g56940.1	1

2770	LOC_Os03g10780.1	3
2770	LOC_Os03g61820.1	3
2770	LOC_Os08g01130.1	8
2770	LOC_Os09g35000.1	9
2771	LOC_Os01g63970.1	1
2771	LOC_Os02g02620.1	2
2771	LOC_Os04g42760.1	4
2771	LOC_Os11g36420.1	11
2771	LOC_Os12g05550.1	12
2772	LOC_Os01g36870.1	1
2772	LOC_Os01g61150.1	1
2772	LOC_Os01g64280.1	1
2772	LOC_Os05g27920.1	5
2772	LOC_Os06g43560.1	6
2773	LOC_Os04g33390.1	4
2773	LOC_Os07g49390.1	7
2773	LOC_Os09g39230.1	9
2773	LOC_Os09g39260.1	9
2773	LOC_Os10g37980.1	10
2774	LOC_Os02g54050.1	2
2774	LOC_Os06g09717.1	6
2774	LOC_Os06g09790.1	6
2774	LOC_Os06g09810.1	6
2774	LOC_Os06g11940.1	6
2775	LOC_Os01g65100.1	1
2775	LOC_Os03g13240.1	3
2775	LOC_Os05g35594.1	5
2775	LOC_Os06g49220.1	6
2775	LOC_Os07g01070.1	7
2776	LOC_Os01g36580.1	1
2776	LOC_Os02g01326.1	2
2776	LOC_Os05g01570.1	5
2776	LOC_Os05g33900.1	5
2776	LOC_Os07g34070.1	7
2777	LOC_Os01g50770.1	1
2777	LOC_Os02g46510.1	2
2777	LOC_Os05g31780.1	5
2777	LOC_Os05g46550.1	5
2777	LOC_Os07g42810.1	7
2778	LOC_Os02g51620.1	2
2778	LOC_Os04g44840.1	4
2778	LOC_Os11g18690.1	11
2778	LOC_Os11g18730.1	11
2778	LOC_Os11g44950.1	11
2779	LOC_Os08g37600.1	8
2779	LOC_Os08g39370.1	8
2779	LOC_Os09g29430.1	9
2779	LOC_Os09g31130.1	9
2779	LOC_Os12g33080.1	12
2780	LOC_Os03g31210.1	3
2780	LOC_Os03g40720.1	3
2780	LOC_Os03g55070.1	3

2780	LOC_Os12g25690.1	12
2780	LOC_Os12g25700.1	12
2781	LOC_Os02g25580.1	2
2781	LOC_Os02g24134.1	2
2781	LOC_Os03g42320.1	3
2781	LOC_Os04g14654.1	4
2781	LOC_Os06g04450.1	6
2782	LOC_Os03g44310.1	3
2782	LOC_Os10g25320.1	10
2782	LOC_Os11g11050.1	11
2782	LOC_Os12g31380.1	12
2782	LOC_Os12g42260.1	12
2783	LOC_Os01g57930.1	1
2783	LOC_Os02g02750.1	2
2783	LOC_Os02g03110.1	2
2783	LOC_Os03g17520.1	3
2783	LOC_Os06g10280.1	6
2784	LOC_Os03g17000.1	3
2784	LOC_Os03g16980.1	3
2784	LOC_Os04g52730.1	4
2784	LOC_Os05g29990.1	5
2784	LOC_Os07g47700.1	7
2785	LOC_Os06g44180.1	6
2785	LOC_Os06g44170.1	6
2785	LOC_Os07g40690.1	7
2785	LOC_Os07g40974.1	7
2785	LOC_Os07g41070.1	7
2786	LOC_Os02g08370.1	2
2786	LOC_Os02g43760.1	2
2786	LOC_Os02g57630.1	2
2786	LOC_Os04g46190.1	4
2786	LOC_Os04g57190.1	4
2787	LOC_Os03g21110.1	3
2787	LOC_Os05g24020.1	5
2787	LOC_Os08g36560.1	8
2787	LOC_Os08g42050.1	8
2787	LOC_Os09g32968.1	9
2788	LOC_Os03g20010.1	3
2788	LOC_Os05g06330.1	5
2788	LOC_Os06g47940.1	6
2788	LOC_Os11g07916.1	11
2788	LOC_Os12g07700.1	12
2789	LOC_Os03g31170.1	3
2789	LOC_Os05g33630.1	5
2789	LOC_Os05g33644.1	5
2789	LOC_Os08g44370.1	8
2789	LOC_Os09g39440.1	9
2790	LOC_Os01g12220.1	1
2790	LOC_Os08g41670.1	8
2790	LOC_Os08g33630.1	8
2790	LOC_Os11g28300.1	11
2790	LOC_Os11g34180.1	11

2791	LOC_Os01g58740.1	1
2791	LOC_Os01g71280.1	1
2791	LOC_Os01g74000.1	1
2791	LOC_Os05g41590.1	5
2791	LOC_Os07g12640.1	7
2792	LOC_Os02g05500.1	2
2792	LOC_Os03g02450.1	3
2792	LOC_Os07g38620.1	7
2792	LOC_Os08g40570.1	8
2792	LOC_Os10g23120.1	10
2793	LOC_Os01g33050.1	1
2793	LOC_Os01g59990.1	1
2793	LOC_Os05g40820.1	5
2793	LOC_Os07g12250.1	7
2793	LOC_Os07g19190.1	7
2794	LOC_Os06g42770.1	6
2794	LOC_Os06g44880.1	6
2794	LOC_Os10g41790.1	10
2794	LOC_Os12g21870.1	12
2794	LOC_Os12g33990.1	12
2795	LOC_Os01g16190.1	1
2795	LOC_Os04g56070.1	4
2795	LOC_Os05g30800.1	5
2795	LOC_Os06g49020.1	6
2795	LOC_Os07g03760.1	7
2796	LOC_Os02g13180.1	2
2796	LOC_Os03g01660.1	3
2796	LOC_Os07g43240.1	7
2796	LOC_Os08g28780.1	8
2796	LOC_Os12g40300.1	12
2797	LOC_Os02g53000.1	2
2797	LOC_Os03g04110.1	3
2797	LOC_Os09g27890.1	9
2797	LOC_Os09g37600.1	9
2797	LOC_Os11g34570.1	11
2798	LOC_Os02g09810.1	2
2798	LOC_Os02g49510.1	2
2798	LOC_Os03g14910.1	3
2798	LOC_Os06g16420.1	6
2798	LOC_Os06g42720.1	6
2799	LOC_Os02g57660.1	2
2799	LOC_Os03g24160.1	3
2799	LOC_Os03g49510.1	3
2799	LOC_Os03g49800.1	3
2799	LOC_Os07g46490.1	7
2800	LOC_Os01g31870.1	1
2800	LOC_Os03g11010.1	3
2800	LOC_Os03g41064.1	3
2800	LOC_Os03g41070.1	3
2800	LOC_Os12g39180.1	12
2801	LOC_Os01g68750.1	1
2801	LOC_Os01g74180.1	1

2801	LOC_Os01g43630.1	1
2801	LOC_Os03g12590.1	3
2801	LOC_Os07g10150.1	7
2802	LOC_Os02g48190.1	2
2802	LOC_Os06g22330.1	6
2802	LOC_Os06g41910.1	6
2802	LOC_Os06g51210.1	6
2802	LOC_Os08g02850.1	8
2803	LOC_Os01g15910.1	1
2803	LOC_Os02g02560.1	2
2803	LOC_Os04g52370.1	4
2803	LOC_Os08g10600.1	8
2803	LOC_Os09g38030.1	9
2804	LOC_Os03g43880.1	3
2804	LOC_Os03g57080.1	3
2804	LOC_Os06g46350.1	6
2804	LOC_Os07g05110.1	7
2804	LOC_Os12g41720.1	12
2805	LOC_Os01g45750.1	1
2805	LOC_Os02g27490.1	2
2805	LOC_Os04g31210.1	4
2805	LOC_Os09g34900.1	9
2805	LOC_Os12g07270.1	12
2806	LOC_Os02g39570.1	2
2806	LOC_Os05g02220.1	5
2806	LOC_Os09g39740.1	9
2806	LOC_Os11g14950.1	11
2806	LOC_Os12g05650.1	12
2807	LOC_Os02g05340.1	2
2807	LOC_Os04g51910.1	4
2807	LOC_Os06g48640.1	6
2807	LOC_Os08g12820.1	8
2807	LOC_Os09g15750.1	9
2808	LOC_Os02g14860.1	2
2808	LOC_Os02g26440.1	2
2808	LOC_Os03g43020.1	3
2808	LOC_Os11g36050.1	11
2808	LOC_Os12g30060.1	12
2809	LOC_Os01g31110.1	1
2809	LOC_Os04g39060.1	4
2809	LOC_Os08g07790.1	8
2809	LOC_Os08g08860.1	8
2809	LOC_Os10g36860.1	10
2810	LOC_Os02g07310.1	2
2810	LOC_Os02g45070.1	2
2810	LOC_Os02g58490.1	2
2810	LOC_Os04g47870.1	4
2810	LOC_Os06g39640.1	6
2811	LOC_Os02g39290.1	2
2811	LOC_Os04g14110.1	4
2811	LOC_Os04g14190.1	4
2811	LOC_Os05g23950.1	5

2811	LOC_Os12g08070.1	12
2812	LOC_Os02g51680.1	2
2812	LOC_Os03g26910.1	3
2812	LOC_Os07g43160.1	7
2812	LOC_Os08g31630.1	8
2812	LOC_Os09g20390.1	9
2813	LOC_Os03g42230.1	3
2813	LOC_Os03g42240.1	3
2813	LOC_Os03g42250.1	3
2813	LOC_Os03g42290.1	3
2813	LOC_Os12g40080.1	12
2814	LOC_Os02g42440.1	2
2814	LOC_Os02g42430.1	2
2814	LOC_Os04g44500.1	4
2814	LOC_Os04g44510.1	4
2814	LOC_Os10g34730.1	10
2815	LOC_Os02g32700.1	2
2815	LOC_Os04g53240.1	4
2815	LOC_Os07g32800.1	7
2815	LOC_Os08g09240.1	8
2815	LOC_Os11g01010.1	11
2816	LOC_Os07g39530.1	7
2816	LOC_Os07g36230.1	7
2816	LOC_Os09g25330.1	9
2816	LOC_Os11g02610.1	11
2816	LOC_Os12g02530.1	12
2817	LOC_Os05g39350.1	5
2817	LOC_Os07g49280.1	7
2817	LOC_Os11g01370.1	11
2817	LOC_Os11g01570.1	11
2817	LOC_Os12g01560.1	12
2818	LOC_Os02g37590.1	2
2818	LOC_Os02g09450.1	2
2818	LOC_Os04g39610.1	4
2818	LOC_Os08g39350.1	8
2818	LOC_Os09g17000.1	9
2819	LOC_Os07g41890.1	7
2819	LOC_Os07g41980.1	7
2819	LOC_Os08g30590.1	8
2819	LOC_Os08g31250.1	8
2819	LOC_Os12g40470.1	12
2820	LOC_Os03g26200.1	3
2820	LOC_Os06g01450.1	6
2820	LOC_Os09g24900.1	9
2820	LOC_Os10g41970.1	10
2820	LOC_Os11g38860.1	11
2821	LOC_Os05g15160.1	5
2821	LOC_Os07g33954.1	7
2821	LOC_Os07g34006.1	7
2821	LOC_Os07g33910.1	7
2821	LOC_Os08g08840.1	8
2822	LOC_Os01g21250.1	1

2822	LOC_Os02g35650.1	2
2822	LOC_Os03g28260.1	3
2822	LOC_Os05g29930.1	5
2822	LOC_Os08g34990.1	8
2823	LOC_Os01g71270.1	1
2823	LOC_Os03g49580.1	3
2823	LOC_Os04g56480.1	4
2823	LOC_Os05g31020.1	5
2823	LOC_Os07g39870.1	7
2824	LOC_Os03g59340.1	3
2824	LOC_Os03g62090.1	3
2824	LOC_Os07g10770.1	7
2824	LOC_Os07g14850.1	7
2824	LOC_Os07g24190.1	7
2825	LOC_Os04g11070.1	4
2825	LOC_Os05g30080.1	5
2825	LOC_Os05g34190.1	5
2825	LOC_Os10g31340.1	10
2825	LOC_Os12g10160.1	12
2826	LOC_Os01g21290.1	1
2826	LOC_Os01g35370.1	1
2826	LOC_Os01g74130.1	1
2826	LOC_Os06g21430.1	6
2826	LOC_Os12g13750.1	12
2827	LOC_Os01g40630.1	1
2827	LOC_Os01g51210.1	1
2827	LOC_Os02g41770.1	2
2827	LOC_Os03g64070.1	3
2827	LOC_Os09g37540.1	9
2828	LOC_Os03g37090.1	3
2828	LOC_Os03g38790.1	3
2828	LOC_Os04g39450.1	4
2828	LOC_Os04g48840.1	4
2828	LOC_Os04g55930.1	4
2829	LOC_Os07g26700.1	7
2829	LOC_Os07g36800.1	7
2829	LOC_Os08g31830.1	8
2829	LOC_Os08g25590.1	8
2829	LOC_Os09g20660.1	9
2830	LOC_Os02g55870.1	2
2830	LOC_Os03g11320.1	3
2830	LOC_Os06g08090.1	6
2830	LOC_Os07g40740.1	7
2830	LOC_Os12g38920.1	12
2831	LOC_Os02g08544.1	2
2831	LOC_Os03g03164.1	3
2831	LOC_Os03g51710.1	3
2831	LOC_Os06g43860.1	6
2831	LOC_Os08g19650.1	8
2832	LOC_Os01g16670.1	1
2832	LOC_Os02g49450.1	2
2832	LOC_Os07g45310.1	7

2832	LOC_Os08g25060.1	8
2832	LOC_Os11g35320.1	11
2833	LOC_Os01g51220.1	1
2833	LOC_Os03g28400.1	3
2833	LOC_Os07g19060.1	7
2833	LOC_Os07g44790.1	7
2833	LOC_Os09g06970.1	9
2834	LOC_Os02g19420.1	2
2834	LOC_Os02g19880.1	2
2834	LOC_Os03g57130.1	3
2834	LOC_Os04g52340.1	4
2834	LOC_Os07g37790.1	7
2835	LOC_Os02g32340.1	2
2835	LOC_Os04g42330.1	4
2835	LOC_Os05g06260.1	5
2835	LOC_Os09g27370.1	9
2835	LOC_Os12g41290.1	12
2836	LOC_Os01g01190.1	1
2836	LOC_Os01g72520.1	1
2836	LOC_Os03g61130.1	3
2836	LOC_Os03g63580.1	3
2836	LOC_Os11g38050.1	11
2837	LOC_Os03g25420.1	3
2837	LOC_Os07g44320.1	7
2837	LOC_Os08g13390.1	8
2837	LOC_Os11g06680.1	11
2837	LOC_Os12g06560.1	12
2838	LOC_Os01g33030.1	1
2838	LOC_Os02g37870.1	2
2838	LOC_Os03g22350.1	3
2838	LOC_Os06g04600.1	6
2838	LOC_Os08g05880.1	8
2839	LOC_Os02g09110.1	2
2839	LOC_Os03g19050.1	3
2839	LOC_Os04g10610.1	4
2839	LOC_Os04g43500.1	4
2839	LOC_Os12g22150.1	12
2840	LOC_Os02g36960.1	2
2840	LOC_Os03g44840.1	3
2840	LOC_Os04g58504.1	4
2840	LOC_Os04g58470.1	4
2840	LOC_Os07g46240.1	7
2841	LOC_Os03g16430.1	3
2841	LOC_Os05g50930.1	5
2841	LOC_Os05g51150.1	5
2841	LOC_Os08g14450.1	8
2841	LOC_Os11g26160.1	11
2842	LOC_Os02g37930.1	2
2842	LOC_Os07g47670.1	7
2842	LOC_Os09g20900.1	9
2842	LOC_Os11g02080.1	11
2842	LOC_Os12g02040.1	12

2843	LOC_Os01g60110.1	1
2843	LOC_Os03g13050.1	3
2843	LOC_Os10g35770.1	10
2843	LOC_Os11g03130.1	11
2843	LOC_Os12g02880.1	12
2844	LOC_Os08g36040.1	8
2844	LOC_Os08g36030.1	8
2844	LOC_Os09g27250.1	9
2844	LOC_Os09g27260.1	9
2844	LOC_Os11g40570.1	11
2845	LOC_Os01g11230.1	1
2845	LOC_Os01g13420.1	1
2845	LOC_Os01g63210.1	1
2845	LOC_Os02g33020.1	2
2845	LOC_Os06g39690.1	6
2846	LOC_Os01g22900.1	1
2846	LOC_Os02g03320.1	2
2846	LOC_Os02g32730.1	2
2846	LOC_Os03g20020.1	3
2846	LOC_Os04g33490.1	4
2847	LOC_Os10g28520.1	10
2847	LOC_Os10g28540.1	10
2847	LOC_Os10g28580.1	10
2847	LOC_Os10g28450.1	10
2847	LOC_Os10g28590.1	10
2848	LOC_Os01g10680.1	1
2848	LOC_Os03g61040.1	3
2848	LOC_Os05g11650.1	5
2848	LOC_Os10g23220.1	10
2848	LOC_Os10g36400.1	10
2849	LOC_Os01g34690.1	1
2849	LOC_Os01g42520.1	1
2849	LOC_Os01g55190.1	1
2849	LOC_Os03g59300.1	3
2849	LOC_Os04g41740.1	4
2850	LOC_Os02g11040.1	2
2850	LOC_Os04g41710.1	4
2850	LOC_Os04g41750.1	4
2850	LOC_Os04g41770.1	4
2850	LOC_Os07g10620.1	7
2851	LOC_Os01g66350.1	1
2851	LOC_Os03g11500.1	3
2851	LOC_Os04g22360.1	4
2851	LOC_Os04g43690.1	4
2851	LOC_Os05g34650.1	5
2852	LOC_Os01g42480.1	1
2852	LOC_Os01g64760.1	1
2852	LOC_Os03g17990.1	3
2852	LOC_Os05g36150.1	5
2852	LOC_Os05g38180.1	5
2853	LOC_Os01g62670.1	1
2853	LOC_Os03g16390.1	3

2853	LOC_Os03g64130.1	3
2853	LOC_Os05g38320.1	5
2853	LOC_Os12g05180.1	12
2854	LOC_Os02g43850.1	2
2854	LOC_Os02g54580.1	2
2854	LOC_Os03g51670.1	3
2854	LOC_Os08g04810.1	8
2854	LOC_Os08g34750.1	8
2855	LOC_Os01g72490.1	1
2855	LOC_Os05g32070.1	5
2855	LOC_Os06g49830.1	6
2855	LOC_Os08g43410.1	8
2855	LOC_Os09g36160.1	9
2856	LOC_Os01g16110.1	1
2856	LOC_Os03g06070.1	3
2856	LOC_Os03g64190.1	3
2856	LOC_Os09g36920.1	9
2856	LOC_Os10g30320.1	10
2857	LOC_Os01g56150.1	1
2857	LOC_Os06g43930.1	6
2857	LOC_Os10g36760.1	10
2857	LOC_Os10g36780.1	10
2857	LOC_Os11g05760.1	11
2858	LOC_Os03g02590.1	3
2858	LOC_Os03g19000.1	3
2858	LOC_Os03g19010.1	3
2858	LOC_Os04g45210.1	4
2858	LOC_Os06g03660.1	6
2859	LOC_Os03g04560.1	3
2859	LOC_Os03g47930.1	3
2859	LOC_Os08g30070.1	8
2859	LOC_Os09g17760.1	9
2859	LOC_Os10g33240.1	10
2860	LOC_Os03g27800.1	3
2860	LOC_Os06g20860.1	6
2860	LOC_Os07g42700.1	7
2860	LOC_Os09g28450.1	9
2860	LOC_Os12g17310.1	12
2861	LOC_Os02g40700.1	2
2861	LOC_Os02g54360.1	2
2861	LOC_Os04g36760.1	4
2861	LOC_Os04g43060.1	4
2861	LOC_Os10g32840.1	10
2862	LOC_Os01g56230.1	1
2862	LOC_Os01g60120.1	1
2862	LOC_Os03g46410.1	3
2862	LOC_Os05g43540.1	5
2862	LOC_Os05g40700.1	5
2863	LOC_Os01g19460.1	1
2863	LOC_Os02g27850.1	2
2863	LOC_Os10g22130.1	10
2863	LOC_Os11g47410.1	11

2863	LOC_Os12g42990.1	12
2864	LOC_Os03g42380.1	3
2864	LOC_Os05g02090.1	5
2864	LOC_Os05g44786.1	5
2864	LOC_Os11g41190.1	11
2864	LOC_Os11g44830.1	11
2865	LOC_Os02g45870.1	2
2865	LOC_Os03g02850.1	3
2865	LOC_Os03g36790.1	3
2865	LOC_Os07g01250.1	7
2865	LOC_Os10g39220.1	10
2866	LOC_Os06g13680.1	6
2866	LOC_Os07g17310.1	7
2866	LOC_Os07g17330.1	7
2866	LOC_Os07g41340.1	7
2866	LOC_Os07g41350.1	7
2867	LOC_Os01g06340.1	1
2867	LOC_Os01g06350.1	1
2867	LOC_Os01g06370.1	1
2867	LOC_Os01g06390.1	1
2867	LOC_Os01g06410.1	1
2868	LOC_Os02g39620.1	2
2868	LOC_Os04g41980.1	4
2868	LOC_Os06g02420.1	6
2868	LOC_Os06g02470.1	6
2868	LOC_Os11g06240.1	11
2869	LOC_Os02g11090.1	2
2869	LOC_Os05g18100.1	5
2869	LOC_Os07g18900.1	7
2869	LOC_Os09g18410.1	9
2869	LOC_Os11g19660.1	11
2870	LOC_Os03g21690.1	3
2870	LOC_Os06g04130.1	6
2870	LOC_Os07g42310.1	7
2870	LOC_Os09g26830.1	9
2870	LOC_Os11g34360.1	11
2871	LOC_Os08g31850.1	8
2871	LOC_Os08g31860.1	8
2871	LOC_Os08g31880.1	8
2871	LOC_Os08g31890.1	8
2871	LOC_Os08g31910.1	8
2872	LOC_Os03g43840.1	3
2872	LOC_Os07g28910.1	7
2872	LOC_Os08g03610.1	8
2872	LOC_Os08g06280.1	8
2872	LOC_Os12g41700.1	12
2873	LOC_Os02g37480.1	2
2873	LOC_Os02g37490.1	2
2873	LOC_Os04g56040.1	4
2873	LOC_Os06g21310.1	6
2873	LOC_Os06g21250.1	6
2874	LOC_Os02g51770.1	2

2874	LOC_Os04g56790.1	4
2874	LOC_Os06g11790.1	6
2874	LOC_Os07g25700.1	7
2874	LOC_Os12g06100.1	12
2875	LOC_Os08g31670.1	8
2875	LOC_Os09g20490.1	9
2875	LOC_Os09g20500.1	9
2875	LOC_Os09g20510.1	9
2875	LOC_Os09g20480.1	9
2876	LOC_Os01g02950.1	1
2876	LOC_Os01g02960.1	1
2876	LOC_Os01g03260.1	1
2876	LOC_Os01g08650.1	1
2876	LOC_Os03g14310.1	3
2877	LOC_Os01g67600.1	1
2877	LOC_Os08g43430.1	8
2877	LOC_Os09g26500.1	9
2877	LOC_Os12g04140.1	12
2877	LOC_Os12g15400.1	12
2878	LOC_Os02g33740.1	2
2878	LOC_Os02g48980.1	2
2878	LOC_Os04g42210.1	4
2878	LOC_Os04g42220.1	4
2878	LOC_Os09g12620.1	9
2879	LOC_Os01g10230.1	1
2879	LOC_Os01g10210.1	1
2879	LOC_Os01g52570.1	1
2879	LOC_Os05g11210.1	5
2879	LOC_Os05g11250.1	5
2880	LOC_Os01g36700.1	1
2880	LOC_Os05g32750.1	5
2880	LOC_Os07g10760.1	7
2880	LOC_Os08g40470.1	8
2880	LOC_Os09g08500.1	9
2881	LOC_Os01g08490.1	1
2881	LOC_Os02g26490.1	2
2881	LOC_Os03g46430.1	3
2881	LOC_Os12g07180.1	12
2881	LOC_Os12g12690.1	12
2882	LOC_Os02g30780.1	2
2882	LOC_Os06g41580.1	6
2882	LOC_Os11g11540.1	11
2882	LOC_Os11g11680.1	11
2882	LOC_Os12g27850.1	12
2883	LOC_Os03g30130.1	3
2883	LOC_Os04g35100.1	4
2883	LOC_Os04g59310.1	4
2883	LOC_Os08g01310.1	8
2883	LOC_Os09g02729.1	9
2884	LOC_Os05g27749.1	5
2884	LOC_Os05g28129.1	5
2884	LOC_Os05g28140.1	5

2884	LOC_Os05g26026.1	5
2884	LOC_Os05g26049.1	5
2885	LOC_Os01g10920.1	1
2885	LOC_Os01g10960.1	1
2885	LOC_Os01g11010.1	1
2885	LOC_Os01g10930.1	1
2885	LOC_Os01g10950.1	1
2886	LOC_Os04g26520.1	4
2886	LOC_Os11g19470.1	11
2886	LOC_Os12g02140.1	12
2886	LOC_Os12g25280.1	12
2886	LOC_Os12g41770.1	12
2887	LOC_Os02g30790.1	2
2887	LOC_Os06g24920.1	6
2887	LOC_Os08g01810.1	8
2887	LOC_Os11g37120.1	11
2887	LOC_Os12g20410.1	12
2888	LOC_Os01g20710.1	1
2888	LOC_Os03g30600.1	3
2888	LOC_Os04g48440.1	4
2888	LOC_Os11g48100.1	11
2888	LOC_Os12g29510.1	12
2889	LOC_Os04g19700.1	4
2889	LOC_Os06g05990.1	6
2889	LOC_Os11g43040.1	11
2889	LOC_Os11g45380.1	11
2889	LOC_Os12g10250.1	12
2890	LOC_Os02g32900.1	2
2890	LOC_Os04g29610.1	4
2890	LOC_Os10g21970.1	10
2890	LOC_Os11g03320.1	11
2890	LOC_Os11g22250.1	11
2891	LOC_Os01g02990.1	1
2891	LOC_Os02g43380.1	2
2891	LOC_Os06g25590.1	6
2891	LOC_Os08g22840.1	8
2891	LOC_Os09g04360.1	9
2892	LOC_Os02g24940.1	2
2892	LOC_Os05g36240.1	5
2892	LOC_Os07g05840.1	7
2892	LOC_Os09g12860.1	9
2892	LOC_Os11g41770.1	11
2893	LOC_Os04g35710.1	4
2893	LOC_Os05g31800.1	5
2893	LOC_Os09g31330.1	9
2893	LOC_Os11g08000.1	11
2893	LOC_Os12g05350.1	12
2894	LOC_Os01g21730.1	1
2894	LOC_Os02g14750.1	2
2894	LOC_Os03g10330.1	3
2894	LOC_Os03g60449.1	3
2894	LOC_Os04g01270.1	4

2895	LOC_Os04g15800.1	4
2895	LOC_Os05g25070.1	5
2895	LOC_Os05g40310.1	5
2895	LOC_Os11g02640.1	11
2895	LOC_Os12g02570.1	12
2896	LOC_Os01g06050.1	1
2896	LOC_Os03g39960.1	3
2896	LOC_Os06g28790.1	6
2896	LOC_Os08g19800.1	8
2896	LOC_Os11g12010.1	11
2897	LOC_Os02g13220.1	2
2897	LOC_Os02g13260.1	2
2897	LOC_Os02g13190.1	2
2897	LOC_Os05g04570.1	5
2897	LOC_Os12g01970.1	12
2898	LOC_Os06g13460.1	6
2898	LOC_Os06g13550.1	6
2898	LOC_Os06g13510.1	6
2898	LOC_Os10g09360.1	10
2898	LOC_Os11g15340.1	11
2899	LOC_Os04g24200.1	4
2899	LOC_Os09g38480.1	9
2899	LOC_Os09g38600.1	9
2899	LOC_Os09g39350.1	9
2899	LOC_Os09g39280.1	9
2900	LOC_Os01g67290.1	1
2900	LOC_Os02g05040.1	2
2900	LOC_Os02g53990.1	2
2900	LOC_Os03g19520.1	3
2900	LOC_Os07g10550.1	7
2901	LOC_Os02g29430.1	2
2901	LOC_Os04g17590.1	4
2901	LOC_Os04g27180.1	4
2901	LOC_Os09g04650.1	9
2901	LOC_Os10g26550.1	10
2902	LOC_Os02g35356.1	2
2902	LOC_Os02g35374.1	2
2902	LOC_Os02g35392.1	2
2902	LOC_Os02g35338.1	2
2902	LOC_Os02g35420.1	2
2903	LOC_Os07g04330.1	7
2903	LOC_Os07g04450.1	7
2903	LOC_Os07g04350.1	7
2903	LOC_Os07g04500.1	7
2903	LOC_Os07g05700.1	7
2904	LOC_Os01g62170.1	1
2904	LOC_Os02g45640.1	2
2904	LOC_Os03g39602.1	3
2904	LOC_Os04g42590.1	4
2904	LOC_Os12g17560.1	12
2905	LOC_Os03g20040.1	3
2905	LOC_Os03g33900.1	3

2905	LOC_Os03g49110.1	3
2905	LOC_Os04g26580.1	4
2905	LOC_Os07g14380.1	7
2906	LOC_Os01g21144.1	1
2906	LOC_Os01g34860.1	1
2906	LOC_Os03g49126.1	3
2906	LOC_Os10g02670.1	10
2906	LOC_Os10g22100.1	10
2907	LOC_Os03g47910.1	3
2907	LOC_Os03g48250.1	3
2907	LOC_Os03g48220.1	3
2907	LOC_Os03g48230.1	3
2907	LOC_Os03g48280.1	3
2908	LOC_Os04g01830.1	4
2908	LOC_Os04g13610.1	4
2908	LOC_Os05g31100.1	5
2908	LOC_Os11g47494.1	11
2908	LOC_Os12g29240.1	12
2909	LOC_Os03g55950.1	3
2909	LOC_Os04g20920.1	4
2909	LOC_Os07g03150.1	7
2909	LOC_Os07g15600.1	7
2909	LOC_Os12g40480.1	12
2910	LOC_Os10g20290.1	10
2910	LOC_Os11g14780.1	11
2910	LOC_Os11g14800.1	11
2910	LOC_Os11g14870.1	11
2910	LOC_Os11g14940.1	11
2911	LOC_Os08g26050.1	8
2911	LOC_Os08g26120.1	8
2911	LOC_Os08g26520.1	8
2911	LOC_Os08g26360.1	8
2911	LOC_Os08g26580.1	8
2912	LOC_Os01g59310.1	1
2912	LOC_Os02g45860.1	2
2912	LOC_Os03g23250.1	3
2912	LOC_Os04g13200.1	4
2912	LOC_Os10g24140.1	10
2913	LOC_Os01g70550.1	1
2913	LOC_Os02g32504.1	2
2913	LOC_Os04g33200.1	4
2913	LOC_Os05g06360.1	5
2913	LOC_Os11g34110.1	11
2914	LOC_Os01g58300.1	1
2914	LOC_Os04g43810.1	4
2914	LOC_Os04g55630.1	4
2914	LOC_Os06g33140.1	6
2914	LOC_Os07g36050.1	7
2915	LOC_Os01g01730.1	1
2915	LOC_Os01g14970.1	1
2915	LOC_Os02g26390.1	2
2915	LOC_Os03g42090.1	3

2915	LOC_Os09g27160.1	9
2916	LOC_Os03g41870.1	3
2916	LOC_Os04g20630.1	4
2916	LOC_Os06g25520.1	6
2916	LOC_Os11g15110.1	11
2916	LOC_Os12g19580.1	12
2917	LOC_Os01g68840.1	1
2917	LOC_Os11g02820.1	11
2917	LOC_Os11g27540.1	11
2917	LOC_Os12g02800.1	12
2917	LOC_Os12g25130.1	12
2918	LOC_Os02g39460.1	2
2918	LOC_Os03g64010.1	3
2918	LOC_Os04g02790.1	4
2918	LOC_Os04g11840.1	4
2918	LOC_Os04g13710.1	4
2919	LOC_Os01g03110.1	1
2919	LOC_Os02g09080.1	2
2919	LOC_Os02g55590.1	2
2919	LOC_Os04g55110.1	4
2919	LOC_Os06g43780.1	6
2920	LOC_Os01g05280.1	1
2920	LOC_Os01g05310.1	1
2920	LOC_Os04g53080.1	4
2920	LOC_Os04g53140.1	4
2920	LOC_Os04g58460.1	4
2921	LOC_Os01g05170.1	1
2921	LOC_Os01g05250.1	1
2921	LOC_Os01g05350.1	1
2921	LOC_Os01g05410.1	1
2921	LOC_Os04g50184.1	4
2922	LOC_Os04g26120.1	4
2922	LOC_Os04g52050.1	4
2922	LOC_Os04g52040.1	4
2922	LOC_Os09g02820.1	9
2922	LOC_Os12g38410.1	12
2923	LOC_Os05g28490.1	5
2923	LOC_Os05g48440.1	5
2923	LOC_Os05g48480.1	5
2923	LOC_Os07g46740.1	7
2923	LOC_Os08g41350.1	8
2924	LOC_Os03g32810.1	3
2924	LOC_Os04g10090.1	4
2924	LOC_Os05g20640.1	5
2924	LOC_Os07g25920.1	7
2924	LOC_Os12g24340.1	12
2925	LOC_Os03g30590.1	3
2925	LOC_Os03g30620.1	3
2925	LOC_Os03g30640.1	3
2925	LOC_Os03g30680.1	3
2925	LOC_Os03g30660.1	3
2926	LOC_Os01g49810.1	1

2926	LOC_Os05g47580.1	5
2926	LOC_Os05g47590.1	5
2926	LOC_Os05g47620.1	5
2926	LOC_Os12g13400.1	12
2927	LOC_Os11g08698.1	11
2927	LOC_Os11g08707.1	11
2927	LOC_Os11g08820.1	11
2927	LOC_Os11g08909.1	11
2927	LOC_Os11g08918.1	11
2928	LOC_Os05g31310.1	5
2928	LOC_Os06g24740.1	6
2928	LOC_Os06g33220.1	6
2928	LOC_Os11g32600.1	11
2928	LOC_Os11g35370.1	11
2929	LOC_Os04g38290.1	4
2929	LOC_Os04g38260.1	4
2929	LOC_Os09g07160.1	9
2929	LOC_Os11g03620.1	11
2929	LOC_Os12g03400.1	12
2930	LOC_Os04g19670.1	4
2930	LOC_Os04g27810.1	4
2930	LOC_Os07g25960.1	7
2930	LOC_Os07g42834.1	7
2930	LOC_Os11g33280.1	11
2931	LOC_Os01g28720.1	1
2931	LOC_Os02g28880.1	2
2931	LOC_Os02g34280.1	2
2931	LOC_Os04g25430.1	4
2931	LOC_Os08g41870.1	8
2932	LOC_Os03g15260.1	3
2932	LOC_Os05g32840.1	5
2932	LOC_Os06g23100.1	6
2932	LOC_Os06g40130.1	6
2932	LOC_Os09g12680.1	9
2933	LOC_Os01g14110.1	1
2933	LOC_Os06g44320.1	6
2933	LOC_Os08g31950.1	8
2933	LOC_Os08g34510.1	8
2933	LOC_Os09g25860.1	9
2934	LOC_Os01g05920.1	1
2934	LOC_Os05g28404.1	5
2934	LOC_Os06g50499.1	6
2934	LOC_Os06g50530.1	6
2934	LOC_Os06g50569.1	6
2935	LOC_Os01g39980.1	1
2935	LOC_Os03g36260.1	3
2935	LOC_Os04g25220.1	4
2935	LOC_Os04g26100.1	4
2935	LOC_Os07g14020.1	7
2936	LOC_Os05g35430.1	5
2936	LOC_Os06g18160.1	6
2936	LOC_Os09g20580.1	9

2936	LOC_Os10g18530.1	10
2936	LOC_Os11g09700.1	11
2937	LOC_Os03g40630.1	3
2937	LOC_Os04g19460.1	4
2937	LOC_Os06g28270.1	6
2937	LOC_Os08g31330.1	8
2937	LOC_Os12g22140.1	12
2938	LOC_Os02g21920.1	2
2938	LOC_Os02g25080.1	2
2938	LOC_Os02g32580.1	2
2938	LOC_Os04g33310.1	4
2938	LOC_Os06g04480.1	6
2939	LOC_Os01g24200.1	1
2939	LOC_Os04g24680.1	4
2939	LOC_Os06g21750.1	6
2939	LOC_Os10g10020.1	10
2939	LOC_Os11g16630.1	11
2940	LOC_Os02g09850.1	2
2940	LOC_Os06g42660.1	6
2940	LOC_Os06g44420.1	6
2940	LOC_Os08g15070.1	8
2940	LOC_Os08g34700.1	8
2941	LOC_Os02g08330.1	2
2941	LOC_Os06g44240.1	6
2941	LOC_Os09g32360.1	9
2941	LOC_Os09g32370.1	9
2941	LOC_Os12g04770.1	12
2942	LOC_Os01g56080.1	1
2942	LOC_Os03g52020.1	3
2942	LOC_Os03g55980.1	3
2942	LOC_Os05g43640.1	5
2942	LOC_Os07g03170.1	7
2943	LOC_Os02g15300.1	2
2943	LOC_Os03g11280.1	3
2943	LOC_Os07g18130.1	7
2943	LOC_Os09g10250.1	9
2943	LOC_Os09g33540.1	9
2944	LOC_Os03g22130.1	3
2944	LOC_Os04g22290.1	4
2944	LOC_Os06g26160.1	6
2944	LOC_Os08g26150.1	8
2944	LOC_Os10g24340.1	10
2945	LOC_Os02g15770.1	2
2945	LOC_Os02g28030.1	2
2945	LOC_Os03g02620.1	3
2945	LOC_Os04g48320.1	4
2945	LOC_Os08g34470.1	8
2946	LOC_Os02g29620.1	2
2946	LOC_Os02g10090.1	2
2946	LOC_Os04g10870.1	4
2946	LOC_Os06g41380.1	6
2946	LOC_Os08g40010.1	8

2947	LOC_Os03g05230.1	3
2947	LOC_Os04g54960.1	4
2947	LOC_Os09g32120.1	9
2947	LOC_Os09g32130.1	9
2947	LOC_Os09g32160.1	9
2948	LOC_Os03g37800.1	3
2948	LOC_Os06g47040.1	6
2948	LOC_Os09g02800.1	9
2948	LOC_Os10g12780.1	10
2948	LOC_Os11g11870.1	11
2949	LOC_Os01g39630.1	1
2949	LOC_Os02g35450.1	2
2949	LOC_Os09g01680.1	9
2949	LOC_Os12g04980.1	12
2949	LOC_Os12g31370.1	12
2950	LOC_Os02g14140.1	2
2950	LOC_Os02g19460.1	2
2950	LOC_Os07g07130.1	7
2950	LOC_Os08g12790.1	8
2950	LOC_Os12g16320.1	12
2951	LOC_Os01g10770.1	1
2951	LOC_Os05g27910.1	5
2951	LOC_Os09g17180.1	9
2951	LOC_Os10g27200.1	10
2951	LOC_Os10g27080.1	10
2952	LOC_Os01g60150.1	1
2952	LOC_Os03g01470.1	3
2952	LOC_Os07g17000.1	7
2952	LOC_Os10g27460.1	10
2952	LOC_Os12g28780.1	12
2953	LOC_Os07g38690.1	7
2953	LOC_Os07g38700.1	7
2953	LOC_Os07g38710.1	7
2953	LOC_Os07g38650.1	7
2953	LOC_Os07g38660.1	7
2954	LOC_Os03g63380.1	3
2954	LOC_Os07g09140.1	7
2954	LOC_Os07g09150.1	7
2954	LOC_Os07g29240.1	7
2954	LOC_Os08g01240.1	8
2955	LOC_Os02g02000.1	2
2955	LOC_Os02g12690.1	2
2955	LOC_Os02g12680.1	2
2955	LOC_Os03g12500.1	3
2955	LOC_Os03g55800.1	3
2956	LOC_Os04g22370.1	4
2956	LOC_Os07g29060.1	7
2956	LOC_Os08g15920.1	8
2956	LOC_Os11g02340.1	11
2956	LOC_Os11g20780.1	11
2957	LOC_Os01g70450.1	1
2957	LOC_Os02g35960.1	2

2957	LOC_Os03g17600.1	3
2957	LOC_Os03g51950.1	3
2957	LOC_Os03g51960.1	3
2958	LOC_Os01g02160.1	1
2958	LOC_Os01g02150.1	1
2958	LOC_Os02g33790.1	2
2958	LOC_Os02g37200.1	2
2958	LOC_Os07g13500.1	7
2959	LOC_Os05g20320.1	5
2959	LOC_Os06g28260.1	6
2959	LOC_Os06g32510.1	6
2959	LOC_Os10g06860.1	10
2959	LOC_Os11g02990.1	11
2960	LOC_Os02g33580.1	2
2960	LOC_Os03g11560.1	3
2960	LOC_Os03g16140.1	3
2960	LOC_Os04g34000.1	4
2960	LOC_Os11g05990.1	11
2961	LOC_Os01g28050.1	1
2961	LOC_Os01g71790.1	1
2961	LOC_Os02g18460.1	2
2961	LOC_Os02g18470.1	2
2961	LOC_Os11g07700.1	11
2962	LOC_Os01g08670.1	1
2962	LOC_Os02g07460.1	2
2962	LOC_Os02g44580.1	2
2962	LOC_Os06g43810.1	6
2962	LOC_Os06g45610.1	6
2963	LOC_Os01g34930.1	1
2963	LOC_Os01g34880.1	1
2963	LOC_Os01g48200.1	1
2963	LOC_Os03g02756.1	3
2963	LOC_Os06g02260.1	6
2964	LOC_Os01g15340.1	1
2964	LOC_Os01g70730.1	1
2964	LOC_Os02g26210.1	2
2964	LOC_Os04g21350.1	4
2964	LOC_Os07g47450.1	7
2965	LOC_Os04g58060.1	4
2965	LOC_Os06g05090.1	6
2965	LOC_Os07g47500.1	7
2965	LOC_Os09g19560.1	9
2965	LOC_Os10g34740.1	10
2966	LOC_Os04g33160.1	4
2966	LOC_Os04g56190.1	4
2966	LOC_Os08g02740.1	8
2966	LOC_Os08g42070.1	8
2966	LOC_Os12g06400.1	12
2967	LOC_Os01g09130.1	1
2967	LOC_Os01g13850.1	1
2967	LOC_Os01g14760.1	1
2967	LOC_Os01g15139.1	1

2967	LOC_Os01g15229.1	1
2968	LOC_Os01g36100.1	1
2968	LOC_Os01g73060.1	1
2968	LOC_Os08g28510.1	8
2968	LOC_Os11g19300.1	11
2968	LOC_Os12g38160.1	12
2969	LOC_Os01g08980.1	1
2969	LOC_Os03g48820.1	3
2969	LOC_Os04g44224.1	4
2969	LOC_Os06g09200.1	6
2969	LOC_Os11g12680.1	11
2970	LOC_Os01g13690.1	1
2970	LOC_Os01g13024.1	1
2970	LOC_Os03g13510.1	3
2970	LOC_Os03g15700.1	3
2970	LOC_Os03g17960.1	3
2971	LOC_Os09g38750.1	9
2971	LOC_Os11g08280.1	11
2971	LOC_Os11g08220.1	11
2971	LOC_Os11g08240.1	11
2971	LOC_Os11g08250.1	11
2972	LOC_Os07g06770.1	7
2972	LOC_Os08g27250.1	8
2972	LOC_Os10g06810.1	10
2972	LOC_Os10g06910.1	10
2972	LOC_Os12g18920.1	12
2973	LOC_Os01g62500.1	1
2973	LOC_Os02g43350.1	2
2973	LOC_Os05g38400.1	5
2973	LOC_Os06g12370.1	6
2973	LOC_Os06g45820.1	6
2973	LOC_Os06g51029.1	6
2974	LOC_Os01g25820.1	1
2974	LOC_Os01g61880.1	1
2974	LOC_Os05g38980.1	5
2974	LOC_Os05g45210.1	5
2974	LOC_Os11g33120.1	11
2974	LOC_Os12g35610.1	12
2975	LOC_Os01g50590.1	1
2975	LOC_Os02g12890.1	2
2975	LOC_Os04g33370.1	4
2975	LOC_Os06g36920.1	6
2975	LOC_Os06g37300.1	6
2975	LOC_Os06g37330.1	6
2976	LOC_Os01g15470.1	1
2976	LOC_Os02g42620.1	2
2976	LOC_Os03g12470.1	3
2976	LOC_Os03g17550.1	3
2976	LOC_Os04g52840.1	4
2976	LOC_Os08g10070.1	8
2977	LOC_Os01g74200.1	1
2977	LOC_Os02g02600.1	2

2977	LOC_Os03g16740.1	3
2977	LOC_Os03g60710.1	3
2977	LOC_Os05g02020.1	5
2977	LOC_Os06g51170.1	6
2978	LOC_Os01g02290.1	1
2978	LOC_Os01g02840.1	1
2978	LOC_Os01g02360.1	1
2978	LOC_Os01g02440.1	1
2978	LOC_Os01g02790.1	1
2978	LOC_Os01g02810.1	1
2979	LOC_Os01g10150.1	1
2979	LOC_Os01g54100.1	1
2979	LOC_Os01g56580.1	1
2979	LOC_Os03g57940.1	3
2979	LOC_Os05g11140.1	5
2979	LOC_Os07g07560.1	7
2980	LOC_Os02g08500.1	2
2980	LOC_Os02g55320.1	2
2980	LOC_Os03g12350.1	3
2980	LOC_Os04g28130.1	4
2980	LOC_Os06g08440.1	6
2980	LOC_Os06g43910.1	6
2981	LOC_Os02g54770.1	2
2981	LOC_Os03g47800.1	3
2981	LOC_Os05g02400.1	5
2981	LOC_Os06g08840.1	6
2981	LOC_Os07g08960.1	7
2981	LOC_Os09g37720.1	9
2982	LOC_Os01g57110.1	1
2982	LOC_Os02g32570.1	2
2982	LOC_Os04g09800.1	4
2982	LOC_Os04g53720.1	4
2982	LOC_Os07g44800.1	7
2982	LOC_Os08g08220.1	8
2983	LOC_Os02g35365.1	2
2983	LOC_Os02g35383.1	2
2983	LOC_Os02g35401.1	2
2983	LOC_Os02g35329.1	2
2983	LOC_Os02g35347.1	2
2983	LOC_Os02g35429.1	2
2984	LOC_Os01g62520.1	1
2984	LOC_Os03g59980.1	3
2984	LOC_Os04g31440.1	4
2984	LOC_Os05g10970.1	5
2984	LOC_Os07g01360.1	7
2984	LOC_Os08g17690.1	8
2985	LOC_Os02g48460.1	2
2985	LOC_Os05g51670.1	5
2985	LOC_Os06g08810.1	6
2985	LOC_Os08g28730.1	8
2985	LOC_Os09g15420.1	9
2985	LOC_Os09g35800.1	9

2986	LOC_Os02g38440.1	2
2986	LOC_Os04g02620.1	4
2986	LOC_Os04g40730.1	4
2986	LOC_Os06g19550.1	6
2986	LOC_Os06g19530.1	6
2986	LOC_Os06g19610.1	6
2987	LOC_Os03g02460.1	3
2987	LOC_Os04g22380.1	4
2987	LOC_Os04g22390.1	4
2987	LOC_Os06g03830.1	6
2987	LOC_Os10g40030.1	10
2987	LOC_Os11g07922.1	11
2988	LOC_Os01g43020.1	1
2988	LOC_Os01g46570.1	1
2988	LOC_Os05g49460.1	5
2988	LOC_Os05g49520.1	5
2988	LOC_Os05g49770.1	5
2988	LOC_Os12g36950.1	12
2989	LOC_Os02g32660.1	2
2989	LOC_Os04g08270.1	4
2989	LOC_Os05g32710.1	5
2989	LOC_Os06g26234.1	6
2989	LOC_Os06g51084.1	6
2989	LOC_Os08g40930.1	8
2990	LOC_Os01g52260.1	1
2990	LOC_Os02g10830.1	2
2990	LOC_Os03g08660.1	3
2990	LOC_Os03g04140.1	3
2990	LOC_Os03g10050.1	3
2990	LOC_Os05g45710.1	5
2991	LOC_Os02g52860.1	2
2991	LOC_Os03g15690.1	3
2991	LOC_Os04g37600.1	4
2991	LOC_Os06g10810.1	6
2991	LOC_Os09g28160.1	9
2991	LOC_Os09g38100.1	9
2992	LOC_Os02g54060.1	2
2992	LOC_Os03g25050.1	3
2992	LOC_Os06g09679.1	6
2992	LOC_Os07g44740.1	7
2992	LOC_Os09g26730.1	9
2992	LOC_Os10g41710.1	10
2993	LOC_Os01g46970.1	1
2993	LOC_Os02g03580.1	2
2993	LOC_Os03g59460.1	3
2993	LOC_Os05g49420.1	5
2993	LOC_Os07g10890.1	7
2993	LOC_Os12g13170.1	12
2994	LOC_Os01g33040.1	1
2994	LOC_Os02g43050.1	2
2994	LOC_Os02g43130.1	2
2994	LOC_Os04g45580.1	4

2994	LOC_Os08g43400.1	8
2994	LOC_Os09g35890.1	9
2995	LOC_Os02g46450.1	2
2995	LOC_Os02g48100.1	2
2995	LOC_Os03g53500.1	3
2995	LOC_Os04g38630.1	4
2995	LOC_Os07g48270.1	7
2995	LOC_Os09g21520.1	9
2996	LOC_Os01g02050.1	1
2996	LOC_Os01g11054.1	1
2996	LOC_Os01g55350.1	1
2996	LOC_Os02g14770.1	2
2996	LOC_Os08g27840.1	8
2996	LOC_Os09g14670.1	9
2997	LOC_Os02g18850.1	2
2997	LOC_Os03g19410.1	3
2997	LOC_Os09g28040.1	9
2997	LOC_Os09g29950.1	9
2997	LOC_Os10g28030.1	10
2997	LOC_Os10g28020.1	10
2998	LOC_Os01g04290.1	1
2998	LOC_Os03g10469.1	3
2998	LOC_Os03g14010.1	3
2998	LOC_Os05g25560.1	5
2998	LOC_Os05g23924.1	5
2998	LOC_Os10g21110.1	10
2999	LOC_Os02g54980.1	2
2999	LOC_Os06g48510.1	6
2999	LOC_Os07g37030.1	7
2999	LOC_Os10g41760.1	10
2999	LOC_Os10g41780.1	10
2999	LOC_Os11g13850.1	11
3000	LOC_Os04g42380.1	4
3000	LOC_Os04g52354.1	4
3000	LOC_Os07g37780.1	7
3000	LOC_Os07g37770.1	7
3000	LOC_Os08g27090.1	8
3000	LOC_Os08g10608.1	8
3001	LOC_Os01g49290.1	1
3001	LOC_Os01g74146.1	1
3001	LOC_Os02g47180.1	2
3001	LOC_Os03g03150.1	3
3001	LOC_Os04g51110.1	4
3001	LOC_Os09g06680.1	9
3002	LOC_Os01g03490.1	1
3002	LOC_Os03g05750.1	3
3002	LOC_Os03g29920.1	3
3002	LOC_Os10g30450.1	10
3002	LOC_Os11g05010.1	11
3002	LOC_Os12g05040.1	12
3003	LOC_Os01g42640.1	1
3003	LOC_Os02g01540.1	2

3003	LOC_Os02g06700.1	2
3003	LOC_Os02g33140.1	2
3003	LOC_Os03g26860.1	3
3003	LOC_Os04g33750.1	4
3004	LOC_Os02g42820.1	2
3004	LOC_Os03g15940.1	3
3004	LOC_Os04g45010.1	4
3004	LOC_Os06g13030.1	6
3004	LOC_Os10g35930.1	10
3004	LOC_Os12g32620.1	12
3005	LOC_Os01g47330.1	1
3005	LOC_Os02g10540.1	2
3005	LOC_Os03g52400.1	3
3005	LOC_Os05g48050.1	5
3005	LOC_Os05g49320.1	5
3005	LOC_Os07g43310.1	7
3006	LOC_Os01g21560.1	1
3006	LOC_Os01g49380.1	1
3006	LOC_Os02g11720.1	2
3006	LOC_Os05g29974.1	5
3006	LOC_Os07g37840.1	7
3006	LOC_Os12g16180.1	12
3007	LOC_Os01g04650.1	1
3007	LOC_Os02g52790.1	2
3007	LOC_Os03g48520.1	3
3007	LOC_Os05g35260.1	5
3007	LOC_Os07g37040.1	7
3007	LOC_Os11g30350.1	11
3008	LOC_Os01g53890.1	1
3008	LOC_Os03g01110.1	3
3008	LOC_Os03g48050.1	3
3008	LOC_Os05g04940.1	5
3008	LOC_Os07g40700.1	7
3008	LOC_Os08g39600.1	8
3009	LOC_Os01g73630.1	1
3009	LOC_Os02g45600.1	2
3009	LOC_Os04g48790.1	4
3009	LOC_Os07g22580.1	7
3009	LOC_Os11g05540.1	11
3009	LOC_Os12g34840.1	12
3010	LOC_Os01g16330.1	1
3010	LOC_Os01g59160.1	1
3010	LOC_Os02g30180.1	2
3010	LOC_Os04g55150.1	4
3010	LOC_Os05g33850.1	5
3010	LOC_Os08g41500.1	8
3011	LOC_Os03g22990.1	3
3011	LOC_Os12g40350.1	12
3011	LOC_Os12g40310.1	12
3011	LOC_Os12g40320.1	12
3011	LOC_Os12g40360.1	12
3011	LOC_Os12g40370.1	12

3012	LOC_Os07g02770.1	7
3012	LOC_Os07g12600.1	7
3012	LOC_Os07g36330.1	7
3012	LOC_Os07g36340.1	7
3012	LOC_Os07g36370.1	7
3012	LOC_Os07g02760.1	7
3013	LOC_Os01g64090.1	1
3013	LOC_Os02g21660.1	2
3013	LOC_Os05g32220.1	5
3013	LOC_Os06g35470.1	6
3013	LOC_Os08g44450.1	8
3013	LOC_Os08g44380.1	8
3014	LOC_Os02g45530.1	2
3014	LOC_Os03g02700.1	3
3014	LOC_Os04g48400.1	4
3014	LOC_Os08g31030.1	8
3014	LOC_Os09g19930.1	9
3014	LOC_Os10g38050.1	10
3015	LOC_Os01g57350.1	1
3015	LOC_Os03g03400.1	3
3015	LOC_Os04g45800.1	4
3015	LOC_Os07g37580.1	7
3015	LOC_Os08g05650.1	8
3015	LOC_Os10g37280.1	10
3016	LOC_Os02g30840.1	2
3016	LOC_Os03g17920.1	3
3016	LOC_Os07g46600.1	7
3016	LOC_Os08g40860.1	8
3016	LOC_Os09g01620.1	9
3016	LOC_Os12g37380.1	12
3017	LOC_Os05g36920.1	5
3017	LOC_Os05g36930.1	5
3017	LOC_Os06g37420.1	6
3017	LOC_Os07g06980.1	7
3017	LOC_Os07g41090.1	7
3017	LOC_Os12g08220.1	12
3018	LOC_Os05g03110.1	5
3018	LOC_Os05g05660.1	5
3018	LOC_Os05g38800.1	5
3018	LOC_Os07g46180.1	7
3018	LOC_Os08g37280.1	8
3018	LOC_Os11g19810.1	11
3019	LOC_Os01g70220.1	1
3019	LOC_Os03g20430.1	3
3019	LOC_Os04g45990.1	4
3019	LOC_Os07g25450.1	7
3019	LOC_Os08g45130.1	8
3019	LOC_Os09g19830.1	9
3020	LOC_Os02g10990.1	2
3020	LOC_Os02g36380.1	2
3020	LOC_Os02g41550.1	2
3020	LOC_Os04g37920.1	4

3020	LOC_Os06g45100.1	6
3020	LOC_Os10g08580.1	10
3021	LOC_Os02g57760.1	2
3021	LOC_Os04g09670.1	4
3021	LOC_Os10g02840.1	10
3021	LOC_Os12g10170.1	12
3021	LOC_Os12g13800.1	12
3021	LOC_Os12g25870.1	12
3022	LOC_Os01g42850.1	1
3022	LOC_Os02g30310.1	2
3022	LOC_Os02g32460.1	2
3022	LOC_Os03g60550.1	3
3022	LOC_Os06g14040.1	6
3022	LOC_Os11g30410.1	11
3023	LOC_Os01g56510.1	1
3023	LOC_Os02g03870.1	2
3023	LOC_Os03g53790.1	3
3023	LOC_Os03g53800.1	3
3023	LOC_Os03g53860.1	3
3023	LOC_Os05g37700.1	5
3024	LOC_Os01g51450.1	1
3024	LOC_Os04g38620.1	4
3024	LOC_Os05g14570.1	5
3024	LOC_Os05g46230.1	5
3024	LOC_Os06g05660.1	6
3024	LOC_Os06g40920.1	6
3025	LOC_Os02g52920.1	2
3025	LOC_Os03g25930.1	3
3025	LOC_Os03g53310.1	3
3025	LOC_Os06g10760.1	6
3025	LOC_Os07g43890.1	7
3025	LOC_Os09g27980.1	9
3026	LOC_Os02g12600.1	2
3026	LOC_Os02g49270.1	2
3026	LOC_Os08g37780.1	8
3026	LOC_Os09g29630.1	9
3026	LOC_Os09g30100.1	9
3026	LOC_Os09g37860.1	9
3027	LOC_Os01g15260.1	1
3027	LOC_Os01g37490.1	1
3027	LOC_Os01g55510.1	1
3027	LOC_Os02g36990.1	2
3027	LOC_Os04g38880.1	4
3027	LOC_Os06g29690.1	6
3028	LOC_Os01g59750.1	1
3028	LOC_Os03g20630.1	3
3028	LOC_Os04g45280.1	4
3028	LOC_Os06g45660.1	6
3028	LOC_Os11g02760.1	11
3028	LOC_Os12g13120.1	12
3029	LOC_Os03g08570.1	3
3029	LOC_Os03g62510.1	3

3029	LOC_Os04g57550.1	4
3029	LOC_Os04g57560.1	4
3029	LOC_Os09g20284.1	9
3029	LOC_Os11g36440.1	11
3030	LOC_Os01g45624.1	1
3030	LOC_Os03g49190.1	3
3030	LOC_Os04g46200.1	4
3030	LOC_Os05g50110.1	5
3030	LOC_Os06g27910.1	6
3030	LOC_Os09g15520.1	9
3031	LOC_Os01g34920.1	1
3031	LOC_Os01g65460.1	1
3031	LOC_Os03g15020.1	3
3031	LOC_Os06g42310.1	6
3031	LOC_Os10g18400.1	10
3031	LOC_Os10g19960.1	10
3032	LOC_Os02g44810.1	2
3032	LOC_Os03g21980.1	3
3032	LOC_Os03g26440.1	3
3032	LOC_Os05g19954.1	5
3032	LOC_Os06g45250.1	6
3032	LOC_Os12g09290.1	12
3033	LOC_Os04g10460.1	4
3033	LOC_Os04g10410.1	4
3033	LOC_Os04g10530.1	4
3033	LOC_Os04g55050.1	4
3033	LOC_Os10g06710.1	10
3033	LOC_Os11g33090.1	11
3034	LOC_Os01g51990.1	1
3034	LOC_Os03g57920.1	3
3034	LOC_Os05g23470.1	5
3034	LOC_Os08g39450.1	8
3034	LOC_Os09g21710.1	9
3034	LOC_Os09g31200.1	9
3035	LOC_Os03g16790.1	3
3035	LOC_Os03g24900.1	3
3035	LOC_Os03g58960.1	3
3035	LOC_Os04g47410.1	4
3035	LOC_Os06g20400.1	6
3035	LOC_Os12g29560.1	12
3036	LOC_Os01g68550.1	1
3036	LOC_Os03g21780.1	3
3036	LOC_Os08g15000.1	8
3036	LOC_Os09g02260.1	9
3036	LOC_Os10g22370.1	10
3036	LOC_Os12g37100.1	12
3037	LOC_Os01g37960.1	1
3037	LOC_Os01g51060.1	1
3037	LOC_Os03g62060.1	3
3037	LOC_Os03g62070.1	3
3037	LOC_Os04g44110.1	4
3037	LOC_Os07g14590.1	7

3038	LOC_Os06g35590.1	6
3038	LOC_Os06g35560.1	6
3038	LOC_Os06g35660.1	6
3038	LOC_Os06g35700.1	6
3038	LOC_Os06g35630.1	6
3038	LOC_Os11g30310.1	11
3039	LOC_Os01g18320.1	1
3039	LOC_Os01g51320.1	1
3039	LOC_Os03g09810.1	3
3039	LOC_Os07g10490.1	7
3039	LOC_Os07g43370.1	7
3039	LOC_Os09g20260.1	9
3040	LOC_Os06g06980.1	6
3040	LOC_Os08g05790.1	8
3040	LOC_Os08g38910.1	8
3040	LOC_Os08g38900.1	8
3040	LOC_Os08g38920.1	8
3040	LOC_Os09g30360.1	9
3041	LOC_Os02g08230.1	2
3041	LOC_Os02g40784.1	2
3041	LOC_Os02g56920.1	2
3041	LOC_Os06g44300.1	6
3041	LOC_Os09g25850.1	9
3041	LOC_Os10g33250.1	10
3042	LOC_Os01g37910.1	1
3042	LOC_Os02g12740.1	2
3042	LOC_Os02g43010.1	2
3042	LOC_Os04g45470.1	4
3042	LOC_Os05g51570.1	5
3042	LOC_Os06g01610.1	6
3043	LOC_Os03g19840.1	3
3043	LOC_Os05g02200.1	5
3043	LOC_Os08g04230.1	8
3043	LOC_Os08g04240.1	8
3043	LOC_Os08g04210.1	8
3043	LOC_Os08g04250.1	8
3044	LOC_Os01g55740.1	1
3044	LOC_Os01g67040.1	1
3044	LOC_Os02g22100.1	2
3044	LOC_Os05g13370.1	5
3044	LOC_Os09g28100.1	9
3044	LOC_Os10g37760.1	10
3045	LOC_Os01g05430.1	1
3045	LOC_Os03g02530.1	3
3045	LOC_Os04g48130.1	4
3045	LOC_Os08g43320.1	8
3045	LOC_Os09g35730.1	9
3045	LOC_Os11g47840.1	11
3046	LOC_Os05g11060.1	5
3046	LOC_Os06g06280.1	6
3046	LOC_Os06g08005.1	6
3046	LOC_Os06g07905.1	6

3046	LOC_Os08g02370.1	8
3046	LOC_Os08g29710.1	8
3047	LOC_Os03g29980.1	3
3047	LOC_Os03g40100.1	3
3047	LOC_Os03g51990.1	3
3047	LOC_Os03g14370.1	3
3047	LOC_Os04g32110.1	4
3047	LOC_Os08g42100.1	8
3048	LOC_Os01g11860.1	1
3048	LOC_Os01g11880.1	1
3048	LOC_Os04g57590.1	4
3048	LOC_Os05g44330.1	5
3048	LOC_Os06g34040.1	6
3048	LOC_Os11g37920.1	11
3049	LOC_Os01g14420.1	1
3049	LOC_Os01g37100.1	1
3049	LOC_Os02g51090.1	2
3049	LOC_Os04g47640.1	4
3049	LOC_Os09g27190.1	9
3049	LOC_Os12g12970.1	12
3050	LOC_Os02g06580.1	2
3050	LOC_Os02g55150.1	2
3050	LOC_Os04g38810.1	4
3050	LOC_Os08g33430.1	8
3050	LOC_Os09g34180.1	9
3050	LOC_Os10g02980.1	10
3051	LOC_Os03g21720.1	3
3051	LOC_Os04g52190.1	4
3051	LOC_Os06g45380.1	6
3051	LOC_Os07g48229.1	7
3051	LOC_Os10g20630.1	10
3051	LOC_Os12g02390.1	12
3052	LOC_Os02g49160.1	2
3052	LOC_Os02g57250.1	2
3052	LOC_Os03g53150.1	3
3052	LOC_Os08g01780.1	8
3052	LOC_Os09g35870.1	9
3052	LOC_Os12g40900.1	12
3053	LOC_Os02g47310.1	2
3053	LOC_Os03g04340.1	3
3053	LOC_Os03g59290.1	3
3053	LOC_Os06g37610.1	6
3053	LOC_Os07g29220.1	7
3053	LOC_Os12g16650.1	12
3054	LOC_Os04g51980.1	4
3054	LOC_Os04g51990.1	4
3054	LOC_Os06g05750.1	6
3054	LOC_Os06g05790.1	6
3054	LOC_Os08g10420.1	8
3054	LOC_Os11g42480.1	11
3055	LOC_Os01g72900.1	1
3055	LOC_Os01g72910.1	1

3055	LOC_Os01g73250.1	1
3055	LOC_Os02g33820.1	2
3055	LOC_Os04g34600.1	4
3055	LOC_Os11g06720.1	11
3056	LOC_Os05g22930.1	5
3056	LOC_Os05g28950.1	5
3056	LOC_Os06g30910.1	6
3056	LOC_Os08g33900.1	8
3056	LOC_Os09g24670.1	9
3056	LOC_Os09g26570.1	9
3057	LOC_Os01g05670.1	1
3057	LOC_Os01g63260.1	1
3057	LOC_Os04g48750.1	4
3057	LOC_Os07g06800.1	7
3057	LOC_Os07g31140.1	7
3057	LOC_Os11g08110.1	11
3058	LOC_Os01g67560.1	1
3058	LOC_Os04g40980.1	4
3058	LOC_Os05g45770.1	5
3058	LOC_Os05g47530.1	5
3058	LOC_Os06g33930.1	6
3058	LOC_Os08g03370.1	8
3059	LOC_Os01g35050.1	1
3059	LOC_Os01g72210.1	1
3059	LOC_Os05g32720.1	5
3059	LOC_Os05g51630.1	5
3059	LOC_Os10g42820.1	10
3059	LOC_Os12g43720.1	12
3060	LOC_Os03g08860.1	3
3060	LOC_Os03g52670.1	3
3060	LOC_Os06g11430.1	6
3060	LOC_Os07g42890.1	7
3060	LOC_Os09g29120.1	9
3060	LOC_Os12g29400.1	12
3061	LOC_Os03g18190.1	3
3061	LOC_Os03g18350.1	3
3061	LOC_Os04g26850.1	4
3061	LOC_Os05g28510.1	5
3061	LOC_Os07g49260.1	7
3061	LOC_Os12g38110.1	12
3062	LOC_Os02g08320.1	2
3062	LOC_Os03g13040.1	3
3062	LOC_Os03g10300.1	3
3062	LOC_Os06g43620.1	6
3062	LOC_Os06g44250.1	6
3062	LOC_Os12g32640.1	12
3063	LOC_Os01g55780.1	1
3063	LOC_Os02g31030.1	2
3063	LOC_Os03g40670.1	3
3063	LOC_Os04g32320.1	4
3063	LOC_Os07g41150.1	7
3063	LOC_Os08g42390.1	8

3064	LOC_Os01g36660.1	1
3064	LOC_Os03g59240.1	3
3064	LOC_Os06g16640.1	6
3064	LOC_Os06g28000.1	6
3064	LOC_Os07g10530.1	7
3064	LOC_Os08g45170.1	8
3065	LOC_Os01g67720.1	1
3065	LOC_Os04g56510.1	4
3065	LOC_Os07g12530.1	7
3065	LOC_Os07g37180.1	7
3065	LOC_Os11g34830.1	11
3065	LOC_Os11g34750.1	11
3066	LOC_Os01g58870.1	1
3066	LOC_Os03g17740.1	3
3066	LOC_Os03g49570.1	3
3066	LOC_Os05g41480.1	5
3066	LOC_Os11g04380.1	11
3066	LOC_Os12g04170.1	12
3067	LOC_Os03g09290.1	3
3067	LOC_Os03g49150.1	3
3067	LOC_Os07g39510.1	7
3067	LOC_Os10g22410.1	10
3067	LOC_Os11g35020.1	11
3067	LOC_Os12g29960.1	12
3068	LOC_Os01g58550.1	1
3068	LOC_Os03g10220.1	3
3068	LOC_Os04g42290.1	4
3068	LOC_Os06g44050.1	6
3068	LOC_Os08g38170.1	8
3068	LOC_Os09g25290.1	9
3069	LOC_Os02g07750.1	2
3069	LOC_Os03g02830.1	3
3069	LOC_Os03g57170.1	3
3069	LOC_Os05g45370.1	5
3069	LOC_Os06g45430.1	6
3069	LOC_Os09g38768.1	9
3070	LOC_Os02g13380.1	2
3070	LOC_Os06g04950.1	6
3070	LOC_Os06g05010.1	6
3070	LOC_Os06g04990.1	6
3070	LOC_Os06g05000.1	6
3070	LOC_Os06g05020.1	6
3071	LOC_Os01g19280.1	1
3071	LOC_Os05g40620.1	5
3071	LOC_Os05g40550.1	5
3071	LOC_Os05g46190.1	5
3071	LOC_Os05g49490.1	5
3071	LOC_Os05g49500.1	5
3072	LOC_Os01g67670.1	1
3072	LOC_Os01g67650.1	1
3072	LOC_Os03g09280.1	3
3072	LOC_Os07g39470.1	7

3072	LOC_Os10g22430.1	10
3072	LOC_Os11g31100.1	11
3073	LOC_Os02g45210.1	2
3073	LOC_Os03g02630.1	3
3073	LOC_Os04g57470.1	4
3073	LOC_Os06g32630.1	6
3073	LOC_Os08g05270.1	8
3073	LOC_Os11g26810.1	11
3074	LOC_Os01g67330.1	1
3074	LOC_Os02g39200.1	2
3074	LOC_Os02g36390.1	2
3074	LOC_Os04g41320.1	4
3074	LOC_Os06g33210.1	6
3074	LOC_Os07g38610.1	7
3075	LOC_Os01g05720.1	1
3075	LOC_Os01g05744.1	1
3075	LOC_Os01g05694.1	1
3075	LOC_Os01g05710.1	1
3075	LOC_Os04g20280.1	4
3075	LOC_Os05g38590.1	5
3076	LOC_Os01g02920.1	1
3076	LOC_Os01g02930.1	1
3076	LOC_Os01g02940.1	1
3076	LOC_Os02g22380.1	2
3076	LOC_Os06g27560.1	6
3076	LOC_Os06g49300.1	6
3077	LOC_Os03g24410.1	3
3077	LOC_Os07g02520.1	7
3077	LOC_Os07g02480.1	7
3077	LOC_Os07g27280.1	7
3077	LOC_Os10g31250.1	10
3077	LOC_Os11g06990.1	11
3078	LOC_Os02g06380.1	2
3078	LOC_Os04g55640.1	4
3078	LOC_Os06g47310.1	6
3078	LOC_Os11g13870.1	11
3078	LOC_Os11g29780.1	11
3078	LOC_Os12g10320.1	12
3079	LOC_Os02g43590.1	2
3079	LOC_Os03g20670.1	3
3079	LOC_Os04g01150.1	4
3079	LOC_Os04g46079.1	4
3079	LOC_Os11g29350.1	11
3079	LOC_Os12g03270.1	12
3080	LOC_Os01g61060.1	1
3080	LOC_Os03g18790.1	3
3080	LOC_Os05g27570.1	5
3080	LOC_Os05g39730.1	5
3080	LOC_Os11g01842.1	11
3080	LOC_Os11g01850.1	11
3081	LOC_Os01g64870.1	1
3081	LOC_Os02g42530.1	2

3081	LOC_Os03g06180.1	3
3081	LOC_Os04g58100.1	4
3081	LOC_Os05g17990.1	5
3081	LOC_Os10g03830.1	10
3082	LOC_Os01g37980.1	1
3082	LOC_Os01g51120.1	1
3082	LOC_Os02g03450.1	2
3082	LOC_Os03g45260.1	3
3082	LOC_Os08g34140.1	8
3082	LOC_Os09g24980.1	9
3083	LOC_Os01g37600.1	1
3083	LOC_Os05g04180.1	5
3083	LOC_Os05g05570.1	5
3083	LOC_Os05g04860.1	5
3083	LOC_Os10g41030.1	10
3083	LOC_Os11g35060.1	11
3084	LOC_Os02g42920.1	2
3084	LOC_Os04g45160.1	4
3084	LOC_Os04g45140.1	4
3084	LOC_Os05g05690.1	5
3084	LOC_Os06g05940.1	6
3084	LOC_Os08g01150.1	8
3085	LOC_Os01g07530.1	1
3085	LOC_Os03g59430.1	3
3085	LOC_Os04g40520.1	4
3085	LOC_Os06g07600.1	6
3085	LOC_Os07g10840.1	7
3085	LOC_Os08g38710.1	8
3086	LOC_Os02g26720.1	2
3086	LOC_Os03g12840.1	3
3086	LOC_Os03g51610.1	3
3086	LOC_Os09g34300.1	9
3086	LOC_Os10g01480.1	10
3086	LOC_Os10g42550.1	10
3087	LOC_Os02g40100.1	2
3087	LOC_Os03g14250.1	3
3087	LOC_Os04g42610.1	4
3087	LOC_Os04g55570.1	4
3087	LOC_Os10g07480.1	10
3087	LOC_Os12g07160.1	12
3088	LOC_Os05g23460.1	5
3088	LOC_Os05g37210.1	5
3088	LOC_Os05g23440.1	5
3088	LOC_Os05g37200.1	5
3088	LOC_Os08g42720.1	8
3088	LOC_Os09g33830.1	9
3089	LOC_Os03g08390.1	3
3089	LOC_Os07g39930.1	7
3089	LOC_Os08g42870.1	8
3089	LOC_Os09g34110.1	9
3089	LOC_Os11g02740.1	11
3089	LOC_Os12g02710.1	12

3090	LOC_Os01g62200.1	1
3090	LOC_Os01g74370.1	1
3090	LOC_Os03g09200.1	3
3090	LOC_Os03g48600.1	3
3090	LOC_Os08g44760.1	8
3090	LOC_Os09g39750.1	9
3091	LOC_Os01g40880.1	1
3091	LOC_Os01g46850.1	1
3091	LOC_Os02g11880.1	2
3091	LOC_Os02g16500.1	2
3091	LOC_Os05g12580.1	5
3091	LOC_Os10g06970.1	10
3092	LOC_Os03g12990.1	3
3092	LOC_Os03g47230.1	3
3092	LOC_Os07g03200.1	7
3092	LOC_Os11g05190.1	11
3092	LOC_Os11g35310.1	11
3092	LOC_Os12g05260.1	12
3093	LOC_Os03g58580.1	3
3093	LOC_Os07g09010.1	7
3093	LOC_Os08g42010.1	8
3093	LOC_Os10g08850.1	10
3093	LOC_Os12g44060.1	12
3093	LOC_Os12g44070.1	12
3094	LOC_Os03g48660.1	3
3094	LOC_Os03g55180.1	3
3094	LOC_Os08g05640.1	8
3094	LOC_Os08g34060.1	8
3094	LOC_Os10g33930.1	10
3094	LOC_Os12g38850.1	12
3095	LOC_Os02g25070.1	2
3095	LOC_Os04g09370.1	4
3095	LOC_Os05g15440.1	5
3095	LOC_Os07g18020.1	7
3095	LOC_Os09g06160.1	9
3095	LOC_Os10g15330.1	10
3096	LOC_Os01g06060.1	1
3096	LOC_Os01g06210.1	1
3096	LOC_Os07g06830.1	7
3096	LOC_Os07g06860.1	7
3096	LOC_Os07g06840.1	7
3096	LOC_Os07g06850.1	7
3097	LOC_Os01g04280.1	1
3097	LOC_Os02g08120.1	2
3097	LOC_Os02g35470.1	2
3097	LOC_Os03g32160.1	3
3097	LOC_Os04g36660.1	4
3097	LOC_Os09g13890.1	9
3098	LOC_Os03g09310.1	3
3098	LOC_Os03g39129.1	3
3098	LOC_Os03g39160.1	3
3098	LOC_Os03g63440.1	3

3098	LOC_Os07g40270.1	7
3098	LOC_Os09g07380.1	9
3099	LOC_Os03g16600.1	3
3099	LOC_Os05g01960.1	5
3099	LOC_Os05g08620.1	5
3099	LOC_Os05g32770.1	5
3099	LOC_Os05g38380.1	5
3099	LOC_Os09g13590.1	9
3100	LOC_Os04g32210.1	4
3100	LOC_Os04g32220.1	4
3100	LOC_Os04g32160.1	4
3100	LOC_Os04g32250.1	4
3100	LOC_Os04g32260.1	4
3100	LOC_Os12g27994.1	12
3101	LOC_Os04g19870.1	4
3101	LOC_Os04g55790.1	4
3101	LOC_Os10g10880.1	10
3101	LOC_Os10g14290.1	10
3101	LOC_Os11g08800.1	11
3101	LOC_Os12g11240.1	12
3102	LOC_Os03g42160.1	3
3102	LOC_Os04g04470.1	4
3102	LOC_Os04g07560.1	4
3102	LOC_Os04g07800.1	4
3102	LOC_Os05g24440.1	5
3102	LOC_Os05g14690.1	5
3103	LOC_Os02g50250.1	2
3103	LOC_Os03g39220.1	3
3103	LOC_Os03g39370.1	3
3103	LOC_Os03g39240.1	3
3103	LOC_Os10g42072.1	10
3103	LOC_Os10g42080.1	10
3104	LOC_Os02g24490.1	2
3104	LOC_Os02g36480.1	2
3104	LOC_Os03g31520.1	3
3104	LOC_Os05g19400.1	5
3104	LOC_Os08g32180.1	8
3104	LOC_Os12g27590.1	12
3105	LOC_Os01g03060.1	1
3105	LOC_Os02g34500.1	2
3105	LOC_Os03g10590.1	3
3105	LOC_Os05g08600.1	5
3105	LOC_Os11g34150.1	11
3105	LOC_Os12g07530.1	12
3106	LOC_Os01g69010.1	1
3106	LOC_Os03g11734.1	3
3106	LOC_Os05g33910.1	5
3106	LOC_Os09g37610.1	9
3106	LOC_Os12g06050.1	12
3106	LOC_Os12g25160.1	12
3107	LOC_Os11g28940.1	11
3107	LOC_Os11g32070.1	11

3107	LOC_Os11g32080.1	11
3107	LOC_Os12g27960.1	12
3107	LOC_Os12g27930.1	12
3107	LOC_Os12g27940.1	12
3108	LOC_Os02g53090.1	2
3108	LOC_Os04g10880.1	4
3108	LOC_Os04g17030.1	4
3108	LOC_Os10g07190.1	10
3108	LOC_Os11g29160.1	11
3108	LOC_Os11g39460.1	11
3109	LOC_Os01g16840.1	1
3109	LOC_Os01g46910.1	1
3109	LOC_Os02g29360.1	2
3109	LOC_Os04g32140.1	4
3109	LOC_Os05g08680.1	5
3109	LOC_Os05g15710.1	5
3110	LOC_Os02g12460.1	2
3110	LOC_Os02g13020.1	2
3110	LOC_Os02g15050.1	2
3110	LOC_Os07g46470.1	7
3110	LOC_Os11g34640.1	11
3110	LOC_Os11g47640.1	11
3111	LOC_Os04g24790.1	4
3111	LOC_Os04g24800.1	4
3111	LOC_Os04g25920.1	4
3111	LOC_Os04g25930.1	4
3111	LOC_Os04g28970.1	4
3111	LOC_Os08g33130.1	8
3112	LOC_Os01g41390.1	1
3112	LOC_Os02g04370.1	2
3112	LOC_Os03g41879.1	3
3112	LOC_Os03g41899.1	3
3112	LOC_Os05g25030.1	5
3112	LOC_Os07g22260.1	7
3113	LOC_Os02g33380.1	2
3113	LOC_Os02g33390.1	2
3113	LOC_Os03g01020.1	3
3113	LOC_Os04g49720.1	4
3113	LOC_Os05g05640.1	5
3113	LOC_Os12g40750.1	12
3114	LOC_Os01g23520.1	1
3114	LOC_Os05g05960.1	5
3114	LOC_Os07g16920.1	7
3114	LOC_Os10g18910.1	10
3114	LOC_Os12g12280.1	12
3114	LOC_Os12g32370.1	12
3115	LOC_Os01g67760.1	1
3115	LOC_Os05g32920.1	5
3115	LOC_Os07g13719.1	7
3115	LOC_Os07g14870.1	7
3115	LOC_Os08g01010.1	8
3115	LOC_Os11g10950.1	11

3116	LOC_Os06g50210.1	6
3116	LOC_Os07g23494.1	7
3116	LOC_Os09g02050.1	9
3116	LOC_Os10g13840.1	10
3116	LOC_Os11g16250.1	11
3116	LOC_Os11g18710.1	11
3117	LOC_Os03g07650.1	3
3117	LOC_Os03g07660.1	3
3117	LOC_Os03g07700.1	3
3117	LOC_Os03g07610.1	3
3117	LOC_Os03g07620.1	3
3117	LOC_Os03g31510.1	3
3118	LOC_Os01g22080.1	1
3118	LOC_Os02g27210.1	2
3118	LOC_Os06g22260.1	6
3118	LOC_Os08g37860.1	8
3118	LOC_Os08g39230.1	8
3118	LOC_Os09g29610.1	9
3119	LOC_Os01g28610.1	1
3119	LOC_Os06g22370.1	6
3119	LOC_Os08g24460.1	8
3119	LOC_Os11g13910.1	11
3119	LOC_Os11g28670.1	11
3119	LOC_Os11g40910.1	11
3120	LOC_Os01g55570.1	1
3120	LOC_Os02g52260.1	2
3120	LOC_Os06g15400.1	6
3120	LOC_Os07g28610.1	7
3120	LOC_Os08g33380.1	8
3120	LOC_Os10g39150.1	10
3121	LOC_Os02g34610.1	2
3121	LOC_Os05g46630.1	5
3121	LOC_Os06g11160.1	6
3121	LOC_Os06g16480.1	6
3121	LOC_Os06g30620.1	6
3121	LOC_Os08g26030.1	8
3122	LOC_Os01g70230.1	1
3122	LOC_Os02g07800.1	2
3122	LOC_Os02g31160.1	2
3122	LOC_Os03g46350.1	3
3122	LOC_Os04g32590.1	4
3122	LOC_Os04g57530.1	4
3123	LOC_Os01g33530.1	1
3123	LOC_Os03g42960.1	3
3123	LOC_Os04g25110.1	4
3123	LOC_Os09g11860.1	9
3123	LOC_Os09g23240.1	9
3123	LOC_Os12g17690.1	12
3124	LOC_Os01g66360.1	1
3124	LOC_Os03g13530.1	3
3124	LOC_Os04g30940.1	4
3124	LOC_Os05g35444.1	5

3124	LOC_Os08g37230.1	8
3124	LOC_Os08g44780.1	8
3125	LOC_Os01g14410.1	1
3125	LOC_Os02g16560.1	2
3125	LOC_Os03g30400.1	3
3125	LOC_Os05g08110.1	5
3125	LOC_Os07g08160.1	7
3125	LOC_Os07g08150.1	7
3126	LOC_Os02g26760.1	2
3126	LOC_Os02g50870.1	2
3126	LOC_Os08g35300.1	8
3126	LOC_Os08g38680.1	8
3126	LOC_Os09g18070.1	9
3126	LOC_Os11g31260.1	11
3127	LOC_Os05g10420.1	5
3127	LOC_Os05g10480.1	5
3127	LOC_Os05g10560.1	5
3127	LOC_Os11g39780.1	11
3127	LOC_Os11g39840.1	11
3127	LOC_Os11g40740.1	11
3128	LOC_Os01g40070.1	1
3128	LOC_Os05g09150.1	5
3128	LOC_Os05g09210.1	5
3128	LOC_Os05g09240.1	5
3128	LOC_Os05g09280.1	5
3128	LOC_Os05g51520.1	5
3129	LOC_Os02g09670.1	2
3129	LOC_Os03g14100.1	3
3129	LOC_Os04g32570.1	4
3129	LOC_Os04g51800.1	4
3129	LOC_Os09g32972.1	9
3129	LOC_Os12g02430.1	12
3130	LOC_Os01g28460.1	1
3130	LOC_Os03g33970.1	3
3130	LOC_Os05g22280.1	5
3130	LOC_Os08g11100.1	8
3130	LOC_Os09g19520.1	9
3130	LOC_Os10g02450.1	10
3131	LOC_Os06g46730.1	6
3131	LOC_Os09g37580.1	9
3131	LOC_Os11g27020.1	11
3131	LOC_Os11g38620.1	11
3131	LOC_Os11g38630.1	11
3131	LOC_Os11g38640.1	11
3132	LOC_Os03g63540.1	3
3132	LOC_Os06g26020.1	6
3132	LOC_Os06g49680.1	6
3132	LOC_Os08g38250.1	8
3132	LOC_Os10g15230.1	10
3132	LOC_Os12g22360.1	12
3133	LOC_Os01g11530.1	1
3133	LOC_Os04g12370.1	4

3133	LOC_Os05g06400.1	5
3133	LOC_Os06g32610.1	6
3133	LOC_Os07g35020.1	7
3133	LOC_Os12g22490.1	12
3134	LOC_Os01g37320.1	1
3134	LOC_Os02g31860.1	2
3134	LOC_Os04g04410.1	4
3134	LOC_Os05g39490.1	5
3134	LOC_Os06g42410.1	6
3134	LOC_Os06g42530.1	6
3135	LOC_Os06g12470.1	6
3135	LOC_Os09g17160.1	9
3135	LOC_Os09g40014.1	9
3135	LOC_Os09g40022.1	9
3135	LOC_Os09g40075.1	9
3135	LOC_Os11g43810.1	11
3136	LOC_Os01g28940.1	1
3136	LOC_Os08g08510.1	8
3136	LOC_Os09g01510.1	9
3136	LOC_Os09g04780.1	9
3136	LOC_Os11g20070.1	11
3136	LOC_Os12g10150.1	12
3137	LOC_Os01g19250.1	1
3137	LOC_Os03g06030.1	3
3137	LOC_Os03g15060.1	3
3137	LOC_Os07g34330.1	7
3137	LOC_Os09g32880.1	9
3137	LOC_Os11g34100.1	11
3138	LOC_Os01g33930.1	1
3138	LOC_Os07g45540.1	7
3138	LOC_Os07g45950.1	7
3138	LOC_Os07g45760.1	7
3138	LOC_Os07g46060.1	7
3138	LOC_Os07g46070.1	7
3139	LOC_Os01g41100.1	1
3139	LOC_Os03g07630.1	3
3139	LOC_Os04g33090.1	4
3139	LOC_Os08g10390.1	8
3139	LOC_Os08g44600.1	8
3139	LOC_Os09g22240.1	9
3140	LOC_Os04g08750.1	4
3140	LOC_Os05g04310.1	5
3140	LOC_Os07g33520.1	7
3140	LOC_Os08g27370.1	8
3140	LOC_Os09g15930.1	9
3140	LOC_Os12g35510.1	12
3141	LOC_Os03g44030.1	3
3141	LOC_Os05g14770.1	5
3141	LOC_Os05g15100.1	5
3141	LOC_Os11g28390.1	11
3141	LOC_Os11g30520.1	11
3141	LOC_Os12g08390.1	12

3142	LOC_Os02g50810.1	2
3142	LOC_Os03g10320.1	3
3142	LOC_Os06g12960.1	6
3142	LOC_Os08g32520.1	8
3142	LOC_Os09g21380.1	9
3142	LOC_Os10g21130.1	10
3143	LOC_Os03g07090.1	3
3143	LOC_Os04g25880.1	4
3143	LOC_Os06g15800.1	6
3143	LOC_Os10g03210.1	10
3143	LOC_Os11g01900.1	11
3143	LOC_Os11g08990.1	11
3144	LOC_Os01g37710.1	1
3144	LOC_Os03g52900.1	3
3144	LOC_Os04g03200.1	4
3144	LOC_Os04g10770.1	4
3144	LOC_Os08g29030.1	8
3144	LOC_Os08g31320.1	8
3145	LOC_Os05g02080.1	5
3145	LOC_Os05g44792.1	5
3145	LOC_Os11g01460.1	11
3145	LOC_Os11g38310.1	11
3145	LOC_Os11g41180.1	11
3145	LOC_Os11g44840.1	11
3146	LOC_Os04g28720.1	4
3146	LOC_Os04g28830.1	4
3146	LOC_Os04g31430.1	4
3146	LOC_Os11g26190.1	11
3146	LOC_Os12g38350.1	12
3146	LOC_Os12g38370.1	12
3147	LOC_Os01g25750.1	1
3147	LOC_Os03g32880.1	3
3147	LOC_Os03g33837.1	3
3147	LOC_Os09g02970.1	9
3147	LOC_Os10g26300.1	10
3147	LOC_Os12g27540.1	12
3148	LOC_Os01g31340.1	1
3148	LOC_Os03g36370.1	3
3148	LOC_Os06g39150.1	6
3148	LOC_Os06g42360.1	6
3148	LOC_Os08g30160.1	8
3148	LOC_Os08g31160.1	8
3149	LOC_Os01g09980.1	1
3149	LOC_Os03g10270.1	3
3149	LOC_Os04g30710.1	4
3149	LOC_Os11g17270.1	11
3149	LOC_Os12g29940.1	12
3149	LOC_Os12g37540.1	12
3150	LOC_Os03g38490.1	3
3150	LOC_Os04g51280.1	4
3150	LOC_Os06g02600.1	6
3150	LOC_Os08g04450.1	8

3150	LOC_Os09g33480.1	9
3150	LOC_Os11g11020.1	11
3151	LOC_Os01g53380.1	1
3151	LOC_Os04g24840.1	4
3151	LOC_Os04g25820.1	4
3151	LOC_Os05g08510.1	5
3151	LOC_Os06g17110.1	6
3151	LOC_Os08g07270.1	8
3152	LOC_Os02g12020.1	2
3152	LOC_Os02g11990.1	2
3152	LOC_Os02g12090.1	2
3152	LOC_Os02g12070.1	2
3152	LOC_Os02g12080.1	2
3152	LOC_Os02g12250.1	2
3153	LOC_Os02g26800.1	2
3153	LOC_Os03g42954.1	3
3153	LOC_Os06g20260.1	6
3153	LOC_Os07g34890.1	7
3153	LOC_Os08g03750.1	8
3153	LOC_Os09g23250.1	9
3154	LOC_Os03g41160.1	3
3154	LOC_Os03g62000.1	3
3154	LOC_Os07g11620.1	7
3154	LOC_Os09g07110.1	9
3154	LOC_Os10g29430.1	10
3154	LOC_Os11g08530.1	11
3155	LOC_Os01g25920.1	1
3155	LOC_Os04g27800.1	4
3155	LOC_Os06g27610.1	6
3155	LOC_Os11g14810.1	11
3155	LOC_Os11g14930.1	11
3155	LOC_Os11g15590.1	11
3156	LOC_Os03g45000.1	3
3156	LOC_Os03g46134.1	3
3156	LOC_Os04g39310.1	4
3156	LOC_Os05g14250.1	5
3156	LOC_Os06g14830.1	6
3156	LOC_Os08g20670.1	8
3157	LOC_Os08g43630.1	8
3157	LOC_Os09g02570.1	9
3157	LOC_Os09g02580.1	9
3157	LOC_Os09g36950.1	9
3157	LOC_Os09g36960.1	9
3157	LOC_Os09g36970.1	9
3158	LOC_Os01g71150.1	1
3158	LOC_Os02g53310.1	2
3158	LOC_Os04g46340.1	4
3158	LOC_Os04g50670.1	4
3158	LOC_Os06g10720.1	6
3158	LOC_Os09g28170.1	9
3159	LOC_Os01g15730.1	1
3159	LOC_Os05g34200.1	5

3159	LOC_Os07g19590.1	7
3159	LOC_Os07g46200.1	7
3159	LOC_Os11g33938.1	11
3159	LOC_Os12g32320.1	12
3160	LOC_Os01g12660.1	1
3160	LOC_Os01g43480.1	1
3160	LOC_Os02g51400.1	2
3160	LOC_Os03g22420.1	3
3160	LOC_Os05g50750.1	5
3160	LOC_Os07g47530.1	7
3160	LOC_Os11g43970.1	11
3161	LOC_Os01g52470.1	1
3161	LOC_Os01g53900.1	1
3161	LOC_Os02g32030.1	2
3161	LOC_Os03g36780.1	3
3161	LOC_Os04g02820.1	4
3161	LOC_Os04g45490.1	4
3161	LOC_Os06g40600.1	6
3162	LOC_Os01g04710.1	1
3162	LOC_Os01g41550.1	1
3162	LOC_Os04g37550.1	4
3162	LOC_Os05g51220.1	5
3162	LOC_Os06g02900.1	6
3162	LOC_Os06g20140.1	6
3162	LOC_Os09g25570.1	9
3163	LOC_Os01g50170.1	1
3163	LOC_Os03g20270.1	3
3163	LOC_Os03g20290.1	3
3163	LOC_Os06g02780.1	6
3163	LOC_Os06g20110.1	6
3163	LOC_Os07g34940.1	7
3163	LOC_Os11g10910.1	11
3164	LOC_Os01g41510.1	1
3164	LOC_Os01g51420.1	1
3164	LOC_Os01g39770.1	1
3164	LOC_Os02g18880.1	2
3164	LOC_Os02g18930.1	2
3164	LOC_Os02g27940.1	2
3164	LOC_Os10g41510.1	10
3165	LOC_Os02g07490.1	2
3165	LOC_Os02g38920.1	2
3165	LOC_Os03g03720.1	3
3165	LOC_Os04g40950.1	4
3165	LOC_Os04g38600.1	4
3165	LOC_Os06g45590.1	6
3165	LOC_Os08g03290.1	8
3166	LOC_Os01g59020.1	1
3166	LOC_Os01g58960.1	1
3166	LOC_Os01g58970.1	1
3166	LOC_Os01g58990.1	1
3166	LOC_Os04g48460.1	4
3166	LOC_Os10g38090.1	10

3166	LOC_Os10g38120.1	10
3167	LOC_Os02g19530.1	2
3167	LOC_Os04g44900.1	4
3167	LOC_Os07g03790.1	7
3167	LOC_Os07g03820.1	7
3167	LOC_Os07g03920.1	7
3167	LOC_Os07g04000.1	7
3167	LOC_Os10g30540.1	10
3168	LOC_Os01g55450.1	1
3168	LOC_Os02g08140.1	2
3168	LOC_Os03g43440.1	3
3168	LOC_Os05g39900.1	5
3168	LOC_Os05g43840.1	5
3168	LOC_Os07g48090.1	7
3168	LOC_Os09g25100.1	9
3169	LOC_Os01g35184.1	1
3169	LOC_Os03g03510.1	3
3169	LOC_Os07g48760.1	7
3169	LOC_Os08g34240.1	8
3169	LOC_Os09g25090.1	9
3169	LOC_Os11g03970.1	11
3169	LOC_Os12g03810.1	12
3170	LOC_Os01g20880.1	1
3170	LOC_Os07g31190.1	7
3170	LOC_Os07g31210.1	7
3170	LOC_Os07g31130.1	7
3170	LOC_Os07g31250.1	7
3170	LOC_Os08g27780.1	8
3170	LOC_Os11g35120.1	11
3171	LOC_Os01g50400.1	1
3171	LOC_Os01g50410.1	1
3171	LOC_Os01g50420.1	1
3171	LOC_Os05g46750.1	5
3171	LOC_Os05g46760.1	5
3171	LOC_Os10g04000.1	10
3171	LOC_Os10g04010.1	10
3172	LOC_Os01g21970.1	1
3172	LOC_Os01g51290.1	1
3172	LOC_Os03g06330.1	3
3172	LOC_Os03g62700.1	3
3172	LOC_Os04g59320.1	4
3172	LOC_Os08g29040.1	8
3172	LOC_Os11g35220.1	11
3173	LOC_Os01g02390.1	1
3173	LOC_Os01g02410.1	1
3173	LOC_Os01g02550.1	1
3173	LOC_Os01g02570.1	1
3173	LOC_Os01g02350.1	1
3173	LOC_Os01g02680.1	1
3173	LOC_Os01g02700.1	1
3174	LOC_Os02g45130.1	2
3174	LOC_Os05g01780.1	5

3174	LOC_Os07g08750.1	7
3174	LOC_Os07g38530.1	7
3174	LOC_Os11g02300.1	11
3174	LOC_Os12g02250.1	12
3174	LOC_Os12g06490.1	12
3175	LOC_Os01g09850.1	1
3175	LOC_Os01g14010.1	1
3175	LOC_Os01g39110.1	1
3175	LOC_Os02g45054.1	2
3175	LOC_Os05g37190.1	5
3175	LOC_Os07g39310.1	7
3175	LOC_Os08g44050.1	8
3176	LOC_Os01g11520.1	1
3176	LOC_Os01g55110.1	1
3176	LOC_Os01g60730.1	1
3176	LOC_Os02g15120.1	2
3176	LOC_Os02g54830.1	2
3176	LOC_Os08g34550.1	8
3176	LOC_Os09g20980.1	9
3177	LOC_Os01g29880.1	1
3177	LOC_Os03g41210.1	3
3177	LOC_Os07g15250.1	7
3177	LOC_Os09g10140.1	9
3177	LOC_Os09g10160.1	9
3177	LOC_Os09g10120.1	9
3177	LOC_Os12g31900.1	12
3178	LOC_Os01g03670.1	1
3178	LOC_Os01g61230.1	1
3178	LOC_Os04g53780.1	4
3178	LOC_Os04g53860.1	4
3178	LOC_Os06g46920.1	6
3178	LOC_Os08g08500.1	8
3178	LOC_Os09g31498.1	9
3179	LOC_Os02g56180.1	2
3179	LOC_Os03g01190.1	3
3179	LOC_Os04g28870.1	4
3179	LOC_Os04g30420.1	4
3179	LOC_Os09g32640.1	9
3179	LOC_Os09g32570.1	9
3179	LOC_Os10g41170.1	10
3180	LOC_Os02g57040.1	2
3180	LOC_Os03g12270.1	3
3180	LOC_Os08g01760.1	8
3180	LOC_Os09g23540.1	9
3180	LOC_Os09g23550.1	9
3180	LOC_Os10g11810.1	10
3180	LOC_Os11g10520.1	11
3181	LOC_Os02g42520.1	2
3181	LOC_Os03g08999.1	3
3181	LOC_Os03g09020.1	3
3181	LOC_Os07g42924.1	7
3181	LOC_Os10g07229.1	10

3181	LOC_Os11g10510.1	11
3181	LOC_Os11g10480.1	11
3182	LOC_Os02g08270.1	2
3182	LOC_Os02g47850.1	2
3182	LOC_Os03g48060.1	3
3182	LOC_Os03g50880.1	3
3182	LOC_Os04g38950.1	4
3182	LOC_Os08g23730.1	8
3182	LOC_Os09g25610.1	9
3183	LOC_Os01g71240.1	1
3183	LOC_Os03g10640.1	3
3183	LOC_Os04g51610.1	4
3183	LOC_Os05g41580.1	5
3183	LOC_Os11g04460.1	11
3183	LOC_Os12g04220.1	12
3183	LOC_Os12g39660.1	12
3184	LOC_Os03g01120.1	3
3184	LOC_Os03g08560.1	3
3184	LOC_Os03g48310.1	3
3184	LOC_Os04g56160.1	4
3184	LOC_Os06g08310.1	6
3184	LOC_Os07g09340.1	7
3184	LOC_Os12g44150.1	12
3185	LOC_Os01g42430.1	1
3185	LOC_Os01g73130.1	1
3185	LOC_Os02g34510.1	2
3185	LOC_Os05g01560.1	5
3185	LOC_Os10g21230.1	10
3185	LOC_Os11g06890.1	11
3185	LOC_Os12g34110.1	12
3186	LOC_Os04g03380.1	4
3186	LOC_Os04g04690.1	4
3186	LOC_Os04g04770.1	4
3186	LOC_Os04g49480.1	4
3186	LOC_Os06g38160.1	6
3186	LOC_Os08g03190.1	8
3186	LOC_Os09g16700.1	9
3187	LOC_Os01g54300.1	1
3187	LOC_Os01g47400.1	1
3187	LOC_Os02g52800.1	2
3187	LOC_Os03g61280.1	3
3187	LOC_Os05g25480.1	5
3187	LOC_Os06g20620.1	6
3187	LOC_Os12g02520.1	12
3188	LOC_Os01g27520.1	1
3188	LOC_Os02g04700.1	2
3188	LOC_Os02g41470.1	2
3188	LOC_Os02g46130.1	2
3188	LOC_Os03g38980.1	3
3188	LOC_Os07g30200.1	7
3188	LOC_Os12g22600.1	12
3189	LOC_Os02g39330.1	2

3189	LOC_Os03g30470.1	3
3189	LOC_Os04g41680.1	4
3189	LOC_Os05g33150.1	5
3189	LOC_Os05g33140.1	5
3189	LOC_Os06g51050.1	6
3189	LOC_Os06g51060.1	6
3190	LOC_Os04g01740.1	4
3190	LOC_Os06g50300.1	6
3190	LOC_Os08g38086.1	8
3190	LOC_Os08g39140.1	8
3190	LOC_Os09g30438.1	9
3190	LOC_Os09g29840.1	9
3190	LOC_Os12g32986.1	12
3191	LOC_Os01g21160.1	1
3191	LOC_Os02g01500.1	2
3191	LOC_Os06g01630.1	6
3191	LOC_Os06g30460.1	6
3191	LOC_Os07g22720.1	7
3191	LOC_Os08g33440.1	8
3191	LOC_Os12g08170.1	12
3192	LOC_Os03g07570.1	3
3192	LOC_Os03g21960.1	3
3192	LOC_Os03g44150.1	3
3192	LOC_Os05g39770.1	5
3192	LOC_Os07g27780.1	7
3192	LOC_Os08g14770.1	8
3192	LOC_Os08g41990.1	8
3193	LOC_Os06g31060.1	6
3193	LOC_Os06g31070.1	6
3193	LOC_Os07g10570.1	7
3193	LOC_Os07g10580.1	7
3193	LOC_Os12g16880.1	12
3193	LOC_Os12g16890.1	12
3193	LOC_Os12g17010.1	12
3194	LOC_Os02g49250.1	2
3194	LOC_Os03g19630.1	3
3194	LOC_Os03g25304.1	3
3194	LOC_Os06g14010.1	6
3194	LOC_Os06g14700.1	6
3194	LOC_Os06g14710.1	6
3194	LOC_Os07g25370.1	7
3195	LOC_Os02g04640.1	2
3195	LOC_Os04g56990.1	4
3195	LOC_Os05g41240.1	5
3195	LOC_Os06g40710.1	6
3195	LOC_Os06g49040.1	6
3195	LOC_Os09g12770.1	9
3195	LOC_Os09g12750.1	9
3196	LOC_Os02g44870.1	2
3196	LOC_Os03g45280.1	3
3196	LOC_Os11g26570.1	11
3196	LOC_Os11g26760.1	11

3196	LOC_Os11g26780.1	11
3196	LOC_Os11g26790.1	11
3196	LOC_Os11g26750.1	11
3197	LOC_Os01g11370.1	1
3197	LOC_Os02g50370.1	2
3197	LOC_Os03g19960.1	3
3197	LOC_Os05g32370.1	5
3197	LOC_Os06g09280.1	6
3197	LOC_Os08g24760.1	8
3197	LOC_Os11g20554.1	11
3198	LOC_Os02g10120.1	2
3198	LOC_Os03g49350.1	3
3198	LOC_Os03g49380.1	3
3198	LOC_Os03g49260.1	3
3198	LOC_Os03g52860.1	3
3198	LOC_Os05g23880.1	5
3198	LOC_Os11g36719.1	11
3199	LOC_Os01g04300.1	1
3199	LOC_Os03g10440.1	3
3199	LOC_Os03g10478.1	3
3199	LOC_Os03g47010.1	3
3199	LOC_Os05g23350.1	5
3199	LOC_Os07g27320.1	7
3199	LOC_Os10g21100.1	10
3200	LOC_Os03g52040.1	3
3200	LOC_Os03g52070.1	3
3200	LOC_Os11g24340.1	11
3200	LOC_Os11g24510.1	11
3200	LOC_Os11g27264.1	11
3200	LOC_Os11g42390.1	11
3200	LOC_Os11g24389.1	11
3201	LOC_Os01g07950.1	1
3201	LOC_Os01g34620.1	1
3201	LOC_Os02g40500.1	2
3201	LOC_Os03g24030.1	3
3201	LOC_Os05g28530.1	5
3201	LOC_Os06g44910.1	6
3201	LOC_Os12g07650.1	12
3202	LOC_Os01g32964.1	1
3202	LOC_Os02g35910.1	2
3202	LOC_Os03g18970.1	3
3202	LOC_Os03g27780.1	3
3202	LOC_Os04g37660.1	4
3202	LOC_Os07g49040.1	7
3202	LOC_Os09g28560.1	9
3203	LOC_Os01g36390.1	1
3203	LOC_Os02g55410.1	2
3203	LOC_Os05g14590.1	5
3203	LOC_Os05g39850.1	5
3203	LOC_Os06g11500.1	6
3203	LOC_Os11g29380.1	11
3203	LOC_Os12g37400.1	12

3204	LOC_Os01g47600.1	1
3204	LOC_Os02g15810.1	2
3204	LOC_Os02g44930.1	2
3204	LOC_Os04g47690.1	4
3204	LOC_Os06g51220.1	6
3204	LOC_Os08g01100.1	8
3204	LOC_Os09g37910.1	9
3205	LOC_Os03g49210.1	3
3205	LOC_Os03g59650.1	3
3205	LOC_Os05g40810.1	5
3205	LOC_Os06g05190.1	6
3205	LOC_Os07g49010.1	7
3205	LOC_Os09g26870.1	9
3205	LOC_Os11g08660.1	11
3206	LOC_Os02g58480.1	2
3206	LOC_Os03g22120.1	3
3206	LOC_Os03g28330.1	3
3206	LOC_Os04g17650.1	4
3206	LOC_Os04g24430.1	4
3206	LOC_Os06g09450.1	6
3206	LOC_Os07g42490.1	7
3207	LOC_Os01g63990.1	1
3207	LOC_Os02g32970.1	2
3207	LOC_Os03g02514.1	3
3207	LOC_Os04g33600.1	4
3207	LOC_Os04g33590.1	4
3207	LOC_Os10g37710.1	10
3207	LOC_Os12g05600.1	12
3208	LOC_Os01g40640.1	1
3208	LOC_Os02g02390.1	2
3208	LOC_Os02g40460.1	2
3208	LOC_Os03g20100.1	3
3208	LOC_Os03g62780.1	3
3208	LOC_Os05g45920.1	5
3208	LOC_Os07g10350.1	7
3209	LOC_Os01g54020.1	1
3209	LOC_Os04g58400.1	4
3209	LOC_Os07g47860.1	7
3209	LOC_Os08g05490.1	8
3209	LOC_Os08g09260.1	8
3209	LOC_Os08g23110.1	8
3209	LOC_Os12g35570.1	12
3210	LOC_Os02g42940.1	2
3210	LOC_Os02g44300.1	2
3210	LOC_Os04g46780.1	4
3210	LOC_Os04g45190.1	4
3210	LOC_Os08g05890.1	8
3210	LOC_Os08g06020.1	8
3210	LOC_Os10g40140.1	10
3211	LOC_Os01g57920.1	1
3211	LOC_Os02g06520.1	2
3211	LOC_Os02g33310.1	2

3211	LOC_Os02g33400.1	2
3211	LOC_Os05g39300.1	5
3211	LOC_Os08g23960.1	8
3211	LOC_Os08g24140.1	8
3212	LOC_Os07g08570.1	7
3212	LOC_Os07g09814.1	7
3212	LOC_Os07g09870.1	7
3212	LOC_Os07g09950.1	7
3212	LOC_Os07g10710.1	7
3212	LOC_Os07g13870.1	7
3212	LOC_Os07g13890.1	7
3213	LOC_Os08g24370.1	8
3213	LOC_Os11g33190.1	11
3213	LOC_Os11g33220.1	11
3213	LOC_Os11g42230.1	11
3213	LOC_Os11g42240.1	11
3213	LOC_Os11g42280.1	11
3213	LOC_Os11g42300.1	11
3214	LOC_Os04g30810.1	4
3214	LOC_Os04g30830.1	4
3214	LOC_Os05g46380.1	5
3214	LOC_Os05g46390.1	5
3214	LOC_Os05g45990.1	5
3214	LOC_Os05g46300.1	5
3214	LOC_Os05g46320.1	5
3215	LOC_Os02g55550.1	2
3215	LOC_Os04g30200.1	4
3215	LOC_Os04g30210.1	4
3215	LOC_Os12g33210.1	12
3215	LOC_Os12g33220.1	12
3215	LOC_Os12g33230.1	12
3215	LOC_Os12g34220.1	12
3216	LOC_Os01g58580.1	1
3216	LOC_Os03g27170.1	3
3216	LOC_Os03g27190.1	3
3216	LOC_Os03g27120.1	3
3216	LOC_Os05g41660.1	5
3216	LOC_Os05g41670.1	5
3216	LOC_Os11g04010.1	11
3217	LOC_Os02g16709.1	2
3217	LOC_Os03g55640.1	3
3217	LOC_Os04g08340.1	4
3217	LOC_Os05g23260.1	5
3217	LOC_Os06g16260.1	6
3217	LOC_Os09g28000.1	9
3217	LOC_Os11g40500.1	11
3218	LOC_Os01g04200.1	1
3218	LOC_Os01g13150.1	1
3218	LOC_Os01g47690.1	1
3218	LOC_Os02g58270.1	2
3218	LOC_Os02g58260.1	2
3218	LOC_Os03g21460.1	3

3218	LOC_Os09g34100.1	9
3219	LOC_Os01g68890.1	1
3219	LOC_Os02g06660.1	2
3219	LOC_Os02g56580.1	2
3219	LOC_Os03g63060.1	3
3219	LOC_Os04g53580.1	4
3219	LOC_Os07g26480.1	7
3219	LOC_Os11g28310.1	11
3220	LOC_Os04g11970.1	4
3220	LOC_Os07g27880.1	7
3220	LOC_Os07g28040.1	7
3220	LOC_Os11g20080.1	11
3220	LOC_Os11g19790.1	11
3220	LOC_Os12g10140.1	12
3220	LOC_Os12g25860.1	12
3221	LOC_Os01g56800.1	1
3221	LOC_Os05g43280.1	5
3221	LOC_Os10g29460.1	10
3221	LOC_Os10g33830.1	10
3221	LOC_Os11g27030.1	11
3221	LOC_Os11g41360.1	11
3221	LOC_Os12g40520.1	12
3222	LOC_Os02g54820.1	2
3222	LOC_Os03g12360.1	3
3222	LOC_Os05g44210.1	5
3222	LOC_Os08g31980.1	8
3222	LOC_Os08g34580.1	8
3222	LOC_Os09g20990.1	9
3222	LOC_Os09g25890.1	9
3223	LOC_Os01g34390.1	1
3223	LOC_Os03g09060.1	3
3223	LOC_Os05g28200.1	5
3223	LOC_Os06g43880.1	6
3223	LOC_Os06g44840.1	6
3223	LOC_Os07g38850.1	7
3223	LOC_Os08g29910.1	8
3224	LOC_Os03g25940.1	3
3224	LOC_Os06g07860.1	6
3224	LOC_Os06g07960.1	6
3224	LOC_Os10g25930.1	10
3224	LOC_Os10g25950.1	10
3224	LOC_Os10g26010.1	10
3224	LOC_Os10g37340.1	10
3225	LOC_Os01g03950.1	1
3225	LOC_Os01g16310.1	1
3225	LOC_Os03g11720.1	3
3225	LOC_Os06g46340.1	6
3225	LOC_Os06g46284.1	6
3225	LOC_Os07g23944.1	7
3225	LOC_Os07g23880.1	7
3226	LOC_Os02g10260.1	2
3226	LOC_Os03g16440.1	3

3226	LOC_Os05g43470.1	5
3226	LOC_Os05g39390.1	5
3226	LOC_Os11g01490.1	11
3226	LOC_Os11g02460.1	11
3226	LOC_Os12g01500.1	12
3227	LOC_Os02g37000.1	2
3227	LOC_Os03g30790.1	3
3227	LOC_Os03g60000.1	3
3227	LOC_Os03g62490.1	3
3227	LOC_Os04g38900.1	4
3227	LOC_Os07g15880.1	7
3227	LOC_Os08g06200.1	8
3228	LOC_Os01g40560.1	1
3228	LOC_Os01g40580.1	1
3228	LOC_Os05g51420.1	5
3228	LOC_Os06g04460.1	6
3228	LOC_Os08g30790.1	8
3228	LOC_Os09g19710.1	9
3228	LOC_Os10g32700.1	10
3229	LOC_Os01g55360.1	1
3229	LOC_Os01g61560.1	1
3229	LOC_Os02g03460.1	2
3229	LOC_Os02g05320.1	2
3229	LOC_Os10g17990.1	10
3229	LOC_Os11g01450.1	11
3229	LOC_Os12g01480.1	12
3230	LOC_Os01g74520.1	1
3230	LOC_Os02g51030.1	2
3230	LOC_Os04g42870.1	4
3230	LOC_Os06g43800.1	6
3230	LOC_Os07g08200.1	7
3230	LOC_Os10g10180.1	10
3230	LOC_Os12g42090.1	12
3231	LOC_Os02g42230.1	2
3231	LOC_Os02g58220.1	2
3231	LOC_Os03g25130.1	3
3231	LOC_Os03g47990.1	3
3231	LOC_Os05g02030.1	5
3231	LOC_Os06g47830.1	6
3231	LOC_Os07g15500.1	7
3232	LOC_Os01g59850.1	1
3232	LOC_Os03g17020.1	3
3232	LOC_Os03g20720.1	3
3232	LOC_Os03g63710.1	3
3232	LOC_Os05g45840.1	5
3232	LOC_Os07g37650.1	7
3232	LOC_Os10g42420.1	10
3233	LOC_Os04g01250.1	4
3233	LOC_Os04g02780.1	4
3233	LOC_Os04g02754.1	4
3233	LOC_Os04g10434.1	4
3233	LOC_Os06g16030.1	6

3233	LOC_Os11g06900.1	11
3233	LOC_Os12g07150.1	12
3234	LOC_Os01g16910.1	1
3234	LOC_Os01g40570.1	1
3234	LOC_Os01g51770.1	1
3234	LOC_Os03g04460.1	3
3234	LOC_Os03g10510.1	3
3234	LOC_Os03g20750.1	3
3234	LOC_Os05g45950.1	5
3235	LOC_Os01g57390.1	1
3235	LOC_Os01g57400.1	1
3235	LOC_Os03g01990.1	3
3235	LOC_Os04g48380.1	4
3235	LOC_Os06g51360.1	6
3235	LOC_Os10g34420.1	10
3235	LOC_Os10g38040.1	10
3236	LOC_Os01g04660.1	1
3236	LOC_Os01g47580.1	1
3236	LOC_Os01g49820.1	1
3236	LOC_Os05g47660.1	5
3236	LOC_Os08g27030.1	8
3236	LOC_Os08g27040.1	8
3236	LOC_Os09g13870.1	9
3237	LOC_Os03g03390.1	3
3237	LOC_Os03g14860.1	3
3237	LOC_Os04g02500.1	4
3237	LOC_Os05g14040.1	5
3237	LOC_Os08g19170.1	8
3237	LOC_Os08g39880.1	8
3237	LOC_Os10g35000.1	10
3238	LOC_Os03g17790.1	3
3238	LOC_Os03g25460.1	3
3238	LOC_Os05g03130.1	5
3238	LOC_Os05g04700.1	5
3238	LOC_Os06g44220.1	6
3238	LOC_Os07g44180.1	7
3238	LOC_Os09g38560.1	9
3239	LOC_Os01g65590.1	1
3239	LOC_Os02g06840.1	2
3239	LOC_Os02g35870.1	2
3239	LOC_Os03g38050.1	3
3239	LOC_Os05g35274.1	5
3239	LOC_Os06g46570.1	6
3239	LOC_Os09g27950.1	9
3240	LOC_Os01g07810.1	1
3240	LOC_Os02g10810.1	2
3240	LOC_Os04g34940.1	4
3240	LOC_Os06g40040.1	6
3240	LOC_Os09g37006.1	9
3240	LOC_Os11g06760.1	11
3240	LOC_Os12g30550.1	12
3241	LOC_Os01g07960.1	1

3241	LOC_Os01g42690.1	1
3241	LOC_Os04g09540.1	4
3241	LOC_Os04g57370.1	4
3241	LOC_Os04g57380.1	4
3241	LOC_Os04g57390.1	4
3241	LOC_Os05g51050.1	5
3242	LOC_Os02g51450.1	2
3242	LOC_Os02g51460.1	2
3242	LOC_Os04g54510.1	4
3242	LOC_Os06g12080.1	6
3242	LOC_Os06g12040.1	6
3242	LOC_Os06g12100.1	6
3242	LOC_Os06g12110.1	6
3243	LOC_Os06g12050.1	6
3243	LOC_Os06g12060.1	6
3243	LOC_Os06g12070.1	6
3243	LOC_Os11g10040.1	11
3243	LOC_Os11g09990.1	11
3243	LOC_Os11g10000.1	11
3243	LOC_Os11g14130.1	11
3244	LOC_Os01g58830.1	1
3244	LOC_Os02g25950.1	2
3244	LOC_Os03g36550.1	3
3244	LOC_Os03g52310.1	3
3244	LOC_Os05g14940.1	5
3244	LOC_Os05g51300.1	5
3244	LOC_Os06g24530.1	6
3245	LOC_Os02g45820.1	2
3245	LOC_Os04g49220.1	4
3245	LOC_Os05g02050.1	5
3245	LOC_Os08g42380.1	8
3245	LOC_Os11g03710.1	11
3245	LOC_Os12g03460.1	12
3245	LOC_Os12g38310.1	12
3246	LOC_Os01g11810.1	1
3246	LOC_Os01g65800.1	1
3246	LOC_Os05g35190.1	5
3246	LOC_Os06g33330.1	6
3246	LOC_Os07g15230.1	7
3246	LOC_Os07g31550.1	7
3246	LOC_Os11g05080.1	11
3247	LOC_Os01g01780.1	1
3247	LOC_Os01g45350.1	1
3247	LOC_Os02g39960.1	2
3247	LOC_Os04g01930.1	4
3247	LOC_Os06g07887.1	6
3247	LOC_Os06g07987.1	6
3247	LOC_Os06g46690.1	6
3248	LOC_Os01g23580.1	1
3248	LOC_Os02g09150.1	2
3248	LOC_Os02g33490.1	2
3248	LOC_Os02g55890.1	2

3248	LOC_Os05g06480.1	5
3248	LOC_Os06g08080.1	6
3248	LOC_Os06g43660.1	6
3249	LOC_Os01g26804.1	1
3249	LOC_Os01g61800.1	1
3249	LOC_Os06g45920.1	6
3249	LOC_Os07g08240.1	7
3249	LOC_Os11g11530.1	11
3249	LOC_Os12g29370.1	12
3249	LOC_Os12g32040.1	12
3250	LOC_Os02g58070.1	2
3250	LOC_Os03g06990.1	3
3250	LOC_Os03g55380.1	3
3250	LOC_Os03g58890.1	3
3250	LOC_Os04g27850.1	4
3250	LOC_Os05g41010.1	5
3250	LOC_Os10g35470.1	10
3251	LOC_Os02g42890.1	2
3251	LOC_Os03g50820.1	3
3251	LOC_Os04g45090.1	4
3251	LOC_Os08g01140.1	8
3251	LOC_Os08g29520.1	8
3251	LOC_Os10g02920.1	10
3251	LOC_Os10g36030.1	10
3252	LOC_Os04g44780.1	4
3252	LOC_Os06g47360.1	6
3252	LOC_Os07g31390.1	7
3252	LOC_Os08g37930.1	8
3252	LOC_Os09g29690.1	9
3252	LOC_Os09g29740.1	9
3252	LOC_Os09g29710.1	9
3253	LOC_Os01g65830.1	1
3253	LOC_Os01g69080.1	1
3253	LOC_Os03g30950.1	3
3253	LOC_Os03g53010.1	3
3253	LOC_Os04g31070.1	4
3253	LOC_Os08g10010.1	8
3253	LOC_Os08g09950.1	8
3254	LOC_Os01g55580.1	1
3254	LOC_Os03g43730.1	3
3254	LOC_Os05g43380.1	5
3254	LOC_Os05g51040.1	5
3254	LOC_Os07g07974.1	7
3254	LOC_Os08g28214.1	8
3254	LOC_Os12g41230.1	12
3255	LOC_Os03g01880.1	3
3255	LOC_Os03g39010.1	3
3255	LOC_Os03g49050.1	3
3255	LOC_Os04g43840.1	4
3255	LOC_Os05g46360.1	5
3255	LOC_Os05g51390.1	5
3255	LOC_Os10g33900.1	10

3256	LOC_Os01g56420.1	1
3256	LOC_Os01g56430.1	1
3256	LOC_Os03g25470.1	3
3256	LOC_Os04g33900.1	4
3256	LOC_Os05g35050.1	5
3256	LOC_Os08g35490.1	8
3256	LOC_Os09g26900.1	9
3257	LOC_Os01g21190.1	1
3257	LOC_Os03g52740.1	3
3257	LOC_Os04g30900.1	4
3257	LOC_Os07g45180.1	7
3257	LOC_Os07g45220.1	7
3257	LOC_Os09g11820.1	9
3257	LOC_Os11g32850.1	11
3258	LOC_Os04g06590.1	4
3258	LOC_Os04g06990.1	4
3258	LOC_Os04g07250.1	4
3258	LOC_Os04g08080.1	4
3258	LOC_Os04g08190.1	4
3258	LOC_Os04g08200.1	4
3258	LOC_Os12g21940.1	12
3259	LOC_Os02g19060.1	2
3259	LOC_Os08g05770.1	8
3259	LOC_Os08g05780.1	8
3259	LOC_Os08g28970.1	8
3259	LOC_Os08g40940.1	8
3259	LOC_Os09g15780.1	9
3259	LOC_Os09g32100.1	9
3260	LOC_Os02g42950.1	2
3260	LOC_Os03g44710.1	3
3260	LOC_Os03g11600.1	3
3260	LOC_Os04g45330.1	4
3260	LOC_Os07g06620.1	7
3260	LOC_Os10g36420.1	10
3260	LOC_Os12g42610.1	12
3261	LOC_Os02g36510.1	2
3261	LOC_Os03g20780.1	3
3261	LOC_Os03g20790.1	3
3261	LOC_Os04g38400.1	4
3261	LOC_Os07g48630.1	7
3261	LOC_Os08g39830.1	8
3261	LOC_Os09g31400.1	9
3262	LOC_Os01g28200.1	1
3262	LOC_Os01g52430.1	1
3262	LOC_Os05g16730.1	5
3262	LOC_Os11g30590.1	11
3262	LOC_Os12g35120.1	12
3262	LOC_Os12g35220.1	12
3262	LOC_Os12g35250.1	12
3263	LOC_Os01g69050.1	1
3263	LOC_Os06g51180.1	6
3263	LOC_Os06g51190.1	6

3263	LOC_Os06g51200.1	6
3263	LOC_Os06g51520.1	6
3263	LOC_Os07g23190.1	7
3263	LOC_Os07g37760.1	7
3264	LOC_Os03g04380.1	3
3264	LOC_Os05g30030.1	5
3264	LOC_Os06g50000.1	6
3264	LOC_Os07g44650.1	7
3264	LOC_Os08g14070.1	8
3264	LOC_Os08g17650.1	8
3264	LOC_Os10g26640.1	10
3265	LOC_Os01g13600.1	1
3265	LOC_Os02g47610.1	2
3265	LOC_Os03g15360.1	3
3265	LOC_Os06g28550.1	6
3265	LOC_Os06g49120.1	6
3265	LOC_Os12g16240.1	12
3265	LOC_Os12g16250.1	12
3266	LOC_Os01g14530.1	1
3266	LOC_Os01g50440.1	1
3266	LOC_Os02g49860.1	2
3266	LOC_Os05g31670.1	5
3266	LOC_Os05g33220.1	5
3266	LOC_Os07g24000.1	7
3266	LOC_Os10g32720.1	10
3267	LOC_Os02g09630.1	2
3267	LOC_Os02g49340.1	2
3267	LOC_Os03g63140.1	3
3267	LOC_Os04g31030.1	4
3267	LOC_Os04g52950.1	4
3267	LOC_Os06g17870.1	6
3267	LOC_Os09g07920.1	9
3268	LOC_Os02g32750.1	2
3268	LOC_Os02g49140.1	2
3268	LOC_Os03g18820.1	3
3268	LOC_Os03g19310.1	3
3268	LOC_Os03g19330.1	3
3268	LOC_Os11g34390.1	11
3268	LOC_Os12g05380.1	12
3269	LOC_Os01g13070.1	1
3269	LOC_Os02g44040.1	2
3269	LOC_Os03g43510.1	3
3269	LOC_Os10g41060.1	10
3269	LOC_Os10g41860.1	10
3269	LOC_Os10g41870.1	10
3269	LOC_Os12g41140.1	12
3270	LOC_Os02g20940.1	2
3270	LOC_Os03g18420.1	3
3270	LOC_Os03g48080.1	3
3270	LOC_Os04g44550.1	4
3270	LOC_Os05g01300.1	5
3270	LOC_Os07g09130.1	7

3270	LOC_Os12g44080.1	12
3271	LOC_Os02g27430.1	2
3271	LOC_Os03g41580.1	3
3271	LOC_Os09g03150.1	9
3271	LOC_Os09g08600.1	9
3271	LOC_Os11g32670.1	11
3271	LOC_Os11g41630.1	11
3271	LOC_Os12g14350.1	12
3272	LOC_Os01g34470.1	1
3272	LOC_Os01g34770.1	1
3272	LOC_Os01g72280.1	1
3272	LOC_Os02g03470.1	2
3272	LOC_Os03g24060.1	3
3272	LOC_Os05g31770.1	5
3272	LOC_Os12g38380.1	12
3273	LOC_Os05g16110.1	5
3273	LOC_Os05g16140.1	5
3273	LOC_Os05g16000.1	5
3273	LOC_Os05g39190.1	5
3273	LOC_Os05g39120.1	5
3273	LOC_Os05g39150.1	5
3273	LOC_Os05g39180.1	5
3274	LOC_Os06g20200.1	6
3274	LOC_Os07g44860.1	7
3274	LOC_Os07g44900.1	7
3274	LOC_Os07g44910.1	7
3274	LOC_Os11g13570.1	11
3274	LOC_Os11g13630.1	11
3274	LOC_Os11g13670.1	11
3275	LOC_Os01g28360.1	1
3275	LOC_Os01g29020.1	1
3275	LOC_Os02g23860.1	2
3275	LOC_Os03g35769.1	3
3275	LOC_Os05g22530.1	5
3275	LOC_Os09g16370.1	9
3275	LOC_Os11g17780.1	11
3276	LOC_Os04g07430.1	4
3276	LOC_Os05g24410.1	5
3276	LOC_Os05g12550.1	5
3276	LOC_Os06g27450.1	6
3276	LOC_Os08g19040.1	8
3276	LOC_Os11g11840.1	11
3276	LOC_Os12g33740.1	12
3277	LOC_Os06g15260.1	6
3277	LOC_Os06g23240.1	6
3277	LOC_Os06g47070.1	6
3277	LOC_Os08g30360.1	8
3277	LOC_Os10g01440.1	10
3277	LOC_Os11g28590.1	11
3277	LOC_Os11g29330.1	11
3278	LOC_Os10g39470.1	10
3278	LOC_Os10g39510.1	10

3278	LOC_Os11g42880.1	11
3278	LOC_Os11g42810.1	11
3278	LOC_Os11g42930.1	11
3278	LOC_Os11g42890.1	11
3278	LOC_Os11g42850.1	11
3279	LOC_Os01g50350.1	1
3279	LOC_Os05g46790.1	5
3279	LOC_Os05g46830.1	5
3279	LOC_Os05g46840.1	5
3279	LOC_Os09g27170.1	9
3279	LOC_Os10g36620.1	10
3279	LOC_Os11g10890.1	11
3280	LOC_Os06g11870.1	6
3280	LOC_Os06g23850.1	6
3280	LOC_Os06g34080.1	6
3280	LOC_Os07g28100.1	7
3280	LOC_Os08g36350.1	8
3280	LOC_Os10g37459.1	10
3280	LOC_Os12g10490.1	12
3281	LOC_Os04g28500.1	4
3281	LOC_Os05g15350.1	5
3281	LOC_Os07g11370.1	7
3281	LOC_Os09g26070.1	9
3281	LOC_Os10g13950.1	10
3281	LOC_Os12g29714.1	12
3281	LOC_Os12g29730.1	12
3282	LOC_Os01g05330.1	1
3282	LOC_Os01g26848.1	1
3282	LOC_Os01g54280.1	1
3282	LOC_Os02g29810.1	2
3282	LOC_Os04g50900.1	4
3282	LOC_Os10g29450.1	10
3282	LOC_Os10g37950.1	10
3283	LOC_Os06g50490.1	6
3283	LOC_Os06g50539.1	6
3283	LOC_Os06g50580.1	6
3283	LOC_Os06g50670.1	6
3283	LOC_Os06g50706.1	6
3283	LOC_Os06g50742.1	6
3283	LOC_Os06g50818.1	6
3284	LOC_Os03g31950.1	3
3284	LOC_Os07g13570.1	7
3284	LOC_Os07g36220.1	7
3284	LOC_Os08g01990.1	8
3284	LOC_Os08g17220.1	8
3284	LOC_Os10g02780.1	10
3284	LOC_Os12g11790.1	12
3285	LOC_Os01g32540.1	1
3285	LOC_Os02g37110.1	2
3285	LOC_Os03g27050.1	3
3285	LOC_Os05g38910.1	5
3285	LOC_Os10g31680.1	10

3285	LOC_Os10g31720.1	10
3285	LOC_Os10g31730.1	10
3286	LOC_Os01g03350.1	1
3286	LOC_Os01g32460.1	1
3286	LOC_Os06g42110.1	6
3286	LOC_Os07g14260.1	7
3286	LOC_Os07g40230.1	7
3286	LOC_Os08g15860.1	8
3286	LOC_Os10g09150.1	10
3287	LOC_Os05g06540.1	5
3287	LOC_Os05g06630.1	5
3287	LOC_Os05g06650.1	5
3287	LOC_Os05g06814.1	5
3287	LOC_Os05g06910.1	5
3287	LOC_Os07g12810.1	7
3287	LOC_Os08g04920.1	8
3288	LOC_Os02g34210.1	2
3288	LOC_Os03g45480.1	3
3288	LOC_Os04g22180.1	4
3288	LOC_Os04g27550.1	4
3288	LOC_Os09g16600.1	9
3288	LOC_Os09g20060.1	9
3288	LOC_Os11g26290.1	11
3289	LOC_Os01g09680.1	1
3289	LOC_Os04g08660.1	4
3289	LOC_Os05g27670.1	5
3289	LOC_Os09g11160.1	9
3289	LOC_Os10g07080.1	10
3289	LOC_Os11g39380.1	11
3289	LOC_Os12g36960.1	12
3290	LOC_Os10g04380.1	10
3290	LOC_Os10g04540.1	10
3290	LOC_Os10g04450.1	10
3290	LOC_Os10g04470.1	10
3290	LOC_Os10g04510.1	10
3290	LOC_Os10g04520.1	10
3290	LOC_Os11g06300.1	11
3291	LOC_Os05g30660.1	5
3291	LOC_Os05g30680.1	5
3291	LOC_Os05g30700.1	5
3291	LOC_Os05g30620.1	5
3291	LOC_Os05g30640.1	5
3291	LOC_Os08g13270.1	8
3291	LOC_Os09g17810.1	9
3292	LOC_Os01g27834.1	1
3292	LOC_Os04g11900.1	4
3292	LOC_Os04g22770.1	4
3292	LOC_Os04g23100.1	4
3292	LOC_Os07g16054.1	7
3292	LOC_Os12g13690.1	12
3292	LOC_Os12g24519.1	12
3293	LOC_Os04g34830.1	4

3293	LOC_Os06g36960.1	6
3293	LOC_Os07g01280.1	7
3293	LOC_Os07g12990.1	7
3293	LOC_Os09g25520.1	9
3293	LOC_Os11g12510.1	11
3293	LOC_Os12g24410.1	12
3294	LOC_Os03g08130.1	3
3294	LOC_Os05g47520.1	5
3294	LOC_Os06g50430.1	6
3294	LOC_Os06g50639.1	6
3294	LOC_Os06g50789.1	6
3294	LOC_Os07g15430.1	7
3294	LOC_Os10g32010.1	10
3295	LOC_Os01g43370.1	1
3295	LOC_Os02g15880.1	2
3295	LOC_Os02g36590.1	2
3295	LOC_Os04g38520.1	4
3295	LOC_Os04g54830.1	4
3295	LOC_Os06g27830.1	6
3295	LOC_Os06g33180.1	6
3296	LOC_Os01g27680.1	1
3296	LOC_Os01g71250.1	1
3296	LOC_Os03g57830.1	3
3296	LOC_Os07g08040.1	7
3296	LOC_Os08g18870.1	8
3296	LOC_Os08g25670.1	8
3296	LOC_Os11g34790.1	11
3297	LOC_Os02g05240.1	2
3297	LOC_Os02g36250.1	2
3297	LOC_Os04g11750.1	4
3297	LOC_Os05g38070.1	5
3297	LOC_Os10g28094.1	10
3297	LOC_Os11g02940.1	11
3297	LOC_Os12g20260.1	12
3298	LOC_Os02g26580.1	2
3298	LOC_Os12g23820.1	12
3298	LOC_Os12g32050.1	12
3298	LOC_Os12g32060.1	12
3298	LOC_Os12g32080.1	12
3298	LOC_Os12g32150.1	12
3298	LOC_Os12g32170.1	12
3299	LOC_Os01g15240.1	1
3299	LOC_Os01g23940.1	1
3299	LOC_Os01g60710.1	1
3299	LOC_Os01g71570.1	1
3299	LOC_Os01g71490.1	1
3299	LOC_Os07g14000.1	7
3299	LOC_Os11g39960.1	11
3300	LOC_Os01g57180.1	1
3300	LOC_Os01g73920.1	1
3300	LOC_Os01g74380.1	1
3300	LOC_Os03g61460.1	3

3300	LOC_Os04g24274.1	4
3300	LOC_Os04g48300.1	4
3300	LOC_Os05g35610.1	5
3301	LOC_Os01g49510.1	1
3301	LOC_Os02g09770.1	2
3301	LOC_Os02g55330.1	2
3301	LOC_Os06g42730.1	6
3301	LOC_Os08g06420.1	8
3301	LOC_Os08g38092.1	8
3301	LOC_Os12g18860.1	12
3302	LOC_Os01g24440.1	1
3302	LOC_Os02g22120.1	2
3302	LOC_Os02g24810.1	2
3302	LOC_Os06g35970.1	6
3302	LOC_Os11g16800.1	11
3302	LOC_Os11g30320.1	11
3302	LOC_Os12g41610.1	12
3303	LOC_Os02g45900.1	2
3303	LOC_Os04g49270.1	4
3303	LOC_Os04g49310.1	4
3303	LOC_Os04g49320.1	4
3303	LOC_Os04g49340.1	4
3303	LOC_Os04g51090.1	4
3303	LOC_Os10g39230.1	10
3304	LOC_Os01g35980.1	1
3304	LOC_Os03g49860.1	3
3304	LOC_Os03g61860.1	3
3304	LOC_Os12g03220.1	12
3304	LOC_Os12g09360.1	12
3304	LOC_Os12g12250.1	12
3304	LOC_Os12g16600.1	12
3305	LOC_Os01g36950.1	1
3305	LOC_Os02g51140.1	2
3305	LOC_Os05g41930.1	5
3305	LOC_Os05g51590.1	5
3305	LOC_Os06g12260.1	6
3305	LOC_Os09g39020.1	9
3305	LOC_Os11g32270.1	11
3306	LOC_Os03g06780.1	3
3306	LOC_Os03g06790.1	3
3306	LOC_Os03g06800.1	3
3306	LOC_Os03g06870.1	3
3306	LOC_Os03g06810.1	3
3306	LOC_Os03g06820.1	3
3306	LOC_Os03g06830.1	3
3307	LOC_Os01g18670.1	1
3307	LOC_Os01g34970.1	1
3307	LOC_Os01g35030.1	1
3307	LOC_Os01g50080.1	1
3307	LOC_Os01g50160.1	1
3307	LOC_Os02g21750.1	2
3307	LOC_Os05g47490.1	5

3307	LOC_Os05g47500.1	5
3308	LOC_Os01g03144.1	1
3308	LOC_Os01g42900.1	1
3308	LOC_Os03g06139.1	3
3308	LOC_Os06g30730.1	6
3308	LOC_Os06g40550.1	6
3308	LOC_Os08g07010.1	8
3308	LOC_Os09g23640.1	9
3308	LOC_Os11g07600.1	11
3309	LOC_Os04g45270.1	4
3309	LOC_Os07g34850.1	7
3309	LOC_Os08g36540.1	8
3309	LOC_Os09g38380.1	9
3309	LOC_Os10g39350.1	10
3309	LOC_Os10g39380.1	10
3309	LOC_Os10g39260.1	10
3309	LOC_Os10g39300.1	10
3310	LOC_Os02g45250.1	2
3310	LOC_Os04g48070.1	4
3310	LOC_Os04g53540.1	4
3310	LOC_Os06g10600.1	6
3310	LOC_Os08g04190.1	8
3310	LOC_Os08g08820.1	8
3310	LOC_Os09g35760.1	9
3310	LOC_Os10g42490.1	10
3311	LOC_Os07g03810.1	7
3311	LOC_Os07g03830.1	7
3311	LOC_Os07g03900.1	7
3311	LOC_Os07g03850.1	7
3311	LOC_Os07g03860.1	7
3311	LOC_Os07g03870.1	7
3311	LOC_Os07g03880.1	7
3311	LOC_Os07g38820.1	7
3312	LOC_Os01g57940.1	1
3312	LOC_Os02g57560.1	2
3312	LOC_Os03g38710.1	3
3312	LOC_Os03g56470.1	3
3312	LOC_Os05g01040.1	5
3312	LOC_Os06g44430.1	6
3312	LOC_Os09g14580.1	9
3312	LOC_Os09g36320.1	9
3313	LOC_Os01g41730.1	1
3313	LOC_Os02g06160.1	2
3313	LOC_Os06g07070.1	6
3313	LOC_Os08g03040.1	8
3313	LOC_Os09g16590.1	9
3313	LOC_Os09g19380.1	9
3313	LOC_Os09g29540.1	9
3313	LOC_Os12g41710.1	12
3314	LOC_Os02g53910.1	2
3314	LOC_Os02g53970.1	2
3314	LOC_Os03g06290.1	3

3314	LOC_Os04g03060.1	4
3314	LOC_Os04g10360.1	4
3314	LOC_Os09g30250.1	9
3314	LOC_Os11g15520.1	11
3314	LOC_Os12g23980.1	12
3315	LOC_Os01g65080.1	1
3315	LOC_Os02g36360.1	2
3315	LOC_Os03g62230.1	3
3315	LOC_Os04g08290.1	4
3315	LOC_Os08g44830.1	8
3315	LOC_Os09g13680.1	9
3315	LOC_Os09g31150.1	9
3315	LOC_Os09g38340.1	9
3316	LOC_Os04g23930.1	4
3316	LOC_Os05g19764.1	5
3316	LOC_Os06g21130.1	6
3316	LOC_Os07g20300.1	7
3316	LOC_Os07g20360.1	7
3316	LOC_Os09g12490.1	9
3316	LOC_Os12g12400.1	12
3316	LOC_Os12g15140.1	12
3317	LOC_Os04g16270.1	4
3317	LOC_Os05g16080.1	5
3317	LOC_Os05g11520.1	5
3317	LOC_Os07g19090.1	7
3317	LOC_Os08g16390.1	8
3317	LOC_Os10g24150.1	10
3317	LOC_Os11g18700.1	11
3317	LOC_Os12g17390.1	12
3318	LOC_Os02g24520.1	2
3318	LOC_Os03g41840.1	3
3318	LOC_Os04g12410.1	4
3318	LOC_Os05g19460.1	5
3318	LOC_Os06g28490.1	6
3318	LOC_Os09g07220.1	9
3318	LOC_Os09g07400.1	9
3318	LOC_Os11g29770.1	11
3319	LOC_Os01g70430.1	1
3319	LOC_Os02g42790.1	2
3319	LOC_Os02g42810.1	2
3319	LOC_Os04g44950.1	4
3319	LOC_Os04g44980.1	4
3319	LOC_Os04g45000.1	4
3319	LOC_Os04g44920.1	4
3319	LOC_Os07g48640.1	7
3320	LOC_Os02g09490.1	2
3320	LOC_Os04g15920.1	4
3320	LOC_Os04g52280.1	4
3320	LOC_Os08g16910.1	8
3320	LOC_Os09g23530.1	9
3320	LOC_Os09g23560.1	9
3320	LOC_Os10g29470.1	10

3320	LOC_Os11g40690.1	11
3321	LOC_Os01g03020.1	1
3321	LOC_Os02g09100.1	2
3321	LOC_Os02g09120.1	2
3321	LOC_Os02g53770.1	2
3321	LOC_Os06g43760.1	6
3321	LOC_Os09g21110.1	9
3321	LOC_Os09g32650.1	9
3321	LOC_Os10g26050.1	10
3322	LOC_Os01g58640.1	1
3322	LOC_Os03g11530.1	3
3322	LOC_Os08g41880.1	8
3322	LOC_Os09g32830.1	9
3322	LOC_Os09g32840.1	9
3322	LOC_Os12g38750.1	12
3322	LOC_Os12g38760.1	12
3322	LOC_Os12g38770.1	12
3323	LOC_Os01g06740.1	1
3323	LOC_Os02g05590.1	2
3323	LOC_Os03g45120.1	3
3323	LOC_Os07g37090.1	7
3323	LOC_Os10g24050.1	10
3323	LOC_Os11g06630.1	11
3323	LOC_Os12g07520.1	12
3323	LOC_Os12g35010.1	12
3324	LOC_Os01g14580.1	1
3324	LOC_Os01g16900.1	1
3324	LOC_Os01g46610.1	1
3324	LOC_Os02g38200.1	2
3324	LOC_Os03g45320.1	3
3324	LOC_Os04g40310.1	4
3324	LOC_Os04g42920.1	4
3324	LOC_Os05g49760.1	5
3325	LOC_Os01g18400.1	1
3325	LOC_Os03g04060.1	3
3325	LOC_Os04g41620.1	4
3325	LOC_Os05g04690.1	5
3325	LOC_Os08g41100.1	8
3325	LOC_Os09g32080.1	9
3325	LOC_Os10g39680.1	10
3325	LOC_Os10g39700.1	10
3326	LOC_Os01g06660.1	1
3326	LOC_Os01g32080.1	1
3326	LOC_Os02g30630.1	2
3326	LOC_Os04g32010.1	4
3326	LOC_Os04g31960.1	4
3326	LOC_Os05g39320.1	5
3326	LOC_Os05g39310.1	5
3326	LOC_Os07g49250.1	7
3327	LOC_Os01g13760.1	1
3327	LOC_Os01g65480.1	1
3327	LOC_Os02g03600.1	2

3327	LOC_Os02g20394.1	2
3327	LOC_Os05g03630.1	5
3327	LOC_Os05g48810.1	5
3327	LOC_Os08g06460.1	8
3327	LOC_Os08g28700.1	8
3328	LOC_Os01g03310.1	1
3328	LOC_Os01g03340.1	1
3328	LOC_Os01g03360.1	1
3328	LOC_Os01g03680.1	1
3328	LOC_Os01g03320.1	1
3328	LOC_Os01g03330.1	1
3328	LOC_Os01g03380.1	1
3328	LOC_Os01g03390.1	1
3329	LOC_Os01g11110.1	1
3329	LOC_Os02g36974.1	2
3329	LOC_Os03g50290.1	3
3329	LOC_Os04g38870.1	4
3329	LOC_Os08g33370.1	8
3329	LOC_Os08g37490.1	8
3329	LOC_Os11g34450.1	11
3329	LOC_Os11g39540.1	11
3330	LOC_Os01g02880.1	1
3330	LOC_Os01g67860.1	1
3330	LOC_Os05g33380.1	5
3330	LOC_Os06g40640.1	6
3330	LOC_Os08g02700.1	8
3330	LOC_Os10g08022.1	10
3330	LOC_Os11g07020.1	11
3330	LOC_Os12g07210.1	12
3331	LOC_Os03g13070.1	3
3331	LOC_Os03g46060.1	3
3331	LOC_Os06g47600.1	6
3331	LOC_Os10g05600.1	10
3331	LOC_Os11g47680.1	11
3331	LOC_Os11g47670.1	11
3331	LOC_Os12g38120.1	12
3331	LOC_Os12g43450.1	12
3332	LOC_Os01g01030.1	1
3332	LOC_Os01g60080.1	1
3332	LOC_Os04g47400.1	4
3332	LOC_Os05g40740.1	5
3332	LOC_Os06g01490.1	6
3332	LOC_Os06g46500.1	6
3332	LOC_Os07g32660.1	7
3332	LOC_Os10g36390.1	10
3333	LOC_Os02g10380.1	2
3333	LOC_Os02g34860.1	2
3333	LOC_Os02g38350.1	2
3333	LOC_Os03g40260.1	3
3333	LOC_Os04g35570.1	4
3333	LOC_Os07g27490.1	7
3333	LOC_Os08g33390.1	8

3333	LOC_Os12g18650.1	12
3334	LOC_Os01g67180.1	1
3334	LOC_Os01g67190.1	1
3334	LOC_Os07g43600.1	7
3334	LOC_Os07g43640.1	7
3334	LOC_Os07g43670.1	7
3334	LOC_Os08g33710.1	8
3334	LOC_Os09g36700.1	9
3334	LOC_Os09g36680.1	9
3335	LOC_Os02g07350.1	2
3335	LOC_Os02g56170.1	2
3335	LOC_Os03g39000.1	3
3335	LOC_Os07g09330.1	7
3335	LOC_Os07g37220.1	7
3335	LOC_Os07g37230.1	7
3335	LOC_Os12g08270.1	12
3335	LOC_Os12g08280.1	12
3336	LOC_Os01g44950.1	1
3336	LOC_Os02g32490.1	2
3336	LOC_Os03g59080.1	3
3336	LOC_Os03g62850.1	3
3336	LOC_Os04g33190.1	4
3336	LOC_Os05g50300.1	5
3336	LOC_Os06g02160.1	6
3336	LOC_Os09g21230.1	9
3337	LOC_Os03g22950.1	3
3337	LOC_Os03g63930.1	3
3337	LOC_Os05g31290.1	5
3337	LOC_Os07g12150.1	7
3337	LOC_Os08g43580.1	8
3337	LOC_Os09g36860.1	9
3337	LOC_Os11g31900.1	11
3337	LOC_Os12g34890.1	12
3338	LOC_Os05g04750.1	5
3338	LOC_Os05g08010.1	5
3338	LOC_Os05g08350.1	5
3338	LOC_Os05g08460.1	5
3338	LOC_Os05g08440.1	5
3338	LOC_Os07g35050.1	7
3338	LOC_Os07g35060.1	7
3338	LOC_Os07g35130.1	7
3339	LOC_Os02g54550.1	2
3339	LOC_Os02g55050.1	2
3339	LOC_Os02g57860.1	2
3339	LOC_Os02g57890.1	2
3339	LOC_Os02g57910.1	2
3339	LOC_Os02g58040.1	2
3339	LOC_Os04g40770.1	4
3339	LOC_Os05g01620.1	5
3340	LOC_Os08g38520.1	8
3340	LOC_Os08g38480.1	8
3340	LOC_Os08g38490.1	8

3340	LOC_Os09g30180.1	9
3340	LOC_Os09g34200.1	9
3340	LOC_Os12g30920.1	12
3340	LOC_Os12g30970.1	12
3340	LOC_Os12g30990.1	12
3341	LOC_Os02g20690.1	2
3341	LOC_Os08g03480.1	8
3341	LOC_Os08g03490.1	8
3341	LOC_Os08g31450.1	8
3341	LOC_Os10g29740.1	10
3341	LOC_Os10g29850.1	10
3341	LOC_Os11g40220.1	11
3341	LOC_Os11g45560.1	11
3342	LOC_Os01g64670.1	1
3342	LOC_Os01g74350.1	1
3342	LOC_Os02g47600.1	2
3342	LOC_Os02g52940.1	2
3342	LOC_Os04g59040.1	4
3342	LOC_Os05g02310.1	5
3342	LOC_Os05g36260.1	5
3342	LOC_Os10g26600.1	10
3343	LOC_Os02g33080.1	2
3343	LOC_Os02g42350.1	2
3343	LOC_Os02g42330.1	2
3343	LOC_Os03g07910.1	3
3343	LOC_Os06g10420.1	6
3343	LOC_Os07g07260.1	7
3343	LOC_Os07g30170.1	7
3343	LOC_Os12g31830.1	12
3344	LOC_Os01g27160.1	1
3344	LOC_Os01g27150.1	1
3344	LOC_Os01g50980.1	1
3344	LOC_Os02g51180.1	2
3344	LOC_Os03g57290.1	3
3344	LOC_Os04g55030.1	4
3344	LOC_Os05g05700.1	5
3344	LOC_Os08g07400.1	8
3345	LOC_Os01g36560.1	1
3345	LOC_Os01g58910.1	1
3345	LOC_Os02g52930.1	2
3345	LOC_Os04g59120.1	4
3345	LOC_Os06g10750.1	6
3345	LOC_Os07g34110.1	7
3345	LOC_Os10g14920.1	10
3345	LOC_Os12g33300.1	12
3346	LOC_Os01g19290.1	1
3346	LOC_Os03g05530.1	3
3346	LOC_Os04g34490.1	4
3346	LOC_Os05g41420.1	5
3346	LOC_Os06g01840.1	6
3346	LOC_Os10g07998.1	10
3346	LOC_Os10g12190.1	10

3346	LOC_Os12g18960.1	12
3347	LOC_Os02g55990.1	2
3347	LOC_Os03g06960.1	3
3347	LOC_Os03g58840.1	3
3347	LOC_Os06g07780.1	6
3347	LOC_Os07g09600.1	7
3347	LOC_Os07g14540.1	7
3347	LOC_Os10g06540.1	10
3347	LOC_Os12g44250.1	12
3348	LOC_Os02g17330.1	2
3348	LOC_Os03g01600.1	3
3348	LOC_Os03g24460.1	3
3348	LOC_Os03g12890.1	3
3348	LOC_Os04g47190.1	4
3348	LOC_Os05g15530.1	5
3348	LOC_Os05g48450.1	5
3348	LOC_Os10g40200.1	10
3349	LOC_Os01g06960.1	1
3349	LOC_Os01g06990.1	1
3349	LOC_Os01g07020.1	1
3349	LOC_Os01g07030.1	1
3349	LOC_Os01g07040.1	1
3349	LOC_Os01g07050.1	1
3349	LOC_Os01g07060.1	1
3349	LOC_Os01g07010.1	1
3350	LOC_Os01g15660.1	1
3350	LOC_Os01g21320.1	1
3350	LOC_Os01g62020.1	1
3350	LOC_Os01g73790.1	1
3350	LOC_Os03g32170.1	3
3350	LOC_Os05g32140.1	5
3350	LOC_Os07g11110.1	7
3350	LOC_Os07g41050.1	7
3351	LOC_Os02g36140.1	2
3351	LOC_Os02g36220.1	2
3351	LOC_Os02g36264.1	2
3351	LOC_Os04g52240.1	4
3351	LOC_Os04g52210.1	4
3351	LOC_Os04g52230.1	4
3351	LOC_Os11g28530.1	11
3351	LOC_Os12g30824.1	12
3352	LOC_Os01g23640.1	1
3352	LOC_Os01g31470.1	1
3352	LOC_Os01g47262.1	1
3352	LOC_Os04g30780.1	4
3352	LOC_Os05g01450.1	5
3352	LOC_Os08g39070.1	8
3352	LOC_Os09g21100.1	9
3352	LOC_Os12g42810.1	12
3353	LOC_Os01g49360.1	1
3353	LOC_Os02g03200.1	2
3353	LOC_Os02g03210.1	2

3353	LOC_Os03g36530.1	3
3353	LOC_Os04g29090.1	4
3353	LOC_Os04g29210.1	4
3353	LOC_Os08g02230.1	8
3353	LOC_Os08g40720.1	8
3354	LOC_Os01g49390.1	1
3354	LOC_Os01g73760.1	1
3354	LOC_Os03g24240.1	3
3354	LOC_Os03g59570.1	3
3354	LOC_Os05g24660.1	5
3354	LOC_Os05g47840.1	5
3354	LOC_Os06g51350.1	6
3354	LOC_Os07g11050.1	7
3355	LOC_Os02g43030.1	2
3355	LOC_Os04g38940.1	4
3355	LOC_Os04g45520.1	4
3355	LOC_Os04g59020.1	4
3355	LOC_Os06g01440.1	6
3355	LOC_Os08g05720.1	8
3355	LOC_Os09g23300.1	9
3355	LOC_Os11g06310.1	11
3356	LOC_Os02g03270.1	2
3356	LOC_Os02g57820.1	2
3356	LOC_Os04g49990.1	4
3356	LOC_Os04g58730.1	4
3356	LOC_Os06g22030.1	6
3356	LOC_Os08g02490.1	8
3356	LOC_Os08g40150.1	8
3356	LOC_Os09g31470.1	9
3357	LOC_Os02g03730.1	2
3357	LOC_Os03g55310.1	3
3357	LOC_Os03g55570.1	3
3357	LOC_Os04g31320.1	4
3357	LOC_Os04g56980.1	4
3357	LOC_Os08g39310.1	8
3357	LOC_Os09g04720.1	9
3357	LOC_Os12g32280.1	12
3358	LOC_Os01g48700.1	1
3358	LOC_Os02g33430.1	2
3358	LOC_Os02g50630.1	2
3358	LOC_Os03g05760.1	3
3358	LOC_Os04g33950.1	4
3358	LOC_Os06g13670.1	6
3358	LOC_Os10g30420.1	10
3358	LOC_Os12g06200.1	12
3359	LOC_Os02g44230.1	2
3359	LOC_Os04g46760.1	4
3359	LOC_Os05g03810.1	5
3359	LOC_Os05g06160.1	5
3359	LOC_Os06g11840.1	6
3359	LOC_Os07g30160.1	7
3359	LOC_Os10g40550.1	10

3359	LOC_Os12g32130.1	12
3360	LOC_Os02g34884.1	2
3360	LOC_Os02g54150.1	2
3360	LOC_Os03g08430.1	3
3360	LOC_Os03g18080.1	3
3360	LOC_Os06g09540.1	6
3360	LOC_Os06g24840.1	6
3360	LOC_Os08g01750.1	8
3360	LOC_Os11g20384.1	11
3361	LOC_Os03g44300.1	3
3361	LOC_Os05g33840.1	5
3361	LOC_Os06g05100.1	6
3361	LOC_Os07g07470.1	7
3361	LOC_Os07g09190.1	7
3361	LOC_Os08g42410.1	8
3361	LOC_Os09g33500.1	9
3361	LOC_Os12g42230.1	12
3362	LOC_Os05g02530.1	5
3362	LOC_Os06g12290.1	6
3362	LOC_Os06g12630.1	6
3362	LOC_Os10g38150.1	10
3362	LOC_Os10g38314.1	10
3362	LOC_Os11g03210.1	11
3362	LOC_Os11g37730.1	11
3362	LOC_Os12g10730.1	12
3363	LOC_Os01g03290.1	1
3363	LOC_Os01g13370.1	1
3363	LOC_Os02g03620.1	2
3363	LOC_Os02g19140.1	2
3363	LOC_Os03g24040.1	3
3363	LOC_Os05g14860.1	5
3363	LOC_Os06g20610.1	6
3363	LOC_Os07g46560.1	7
3364	LOC_Os02g46380.1	2
3364	LOC_Os03g08880.1	3
3364	LOC_Os04g49748.1	4
3364	LOC_Os04g49739.1	4
3364	LOC_Os04g49757.1	4
3364	LOC_Os09g29210.1	9
3364	LOC_Os09g37640.1	9
3364	LOC_Os09g38510.1	9
3365	LOC_Os01g45370.1	1
3365	LOC_Os01g56310.1	1
3365	LOC_Os01g74190.1	1
3365	LOC_Os03g10520.1	3
3365	LOC_Os04g38330.1	4
3365	LOC_Os05g43576.1	5
3365	LOC_Os08g42730.1	8
3365	LOC_Os12g17070.1	12
3366	LOC_Os05g27890.1	5
3366	LOC_Os06g06660.1	6
3366	LOC_Os06g06680.1	6

3366	LOC_Os06g06580.1	6
3366	LOC_Os08g06690.1	8
3366	LOC_Os08g06700.1	8
3366	LOC_Os08g06890.1	8
3366	LOC_Os10g21490.1	10
3367	LOC_Os03g49720.1	3
3367	LOC_Os04g34460.1	4
3367	LOC_Os04g46610.1	4
3367	LOC_Os04g51792.1	4
3367	LOC_Os07g28790.1	7
3367	LOC_Os09g04790.1	9
3367	LOC_Os10g42500.1	10
3367	LOC_Os11g38260.1	11
3368	LOC_Os01g11220.1	1
3368	LOC_Os01g19870.1	1
3368	LOC_Os01g54530.1	1
3368	LOC_Os02g51160.1	2
3368	LOC_Os03g55920.1	3
3368	LOC_Os05g45490.1	5
3368	LOC_Os06g50860.1	6
3368	LOC_Os11g09170.1	11
3369	LOC_Os03g25510.1	3
3369	LOC_Os05g52120.1	5
3369	LOC_Os07g17820.1	7
3369	LOC_Os07g24578.1	7
3369	LOC_Os07g24498.1	7
3369	LOC_Os09g10190.1	9
3369	LOC_Os11g30750.1	11
3369	LOC_Os12g30740.1	12
3370	LOC_Os01g68140.1	1
3370	LOC_Os02g10530.1	2
3370	LOC_Os03g59200.1	3
3370	LOC_Os05g01790.1	5
3370	LOC_Os06g08350.1	6
3370	LOC_Os07g38460.1	7
3370	LOC_Os08g44310.1	8
3370	LOC_Os11g05280.1	11
3371	LOC_Os02g25250.1	2
3371	LOC_Os05g26340.1	5
3371	LOC_Os05g18040.1	5
3371	LOC_Os08g19940.1	8
3371	LOC_Os08g30960.1	8
3371	LOC_Os09g28990.1	9
3371	LOC_Os10g24320.1	10
3371	LOC_Os12g22560.1	12
3372	LOC_Os05g35910.1	5
3372	LOC_Os05g35930.1	5
3372	LOC_Os05g35950.1	5
3372	LOC_Os05g35820.1	5
3372	LOC_Os05g35830.1	5
3372	LOC_Os05g35840.1	5
3372	LOC_Os05g35960.1	5

3372	LOC_Os06g02440.1	6
3373	LOC_Os01g59540.1	1
3373	LOC_Os01g62720.1	1
3373	LOC_Os04g38820.1	4
3373	LOC_Os04g40350.1	4
3373	LOC_Os04g53020.1	4
3373	LOC_Os04g55580.1	4
3373	LOC_Os06g23220.1	6
3373	LOC_Os12g38510.1	12
3374	LOC_Os01g45250.1	1
3374	LOC_Os01g47270.1	1
3374	LOC_Os02g04130.1	2
3374	LOC_Os02g52380.1	2
3374	LOC_Os05g49350.1	5
3374	LOC_Os05g49370.1	5
3374	LOC_Os05g50230.1	5
3374	LOC_Os07g42740.1	7
3375	LOC_Os01g28894.1	1
3375	LOC_Os01g59210.1	1
3375	LOC_Os02g19710.1	2
3375	LOC_Os07g24130.1	7
3375	LOC_Os07g27090.1	7
3375	LOC_Os09g15210.1	9
3375	LOC_Os09g19470.1	9
3375	LOC_Os11g25650.1	11
3376	LOC_Os03g49280.1	3
3376	LOC_Os03g49300.1	3
3376	LOC_Os03g49310.1	3
3376	LOC_Os03g49270.1	3
3376	LOC_Os09g11200.1	9
3376	LOC_Os09g11210.1	9
3376	LOC_Os12g27020.1	12
3376	LOC_Os12g26960.1	12
3377	LOC_Os01g62584.1	1
3377	LOC_Os04g53150.1	4
3377	LOC_Os07g24120.1	7
3377	LOC_Os07g34250.1	7
3377	LOC_Os07g41220.1	7
3377	LOC_Os08g09820.1	8
3377	LOC_Os08g36520.1	8
3377	LOC_Os09g20730.1	9
3378	LOC_Os01g15080.1	1
3378	LOC_Os01g28670.1	1
3378	LOC_Os02g20180.1	2
3378	LOC_Os02g32720.1	2
3378	LOC_Os02g34620.1	2
3378	LOC_Os06g14790.1	6
3378	LOC_Os07g05220.1	7
3378	LOC_Os08g37220.1	8
3379	LOC_Os05g45000.1	5
3379	LOC_Os05g44950.1	5
3379	LOC_Os09g17880.1	9

3379	LOC_Os09g19180.1	9
3379	LOC_Os09g19360.1	9
3379	LOC_Os09g18000.1	9
3379	LOC_Os09g18550.1	9
3379	LOC_Os09g19500.1	9
3380	LOC_Os03g06480.1	3
3380	LOC_Os08g41660.1	8
3380	LOC_Os10g21040.1	10
3380	LOC_Os11g29740.1	11
3380	LOC_Os11g41950.1	11
3380	LOC_Os11g41960.1	11
3380	LOC_Os12g29720.1	12
3380	LOC_Os12g29719.1	12
3381	LOC_Os01g01450.1	1
3381	LOC_Os03g20920.1	3
3381	LOC_Os03g20940.1	3
3381	LOC_Os03g21040.1	3
3381	LOC_Os07g48460.1	7
3381	LOC_Os07g48500.1	7
3381	LOC_Os07g48490.1	7
3381	LOC_Os12g08340.1	12
3382	LOC_Os02g37240.1	2
3382	LOC_Os06g16040.1	6
3382	LOC_Os08g36890.1	8
3382	LOC_Os08g38190.1	8
3382	LOC_Os08g40350.1	8
3382	LOC_Os09g11900.1	9
3382	LOC_Os11g31790.1	11
3382	LOC_Os12g15980.1	12
3383	LOC_Os02g34220.1	2
3383	LOC_Os05g06000.1	5
3383	LOC_Os05g29680.1	5
3383	LOC_Os10g40450.1	10
3383	LOC_Os11g39100.1	11
3383	LOC_Os11g39060.1	11
3383	LOC_Os11g39070.1	11
3383	LOC_Os11g39120.1	11
3384	LOC_Os03g25160.1	3
3384	LOC_Os03g39540.1	3
3384	LOC_Os03g40600.1	3
3384	LOC_Os04g09430.1	4
3384	LOC_Os06g42610.1	6
3384	LOC_Os06g43440.1	6
3384	LOC_Os07g19130.1	7
3384	LOC_Os09g08920.1	9
3385	LOC_Os01g19370.1	1
3385	LOC_Os03g41200.1	3
3385	LOC_Os04g47440.1	4
3385	LOC_Os05g50420.1	5
3385	LOC_Os09g25170.1	9
3385	LOC_Os11g06800.1	11
3385	LOC_Os11g14620.1	11

3385	LOC_Os12g06420.1	12
3386	LOC_Os01g62530.1	1
3386	LOC_Os01g62550.1	1
3386	LOC_Os01g62560.1	1
3386	LOC_Os11g06330.1	11
3386	LOC_Os11g06070.1	11
3386	LOC_Os11g06090.1	11
3386	LOC_Os11g06370.1	11
3386	LOC_Os11g44000.1	11
3387	LOC_Os01g45150.1	1
3387	LOC_Os01g58410.1	1
3387	LOC_Os02g26220.1	2
3387	LOC_Os03g41310.1	3
3387	LOC_Os04g37930.1	4
3387	LOC_Os06g02280.1	6
3387	LOC_Os07g17380.1	7
3387	LOC_Os10g24500.1	10
3388	LOC_Os02g34440.1	2
3388	LOC_Os04g11320.1	4
3388	LOC_Os06g24970.1	6
3388	LOC_Os07g26230.1	7
3388	LOC_Os09g08740.1	9
3388	LOC_Os11g28890.1	11
3388	LOC_Os12g20070.1	12
3388	LOC_Os12g28650.1	12
3389	LOC_Os02g54420.1	2
3389	LOC_Os02g54450.1	2
3389	LOC_Os02g54370.1	2
3389	LOC_Os02g54390.1	2
3389	LOC_Os06g09270.1	6
3389	LOC_Os09g26310.1	9
3389	LOC_Os09g26320.1	9
3389	LOC_Os09g26330.1	9
3390	LOC_Os03g41890.1	3
3390	LOC_Os04g20730.1	4
3390	LOC_Os04g18710.1	4
3390	LOC_Os05g18428.1	5
3390	LOC_Os05g18446.1	5
3390	LOC_Os08g23520.1	8
3390	LOC_Os09g09410.1	9
3390	LOC_Os11g24260.1	11
3391	LOC_Os03g18650.1	3
3391	LOC_Os03g28360.1	3
3391	LOC_Os03g40430.1	3
3391	LOC_Os06g29230.1	6
3391	LOC_Os08g15900.1	8
3391	LOC_Os11g17520.1	11
3391	LOC_Os12g03160.1	12
3391	LOC_Os12g39950.1	12
3392	LOC_Os02g42450.1	2
3392	LOC_Os04g29550.1	4
3392	LOC_Os06g02640.1	6

3392	LOC_Os10g34760.1	10
3392	LOC_Os10g34770.1	10
3392	LOC_Os10g34840.1	10
3392	LOC_Os10g34896.1	10
3392	LOC_Os10g34902.1	10
3393	LOC_Os01g50230.1	1
3393	LOC_Os03g10290.1	3
3393	LOC_Os03g56920.1	3
3393	LOC_Os04g23200.1	4
3393	LOC_Os04g49280.1	4
3393	LOC_Os04g50840.1	4
3393	LOC_Os06g16360.1	6
3393	LOC_Os08g09890.1	8
3394	LOC_Os01g52190.1	1
3394	LOC_Os01g62630.1	1
3394	LOC_Os03g08790.1	3
3394	LOC_Os03g16500.1	3
3394	LOC_Os04g37570.1	4
3394	LOC_Os04g50850.1	4
3394	LOC_Os04g58070.1	4
3394	LOC_Os07g34870.1	7
3394	LOC_Os07g34900.1	7
3395	LOC_Os02g13310.1	2
3395	LOC_Os03g47730.1	3
3395	LOC_Os03g52239.1	3
3395	LOC_Os03g03260.1	3
3395	LOC_Os06g01934.1	6
3395	LOC_Os06g36680.1	6
3395	LOC_Os10g39030.1	10
3395	LOC_Os11g06020.1	11
3395	LOC_Os12g43950.1	12
3396	LOC_Os04g09770.1	4
3396	LOC_Os04g43020.1	4
3396	LOC_Os05g25390.1	5
3396	LOC_Os08g24630.1	8
3396	LOC_Os09g29520.1	9
3396	LOC_Os10g05250.1	10
3396	LOC_Os10g05400.1	10
3396	LOC_Os11g35274.1	11
3396	LOC_Os11g35290.1	11
3397	LOC_Os01g01150.1	1
3397	LOC_Os02g03040.1	2
3397	LOC_Os03g15890.1	3
3397	LOC_Os03g27030.1	3
3397	LOC_Os05g30140.1	5
3397	LOC_Os06g50890.1	6
3397	LOC_Os07g43050.1	7
3397	LOC_Os11g41820.1	11
3397	LOC_Os11g47830.1	11
3398	LOC_Os01g66720.1	1
3398	LOC_Os04g41960.1	4
3398	LOC_Os06g40070.1	6

3398	LOC_Os11g14910.1	11
3398	LOC_Os12g12560.1	12
3398	LOC_Os12g12580.1	12
3398	LOC_Os12g12590.1	12
3398	LOC_Os12g12470.1	12
3398	LOC_Os12g12514.1	12
3399	LOC_Os02g41630.1	2
3399	LOC_Os02g41650.1	2
3399	LOC_Os02g41670.1	2
3399	LOC_Os02g41680.1	2
3399	LOC_Os04g43760.1	4
3399	LOC_Os04g43800.1	4
3399	LOC_Os05g35290.1	5
3399	LOC_Os11g48110.1	11
3399	LOC_Os12g33610.1	12
3400	LOC_Os01g70680.1	1
3400	LOC_Os02g07600.1	2
3400	LOC_Os02g07550.1	2
3400	LOC_Os02g12060.1	2
3400	LOC_Os03g03810.1	3
3400	LOC_Os04g11130.1	4
3400	LOC_Os04g44130.1	4
3400	LOC_Os04g31250.1	4
3400	LOC_Os12g41790.1	12
3401	LOC_Os01g09460.1	1
3401	LOC_Os01g53930.1	1
3401	LOC_Os01g71320.1	1
3401	LOC_Os05g31110.1	5
3401	LOC_Os05g44760.1	5
3401	LOC_Os05g45590.1	5
3401	LOC_Os05g09500.1	5
3401	LOC_Os07g09890.1	7
3401	LOC_Os07g26540.1	7
3402	LOC_Os01g48900.1	1
3402	LOC_Os01g69130.1	1
3402	LOC_Os03g15420.1	3
3402	LOC_Os03g50520.1	3
3402	LOC_Os04g04060.1	4
3402	LOC_Os04g31190.1	4
3402	LOC_Os05g48240.1	5
3402	LOC_Os09g39960.1	9
3402	LOC_Os10g41820.1	10
3403	LOC_Os03g18850.1	3
3403	LOC_Os04g39150.1	4
3403	LOC_Os04g50700.1	4
3403	LOC_Os04g50710.1	4
3403	LOC_Os08g28670.1	8
3403	LOC_Os12g36850.1	12
3403	LOC_Os12g36830.1	12
3403	LOC_Os12g36840.1	12
3403	LOC_Os12g36880.1	12
3404	LOC_Os01g09510.1	1

3404	LOC_Os01g13080.1	1
3404	LOC_Os02g32760.1	2
3404	LOC_Os05g37330.1	5
3404	LOC_Os06g48780.1	6
3404	LOC_Os07g14750.1	7
3404	LOC_Os07g28710.1	7
3404	LOC_Os08g02340.1	8
3404	LOC_Os08g15180.1	8
3405	LOC_Os02g42660.1	2
3405	LOC_Os02g48560.1	2
3405	LOC_Os03g18070.1	3
3405	LOC_Os07g23410.1	7
3405	LOC_Os07g23430.1	7
3405	LOC_Os07g49310.1	7
3405	LOC_Os08g34220.1	8
3405	LOC_Os11g01340.1	11
3405	LOC_Os12g01370.1	12
3406	LOC_Os01g32730.1	1
3406	LOC_Os02g34540.1	2
3406	LOC_Os02g37470.1	2
3406	LOC_Os02g57470.1	2
3406	LOC_Os03g05540.1	3
3406	LOC_Os03g18360.1	3
3406	LOC_Os05g31056.1	5
3406	LOC_Os07g03070.1	7
3406	LOC_Os11g37520.1	11
3407	LOC_Os01g48420.1	1
3407	LOC_Os01g16152.1	1
3407	LOC_Os02g09940.1	2
3407	LOC_Os02g33450.1	2
3407	LOC_Os04g33970.1	4
3407	LOC_Os06g42000.1	6
3407	LOC_Os06g09610.1	6
3407	LOC_Os07g44440.1	7
3407	LOC_Os07g44430.1	7
3408	LOC_Os01g66420.1	1
3408	LOC_Os02g35600.1	2
3408	LOC_Os03g60390.1	3
3408	LOC_Os04g36730.1	4
3408	LOC_Os05g07040.1	5
3408	LOC_Os05g34640.1	5
3408	LOC_Os07g12910.1	7
3408	LOC_Os07g41740.1	7
3408	LOC_Os11g14010.1	11
3409	LOC_Os03g24200.1	3
3409	LOC_Os03g44920.1	3
3409	LOC_Os03g46140.1	3
3409	LOC_Os03g46720.1	3
3409	LOC_Os03g46510.1	3
3409	LOC_Os03g47420.1	3
3409	LOC_Os10g24010.1	10
3409	LOC_Os11g41570.1	11

3409	LOC_Os12g43770.1	12
3410	LOC_Os04g18790.1	4
3410	LOC_Os07g36830.1	7
3410	LOC_Os07g36840.1	7
3410	LOC_Os07g36870.1	7
3410	LOC_Os07g36910.1	7
3410	LOC_Os07g36920.1	7
3410	LOC_Os10g04590.1	10
3410	LOC_Os10g04600.1	10
3410	LOC_Os10g04610.1	10
3411	LOC_Os09g15570.1	9
3411	LOC_Os09g15440.1	9
3411	LOC_Os09g15540.1	9
3411	LOC_Os09g15550.1	9
3411	LOC_Os09g16810.1	9
3411	LOC_Os09g17190.1	9
3411	LOC_Os09g17530.1	9
3411	LOC_Os09g17540.1	9
3411	LOC_Os09g17152.1	9
3412	LOC_Os01g16000.1	1
3412	LOC_Os01g70210.1	1
3412	LOC_Os02g10650.1	2
3412	LOC_Os02g51610.1	2
3412	LOC_Os03g63370.1	3
3412	LOC_Os05g18470.1	5
3412	LOC_Os06g40510.1	6
3412	LOC_Os07g27310.1	7
3412	LOC_Os09g08390.1	9
3413	LOC_Os04g35370.1	4
3413	LOC_Os10g28770.1	10
3413	LOC_Os10g29100.1	10
3413	LOC_Os10g29110.1	10
3413	LOC_Os10g29150.1	10
3413	LOC_Os10g29180.1	10
3413	LOC_Os10g29310.1	10
3413	LOC_Os11g40680.1	11
3413	LOC_Os11g41260.1	11
3414	LOC_Os04g53390.1	4
3414	LOC_Os06g45720.1	6
3414	LOC_Os08g13090.1	8
3414	LOC_Os08g13060.1	8
3414	LOC_Os08g40490.1	8
3414	LOC_Os08g41220.1	8
3414	LOC_Os08g41230.1	8
3414	LOC_Os09g06890.1	9
3414	LOC_Os09g16850.1	9
3415	LOC_Os01g08400.1	1
3415	LOC_Os01g15110.1	1
3415	LOC_Os02g47440.1	2
3415	LOC_Os03g57310.1	3
3415	LOC_Os06g02570.1	6
3415	LOC_Os06g07200.1	6

3415	LOC_Os06g11930.1	6
3415	LOC_Os06g39050.1	6
3415	LOC_Os07g07000.1	7
3416	LOC_Os01g41050.1	1
3416	LOC_Os01g52130.1	1
3416	LOC_Os03g09930.1	3
3416	LOC_Os03g09970.1	3
3416	LOC_Os03g06520.1	3
3416	LOC_Os03g09980.1	3
3416	LOC_Os04g55800.1	4
3416	LOC_Os06g05160.1	6
3416	LOC_Os08g31410.1	8
3417	LOC_Os07g26740.1	7
3417	LOC_Os07g33860.1	7
3417	LOC_Os07g33898.1	7
3417	LOC_Os07g33921.1	7
3417	LOC_Os07g33943.1	7
3417	LOC_Os07g33870.1	7
3417	LOC_Os07g33880.1	7
3417	LOC_Os07g33979.1	7
3417	LOC_Os07g33997.1	7
3418	LOC_Os03g61290.1	3
3418	LOC_Os05g19500.1	5
3418	LOC_Os05g40650.1	5
3418	LOC_Os08g02450.1	8
3418	LOC_Os11g01820.1	11
3418	LOC_Os11g03070.1	11
3418	LOC_Os12g01820.1	12
3418	LOC_Os12g02840.1	12
3418	LOC_Os12g42200.1	12
3419	LOC_Os01g52214.1	1
3419	LOC_Os01g58030.1	1
3419	LOC_Os03g30610.1	3
3419	LOC_Os05g45730.1	5
3419	LOC_Os06g22010.1	6
3419	LOC_Os07g02580.1	7
3419	LOC_Os10g21260.1	10
3419	LOC_Os10g38210.1	10
3419	LOC_Os12g19420.1	12
3420	LOC_Os02g56160.1	2
3420	LOC_Os02g57690.1	2
3420	LOC_Os04g40740.1	4
3420	LOC_Os04g41020.1	4
3420	LOC_Os08g03420.1	8
3420	LOC_Os08g13360.1	8
3420	LOC_Os09g07460.1	9
3420	LOC_Os11g43590.1	11
3420	LOC_Os12g35350.1	12
3421	LOC_Os01g13550.1	1
3421	LOC_Os03g04770.1	3
3421	LOC_Os03g22790.1	3
3421	LOC_Os07g35880.1	7

3421	LOC_Os07g35940.1	7
3421	LOC_Os07g47120.1	7
3421	LOC_Os09g39570.1	9
3421	LOC_Os10g32810.1	10
3421	LOC_Os10g41550.1	10
3422	LOC_Os07g05160.1	7
3422	LOC_Os07g22680.1	7
3422	LOC_Os07g43180.1	7
3422	LOC_Os07g43250.1	7
3422	LOC_Os07g43260.1	7
3422	LOC_Os07g43270.1	7
3422	LOC_Os09g10270.1	9
3422	LOC_Os10g30200.1	10
3422	LOC_Os11g26910.1	11
3423	LOC_Os01g40410.1	1
3423	LOC_Os01g41400.1	1
3423	LOC_Os01g41420.1	1
3423	LOC_Os02g01100.1	2
3423	LOC_Os02g54730.1	2
3423	LOC_Os04g38680.1	4
3423	LOC_Os06g12320.1	6
3423	LOC_Os11g19240.1	11
3423	LOC_Os12g38570.1	12
3424	LOC_Os01g64810.1	1
3424	LOC_Os01g70100.1	1
3424	LOC_Os05g36090.1	5
3424	LOC_Os08g42370.1	8
3424	LOC_Os08g42620.1	8
3424	LOC_Os09g33450.1	9
3424	LOC_Os11g03910.1	11
3424	LOC_Os11g32960.1	11
3424	LOC_Os12g03730.1	12
3425	LOC_Os01g64890.1	1
3425	LOC_Os01g68040.1	1
3425	LOC_Os03g04480.1	3
3425	LOC_Os03g48000.1	3
3425	LOC_Os03g53110.1	3
3425	LOC_Os04g35160.1	4
3425	LOC_Os04g42280.1	4
3425	LOC_Os06g44150.1	6
3425	LOC_Os10g39790.1	10
3426	LOC_Os01g19430.1	1
3426	LOC_Os01g47100.1	1
3426	LOC_Os01g47180.1	1
3426	LOC_Os01g53580.1	1
3426	LOC_Os01g53590.1	1
3426	LOC_Os04g03990.1	4
3426	LOC_Os04g14810.1	4
3426	LOC_Os07g02150.1	7
3426	LOC_Os07g02180.1	7
3427	LOC_Os01g05050.1	1
3427	LOC_Os01g05060.1	1

3427	LOC_Os01g05010.1	1
3427	LOC_Os01g56460.1	1
3427	LOC_Os05g38210.1	5
3427	LOC_Os06g22070.1	6
3427	LOC_Os08g34130.1	8
3427	LOC_Os09g38500.1	9
3427	LOC_Os11g04440.1	11
3428	LOC_Os03g39050.1	3
3428	LOC_Os03g39100.1	3
3428	LOC_Os05g25960.1	5
3428	LOC_Os07g09740.1	7
3428	LOC_Os07g09830.1	7
3428	LOC_Os07g09860.1	7
3428	LOC_Os07g13920.1	7
3428	LOC_Os07g17180.1	7
3428	LOC_Os07g27330.1	7
3429	LOC_Os03g14550.1	3
3429	LOC_Os03g41060.1	3
3429	LOC_Os03g55290.1	3
3429	LOC_Os04g39110.1	4
3429	LOC_Os05g31280.1	5
3429	LOC_Os05g35690.1	5
3429	LOC_Os06g15620.1	6
3429	LOC_Os06g51320.1	6
3429	LOC_Os09g24840.1	9
3430	LOC_Os01g72140.1	1
3430	LOC_Os01g72150.1	1
3430	LOC_Os03g39850.1	3
3430	LOC_Os07g07320.1	7
3430	LOC_Os10g22070.1	10
3430	LOC_Os10g25590.1	10
3430	LOC_Os10g38720.1	10
3430	LOC_Os10g38730.1	10
3430	LOC_Os10g38600.1	10
3431	LOC_Os02g25700.1	2
3431	LOC_Os02g26840.1	2
3431	LOC_Os02g45344.1	2
3431	LOC_Os04g20880.1	4
3431	LOC_Os04g40580.1	4
3431	LOC_Os05g05200.1	5
3431	LOC_Os05g10810.1	5
3431	LOC_Os06g36800.1	6
3431	LOC_Os07g35570.1	7
3432	LOC_Os01g66510.1	1
3432	LOC_Os02g10350.1	2
3432	LOC_Os03g03700.1	3
3432	LOC_Os04g36680.1	4
3432	LOC_Os05g09050.1	5
3432	LOC_Os05g34550.1	5
3432	LOC_Os06g29110.1	6
3432	LOC_Os06g40790.1	6
3432	LOC_Os10g39520.1	10

3433	LOC_Os01g07310.1	1
3433	LOC_Os02g41780.1	2
3433	LOC_Os05g07670.1	5
3433	LOC_Os05g12490.1	5
3433	LOC_Os06g30950.1	6
3433	LOC_Os07g39280.1	7
3433	LOC_Os10g33920.1	10
3433	LOC_Os12g05780.1	12
3433	LOC_Os12g05830.1	12
3434	LOC_Os01g43940.1	1
3434	LOC_Os02g46850.1	2
3434	LOC_Os03g54000.1	3
3434	LOC_Os04g50820.1	4
3434	LOC_Os06g03540.1	6
3434	LOC_Os06g03560.1	6
3434	LOC_Os06g03700.1	6
3434	LOC_Os08g23130.1	8
3434	LOC_Os08g38400.1	8
3435	LOC_Os07g43080.1	7
3435	LOC_Os08g06160.1	8
3435	LOC_Os08g15790.1	8
3435	LOC_Os08g19000.1	8
3435	LOC_Os09g17510.1	9
3435	LOC_Os11g12160.1	11
3435	LOC_Os12g13620.1	12
3435	LOC_Os12g33820.1	12
3435	LOC_Os12g43860.1	12
3436	LOC_Os03g06890.1	3
3436	LOC_Os03g44800.1	3
3436	LOC_Os03g57530.1	3
3436	LOC_Os03g58490.1	3
3436	LOC_Os04g38920.1	4
3436	LOC_Os08g16790.1	8
3436	LOC_Os08g34350.1	8
3436	LOC_Os11g36740.1	11
3436	LOC_Os12g30500.1	12
3437	LOC_Os02g50140.1	2
3437	LOC_Os02g50150.1	2
3437	LOC_Os02g50174.1	2
3437	LOC_Os03g12230.1	3
3437	LOC_Os04g43170.1	4
3437	LOC_Os04g43200.1	4
3437	LOC_Os06g14324.1	6
3437	LOC_Os06g14350.1	6
3437	LOC_Os06g14370.1	6
3438	LOC_Os01g08610.1	1
3438	LOC_Os01g18920.1	1
3438	LOC_Os02g43780.1	2
3438	LOC_Os06g11830.1	6
3438	LOC_Os06g36530.1	6
3438	LOC_Os07g15340.1	7
3438	LOC_Os09g14530.1	9

3438	LOC_Os11g14270.1	11
3438	LOC_Os11g28740.1	11
3439	LOC_Os01g52960.1	1
3439	LOC_Os01g70650.1	1
3439	LOC_Os02g53840.1	2
3439	LOC_Os03g06310.1	3
3439	LOC_Os03g62210.1	3
3439	LOC_Os04g03050.1	4
3439	LOC_Os04g03796.1	4
3439	LOC_Os05g35740.1	5
3439	LOC_Os07g25690.1	7
3440	LOC_Os01g16260.1	1
3440	LOC_Os11g04020.1	11
3440	LOC_Os11g04030.1	11
3440	LOC_Os11g04060.1	11
3440	LOC_Os11g04104.1	11
3440	LOC_Os12g03830.1	12
3440	LOC_Os12g03860.1	12
3440	LOC_Os12g03870.1	12
3440	LOC_Os12g03899.1	12
3441	LOC_Os01g04100.1	1
3441	LOC_Os01g49410.1	1
3441	LOC_Os01g49440.1	1
3441	LOC_Os01g48830.1	1
3441	LOC_Os03g59250.1	3
3441	LOC_Os05g47810.1	5
3441	LOC_Os05g47820.1	5
3441	LOC_Os05g47830.1	5
3441	LOC_Os05g48280.1	5
3442	LOC_Os01g42700.1	1
3442	LOC_Os02g05710.1	2
3442	LOC_Os02g35840.1	2
3442	LOC_Os02g53110.1	2
3442	LOC_Os04g04010.1	4
3442	LOC_Os04g37530.1	4
3442	LOC_Os06g47860.1	6
3442	LOC_Os07g23790.1	7
3442	LOC_Os09g27860.1	9
3443	LOC_Os01g24960.1	1
3443	LOC_Os01g25030.1	1
3443	LOC_Os01g26812.1	1
3443	LOC_Os10g18870.1	10
3443	LOC_Os10g18760.1	10
3443	LOC_Os11g40180.1	11
3443	LOC_Os12g09720.1	12
3443	LOC_Os12g12600.1	12
3443	LOC_Os12g26380.1	12
3444	LOC_Os01g71060.1	1
3444	LOC_Os01g71070.1	1
3444	LOC_Os01g71080.1	1
3444	LOC_Os01g71140.1	1
3444	LOC_Os01g71090.1	1

3444	LOC_Os01g71160.1	1
3444	LOC_Os05g33400.1	5
3444	LOC_Os05g33410.1	5
3444	LOC_Os09g25910.1	9
3445	LOC_Os02g15640.1	2
3445	LOC_Os02g15620.1	2
3445	LOC_Os05g12260.1	5
3445	LOC_Os06g33480.1	6
3445	LOC_Os06g33490.1	6
3445	LOC_Os06g33640.1	6
3445	LOC_Os06g33690.1	6
3445	LOC_Os06g36670.1	6
3445	LOC_Os10g42280.1	10
3446	LOC_Os03g22360.1	3
3446	LOC_Os06g09970.1	6
3446	LOC_Os06g10000.1	6
3446	LOC_Os06g10020.1	6
3446	LOC_Os06g10030.1	6
3446	LOC_Os06g10040.1	6
3446	LOC_Os06g10050.1	6
3446	LOC_Os06g10070.1	6
3446	LOC_Os06g10100.1	6
3447	LOC_Os01g18250.1	1
3447	LOC_Os03g53210.1	3
3447	LOC_Os05g03420.1	5
3447	LOC_Os09g10610.1	9
3447	LOC_Os09g30060.1	9
3447	LOC_Os09g38990.1	9
3447	LOC_Os10g22190.1	10
3447	LOC_Os10g39744.1	10
3447	LOC_Os12g06930.1	12
3448	LOC_Os02g45030.1	2
3448	LOC_Os03g40910.1	3
3448	LOC_Os04g05600.1	4
3448	LOC_Os05g23760.1	5
3448	LOC_Os06g31290.1	6
3448	LOC_Os07g11530.1	7
3448	LOC_Os07g36020.1	7
3448	LOC_Os10g31560.1	10
3448	LOC_Os12g12830.1	12
3449	LOC_Os02g20510.1	2
3449	LOC_Os03g37736.1	3
3449	LOC_Os04g19250.1	4
3449	LOC_Os06g26110.1	6
3449	LOC_Os07g15300.1	7
3449	LOC_Os07g24010.1	7
3449	LOC_Os07g29020.1	7
3449	LOC_Os10g23960.1	10
3449	LOC_Os10g27160.1	10
3450	LOC_Os01g32550.1	1
3450	LOC_Os04g09330.1	4
3450	LOC_Os04g13560.1	4

3450	LOC_Os04g19170.1	4
3450	LOC_Os04g30120.1	4
3450	LOC_Os05g19580.1	5
3450	LOC_Os06g16760.1	6
3450	LOC_Os09g09900.1	9
3450	LOC_Os09g13300.1	9
3451	LOC_Os02g06560.1	2
3451	LOC_Os04g54210.1	4
3451	LOC_Os04g54300.1	4
3451	LOC_Os04g54230.1	4
3451	LOC_Os04g54240.1	4
3451	LOC_Os04g54310.1	4
3451	LOC_Os06g46970.1	6
3451	LOC_Os07g37290.1	7
3451	LOC_Os08g08090.1	8
3452	LOC_Os04g37940.1	4
3452	LOC_Os05g48120.1	5
3452	LOC_Os06g06170.1	6
3452	LOC_Os07g45194.1	7
3452	LOC_Os07g45520.1	7
3452	LOC_Os11g03630.1	11
3452	LOC_Os12g01710.1	12
3452	LOC_Os12g10810.1	12
3452	LOC_Os12g10820.1	12
3453	LOC_Os02g46560.1	2
3453	LOC_Os02g52190.1	2
3453	LOC_Os03g08930.1	3
3453	LOC_Os03g46860.1	3
3453	LOC_Os04g50090.1	4
3453	LOC_Os05g50900.1	5
3453	LOC_Os05g51820.1	5
3453	LOC_Os08g37730.1	8
3453	LOC_Os09g29360.1	9
3453	LOC_Os10g23050.1	10
3454	LOC_Os01g08810.1	1
3454	LOC_Os01g58950.1	1
3454	LOC_Os01g59000.1	1
3454	LOC_Os01g63540.1	1
3454	LOC_Os02g47470.1	2
3454	LOC_Os03g07250.1	3
3454	LOC_Os05g08850.1	5
3454	LOC_Os10g38110.1	10
3454	LOC_Os11g29290.1	11
3454	LOC_Os12g25660.1	12
3455	LOC_Os01g11300.1	1
3455	LOC_Os01g11280.1	1
3455	LOC_Os02g02230.1	2
3455	LOC_Os02g07680.1	2
3455	LOC_Os03g04530.1	3
3455	LOC_Os03g04680.1	3
3455	LOC_Os07g11890.1	7
3455	LOC_Os10g09090.1	10

3455	LOC_Os10g23180.1	10
3455	LOC_Os10g36740.1	10
3456	LOC_Os01g10890.1	1
3456	LOC_Os01g60910.1	1
3456	LOC_Os02g06570.1	2
3456	LOC_Os03g20380.1	3
3456	LOC_Os05g11790.1	5
3456	LOC_Os05g39870.1	5
3456	LOC_Os07g05620.1	7
3456	LOC_Os07g48100.1	7
3456	LOC_Os11g02240.1	11
3456	LOC_Os12g02200.1	12
3457	LOC_Os01g62130.1	1
3457	LOC_Os03g60560.1	3
3457	LOC_Os03g60570.1	3
3457	LOC_Os05g02390.1	5
3457	LOC_Os05g38620.1	5
3457	LOC_Os07g39970.1	7
3457	LOC_Os08g20580.1	8
3457	LOC_Os08g44190.1	8
3457	LOC_Os11g47620.1	11
3457	LOC_Os11g48000.1	11
3458	LOC_Os01g58040.1	1
3458	LOC_Os02g24630.1	2
3458	LOC_Os04g16770.1	4
3458	LOC_Os04g16800.1	4
3458	LOC_Os04g16730.1	4
3458	LOC_Os08g35420.1	8
3458	LOC_Os10g21200.1	10
3458	LOC_Os10g21210.1	10
3458	LOC_Os10g39880.1	10
3458	LOC_Os10g41689.1	10
3459	LOC_Os02g07450.1	2
3459	LOC_Os03g14669.1	3
3459	LOC_Os04g58680.1	4
3459	LOC_Os06g45640.1	6
3459	LOC_Os07g36130.1	7
3459	LOC_Os07g36140.1	7
3459	LOC_Os08g33100.1	8
3459	LOC_Os08g38780.1	8
3459	LOC_Os09g30310.1	9
3459	LOC_Os12g25120.1	12
3460	LOC_Os01g51754.1	1
3460	LOC_Os02g52700.1	2
3460	LOC_Os02g52710.1	2
3460	LOC_Os04g33040.1	4
3460	LOC_Os06g49970.1	6
3460	LOC_Os08g36900.1	8
3460	LOC_Os08g36910.1	8
3460	LOC_Os09g28400.1	9
3460	LOC_Os09g28420.1	9
3460	LOC_Os09g28430.1	9

3461	LOC_Os01g17260.1	1
3461	LOC_Os01g59350.1	1
3461	LOC_Os02g10140.1	2
3461	LOC_Os04g54474.1	4
3461	LOC_Os06g15480.1	6
3461	LOC_Os06g41100.1	6
3461	LOC_Os07g48820.1	7
3461	LOC_Os08g07970.1	8
3461	LOC_Os09g10840.1	9
3461	LOC_Os09g31390.1	9
3462	LOC_Os01g31270.1	1
3462	LOC_Os02g51750.1	2
3462	LOC_Os05g31760.1	5
3462	LOC_Os05g31750.1	5
3462	LOC_Os06g11800.1	6
3462	LOC_Os07g46550.1	7
3462	LOC_Os08g32970.1	8
3462	LOC_Os09g20330.1	9
3462	LOC_Os09g23160.1	9
3462	LOC_Os09g27990.1	9
3463	LOC_Os02g33030.1	2
3463	LOC_Os04g33660.1	4
3463	LOC_Os08g32750.1	8
3463	LOC_Os08g32780.1	8
3463	LOC_Os08g32840.1	8
3463	LOC_Os08g36630.1	8
3463	LOC_Os08g36680.1	8
3463	LOC_Os09g28150.1	9
3463	LOC_Os11g05520.1	11
3463	LOC_Os12g05730.1	12
3464	LOC_Os01g16960.1	1
3464	LOC_Os01g47080.1	1
3464	LOC_Os03g20880.1	3
3464	LOC_Os03g46910.1	3
3464	LOC_Os04g58110.1	4
3464	LOC_Os07g08340.1	7
3464	LOC_Os10g42100.1	10
3464	LOC_Os11g05110.1	11
3464	LOC_Os11g10980.1	11
3464	LOC_Os12g05110.1	12
3465	LOC_Os02g44470.1	2
3465	LOC_Os03g13950.1	3
3465	LOC_Os03g56790.1	3
3465	LOC_Os03g60580.1	3
3465	LOC_Os03g60590.1	3
3465	LOC_Os04g46910.1	4
3465	LOC_Os07g20170.1	7
3465	LOC_Os07g30090.1	7
3465	LOC_Os10g37670.1	10
3465	LOC_Os12g43340.1	12
3466	LOC_Os01g22550.1	1
3466	LOC_Os02g36770.1	2

3466	LOC_Os03g48610.1	3
3466	LOC_Os03g58900.1	3
3466	LOC_Os03g58920.1	3
3466	LOC_Os06g12390.1	6
3466	LOC_Os07g09670.1	7
3466	LOC_Os07g09690.1	7
3466	LOC_Os08g03670.1	8
3466	LOC_Os12g41956.1	12
3467	LOC_Os01g51540.1	1
3467	LOC_Os01g55974.1	1
3467	LOC_Os03g20570.1	3
3467	LOC_Os03g61810.1	3
3467	LOC_Os03g02600.1	3
3467	LOC_Os04g09810.1	4
3467	LOC_Os06g29430.1	6
3467	LOC_Os06g40910.1	6
3467	LOC_Os07g14150.1	7
3467	LOC_Os09g04210.1	9
3468	LOC_Os01g12830.1	1
3468	LOC_Os02g01150.1	2
3468	LOC_Os04g01600.1	4
3468	LOC_Os04g01650.1	4
3468	LOC_Os04g01660.1	4
3468	LOC_Os04g01674.1	4
3468	LOC_Os06g29180.1	6
3468	LOC_Os06g29220.1	6
3468	LOC_Os07g15970.1	7
3468	LOC_Os07g16040.1	7
3469	LOC_Os02g53790.1	2
3469	LOC_Os03g03820.1	3
3469	LOC_Os04g01530.1	4
3469	LOC_Os04g57540.1	4
3469	LOC_Os06g02000.1	6
3469	LOC_Os06g10200.1	6
3469	LOC_Os07g43170.1	7
3469	LOC_Os08g01770.1	8
3469	LOC_Os08g02540.1	8
3469	LOC_Os08g19140.1	8
3470	LOC_Os01g04730.1	1
3470	LOC_Os02g53230.1	2
3470	LOC_Os03g37330.1	3
3470	LOC_Os03g61030.1	3
3470	LOC_Os05g43060.1	5
3470	LOC_Os06g10620.1	6
3470	LOC_Os06g46930.1	6
3470	LOC_Os11g04370.1	11
3470	LOC_Os12g04160.1	12
3470	LOC_Os12g05430.1	12
3471	LOC_Os01g04920.1	1
3471	LOC_Os01g15780.1	1
3471	LOC_Os01g46430.1	1
3471	LOC_Os03g08300.1	3

3471	LOC_Os03g15840.1	3
3471	LOC_Os04g49960.1	4
3471	LOC_Os07g01030.1	7
3471	LOC_Os07g16960.1	7
3471	LOC_Os09g12530.1	9
3471	LOC_Os12g39340.1	12
3472	LOC_Os01g08830.1	1
3472	LOC_Os01g56710.1	1
3472	LOC_Os01g71430.1	1
3472	LOC_Os01g71440.1	1
3472	LOC_Os02g44990.1	2
3472	LOC_Os03g50050.1	3
3472	LOC_Os07g25800.1	7
3472	LOC_Os11g38230.1	11
3472	LOC_Os11g38500.1	11
3472	LOC_Os12g39520.1	12
3473	LOC_Os01g27230.1	1
3473	LOC_Os01g27240.1	1
3473	LOC_Os02g35310.1	2
3473	LOC_Os06g11250.1	6
3473	LOC_Os06g11280.1	6
3473	LOC_Os06g11200.1	6
3473	LOC_Os06g11210.1	6
3473	LOC_Os06g11290.1	6
3473	LOC_Os06g11240.1	6
3473	LOC_Os08g35740.1	8
3474	LOC_Os01g12880.1	1
3474	LOC_Os01g61030.1	1
3474	LOC_Os02g17350.1	2
3474	LOC_Os02g46962.1	2
3474	LOC_Os05g39760.1	5
3474	LOC_Os05g27320.1	5
3474	LOC_Os06g22650.1	6
3474	LOC_Os08g01740.1	8
3474	LOC_Os10g42730.1	10
3474	LOC_Os11g09329.1	11
3475	LOC_Os01g25110.1	1
3475	LOC_Os01g62150.1	1
3475	LOC_Os02g21250.1	2
3475	LOC_Os03g12710.1	3
3475	LOC_Os03g57040.1	3
3475	LOC_Os03g59660.1	3
3475	LOC_Os05g38610.1	5
3475	LOC_Os08g30480.1	8
3475	LOC_Os10g22330.1	10
3475	LOC_Os12g10560.1	12
3476	LOC_Os01g52030.1	1
3476	LOC_Os01g56040.1	1
3476	LOC_Os02g10200.1	2
3476	LOC_Os02g32840.1	2
3476	LOC_Os03g57900.1	3
3476	LOC_Os03g57890.1	3

3476	LOC_Os06g41010.1	6
3476	LOC_Os07g07350.1	7
3476	LOC_Os07g07400.1	7
3476	LOC_Os08g33880.1	8
3477	LOC_Os01g05650.1	1
3477	LOC_Os01g74300.1	1
3477	LOC_Os03g17870.1	3
3477	LOC_Os05g02070.1	5
3477	LOC_Os05g11320.1	5
3477	LOC_Os12g38064.1	12
3477	LOC_Os12g38010.1	12
3477	LOC_Os12g38300.1	12
3477	LOC_Os12g38270.1	12
3477	LOC_Os12g38290.1	12
3478	LOC_Os06g02360.1	6
3478	LOC_Os07g05150.1	7
3478	LOC_Os07g05180.1	7
3478	LOC_Os08g28800.1	8
3478	LOC_Os08g28820.1	8
3478	LOC_Os09g10260.1	9
3478	LOC_Os09g10300.1	9
3478	LOC_Os09g10200.1	9
3478	LOC_Os09g10230.1	9
3478	LOC_Os09g36830.1	9
3479	LOC_Os01g42620.1	1
3479	LOC_Os01g48140.1	1
3479	LOC_Os01g57630.1	1
3479	LOC_Os02g07360.1	2
3479	LOC_Os05g04160.1	5
3479	LOC_Os06g13950.1	6
3479	LOC_Os07g01130.1	7
3479	LOC_Os08g20610.1	8
3479	LOC_Os11g39360.1	11
3479	LOC_Os12g05640.1	12
3480	LOC_Os01g62070.1	1
3480	LOC_Os01g03914.1	1
3480	LOC_Os02g53490.1	2
3480	LOC_Os02g58580.1	2
3480	LOC_Os03g12530.1	3
3480	LOC_Os03g22550.1	3
3480	LOC_Os04g23180.1	4
3480	LOC_Os05g03780.1	5
3480	LOC_Os05g38670.1	5
3480	LOC_Os08g32650.1	8
3481	LOC_Os01g57360.1	1
3481	LOC_Os01g70570.1	1
3481	LOC_Os04g53370.1	4
3481	LOC_Os05g28960.1	5
3481	LOC_Os05g42270.1	5
3481	LOC_Os06g49790.1	6
3481	LOC_Os07g34730.1	7
3481	LOC_Os10g35390.1	10

3481	LOC_Os10g42720.1	10
3481	LOC_Os11g41900.1	11
3482	LOC_Os01g09260.1	1
3482	LOC_Os01g56810.1	1
3482	LOC_Os01g71310.1	1
3482	LOC_Os02g12770.1	2
3482	LOC_Os02g12780.1	2
3482	LOC_Os04g44230.1	4
3482	LOC_Os05g31040.1	5
3482	LOC_Os06g37500.1	6
3482	LOC_Os08g35860.1	8
3482	LOC_Os10g34230.1	10
3483	LOC_Os01g04260.1	1
3483	LOC_Os01g46940.1	1
3483	LOC_Os02g51150.1	2
3483	LOC_Os03g01820.1	3
3483	LOC_Os06g12250.1	6
3483	LOC_Os06g22490.1	6
3483	LOC_Os07g01150.1	7
3483	LOC_Os07g43460.1	7
3483	LOC_Os10g39810.1	10
3483	LOC_Os11g48020.1	11
3484	LOC_Os01g67310.1	1
3484	LOC_Os03g27610.1	3
3484	LOC_Os08g28880.1	8
3484	LOC_Os08g37250.1	8
3484	LOC_Os08g37180.1	8
3484	LOC_Os08g37210.1	8
3484	LOC_Os09g28770.1	9
3484	LOC_Os11g39990.1	11
3484	LOC_Os12g36530.1	12
3484	LOC_Os12g36610.1	12
3485	LOC_Os01g67490.1	1
3485	LOC_Os02g06890.1	2
3485	LOC_Os02g57410.1	2
3485	LOC_Os03g15930.1	3
3485	LOC_Os03g39230.1	3
3485	LOC_Os04g33780.1	4
3485	LOC_Os04g52850.1	4
3485	LOC_Os04g57480.1	4
3485	LOC_Os08g39560.1	8
3485	LOC_Os09g31280.1	9
3486	LOC_Os01g38580.1	1
3486	LOC_Os02g47510.1	2
3486	LOC_Os03g44380.1	3
3486	LOC_Os04g46470.1	4
3486	LOC_Os07g05940.1	7
3486	LOC_Os08g28410.1	8
3486	LOC_Os10g08980.1	10
3486	LOC_Os12g24800.1	12
3486	LOC_Os12g42280.1	12
3486	LOC_Os12g44310.1	12

3487	LOC_Os01g55799.1	1
3487	LOC_Os01g61180.1	1
3487	LOC_Os01g67820.1	1
3487	LOC_Os01g69230.1	1
3487	LOC_Os02g30230.1	2
3487	LOC_Os04g31330.1	4
3487	LOC_Os05g39610.1	5
3487	LOC_Os08g41820.1	8
3487	LOC_Os11g01050.1	11
3487	LOC_Os12g01040.1	12
3488	LOC_Os01g10010.1	1
3488	LOC_Os01g61170.1	1
3488	LOC_Os01g62890.1	1
3488	LOC_Os03g53070.1	3
3488	LOC_Os03g58410.1	3
3488	LOC_Os05g11120.1	5
3488	LOC_Os05g38160.1	5
3488	LOC_Os05g39670.1	5
3488	LOC_Os11g01610.1	11
3488	LOC_Os12g01690.1	12
3489	LOC_Os01g21630.1	1
3489	LOC_Os01g66830.1	1
3489	LOC_Os01g66840.1	1
3489	LOC_Os01g74330.1	1
3489	LOC_Os01g66850.1	1
3489	LOC_Os02g47400.1	2
3489	LOC_Os04g51340.1	4
3489	LOC_Os05g02120.1	5
3489	LOC_Os07g44070.1	7
3489	LOC_Os10g40290.1	10
3490	LOC_Os01g06450.1	1
3490	LOC_Os01g48440.1	1
3490	LOC_Os03g17850.1	3
3490	LOC_Os04g01280.1	4
3490	LOC_Os04g55670.1	4
3490	LOC_Os05g03174.1	5
3490	LOC_Os05g48600.1	5
3490	LOC_Os06g47340.1	6
3490	LOC_Os07g49370.1	7
3490	LOC_Os10g13810.1	10
3491	LOC_Os01g49350.1	1
3491	LOC_Os01g51010.1	1
3491	LOC_Os02g06430.1	2
3491	LOC_Os03g15210.1	3
3491	LOC_Os03g15220.1	3
3491	LOC_Os03g15230.1	3
3491	LOC_Os03g50210.1	3
3491	LOC_Os06g47250.1	6
3491	LOC_Os08g07830.1	8
3491	LOC_Os09g37510.1	9
3492	LOC_Os01g49200.1	1
3492	LOC_Os02g03400.1	2

3492	LOC_Os02g48830.1	2
3492	LOC_Os03g13460.1	3
3492	LOC_Os05g33890.1	5
3492	LOC_Os05g47970.1	5
3492	LOC_Os06g20370.1	6
3492	LOC_Os06g40840.1	6
3492	LOC_Os08g41890.1	8
3492	LOC_Os09g27700.1	9
3493	LOC_Os01g68500.1	1
3493	LOC_Os03g48710.1	3
3493	LOC_Os03g48626.1	3
3493	LOC_Os05g29900.1	5
3493	LOC_Os05g29920.1	5
3493	LOC_Os07g36410.1	7
3493	LOC_Os11g13720.1	11
3493	LOC_Os11g13750.1	11
3493	LOC_Os11g13770.1	11
3493	LOC_Os12g09620.1	12
3494	LOC_Os01g33370.1	1
3494	LOC_Os01g33350.1	1
3494	LOC_Os02g07650.1	2
3494	LOC_Os02g09070.1	2
3494	LOC_Os02g46610.1	2
3494	LOC_Os04g50120.1	4
3494	LOC_Os06g45540.1	6
3494	LOC_Os06g41930.1	6
3494	LOC_Os08g44620.1	8
3494	LOC_Os10g42410.1	10
3495	LOC_Os01g04590.1	1
3495	LOC_Os01g20120.1	1
3495	LOC_Os01g33000.1	1
3495	LOC_Os02g58100.1	2
3495	LOC_Os03g61580.1	3
3495	LOC_Os04g44580.1	4
3495	LOC_Os04g57870.1	4
3495	LOC_Os06g51330.1	6
3495	LOC_Os07g14310.1	7
3495	LOC_Os10g35150.1	10
3496	LOC_Os01g65310.1	1
3496	LOC_Os01g65986.1	1
3496	LOC_Os02g31874.1	2
3496	LOC_Os02g29510.1	2
3496	LOC_Os04g30450.1	4
3496	LOC_Os05g35570.1	5
3496	LOC_Os05g35060.1	5
3496	LOC_Os05g43790.1	5
3496	LOC_Os06g50180.1	6
3496	LOC_Os11g09140.1	11
3497	LOC_Os02g43840.1	2
3497	LOC_Os02g56900.1	2
3497	LOC_Os03g01130.1	3
3497	LOC_Os03g10200.1	3

3497	LOC_Os04g46290.1	4
3497	LOC_Os06g01780.1	6
3497	LOC_Os06g08360.1	6
3497	LOC_Os06g36490.1	6
3497	LOC_Os10g33350.1	10
3497	LOC_Os10g38970.1	10
3498	LOC_Os01g34760.1	1
3498	LOC_Os01g51830.1	1
3498	LOC_Os02g05170.1	2
3498	LOC_Os03g36580.1	3
3498	LOC_Os04g22620.1	4
3498	LOC_Os06g34150.1	6
3498	LOC_Os07g22560.1	7
3498	LOC_Os08g16230.1	8
3498	LOC_Os12g14390.1	12
3498	LOC_Os12g15020.1	12
3499	LOC_Os01g28010.1	1
3499	LOC_Os01g29730.1	1
3499	LOC_Os04g27160.1	4
3499	LOC_Os05g18190.1	5
3499	LOC_Os06g25780.1	6
3499	LOC_Os06g26140.1	6
3499	LOC_Os08g29270.1	8
3499	LOC_Os10g15240.1	10
3499	LOC_Os11g03020.1	11
3499	LOC_Os12g33710.1	12
3500	LOC_Os02g44160.1	2
3500	LOC_Os04g01124.1	4
3500	LOC_Os04g48670.1	4
3500	LOC_Os07g09210.1	7
3500	LOC_Os07g33510.1	7
3500	LOC_Os09g10510.1	9
3500	LOC_Os10g28900.1	10
3500	LOC_Os11g18590.1	11
3500	LOC_Os11g27040.1	11
3500	LOC_Os12g26800.1	12
3501	LOC_Os01g48320.1	1
3501	LOC_Os01g52090.1	1
3501	LOC_Os02g35690.1	2
3501	LOC_Os03g18330.1	3
3501	LOC_Os03g18340.1	3
3501	LOC_Os04g36790.1	4
3501	LOC_Os04g45940.1	4
3501	LOC_Os08g37810.1	8
3501	LOC_Os09g38570.1	9
3501	LOC_Os10g41460.1	10
3502	LOC_Os01g28650.1	1
3502	LOC_Os01g31140.1	1
3502	LOC_Os02g04400.1	2
3502	LOC_Os02g24390.1	2
3502	LOC_Os02g28760.1	2
3502	LOC_Os03g44974.1	3

3502	LOC_Os05g04270.1	5
3502	LOC_Os05g19300.1	5
3502	LOC_Os07g37500.1	7
3502	LOC_Os12g24900.1	12
3503	LOC_Os01g03860.1	1
3503	LOC_Os01g38040.1	1
3503	LOC_Os01g44270.1	1
3503	LOC_Os03g17380.1	3
3503	LOC_Os03g64160.1	3
3503	LOC_Os05g45360.1	5
3503	LOC_Os06g13970.1	6
3503	LOC_Os07g03080.1	7
3503	LOC_Os09g28250.1	9
3503	LOC_Os11g03170.1	11
3504	LOC_Os02g55510.1	2
3504	LOC_Os02g55500.1	2
3504	LOC_Os02g55649.1	2
3504	LOC_Os02g55698.1	2
3504	LOC_Os02g55658.1	2
3504	LOC_Os02g55738.1	2
3504	LOC_Os02g55747.1	2
3504	LOC_Os02g55756.1	2
3504	LOC_Os02g55810.1	2
3504	LOC_Os08g02880.1	8
3505	LOC_Os04g29300.1	4
3505	LOC_Os04g28480.1	4
3505	LOC_Os04g51490.1	4
3505	LOC_Os08g22970.1	8
3505	LOC_Os09g01390.1	9
3505	LOC_Os10g01780.1	10
3505	LOC_Os11g28650.1	11
3505	LOC_Os12g16620.1	12
3505	LOC_Os12g27370.1	12
3505	LOC_Os12g35410.1	12
3506	LOC_Os01g19040.1	1
3506	LOC_Os01g63040.1	1
3506	LOC_Os02g40940.1	2
3506	LOC_Os02g47530.1	2
3506	LOC_Os04g28590.1	4
3506	LOC_Os04g54890.1	4
3506	LOC_Os05g37550.1	5
3506	LOC_Os07g32090.1	7
3506	LOC_Os07g32100.1	7
3506	LOC_Os11g20239.1	11
3507	LOC_Os03g49530.1	3
3507	LOC_Os03g62350.1	3
3507	LOC_Os04g29590.1	4
3507	LOC_Os05g35750.1	5
3507	LOC_Os05g30170.1	5
3507	LOC_Os05g46010.1	5
3507	LOC_Os06g38360.1	6
3507	LOC_Os07g33530.1	7

3507	LOC_Os08g29410.1	8
3507	LOC_Os12g31930.1	12
3508	LOC_Os01g38720.1	1
3508	LOC_Os01g38760.1	1
3508	LOC_Os01g38780.1	1
3508	LOC_Os01g38690.1	1
3508	LOC_Os01g38700.1	1
3508	LOC_Os01g38710.1	1
3508	LOC_Os07g40330.1	7
3508	LOC_Os07g43830.1	7
3508	LOC_Os07g43840.1	7
3508	LOC_Os07g43850.1	7
3509	LOC_Os04g19440.1	4
3509	LOC_Os06g22840.1	6
3509	LOC_Os06g25190.1	6
3509	LOC_Os06g39770.1	6
3509	LOC_Os07g12290.1	7
3509	LOC_Os08g19070.1	8
3509	LOC_Os08g28070.1	8
3509	LOC_Os10g21820.1	10
3509	LOC_Os10g39934.1	10
3509	LOC_Os11g22820.1	11
3510	LOC_Os04g09420.1	4
3510	LOC_Os04g18100.1	4
3510	LOC_Os05g41430.1	5
3510	LOC_Os07g07850.1	7
3510	LOC_Os07g09360.1	7
3510	LOC_Os07g34530.1	7
3510	LOC_Os11g14700.1	11
3510	LOC_Os11g16380.1	11
3510	LOC_Os11g32800.1	11
3510	LOC_Os12g09650.1	12
3511	LOC_Os04g16640.1	4
3511	LOC_Os06g28430.1	6
3511	LOC_Os06g35340.1	6
3511	LOC_Os06g35310.1	6
3511	LOC_Os09g08290.1	9
3511	LOC_Os09g12460.1	9
3511	LOC_Os11g23110.1	11
3511	LOC_Os11g26430.1	11
3511	LOC_Os11g27360.1	11
3511	LOC_Os12g17650.1	12
3512	LOC_Os01g01600.1	1
3512	LOC_Os01g13000.1	1
3512	LOC_Os02g17680.1	2
3512	LOC_Os02g45010.1	2
3512	LOC_Os04g41570.1	4
3512	LOC_Os04g47810.1	4
3512	LOC_Os04g53990.1	4
3512	LOC_Os05g14010.1	5
3512	LOC_Os05g42180.1	5
3512	LOC_Os07g28890.1	7

3513	LOC_Os01g33180.1	1
3513	LOC_Os01g48650.1	1
3513	LOC_Os02g14740.1	2
3513	LOC_Os02g14650.1	2
3513	LOC_Os04g21620.1	4
3513	LOC_Os05g34250.1	5
3513	LOC_Os07g28980.1	7
3513	LOC_Os08g06000.1	8
3513	LOC_Os10g05470.1	10
3513	LOC_Os11g29820.1	11
3514	LOC_Os01g56870.1	1
3514	LOC_Os03g01940.1	3
3514	LOC_Os03g62910.1	3
3514	LOC_Os06g04970.1	6
3514	LOC_Os07g47000.1	7
3514	LOC_Os07g47030.1	7
3514	LOC_Os10g33490.1	10
3514	LOC_Os10g33500.1	10
3514	LOC_Os11g05000.1	11
3514	LOC_Os12g04750.1	12
3515	LOC_Os01g16440.1	1
3515	LOC_Os02g19900.1	2
3515	LOC_Os03g40760.1	3
3515	LOC_Os04g22040.1	4
3515	LOC_Os05g18700.1	5
3515	LOC_Os08g14300.1	8
3515	LOC_Os08g23030.1	8
3515	LOC_Os08g23849.1	8
3515	LOC_Os08g33730.1	8
3515	LOC_Os12g30380.1	12
3516	LOC_Os01g51500.1	1
3516	LOC_Os01g56500.1	1
3516	LOC_Os02g40590.1	2
3516	LOC_Os02g40600.1	2
3516	LOC_Os02g40620.1	2
3516	LOC_Os02g40630.1	2
3516	LOC_Os02g40640.1	2
3516	LOC_Os02g40650.1	2
3516	LOC_Os04g42970.1	4
3516	LOC_Os10g34550.1	10
3517	LOC_Os02g15980.1	2
3517	LOC_Os02g30280.1	2
3517	LOC_Os02g39050.1	2
3517	LOC_Os04g31400.1	4
3517	LOC_Os04g53350.1	4
3517	LOC_Os05g34800.1	5
3517	LOC_Os05g41910.1	5
3517	LOC_Os06g31190.1	6
3517	LOC_Os07g01230.1	7
3517	LOC_Os07g12920.1	7
3518	LOC_Os01g50100.1	1
3518	LOC_Os01g52550.1	1

3518	LOC_Os01g74470.1	1
3518	LOC_Os03g08380.1	3
3518	LOC_Os03g17180.1	3
3518	LOC_Os03g54790.1	3
3518	LOC_Os04g40570.1	4
3518	LOC_Os04g54930.1	4
3518	LOC_Os05g04610.1	5
3518	LOC_Os08g05690.1	8
3518	LOC_Os08g05710.1	8
3519	LOC_Os01g49190.1	1
3519	LOC_Os01g51380.1	1
3519	LOC_Os02g07870.1	2
3519	LOC_Os04g16740.1	4
3519	LOC_Os05g47980.1	5
3519	LOC_Os06g37180.1	6
3519	LOC_Os06g45120.1	6
3519	LOC_Os09g08910.1	9
3519	LOC_Os10g21240.1	10
3519	LOC_Os10g21270.1	10
3519	LOC_Os10g38270.1	10
3520	LOC_Os01g02720.1	1
3520	LOC_Os02g25870.1	2
3520	LOC_Os02g38210.1	2
3520	LOC_Os03g08050.1	3
3520	LOC_Os03g08060.1	3
3520	LOC_Os03g63410.1	3
3520	LOC_Os03g08020.1	3
3520	LOC_Os03g08010.1	3
3520	LOC_Os04g20220.1	4
3520	LOC_Os04g50870.1	4
3520	LOC_Os04g58140.1	4
3521	LOC_Os01g46070.1	1
3521	LOC_Os01g61380.1	1
3521	LOC_Os02g01510.1	2
3521	LOC_Os03g56280.1	3
3521	LOC_Os04g46560.1	4
3521	LOC_Os05g49880.1	5
3521	LOC_Os07g43700.1	7
3521	LOC_Os08g33720.1	8
3521	LOC_Os08g44810.1	8
3521	LOC_Os10g33800.1	10
3521	LOC_Os12g43630.1	12
3522	LOC_Os02g17760.1	2
3522	LOC_Os03g30420.1	3
3522	LOC_Os04g18380.1	4
3522	LOC_Os07g41240.1	7
3522	LOC_Os08g01510.1	8
3522	LOC_Os08g01520.1	8
3522	LOC_Os08g43390.1	8
3522	LOC_Os09g08990.1	9
3522	LOC_Os09g35940.1	9
3522	LOC_Os10g26340.1	10

3522	LOC_Os12g39240.1	12
3523	LOC_Os01g14926.1	1
3523	LOC_Os02g48080.1	2
3523	LOC_Os02g06930.1	2
3523	LOC_Os03g15770.1	3
3523	LOC_Os04g35080.1	4
3523	LOC_Os04g56360.1	4
3523	LOC_Os04g58250.1	4
3523	LOC_Os06g35850.1	6
3523	LOC_Os06g46330.1	6
3523	LOC_Os08g04420.1	8
3523	LOC_Os09g03620.1	9
3524	LOC_Os01g43910.1	1
3524	LOC_Os01g45620.1	1
3524	LOC_Os01g47530.1	1
3524	LOC_Os02g04230.1	2
3524	LOC_Os05g05160.1	5
3524	LOC_Os05g49140.1	5
3524	LOC_Os05g50120.1	5
3524	LOC_Os05g50560.1	5
3524	LOC_Os06g26340.1	6
3524	LOC_Os06g49430.1	6
3524	LOC_Os11g17080.1	11
3525	LOC_Os01g12710.1	1
3525	LOC_Os02g02470.1	2
3525	LOC_Os02g47350.1	2
3525	LOC_Os04g38420.1	4
3525	LOC_Os06g19500.1	6
3525	LOC_Os06g19540.1	6
3525	LOC_Os08g37130.1	8
3525	LOC_Os09g17750.1	9
3525	LOC_Os11g16410.1	11
3525	LOC_Os12g41590.1	12
3525	LOC_Os12g43740.1	12
3526	LOC_Os01g52530.1	1
3526	LOC_Os02g03540.1	2
3526	LOC_Os02g48390.1	2
3526	LOC_Os02g40010.1	2
3526	LOC_Os04g42520.1	4
3526	LOC_Os05g08950.1	5
3526	LOC_Os05g38170.1	5
3526	LOC_Os06g41360.1	6
3526	LOC_Os07g30150.1	7
3526	LOC_Os12g39860.1	12
3526	LOC_Os12g40130.1	12
3527	LOC_Os01g07760.1	1
3527	LOC_Os03g02740.1	3
3527	LOC_Os03g27370.1	3
3527	LOC_Os03g62410.1	3
3527	LOC_Os05g07880.1	5
3527	LOC_Os06g40170.1	6
3527	LOC_Os06g40180.1	6

3527	LOC_Os06g40190.1	6
3527	LOC_Os07g15680.1	7
3527	LOC_Os09g25390.1	9
3527	LOC_Os09g37100.1	9
3528	LOC_Os01g74480.1	1
3528	LOC_Os02g25860.1	2
3528	LOC_Os03g10110.1	3
3528	LOC_Os03g21790.1	3
3528	LOC_Os03g46100.1	3
3528	LOC_Os03g57960.1	3
3528	LOC_Os03g58990.1	3
3528	LOC_Os05g02520.1	5
3528	LOC_Os08g13440.1	8
3528	LOC_Os09g37958.1	9
3528	LOC_Os09g37967.1	9
3529	LOC_Os02g56040.1	2
3529	LOC_Os02g43930.1	2
3529	LOC_Os03g44620.1	3
3529	LOC_Os03g57340.1	3
3529	LOC_Os04g46390.1	4
3529	LOC_Os05g26914.1	5
3529	LOC_Os05g26926.1	5
3529	LOC_Os05g26902.1	5
3529	LOC_Os06g02620.1	6
3529	LOC_Os12g07060.1	12
3529	LOC_Os12g42440.1	12
3530	LOC_Os01g73580.1	1
3530	LOC_Os02g01590.1	2
3530	LOC_Os02g33110.1	2
3530	LOC_Os03g52560.1	3
3530	LOC_Os04g33740.1	4
3530	LOC_Os04g33720.1	4
3530	LOC_Os04g45290.1	4
3530	LOC_Os04g56920.1	4
3530	LOC_Os04g56930.1	4
3530	LOC_Os09g08072.1	9
3530	LOC_Os09g08120.1	9
3531	LOC_Os01g58790.1	1
3531	LOC_Os02g04840.1	2
3531	LOC_Os02g58510.1	2
3531	LOC_Os02g01920.1	2
3531	LOC_Os03g02410.1	3
3531	LOC_Os03g14830.1	3
3531	LOC_Os03g61710.1	3
3531	LOC_Os04g51880.1	4
3531	LOC_Os06g48940.1	6
3531	LOC_Os10g18220.1	10
3531	LOC_Os11g11060.1	11
3532	LOC_Os01g47350.1	1
3532	LOC_Os01g54860.1	1
3532	LOC_Os01g70090.1	1
3532	LOC_Os02g43710.1	2

3532	LOC_Os02g43720.1	2
3532	LOC_Os03g19680.1	3
3532	LOC_Os05g45300.1	5
3532	LOC_Os06g39344.1	6
3532	LOC_Os10g40540.1	10
3532	LOC_Os10g42220.1	10
3532	LOC_Os12g16350.1	12
3533	LOC_Os01g46040.1	1
3533	LOC_Os02g38980.1	2
3533	LOC_Os03g03870.1	3
3533	LOC_Os03g21450.1	3
3533	LOC_Os06g04640.1	6
3533	LOC_Os06g24870.1	6
3533	LOC_Os07g32420.1	7
3533	LOC_Os07g32760.1	7
3533	LOC_Os07g37800.1	7
3533	LOC_Os08g01794.1	8
3533	LOC_Os09g37760.1	9
3534	LOC_Os03g03560.1	3
3534	LOC_Os03g51870.1	3
3534	LOC_Os03g58640.1	3
3534	LOC_Os05g46860.1	5
3534	LOC_Os08g23680.1	8
3534	LOC_Os08g41710.1	8
3534	LOC_Os10g34580.1	10
3534	LOC_Os11g03390.1	11
3534	LOC_Os11g07050.1	11
3534	LOC_Os11g27530.1	11
3534	LOC_Os12g03070.1	12
3535	LOC_Os02g52420.1	2
3535	LOC_Os04g25550.1	4
3535	LOC_Os04g52100.1	4
3535	LOC_Os05g28280.1	5
3535	LOC_Os07g25410.1	7
3535	LOC_Os07g32590.1	7
3535	LOC_Os08g31240.1	8
3535	LOC_Os08g42910.1	8
3535	LOC_Os10g36470.1	10
3535	LOC_Os12g26030.1	12
3535	LOC_Os12g43370.1	12
3536	LOC_Os01g67120.1	1
3536	LOC_Os02g01220.1	2
3536	LOC_Os02g06290.1	2
3536	LOC_Os02g56050.1	2
3536	LOC_Os03g64350.1	3
3536	LOC_Os04g17660.1	4
3536	LOC_Os05g25780.1	5
3536	LOC_Os06g50930.1	6
3536	LOC_Os09g36040.1	9
3536	LOC_Os12g24020.1	12
3536	LOC_Os12g41500.1	12
3537	LOC_Os01g09790.1	1

3537	LOC_Os01g51230.1	1
3537	LOC_Os01g61720.1	1
3537	LOC_Os01g67090.1	1
3537	LOC_Os03g44610.1	3
3537	LOC_Os03g57330.1	3
3537	LOC_Os05g38790.1	5
3537	LOC_Os05g46350.1	5
3537	LOC_Os06g06160.1	6
3537	LOC_Os08g02250.1	8
3537	LOC_Os12g42430.1	12
3538	LOC_Os02g17180.1	2
3538	LOC_Os02g38499.1	2
3538	LOC_Os02g38589.1	2
3538	LOC_Os02g38720.1	2
3538	LOC_Os04g56250.1	4
3538	LOC_Os09g17080.1	9
3538	LOC_Os09g21620.1	9
3538	LOC_Os09g21700.1	9
3538	LOC_Os09g21800.1	9
3538	LOC_Os09g21580.1	9
3538	LOC_Os09g22460.1	9
3539	LOC_Os01g55500.1	1
3539	LOC_Os01g63870.1	1
3539	LOC_Os02g50820.1	2
3539	LOC_Os03g48810.1	3
3539	LOC_Os03g60880.1	3
3539	LOC_Os07g30810.1	7
3539	LOC_Os08g28170.1	8
3539	LOC_Os08g32500.1	8
3539	LOC_Os09g15170.1	9
3539	LOC_Os09g21340.1	9
3539	LOC_Os12g39420.1	12
3540	LOC_Os01g61550.1	1
3540	LOC_Os01g61510.1	1
3540	LOC_Os01g65000.1	1
3540	LOC_Os02g34580.1	2
3540	LOC_Os02g40710.1	2
3540	LOC_Os02g40730.1	2
3540	LOC_Os03g62200.1	3
3540	LOC_Os04g43070.1	4
3540	LOC_Os05g39240.1	5
3540	LOC_Os11g01410.1	11
3540	LOC_Os12g01420.1	12
3541	LOC_Os03g03590.1	3
3541	LOC_Os05g05590.1	5
3541	LOC_Os05g31730.1	5
3541	LOC_Os06g21360.1	6
3541	LOC_Os06g36590.1	6
3541	LOC_Os07g47100.1	7
3541	LOC_Os08g43690.1	8
3541	LOC_Os09g11450.1	9
3541	LOC_Os09g30446.1	9

3541	LOC_Os09g37300.1	9
3541	LOC_Os11g42790.1	11
3542	LOC_Os03g21120.1	3
3542	LOC_Os03g26080.1	3
3542	LOC_Os07g48430.1	7
3542	LOC_Os08g33850.1	8
3542	LOC_Os10g21000.1	10
3542	LOC_Os11g03290.1	11
3542	LOC_Os11g03230.1	11
3542	LOC_Os11g03270.1	11
3542	LOC_Os11g25260.1	11
3542	LOC_Os11g25330.1	11
3542	LOC_Os12g02980.1	12
3543	LOC_Os01g52660.1	1
3543	LOC_Os01g52650.1	1
3543	LOC_Os04g32680.1	4
3543	LOC_Os05g45480.1	5
3543	LOC_Os05g45460.1	5
3543	LOC_Os06g36240.1	6
3543	LOC_Os07g40100.1	7
3543	LOC_Os09g32988.1	9
3543	LOC_Os09g39950.1	9
3543	LOC_Os10g22590.1	10
3543	LOC_Os10g39890.1	10
3544	LOC_Os05g33100.1	5
3544	LOC_Os05g33110.1	5
3544	LOC_Os05g33190.1	5
3544	LOC_Os05g33200.1	5
3544	LOC_Os08g14000.1	8
3544	LOC_Os08g14020.1	8
3544	LOC_Os08g14090.1	8
3544	LOC_Os08g14109.1	8
3544	LOC_Os11g17480.1	11
3544	LOC_Os11g17540.1	11
3544	LOC_Os11g17504.1	11
3545	LOC_Os02g53620.1	2
3545	LOC_Os03g07880.1	3
3545	LOC_Os03g29760.1	3
3545	LOC_Os03g44540.1	3
3545	LOC_Os03g48970.1	3
3545	LOC_Os07g06470.1	7
3545	LOC_Os07g41720.1	7
3545	LOC_Os08g09690.1	8
3545	LOC_Os10g25850.1	10
3545	LOC_Os12g42400.1	12
3545	LOC_Os12g41880.1	12
3546	LOC_Os01g03160.1	1
3546	LOC_Os01g10440.1	1
3546	LOC_Os01g56570.1	1
3546	LOC_Os03g16890.1	3
3546	LOC_Os03g48560.1	3
3546	LOC_Os04g23580.1	4

3546	LOC_Os05g06050.1	5
3546	LOC_Os06g40060.1	6
3546	LOC_Os08g04790.1	8
3546	LOC_Os10g30080.1	10
3546	LOC_Os12g44240.1	12
3547	LOC_Os01g26340.1	1
3547	LOC_Os07g42520.1	7
3547	LOC_Os08g26180.1	8
3547	LOC_Os08g26110.1	8
3547	LOC_Os08g26560.1	8
3547	LOC_Os08g28790.1	8
3547	LOC_Os10g18820.1	10
3547	LOC_Os11g07740.1	11
3547	LOC_Os11g07770.1	11
3547	LOC_Os11g07830.1	11
3547	LOC_Os12g07580.1	12
3548	LOC_Os01g43870.1	1
3548	LOC_Os01g47540.1	1
3548	LOC_Os01g55700.1	1
3548	LOC_Os01g61640.1	1
3548	LOC_Os03g63300.1	3
3548	LOC_Os05g11570.1	5
3548	LOC_Os05g39070.1	5
3548	LOC_Os05g43770.1	5
3548	LOC_Os05g49120.1	5
3548	LOC_Os07g01850.1	7
3548	LOC_Os07g10690.1	7
3549	LOC_Os01g08570.1	1
3549	LOC_Os01g31770.1	1
3549	LOC_Os03g60190.1	3
3549	LOC_Os04g51360.1	4
3549	LOC_Os05g33310.1	5
3549	LOC_Os06g04660.1	6
3549	LOC_Os06g17830.1	6
3549	LOC_Os09g18470.1	9
3549	LOC_Os10g40960.1	10
3549	LOC_Os11g29690.1	11
3549	LOC_Os11g43610.1	11
3550	LOC_Os01g48410.1	1
3550	LOC_Os01g55520.1	1
3550	LOC_Os01g62990.1	1
3550	LOC_Os02g17240.1	2
3550	LOC_Os02g47420.1	2
3550	LOC_Os04g47170.1	4
3550	LOC_Os05g38000.1	5
3550	LOC_Os05g48640.1	5
3550	LOC_Os07g29780.1	7
3550	LOC_Os09g37270.1	9
3550	LOC_Os10g40270.1	10
3551	LOC_Os02g51970.1	2
3551	LOC_Os02g52000.1	2
3551	LOC_Os02g52010.1	2

3551	LOC_Os02g52040.1	2
3551	LOC_Os06g04250.1	6
3551	LOC_Os06g11650.1	6
3551	LOC_Os06g11660.1	6
3551	LOC_Os06g11680.1	6
3551	LOC_Os06g11700.1	6
3551	LOC_Os07g31430.1	7
3551	LOC_Os08g37840.1	8
3552	LOC_Os01g73230.1	1
3552	LOC_Os02g02720.1	2
3552	LOC_Os02g24080.1	2
3552	LOC_Os02g32740.1	2
3552	LOC_Os02g57510.1	2
3552	LOC_Os03g11380.1	3
3552	LOC_Os05g48020.1	5
3552	LOC_Os08g17600.1	8
3552	LOC_Os08g44930.1	8
3552	LOC_Os09g19550.1	9
3552	LOC_Os11g06740.1	11
3553	LOC_Os03g25700.1	3
3553	LOC_Os03g33870.1	3
3553	LOC_Os04g17610.1	4
3553	LOC_Os04g17540.1	4
3553	LOC_Os04g18520.1	4
3553	LOC_Os04g59090.1	4
3553	LOC_Os05g22480.1	5
3553	LOC_Os06g26030.1	6
3553	LOC_Os08g10970.1	8
3553	LOC_Os08g19860.1	8
3553	LOC_Os12g33720.1	12
3554	LOC_Os01g09030.1	1
3554	LOC_Os01g40450.1	1
3554	LOC_Os03g45250.1	3
3554	LOC_Os03g45210.1	3
3554	LOC_Os04g06790.1	4
3554	LOC_Os05g51470.1	5
3554	LOC_Os08g03870.1	8
3554	LOC_Os08g37470.1	8
3554	LOC_Os09g14700.1	9
3554	LOC_Os09g29480.1	9
3554	LOC_Os12g42860.1	12
3555	LOC_Os01g29550.1	1
3555	LOC_Os01g45130.1	1
3555	LOC_Os02g55850.1	2
3555	LOC_Os03g31500.1	3
3555	LOC_Os05g27640.1	5
3555	LOC_Os06g44670.1	6
3555	LOC_Os07g22870.1	7
3555	LOC_Os08g05330.1	8
3555	LOC_Os08g11550.1	8
3555	LOC_Os08g23754.1	8
3555	LOC_Os12g31690.1	12

3556	LOC_Os01g06030.1	1
3556	LOC_Os01g06040.1	1
3556	LOC_Os01g06140.1	1
3556	LOC_Os01g06150.1	1
3556	LOC_Os01g06070.1	1
3556	LOC_Os01g06090.1	1
3556	LOC_Os01g06120.1	1
3556	LOC_Os01g06130.1	1
3556	LOC_Os04g50800.1	4
3556	LOC_Os05g02480.1	5
3556	LOC_Os09g37260.1	9
3557	LOC_Os01g14470.1	1
3557	LOC_Os02g29650.1	2
3557	LOC_Os03g32830.1	3
3557	LOC_Os04g17330.1	4
3557	LOC_Os07g15720.1	7
3557	LOC_Os07g22150.1	7
3557	LOC_Os07g26190.1	7
3557	LOC_Os08g29450.1	8
3557	LOC_Os11g20130.1	11
3557	LOC_Os12g24850.1	12
3557	LOC_Os12g34750.1	12
3558	LOC_Os01g58440.1	1
3558	LOC_Os04g31470.1	4
3558	LOC_Os04g33430.1	4
3558	LOC_Os07g08490.1	7
3558	LOC_Os07g35820.1	7
3558	LOC_Os07g41420.1	7
3558	LOC_Os08g14590.1	8
3558	LOC_Os08g21540.1	8
3558	LOC_Os08g30430.1	8
3558	LOC_Os09g10180.1	9
3558	LOC_Os11g28420.1	11
3559	LOC_Os02g32420.1	2
3559	LOC_Os03g03490.1	3
3559	LOC_Os03g15730.1	3
3559	LOC_Os03g46570.1	3
3559	LOC_Os04g28860.1	4
3559	LOC_Os04g33030.1	4
3559	LOC_Os04g32980.1	4
3559	LOC_Os08g02290.1	8
3559	LOC_Os10g41300.1	10
3559	LOC_Os10g41580.1	10
3559	LOC_Os11g34020.1	11
3560	LOC_Os06g17570.1	6
3560	LOC_Os06g17700.1	6
3560	LOC_Os06g18960.1	6
3560	LOC_Os06g19050.1	6
3560	LOC_Os06g18990.1	6
3560	LOC_Os06g19360.1	6
3560	LOC_Os11g04830.1	11
3560	LOC_Os11g11350.1	11

3560	LOC_Os11g11360.1	11
3560	LOC_Os11g45580.1	11
3560	LOC_Os12g04690.1	12
3561	LOC_Os01g25080.1	1
3561	LOC_Os01g37370.1	1
3561	LOC_Os02g39510.1	2
3561	LOC_Os03g35360.1	3
3561	LOC_Os04g34699.1	4
3561	LOC_Os06g24280.1	6
3561	LOC_Os08g27100.1	8
3561	LOC_Os09g26710.1	9
3561	LOC_Os10g20420.1	10
3561	LOC_Os11g26400.1	11
3561	LOC_Os12g25950.1	12
3562	LOC_Os01g56520.1	1
3562	LOC_Os01g63820.1	1
3562	LOC_Os02g42600.1	2
3562	LOC_Os04g44710.1	4
3562	LOC_Os05g05790.1	5
3562	LOC_Os05g24160.1	5
3562	LOC_Os08g29530.1	8
3562	LOC_Os09g33460.1	9
3562	LOC_Os09g24954.1	9
3562	LOC_Os10g33970.1	10
3562	LOC_Os11g01869.1	11
3562	LOC_Os12g01916.1	12
3563	LOC_Os02g02540.1	2
3563	LOC_Os02g54640.1	2
3563	LOC_Os04g49570.1	4
3563	LOC_Os06g06130.1	6
3563	LOC_Os06g46670.1	6
3563	LOC_Os07g01310.1	7
3563	LOC_Os09g25920.1	9
3563	LOC_Os09g25960.1	9
3563	LOC_Os09g25990.1	9
3563	LOC_Os09g26000.1	9
3563	LOC_Os09g26144.1	9
3563	LOC_Os09g26160.1	9
3564	LOC_Os01g46860.1	1
3564	LOC_Os03g05610.1	3
3564	LOC_Os03g05620.1	3
3564	LOC_Os03g04360.1	3
3564	LOC_Os03g05640.1	3
3564	LOC_Os04g10690.1	4
3564	LOC_Os04g10750.1	4
3564	LOC_Os04g10800.1	4
3564	LOC_Os06g21950.1	6
3564	LOC_Os08g45000.1	8
3564	LOC_Os10g30770.1	10
3564	LOC_Os10g30790.1	10
3565	LOC_Os03g59610.1	3
3565	LOC_Os03g61740.1	3

3565	LOC_Os03g63290.1	3
3565	LOC_Os04g10000.1	4
3565	LOC_Os04g10010.1	4
3565	LOC_Os04g33240.1	4
3565	LOC_Os07g46860.1	7
3565	LOC_Os07g46846.1	7
3565	LOC_Os07g46930.1	7
3565	LOC_Os07g46870.1	7
3565	LOC_Os07g46920.1	7
3565	LOC_Os07g49120.1	7
3566	LOC_Os01g64120.1	1
3566	LOC_Os03g45710.1	3
3566	LOC_Os03g50540.1	3
3566	LOC_Os03g61960.1	3
3566	LOC_Os03g48040.1	3
3566	LOC_Os04g33630.1	4
3566	LOC_Os05g48160.1	5
3566	LOC_Os05g37140.1	5
3566	LOC_Os07g01930.1	7
3566	LOC_Os07g30670.1	7
3566	LOC_Os08g01380.1	8
3566	LOC_Os09g26650.1	9
3567	LOC_Os01g73990.1	1
3567	LOC_Os02g42740.1	2
3567	LOC_Os02g43360.1	2
3567	LOC_Os02g55060.1	2
3567	LOC_Os03g20470.1	3
3567	LOC_Os05g01820.1	5
3567	LOC_Os07g12830.1	7
3567	LOC_Os09g16520.1	9
3567	LOC_Os10g35870.1	10
3567	LOC_Os10g35850.1	10
3567	LOC_Os10g37420.1	10
3567	LOC_Os12g12170.1	12
3568	LOC_Os01g01080.1	1
3568	LOC_Os01g56380.1	1
3568	LOC_Os02g33710.1	2
3568	LOC_Os04g04640.1	4
3568	LOC_Os05g43510.1	5
3568	LOC_Os07g25590.1	7
3568	LOC_Os08g04540.1	8
3568	LOC_Os08g04560.1	8
3568	LOC_Os08g36320.1	8
3568	LOC_Os10g01640.1	10
3568	LOC_Os10g23900.1	10
3568	LOC_Os10g26110.1	10
3569	LOC_Os06g31930.1	6
3569	LOC_Os06g31960.1	6
3569	LOC_Os06g32020.1	6
3569	LOC_Os06g32160.1	6
3569	LOC_Os06g31280.1	6
3569	LOC_Os06g31800.1	6

3569	LOC_Os06g31890.1	6
3569	LOC_Os06g32240.1	6
3569	LOC_Os06g32350.1	6
3569	LOC_Os06g32370.1	6
3569	LOC_Os06g32550.1	6
3569	LOC_Os06g32600.1	6
3570	LOC_Os01g14630.1	1
3570	LOC_Os01g50050.1	1
3570	LOC_Os01g50760.1	1
3570	LOC_Os02g44780.1	2
3570	LOC_Os04g56210.1	4
3570	LOC_Os04g56230.1	4
3570	LOC_Os05g46580.1	5
3570	LOC_Os05g50550.1	5
3570	LOC_Os06g46450.1	6
3570	LOC_Os07g39270.1	7
3570	LOC_Os08g09370.1	8
3570	LOC_Os12g17320.1	12
3571	LOC_Os01g25360.1	1
3571	LOC_Os01g37650.1	1
3571	LOC_Os01g37630.1	1
3571	LOC_Os01g57770.1	1
3571	LOC_Os01g70860.1	1
3571	LOC_Os01g70840.1	1
3571	LOC_Os01g70850.1	1
3571	LOC_Os05g30760.1	5
3571	LOC_Os07g41230.1	7
3571	LOC_Os08g01850.1	8
3571	LOC_Os11g02580.1	11
3571	LOC_Os12g02500.1	12
3572	LOC_Os01g37460.1	1
3572	LOC_Os01g59980.1	1
3572	LOC_Os02g10920.1	2
3572	LOC_Os02g17470.1	2
3572	LOC_Os03g50120.1	3
3572	LOC_Os03g50430.1	3
3572	LOC_Os04g02000.1	4
3572	LOC_Os06g04920.1	6
3572	LOC_Os06g22700.1	6
3572	LOC_Os07g22024.1	7
3572	LOC_Os07g30820.1	7
3572	LOC_Os08g41010.1	8
3573	LOC_Os01g10580.1	1
3573	LOC_Os02g07930.1	2
3573	LOC_Os02g39360.1	2
3573	LOC_Os02g43170.1	2
3573	LOC_Os04g41560.1	4
3573	LOC_Os04g45690.1	4
3573	LOC_Os05g11510.1	5
3573	LOC_Os06g05890.1	6
3573	LOC_Os06g45040.1	6
3573	LOC_Os06g49880.1	6

3573	LOC_Os09g35880.1	9
3573	LOC_Os12g10660.1	12
3574	LOC_Os02g02350.1	2
3574	LOC_Os02g10850.1	2
3574	LOC_Os02g21110.1	2
3574	LOC_Os02g30210.1	2
3574	LOC_Os02g36520.1	2
3574	LOC_Os04g31120.1	4
3574	LOC_Os06g44500.1	6
3574	LOC_Os07g05880.1	7
3574	LOC_Os07g47650.1	7
3574	LOC_Os09g38300.1	9
3574	LOC_Os10g21930.1	10
3574	LOC_Os10g24900.1	10
3575	LOC_Os01g58510.1	1
3575	LOC_Os02g10900.1	2
3575	LOC_Os02g27680.1	2
3575	LOC_Os10g17690.1	10
3575	LOC_Os11g12240.1	11
3575	LOC_Os11g15700.1	11
3575	LOC_Os11g38000.1	11
3575	LOC_Os11g39590.1	11
3575	LOC_Os11g46080.1	11
3575	LOC_Os12g10410.1	12
3575	LOC_Os12g31200.1	12
3575	LOC_Os12g32590.1	12
3576	LOC_Os01g16890.1	1
3576	LOC_Os01g59730.1	1
3576	LOC_Os01g70010.1	1
3576	LOC_Os02g49610.1	2
3576	LOC_Os03g13800.1	3
3576	LOC_Os05g41110.1	5
3576	LOC_Os06g16290.1	6
3576	LOC_Os07g05580.1	7
3576	LOC_Os07g12650.1	7
3576	LOC_Os08g23710.1	8
3576	LOC_Os09g32976.1	9
3576	LOC_Os10g03540.1	10
3577	LOC_Os01g21380.1	1
3577	LOC_Os01g41160.1	1
3577	LOC_Os01g59490.1	1
3577	LOC_Os03g05840.1	3
3577	LOC_Os04g14790.1	4
3577	LOC_Os06g08300.1	6
3577	LOC_Os07g06080.1	7
3577	LOC_Os07g34570.1	7
3577	LOC_Os09g33900.1	9
3577	LOC_Os10g37210.1	10
3577	LOC_Os12g35890.1	12
3577	LOC_Os12g43590.1	12
3578	LOC_Os01g49670.1	1
3578	LOC_Os02g04120.1	2

3578	LOC_Os02g07720.1	2
3578	LOC_Os02g56980.1	2
3578	LOC_Os07g08210.1	7
3578	LOC_Os08g06450.1	8
3578	LOC_Os08g12830.1	8
3578	LOC_Os09g17870.1	9
3578	LOC_Os10g24810.1	10
3578	LOC_Os10g42790.1	10
3578	LOC_Os11g03050.1	11
3578	LOC_Os12g02820.1	12
3579	LOC_Os01g14900.1	1
3579	LOC_Os01g19390.1	1
3579	LOC_Os01g44069.1	1
3579	LOC_Os02g02340.1	2
3579	LOC_Os03g52570.1	3
3579	LOC_Os03g61720.1	3
3579	LOC_Os05g37600.1	5
3579	LOC_Os05g20100.1	5
3579	LOC_Os08g03700.1	8
3579	LOC_Os10g27330.1	10
3579	LOC_Os11g45400.1	11
3579	LOC_Os12g37600.1	12
3580	LOC_Os01g53630.1	1
3580	LOC_Os03g22400.1	3
3580	LOC_Os03g29630.1	3
3580	LOC_Os04g54680.1	4
3580	LOC_Os05g11770.1	5
3580	LOC_Os05g34520.1	5
3580	LOC_Os06g29310.1	6
3580	LOC_Os11g01180.1	11
3580	LOC_Os11g17290.1	11
3580	LOC_Os12g01290.1	12
3580	LOC_Os12g03410.1	12
3580	LOC_Os12g41380.1	12
3581	LOC_Os01g12910.1	1
3581	LOC_Os01g12920.1	1
3581	LOC_Os01g65960.1	1
3581	LOC_Os02g44200.1	2
3581	LOC_Os02g44134.1	2
3581	LOC_Os03g48480.1	3
3581	LOC_Os04g35590.1	4
3581	LOC_Os04g46710.1	4
3581	LOC_Os04g46730.1	4
3581	LOC_Os05g04660.1	5
3581	LOC_Os07g27870.1	7
3581	LOC_Os09g34190.1	9
3582	LOC_Os04g10730.1	4
3582	LOC_Os05g07100.1	5
3582	LOC_Os05g09010.1	5
3582	LOC_Os05g26640.1	5
3582	LOC_Os05g33720.1	5
3582	LOC_Os06g20230.1	6

3582	LOC_Os08g06150.1	8
3582	LOC_Os08g08530.1	8
3582	LOC_Os08g14370.1	8
3582	LOC_Os08g28280.1	8
3582	LOC_Os08g28490.1	8
3582	LOC_Os12g33170.1	12
3583	LOC_Os02g39000.1	2
3583	LOC_Os02g42880.1	2
3583	LOC_Os02g44102.1	2
3583	LOC_Os02g52810.1	2
3583	LOC_Os02g57840.1	2
3583	LOC_Os03g02040.1	3
3583	LOC_Os04g45070.1	4
3583	LOC_Os08g36760.1	8
3583	LOC_Os09g28300.1	9
3583	LOC_Os10g17790.1	10
3583	LOC_Os10g36000.1	10
3583	LOC_Os11g40210.1	11
3584	LOC_Os01g09540.1	1
3584	LOC_Os03g21480.1	3
3584	LOC_Os05g09660.1	5
3584	LOC_Os05g09704.1	5
3584	LOC_Os05g09724.1	5
3584	LOC_Os05g09740.1	5
3584	LOC_Os05g10210.1	5
3584	LOC_Os05g10330.1	5
3584	LOC_Os06g04790.1	6
3584	LOC_Os06g36400.1	6
3584	LOC_Os07g27580.1	7
3584	LOC_Os07g48320.1	7
3585	LOC_Os01g54340.1	1
3585	LOC_Os01g65740.1	1
3585	LOC_Os01g68650.1	1
3585	LOC_Os01g74250.1	1
3585	LOC_Os02g48850.1	2
3585	LOC_Os03g06680.1	3
3585	LOC_Os03g58230.1	3
3585	LOC_Os05g44300.1	5
3585	LOC_Os07g08390.1	7
3585	LOC_Os10g28210.1	10
3585	LOC_Os11g25020.1	11
3585	LOC_Os11g25040.1	11
3586	LOC_Os02g47770.1	2
3586	LOC_Os03g50920.1	3
3586	LOC_Os04g35500.1	4
3586	LOC_Os05g50310.1	5
3586	LOC_Os06g23030.1	6
3586	LOC_Os09g24810.1	9
3586	LOC_Os09g24820.1	9
3586	LOC_Os09g29130.1	9
3586	LOC_Os11g03420.1	11
3586	LOC_Os11g13930.1	11

3586	LOC_Os12g03110.1	12
3586	LOC_Os12g10630.1	12
3587	LOC_Os01g61310.1	1
3587	LOC_Os02g07030.1	2
3587	LOC_Os02g41460.1	2
3587	LOC_Os02g56610.1	2
3587	LOC_Os04g43580.1	4
3587	LOC_Os05g28040.1	5
3587	LOC_Os05g39500.1	5
3587	LOC_Os06g46030.1	6
3587	LOC_Os07g04670.1	7
3587	LOC_Os08g09660.1	8
3587	LOC_Os08g14970.1	8
3587	LOC_Os10g33780.1	10
3588	LOC_Os01g10470.1	1
3588	LOC_Os01g15320.1	1
3588	LOC_Os01g25540.1	1
3588	LOC_Os01g25560.1	1
3588	LOC_Os01g70690.1	1
3588	LOC_Os03g22440.1	3
3588	LOC_Os04g54090.1	4
3588	LOC_Os05g11330.1	5
3588	LOC_Os10g18170.1	10
3588	LOC_Os11g26880.1	11
3588	LOC_Os12g35670.1	12
3588	LOC_Os12g35690.1	12
3589	LOC_Os01g61900.1	1
3589	LOC_Os02g01990.1	2
3589	LOC_Os02g05470.1	2
3589	LOC_Os03g04620.1	3
3589	LOC_Os05g38990.1	5
3589	LOC_Os05g51690.1	5
3589	LOC_Os06g48610.1	6
3589	LOC_Os07g15770.1	7
3589	LOC_Os10g32900.1	10
3589	LOC_Os10g41100.1	10
3589	LOC_Os12g01080.1	12
3589	LOC_Os12g16160.1	12
3590	LOC_Os03g14130.1	3
3590	LOC_Os10g05860.1	10
3590	LOC_Os10g05880.1	10
3590	LOC_Os10g05750.1	10
3590	LOC_Os10g05820.1	10
3590	LOC_Os10g05950.1	10
3590	LOC_Os10g05970.1	10
3590	LOC_Os10g06000.1	10
3590	LOC_Os10g05910.1	10
3590	LOC_Os10g05930.1	10
3590	LOC_Os10g05980.1	10
3590	LOC_Os10g05990.1	10
3591	LOC_Os01g14960.1	1
3591	LOC_Os02g47450.1	2

3591	LOC_Os04g58160.1	4
3591	LOC_Os04g58170.1	4
3591	LOC_Os05g02990.1	5
3591	LOC_Os05g38960.1	5
3591	LOC_Os05g35800.1	5
3591	LOC_Os07g27810.1	7
3591	LOC_Os08g04410.1	8
3591	LOC_Os08g16130.1	8
3591	LOC_Os09g10910.1	9
3591	LOC_Os10g35294.1	10
3592	LOC_Os07g02960.1	7
3592	LOC_Os07g02970.1	7
3592	LOC_Os07g02980.1	7
3592	LOC_Os07g02990.1	7
3592	LOC_Os07g03010.1	7
3592	LOC_Os07g03020.1	7
3592	LOC_Os07g03030.1	7
3592	LOC_Os07g03050.1	7
3592	LOC_Os07g03060.1	7
3592	LOC_Os07g18570.1	7
3592	LOC_Os07g03040.1	7
3592	LOC_Os12g29820.1	12
3593	LOC_Os04g11610.1	4
3593	LOC_Os05g49720.1	5
3593	LOC_Os06g20640.1	6
3593	LOC_Os06g34250.1	6
3593	LOC_Os07g14040.1	7
3593	LOC_Os07g26020.1	7
3593	LOC_Os08g11190.1	8
3593	LOC_Os08g21000.1	8
3593	LOC_Os08g28360.1	8
3593	LOC_Os09g19260.1	9
3593	LOC_Os10g09020.1	10
3593	LOC_Os10g16490.1	10
3594	LOC_Os01g05530.1	1
3594	LOC_Os02g21160.1	2
3594	LOC_Os02g22800.1	2
3594	LOC_Os03g39300.1	3
3594	LOC_Os03g40800.1	3
3594	LOC_Os04g12450.1	4
3594	LOC_Os04g17220.1	4
3594	LOC_Os04g34340.1	4
3594	LOC_Os05g32250.1	5
3594	LOC_Os07g12280.1	7
3594	LOC_Os07g49130.1	7
3594	LOC_Os08g44610.1	8
3595	LOC_Os02g45570.1	2
3595	LOC_Os02g53690.1	2
3595	LOC_Os02g47280.1	2
3595	LOC_Os03g47140.1	3
3595	LOC_Os03g51970.1	3
3595	LOC_Os04g48510.1	4

3595	LOC_Os04g51190.1	4
3595	LOC_Os06g10310.1	6
3595	LOC_Os06g02560.1	6
3595	LOC_Os07g28430.1	7
3595	LOC_Os11g35030.1	11
3595	LOC_Os12g29980.1	12
3596	LOC_Os01g33470.1	1
3596	LOC_Os02g49110.1	2
3596	LOC_Os05g16520.1	5
3596	LOC_Os05g16530.1	5
3596	LOC_Os05g13890.1	5
3596	LOC_Os05g16450.1	5
3596	LOC_Os07g28810.1	7
3596	LOC_Os09g08010.1	9
3596	LOC_Os09g10430.1	9
3596	LOC_Os09g10420.1	9
3596	LOC_Os10g36300.1	10
3596	LOC_Os12g07410.1	12
3597	LOC_Os01g69140.1	1
3597	LOC_Os01g69160.1	1
3597	LOC_Os01g69190.1	1
3597	LOC_Os01g69200.1	1
3597	LOC_Os02g46120.1	2
3597	LOC_Os03g03730.1	3
3597	LOC_Os03g52160.1	3
3597	LOC_Os03g63270.1	3
3597	LOC_Os03g63280.1	3
3597	LOC_Os04g49590.1	4
3597	LOC_Os07g19444.1	7
3597	LOC_Os08g41800.1	8
3598	LOC_Os01g06800.1	1
3598	LOC_Os01g37390.1	1
3598	LOC_Os03g11860.1	3
3598	LOC_Os03g14330.1	3
3598	LOC_Os03g29140.1	3
3598	LOC_Os04g13230.1	4
3598	LOC_Os04g24360.1	4
3598	LOC_Os07g38310.1	7
3598	LOC_Os09g04060.1	9
3598	LOC_Os10g02950.1	10
3598	LOC_Os11g24820.1	11
3598	LOC_Os12g22750.1	12
3599	LOC_Os01g13100.1	1
3599	LOC_Os01g13470.1	1
3599	LOC_Os01g57910.1	1
3599	LOC_Os02g03310.1	2
3599	LOC_Os02g57640.1	2
3599	LOC_Os03g02420.1	3
3599	LOC_Os03g42900.1	3
3599	LOC_Os06g23460.1	6
3599	LOC_Os07g25740.1	7
3599	LOC_Os10g27470.1	10

3599	LOC_Os10g35220.1	10
3599	LOC_Os10g41440.1	10
3599	LOC_Os12g40560.1	12
3600	LOC_Os02g56530.1	2
3600	LOC_Os03g05260.1	3
3600	LOC_Os03g04300.1	3
3600	LOC_Os03g63480.1	3
3600	LOC_Os04g41290.1	4
3600	LOC_Os04g48520.1	4
3600	LOC_Os07g25470.1	7
3600	LOC_Os07g26490.1	7
3600	LOC_Os07g29830.1	7
3600	LOC_Os07g32790.1	7
3600	LOC_Os08g42690.1	8
3600	LOC_Os10g32050.1	10
3600	LOC_Os11g04910.1	11
3601	LOC_Os01g40480.1	1
3601	LOC_Os02g44490.1	2
3601	LOC_Os02g57090.1	2
3601	LOC_Os03g44890.1	3
3601	LOC_Os04g39680.1	4
3601	LOC_Os04g58720.1	4
3601	LOC_Os04g59520.1	4
3601	LOC_Os05g30750.1	5
3601	LOC_Os05g35480.1	5
3601	LOC_Os06g41090.1	6
3601	LOC_Os07g07070.1	7
3601	LOC_Os07g30020.1	7
3601	LOC_Os12g08670.1	12
3602	LOC_Os01g07430.1	1
3602	LOC_Os01g09590.1	1
3602	LOC_Os01g52410.1	1
3602	LOC_Os02g02370.1	2
3602	LOC_Os02g09480.1	2
3602	LOC_Os03g25550.1	3
3602	LOC_Os03g29614.1	3
3602	LOC_Os05g28320.1	5
3602	LOC_Os05g46610.1	5
3602	LOC_Os08g05520.1	8
3602	LOC_Os11g03440.1	11
3602	LOC_Os12g03150.1	12
3602	LOC_Os12g33070.1	12
3603	LOC_Os01g06490.1	1
3603	LOC_Os03g26920.1	3
3603	LOC_Os04g25560.1	4
3603	LOC_Os04g32540.1	4
3603	LOC_Os06g08720.1	6
3603	LOC_Os07g46350.1	7
3603	LOC_Os09g28840.1	9
3603	LOC_Os10g01110.1	10
3603	LOC_Os10g39560.1	10
3603	LOC_Os11g10750.1	11

3603	LOC_Os11g24200.1	11
3603	LOC_Os11g24290.1	11
3603	LOC_Os12g15470.1	12
3604	LOC_Os01g48370.1	1
3604	LOC_Os01g55430.1	1
3604	LOC_Os01g64700.1	1
3604	LOC_Os02g47640.1	2
3604	LOC_Os03g22800.1	3
3604	LOC_Os04g59130.1	4
3604	LOC_Os05g36190.1	5
3604	LOC_Os05g43850.1	5
3604	LOC_Os05g48670.1	5
3604	LOC_Os07g47110.1	7
3604	LOC_Os08g01290.1	8
3604	LOC_Os11g06420.1	11
3604	LOC_Os12g06630.1	12
3605	LOC_Os04g53410.1	4
3605	LOC_Os08g03470.1	8
3605	LOC_Os08g13000.1	8
3605	LOC_Os08g13030.1	8
3605	LOC_Os08g13180.1	8
3605	LOC_Os10g28860.1	10
3605	LOC_Os10g28780.1	10
3605	LOC_Os10g28970.1	10
3605	LOC_Os10g28790.1	10
3605	LOC_Os10g28810.1	10
3605	LOC_Os11g24550.1	11
3605	LOC_Os11g41350.1	11
3605	LOC_Os11g41300.1	11
3606	LOC_Os01g07850.1	1
3606	LOC_Os02g07160.1	2
3606	LOC_Os02g17920.1	2
3606	LOC_Os03g16940.1	3
3606	LOC_Os03g45720.1	3
3606	LOC_Os04g45590.1	4
3606	LOC_Os05g07940.1	5
3606	LOC_Os05g14194.1	5
3606	LOC_Os05g22970.1	5
3606	LOC_Os06g06420.1	6
3606	LOC_Os07g06660.1	7
3606	LOC_Os07g46360.1	7
3606	LOC_Os08g09250.1	8
3607	LOC_Os01g46950.1	1
3607	LOC_Os02g08030.1	2
3607	LOC_Os02g36600.1	2
3607	LOC_Os03g26430.1	3
3607	LOC_Os03g53710.1	3
3607	LOC_Os04g38530.1	4
3607	LOC_Os04g51390.1	4
3607	LOC_Os04g56290.1	4
3607	LOC_Os04g38540.1	4
3607	LOC_Os05g49430.1	5

3607	LOC_Os08g14330.1	8
3607	LOC_Os09g15820.1	9
3607	LOC_Os10g06720.1	10
3608	LOC_Os01g15000.1	1
3608	LOC_Os01g43140.1	1
3608	LOC_Os01g67420.1	1
3608	LOC_Os01g73740.1	1
3608	LOC_Os02g28040.1	2
3608	LOC_Os02g52830.1	2
3608	LOC_Os04g41200.1	4
3608	LOC_Os04g56240.1	4
3608	LOC_Os05g06140.1	5
3608	LOC_Os06g10850.1	6
3608	LOC_Os09g22450.1	9
3608	LOC_Os09g39790.1	9
3608	LOC_Os11g09010.1	11
3609	LOC_Os02g36780.1	2
3609	LOC_Os02g39040.1	2
3609	LOC_Os03g24590.1	3
3609	LOC_Os05g33440.1	5
3609	LOC_Os05g33460.1	5
3609	LOC_Os05g33500.1	5
3609	LOC_Os05g34160.1	5
3609	LOC_Os07g04230.1	7
3609	LOC_Os07g22670.1	7
3609	LOC_Os07g24090.1	7
3609	LOC_Os08g40630.1	8
3609	LOC_Os08g40430.1	8
3609	LOC_Os09g38720.1	9
3610	LOC_Os01g61190.1	1
3610	LOC_Os02g36619.1	2
3610	LOC_Os03g33520.1	3
3610	LOC_Os04g58870.1	4
3610	LOC_Os04g58880.1	4
3610	LOC_Os06g08460.1	6
3610	LOC_Os08g35470.1	8
3610	LOC_Os08g40840.1	8
3610	LOC_Os09g26820.1	9
3610	LOC_Os11g05880.1	11
3610	LOC_Os11g06700.1	11
3610	LOC_Os11g42989.1	11
3610	LOC_Os11g43049.1	11
3611	LOC_Os01g05930.1	1
3611	LOC_Os01g37310.1	1
3611	LOC_Os03g24970.1	3
3611	LOC_Os03g24980.1	3
3611	LOC_Os04g53090.1	4
3611	LOC_Os06g14850.1	6
3611	LOC_Os07g04480.1	7
3611	LOC_Os07g13020.1	7
3611	LOC_Os07g35710.1	7
3611	LOC_Os07g43400.1	7

3611	LOC_Os09g13880.1	9
3611	LOC_Os11g32970.1	11
3611	LOC_Os12g24870.1	12
3612	LOC_Os01g16320.1	1
3612	LOC_Os01g45510.1	1
3612	LOC_Os01g59200.1	1
3612	LOC_Os02g47980.1	2
3612	LOC_Os03g52290.1	3
3612	LOC_Os03g59690.1	3
3612	LOC_Os04g54600.1	4
3612	LOC_Os05g20030.1	5
3612	LOC_Os05g50160.1	5
3612	LOC_Os06g30030.1	6
3612	LOC_Os08g07500.1	8
3612	LOC_Os08g37150.1	8
3612	LOC_Os09g28880.1	9
3613	LOC_Os02g49970.1	2
3613	LOC_Os03g08330.1	3
3613	LOC_Os03g08310.1	3
3613	LOC_Os03g08320.1	3
3613	LOC_Os03g27900.1	3
3613	LOC_Os04g32480.1	4
3613	LOC_Os04g55920.1	4
3613	LOC_Os07g05830.1	7
3613	LOC_Os08g33160.1	8
3613	LOC_Os09g23660.1	9
3613	LOC_Os09g26780.1	9
3613	LOC_Os10g25230.1	10
3613	LOC_Os10g25250.1	10
3614	LOC_Os03g18960.1	3
3614	LOC_Os03g60890.1	3
3614	LOC_Os03g56660.1	3
3614	LOC_Os08g27170.1	8
3614	LOC_Os11g44170.1	11
3614	LOC_Os11g44310.1	11
3614	LOC_Os11g44630.1	11
3614	LOC_Os11g44680.1	11
3614	LOC_Os11g44700.1	11
3614	LOC_Os12g36110.1	12
3614	LOC_Os12g36910.1	12
3614	LOC_Os12g36920.1	12
3614	LOC_Os12g36940.1	12
3615	LOC_Os01g03240.1	1
3615	LOC_Os01g59040.1	1
3615	LOC_Os02g19350.1	2
3615	LOC_Os02g19360.1	2
3615	LOC_Os03g40460.1	3
3615	LOC_Os08g18200.1	8
3615	LOC_Os08g18790.1	8
3615	LOC_Os09g35530.1	9
3615	LOC_Os09g37140.1	9
3615	LOC_Os09g37150.1	9

3615	LOC_Os11g14710.1	11
3615	LOC_Os11g14720.1	11
3615	LOC_Os12g37440.1	12
3616	LOC_Os01g56400.1	1
3616	LOC_Os01g73530.1	1
3616	LOC_Os01g11946.1	1
3616	LOC_Os02g56550.1	2
3616	LOC_Os03g20170.1	3
3616	LOC_Os03g21490.1	3
3616	LOC_Os03g46740.1	3
3616	LOC_Os04g53550.1	4
3616	LOC_Os04g56330.1	4
3616	LOC_Os05g01700.1	5
3616	LOC_Os05g31910.1	5
3616	LOC_Os06g48060.1	6
3616	LOC_Os08g30740.1	8
3616	LOC_Os11g29850.1	11
3617	LOC_Os01g55690.1	1
3617	LOC_Os01g55630.1	1
3617	LOC_Os02g14600.1	2
3617	LOC_Os02g15070.1	2
3617	LOC_Os02g15090.1	2
3617	LOC_Os02g15150.1	2
3617	LOC_Os02g15169.1	2
3617	LOC_Os02g15178.1	2
3617	LOC_Os02g16820.1	2
3617	LOC_Os02g16830.1	2
3617	LOC_Os02g25640.1	2
3617	LOC_Os03g31360.1	3
3617	LOC_Os08g03410.1	8
3617	LOC_Os10g26060.1	10
3618	LOC_Os01g45400.1	1
3618	LOC_Os01g45420.1	1
3618	LOC_Os02g06640.1	2
3618	LOC_Os04g53620.1	4
3618	LOC_Os05g42424.1	5
3618	LOC_Os06g44080.1	6
3618	LOC_Os06g46120.1	6
3618	LOC_Os06g46770.1	6
3618	LOC_Os07g30990.1	7
3618	LOC_Os08g08760.1	8
3618	LOC_Os09g25320.1	9
3618	LOC_Os09g31019.1	9
3618	LOC_Os10g33620.1	10
3618	LOC_Os11g18670.1	11
3619	LOC_Os01g44250.1	1
3619	LOC_Os01g44360.1	1
3619	LOC_Os01g50860.1	1
3619	LOC_Os01g69240.1	1
3619	LOC_Os01g40420.1	1
3619	LOC_Os02g06410.1	2
3619	LOC_Os02g57280.1	2

3619	LOC_Os03g52690.1	3
3619	LOC_Os03g63940.1	3
3619	LOC_Os04g05010.1	4
3619	LOC_Os04g31340.1	4
3619	LOC_Os04g32880.1	4
3619	LOC_Os08g22149.1	8
3619	LOC_Os09g02710.1	9
3620	LOC_Os01g16530.1	1
3620	LOC_Os01g32350.1	1
3620	LOC_Os02g42290.1	2
3620	LOC_Os03g19510.1	3
3620	LOC_Os03g22430.1	3
3620	LOC_Os03g29810.1	3
3620	LOC_Os04g44400.1	4
3620	LOC_Os05g51450.1	5
3620	LOC_Os06g04530.1	6
3620	LOC_Os06g39720.1	6
3620	LOC_Os08g15270.1	8
3620	LOC_Os10g21300.1	10
3620	LOC_Os10g43050.1	10
3620	LOC_Os12g10590.1	12
3621	LOC_Os01g11414.1	1
3621	LOC_Os01g37690.1	1
3621	LOC_Os02g04630.1	2
3621	LOC_Os02g21009.1	2
3621	LOC_Os03g08230.1	3
3621	LOC_Os03g45370.1	3
3621	LOC_Os03g27960.1	3
3621	LOC_Os04g55940.1	4
3621	LOC_Os05g51610.1	5
3621	LOC_Os10g30070.1	10
3621	LOC_Os11g01580.1	11
3621	LOC_Os11g05070.1	11
3621	LOC_Os11g43860.1	11
3621	LOC_Os12g42910.1	12
3622	LOC_Os01g42810.1	1
3622	LOC_Os01g46410.1	1
3622	LOC_Os01g61460.1	1
3622	LOC_Os01g68630.1	1
3622	LOC_Os02g49650.1	2
3622	LOC_Os02g53380.1	2
3622	LOC_Os03g60340.1	3
3622	LOC_Os03g60350.1	3
3622	LOC_Os04g42900.1	4
3622	LOC_Os05g28630.1	5
3622	LOC_Os05g51020.1	5
3622	LOC_Os06g10560.1	6
3622	LOC_Os06g12870.1	6
3622	LOC_Os07g15320.1	7
3623	LOC_Os01g12160.1	1
3623	LOC_Os01g55940.1	1
3623	LOC_Os01g57610.1	1

3623	LOC_Os05g05180.1	5
3623	LOC_Os05g42150.1	5
3623	LOC_Os05g50890.1	5
3623	LOC_Os06g30440.1	6
3623	LOC_Os07g38860.1	7
3623	LOC_Os07g38890.1	7
3623	LOC_Os07g40290.1	7
3623	LOC_Os07g47490.1	7
3623	LOC_Os11g08340.1	11
3623	LOC_Os11g32510.1	11
3623	LOC_Os11g32520.1	11
3624	LOC_Os02g55300.1	2
3624	LOC_Os04g39260.1	4
3624	LOC_Os04g58810.1	4
3624	LOC_Os06g04670.1	6
3624	LOC_Os06g30770.1	6
3624	LOC_Os06g30790.1	6
3624	LOC_Os06g34520.1	6
3624	LOC_Os07g07430.1	7
3624	LOC_Os08g34170.1	8
3624	LOC_Os08g34160.1	8
3624	LOC_Os09g24990.1	9
3624	LOC_Os10g03530.1	10
3624	LOC_Os10g03560.1	10
3624	LOC_Os10g03880.1	10
3625	LOC_Os01g07360.1	1
3625	LOC_Os01g07370.1	1
3625	LOC_Os01g61910.1	1
3625	LOC_Os01g74510.1	1
3625	LOC_Os03g01980.1	3
3625	LOC_Os03g06510.1	3
3625	LOC_Os03g43684.1	3
3625	LOC_Os05g02790.1	5
3625	LOC_Os05g07680.1	5
3625	LOC_Os05g39000.1	5
3625	LOC_Os07g47090.1	7
3625	LOC_Os07g49480.1	7
3625	LOC_Os10g28610.1	10
3625	LOC_Os12g41200.1	12
3626	LOC_Os01g14140.1	1
3626	LOC_Os01g44090.1	1
3626	LOC_Os01g55820.1	1
3626	LOC_Os02g29980.1	2
3626	LOC_Os03g30830.1	3
3626	LOC_Os03g54910.1	3
3626	LOC_Os03g61780.1	3
3626	LOC_Os05g43690.1	5
3626	LOC_Os05g50490.1	5
3626	LOC_Os06g07240.1	6
3626	LOC_Os06g34660.1	6
3626	LOC_Os07g40940.1	7
3626	LOC_Os07g43940.1	7

3626	LOC_Os08g04140.1	8
3627	LOC_Os01g62960.1	1
3627	LOC_Os03g25364.1	3
3627	LOC_Os03g46046.1	3
3627	LOC_Os04g17040.1	4
3627	LOC_Os07g11690.1	7
3627	LOC_Os07g31220.1	7
3627	LOC_Os08g06750.1	8
3627	LOC_Os08g26100.1	8
3627	LOC_Os09g09740.1	9
3627	LOC_Os09g11930.1	9
3627	LOC_Os10g05390.1	10
3627	LOC_Os10g19990.1	10
3627	LOC_Os12g06120.1	12
3627	LOC_Os12g32010.1	12
3628	LOC_Os02g12360.1	2
3628	LOC_Os02g15750.1	2
3628	LOC_Os03g48190.1	3
3628	LOC_Os05g02630.1	5
3628	LOC_Os05g03310.1	5
3628	LOC_Os05g03330.1	5
3628	LOC_Os05g13850.1	5
3628	LOC_Os06g30060.1	6
3628	LOC_Os06g21890.1	6
3628	LOC_Os07g09580.1	7
3628	LOC_Os09g29900.1	9
3628	LOC_Os09g14614.1	9
3628	LOC_Os10g04280.1	10
3628	LOC_Os11g06150.1	11
3629	LOC_Os01g50020.1	1
3629	LOC_Os02g21320.1	2
3629	LOC_Os02g27350.1	2
3629	LOC_Os02g48940.1	2
3629	LOC_Os02g54790.1	2
3629	LOC_Os02g56450.1	2
3629	LOC_Os04g10250.1	4
3629	LOC_Os05g16490.1	5
3629	LOC_Os05g11310.1	5
3629	LOC_Os06g06310.1	6
3629	LOC_Os07g08940.1	7
3629	LOC_Os08g23280.1	8
3629	LOC_Os09g04770.1	9
3629	LOC_Os12g12190.1	12
3630	LOC_Os01g10040.1	1
3630	LOC_Os03g61980.1	3
3630	LOC_Os05g01120.1	5
3630	LOC_Os05g11130.1	5
3630	LOC_Os05g40384.1	5
3630	LOC_Os07g33420.1	7
3630	LOC_Os07g33440.1	7
3630	LOC_Os07g33550.1	7
3630	LOC_Os07g33560.1	7

3630	LOC_Os07g33580.1	7
3630	LOC_Os07g33610.1	7
3630	LOC_Os08g36860.1	8
3630	LOC_Os09g28390.1	9
3630	LOC_Os11g04710.1	11
3630	LOC_Os12g04480.1	12
3631	LOC_Os01g01312.1	1
3631	LOC_Os02g02290.1	2
3631	LOC_Os02g52510.1	2
3631	LOC_Os02g06592.1	2
3631	LOC_Os03g01200.1	3
3631	LOC_Os03g22900.1	3
3631	LOC_Os03g51230.1	3
3631	LOC_Os04g59620.1	4
3631	LOC_Os05g05780.1	5
3631	LOC_Os05g15890.1	5
3631	LOC_Os07g44210.1	7
3631	LOC_Os07g46590.1	7
3631	LOC_Os07g49210.1	7
3631	LOC_Os09g27060.1	9
3631	LOC_Os10g31970.1	10
3632	LOC_Os01g42860.1	1
3632	LOC_Os02g03140.1	2
3632	LOC_Os02g03150.1	2
3632	LOC_Os02g03170.1	2
3632	LOC_Os02g03180.1	2
3632	LOC_Os02g03190.1	2
3632	LOC_Os05g01920.1	5
3632	LOC_Os05g25630.1	5
3632	LOC_Os06g21540.1	6
3632	LOC_Os08g34249.1	8
3632	LOC_Os08g34258.1	8
3632	LOC_Os11g17790.1	11
3632	LOC_Os12g36210.1	12
3632	LOC_Os12g36220.1	12
3632	LOC_Os12g36240.1	12
3633	LOC_Os01g01620.1	1
3633	LOC_Os01g47550.1	1
3633	LOC_Os01g66940.1	1
3633	LOC_Os01g63220.1	1
3633	LOC_Os02g41590.1	2
3633	LOC_Os03g06880.1	3
3633	LOC_Os03g40550.1	3
3633	LOC_Os03g52760.1	3
3633	LOC_Os04g43750.1	4
3633	LOC_Os05g09370.1	5
3633	LOC_Os06g12600.1	6
3633	LOC_Os08g02120.1	8
3633	LOC_Os08g45180.1	8
3633	LOC_Os10g32830.1	10
3633	LOC_Os10g42240.1	10
3634	LOC_Os01g13570.1	1

3634	LOC_Os01g47190.1	1
3634	LOC_Os02g22000.1	2
3634	LOC_Os02g51590.1	2
3634	LOC_Os03g01180.1	3
3634	LOC_Os04g01230.1	4
3634	LOC_Os04g14760.1	4
3634	LOC_Os05g04960.1	5
3634	LOC_Os06g01950.1	6
3634	LOC_Os08g37140.1	8
3634	LOC_Os09g11510.1	9
3634	LOC_Os11g04320.1	11
3634	LOC_Os11g05260.1	11
3634	LOC_Os11g31880.1	11
3634	LOC_Os12g04120.1	12
3635	LOC_Os01g17279.1	1
3635	LOC_Os01g43080.1	1
3635	LOC_Os02g26660.1	2
3635	LOC_Os02g47920.1	2
3635	LOC_Os03g18940.1	3
3635	LOC_Os04g23830.1	4
3635	LOC_Os04g55700.1	4
3635	LOC_Os04g53200.1	4
3635	LOC_Os05g01200.1	5
3635	LOC_Os06g24594.1	6
3635	LOC_Os08g28980.1	8
3635	LOC_Os10g26720.1	10
3635	LOC_Os10g26730.1	10
3635	LOC_Os11g32290.1	11
3635	LOC_Os12g08740.1	12
3636	LOC_Os01g24710.1	1
3636	LOC_Os01g25150.1	1
3636	LOC_Os01g25160.1	1
3636	LOC_Os01g25280.1	1
3636	LOC_Os01g51050.1	1
3636	LOC_Os03g28160.1	3
3636	LOC_Os04g03320.1	4
3636	LOC_Os04g03360.1	4
3636	LOC_Os05g05170.1	5
3636	LOC_Os05g43240.1	5
3636	LOC_Os06g07300.1	6
3636	LOC_Os06g07250.1	6
3636	LOC_Os06g12180.1	6
3636	LOC_Os10g04270.1	10
3636	LOC_Os11g39480.1	11
3637	LOC_Os01g27730.1	1
3637	LOC_Os01g53150.1	1
3637	LOC_Os01g12540.1	1
3637	LOC_Os03g22890.1	3
3637	LOC_Os03g44530.1	3
3637	LOC_Os03g51820.1	3
3637	LOC_Os03g60180.1	3
3637	LOC_Os05g35540.1	5

3637	LOC_Os08g10649.1	8
3637	LOC_Os08g31460.1	8
3637	LOC_Os10g42170.1	10
3637	LOC_Os11g26030.1	11
3637	LOC_Os11g38020.1	11
3637	LOC_Os11g41910.1	11
3637	LOC_Os12g42370.1	12
3638	LOC_Os01g52780.1	1
3638	LOC_Os02g02920.1	2
3638	LOC_Os02g51290.1	2
3638	LOC_Os03g04030.1	3
3638	LOC_Os03g14610.1	3
3638	LOC_Os04g33880.1	4
3638	LOC_Os06g12220.1	6
3638	LOC_Os07g38100.1	7
3638	LOC_Os08g36440.1	8
3638	LOC_Os09g27730.1	9
3638	LOC_Os09g36340.1	9
3638	LOC_Os10g09870.1	10
3638	LOC_Os11g05800.1	11
3638	LOC_Os11g30500.1	11
3638	LOC_Os11g38920.1	11
3639	LOC_Os01g01350.1	1
3639	LOC_Os01g18280.1	1
3639	LOC_Os02g38300.1	2
3639	LOC_Os03g01810.1	3
3639	LOC_Os03g43860.1	3
3639	LOC_Os05g01250.1	5
3639	LOC_Os06g40620.1	6
3639	LOC_Os06g43590.1	6
3639	LOC_Os07g13270.1	7
3639	LOC_Os07g29630.1	7
3639	LOC_Os09g09480.1	9
3639	LOC_Os10g33660.1	10
3639	LOC_Os11g03060.1	11
3639	LOC_Os11g47710.1	11
3639	LOC_Os12g02830.1	12
3640	LOC_Os01g40330.1	1
3640	LOC_Os01g72970.1	1
3640	LOC_Os02g07850.1	2
3640	LOC_Os02g43770.1	2
3640	LOC_Os03g20530.1	3
3640	LOC_Os04g47480.1	4
3640	LOC_Os05g07980.1	5
3640	LOC_Os05g35070.1	5
3640	LOC_Os05g16640.1	5
3640	LOC_Os07g11070.1	7
3640	LOC_Os07g48690.1	7
3640	LOC_Os08g43730.1	8
3640	LOC_Os09g20340.1	9
3640	LOC_Os09g37040.1	9
3640	LOC_Os09g37520.1	9

3641	LOC_Os02g15380.1	2
3641	LOC_Os02g24760.1	2
3641	LOC_Os02g58170.1	2
3641	LOC_Os04g01100.1	4
3641	LOC_Os04g16130.1	4
3641	LOC_Os05g46070.1	5
3641	LOC_Os06g07810.1	6
3641	LOC_Os08g07550.1	8
3641	LOC_Os08g17850.1	8
3641	LOC_Os08g31310.1	8
3641	LOC_Os10g17510.1	10
3641	LOC_Os11g39200.1	11
3641	LOC_Os12g10270.1	12
3641	LOC_Os12g29970.1	12
3641	LOC_Os12g31530.1	12
3642	LOC_Os01g18880.1	1
3642	LOC_Os02g26550.1	2
3642	LOC_Os02g32540.1	2
3642	LOC_Os03g08350.1	3
3642	LOC_Os03g44430.1	3
3642	LOC_Os03g46460.1	3
3642	LOC_Os03g46560.1	3
3642	LOC_Os03g57270.1	3
3642	LOC_Os04g24710.1	4
3642	LOC_Os04g46170.1	4
3642	LOC_Os05g49610.1	5
3642	LOC_Os06g15550.1	6
3642	LOC_Os09g29760.1	9
3642	LOC_Os10g36680.1	10
3642	LOC_Os11g34160.1	11
3643	LOC_Os02g07510.1	2
3643	LOC_Os02g14400.1	2
3643	LOC_Os04g02240.1	4
3643	LOC_Os04g09510.1	4
3643	LOC_Os04g16210.1	4
3643	LOC_Os04g16390.1	4
3643	LOC_Os04g19350.1	4
3643	LOC_Os06g15300.1	6
3643	LOC_Os08g12360.1	8
3643	LOC_Os09g05100.1	9
3643	LOC_Os09g06250.1	9
3643	LOC_Os10g26220.1	10
3643	LOC_Os11g20000.1	11
3643	LOC_Os11g27770.1	11
3643	LOC_Os11g27870.1	11
3644	LOC_Os02g26370.1	2
3644	LOC_Os03g56944.1	3
3644	LOC_Os03g56930.1	3
3644	LOC_Os03g56940.1	3
3644	LOC_Os05g50470.1	5
3644	LOC_Os07g04930.1	7
3644	LOC_Os07g04950.1	7

3644	LOC_Os07g04960.1	7
3644	LOC_Os07g28640.1	7
3644	LOC_Os07g37620.1	7
3644	LOC_Os07g40830.1	7
3644	LOC_Os07g40870.1	7
3644	LOC_Os07g40890.1	7
3644	LOC_Os07g40910.1	7
3644	LOC_Os08g37300.1	8
3645	LOC_Os04g01850.1	4
3645	LOC_Os04g25700.1	4
3645	LOC_Os04g28760.1	4
3645	LOC_Os06g26000.1	6
3645	LOC_Os06g28030.1	6
3645	LOC_Os07g15310.1	7
3645	LOC_Os08g07350.1	8
3645	LOC_Os08g13140.1	8
3645	LOC_Os08g30440.1	8
3645	LOC_Os08g31380.1	8
3645	LOC_Os09g02480.1	9
3645	LOC_Os10g07000.1	10
3645	LOC_Os10g18710.1	10
3645	LOC_Os10g23950.1	10
3645	LOC_Os12g26990.1	12
3646	LOC_Os01g51660.1	1
3646	LOC_Os01g59030.1	1
3646	LOC_Os02g03480.1	2
3646	LOC_Os02g12430.1	2
3646	LOC_Os02g55689.1	2
3646	LOC_Os02g55790.1	2
3646	LOC_Os02g55729.1	2
3646	LOC_Os03g42060.1	3
3646	LOC_Os04g02400.1	4
3646	LOC_Os04g09320.1	4
3646	LOC_Os06g02770.1	6
3646	LOC_Os10g31630.1	10
3646	LOC_Os10g33610.1	10
3646	LOC_Os10g35560.1	10
3646	LOC_Os12g39130.1	12
3647	LOC_Os01g18050.1	1
3647	LOC_Os01g59150.1	1
3647	LOC_Os02g07060.1	2
3647	LOC_Os03g01530.1	3
3647	LOC_Os03g11970.1	3
3647	LOC_Os03g44420.1	3
3647	LOC_Os03g45920.1	3
3647	LOC_Os03g56810.1	3
3647	LOC_Os03g51600.1	3
3647	LOC_Os04g56970.1	4
3647	LOC_Os05g06450.1	5
3647	LOC_Os05g37160.1	5
3647	LOC_Os05g34170.1	5
3647	LOC_Os06g46000.1	6

3647	LOC_Os07g38730.1	7
3647	LOC_Os11g14220.1	11
3648	LOC_Os01g43160.1	1
3648	LOC_Os01g66710.1	1
3648	LOC_Os02g15690.1	2
3648	LOC_Os02g54030.1	2
3648	LOC_Os03g03350.1	3
3648	LOC_Os03g59330.1	3
3648	LOC_Os03g61800.1	3
3648	LOC_Os05g50960.1	5
3648	LOC_Os07g10700.1	7
3648	LOC_Os07g10730.1	7
3648	LOC_Os07g14160.1	7
3648	LOC_Os08g01600.1	8
3648	LOC_Os08g23790.1	8
3648	LOC_Os09g26800.1	9
3648	LOC_Os09g31270.1	9
3648	LOC_Os12g36810.1	12
3649	LOC_Os01g74570.1	1
3649	LOC_Os02g12750.1	2
3649	LOC_Os02g49630.1	2
3649	LOC_Os03g63600.1	3
3649	LOC_Os03g63620.1	3
3649	LOC_Os05g03140.1	5
3649	LOC_Os05g03530.1	5
3649	LOC_Os06g37510.1	6
3649	LOC_Os06g44310.1	6
3649	LOC_Os08g06250.1	8
3649	LOC_Os08g16050.1	8
3649	LOC_Os08g18044.1	8
3649	LOC_Os08g34460.1	8
3649	LOC_Os09g25760.1	9
3649	LOC_Os10g35980.1	10
3649	LOC_Os12g14580.1	12
3650	LOC_Os02g36400.1	2
3650	LOC_Os02g46650.1	2
3650	LOC_Os02g55180.1	2
3650	LOC_Os03g09080.1	3
3650	LOC_Os03g06950.1	3
3650	LOC_Os03g09260.1	3
3650	LOC_Os04g37950.1	4
3650	LOC_Os04g55360.1	4
3650	LOC_Os05g43480.1	5
3650	LOC_Os06g08530.1	6
3650	LOC_Os07g06610.1	7
3650	LOC_Os07g46660.1	7
3650	LOC_Os09g24250.1	9
3650	LOC_Os09g28940.1	9
3650	LOC_Os11g34270.1	11
3650	LOC_Os12g42600.1	12
3651	LOC_Os01g20940.1	1
3651	LOC_Os01g24470.1	1

3651	LOC_Os01g29469.1	1
3651	LOC_Os01g53710.1	1
3651	LOC_Os01g64010.1	1
3651	LOC_Os02g48840.1	2
3651	LOC_Os03g01750.1	3
3651	LOC_Os05g44910.1	5
3651	LOC_Os06g05870.1	6
3651	LOC_Os06g20340.1	6
3651	LOC_Os10g41240.1	10
3651	LOC_Os11g02180.1	11
3651	LOC_Os11g04180.1	11
3651	LOC_Os12g02120.1	12
3651	LOC_Os12g03990.1	12
3651	LOC_Os12g05660.1	12
3652	LOC_Os01g65730.1	1
3652	LOC_Os02g39800.1	2
3652	LOC_Os02g49326.1	2
3652	LOC_Os02g50100.1	2
3652	LOC_Os03g19480.1	3
3652	LOC_Os05g50980.1	5
3652	LOC_Os06g16390.1	6
3652	LOC_Os06g03676.1	6
3652	LOC_Os07g28840.1	7
3652	LOC_Os08g08210.1	8
3652	LOC_Os08g14660.1	8
3652	LOC_Os08g34370.1	8
3652	LOC_Os09g13740.1	9
3652	LOC_Os09g24530.1	9
3652	LOC_Os12g13460.1	12
3652	LOC_Os12g41900.1	12
3653	LOC_Os04g25150.1	4
3653	LOC_Os04g25160.1	4
3653	LOC_Os04g25190.1	4
3653	LOC_Os04g26190.1	4
3653	LOC_Os04g26220.1	4
3653	LOC_Os04g26230.1	4
3653	LOC_Os06g44470.1	6
3653	LOC_Os06g45150.1	6
3653	LOC_Os06g45190.1	6
3653	LOC_Os06g45200.1	6
3653	LOC_Os06g45210.1	6
3653	LOC_Os06g45230.1	6
3653	LOC_Os06g45160.1	6
3653	LOC_Os06g45180.1	6
3653	LOC_Os06g45290.1	6
3653	LOC_Os06g50960.1	6
3654	LOC_Os01g17180.1	1
3654	LOC_Os01g32800.1	1
3654	LOC_Os03g02540.1	3
3654	LOC_Os03g10120.1	3
3654	LOC_Os03g11570.1	3
3654	LOC_Os03g37950.1	3

3654	LOC_Os03g63430.1	3
3654	LOC_Os04g01290.1	4
3654	LOC_Os04g36700.1	4
3654	LOC_Os07g07250.1	7
3654	LOC_Os07g12110.1	7
3654	LOC_Os07g32040.1	7
3654	LOC_Os08g43640.1	8
3654	LOC_Os08g02550.1	8
3654	LOC_Os09g21760.1	9
3654	LOC_Os09g37000.1	9
3655	LOC_Os01g01430.1	1
3655	LOC_Os01g66120.1	1
3655	LOC_Os01g70110.1	1
3655	LOC_Os02g36880.1	2
3655	LOC_Os02g57650.1	2
3655	LOC_Os03g21060.1	3
3655	LOC_Os03g42630.1	3
3655	LOC_Os03g59730.1	3
3655	LOC_Os05g34830.1	5
3655	LOC_Os05g37080.1	5
3655	LOC_Os07g27340.1	7
3655	LOC_Os08g23880.1	8
3655	LOC_Os09g24560.1	9
3655	LOC_Os10g33760.1	10
3655	LOC_Os12g05990.1	12
3655	LOC_Os12g41680.1	12
3656	LOC_Os01g04640.1	1
3656	LOC_Os01g12650.1	1
3656	LOC_Os01g49660.1	1
3656	LOC_Os01g53520.1	1
3656	LOC_Os01g63240.1	1
3656	LOC_Os02g16620.1	2
3656	LOC_Os02g19990.1	2
3656	LOC_Os03g21840.1	3
3656	LOC_Os03g56900.1	3
3656	LOC_Os04g29920.1	4
3656	LOC_Os04g57420.1	4
3656	LOC_Os05g24770.1	5
3656	LOC_Os05g45050.1	5
3656	LOC_Os05g47690.1	5
3656	LOC_Os06g30750.1	6
3656	LOC_Os07g04910.1	7
3657	LOC_Os01g74110.1	1
3657	LOC_Os02g10230.1	2
3657	LOC_Os03g29850.1	3
3657	LOC_Os03g46454.1	3
3657	LOC_Os03g46470.1	3
3657	LOC_Os04g52310.1	4
3657	LOC_Os05g07210.1	5
3657	LOC_Os05g25194.1	5
3657	LOC_Os05g10940.1	5
3657	LOC_Os05g39540.1	5

3657	LOC_Os05g39560.1	5
3657	LOC_Os06g37010.1	6
3657	LOC_Os07g12890.1	7
3657	LOC_Os08g01030.1	8
3657	LOC_Os08g10630.1	8
3657	LOC_Os08g36420.1	8
3658	LOC_Os01g46350.1	1
3658	LOC_Os03g18120.1	3
3658	LOC_Os03g19470.1	3
3658	LOC_Os03g60300.1	3
3658	LOC_Os06g12810.1	6
3658	LOC_Os06g12840.1	6
3658	LOC_Os06g12850.1	6
3658	LOC_Os06g16170.1	6
3658	LOC_Os06g16160.1	6
3658	LOC_Os06g16200.1	6
3658	LOC_Os06g16240.1	6
3658	LOC_Os06g34060.1	6
3658	LOC_Os09g20850.1	9
3658	LOC_Os11g37630.1	11
3658	LOC_Os12g01380.1	12
3658	LOC_Os12g05080.1	12
3659	LOC_Os01g53240.1	1
3659	LOC_Os02g18690.1	2
3659	LOC_Os04g14990.1	4
3659	LOC_Os05g12400.1	5
3659	LOC_Os05g12410.1	5
3659	LOC_Os05g13490.1	5
3659	LOC_Os05g12640.1	5
3659	LOC_Os06g13240.1	6
3659	LOC_Os06g17000.1	6
3659	LOC_Os06g19800.1	6
3659	LOC_Os08g29200.1	8
3659	LOC_Os08g38810.1	8
3659	LOC_Os09g16010.1	9
3659	LOC_Os09g30320.1	9
3659	LOC_Os10g26940.1	10
3659	LOC_Os11g06980.1	11
3660	LOC_Os01g13860.1	1
3660	LOC_Os01g38880.1	1
3660	LOC_Os01g49260.1	1
3660	LOC_Os02g55040.1	2
3660	LOC_Os03g02090.1	3
3660	LOC_Os03g26784.1	3
3660	LOC_Os03g47920.1	3
3660	LOC_Os03g51490.1	3
3660	LOC_Os03g57600.1	3
3660	LOC_Os03g58000.1	3
3660	LOC_Os05g23230.1	5
3660	LOC_Os06g06610.1	6
3660	LOC_Os09g27670.1	9
3660	LOC_Os09g35540.1	9

3660	LOC_Os12g06550.1	12
3660	LOC_Os12g07890.1	12
3661	LOC_Os02g35940.1	2
3661	LOC_Os02g53960.1	2
3661	LOC_Os06g11090.1	6
3661	LOC_Os06g11130.1	6
3661	LOC_Os07g31790.1	7
3661	LOC_Os07g34240.1	7
3661	LOC_Os07g34280.1	7
3661	LOC_Os08g37030.1	8
3661	LOC_Os08g37050.1	8
3661	LOC_Os08g37060.1	8
3661	LOC_Os09g28620.1	9
3661	LOC_Os09g28660.1	9
3661	LOC_Os09g28690.1	9
3661	LOC_Os09g28750.1	9
3661	LOC_Os09g28630.1	9
3661	LOC_Os09g28740.1	9
3662	LOC_Os01g10260.1	1
3662	LOC_Os01g10310.1	1
3662	LOC_Os01g54140.1	1
3662	LOC_Os01g54170.1	1
3662	LOC_Os02g13620.1	2
3662	LOC_Os03g08250.1	3
3662	LOC_Os03g06430.1	3
3662	LOC_Os03g29360.1	3
3662	LOC_Os03g46300.1	3
3662	LOC_Os03g53540.1	3
3662	LOC_Os05g11300.1	5
3662	LOC_Os05g44410.1	5
3662	LOC_Os06g09900.1	6
3662	LOC_Os06g36040.1	6
3662	LOC_Os07g42220.1	7
3662	LOC_Os10g28700.1	10
3663	LOC_Os03g42450.1	3
3663	LOC_Os03g42490.1	3
3663	LOC_Os03g42500.1	3
3663	LOC_Os05g11960.1	5
3663	LOC_Os07g32140.1	7
3663	LOC_Os07g32830.1	7
3663	LOC_Os07g32839.1	7
3663	LOC_Os07g32850.1	7
3663	LOC_Os07g32870.1	7
3663	LOC_Os07g33040.1	7
3663	LOC_Os07g33070.1	7
3663	LOC_Os07g32960.1	7
3663	LOC_Os07g32979.1	7
3663	LOC_Os07g32990.1	7
3663	LOC_Os07g33030.1	7
3663	LOC_Os07g33090.1	7
3664	LOC_Os01g15060.1	1
3664	LOC_Os04g08640.1	4

3664	LOC_Os06g17560.1	6
3664	LOC_Os06g17600.1	6
3664	LOC_Os06g18850.1	6
3664	LOC_Os06g18930.1	6
3664	LOC_Os06g18880.1	6
3664	LOC_Os06g18900.1	6
3664	LOC_Os06g19010.1	6
3664	LOC_Os06g19170.1	6
3664	LOC_Os06g19210.1	6
3664	LOC_Os06g19110.1	6
3664	LOC_Os06g19130.1	6
3664	LOC_Os06g19260.1	6
3664	LOC_Os06g19300.1	6
3664	LOC_Os06g19370.1	6
3665	LOC_Os01g16414.1	1
3665	LOC_Os01g73310.1	1
3665	LOC_Os01g64630.1	1
3665	LOC_Os02g38340.1	2
3665	LOC_Os03g50890.1	3
3665	LOC_Os03g56970.1	3
3665	LOC_Os03g61970.1	3
3665	LOC_Os04g57210.1	4
3665	LOC_Os05g01600.1	5
3665	LOC_Os05g36290.1	5
3665	LOC_Os08g03440.1	8
3665	LOC_Os08g04280.1	8
3665	LOC_Os08g28190.1	8
3665	LOC_Os10g36650.1	10
3665	LOC_Os11g06390.1	11
3665	LOC_Os12g06660.1	12
3665	LOC_Os12g44350.1	12
3666	LOC_Os01g16200.1	1
3666	LOC_Os01g56010.1	1
3666	LOC_Os03g41419.1	3
3666	LOC_Os03g41438.1	3
3666	LOC_Os04g45110.1	4
3666	LOC_Os04g45120.1	4
3666	LOC_Os05g43590.1	5
3666	LOC_Os11g11500.1	11
3666	LOC_Os11g11720.1	11
3666	LOC_Os11g12410.1	11
3666	LOC_Os11g12520.1	11
3666	LOC_Os11g11760.1	11
3666	LOC_Os11g12420.1	11
3666	LOC_Os11g12460.1	11
3666	LOC_Os11g13530.1	11
3666	LOC_Os11g13540.1	11
3666	LOC_Os11g13560.1	11
3667	LOC_Os01g53100.1	1
3667	LOC_Os01g67950.1	1
3667	LOC_Os02g38410.1	2
3667	LOC_Os02g41820.1	2

3667	LOC_Os03g24920.1	3
3667	LOC_Os03g59370.1	3
3667	LOC_Os04g40680.1	4
3667	LOC_Os05g38310.1	5
3667	LOC_Os06g05760.1	6
3667	LOC_Os07g31540.1	7
3667	LOC_Os08g08790.1	8
3667	LOC_Os08g08700.1	8
3667	LOC_Os08g19830.1	8
3667	LOC_Os10g34960.1	10
3667	LOC_Os10g34990.1	10
3667	LOC_Os10g39590.1	10
3667	LOC_Os11g04880.1	11
3668	LOC_Os01g67530.1	1
3668	LOC_Os01g67540.1	1
3668	LOC_Os02g02700.1	2
3668	LOC_Os02g08100.1	2
3668	LOC_Os02g46970.1	2
3668	LOC_Os03g03790.1	3
3668	LOC_Os03g04000.1	3
3668	LOC_Os04g24530.1	4
3668	LOC_Os04g57850.1	4
3668	LOC_Os04g58710.1	4
3668	LOC_Os06g44620.1	6
3668	LOC_Os07g44560.1	7
3668	LOC_Os08g04770.1	8
3668	LOC_Os08g14760.1	8
3668	LOC_Os08g34790.1	8
3668	LOC_Os09g38350.1	9
3668	LOC_Os10g42800.1	10
3669	LOC_Os01g41710.1	1
3669	LOC_Os01g52240.1	1
3669	LOC_Os01g64960.1	1
3669	LOC_Os02g10390.1	2
3669	LOC_Os02g52650.1	2
3669	LOC_Os03g39610.1	3
3669	LOC_Os04g38410.1	4
3669	LOC_Os04g59440.1	4
3669	LOC_Os06g21590.1	6
3669	LOC_Os07g37240.1	7
3669	LOC_Os07g37550.1	7
3669	LOC_Os07g38960.1	7
3669	LOC_Os08g33820.1	8
3669	LOC_Os09g12540.1	9
3669	LOC_Os09g17740.1	9
3669	LOC_Os09g26810.1	9
3669	LOC_Os11g13890.1	11
3670	LOC_Os01g01010.1	1
3670	LOC_Os01g61860.1	1
3670	LOC_Os01g68010.1	1
3670	LOC_Os02g48000.1	2
3670	LOC_Os02g56570.1	2

3670	LOC_Os03g17540.1	3
3670	LOC_Os04g30880.1	4
3670	LOC_Os05g38950.1	5
3670	LOC_Os07g34130.1	7
3670	LOC_Os08g25010.1	8
3670	LOC_Os08g31840.1	8
3670	LOC_Os08g43380.1	8
3670	LOC_Os09g34040.1	9
3670	LOC_Os09g35960.1	9
3670	LOC_Os10g37410.1	10
3670	LOC_Os11g28990.1	11
3670	LOC_Os11g37690.1	11
3671	LOC_Os01g08820.1	1
3671	LOC_Os01g65600.1	1
3671	LOC_Os01g66070.1	1
3671	LOC_Os02g09910.1	2
3671	LOC_Os02g09920.1	2
3671	LOC_Os02g52960.1	2
3671	LOC_Os03g50780.1	3
3671	LOC_Os03g53700.1	3
3671	LOC_Os03g04980.1	3
3671	LOC_Os04g14510.1	4
3671	LOC_Os04g34720.1	4
3671	LOC_Os04g52020.1	4
3671	LOC_Os06g10690.1	6
3671	LOC_Os06g51490.1	6
3671	LOC_Os08g01420.1	8
3671	LOC_Os09g27620.1	9
3671	LOC_Os11g12650.1	11
3672	LOC_Os02g08150.1	2
3672	LOC_Os02g39710.1	2
3672	LOC_Os02g49230.1	2
3672	LOC_Os02g49880.1	2
3672	LOC_Os03g22770.1	3
3672	LOC_Os03g50310.1	3
3672	LOC_Os04g42020.1	4
3672	LOC_Os06g01340.1	6
3672	LOC_Os06g15330.1	6
3672	LOC_Os06g16370.1	6
3672	LOC_Os06g19444.1	6
3672	LOC_Os06g44450.1	6
3672	LOC_Os07g47140.1	7
3672	LOC_Os08g15050.1	8
3672	LOC_Os08g42440.1	8
3672	LOC_Os09g06464.1	9
3672	LOC_Os09g33550.1	9
3673	LOC_Os02g16000.1	2
3673	LOC_Os03g48120.1	3
3673	LOC_Os05g27880.1	5
3673	LOC_Os05g44530.1	5
3673	LOC_Os06g21330.1	6
3673	LOC_Os06g31100.1	6

3673	LOC_Os06g45770.1	6
3673	LOC_Os06g46240.1	6
3673	LOC_Os07g15490.1	7
3673	LOC_Os08g01320.1	8
3673	LOC_Os08g38700.1	8
3673	LOC_Os08g40460.1	8
3673	LOC_Os10g28870.1	10
3673	LOC_Os10g29360.1	10
3673	LOC_Os10g30040.1	10
3673	LOC_Os11g02070.1	11
3673	LOC_Os11g41290.1	11
3674	LOC_Os01g12210.1	1
3674	LOC_Os01g53570.1	1
3674	LOC_Os02g03280.1	2
3674	LOC_Os02g45160.1	2
3674	LOC_Os02g49790.1	2
3674	LOC_Os03g53400.1	3
3674	LOC_Os03g58140.1	3
3674	LOC_Os03g58150.1	3
3674	LOC_Os04g34010.1	4
3674	LOC_Os04g47930.1	4
3674	LOC_Os05g33360.1	5
3674	LOC_Os06g15779.1	6
3674	LOC_Os06g22600.1	6
3674	LOC_Os07g08060.1	7
3674	LOC_Os07g08070.1	7
3674	LOC_Os10g42180.1	10
3674	LOC_Os11g37200.1	11
3675	LOC_Os01g19770.1	1
3675	LOC_Os02g45100.1	2
3675	LOC_Os02g48610.1	2
3675	LOC_Os03g02390.1	3
3675	LOC_Os03g09870.1	3
3675	LOC_Os03g19290.1	3
3675	LOC_Os03g30200.1	3
3675	LOC_Os03g30220.1	3
3675	LOC_Os03g30230.1	3
3675	LOC_Os03g18500.1	3
3675	LOC_Os04g30740.1	4
3675	LOC_Os05g02060.1	5
3675	LOC_Os05g07250.1	5
3675	LOC_Os07g41330.1	7
3675	LOC_Os09g10740.1	9
3675	LOC_Os10g37530.1	10
3675	LOC_Os12g33020.1	12
3676	LOC_Os01g06580.1	1
3676	LOC_Os01g47780.1	1
3676	LOC_Os02g20540.1	2
3676	LOC_Os02g20560.1	2
3676	LOC_Os03g57460.1	3
3676	LOC_Os03g57490.1	3
3676	LOC_Os04g39590.1	4

3676	LOC_Os04g39600.1	4
3676	LOC_Os05g07060.1	5
3676	LOC_Os05g48890.1	5
3676	LOC_Os05g48900.1	5
3676	LOC_Os06g17460.1	6
3676	LOC_Os07g06680.1	7
3676	LOC_Os08g38270.1	8
3676	LOC_Os08g39270.1	8
3676	LOC_Os09g30010.1	9
3676	LOC_Os09g30486.1	9
3677	LOC_Os02g17534.1	2
3677	LOC_Os02g52560.1	2
3677	LOC_Os02g52640.1	2
3677	LOC_Os02g52590.1	2
3677	LOC_Os02g52610.1	2
3677	LOC_Os02g52630.1	2
3677	LOC_Os04g37640.1	4
3677	LOC_Os04g37650.1	4
3677	LOC_Os06g10950.1	6
3677	LOC_Os06g10960.1	6
3677	LOC_Os06g10970.1	6
3677	LOC_Os06g10980.1	6
3677	LOC_Os06g10910.1	6
3677	LOC_Os06g10920.1	6
3677	LOC_Os06g10930.1	6
3677	LOC_Os08g24750.1	8
3677	LOC_Os09g28460.1	9
3678	LOC_Os01g14430.1	1
3678	LOC_Os01g72450.1	1
3678	LOC_Os02g25020.1	2
3678	LOC_Os02g48320.1	2
3678	LOC_Os02g57520.1	2
3678	LOC_Os03g16350.1	3
3678	LOC_Os03g01540.1	3
3678	LOC_Os04g50030.1	4
3678	LOC_Os06g04540.1	6
3678	LOC_Os06g22100.1	6
3678	LOC_Os06g41860.1	6
3678	LOC_Os07g13100.1	7
3678	LOC_Os08g06320.1	8
3678	LOC_Os08g44910.1	8
3678	LOC_Os09g28930.1	9
3678	LOC_Os11g05160.1	11
3678	LOC_Os12g05200.1	12
3679	LOC_Os01g15500.1	1
3679	LOC_Os01g52730.1	1
3679	LOC_Os01g52740.1	1
3679	LOC_Os01g54450.1	1
3679	LOC_Os01g64300.1	1
3679	LOC_Os02g41840.1	2
3679	LOC_Os03g02280.1	3
3679	LOC_Os04g33760.1	4

3679	LOC_Os04g43990.1	4
3679	LOC_Os04g45834.1	4
3679	LOC_Os05g44260.1	5
3679	LOC_Os05g45450.1	5
3679	LOC_Os07g32810.1	7
3679	LOC_Os07g33270.1	7
3679	LOC_Os10g33990.1	10
3679	LOC_Os11g05600.1	11
3679	LOC_Os12g05980.1	12
3680	LOC_Os01g02900.1	1
3680	LOC_Os01g02910.1	1
3680	LOC_Os01g31370.1	1
3680	LOC_Os01g72610.1	1
3680	LOC_Os02g04250.1	2
3680	LOC_Os02g22190.1	2
3680	LOC_Os02g22480.1	2
3680	LOC_Os02g22650.1	2
3680	LOC_Os03g37010.1	3
3680	LOC_Os04g12010.1	4
3680	LOC_Os05g32544.1	5
3680	LOC_Os06g20570.1	6
3680	LOC_Os06g28124.1	6
3680	LOC_Os06g49320.1	6
3680	LOC_Os07g46380.1	7
3680	LOC_Os10g35020.1	10
3680	LOC_Os12g13640.1	12
3681	LOC_Os01g23080.1	1
3681	LOC_Os02g33890.1	2
3681	LOC_Os04g05770.1	4
3681	LOC_Os04g06970.1	4
3681	LOC_Os05g31710.1	5
3681	LOC_Os06g02650.1	6
3681	LOC_Os07g01520.1	7
3681	LOC_Os07g12100.1	7
3681	LOC_Os07g16740.1	7
3681	LOC_Os07g17050.1	7
3681	LOC_Os08g11500.1	8
3681	LOC_Os10g34050.1	10
3681	LOC_Os10g34110.1	10
3681	LOC_Os11g07010.1	11
3681	LOC_Os11g40340.1	11
3681	LOC_Os11g43180.1	11
3681	LOC_Os12g08650.1	12
3682	LOC_Os01g08840.1	1
3682	LOC_Os01g13660.1	1
3682	LOC_Os01g40290.1	1
3682	LOC_Os01g51670.1	1
3682	LOC_Os02g12480.1	2
3682	LOC_Os02g12560.1	2
3682	LOC_Os05g08860.1	5
3682	LOC_Os05g08900.1	5
3682	LOC_Os06g05410.1	6

3682	LOC_Os06g05470.1	6
3682	LOC_Os06g05480.1	6
3682	LOC_Os06g05430.1	6
3682	LOC_Os06g05440.1	6
3682	LOC_Os06g05510.1	6
3682	LOC_Os06g38210.1	6
3682	LOC_Os06g05420.1	6
3682	LOC_Os11g37570.1	11
3683	LOC_Os02g16020.1	2
3683	LOC_Os05g31350.1	5
3683	LOC_Os05g32520.1	5
3683	LOC_Os06g04100.1	6
3683	LOC_Os06g09770.1	6
3683	LOC_Os06g21850.1	6
3683	LOC_Os07g05780.1	7
3683	LOC_Os07g11990.1	7
3683	LOC_Os08g18800.1	8
3683	LOC_Os08g18810.1	8
3683	LOC_Os08g18720.1	8
3683	LOC_Os08g18750.1	8
3683	LOC_Os09g24400.1	9
3683	LOC_Os10g19980.1	10
3683	LOC_Os10g21450.1	10
3683	LOC_Os10g27250.1	10
3683	LOC_Os10g40806.1	10
3684	LOC_Os01g02750.1	1
3684	LOC_Os01g12300.1	1
3684	LOC_Os02g09359.1	2
3684	LOC_Os02g58610.1	2
3684	LOC_Os03g03880.1	3
3684	LOC_Os03g12680.1	3
3684	LOC_Os03g16260.1	3
3684	LOC_Os05g03460.1	5
3684	LOC_Os05g41950.1	5
3684	LOC_Os06g30130.1	6
3684	LOC_Os06g34960.1	6
3684	LOC_Os06g47790.1	6
3684	LOC_Os07g35310.1	7
3684	LOC_Os09g20880.1	9
3684	LOC_Os10g01560.1	10
3684	LOC_Os10g18260.1	10
3684	LOC_Os10g38920.1	10
3684	LOC_Os11g34610.1	11
3685	LOC_Os01g40870.1	1
3685	LOC_Os01g40860.1	1
3685	LOC_Os02g07760.1	2
3685	LOC_Os02g43280.1	2
3685	LOC_Os02g43194.1	2
3685	LOC_Os02g49720.1	2
3685	LOC_Os04g39020.1	4
3685	LOC_Os04g45720.1	4
3685	LOC_Os05g45960.1	5

3685	LOC_Os06g15990.1	6
3685	LOC_Os06g39230.1	6
3685	LOC_Os07g09060.1	7
3685	LOC_Os07g48920.1	7
3685	LOC_Os08g32870.1	8
3685	LOC_Os08g34210.1	8
3685	LOC_Os09g26880.1	9
3685	LOC_Os11g08300.1	11
3685	LOC_Os12g07810.1	12
3686	LOC_Os01g49890.1	1
3686	LOC_Os01g59920.1	1
3686	LOC_Os01g74650.1	1
3686	LOC_Os02g12900.1	2
3686	LOC_Os03g11660.1	3
3686	LOC_Os03g53650.1	3
3686	LOC_Os04g08350.1	4
3686	LOC_Os04g46930.1	4
3686	LOC_Os05g47640.1	5
3686	LOC_Os06g05690.1	6
3686	LOC_Os06g05700.1	6
3686	LOC_Os06g36830.1	6
3686	LOC_Os06g36840.1	6
3686	LOC_Os06g36850.1	6
3686	LOC_Os06g36880.1	6
3686	LOC_Os06g42560.1	6
3686	LOC_Os08g04180.1	8
3686	LOC_Os12g42980.1	12
3687	LOC_Os01g24070.1	1
3687	LOC_Os01g54210.1	1
3687	LOC_Os01g74540.1	1
3687	LOC_Os02g12790.1	2
3687	LOC_Os02g43150.1	2
3687	LOC_Os02g56250.1	2
3687	LOC_Os03g03850.1	3
3687	LOC_Os03g05160.1	3
3687	LOC_Os04g46020.1	4
3687	LOC_Os05g06340.1	5
3687	LOC_Os05g44400.1	5
3687	LOC_Os05g50270.1	5
3687	LOC_Os06g37450.1	6
3687	LOC_Os10g32070.1	10
3687	LOC_Os10g40810.1	10
3687	LOC_Os11g08410.1	11
3687	LOC_Os12g07120.1	12
3687	LOC_Os12g42970.1	12
3688	LOC_Os01g13320.1	1
3688	LOC_Os01g15039.1	1
3688	LOC_Os01g20980.1	1
3688	LOC_Os01g57854.1	1
3688	LOC_Os02g18650.1	2
3688	LOC_Os02g54190.1	2
3688	LOC_Os03g18860.1	3

3688	LOC_Os03g28090.1	3
3688	LOC_Os04g38560.1	4
3688	LOC_Os04g54850.1	4
3688	LOC_Os05g29790.1	5
3688	LOC_Os06g09340.1	6
3688	LOC_Os07g47830.1	7
3688	LOC_Os07g49100.1	7
3688	LOC_Os08g34900.1	8
3688	LOC_Os08g34910.1	8
3688	LOC_Os09g26360.1	9
3688	LOC_Os11g07090.1	11
3689	LOC_Os01g18850.1	1
3689	LOC_Os01g69830.1	1
3689	LOC_Os02g04680.1	2
3689	LOC_Os02g07780.1	2
3689	LOC_Os02g08070.1	2
3689	LOC_Os03g61760.1	3
3689	LOC_Os04g46580.1	4
3689	LOC_Os04g56170.1	4
3689	LOC_Os05g33810.1	5
3689	LOC_Os06g44860.1	6
3689	LOC_Os06g45310.1	6
3689	LOC_Os06g49010.1	6
3689	LOC_Os07g32170.1	7
3689	LOC_Os08g39890.1	8
3689	LOC_Os08g41940.1	8
3689	LOC_Os09g31438.1	9
3689	LOC_Os09g32944.1	9
3689	LOC_Os11g30380.1	11
3690	LOC_Os01g22540.1	1
3690	LOC_Os01g72720.1	1
3690	LOC_Os03g56730.1	3
3690	LOC_Os03g57050.1	3
3690	LOC_Os04g47550.1	4
3690	LOC_Os05g05760.1	5
3690	LOC_Os05g12560.1	5
3690	LOC_Os06g05560.1	6
3690	LOC_Os06g35090.1	6
3690	LOC_Os07g08510.1	7
3690	LOC_Os07g08650.1	7
3690	LOC_Os07g39000.1	7
3690	LOC_Os09g36000.1	9
3690	LOC_Os10g36634.1	10
3690	LOC_Os11g12470.1	11
3690	LOC_Os12g05529.1	12
3690	LOC_Os12g40290.1	12
3690	LOC_Os12g41460.1	12
3691	LOC_Os01g50480.1	1
3691	LOC_Os01g50610.1	1
3691	LOC_Os02g48770.1	2
3691	LOC_Os06g13350.1	6
3691	LOC_Os06g13310.1	6

3691	LOC_Os06g13390.1	6
3691	LOC_Os06g13560.1	6
3691	LOC_Os06g13470.1	6
3691	LOC_Os06g20630.1	6
3691	LOC_Os06g20770.1	6
3691	LOC_Os06g20790.1	6
3691	LOC_Os06g20960.1	6
3691	LOC_Os06g22440.1	6
3691	LOC_Os06g20920.1	6
3691	LOC_Os11g15030.1	11
3691	LOC_Os11g15180.1	11
3691	LOC_Os11g15300.1	11
3691	LOC_Os11g15060.1	11
3692	LOC_Os01g37720.1	1
3692	LOC_Os01g46890.1	1
3692	LOC_Os02g37900.1	2
3692	LOC_Os02g44450.1	2
3692	LOC_Os03g04910.1	3
3692	LOC_Os03g07040.1	3
3692	LOC_Os04g14090.1	4
3692	LOC_Os04g18110.1	4
3692	LOC_Os04g18820.1	4
3692	LOC_Os04g20900.1	4
3692	LOC_Os07g35170.1	7
3692	LOC_Os07g37520.1	7
3692	LOC_Os07g37940.1	7
3692	LOC_Os08g22550.1	8
3692	LOC_Os09g11710.1	9
3692	LOC_Os10g11160.1	10
3692	LOC_Os10g17110.1	10
3692	LOC_Os11g24220.1	11
3693	LOC_Os01g56450.1	1
3693	LOC_Os01g60170.1	1
3693	LOC_Os01g70600.1	1
3693	LOC_Os01g70590.1	1
3693	LOC_Os03g08200.1	3
3693	LOC_Os03g22410.1	3
3693	LOC_Os03g60210.1	3
3693	LOC_Os03g60220.1	3
3693	LOC_Os04g36830.1	4
3693	LOC_Os05g40630.1	5
3693	LOC_Os05g24650.1	5
3693	LOC_Os05g43460.1	5
3693	LOC_Os07g12730.1	7
3693	LOC_Os07g47540.1	7
3693	LOC_Os11g02800.1	11
3693	LOC_Os11g02750.1	11
3693	LOC_Os12g02720.1	12
3693	LOC_Os12g02760.1	12
3694	LOC_Os01g06460.1	1
3694	LOC_Os01g16210.1	1
3694	LOC_Os02g18540.1	2

3694	LOC_Os02g36940.1	2
3694	LOC_Os02g52550.1	2
3694	LOC_Os02g36950.1	2
3694	LOC_Os03g03180.1	3
3694	LOC_Os03g61430.1	3
3694	LOC_Os03g61440.1	3
3694	LOC_Os03g61470.1	3
3694	LOC_Os03g61490.1	3
3694	LOC_Os03g61500.1	3
3694	LOC_Os03g01210.1	3
3694	LOC_Os06g15590.1	6
3694	LOC_Os06g32910.1	6
3694	LOC_Os07g36820.1	7
3694	LOC_Os10g02300.1	10
3694	LOC_Os10g39100.1	10
3695	LOC_Os01g26904.1	1
3695	LOC_Os01g27070.1	1
3695	LOC_Os01g27120.1	1
3695	LOC_Os01g28080.1	1
3695	LOC_Os01g28089.1	1
3695	LOC_Os01g28109.1	1
3695	LOC_Os01g29330.1	1
3695	LOC_Os01g29240.1	1
3695	LOC_Os01g29280.1	1
3695	LOC_Os01g65992.1	1
3695	LOC_Os02g27800.1	2
3695	LOC_Os03g25440.1	3
3695	LOC_Os05g48840.1	5
3695	LOC_Os06g24490.1	6
3695	LOC_Os07g22510.1	7
3695	LOC_Os07g45080.1	7
3695	LOC_Os08g01530.1	8
3695	LOC_Os12g22270.1	12
3696	LOC_Os02g49010.1	2
3696	LOC_Os05g15260.1	5
3696	LOC_Os05g22450.1	5
3696	LOC_Os05g24530.1	5
3696	LOC_Os06g26320.1	6
3696	LOC_Os07g23030.1	7
3696	LOC_Os07g24760.1	7
3696	LOC_Os07g27220.1	7
3696	LOC_Os07g30850.1	7
3696	LOC_Os08g04070.1	8
3696	LOC_Os08g05210.1	8
3696	LOC_Os08g10980.1	8
3696	LOC_Os08g11030.1	8
3696	LOC_Os08g19880.1	8
3696	LOC_Os08g26740.1	8
3696	LOC_Os10g09390.1	10
3696	LOC_Os11g18260.1	11
3696	LOC_Os12g15050.1	12
3697	LOC_Os01g17240.1	1

3697	LOC_Os01g36720.1	1
3697	LOC_Os01g50820.1	1
3697	LOC_Os01g63290.1	1
3697	LOC_Os02g02170.1	2
3697	LOC_Os02g02190.1	2
3697	LOC_Os02g43620.1	2
3697	LOC_Os02g58080.1	2
3697	LOC_Os03g59450.1	3
3697	LOC_Os04g44430.1	4
3697	LOC_Os04g46880.1	4
3697	LOC_Os06g08170.1	6
3697	LOC_Os07g10820.1	7
3697	LOC_Os08g06010.1	8
3697	LOC_Os09g20520.1	9
3697	LOC_Os09g38410.1	9
3697	LOC_Os11g08370.1	11
3697	LOC_Os12g07970.1	12
3698	LOC_Os01g31000.1	1
3698	LOC_Os01g35620.1	1
3698	LOC_Os02g24060.1	2
3698	LOC_Os04g06540.1	4
3698	LOC_Os04g19020.1	4
3698	LOC_Os04g21450.1	4
3698	LOC_Os04g28750.1	4
3698	LOC_Os06g28580.1	6
3698	LOC_Os07g17830.1	7
3698	LOC_Os07g25400.1	7
3698	LOC_Os08g21820.1	8
3698	LOC_Os08g22020.1	8
3698	LOC_Os09g01006.1	9
3698	LOC_Os09g06340.1	9
3698	LOC_Os10g15190.1	10
3698	LOC_Os10g23870.1	10
3698	LOC_Os12g19450.1	12
3698	LOC_Os12g34410.1	12
3699	LOC_Os01g31100.1	1
3699	LOC_Os01g32060.1	1
3699	LOC_Os01g32480.1	1
3699	LOC_Os02g21800.1	2
3699	LOC_Os02g23830.1	2
3699	LOC_Os02g25200.1	2
3699	LOC_Os02g25220.1	2
3699	LOC_Os04g14500.1	4
3699	LOC_Os04g15970.1	4
3699	LOC_Os04g16314.1	4
3699	LOC_Os05g23640.1	5
3699	LOC_Os07g20400.1	7
3699	LOC_Os08g10810.1	8
3699	LOC_Os08g14510.1	8
3699	LOC_Os08g21470.1	8
3699	LOC_Os10g24360.1	10
3699	LOC_Os12g08950.1	12

3699	LOC_Os12g20120.1	12
3700	LOC_Os01g31380.1	1
3700	LOC_Os02g22690.1	2
3700	LOC_Os02g24910.1	2
3700	LOC_Os03g33000.1	3
3700	LOC_Os03g33310.1	3
3700	LOC_Os04g06320.1	4
3700	LOC_Os04g07960.1	4
3700	LOC_Os04g16620.1	4
3700	LOC_Os04g20740.1	4
3700	LOC_Os04g26640.1	4
3700	LOC_Os05g20080.1	5
3700	LOC_Os09g19480.1	9
3700	LOC_Os10g08240.1	10
3700	LOC_Os10g16580.1	10
3700	LOC_Os10g18930.1	10
3700	LOC_Os11g22880.1	11
3700	LOC_Os11g32190.1	11
3700	LOC_Os12g19960.1	12
3701	LOC_Os01g25100.1	1
3701	LOC_Os01g27260.1	1
3701	LOC_Os01g27380.1	1
3701	LOC_Os01g27390.1	1
3701	LOC_Os01g27210.1	1
3701	LOC_Os01g27340.1	1
3701	LOC_Os01g27360.1	1
3701	LOC_Os01g27480.1	1
3701	LOC_Os01g27630.1	1
3701	LOC_Os01g55830.1	1
3701	LOC_Os01g70770.1	1
3701	LOC_Os02g35590.1	2
3701	LOC_Os03g04240.1	3
3701	LOC_Os03g04250.1	3
3701	LOC_Os04g35560.1	4
3701	LOC_Os05g05620.1	5
3701	LOC_Os10g38189.1	10
3701	LOC_Os11g14040.1	11
3701	LOC_Os12g10720.1	12
3702	LOC_Os01g72330.1	1
3702	LOC_Os02g35180.1	2
3702	LOC_Os02g42060.1	2
3702	LOC_Os02g58350.1	2
3702	LOC_Os03g53100.1	3
3702	LOC_Os04g13480.1	4
3702	LOC_Os04g28160.1	4
3702	LOC_Os04g28120.1	4
3702	LOC_Os04g36070.1	4
3702	LOC_Os04g44280.1	4
3702	LOC_Os04g57720.1	4
3702	LOC_Os05g32880.1	5
3702	LOC_Os05g32890.1	5
3702	LOC_Os07g26720.1	7

3702	LOC_Os08g26990.1	8
3702	LOC_Os08g28900.1	8
3702	LOC_Os08g28950.1	8
3702	LOC_Os11g04720.1	11
3702	LOC_Os12g04500.1	12
3703	LOC_Os02g01280.1	2
3703	LOC_Os02g14929.1	2
3703	LOC_Os02g22780.1	2
3703	LOC_Os03g04970.1	3
3703	LOC_Os03g42220.1	3
3703	LOC_Os03g59020.1	3
3703	LOC_Os03g64210.1	3
3703	LOC_Os04g46620.1	4
3703	LOC_Os05g05470.1	5
3703	LOC_Os05g46290.1	5
3703	LOC_Os05g48290.1	5
3703	LOC_Os06g02380.1	6
3703	LOC_Os06g34690.1	6
3703	LOC_Os06g36700.1	6
3703	LOC_Os06g47320.1	6
3703	LOC_Os09g38980.1	9
3703	LOC_Os10g32550.1	10
3703	LOC_Os10g37060.1	10
3703	LOC_Os12g17910.1	12
3704	LOC_Os01g02120.1	1
3704	LOC_Os01g10590.1	1
3704	LOC_Os01g11940.1	1
3704	LOC_Os01g54490.1	1
3704	LOC_Os02g13830.1	2
3704	LOC_Os02g32950.1	2
3704	LOC_Os04g33570.1	4
3704	LOC_Os04g41130.1	4
3704	LOC_Os05g39250.1	5
3704	LOC_Os05g44180.1	5
3704	LOC_Os06g06300.1	6
3704	LOC_Os06g06320.1	6
3704	LOC_Os06g30370.1	6
3704	LOC_Os06g35940.1	6
3704	LOC_Os09g33850.1	9
3704	LOC_Os11g05470.1	11
3704	LOC_Os11g18870.1	11
3704	LOC_Os12g05590.1	12
3704	LOC_Os12g13030.1	12
3705	LOC_Os01g34560.1	1
3705	LOC_Os02g11070.1	2
3705	LOC_Os02g49920.1	2
3705	LOC_Os02g56860.1	2
3705	LOC_Os03g06700.1	3
3705	LOC_Os03g12030.1	3
3705	LOC_Os03g14170.1	3
3705	LOC_Os03g26620.1	3
3705	LOC_Os03g26530.1	3

3705	LOC_Os05g49290.1	5
3705	LOC_Os05g49900.1	5
3705	LOC_Os06g14810.1	6
3705	LOC_Os06g15020.1	6
3705	LOC_Os06g15250.1	6
3705	LOC_Os06g15170.1	6
3705	LOC_Os06g39750.1	6
3705	LOC_Os10g07010.1	10
3705	LOC_Os10g28060.1	10
3705	LOC_Os11g37900.1	11
3706	LOC_Os02g34690.1	2
3706	LOC_Os02g49050.1	2
3706	LOC_Os02g55440.1	2
3706	LOC_Os03g13380.1	3
3706	LOC_Os05g07650.1	5
3706	LOC_Os06g37160.1	6
3706	LOC_Os06g44140.1	6
3706	LOC_Os08g17370.1	8
3706	LOC_Os08g19160.1	8
3706	LOC_Os08g38820.1	8
3706	LOC_Os08g44140.1	8
3706	LOC_Os08g44150.1	8
3706	LOC_Os08g44090.1	8
3706	LOC_Os08g44100.1	8
3706	LOC_Os08g06470.1	8
3706	LOC_Os09g38530.1	9
3706	LOC_Os10g02350.1	10
3706	LOC_Os11g07910.1	11
3706	LOC_Os12g07670.1	12
3707	LOC_Os01g50330.1	1
3707	LOC_Os03g15710.1	3
3707	LOC_Os03g53950.1	3
3707	LOC_Os06g41820.1	6
3707	LOC_Os06g41830.1	6
3707	LOC_Os06g41850.1	6
3707	LOC_Os07g35990.1	7
3707	LOC_Os07g36040.1	7
3707	LOC_Os07g36060.1	7
3707	LOC_Os07g35970.1	7
3707	LOC_Os07g42250.1	7
3707	LOC_Os08g07810.1	8
3707	LOC_Os08g34330.1	8
3707	LOC_Os09g20810.1	9
3707	LOC_Os09g20684.1	9
3707	LOC_Os09g20700.1	9
3707	LOC_Os10g39710.1	10
3707	LOC_Os11g04660.1	11
3707	LOC_Os12g04424.1	12
3708	LOC_Os01g45550.1	1
3708	LOC_Os01g51780.1	1
3708	LOC_Os01g58860.1	1
3708	LOC_Os01g60230.1	1

3708	LOC_Os01g69070.1	1
3708	LOC_Os02g50960.1	2
3708	LOC_Os05g40330.1	5
3708	LOC_Os05g50140.1	5
3708	LOC_Os06g12610.1	6
3708	LOC_Os06g44970.1	6
3708	LOC_Os08g09190.1	8
3708	LOC_Os08g41720.1	8
3708	LOC_Os09g32770.1	9
3708	LOC_Os09g38130.1	9
3708	LOC_Os09g38210.1	9
3708	LOC_Os09g31478.1	9
3708	LOC_Os11g02950.1	11
3708	LOC_Os11g04190.1	11
3708	LOC_Os12g04000.1	12
3709	LOC_Os01g11550.1	1
3709	LOC_Os01g55750.1	1
3709	LOC_Os01g69980.1	1
3709	LOC_Os02g42380.1	2
3709	LOC_Os02g51310.1	2
3709	LOC_Os03g49880.1	3
3709	LOC_Os03g57190.1	3
3709	LOC_Os04g11830.1	4
3709	LOC_Os04g44440.1	4
3709	LOC_Os05g43760.1	5
3709	LOC_Os07g04510.1	7
3709	LOC_Os07g05720.1	7
3709	LOC_Os08g33530.1	8
3709	LOC_Os08g43160.1	8
3709	LOC_Os09g24480.1	9
3709	LOC_Os09g34950.1	9
3709	LOC_Os11g07460.1	11
3709	LOC_Os12g02090.1	12
3709	LOC_Os12g07480.1	12
3710	LOC_Os01g19180.1	1
3710	LOC_Os01g28660.1	1
3710	LOC_Os01g28920.1	1
3710	LOC_Os01g47500.1	1
3710	LOC_Os02g37170.1	2
3710	LOC_Os03g16270.1	3
3710	LOC_Os03g31634.1	3
3710	LOC_Os03g62360.1	3
3710	LOC_Os04g58120.1	4
3710	LOC_Os05g24830.1	5
3710	LOC_Os05g41820.1	5
3710	LOC_Os06g10460.1	6
3710	LOC_Os06g23620.1	6
3710	LOC_Os07g29560.1	7
3710	LOC_Os09g15720.1	9
3710	LOC_Os10g36510.1	10
3710	LOC_Os11g33440.1	11
3710	LOC_Os12g10530.1	12

3710	LOC_Os12g42284.1	12
3711	LOC_Os01g08800.1	1
3711	LOC_Os01g63930.1	1
3711	LOC_Os01g72270.1	1
3711	LOC_Os02g38290.1	2
3711	LOC_Os02g44654.1	2
3711	LOC_Os03g04630.1	3
3711	LOC_Os03g04640.1	3
3711	LOC_Os03g04650.1	3
3711	LOC_Os03g12260.1	3
3711	LOC_Os04g47250.1	4
3711	LOC_Os05g37250.1	5
3711	LOC_Os06g03930.1	6
3711	LOC_Os08g16260.1	8
3711	LOC_Os08g16320.1	8
3711	LOC_Os10g34480.1	10
3711	LOC_Os11g04290.1	11
3711	LOC_Os11g04310.1	11
3711	LOC_Os11g05380.1	11
3711	LOC_Os12g04100.1	12
3711	LOC_Os12g05440.1	12
3712	LOC_Os01g02080.1	1
3712	LOC_Os01g18210.1	1
3712	LOC_Os01g40050.1	1
3712	LOC_Os02g02090.1	2
3712	LOC_Os02g02890.1	2
3712	LOC_Os02g10970.1	2
3712	LOC_Os03g59700.1	3
3712	LOC_Os03g10400.1	3
3712	LOC_Os05g01270.1	5
3712	LOC_Os06g04000.1	6
3712	LOC_Os06g49470.1	6
3712	LOC_Os06g49480.1	6
3712	LOC_Os07g29390.1	7
3712	LOC_Os08g19610.1	8
3712	LOC_Os08g29370.1	8
3712	LOC_Os08g44520.1	8
3712	LOC_Os09g36670.1	9
3712	LOC_Os09g39780.1	9
3712	LOC_Os10g06630.1	10
3712	LOC_Os11g38990.1	11
3713	LOC_Os01g38359.1	1
3713	LOC_Os01g62610.1	1
3713	LOC_Os01g68710.1	1
3713	LOC_Os02g02550.1	2
3713	LOC_Os02g07220.1	2
3713	LOC_Os02g10590.1	2
3713	LOC_Os02g51570.1	2
3713	LOC_Os02g52290.1	2
3713	LOC_Os03g50080.1	3
3713	LOC_Os04g36890.1	4
3713	LOC_Os05g38370.1	5

3713	LOC_Os06g20320.1	6
3713	LOC_Os06g45340.1	6
3713	LOC_Os07g04160.1	7
3713	LOC_Os07g09040.1	7
3713	LOC_Os08g42850.1	8
3713	LOC_Os09g01650.1	9
3713	LOC_Os09g01670.1	9
3713	LOC_Os09g12270.1	9
3713	LOC_Os09g32526.1	9
3714	LOC_Os01g23760.1	1
3714	LOC_Os01g23770.1	1
3714	LOC_Os01g67890.1	1
3714	LOC_Os01g68560.1	1
3714	LOC_Os01g69850.1	1
3714	LOC_Os03g03100.1	3
3714	LOC_Os03g14850.1	3
3714	LOC_Os04g31804.1	4
3714	LOC_Os04g38770.1	4
3714	LOC_Os05g11380.1	5
3714	LOC_Os05g23780.1	5
3714	LOC_Os06g11970.1	6
3714	LOC_Os06g22760.1	6
3714	LOC_Os06g23950.1	6
3714	LOC_Os07g04170.1	7
3714	LOC_Os08g20460.1	8
3714	LOC_Os08g38590.1	8
3714	LOC_Os08g41960.1	8
3714	LOC_Os11g43740.1	11
3714	LOC_Os12g21880.1	12
3715	LOC_Os01g11160.1	1
3715	LOC_Os01g19850.1	1
3715	LOC_Os01g42234.1	1
3715	LOC_Os01g71700.1	1
3715	LOC_Os01g71710.1	1
3715	LOC_Os01g71720.1	1
3715	LOC_Os01g71740.1	1
3715	LOC_Os01g71760.1	1
3715	LOC_Os02g43860.1	2
3715	LOC_Os03g43970.1	3
3715	LOC_Os03g45170.1	3
3715	LOC_Os04g35540.1	4
3715	LOC_Os04g45950.1	4
3715	LOC_Os06g34830.1	6
3715	LOC_Os08g23440.1	8
3715	LOC_Os10g30090.1	10
3715	LOC_Os11g05690.1	11
3715	LOC_Os12g06060.1	12
3715	LOC_Os12g41890.1	12
3715	LOC_Os12g42850.1	12
3716	LOC_Os01g73750.1	1
3716	LOC_Os02g13640.1	2
3716	LOC_Os02g13990.1	2

3716	LOC_Os02g20190.1	2
3716	LOC_Os02g20460.1	2
3716	LOC_Os02g39970.1	2
3716	LOC_Os02g46770.1	2
3716	LOC_Os02g54170.1	2
3716	LOC_Os02g44104.1	2
3716	LOC_Os03g12160.1	3
3716	LOC_Os03g63220.1	3
3716	LOC_Os03g08140.1	3
3716	LOC_Os04g32940.1	4
3716	LOC_Os04g53000.1	4
3716	LOC_Os04g42470.1	4
3716	LOC_Os05g46560.1	5
3716	LOC_Os06g46380.1	6
3716	LOC_Os08g35700.1	8
3716	LOC_Os10g05570.1	10
3716	LOC_Os12g12740.1	12
3717	LOC_Os01g51840.1	1
3717	LOC_Os02g01520.1	2
3717	LOC_Os02g19640.1	2
3717	LOC_Os03g04210.1	3
3717	LOC_Os03g06570.1	3
3717	LOC_Os03g23960.1	3
3717	LOC_Os03g43580.1	3
3717	LOC_Os04g48160.1	4
3717	LOC_Os04g56740.1	4
3717	LOC_Os05g43670.1	5
3717	LOC_Os05g44630.1	5
3717	LOC_Os05g45930.1	5
3717	LOC_Os06g01500.1	6
3717	LOC_Os06g08850.1	6
3717	LOC_Os06g23380.1	6
3717	LOC_Os06g23390.1	6
3717	LOC_Os06g40850.1	6
3717	LOC_Os10g19342.1	10
3717	LOC_Os10g28420.1	10
3717	LOC_Os12g41160.1	12
3718	LOC_Os01g21070.1	1
3718	LOC_Os02g03120.1	2
3718	LOC_Os02g05744.1	2
3718	LOC_Os02g50040.1	2
3718	LOC_Os02g50490.1	2
3718	LOC_Os02g53820.1	2
3718	LOC_Os03g21210.1	3
3718	LOC_Os03g52630.1	3
3718	LOC_Os04g36610.1	4
3718	LOC_Os04g41970.1	4
3718	LOC_Os05g03840.1	5
3718	LOC_Os06g13830.1	6
3718	LOC_Os06g14540.1	6
3718	LOC_Os06g50140.1	6
3718	LOC_Os08g02220.1	8

3718	LOC_Os08g29770.1	8
3718	LOC_Os08g32940.1	8
3718	LOC_Os09g23084.1	9
3718	LOC_Os09g36060.1	9
3718	LOC_Os09g36350.1	9
3719	LOC_Os01g24790.1	1
3719	LOC_Os03g45740.1	3
3719	LOC_Os04g11810.1	4
3719	LOC_Os04g42830.1	4
3719	LOC_Os05g04930.1	5
3719	LOC_Os05g06380.1	5
3719	LOC_Os05g22300.1	5
3719	LOC_Os05g25150.1	5
3719	LOC_Os06g08610.1	6
3719	LOC_Os06g49660.1	6
3719	LOC_Os07g14080.1	7
3719	LOC_Os07g23960.1	7
3719	LOC_Os07g36560.1	7
3719	LOC_Os09g25460.1	9
3719	LOC_Os10g01720.1	10
3719	LOC_Os10g01660.1	10
3719	LOC_Os10g01690.1	10
3719	LOC_Os10g04429.1	10
3719	LOC_Os10g11980.1	10
3719	LOC_Os10g35950.1	10
3720	LOC_Os01g24870.1	1
3720	LOC_Os03g15170.1	3
3720	LOC_Os03g32890.1	3
3720	LOC_Os03g33810.1	3
3720	LOC_Os03g37230.1	3
3720	LOC_Os04g03480.1	4
3720	LOC_Os04g48550.1	4
3720	LOC_Os06g09040.1	6
3720	LOC_Os06g17380.1	6
3720	LOC_Os06g25690.1	6
3720	LOC_Os06g27710.1	6
3720	LOC_Os06g32400.1	6
3720	LOC_Os07g15480.1	7
3720	LOC_Os07g20520.1	7
3720	LOC_Os08g36510.1	8
3720	LOC_Os09g14460.1	9
3720	LOC_Os10g05360.1	10
3720	LOC_Os10g10290.1	10
3720	LOC_Os11g09910.1	11
3720	LOC_Os11g31670.1	11
3721	LOC_Os01g13440.1	1
3721	LOC_Os09g23908.1	9
3721	LOC_Os09g23939.1	9
3721	LOC_Os09g23948.1	9
3721	LOC_Os09g23957.1	9
3721	LOC_Os09g24020.1	9
3721	LOC_Os09g24029.1	9

3721	LOC_Os09g23899.1	9
3721	LOC_Os09g23966.1	9
3721	LOC_Os09g23999.1	9
3721	LOC_Os09g24008.1	9
3721	LOC_Os09g24100.1	9
3721	LOC_Os09g24110.1	9
3721	LOC_Os09g24120.1	9
3721	LOC_Os09g24130.1	9
3721	LOC_Os09g24140.1	9
3721	LOC_Os09g24038.1	9
3721	LOC_Os09g24159.1	9
3721	LOC_Os09g27710.1	9
3721	LOC_Os09g27720.1	9
3722	LOC_Os05g26350.1	5
3722	LOC_Os05g26359.1	5
3722	LOC_Os05g26368.1	5
3722	LOC_Os05g26440.1	5
3722	LOC_Os05g26460.1	5
3722	LOC_Os05g26480.1	5
3722	LOC_Os05g26620.1	5
3722	LOC_Os05g26750.1	5
3722	LOC_Os05g26770.1	5
3722	LOC_Os05g26240.1	5
3722	LOC_Os05g26377.1	5
3722	LOC_Os05g26386.1	5
3722	LOC_Os05g26400.1	5
3722	LOC_Os05g26490.1	5
3722	LOC_Os05g26690.1	5
3722	LOC_Os05g26720.1	5
3722	LOC_Os07g11950.1	7
3722	LOC_Os07g11900.1	7
3722	LOC_Os07g11910.1	7
3722	LOC_Os07g11920.1	7
3723	LOC_Os01g02110.1	1
3723	LOC_Os01g09900.1	1
3723	LOC_Os01g09990.1	1
3723	LOC_Os01g38610.1	1
3723	LOC_Os02g49480.1	2
3723	LOC_Os02g56140.1	2
3723	LOC_Os03g10770.1	3
3723	LOC_Os03g17130.1	3
3723	LOC_Os03g42100.1	3
3723	LOC_Os03g43810.1	3
3723	LOC_Os03g51580.1	3
3723	LOC_Os03g51910.1	3
3723	LOC_Os03g55550.1	3
3723	LOC_Os05g04740.1	5
3723	LOC_Os06g06900.1	6
3723	LOC_Os06g10820.1	6
3723	LOC_Os06g30090.1	6
3723	LOC_Os07g39940.1	7
3723	LOC_Os12g32400.1	12

3723	LOC_Os12g39850.1	12
3723	LOC_Os12g40730.1	12
3724	LOC_Os01g16030.1	1
3724	LOC_Os01g15010.1	1
3724	LOC_Os01g23620.1	1
3724	LOC_Os01g59790.1	1
3724	LOC_Os02g03610.1	2
3724	LOC_Os02g22140.1	2
3724	LOC_Os02g49980.1	2
3724	LOC_Os02g47110.1	2
3724	LOC_Os03g10370.1	3
3724	LOC_Os03g27450.1	3
3724	LOC_Os03g59740.1	3
3724	LOC_Os05g41060.1	5
3724	LOC_Os06g02390.1	6
3724	LOC_Os06g12090.1	6
3724	LOC_Os07g12200.1	7
3724	LOC_Os07g42820.1	7
3724	LOC_Os07g12170.1	7
3724	LOC_Os08g15040.1	8
3724	LOC_Os10g42940.1	10
3724	LOC_Os11g37640.1	11
3724	LOC_Os12g37360.1	12
3725	LOC_Os01g59120.1	1
3725	LOC_Os01g17402.1	1
3725	LOC_Os03g11030.1	3
3725	LOC_Os03g11040.1	3
3725	LOC_Os03g12414.1	3
3725	LOC_Os03g27420.1	3
3725	LOC_Os03g41100.1	3
3725	LOC_Os03g42070.1	3
3725	LOC_Os04g47580.1	4
3725	LOC_Os05g41390.1	5
3725	LOC_Os06g12980.1	6
3725	LOC_Os06g51110.1	6
3725	LOC_Os07g37010.1	7
3725	LOC_Os07g42860.1	7
3725	LOC_Os08g32540.1	8
3725	LOC_Os08g37390.1	8
3725	LOC_Os09g29100.1	9
3725	LOC_Os11g47950.1	11
3725	LOC_Os12g31810.1	12
3725	LOC_Os12g39210.1	12
3725	LOC_Os12g39830.1	12
3726	LOC_Os01g39020.1	1
3726	LOC_Os01g43590.1	1
3726	LOC_Os01g53220.1	1
3726	LOC_Os01g54550.1	1
3726	LOC_Os02g13800.1	2
3726	LOC_Os02g29340.1	2
3726	LOC_Os02g32590.1	2
3726	LOC_Os03g12370.1	3

3726	LOC_Os03g25120.1	3
3726	LOC_Os03g53340.1	3
3726	LOC_Os03g63750.1	3
3726	LOC_Os04g48030.1	4
3726	LOC_Os05g45410.1	5
3726	LOC_Os06g35960.1	6
3726	LOC_Os06g36930.1	6
3726	LOC_Os07g44690.1	7
3726	LOC_Os08g36700.1	8
3726	LOC_Os08g43334.1	8
3726	LOC_Os09g28200.1	9
3726	LOC_Os09g28354.1	9
3726	LOC_Os09g35790.1	9
3727	LOC_Os01g20950.1	1
3727	LOC_Os02g45890.1	2
3727	LOC_Os04g44460.1	4
3727	LOC_Os06g42120.1	6
3727	LOC_Os07g05450.1	7
3727	LOC_Os07g41540.1	7
3727	LOC_Os08g17510.1	8
3727	LOC_Os08g40330.1	8
3727	LOC_Os08g40380.1	8
3727	LOC_Os08g40390.1	8
3727	LOC_Os09g08190.1	9
3727	LOC_Os09g38239.1	9
3727	LOC_Os11g04530.1	11
3727	LOC_Os11g04540.1	11
3727	LOC_Os11g04550.1	11
3727	LOC_Os11g26390.1	11
3727	LOC_Os11g30810.1	11
3727	LOC_Os12g04320.1	12
3727	LOC_Os12g04300.1	12
3727	LOC_Os12g04340.1	12
3727	LOC_Os12g04350.1	12
3728	LOC_Os01g15310.1	1
3728	LOC_Os01g64690.1	1
3728	LOC_Os01g73604.1	1
3728	LOC_Os02g01250.1	2
3728	LOC_Os02g03440.1	2
3728	LOC_Os02g08090.1	2
3728	LOC_Os02g37430.1	2
3728	LOC_Os02g30624.1	2
3728	LOC_Os03g13760.1	3
3728	LOC_Os03g29740.1	3
3728	LOC_Os04g31950.1	4
3728	LOC_Os04g36810.1	4
3728	LOC_Os05g24970.1	5
3728	LOC_Os05g32310.1	5
3728	LOC_Os05g51650.1	5
3728	LOC_Os07g07220.1	7
3728	LOC_Os07g41790.1	7
3728	LOC_Os08g05530.1	8

3728	LOC_Os08g05850.1	8
3728	LOC_Os08g08040.1	8
3728	LOC_Os11g43620.1	11
3729	LOC_Os01g43320.1	1
3729	LOC_Os01g63770.1	1
3729	LOC_Os01g63854.1	1
3729	LOC_Os01g68050.1	1
3729	LOC_Os01g61044.1	1
3729	LOC_Os02g44980.1	2
3729	LOC_Os03g14080.1	3
3729	LOC_Os03g44230.1	3
3729	LOC_Os04g38660.1	4
3729	LOC_Os04g47420.1	4
3729	LOC_Os04g47780.1	4
3729	LOC_Os05g14820.1	5
3729	LOC_Os05g50920.1	5
3729	LOC_Os05g37470.1	5
3729	LOC_Os06g43700.1	6
3729	LOC_Os07g01090.1	7
3729	LOC_Os07g12770.1	7
3729	LOC_Os10g05690.1	10
3729	LOC_Os10g27980.1	10
3729	LOC_Os11g06820.1	11
3729	LOC_Os12g30040.1	12
3730	LOC_Os01g65660.1	1
3730	LOC_Os01g65670.1	1
3730	LOC_Os01g66010.1	1
3730	LOC_Os02g01210.1	2
3730	LOC_Os02g49060.1	2
3730	LOC_Os04g39489.1	4
3730	LOC_Os04g41350.1	4
3730	LOC_Os04g56470.1	4
3730	LOC_Os05g34980.1	5
3730	LOC_Os06g12330.1	6
3730	LOC_Os06g12350.1	6
3730	LOC_Os06g36210.1	6
3730	LOC_Os06g36180.1	6
3730	LOC_Os07g04180.1	7
3730	LOC_Os08g03350.1	8
3730	LOC_Os11g09020.1	11
3730	LOC_Os12g08090.1	12
3730	LOC_Os12g08130.1	12
3730	LOC_Os12g09300.1	12
3730	LOC_Os12g09320.1	12
3730	LOC_Os12g14100.1	12
3731	LOC_Os01g08320.1	1
3731	LOC_Os01g09450.1	1
3731	LOC_Os01g13030.1	1
3731	LOC_Os01g18360.1	1
3731	LOC_Os01g48450.1	1
3731	LOC_Os01g53880.1	1
3731	LOC_Os02g13520.1	2

3731	LOC_Os02g56120.1	2
3731	LOC_Os03g43410.1	3
3731	LOC_Os03g58350.1	3
3731	LOC_Os05g14180.1	5
3731	LOC_Os05g44810.1	5
3731	LOC_Os05g48590.1	5
3731	LOC_Os05g08570.1	5
3731	LOC_Os06g07040.1	6
3731	LOC_Os06g22870.1	6
3731	LOC_Os06g24850.1	6
3731	LOC_Os06g39590.1	6
3731	LOC_Os07g08460.1	7
3731	LOC_Os11g11410.1	11
3731	LOC_Os12g40890.1	12
3732	LOC_Os08g43750.1	8
3732	LOC_Os08g43860.1	8
3732	LOC_Os08g43870.1	8
3732	LOC_Os08g43890.1	8
3732	LOC_Os08g43900.1	8
3732	LOC_Os08g43910.1	8
3732	LOC_Os08g43920.1	8
3732	LOC_Os08g43930.1	8
3732	LOC_Os08g43760.1	8
3732	LOC_Os08g43770.1	8
3732	LOC_Os08g43790.1	8
3732	LOC_Os08g43800.1	8
3732	LOC_Os08g43810.1	8
3732	LOC_Os08g43820.1	8
3732	LOC_Os08g43830.1	8
3732	LOC_Os08g43960.1	8
3732	LOC_Os08g43970.1	8
3732	LOC_Os08g43940.1	8
3732	LOC_Os08g43980.1	8
3732	LOC_Os11g06100.1	11
3732	LOC_Os11g06110.1	11
3733	LOC_Os01g14440.1	1
3733	LOC_Os01g18584.1	1
3733	LOC_Os01g40430.1	1
3733	LOC_Os01g43550.1	1
3733	LOC_Os01g47560.1	1
3733	LOC_Os01g53040.1	1
3733	LOC_Os01g53260.1	1
3733	LOC_Os01g74140.1	1
3733	LOC_Os01g08710.1	1
3733	LOC_Os02g53100.1	2
3733	LOC_Os03g55164.1	3
3733	LOC_Os04g46060.1	4
3733	LOC_Os05g04640.1	5
3733	LOC_Os05g25770.1	5
3733	LOC_Os05g49100.1	5
3733	LOC_Os06g06360.1	6
3733	LOC_Os06g44010.1	6

3733	LOC_Os07g02060.1	7
3733	LOC_Os07g48260.1	7
3733	LOC_Os09g25070.1	9
3733	LOC_Os11g29870.1	11
3734	LOC_Os01g07410.1	1
3734	LOC_Os01g62390.1	1
3734	LOC_Os01g63230.1	1
3734	LOC_Os02g06400.1	2
3734	LOC_Os03g07310.1	3
3734	LOC_Os04g47520.1	4
3734	LOC_Os04g46570.1	4
3734	LOC_Os05g04190.1	5
3734	LOC_Os05g38490.1	5
3734	LOC_Os05g37880.1	5
3734	LOC_Os06g11600.1	6
3734	LOC_Os06g13220.1	6
3734	LOC_Os06g35410.1	6
3734	LOC_Os06g47290.1	6
3734	LOC_Os09g24570.1	9
3734	LOC_Os09g27080.1	9
3734	LOC_Os11g07510.1	11
3734	LOC_Os11g29120.1	11
3734	LOC_Os12g07540.1	12
3734	LOC_Os12g08820.1	12
3734	LOC_Os12g23760.1	12
3735	LOC_Os01g61250.1	1
3735	LOC_Os01g62800.1	1
3735	LOC_Os01g66110.1	1
3735	LOC_Os01g67360.1	1
3735	LOC_Os02g45310.1	2
3735	LOC_Os02g51860.1	2
3735	LOC_Os03g56380.1	3
3735	LOC_Os04g14150.1	4
3735	LOC_Os04g48140.1	4
3735	LOC_Os04g48230.1	4
3735	LOC_Os04g59590.1	4
3735	LOC_Os05g31480.1	5
3735	LOC_Os05g39520.1	5
3735	LOC_Os06g03750.1	6
3735	LOC_Os06g49860.1	6
3735	LOC_Os10g33720.1	10
3735	LOC_Os10g36690.1	10
3735	LOC_Os10g37770.1	10
3735	LOC_Os11g08314.1	11
3735	LOC_Os12g07870.1	12
3735	LOC_Os12g07840.1	12
3736	LOC_Os01g54620.1	1
3736	LOC_Os02g49332.1	2
3736	LOC_Os04g35020.1	4
3736	LOC_Os06g02180.1	6
3736	LOC_Os06g22980.1	6
3736	LOC_Os06g39970.1	6

3736	LOC_Os07g36610.1	7
3736	LOC_Os07g36630.1	7
3736	LOC_Os07g36690.1	7
3736	LOC_Os07g36700.1	7
3736	LOC_Os07g36740.1	7
3736	LOC_Os07g36750.1	7
3736	LOC_Os08g06380.1	8
3736	LOC_Os08g25710.1	8
3736	LOC_Os09g30120.1	9
3736	LOC_Os09g30130.1	9
3736	LOC_Os10g20090.1	10
3736	LOC_Os10g20260.1	10
3736	LOC_Os10g32980.1	10
3736	LOC_Os10g42750.1	10
3736	LOC_Os12g36890.1	12
3737	LOC_Os02g20670.1	2
3737	LOC_Os03g26900.1	3
3737	LOC_Os04g01390.1	4
3737	LOC_Os04g05840.1	4
3737	LOC_Os06g26300.1	6
3737	LOC_Os06g34570.1	6
3737	LOC_Os07g20770.1	7
3737	LOC_Os07g26370.1	7
3737	LOC_Os08g11890.1	8
3737	LOC_Os10g13790.1	10
3737	LOC_Os10g15320.1	10
3737	LOC_Os10g26200.1	10
3737	LOC_Os11g08720.1	11
3737	LOC_Os11g08900.1	11
3737	LOC_Os11g15660.1	11
3737	LOC_Os11g19580.1	11
3737	LOC_Os11g19970.1	11
3737	LOC_Os11g20730.1	11
3737	LOC_Os11g45450.1	11
3737	LOC_Os12g22040.1	12
3737	LOC_Os12g22450.1	12
3738	LOC_Os01g25910.1	1
3738	LOC_Os03g43830.1	3
3738	LOC_Os05g13930.1	5
3738	LOC_Os05g17380.1	5
3738	LOC_Os05g22640.1	5
3738	LOC_Os06g19910.1	6
3738	LOC_Os06g24120.1	6
3738	LOC_Os06g28900.1	6
3738	LOC_Os08g12580.1	8
3738	LOC_Os08g23010.1	8
3738	LOC_Os08g27560.1	8
3738	LOC_Os08g29380.1	8
3738	LOC_Os10g03820.1	10
3738	LOC_Os10g08070.1	10
3738	LOC_Os10g11940.1	10
3738	LOC_Os10g14840.1	10

3738	LOC_Os10g17020.1	10
3738	LOC_Os11g23910.1	11
3738	LOC_Os12g17440.1	12
3738	LOC_Os12g20030.1	12
3738	LOC_Os12g30660.1	12
3739	LOC_Os01g33940.1	1
3739	LOC_Os02g16930.1	2
3739	LOC_Os02g28840.1	2
3739	LOC_Os04g24560.1	4
3739	LOC_Os05g32930.1	5
3739	LOC_Os06g16180.1	6
3739	LOC_Os06g36690.1	6
3739	LOC_Os07g07960.1	7
3739	LOC_Os07g10280.1	7
3739	LOC_Os07g27250.1	7
3739	LOC_Os08g14160.1	8
3739	LOC_Os08g33970.1	8
3739	LOC_Os08g38430.1	8
3739	LOC_Os09g23210.1	9
3739	LOC_Os11g07220.1	11
3739	LOC_Os11g11560.1	11
3739	LOC_Os11g11570.1	11
3739	LOC_Os11g34580.1	11
3739	LOC_Os12g09180.1	12
3739	LOC_Os12g16370.1	12
3739	LOC_Os12g34430.1	12
3740	LOC_Os01g48270.1	1
3740	LOC_Os01g49000.1	1
3740	LOC_Os01g55260.1	1
3740	LOC_Os02g46990.1	2
3740	LOC_Os02g47760.1	2
3740	LOC_Os02g50680.1	2
3740	LOC_Os02g53500.1	2
3740	LOC_Os03g15810.1	3
3740	LOC_Os04g21660.1	4
3740	LOC_Os04g39190.1	4
3740	LOC_Os04g40290.1	4
3740	LOC_Os04g48060.1	4
3740	LOC_Os04g56320.1	4
3740	LOC_Os05g44310.1	5
3740	LOC_Os06g03940.1	6
3740	LOC_Os06g12160.1	6
3740	LOC_Os06g50050.1	6
3740	LOC_Os08g44240.1	8
3740	LOC_Os11g13990.1	11
3740	LOC_Os11g47970.1	11
3740	LOC_Os12g07720.1	12
3740	LOC_Os12g10670.1	12
3741	LOC_Os01g27700.1	1
3741	LOC_Os01g44330.1	1
3741	LOC_Os01g61160.1	1
3741	LOC_Os01g62480.1	1

3741	LOC_Os01g62490.1	1
3741	LOC_Os01g62600.1	1
3741	LOC_Os01g63180.1	1
3741	LOC_Os01g63190.1	1
3741	LOC_Os01g63200.1	1
3741	LOC_Os02g51440.1	2
3741	LOC_Os03g16610.1	3
3741	LOC_Os03g18640.1	3
3741	LOC_Os05g38410.1	5
3741	LOC_Os05g38420.1	5
3741	LOC_Os05g38390.1	5
3741	LOC_Os07g01110.1	7
3741	LOC_Os11g16260.1	11
3741	LOC_Os11g42220.1	11
3741	LOC_Os11g42200.1	11
3741	LOC_Os11g47390.1	11
3741	LOC_Os12g15680.1	12
3741	LOC_Os12g15920.1	12
3742	LOC_Os01g01290.1	1
3742	LOC_Os01g08790.1	1
3742	LOC_Os01g24460.1	1
3742	LOC_Os01g39850.1	1
3742	LOC_Os01g61810.1	1
3742	LOC_Os01g70880.1	1
3742	LOC_Os01g70890.1	1
3742	LOC_Os02g49410.1	2
3742	LOC_Os02g49370.1	2
3742	LOC_Os03g29970.1	3
3742	LOC_Os03g63530.1	3
3742	LOC_Os05g23910.1	5
3742	LOC_Os05g41450.1	5
3742	LOC_Os05g49780.1	5
3742	LOC_Os05g38820.1	5
3742	LOC_Os06g17480.1	6
3742	LOC_Os07g41580.1	7
3742	LOC_Os08g07740.1	8
3742	LOC_Os08g29500.1	8
3742	LOC_Os09g39490.1	9
3742	LOC_Os10g11580.1	10
3742	LOC_Os11g34200.1	11
3743	LOC_Os01g12130.1	1
3743	LOC_Os01g21230.1	1
3743	LOC_Os01g36070.1	1
3743	LOC_Os01g40960.1	1
3743	LOC_Os01g42110.1	1
3743	LOC_Os01g42090.1	1
3743	LOC_Os01g65880.1	1
3743	LOC_Os01g50460.1	1
3743	LOC_Os02g19820.1	2
3743	LOC_Os02g30910.1	2
3743	LOC_Os03g22200.1	3
3743	LOC_Os03g22590.1	3

3743	LOC_Os05g12320.1	5
3743	LOC_Os05g35140.1	5
3743	LOC_Os05g51090.1	5
3743	LOC_Os08g42350.1	8
3743	LOC_Os09g08030.1	9
3743	LOC_Os09g08270.1	9
3743	LOC_Os09g08440.1	9
3743	LOC_Os11g31190.1	11
3743	LOC_Os12g07860.1	12
3743	LOC_Os12g29220.1	12
3744	LOC_Os01g24050.1	1
3744	LOC_Os02g14810.1	2
3744	LOC_Os02g14870.1	2
3744	LOC_Os04g12110.1	4
3744	LOC_Os04g14540.1	4
3744	LOC_Os05g14680.1	5
3744	LOC_Os05g25890.1	5
3744	LOC_Os05g46690.1	5
3744	LOC_Os06g49000.1	6
3744	LOC_Os07g01840.1	7
3744	LOC_Os07g08270.1	7
3744	LOC_Os07g08290.1	7
3744	LOC_Os07g16600.1	7
3744	LOC_Os07g16930.1	7
3744	LOC_Os09g38660.1	9
3744	LOC_Os09g38930.1	9
3744	LOC_Os10g05410.1	10
3744	LOC_Os10g05440.1	10
3744	LOC_Os10g20770.1	10
3744	LOC_Os10g39570.1	10
3744	LOC_Os11g15510.1	11
3744	LOC_Os11g39920.1	11
3745	LOC_Os04g09500.1	4
3745	LOC_Os04g12360.1	4
3745	LOC_Os04g13660.1	4
3745	LOC_Os04g16410.1	4
3745	LOC_Os04g22560.1	4
3745	LOC_Os05g39290.1	5
3745	LOC_Os06g07730.1	6
3745	LOC_Os06g20600.1	6
3745	LOC_Os07g18330.1	7
3745	LOC_Os07g28330.1	7
3745	LOC_Os08g04040.1	8
3745	LOC_Os08g19730.1	8
3745	LOC_Os08g22520.1	8
3745	LOC_Os08g29230.1	8
3745	LOC_Os10g01370.1	10
3745	LOC_Os10g16400.1	10
3745	LOC_Os10g17380.1	10
3745	LOC_Os10g17800.1	10
3745	LOC_Os10g24210.1	10
3745	LOC_Os11g02870.1	11

3745	LOC_Os11g18410.1	11
3745	LOC_Os11g25680.1	11
3746	LOC_Os01g47710.1	1
3746	LOC_Os01g48170.1	1
3746	LOC_Os01g60270.1	1
3746	LOC_Os01g62310.1	1
3746	LOC_Os01g63510.1	1
3746	LOC_Os01g70810.1	1
3746	LOC_Os03g10210.1	3
3746	LOC_Os03g55990.1	3
3746	LOC_Os03g20910.1	3
3746	LOC_Os04g55590.1	4
3746	LOC_Os04g56780.1	4
3746	LOC_Os05g02730.1	5
3746	LOC_Os05g48990.1	5
3746	LOC_Os06g29020.1	6
3746	LOC_Os06g39906.1	6
3746	LOC_Os07g24350.1	7
3746	LOC_Os07g34880.1	7
3746	LOC_Os07g39320.1	7
3746	LOC_Os07g48560.1	7
3746	LOC_Os08g14400.1	8
3746	LOC_Os10g39720.1	10
3746	LOC_Os11g01130.1	11
3746	LOC_Os12g01120.1	12
3747	LOC_Os01g45570.1	1
3747	LOC_Os02g05640.1	2
3747	LOC_Os02g35770.1	2
3747	LOC_Os02g43330.1	2
3747	LOC_Os02g49700.1	2
3747	LOC_Os03g08960.1	3
3747	LOC_Os03g12860.1	3
3747	LOC_Os03g07450.1	3
3747	LOC_Os04g45810.1	4
3747	LOC_Os04g46350.1	4
3747	LOC_Os06g04850.1	6
3747	LOC_Os06g04870.1	6
3747	LOC_Os06g48290.1	6
3747	LOC_Os08g32080.1	8
3747	LOC_Os08g37580.1	8
3747	LOC_Os09g21180.1	9
3747	LOC_Os09g27450.1	9
3747	LOC_Os09g29460.1	9
3747	LOC_Os09g35910.1	9
3747	LOC_Os10g01470.1	10
3747	LOC_Os10g26500.1	10
3747	LOC_Os10g41230.1	10
3747	LOC_Os10g23090.1	10
3748	LOC_Os01g03820.1	1
3748	LOC_Os01g14050.1	1
3748	LOC_Os01g27190.1	1
3748	LOC_Os01g47040.1	1

3748	LOC_Os01g62430.1	1
3748	LOC_Os01g70790.1	1
3748	LOC_Os01g72420.1	1
3748	LOC_Os02g20970.1	2
3748	LOC_Os02g42710.1	2
3748	LOC_Os02g44560.1	2
3748	LOC_Os02g57000.1	2
3748	LOC_Os02g58230.1	2
3748	LOC_Os03g09840.1	3
3748	LOC_Os04g44870.1	4
3748	LOC_Os04g58570.1	4
3748	LOC_Os05g05650.1	5
3748	LOC_Os05g30740.1	5
3748	LOC_Os06g43190.1	6
3748	LOC_Os07g39620.1	7
3748	LOC_Os07g47390.1	7
3748	LOC_Os07g47400.1	7
3748	LOC_Os08g44850.1	8
3748	LOC_Os09g39770.1	9
3749	LOC_Os01g59600.1	1
3749	LOC_Os02g04100.1	2
3749	LOC_Os02g08520.1	2
3749	LOC_Os02g42320.1	2
3749	LOC_Os02g53060.1	2
3749	LOC_Os03g26970.1	3
3749	LOC_Os03g48930.1	3
3749	LOC_Os03g08280.1	3
3749	LOC_Os05g09490.1	5
3749	LOC_Os05g41180.1	5
3749	LOC_Os06g04800.1	6
3749	LOC_Os06g06030.1	6
3749	LOC_Os06g07140.1	6
3749	LOC_Os06g07978.1	6
3749	LOC_Os06g07878.1	6
3749	LOC_Os06g43570.1	6
3749	LOC_Os07g42260.1	7
3749	LOC_Os08g41730.1	8
3749	LOC_Os08g43540.1	8
3749	LOC_Os09g32800.1	9
3749	LOC_Os09g36710.1	9
3749	LOC_Os09g33986.1	9
3749	LOC_Os11g40140.1	11
3750	LOC_Os02g13350.1	2
3750	LOC_Os02g32060.1	2
3750	LOC_Os02g50130.1	2
3750	LOC_Os02g55030.1	2
3750	LOC_Os02g56210.1	2
3750	LOC_Os03g59580.1	3
3750	LOC_Os04g32740.1	4
3750	LOC_Os04g46280.1	4
3750	LOC_Os04g58900.1	4
3750	LOC_Os05g02640.1	5

3750	LOC_Os05g11870.1	5
3750	LOC_Os05g34180.1	5
3750	LOC_Os06g03910.1	6
3750	LOC_Os06g04910.1	6
3750	LOC_Os06g14420.1	6
3750	LOC_Os06g42790.1	6
3750	LOC_Os07g11120.1	7
3750	LOC_Os07g36190.1	7
3750	LOC_Os08g28840.1	8
3750	LOC_Os08g28860.1	8
3750	LOC_Os09g15340.1	9
3750	LOC_Os09g38040.1	9
3750	LOC_Os11g32750.1	11
3751	LOC_Os01g62260.1	1
3751	LOC_Os02g02200.1	2
3751	LOC_Os03g14030.1	3
3751	LOC_Os03g45960.1	3
3751	LOC_Os03g46070.1	3
3751	LOC_Os04g59370.1	4
3751	LOC_Os06g50240.1	6
3751	LOC_Os07g23730.1	7
3751	LOC_Os07g23470.1	7
3751	LOC_Os08g40600.1	8
3751	LOC_Os08g43510.1	8
3751	LOC_Os09g32280.1	9
3751	LOC_Os09g36580.1	9
3751	LOC_Os09g36560.1	9
3751	LOC_Os10g05660.1	10
3751	LOC_Os10g27280.1	10
3751	LOC_Os11g47944.1	11
3751	LOC_Os12g43410.1	12
3751	LOC_Os12g43380.1	12
3751	LOC_Os12g43390.1	12
3751	LOC_Os12g43490.1	12
3751	LOC_Os12g43430.1	12
3751	LOC_Os12g43440.1	12
3752	LOC_Os01g12490.1	1
3752	LOC_Os01g16714.1	1
3752	LOC_Os01g16750.1	1
3752	LOC_Os01g27050.1	1
3752	LOC_Os01g26876.1	1
3752	LOC_Os01g53200.1	1
3752	LOC_Os02g17230.1	2
3752	LOC_Os03g06654.1	3
3752	LOC_Os03g08410.1	3
3752	LOC_Os04g03980.1	4
3752	LOC_Os04g14710.1	4
3752	LOC_Os04g14690.1	4
3752	LOC_Os05g45240.1	5
3752	LOC_Os06g10170.1	6
3752	LOC_Os07g25540.1	7
3752	LOC_Os09g37620.1	9

3752	LOC_Os09g37650.1	9
3752	LOC_Os09g37690.1	9
3752	LOC_Os10g40610.1	10
3752	LOC_Os11g10140.1	11
3752	LOC_Os11g10170.1	11
3752	LOC_Os12g08780.1	12
3752	LOC_Os12g32750.1	12
3753	LOC_Os01g02070.1	1
3753	LOC_Os01g14940.1	1
3753	LOC_Os01g20970.1	1
3753	LOC_Os01g50810.1	1
3753	LOC_Os02g01300.1	2
3753	LOC_Os02g01310.1	2
3753	LOC_Os02g46290.1	2
3753	LOC_Os02g46360.1	2
3753	LOC_Os03g61530.1	3
3753	LOC_Os04g01570.1	4
3753	LOC_Os04g49730.1	4
3753	LOC_Os05g29740.1	5
3753	LOC_Os05g20570.1	5
3753	LOC_Os05g46530.1	5
3753	LOC_Os06g49760.1	6
3753	LOC_Os08g01670.1	8
3753	LOC_Os08g24160.1	8
3753	LOC_Os10g10560.1	10
3753	LOC_Os10g10700.1	10
3753	LOC_Os10g10620.1	10
3753	LOC_Os10g36500.1	10
3753	LOC_Os12g18560.1	12
3753	LOC_Os12g37480.1	12
3754	LOC_Os01g07590.1	1
3754	LOC_Os01g19820.1	1
3754	LOC_Os01g32780.1	1
3754	LOC_Os01g57450.1	1
3754	LOC_Os01g63010.1	1
3754	LOC_Os01g65440.1	1
3754	LOC_Os02g47650.1	2
3754	LOC_Os02g53320.1	2
3754	LOC_Os03g19270.1	3
3754	LOC_Os03g22390.1	3
3754	LOC_Os03g40130.1	3
3754	LOC_Os03g53900.1	3
3754	LOC_Os05g06500.1	5
3754	LOC_Os05g07810.1	5
3754	LOC_Os05g28740.1	5
3754	LOC_Os05g35380.1	5
3754	LOC_Os05g42230.1	5
3754	LOC_Os05g37970.1	5
3754	LOC_Os07g47620.1	7
3754	LOC_Os10g30150.1	10
3754	LOC_Os10g32590.1	10
3754	LOC_Os12g31710.1	12

3754	LOC_Os12g36630.1	12
3754	LOC_Os12g36640.1	12
3755	LOC_Os01g42470.1	1
3755	LOC_Os02g48650.1	2
3755	LOC_Os02g53260.1	2
3755	LOC_Os03g05710.1	3
3755	LOC_Os03g07990.1	3
3755	LOC_Os03g09860.1	3
3755	LOC_Os03g46200.1	3
3755	LOC_Os03g46250.1	3
3755	LOC_Os03g49230.1	3
3755	LOC_Os03g58010.1	3
3755	LOC_Os03g58020.1	3
3755	LOC_Os03g58030.1	3
3755	LOC_Os04g39140.1	4
3755	LOC_Os04g54330.1	4
3755	LOC_Os05g32180.1	5
3755	LOC_Os05g31254.1	5
3755	LOC_Os05g40260.1	5
3755	LOC_Os07g16130.1	7
3755	LOC_Os08g01170.1	8
3755	LOC_Os08g16010.1	8
3755	LOC_Os09g31310.1	9
3755	LOC_Os10g35680.1	10
3755	LOC_Os11g32280.1	11
3755	LOC_Os12g37490.1	12
3756	LOC_Os01g48290.1	1
3756	LOC_Os01g55340.1	1
3756	LOC_Os01g64590.1	1
3756	LOC_Os02g15350.1	2
3756	LOC_Os02g47810.1	2
3756	LOC_Os02g49440.1	2
3756	LOC_Os02g45200.1	2
3756	LOC_Os03g16850.1	3
3756	LOC_Os03g38870.1	3
3756	LOC_Os03g42200.1	3
3756	LOC_Os03g55610.1	3
3756	LOC_Os03g60630.1	3
3756	LOC_Os04g47990.1	4
3756	LOC_Os04g58190.1	4
3756	LOC_Os05g36900.1	5
3756	LOC_Os06g17410.1	6
3756	LOC_Os07g13260.1	7
3756	LOC_Os07g32510.1	7
3756	LOC_Os08g38220.1	8
3756	LOC_Os09g29960.1	9
3756	LOC_Os10g26620.1	10
3756	LOC_Os10g35300.1	10
3756	LOC_Os12g38200.1	12
3756	LOC_Os12g39990.1	12
3757	LOC_Os01g27170.1	1
3757	LOC_Os01g70490.1	1

3757	LOC_Os01g70660.1	1
3757	LOC_Os01g70940.1	1
3757	LOC_Os02g31910.1	2
3757	LOC_Os02g31940.1	2
3757	LOC_Os02g49760.1	2
3757	LOC_Os03g21890.1	3
3757	LOC_Os03g37930.1	3
3757	LOC_Os03g37830.1	3
3757	LOC_Os03g37840.1	3
3757	LOC_Os04g32920.1	4
3757	LOC_Os04g52120.1	4
3757	LOC_Os04g52390.1	4
3757	LOC_Os06g45940.1	6
3757	LOC_Os07g32530.1	7
3757	LOC_Os07g48130.1	7
3757	LOC_Os07g47350.1	7
3757	LOC_Os08g10550.1	8
3757	LOC_Os08g36340.1	8
3757	LOC_Os08g39950.1	8
3757	LOC_Os09g21000.1	9
3757	LOC_Os09g27580.1	9
3757	LOC_Os09g38960.1	9
3758	LOC_Os01g11240.1	1
3758	LOC_Os01g38500.1	1
3758	LOC_Os01g41220.1	1
3758	LOC_Os01g46390.1	1
3758	LOC_Os03g11210.1	3
3758	LOC_Os03g18560.1	3
3758	LOC_Os04g36600.1	4
3758	LOC_Os05g27950.1	5
3758	LOC_Os05g34810.1	5
3758	LOC_Os05g49790.1	5
3758	LOC_Os06g34790.1	6
3758	LOC_Os07g02690.1	7
3758	LOC_Os07g02870.1	7
3758	LOC_Os07g02880.1	7
3758	LOC_Os07g02700.1	7
3758	LOC_Os07g02850.1	7
3758	LOC_Os07g02900.1	7
3758	LOC_Os07g02940.1	7
3758	LOC_Os07g02920.1	7
3758	LOC_Os10g37400.1	10
3758	LOC_Os11g38210.1	11
3758	LOC_Os11g38220.1	11
3758	LOC_Os11g38240.1	11
3758	LOC_Os12g37650.1	12
3759	LOC_Os01g04250.1	1
3759	LOC_Os01g35880.1	1
3759	LOC_Os02g06310.1	2
3759	LOC_Os02g16690.1	2
3759	LOC_Os02g17930.1	2
3759	LOC_Os02g25600.1	2

3759	LOC_Os05g03400.1	5
3759	LOC_Os05g34490.1	5
3759	LOC_Os06g16140.1	6
3759	LOC_Os06g17500.1	6
3759	LOC_Os06g18810.1	6
3759	LOC_Os06g19340.1	6
3759	LOC_Os06g28840.1	6
3759	LOC_Os06g37790.1	6
3759	LOC_Os07g45120.1	7
3759	LOC_Os08g12180.1	8
3759	LOC_Os08g12220.1	8
3759	LOC_Os10g26490.1	10
3759	LOC_Os10g35160.1	10
3759	LOC_Os11g36820.1	11
3759	LOC_Os12g10480.1	12
3759	LOC_Os12g28020.1	12
3759	LOC_Os12g36330.1	12
3759	LOC_Os12g40440.1	12
3760	LOC_Os01g14090.1	1
3760	LOC_Os01g15540.1	1
3760	LOC_Os01g42070.1	1
3760	LOC_Os02g13580.1	2
3760	LOC_Os02g53520.1	2
3760	LOC_Os02g56540.1	2
3760	LOC_Os02g01180.1	2
3760	LOC_Os03g53920.1	3
3760	LOC_Os03g56260.1	3
3760	LOC_Os03g64415.1	3
3760	LOC_Os03g02290.1	3
3760	LOC_Os04g30720.1	4
3760	LOC_Os04g57140.1	4
3760	LOC_Os04g53760.1	4
3760	LOC_Os05g02670.1	5
3760	LOC_Os05g33030.1	5
3760	LOC_Os07g01490.1	7
3760	LOC_Os08g02380.1	8
3760	LOC_Os08g44420.1	8
3760	LOC_Os09g02650.1	9
3760	LOC_Os10g36880.1	10
3760	LOC_Os11g35090.1	11
3760	LOC_Os11g42800.1	11
3760	LOC_Os12g36100.1	12
3760	LOC_Os12g42160.1	12
3761	LOC_Os04g01470.1	4
3761	LOC_Os04g09604.1	4
3761	LOC_Os04g09654.1	4
3761	LOC_Os05g43930.1	5
3761	LOC_Os05g43940.1	5
3761	LOC_Os06g13280.1	6
3761	LOC_Os06g16960.1	6
3761	LOC_Os07g27970.1	7
3761	LOC_Os08g06100.1	8

3761	LOC_Os08g07260.1	8
3761	LOC_Os08g19420.1	8
3761	LOC_Os08g35310.1	8
3761	LOC_Os09g17560.1	9
3761	LOC_Os10g02880.1	10
3761	LOC_Os11g12760.1	11
3761	LOC_Os11g19840.1	11
3761	LOC_Os11g19880.1	11
3761	LOC_Os11g20040.1	11
3761	LOC_Os11g20090.1	11
3761	LOC_Os11g20160.1	11
3761	LOC_Os11g33300.1	11
3761	LOC_Os12g09770.1	12
3761	LOC_Os12g25450.1	12
3761	LOC_Os12g25490.1	12
3761	LOC_Os12g25820.1	12
3762	LOC_Os01g19440.1	1
3762	LOC_Os01g21034.1	1
3762	LOC_Os01g44340.1	1
3762	LOC_Os01g53990.1	1
3762	LOC_Os01g65790.1	1
3762	LOC_Os02g46310.1	2
3762	LOC_Os03g19610.1	3
3762	LOC_Os04g35770.1	4
3762	LOC_Os04g43370.1	4
3762	LOC_Os04g46740.1	4
3762	LOC_Os05g44600.1	5
3762	LOC_Os07g41650.1	7
3762	LOC_Os07g46190.1	7
3762	LOC_Os08g10604.1	8
3762	LOC_Os08g12410.1	8
3762	LOC_Os09g37360.1	9
3762	LOC_Os09g39760.1	9
3762	LOC_Os10g26680.1	10
3762	LOC_Os11g08750.1	11
3762	LOC_Os11g36240.1	11
3762	LOC_Os11g43830.1	11
3762	LOC_Os11g43850.1	11
3762	LOC_Os11g45720.1	11
3762	LOC_Os11g45730.1	11
3762	LOC_Os12g37660.1	12
3763	LOC_Os01g52710.1	1
3763	LOC_Os02g29530.1	2
3763	LOC_Os02g41520.1	2
3763	LOC_Os02g50600.1	2
3763	LOC_Os02g51130.1	2
3763	LOC_Os03g11330.1	3
3763	LOC_Os03g18890.1	3
3763	LOC_Os03g20120.1	3
3763	LOC_Os03g24510.1	3
3763	LOC_Os03g30000.1	3
3763	LOC_Os03g47530.1	3

3763	LOC_Os04g43700.1	4
3763	LOC_Os04g44850.1	4
3763	LOC_Os04g46750.1	4
3763	LOC_Os04g54360.1	4
3763	LOC_Os06g12280.1	6
3763	LOC_Os06g13760.1	6
3763	LOC_Os07g45260.1	7
3763	LOC_Os07g48830.1	7
3763	LOC_Os08g23780.1	8
3763	LOC_Os08g38740.1	8
3763	LOC_Os09g30280.1	9
3763	LOC_Os10g31650.1	10
3763	LOC_Os10g40640.1	10
3763	LOC_Os12g38930.1	12
3764	LOC_Os01g59630.1	1
3764	LOC_Os01g69220.1	1
3764	LOC_Os01g70180.1	1
3764	LOC_Os01g70190.1	1
3764	LOC_Os01g70200.1	1
3764	LOC_Os02g09430.1	2
3764	LOC_Os02g32110.1	2
3764	LOC_Os03g01760.1	3
3764	LOC_Os03g05060.1	3
3764	LOC_Os03g05070.1	3
3764	LOC_Os03g20850.1	3
3764	LOC_Os04g32670.1	4
3764	LOC_Os04g48480.1	4
3764	LOC_Os04g57510.1	4
3764	LOC_Os06g23420.1	6
3764	LOC_Os06g43160.1	6
3764	LOC_Os07g37960.1	7
3764	LOC_Os08g34020.1	8
3764	LOC_Os10g32110.1	10
3764	LOC_Os10g40559.1	10
3764	LOC_Os10g10080.1	10
3764	LOC_Os11g03410.1	11
3764	LOC_Os12g03100.1	12
3764	LOC_Os12g12290.1	12
3764	LOC_Os12g38450.1	12
3765	LOC_Os02g17790.1	2
3765	LOC_Os02g29790.1	2
3765	LOC_Os03g32460.1	3
3765	LOC_Os03g34096.1	3
3765	LOC_Os04g07440.1	4
3765	LOC_Os05g18180.1	5
3765	LOC_Os05g20630.1	5
3765	LOC_Os05g21200.1	5
3765	LOC_Os05g28240.1	5
3765	LOC_Os06g27410.1	6
3765	LOC_Os06g51560.1	6
3765	LOC_Os07g15060.1	7
3765	LOC_Os07g20740.1	7

3765	LOC_Os08g17250.1	8
3765	LOC_Os08g22920.1	8
3765	LOC_Os08g26140.1	8
3765	LOC_Os09g04140.1	9
3765	LOC_Os09g06420.1	9
3765	LOC_Os09g16490.1	9
3765	LOC_Os10g03180.1	10
3765	LOC_Os11g15150.1	11
3765	LOC_Os11g22420.1	11
3765	LOC_Os11g23070.1	11
3765	LOC_Os12g15130.1	12
3765	LOC_Os12g33500.1	12
3766	LOC_Os01g19090.1	1
3766	LOC_Os01g23190.1	1
3766	LOC_Os01g34240.1	1
3766	LOC_Os02g28890.1	2
3766	LOC_Os03g41720.1	3
3766	LOC_Os03g44350.1	3
3766	LOC_Os03g59820.1	3
3766	LOC_Os04g04610.1	4
3766	LOC_Os04g05740.1	4
3766	LOC_Os04g07520.1	4
3766	LOC_Os04g07530.1	4
3766	LOC_Os04g13980.1	4
3766	LOC_Os04g43940.1	4
3766	LOC_Os07g17100.1	7
3766	LOC_Os07g20320.1	7
3766	LOC_Os07g25320.1	7
3766	LOC_Os07g30440.1	7
3766	LOC_Os10g28920.1	10
3766	LOC_Os11g19920.1	11
3766	LOC_Os11g23990.1	11
3766	LOC_Os11g24040.1	11
3766	LOC_Os11g28870.1	11
3766	LOC_Os11g30870.1	11
3766	LOC_Os12g18330.1	12
3766	LOC_Os12g23070.1	12
3767	LOC_Os01g43410.1	1
3767	LOC_Os01g59360.1	1
3767	LOC_Os02g46090.1	2
3767	LOC_Os02g58520.1	2
3767	LOC_Os03g48270.1	3
3767	LOC_Os03g57450.1	3
3767	LOC_Os03g57510.1	3
3767	LOC_Os03g59390.1	3
3767	LOC_Os03g03660.1	3
3767	LOC_Os04g47300.1	4
3767	LOC_Os04g49510.1	4
3767	LOC_Os05g41270.1	5
3767	LOC_Os05g41090.1	5
3767	LOC_Os05g50810.1	5
3767	LOC_Os07g06740.1	7

3767	LOC_Os07g33110.1	7
3767	LOC_Os07g38120.1	7
3767	LOC_Os08g42750.1	8
3767	LOC_Os09g33910.1	9
3767	LOC_Os10g39420.1	10
3767	LOC_Os11g04170.1	11
3767	LOC_Os11g07040.1	11
3767	LOC_Os12g03970.1	12
3767	LOC_Os12g07230.1	12
3767	LOC_Os12g12860.1	12
3767	LOC_Os12g30150.1	12
3768	LOC_Os01g11270.1	1
3768	LOC_Os01g11340.1	1
3768	LOC_Os01g50530.1	1
3768	LOC_Os01g50580.1	1
3768	LOC_Os01g59050.1	1
3768	LOC_Os01g72260.1	1
3768	LOC_Os02g45280.1	2
3768	LOC_Os03g12660.1	3
3768	LOC_Os03g21400.1	3
3768	LOC_Os03g37080.1	3
3768	LOC_Os03g40540.1	3
3768	LOC_Os03g45619.1	3
3768	LOC_Os04g39430.1	4
3768	LOC_Os04g48170.1	4
3768	LOC_Os04g48200.1	4
3768	LOC_Os04g48210.1	4
3768	LOC_Os05g31740.1	5
3768	LOC_Os05g33590.1	5
3768	LOC_Os05g34380.1	5
3768	LOC_Os06g02019.1	6
3768	LOC_Os07g29960.1	7
3768	LOC_Os07g33540.1	7
3768	LOC_Os07g45000.1	7
3768	LOC_Os10g10040.1	10
3768	LOC_Os11g18570.1	11
3768	LOC_Os12g18820.1	12
3769	LOC_Os01g07790.1	1
3769	LOC_Os01g19170.1	1
3769	LOC_Os01g33300.1	1
3769	LOC_Os01g36830.1	1
3769	LOC_Os01g43490.1	1
3769	LOC_Os01g44970.1	1
3769	LOC_Os01g45060.1	1
3769	LOC_Os02g03750.1	2
3769	LOC_Os02g10300.1	2
3769	LOC_Os03g11760.1	3
3769	LOC_Os05g20020.1	5
3769	LOC_Os05g46520.1	5
3769	LOC_Os05g46510.1	5
3769	LOC_Os05g50260.1	5
3769	LOC_Os06g28670.1	6

3769	LOC_Os06g31270.1	6
3769	LOC_Os06g35300.1	6
3769	LOC_Os06g35320.1	6
3769	LOC_Os06g35370.1	6
3769	LOC_Os06g40880.1	6
3769	LOC_Os06g40890.1	6
3769	LOC_Os07g10680.1	7
3769	LOC_Os07g10740.1	7
3769	LOC_Os11g14400.1	11
3769	LOC_Os11g14410.1	11
3769	LOC_Os11g43750.1	11
3770	LOC_Os01g11670.1	1
3770	LOC_Os01g22954.1	1
3770	LOC_Os01g22980.1	1
3770	LOC_Os01g43890.1	1
3770	LOC_Os01g61690.1	1
3770	LOC_Os02g02320.1	2
3770	LOC_Os02g26480.1	2
3770	LOC_Os02g42310.1	2
3770	LOC_Os02g55130.1	2
3770	LOC_Os02g46260.1	2
3770	LOC_Os03g09190.1	3
3770	LOC_Os03g26930.1	3
3770	LOC_Os03g52080.1	3
3770	LOC_Os04g09720.1	4
3770	LOC_Os04g44410.1	4
3770	LOC_Os05g06660.1	5
3770	LOC_Os05g50570.1	5
3770	LOC_Os05g50580.1	5
3770	LOC_Os05g50600.1	5
3770	LOC_Os06g36570.1	6
3770	LOC_Os06g51370.1	6
3770	LOC_Os07g29620.1	7
3770	LOC_Os09g28830.1	9
3770	LOC_Os10g01134.1	10
3770	LOC_Os11g31980.1	11
3770	LOC_Os12g39170.1	12
3771	LOC_Os01g14730.1	1
3771	LOC_Os01g43200.1	1
3771	LOC_Os02g11120.1	2
3771	LOC_Os02g26820.1	2
3771	LOC_Os03g04720.1	3
3771	LOC_Os03g20150.1	3
3771	LOC_Os03g22220.1	3
3771	LOC_Os03g26730.1	3
3771	LOC_Os04g01080.1	4
3771	LOC_Os04g06560.1	4
3771	LOC_Os04g28700.1	4
3771	LOC_Os04g49830.1	4
3771	LOC_Os06g14950.1	6
3771	LOC_Os06g24030.1	6
3771	LOC_Os06g25000.1	6

3771	LOC_Os06g35450.1	6
3771	LOC_Os07g12750.1	7
3771	LOC_Os07g16290.1	7
3771	LOC_Os08g41930.1	8
3771	LOC_Os09g20770.1	9
3771	LOC_Os11g12170.1	11
3771	LOC_Os12g12530.1	12
3771	LOC_Os12g13870.1	12
3771	LOC_Os12g17400.1	12
3771	LOC_Os12g19840.1	12
3771	LOC_Os12g28960.1	12
3772	LOC_Os01g40970.1	1
3772	LOC_Os01g43610.1	1
3772	LOC_Os01g53160.1	1
3772	LOC_Os01g54570.1	1
3772	LOC_Os01g60810.1	1
3772	LOC_Os01g64430.1	1
3772	LOC_Os02g45620.1	2
3772	LOC_Os03g03480.1	3
3772	LOC_Os03g06350.1	3
3772	LOC_Os03g10150.1	3
3772	LOC_Os03g21870.1	3
3772	LOC_Os04g33870.1	4
3772	LOC_Os04g37510.1	4
3772	LOC_Os04g48830.1	4
3772	LOC_Os05g12808.1	5
3772	LOC_Os05g25910.1	5
3772	LOC_Os05g44090.1	5
3772	LOC_Os05g36970.1	5
3772	LOC_Os05g39950.1	5
3772	LOC_Os07g48150.1	7
3772	LOC_Os10g29610.1	10
3772	LOC_Os10g38880.1	10
3772	LOC_Os11g05770.1	11
3772	LOC_Os11g05780.1	11
3772	LOC_Os12g06150.1	12
3772	LOC_Os12g06160.1	12
3773	LOC_Os02g25800.1	2
3773	LOC_Os03g32850.1	3
3773	LOC_Os03g32690.1	3
3773	LOC_Os03g34270.1	3
3773	LOC_Os04g02090.1	4
3773	LOC_Os04g04930.1	4
3773	LOC_Os05g18830.1	5
3773	LOC_Os05g12350.1	5
3773	LOC_Os05g23980.1	5
3773	LOC_Os06g25380.1	6
3773	LOC_Os06g28930.1	6
3773	LOC_Os06g30980.1	6
3773	LOC_Os06g40000.1	6
3773	LOC_Os06g46170.1	6
3773	LOC_Os07g08900.1	7

3773	LOC_Os07g11460.1	7
3773	LOC_Os08g11040.1	8
3773	LOC_Os09g12080.1	9
3773	LOC_Os09g14640.1	9
3773	LOC_Os10g08130.1	10
3773	LOC_Os10g12730.1	10
3773	LOC_Os10g17620.1	10
3773	LOC_Os10g18350.1	10
3773	LOC_Os10g30010.1	10
3773	LOC_Os12g04820.1	12
3773	LOC_Os12g19140.1	12
3774	LOC_Os01g15070.1	1
3774	LOC_Os01g15090.1	1
3774	LOC_Os01g68880.1	1
3774	LOC_Os02g24040.1	2
3774	LOC_Os02g55450.1	2
3774	LOC_Os04g56340.1	4
3774	LOC_Os05g32910.1	5
3774	LOC_Os06g24020.1	6
3774	LOC_Os06g40660.1	6
3774	LOC_Os07g06230.1	7
3774	LOC_Os07g06148.1	7
3774	LOC_Os07g06210.1	7
3774	LOC_Os07g06325.1	7
3774	LOC_Os07g10260.1	7
3774	LOC_Os07g12010.1	7
3774	LOC_Os07g20650.1	7
3774	LOC_Os08g01500.1	8
3774	LOC_Os08g14140.1	8
3774	LOC_Os09g26790.1	9
3774	LOC_Os10g02314.1	10
3774	LOC_Os10g05110.1	10
3774	LOC_Os12g05010.1	12
3774	LOC_Os12g05151.1	12
3774	LOC_Os12g08540.1	12
3774	LOC_Os12g37040.1	12
3774	LOC_Os12g37050.1	12
3775	LOC_Os01g04360.1	1
3775	LOC_Os01g04370.1	1
3775	LOC_Os01g04340.1	1
3775	LOC_Os01g04350.1	1
3775	LOC_Os01g04380.1	1
3775	LOC_Os01g08860.1	1
3775	LOC_Os01g40550.1	1
3775	LOC_Os02g03570.1	2
3775	LOC_Os02g10710.1	2
3775	LOC_Os02g12610.1	2
3775	LOC_Os02g48140.1	2
3775	LOC_Os02g54140.1	2
3775	LOC_Os03g06170.1	3
3775	LOC_Os03g14180.1	3
3775	LOC_Os03g16020.1	3

3775	LOC_Os03g16030.1	3
3775	LOC_Os03g15960.1	3
3775	LOC_Os03g16040.1	3
3775	LOC_Os04g36750.1	4
3775	LOC_Os05g51440.1	5
3775	LOC_Os06g14240.1	6
3775	LOC_Os07g33350.1	7
3775	LOC_Os10g07200.1	10
3775	LOC_Os10g07210.1	10
3775	LOC_Os10g30180.1	10
3775	LOC_Os10g30162.1	10
3775	LOC_Os11g13980.1	11
3776	LOC_Os01g41834.1	1
3776	LOC_Os04g01354.1	4
3776	LOC_Os04g23940.1	4
3776	LOC_Os05g12180.1	5
3776	LOC_Os05g12190.1	5
3776	LOC_Os05g12210.1	5
3776	LOC_Os05g12240.1	5
3776	LOC_Os07g11440.1	7
3776	LOC_Os07g17010.1	7
3776	LOC_Os07g22850.1	7
3776	LOC_Os07g31770.1	7
3776	LOC_Os07g31750.1	7
3776	LOC_Os07g34140.1	7
3776	LOC_Os07g34260.1	7
3776	LOC_Os07g34190.1	7
3776	LOC_Os10g07040.1	10
3776	LOC_Os10g08620.1	10
3776	LOC_Os10g08670.1	10
3776	LOC_Os10g08710.1	10
3776	LOC_Os10g09860.1	10
3776	LOC_Os10g34360.1	10
3776	LOC_Os11g32540.1	11
3776	LOC_Os11g32580.1	11
3776	LOC_Os11g32610.1	11
3776	LOC_Os11g32620.1	11
3776	LOC_Os11g32650.1	11
3776	LOC_Os11g35930.1	11
3777	LOC_Os01g01120.1	1
3777	LOC_Os01g55310.1	1
3777	LOC_Os02g07730.1	2
3777	LOC_Os02g57100.1	2
3777	LOC_Os03g16670.1	3
3777	LOC_Os03g36750.1	3
3777	LOC_Os03g49440.1	3
3777	LOC_Os04g41340.1	4
3777	LOC_Os04g47020.1	4
3777	LOC_Os05g07632.1	5
3777	LOC_Os06g01990.1	6
3777	LOC_Os06g45440.1	6
3777	LOC_Os07g44060.1	7

3777	LOC_Os07g46520.1	7
3777	LOC_Os08g07300.1	8
3777	LOC_Os08g14580.1	8
3777	LOC_Os08g37940.1	8
3777	LOC_Os08g42950.1	8
3777	LOC_Os09g08660.1	9
3777	LOC_Os09g22000.1	9
3777	LOC_Os09g24230.1	9
3777	LOC_Os09g39560.1	9
3777	LOC_Os10g41930.1	10
3777	LOC_Os11g19460.1	11
3777	LOC_Os11g29370.1	11
3777	LOC_Os11g41160.1	11
3777	LOC_Os12g31820.1	12
3778	LOC_Os01g06250.1	1
3778	LOC_Os03g05030.1	3
3778	LOC_Os03g17220.1	3
3778	LOC_Os03g28190.1	3
3778	LOC_Os03g59440.1	3
3778	LOC_Os07g01660.1	7
3778	LOC_Os07g01600.1	7
3778	LOC_Os07g01630.1	7
3778	LOC_Os07g01680.1	7
3778	LOC_Os07g44250.1	7
3778	LOC_Os07g44260.1	7
3778	LOC_Os07g44370.1	7
3778	LOC_Os07g44380.1	7
3778	LOC_Os07g44450.1	7
3778	LOC_Os07g44280.1	7
3778	LOC_Os07g44920.1	7
3778	LOC_Os07g44930.1	7
3778	LOC_Os07g01620.1	7
3778	LOC_Os10g25870.1	10
3778	LOC_Os11g07680.1	11
3778	LOC_Os11g07670.1	11
3778	LOC_Os11g10850.1	11
3778	LOC_Os11g10870.1	11
3778	LOC_Os11g10800.1	11
3778	LOC_Os11g27620.1	11
3778	LOC_Os11g42500.1	11
3778	LOC_Os11g42550.1	11
3779	LOC_Os02g21180.1	2
3779	LOC_Os02g26830.1	2
3779	LOC_Os02g45220.1	2
3779	LOC_Os02g54320.1	2
3779	LOC_Os03g37440.1	3
3779	LOC_Os03g39080.1	3
3779	LOC_Os03g45500.1	3
3779	LOC_Os04g04380.1	4
3779	LOC_Os04g08796.1	4
3779	LOC_Os04g10040.1	4
3779	LOC_Os04g20570.1	4

3779	LOC_Os06g31780.1	6
3779	LOC_Os06g36600.1	6
3779	LOC_Os07g05920.1	7
3779	LOC_Os07g16100.1	7
3779	LOC_Os07g17730.1	7
3779	LOC_Os07g35250.1	7
3779	LOC_Os07g36070.1	7
3779	LOC_Os08g10950.1	8
3779	LOC_Os08g14380.1	8
3779	LOC_Os08g17540.1	8
3779	LOC_Os08g17800.1	8
3779	LOC_Os09g26750.1	9
3779	LOC_Os10g25120.1	10
3779	LOC_Os12g25920.1	12
3779	LOC_Os12g27860.1	12
3779	LOC_Os12g43220.1	12
3780	LOC_Os01g08270.1	1
3780	LOC_Os01g55540.1	1
3780	LOC_Os01g65090.1	1
3780	LOC_Os01g53450.1	1
3780	LOC_Os02g14110.1	2
3780	LOC_Os02g19924.1	2
3780	LOC_Os02g19970.1	2
3780	LOC_Os02g47940.1	2
3780	LOC_Os02g55420.1	2
3780	LOC_Os02g56300.1	2
3780	LOC_Os03g08530.1	3
3780	LOC_Os03g09910.1	3
3780	LOC_Os03g18810.1	3
3780	LOC_Os03g51740.1	3
3780	LOC_Os04g48850.1	4
3780	LOC_Os05g10780.1	5
3780	LOC_Os05g25490.1	5
3780	LOC_Os06g03990.1	6
3780	LOC_Os06g35540.1	6
3780	LOC_Os07g01760.1	7
3780	LOC_Os07g42600.1	7
3780	LOC_Os09g26380.1	9
3780	LOC_Os10g25130.1	10
3780	LOC_Os10g25140.1	10
3780	LOC_Os10g34350.1	10
3780	LOC_Os10g41150.1	10
3780	LOC_Os11g35040.1	11
3780	LOC_Os11g42510.1	11
3781	LOC_Os01g53750.1	1
3781	LOC_Os02g04670.1	2
3781	LOC_Os02g53200.1	2
3781	LOC_Os03g12140.1	3
3781	LOC_Os03g25790.1	3
3781	LOC_Os03g27980.1	3
3781	LOC_Os03g45390.1	3
3781	LOC_Os03g46660.1	3

3781	LOC_Os03g57880.1	3
3781	LOC_Os03g62860.1	3
3781	LOC_Os05g45860.1	5
3781	LOC_Os06g39060.1	6
3781	LOC_Os07g07340.1	7
3781	LOC_Os07g32600.1	7
3781	LOC_Os07g35350.1	7
3781	LOC_Os07g35510.1	7
3781	LOC_Os07g35560.1	7
3781	LOC_Os07g35480.1	7
3781	LOC_Os07g35520.1	7
3781	LOC_Os07g38930.1	7
3781	LOC_Os08g12800.1	8
3781	LOC_Os08g14700.1	8
3781	LOC_Os08g23720.1	8
3781	LOC_Os08g41410.1	8
3781	LOC_Os09g09980.1	9
3781	LOC_Os09g32550.1	9
3781	LOC_Os11g36940.1	11
3781	LOC_Os11g47820.1	11
3782	LOC_Os02g03550.1	2
3782	LOC_Os02g17880.1	2
3782	LOC_Os02g17900.1	2
3782	LOC_Os02g46910.1	2
3782	LOC_Os02g57770.1	2
3782	LOC_Os03g01800.1	3
3782	LOC_Os03g13570.1	3
3782	LOC_Os03g63760.1	3
3782	LOC_Os04g51450.1	4
3782	LOC_Os04g51460.1	4
3782	LOC_Os04g51510.1	4
3782	LOC_Os04g51520.1	4
3782	LOC_Os06g13040.1	6
3782	LOC_Os06g22919.1	6
3782	LOC_Os06g48180.1	6
3782	LOC_Os06g48160.1	6
3782	LOC_Os06g48170.1	6
3782	LOC_Os06g48200.1	6
3782	LOC_Os07g29750.1	7
3782	LOC_Os08g13980.1	8
3782	LOC_Os08g13920.1	8
3782	LOC_Os08g14200.1	8
3782	LOC_Os08g14210.1	8
3782	LOC_Os09g23220.1	9
3782	LOC_Os10g02770.1	10
3782	LOC_Os10g39840.1	10
3782	LOC_Os10g42670.1	10
3782	LOC_Os11g33270.1	11
3783	LOC_Os01g23530.1	1
3783	LOC_Os01g42610.1	1
3783	LOC_Os02g02930.1	2
3783	LOC_Os02g26014.1	2

3783	LOC_Os03g22634.1	3
3783	LOC_Os03g24680.1	3
3783	LOC_Os03g24690.1	3
3783	LOC_Os03g24760.1	3
3783	LOC_Os03g24640.1	3
3783	LOC_Os04g01810.1	4
3783	LOC_Os04g26960.1	4
3783	LOC_Os04g26980.1	4
3783	LOC_Os04g27070.1	4
3783	LOC_Os04g27190.1	4
3783	LOC_Os04g27340.1	4
3783	LOC_Os04g27400.1	4
3783	LOC_Os04g27430.1	4
3783	LOC_Os04g27670.1	4
3783	LOC_Os04g27540.1	4
3783	LOC_Os04g27720.1	4
3783	LOC_Os04g27760.1	4
3783	LOC_Os04g27790.1	4
3783	LOC_Os07g11790.1	7
3783	LOC_Os08g04500.1	8
3783	LOC_Os08g07080.1	8
3783	LOC_Os08g07100.1	8
3783	LOC_Os08g07120.1	8
3783	LOC_Os10g34790.1	10
3784	LOC_Os01g08780.1	1
3784	LOC_Os01g42490.1	1
3784	LOC_Os01g51890.1	1
3784	LOC_Os01g58690.1	1
3784	LOC_Os02g51600.1	2
3784	LOC_Os02g27620.1	2
3784	LOC_Os03g06460.1	3
3784	LOC_Os03g07080.1	3
3784	LOC_Os03g13520.1	3
3784	LOC_Os03g24610.1	3
3784	LOC_Os03g46090.1	3
3784	LOC_Os03g42810.1	3
3784	LOC_Os03g57950.1	3
3784	LOC_Os05g02350.1	5
3784	LOC_Os05g41000.1	5
3784	LOC_Os05g32330.1	5
3784	LOC_Os05g45900.1	5
3784	LOC_Os06g11920.1	6
3784	LOC_Os07g07950.1	7
3784	LOC_Os07g45100.1	7
3784	LOC_Os08g32960.1	8
3784	LOC_Os08g41270.1	8
3784	LOC_Os09g23140.1	9
3784	LOC_Os09g32440.1	9
3784	LOC_Os10g26164.1	10
3784	LOC_Os10g27230.1	10
3784	LOC_Os10g28660.1	10
3784	LOC_Os12g18200.1	12

3785	LOC_Os01g08520.1	1
3785	LOC_Os01g41010.1	1
3785	LOC_Os01g52100.1	1
3785	LOC_Os02g07820.1	2
3785	LOC_Os02g37970.1	2
3785	LOC_Os02g46180.1	2
3785	LOC_Os02g46190.1	2
3785	LOC_Os02g46210.1	2
3785	LOC_Os02g51550.1	2
3785	LOC_Os03g08520.1	3
3785	LOC_Os03g46260.1	3
3785	LOC_Os04g49620.1	4
3785	LOC_Os04g49680.1	4
3785	LOC_Os04g49650.1	4
3785	LOC_Os04g49660.1	4
3785	LOC_Os04g49670.1	4
3785	LOC_Os05g08800.1	5
3785	LOC_Os06g03520.1	6
3785	LOC_Os06g05970.1	6
3785	LOC_Os06g11980.1	6
3785	LOC_Os06g14070.1	6
3785	LOC_Os06g50080.1	6
3785	LOC_Os07g42390.1	7
3785	LOC_Os08g31510.1	8
3785	LOC_Os09g20240.1	9
3785	LOC_Os09g26370.1	9
3785	LOC_Os10g28680.1	10
3785	LOC_Os11g43790.1	11
3786	LOC_Os01g07930.1	1
3786	LOC_Os01g39100.1	1
3786	LOC_Os01g42970.1	1
3786	LOC_Os01g45730.1	1
3786	LOC_Os01g61830.1	1
3786	LOC_Os02g06584.1	2
3786	LOC_Os02g58440.1	2
3786	LOC_Os03g02160.1	3
3786	LOC_Os03g18950.1	3
3786	LOC_Os04g35800.1	4
3786	LOC_Os04g41060.1	4
3786	LOC_Os04g56750.1	4
3786	LOC_Os04g57600.1	4
3786	LOC_Os05g08400.1	5
3786	LOC_Os05g41790.1	5
3786	LOC_Os05g50080.1	5
3786	LOC_Os06g32720.1	6
3786	LOC_Os06g49080.1	6
3786	LOC_Os07g04650.1	7
3786	LOC_Os07g04580.1	7
3786	LOC_Os07g39440.1	7
3786	LOC_Os08g04170.1	8
3786	LOC_Os08g06330.1	8
3786	LOC_Os08g38370.1	8

3786	LOC_Os09g13530.1	9
3786	LOC_Os11g28270.1	11
3786	LOC_Os12g03554.1	12
3786	LOC_Os12g18120.1	12
3786	LOC_Os12g21700.1	12
3787	LOC_Os01g13560.1	1
3787	LOC_Os01g26120.1	1
3787	LOC_Os01g52610.1	1
3787	LOC_Os01g62850.1	1
3787	LOC_Os02g08110.1	2
3787	LOC_Os02g51010.1	2
3787	LOC_Os03g10870.1	3
3787	LOC_Os03g10030.1	3
3787	LOC_Os03g18680.1	3
3787	LOC_Os03g55870.1	3
3787	LOC_Os03g60250.1	3
3787	LOC_Os04g21320.1	4
3787	LOC_Os04g38690.1	4
3787	LOC_Os04g58760.1	4
3787	LOC_Os05g15630.1	5
3787	LOC_Os05g27790.1	5
3787	LOC_Os05g38250.1	5
3787	LOC_Os06g12500.1	6
3787	LOC_Os06g44610.1	6
3787	LOC_Os07g26110.1	7
3787	LOC_Os07g49200.1	7
3787	LOC_Os08g01160.1	8
3787	LOC_Os08g42430.1	8
3787	LOC_Os09g07480.1	9
3787	LOC_Os10g20250.1	10
3787	LOC_Os11g34730.1	11
3787	LOC_Os12g32970.1	12
3787	LOC_Os12g38100.1	12
3787	LOC_Os12g41690.1	12
3788	LOC_Os01g08560.1	1
3788	LOC_Os01g33360.1	1
3788	LOC_Os01g49430.1	1
3788	LOC_Os01g62290.1	1
3788	LOC_Os02g02410.1	2
3788	LOC_Os02g48110.1	2
3788	LOC_Os02g53420.1	2
3788	LOC_Os03g02260.1	3
3788	LOC_Os03g11910.1	3
3788	LOC_Os03g16880.1	3
3788	LOC_Os03g16920.1	3
3788	LOC_Os03g16860.1	3
3788	LOC_Os03g50250.1	3
3788	LOC_Os03g60620.1	3
3788	LOC_Os05g08840.1	5
3788	LOC_Os05g23740.1	5
3788	LOC_Os05g35400.1	5
3788	LOC_Os05g30480.1	5

3788	LOC_Os05g38530.1	5
3788	LOC_Os05g51360.1	5
3788	LOC_Os06g10990.1	6
3788	LOC_Os06g46600.1	6
3788	LOC_Os08g09770.1	8
3788	LOC_Os09g31486.1	9
3788	LOC_Os11g08440.1	11
3788	LOC_Os11g08460.1	11
3788	LOC_Os11g08470.1	11
3788	LOC_Os11g47760.1	11
3788	LOC_Os12g05760.1	12
3788	LOC_Os12g14070.1	12
3789	LOC_Os01g24810.1	1
3789	LOC_Os01g36294.1	1
3789	LOC_Os02g12540.1	2
3789	LOC_Os02g57290.1	2
3789	LOC_Os03g39690.1	3
3789	LOC_Os04g03870.1	4
3789	LOC_Os04g03890.1	4
3789	LOC_Os04g08824.1	4
3789	LOC_Os04g08828.1	4
3789	LOC_Os05g25640.1	5
3789	LOC_Os05g30890.1	5
3789	LOC_Os06g22340.1	6
3789	LOC_Os06g37224.1	6
3789	LOC_Os06g46680.1	6
3789	LOC_Os06g37364.1	6
3789	LOC_Os07g19210.1	7
3789	LOC_Os08g01470.1	8
3789	LOC_Os08g05610.1	8
3789	LOC_Os08g39640.1	8
3789	LOC_Os09g27510.1	9
3789	LOC_Os09g36070.1	9
3789	LOC_Os09g36080.1	9
3789	LOC_Os10g09160.1	10
3789	LOC_Os10g21050.1	10
3789	LOC_Os10g37034.1	10
3789	LOC_Os10g37070.1	10
3789	LOC_Os10g37100.1	10
3789	LOC_Os11g28060.1	11
3789	LOC_Os11g29720.1	11
3789	LOC_Os12g39310.1	12
3790	LOC_Os01g43090.1	1
3790	LOC_Os01g62860.1	1
3790	LOC_Os01g62870.1	1
3790	LOC_Os01g62880.1	1
3790	LOC_Os02g57240.1	2
3790	LOC_Os02g03100.1	2
3790	LOC_Os03g41510.1	3
3790	LOC_Os03g13390.1	3
3790	LOC_Os04g08550.1	4
3790	LOC_Os04g26910.1	4

3790	LOC_Os04g26920.1	4
3790	LOC_Os04g26870.1	4
3790	LOC_Os04g27060.1	4
3790	LOC_Os04g37480.1	4
3790	LOC_Os04g37470.1	4
3790	LOC_Os04g37490.1	4
3790	LOC_Os05g38220.1	5
3790	LOC_Os05g39690.1	5
3790	LOC_Os05g38230.1	5
3790	LOC_Os07g04990.1	7
3790	LOC_Os07g05000.1	7
3790	LOC_Os09g39390.1	9
3790	LOC_Os10g02390.1	10
3790	LOC_Os10g02490.1	10
3790	LOC_Os10g02380.1	10
3790	LOC_Os10g02480.1	10
3790	LOC_Os10g28320.1	10
3790	LOC_Os10g37330.1	10
3790	LOC_Os11g42540.1	11
3790	LOC_Os12g29760.1	12
3791	LOC_Os01g21350.1	1
3791	LOC_Os01g24390.1	1
3791	LOC_Os01g27560.1	1
3791	LOC_Os01g31120.1	1
3791	LOC_Os01g42560.1	1
3791	LOC_Os01g42580.1	1
3791	LOC_Os02g29270.1	2
3791	LOC_Os02g34330.1	2
3791	LOC_Os03g33370.1	3
3791	LOC_Os03g33930.1	3
3791	LOC_Os03g34320.1	3
3791	LOC_Os03g36310.1	3
3791	LOC_Os03g44460.1	3
3791	LOC_Os04g20870.1	4
3791	LOC_Os04g26280.1	4
3791	LOC_Os05g02290.1	5
3791	LOC_Os05g13400.1	5
3791	LOC_Os05g13410.1	5
3791	LOC_Os05g44840.1	5
3791	LOC_Os06g11470.1	6
3791	LOC_Os06g15860.1	6
3791	LOC_Os06g25870.1	6
3791	LOC_Os06g29640.1	6
3791	LOC_Os06g49900.1	6
3791	LOC_Os07g23230.1	7
3791	LOC_Os07g39150.1	7
3791	LOC_Os10g01590.1	10
3791	LOC_Os10g20670.1	10
3791	LOC_Os11g02920.1	11
3791	LOC_Os12g12440.1	12
3792	LOC_Os01g11020.1	1
3792	LOC_Os01g16070.1	1

3792	LOC_Os01g29690.1	1
3792	LOC_Os01g32430.1	1
3792	LOC_Os01g45410.1	1
3792	LOC_Os01g52140.1	1
3792	LOC_Os01g59240.1	1
3792	LOC_Os01g73030.1	1
3792	LOC_Os02g29940.1	2
3792	LOC_Os03g33450.1	3
3792	LOC_Os03g38040.1	3
3792	LOC_Os03g38170.1	3
3792	LOC_Os03g41260.1	3
3792	LOC_Os04g03510.1	4
3792	LOC_Os04g16020.1	4
3792	LOC_Os05g04250.1	5
3792	LOC_Os05g27440.1	5
3792	LOC_Os05g12690.1	5
3792	LOC_Os05g32020.1	5
3792	LOC_Os06g03350.1	6
3792	LOC_Os07g16280.1	7
3792	LOC_Os07g24950.1	7
3792	LOC_Os08g10680.1	8
3792	LOC_Os08g16730.1	8
3792	LOC_Os08g17620.1	8
3792	LOC_Os09g14050.1	9
3792	LOC_Os10g18234.1	10
3792	LOC_Os11g01230.1	11
3792	LOC_Os11g22050.1	11
3792	LOC_Os12g19230.1	12
3793	LOC_Os01g37750.1	1
3793	LOC_Os01g49710.1	1
3793	LOC_Os01g49720.1	1
3793	LOC_Os01g72160.1	1
3793	LOC_Os01g72170.1	1
3793	LOC_Os01g72120.1	1
3793	LOC_Os01g72130.1	1
3793	LOC_Os03g57200.1	3
3793	LOC_Os05g34150.1	5
3793	LOC_Os07g28480.1	7
3793	LOC_Os09g20220.1	9
3793	LOC_Os09g29200.1	9
3793	LOC_Os10g34020.1	10
3793	LOC_Os10g38140.1	10
3793	LOC_Os10g38350.1	10
3793	LOC_Os10g38360.1	10
3793	LOC_Os10g38590.1	10
3793	LOC_Os10g38710.1	10
3793	LOC_Os10g38740.1	10
3793	LOC_Os10g38780.1	10
3793	LOC_Os10g38160.1	10
3793	LOC_Os10g38470.1	10
3793	LOC_Os10g38540.1	10
3793	LOC_Os10g38580.1	10

3793	LOC_Os10g38610.1	10
3793	LOC_Os10g38640.1	10
3793	LOC_Os10g38660.1	10
3793	LOC_Os10g38670.1	10
3793	LOC_Os10g38690.1	10
3793	LOC_Os10g38700.1	10
3793	LOC_Os10g38630.1	10
3794	LOC_Os01g62190.1	1
3794	LOC_Os01g66570.1	1
3794	LOC_Os02g01090.1	2
3794	LOC_Os02g34680.1	2
3794	LOC_Os02g44120.1	2
3794	LOC_Os02g02424.1	2
3794	LOC_Os03g05480.1	3
3794	LOC_Os03g13400.1	3
3794	LOC_Os03g31240.1	3
3794	LOC_Os03g40710.1	3
3794	LOC_Os03g49132.1	3
3794	LOC_Os03g05690.1	3
3794	LOC_Os04g08600.1	4
3794	LOC_Os05g01550.1	5
3794	LOC_Os05g03020.1	5
3794	LOC_Os05g38600.1	5
3794	LOC_Os05g51830.1	5
3794	LOC_Os06g07020.1	6
3794	LOC_Os06g20020.1	6
3794	LOC_Os06g51140.1	6
3794	LOC_Os07g01180.1	7
3794	LOC_Os07g23450.1	7
3794	LOC_Os07g40780.1	7
3794	LOC_Os07g40950.1	7
3794	LOC_Os08g37904.1	8
3794	LOC_Os08g37920.1	8
3794	LOC_Os09g03500.1	9
3794	LOC_Os09g26210.1	9
3794	LOC_Os09g39660.1	9
3794	LOC_Os11g47630.1	11
3794	LOC_Os12g38940.1	12
3795	LOC_Os01g10504.1	1
3795	LOC_Os01g52680.1	1
3795	LOC_Os01g66030.1	1
3795	LOC_Os01g66290.1	1
3795	LOC_Os02g07430.1	2
3795	LOC_Os02g36924.1	2
3795	LOC_Os02g45770.1	2
3795	LOC_Os02g49840.1	2
3795	LOC_Os02g52340.1	2
3795	LOC_Os03g08754.1	3
3795	LOC_Os03g11614.1	3
3795	LOC_Os03g54160.1	3
3795	LOC_Os03g54170.1	3
3795	LOC_Os04g23910.1	4

3795	LOC_Os04g49150.1	4
3795	LOC_Os04g52410.1	4
3795	LOC_Os05g34940.1	5
3795	LOC_Os05g11414.1	5
3795	LOC_Os06g06750.1	6
3795	LOC_Os06g11330.1	6
3795	LOC_Os06g45650.1	6
3795	LOC_Os06g49840.1	6
3795	LOC_Os07g41370.1	7
3795	LOC_Os07g01820.1	7
3795	LOC_Os08g02070.1	8
3795	LOC_Os08g33488.1	8
3795	LOC_Os08g41950.1	8
3795	LOC_Os09g32948.1	9
3795	LOC_Os10g39130.1	10
3795	LOC_Os12g10520.1	12
3795	LOC_Os12g10540.1	12
3796	LOC_Os01g25600.1	1
3796	LOC_Os01g43030.1	1
3796	LOC_Os01g67920.1	1
3796	LOC_Os01g68680.1	1
3796	LOC_Os02g01960.1	2
3796	LOC_Os02g10940.1	2
3796	LOC_Os02g28830.1	2
3796	LOC_Os02g34830.1	2
3796	LOC_Os02g47880.1	2
3796	LOC_Os02g01030.1	2
3796	LOC_Os03g07260.1	3
3796	LOC_Os03g13370.1	3
3796	LOC_Os03g19560.1	3
3796	LOC_Os03g22140.1	3
3796	LOC_Os03g53910.1	3
3796	LOC_Os04g52580.1	4
3796	LOC_Os04g55230.1	4
3796	LOC_Os04g57780.1	4
3796	LOC_Os05g05720.1	5
3796	LOC_Os05g01460.1	5
3796	LOC_Os06g41750.1	6
3796	LOC_Os06g07474.1	6
3796	LOC_Os07g02300.1	7
3796	LOC_Os07g08120.1	7
3796	LOC_Os07g23990.1	7
3796	LOC_Os07g27180.1	7
3796	LOC_Os07g29360.1	7
3796	LOC_Os08g39080.1	8
3796	LOC_Os10g40050.1	10
3796	LOC_Os11g16540.1	11
3796	LOC_Os12g41190.1	12
3797	LOC_Os01g04790.1	1
3797	LOC_Os01g18760.1	1
3797	LOC_Os01g33700.1	1
3797	LOC_Os02g40850.1	2

3797	LOC_Os02g49500.1	2
3797	LOC_Os04g05640.1	4
3797	LOC_Os04g06400.1	4
3797	LOC_Os04g08530.1	4
3797	LOC_Os04g13400.1	4
3797	LOC_Os04g14980.1	4
3797	LOC_Os04g26140.1	4
3797	LOC_Os05g15400.1	5
3797	LOC_Os05g45600.1	5
3797	LOC_Os06g07110.1	6
3797	LOC_Os06g36980.1	6
3797	LOC_Os06g40540.1	6
3797	LOC_Os06g42900.1	6
3797	LOC_Os06g48490.1	6
3797	LOC_Os07g12740.1	7
3797	LOC_Os07g24280.1	7
3797	LOC_Os07g48210.1	7
3797	LOC_Os08g21710.1	8
3797	LOC_Os09g01800.1	9
3797	LOC_Os09g11350.1	9
3797	LOC_Os09g19990.1	9
3797	LOC_Os11g18070.1	11
3797	LOC_Os11g47250.1	11
3797	LOC_Os12g11670.1	12
3797	LOC_Os12g14490.1	12
3797	LOC_Os12g33670.1	12
3797	LOC_Os12g36380.1	12
3798	LOC_Os01g03440.1	1
3798	LOC_Os01g14290.1	1
3798	LOC_Os01g35410.1	1
3798	LOC_Os01g39940.1	1
3798	LOC_Os01g56250.1	1
3798	LOC_Os01g56340.1	1
3798	LOC_Os01g57190.1	1
3798	LOC_Os02g05110.1	2
3798	LOC_Os02g41750.1	2
3798	LOC_Os02g54950.1	2
3798	LOC_Os03g26380.1	3
3798	LOC_Os03g60830.1	3
3798	LOC_Os04g04220.1	4
3798	LOC_Os05g06850.1	5
3798	LOC_Os05g07970.1	5
3798	LOC_Os05g16500.1	5
3798	LOC_Os05g25600.1	5
3798	LOC_Os05g42030.1	5
3798	LOC_Os06g12640.1	6
3798	LOC_Os06g37890.1	6
3798	LOC_Os07g36970.1	7
3798	LOC_Os08g36670.1	8
3798	LOC_Os08g38050.1	8
3798	LOC_Os09g19870.1	9
3798	LOC_Os09g22380.1	9

3798	LOC_Os10g30620.1	10
3798	LOC_Os11g09120.1	11
3798	LOC_Os11g46260.1	11
3798	LOC_Os12g02270.1	12
3798	LOC_Os12g31940.1	12
3798	LOC_Os12g39140.1	12
3799	LOC_Os01g52750.1	1
3799	LOC_Os01g56320.1	1
3799	LOC_Os01g58240.1	1
3799	LOC_Os01g58270.1	1
3799	LOC_Os01g64850.1	1
3799	LOC_Os01g64860.1	1
3799	LOC_Os01g58290.1	1
3799	LOC_Os02g10520.1	2
3799	LOC_Os02g17090.1	2
3799	LOC_Os02g44590.1	2
3799	LOC_Os02g53850.1	2
3799	LOC_Os02g53860.1	2
3799	LOC_Os03g02750.1	3
3799	LOC_Os03g04950.1	3
3799	LOC_Os03g13930.1	3
3799	LOC_Os03g40830.1	3
3799	LOC_Os03g55350.1	3
3799	LOC_Os04g02980.1	4
3799	LOC_Os04g35140.1	4
3799	LOC_Os04g47150.1	4
3799	LOC_Os04g47160.1	4
3799	LOC_Os05g36010.1	5
3799	LOC_Os05g30580.1	5
3799	LOC_Os07g39020.1	7
3799	LOC_Os07g48650.1	7
3799	LOC_Os08g23740.1	8
3799	LOC_Os08g35090.1	8
3799	LOC_Os09g26920.1	9
3799	LOC_Os09g30458.1	9
3799	LOC_Os09g36110.1	9
3799	LOC_Os10g25450.1	10
3799	LOC_Os10g38080.1	10
3800	LOC_Os01g14920.1	1
3800	LOC_Os01g51710.1	1
3800	LOC_Os02g52460.1	2
3800	LOC_Os03g39040.1	3
3800	LOC_Os03g39880.1	3
3800	LOC_Os03g60600.1	3
3800	LOC_Os04g08800.1	4
3800	LOC_Os04g20610.1	4
3800	LOC_Os04g20320.1	4
3800	LOC_Os04g22050.1	4
3800	LOC_Os04g54530.1	4
3800	LOC_Os04g46920.1	4
3800	LOC_Os05g15200.1	5
3800	LOC_Os05g40490.1	5

3800	LOC_Os06g11950.1	6
3800	LOC_Os06g17540.1	6
3800	LOC_Os06g37520.1	6
3800	LOC_Os06g49510.1	6
3800	LOC_Os08g32550.1	8
3800	LOC_Os08g39540.1	8
3800	LOC_Os09g34980.1	9
3800	LOC_Os10g28150.1	10
3800	LOC_Os10g39820.1	10
3800	LOC_Os11g09310.1	11
3800	LOC_Os11g09540.1	11
3800	LOC_Os11g14360.1	11
3800	LOC_Os11g25750.1	11
3800	LOC_Os11g32720.1	11
3800	LOC_Os11g34820.1	11
3800	LOC_Os11g36480.1	11
3800	LOC_Os12g18260.1	12
3800	LOC_Os12g38040.1	12
3801	LOC_Os01g07376.1	1
3801	LOC_Os01g68480.1	1
3801	LOC_Os02g01010.1	2
3801	LOC_Os02g34530.1	2
3801	LOC_Os02g34940.1	2
3801	LOC_Os02g35900.1	2
3801	LOC_Os02g42700.1	2
3801	LOC_Os03g17860.1	3
3801	LOC_Os03g21000.1	3
3801	LOC_Os03g55820.1	3
3801	LOC_Os03g58630.1	3
3801	LOC_Os04g35150.1	4
3801	LOC_Os04g35290.1	4
3801	LOC_Os04g35600.1	4
3801	LOC_Os04g44830.1	4
3801	LOC_Os04g57930.1	4
3801	LOC_Os04g53740.1	4
3801	LOC_Os05g07690.1	5
3801	LOC_Os05g11090.1	5
3801	LOC_Os05g40190.1	5
3801	LOC_Os05g47930.1	5
3801	LOC_Os06g06790.1	6
3801	LOC_Os06g45510.1	6
3801	LOC_Os07g08840.1	7
3801	LOC_Os07g09310.1	7
3801	LOC_Os07g29410.1	7
3801	LOC_Os07g48510.1	7
3801	LOC_Os08g29110.1	8
3801	LOC_Os09g27830.1	9
3801	LOC_Os09g38670.1	9
3801	LOC_Os11g09280.1	11
3801	LOC_Os12g08730.1	12
3801	LOC_Os12g18220.1	12
3802	LOC_Os01g51570.1	1

3802	LOC_Os01g58730.1	1
3802	LOC_Os01g64170.1	1
3802	LOC_Os01g71350.1	1
3802	LOC_Os01g71380.1	1
3802	LOC_Os01g71400.1	1
3802	LOC_Os01g71340.1	1
3802	LOC_Os01g71410.1	1
3802	LOC_Os01g71474.1	1
3802	LOC_Os01g71680.1	1
3802	LOC_Os01g71650.1	1
3802	LOC_Os01g71670.1	1
3802	LOC_Os01g71830.1	1
3802	LOC_Os01g71860.1	1
3802	LOC_Os01g71810.1	1
3802	LOC_Os01g71820.1	1
3802	LOC_Os02g10660.1	2
3802	LOC_Os02g33000.1	2
3802	LOC_Os03g12620.1	3
3802	LOC_Os03g14210.1	3
3802	LOC_Os03g22530.1	3
3802	LOC_Os03g40330.1	3
3802	LOC_Os03g51240.1	3
3802	LOC_Os03g56130.1	3
3802	LOC_Os04g33640.1	4
3802	LOC_Os05g31140.1	5
3802	LOC_Os05g37130.1	5
3802	LOC_Os05g41610.1	5
3802	LOC_Os06g04080.1	6
3802	LOC_Os06g34020.1	6
3802	LOC_Os06g40490.1	6
3802	LOC_Os09g36280.1	9
3802	LOC_Os10g07290.1	10
3803	LOC_Os01g70750.1	1
3803	LOC_Os02g33370.1	2
3803	LOC_Os02g50610.1	2
3803	LOC_Os03g33730.1	3
3803	LOC_Os03g33740.1	3
3803	LOC_Os04g07240.1	4
3803	LOC_Os04g10130.1	4
3803	LOC_Os04g13650.1	4
3803	LOC_Os04g17210.1	4
3803	LOC_Os04g54780.1	4
3803	LOC_Os05g08130.1	5
3803	LOC_Os06g26130.1	6
3803	LOC_Os06g35270.1	6
3803	LOC_Os07g20660.1	7
3803	LOC_Os08g17490.1	8
3803	LOC_Os08g19710.1	8
3803	LOC_Os08g22510.1	8
3803	LOC_Os08g32190.1	8
3803	LOC_Os09g03100.1	9
3803	LOC_Os10g01940.1	10

3803	LOC_Os11g03040.1	11
3803	LOC_Os11g02910.1	11
3803	LOC_Os11g15070.1	11
3803	LOC_Os11g27920.1	11
3803	LOC_Os11g31300.1	11
3803	LOC_Os12g01340.1	12
3803	LOC_Os12g10440.1	12
3803	LOC_Os12g11200.1	12
3803	LOC_Os12g11310.1	12
3803	LOC_Os12g14280.1	12
3803	LOC_Os12g22000.1	12
3803	LOC_Os12g22520.1	12
3803	LOC_Os12g33380.1	12
3804	LOC_Os01g32364.1	1
3804	LOC_Os01g59819.1	1
3804	LOC_Os01g59840.1	1
3804	LOC_Os01g67220.1	1
3804	LOC_Os01g70520.1	1
3804	LOC_Os03g11420.1	3
3804	LOC_Os03g20710.1	3
3804	LOC_Os03g49600.1	3
3804	LOC_Os03g49610.1	3
3804	LOC_Os04g39840.1	4
3804	LOC_Os04g39864.1	4
3804	LOC_Os04g39880.1	4
3804	LOC_Os04g39900.1	4
3804	LOC_Os04g43360.1	4
3804	LOC_Os04g43390.1	4
3804	LOC_Os04g43410.1	4
3804	LOC_Os04g39814.1	4
3804	LOC_Os05g30350.1	5
3804	LOC_Os05g30250.1	5
3804	LOC_Os05g30300.1	5
3804	LOC_Os05g30390.1	5
3804	LOC_Os05g30280.1	5
3804	LOC_Os06g21570.1	6
3804	LOC_Os06g46940.1	6
3804	LOC_Os07g46280.1	7
3804	LOC_Os08g39860.1	8
3804	LOC_Os08g39870.1	8
3804	LOC_Os09g31410.1	9
3804	LOC_Os09g31430.1	9
3804	LOC_Os09g33710.1	9
3804	LOC_Os09g33680.1	9
3804	LOC_Os10g17650.1	10
3804	LOC_Os11g08120.1	11
3804	LOC_Os12g23170.1	12
3805	LOC_Os01g09830.1	1
3805	LOC_Os01g13480.1	1
3805	LOC_Os01g13950.1	1
3805	LOC_Os01g26912.1	1
3805	LOC_Os01g27140.1	1

3805	LOC_Os01g47760.1	1
3805	LOC_Os01g61350.1	1
3805	LOC_Os01g70990.1	1
3805	LOC_Os02g01200.1	2
3805	LOC_Os02g30850.1	2
3805	LOC_Os02g43180.1	2
3805	LOC_Os02g51370.1	2
3805	LOC_Os03g07470.1	3
3805	LOC_Os03g44650.1	3
3805	LOC_Os04g32300.1	4
3805	LOC_Os04g33680.1	4
3805	LOC_Os04g42930.1	4
3805	LOC_Os04g54860.1	4
3805	LOC_Os05g05730.1	5
3805	LOC_Os05g10930.1	5
3805	LOC_Os05g39450.1	5
3805	LOC_Os05g48930.1	5
3805	LOC_Os06g12190.1	6
3805	LOC_Os07g05630.1	7
3805	LOC_Os07g06600.1	7
3805	LOC_Os07g46570.1	7
3805	LOC_Os08g07450.1	8
3805	LOC_Os08g45140.1	8
3805	LOC_Os11g43520.1	11
3805	LOC_Os11g43530.1	11
3805	LOC_Os11g43580.1	11
3805	LOC_Os11g43550.1	11
3805	LOC_Os12g35330.1	12
3805	LOC_Os12g35340.1	12
3806	LOC_Os01g19750.1	1
3806	LOC_Os01g43220.1	1
3806	LOC_Os01g47070.1	1
3806	LOC_Os01g49320.1	1
3806	LOC_Os01g64100.1	1
3806	LOC_Os01g64110.1	1
3806	LOC_Os04g27980.1	4
3806	LOC_Os04g30770.1	4
3806	LOC_Os05g15770.1	5
3806	LOC_Os05g15880.1	5
3806	LOC_Os05g15920.1	5
3806	LOC_Os05g15850.1	5
3806	LOC_Os06g25010.1	6
3806	LOC_Os07g19040.1	7
3806	LOC_Os07g23850.1	7
3806	LOC_Os07g43820.1	7
3806	LOC_Os08g40680.1	8
3806	LOC_Os08g40690.1	8
3806	LOC_Os08g40740.1	8
3806	LOC_Os10g28050.1	10
3806	LOC_Os10g28080.1	10
3806	LOC_Os10g28120.1	10
3806	LOC_Os11g27400.1	11

3806	LOC_Os11g47500.1	11
3806	LOC_Os11g47510.1	11
3806	LOC_Os11g47560.1	11
3806	LOC_Os11g47570.1	11
3806	LOC_Os11g47580.1	11
3806	LOC_Os11g47590.1	11
3806	LOC_Os11g47520.1	11
3806	LOC_Os11g47530.1	11
3806	LOC_Os11g47550.1	11
3806	LOC_Os11g47600.1	11
3806	LOC_Os11g47610.1	11
3807	LOC_Os01g33060.1	1
3807	LOC_Os01g40250.1	1
3807	LOC_Os01g56590.1	1
3807	LOC_Os01g60440.1	1
3807	LOC_Os01g60040.1	1
3807	LOC_Os02g07120.1	2
3807	LOC_Os02g07340.1	2
3807	LOC_Os02g40430.1	2
3807	LOC_Os02g54110.1	2
3807	LOC_Os02g57120.1	2
3807	LOC_Os03g05330.1	3
3807	LOC_Os03g12300.1	3
3807	LOC_Os03g15540.1	3
3807	LOC_Os03g16460.1	3
3807	LOC_Os03g17340.1	3
3807	LOC_Os03g42040.1	3
3807	LOC_Os03g49420.1	3
3807	LOC_Os03g51140.1	3
3807	LOC_Os04g42840.1	4
3807	LOC_Os04g46310.1	4
3807	LOC_Os05g09620.1	5
3807	LOC_Os06g17840.1	6
3807	LOC_Os06g19730.1	6
3807	LOC_Os06g30894.1	6
3807	LOC_Os07g38760.1	7
3807	LOC_Os07g46540.1	7
3807	LOC_Os07g49320.1	7
3807	LOC_Os08g05670.1	8
3807	LOC_Os08g07290.1	8
3807	LOC_Os09g07510.1	9
3807	LOC_Os10g36490.1	10
3807	LOC_Os11g04220.1	11
3807	LOC_Os12g04030.1	12
3807	LOC_Os12g36170.1	12
3808	LOC_Os01g24750.1	1
3808	LOC_Os01g49690.1	1
3808	LOC_Os01g66920.1	1
3808	LOC_Os01g71420.1	1
3808	LOC_Os02g12580.1	2
3808	LOC_Os02g16550.1	2
3808	LOC_Os02g57450.1	2

3808	LOC_Os03g07150.1	3
3808	LOC_Os03g13540.1	3
3808	LOC_Os03g16110.1	3
3808	LOC_Os03g27950.1	3
3808	LOC_Os03g51540.1	3
3808	LOC_Os03g59060.1	3
3808	LOC_Os03g15430.1	3
3808	LOC_Os04g33470.1	4
3808	LOC_Os06g06880.1	6
3808	LOC_Os06g08390.1	6
3808	LOC_Os06g37660.1	6
3808	LOC_Os06g48570.1	6
3808	LOC_Os07g01540.1	7
3808	LOC_Os07g02090.1	7
3808	LOC_Os07g04210.1	7
3808	LOC_Os07g17280.1	7
3808	LOC_Os07g48840.1	7
3808	LOC_Os08g35440.1	8
3808	LOC_Os08g40200.1	8
3808	LOC_Os09g11230.1	9
3808	LOC_Os09g36290.1	9
3808	LOC_Os10g02750.1	10
3808	LOC_Os10g25430.1	10
3808	LOC_Os10g27050.1	10
3808	LOC_Os10g35060.1	10
3808	LOC_Os11g15570.1	11
3808	LOC_Os11g34710.1	11
3808	LOC_Os11g34720.1	11
3809	LOC_Os01g03890.1	1
3809	LOC_Os01g07480.1	1
3809	LOC_Os01g14030.1	1
3809	LOC_Os01g32770.1	1
3809	LOC_Os01g39040.1	1
3809	LOC_Os01g39070.1	1
3809	LOC_Os01g39220.1	1
3809	LOC_Os01g39150.1	1
3809	LOC_Os01g39160.1	1
3809	LOC_Os01g39180.1	1
3809	LOC_Os01g56530.1	1
3809	LOC_Os01g60960.1	1
3809	LOC_Os01g66590.1	1
3809	LOC_Os02g48270.1	2
3809	LOC_Os02g57490.1	2
3809	LOC_Os03g05500.1	3
3809	LOC_Os03g05510.1	3
3809	LOC_Os03g14270.1	3
3809	LOC_Os03g17810.1	3
3809	LOC_Os03g33090.1	3
3809	LOC_Os03g41600.1	3
3809	LOC_Os03g41330.1	3
3809	LOC_Os03g45750.1	3
3809	LOC_Os03g57670.1	3

3809	LOC_Os05g03160.1	5
3809	LOC_Os05g07270.1	5
3809	LOC_Os05g27980.1	5
3809	LOC_Os05g34450.1	5
3809	LOC_Os07g40000.1	7
3809	LOC_Os08g31080.1	8
3809	LOC_Os08g44940.1	8
3809	LOC_Os09g19950.1	9
3809	LOC_Os10g07510.1	10
3809	LOC_Os11g01550.1	11
3809	LOC_Os12g01550.1	12
3810	LOC_Os01g18030.1	1
3810	LOC_Os02g24560.1	2
3810	LOC_Os03g20060.1	3
3810	LOC_Os03g28380.1	3
3810	LOC_Os03g32560.1	3
3810	LOC_Os03g34150.1	3
3810	LOC_Os03g40580.1	3
3810	LOC_Os04g03290.1	4
3810	LOC_Os04g19860.1	4
3810	LOC_Os04g26560.1	4
3810	LOC_Os04g27640.1	4
3810	LOC_Os04g29200.1	4
3810	LOC_Os04g29530.1	4
3810	LOC_Os05g10710.1	5
3810	LOC_Os05g14610.1	5
3810	LOC_Os05g17650.1	5
3810	LOC_Os05g19990.1	5
3810	LOC_Os06g20670.1	6
3810	LOC_Os07g14400.1	7
3810	LOC_Os07g15050.1	7
3810	LOC_Os08g21790.1	8
3810	LOC_Os08g22320.1	8
3810	LOC_Os08g24130.1	8
3810	LOC_Os09g06140.1	9
3810	LOC_Os09g04820.1	9
3810	LOC_Os09g14860.1	9
3810	LOC_Os10g01870.1	10
3810	LOC_Os10g16470.1	10
3810	LOC_Os10g22000.1	10
3810	LOC_Os10g25880.1	10
3810	LOC_Os11g14750.1	11
3810	LOC_Os11g14840.1	11
3810	LOC_Os11g29890.1	11
3810	LOC_Os12g14880.1	12
3810	LOC_Os12g20000.1	12
3811	LOC_Os01g02190.1	1
3811	LOC_Os01g08660.1	1
3811	LOC_Os01g10530.1	1
3811	LOC_Os01g10600.1	1
3811	LOC_Os01g13120.1	1
3811	LOC_Os01g13130.1	1

3811	LOC_Os01g74450.1	1
3811	LOC_Os02g13870.1	2
3811	LOC_Os02g41860.1	2
3811	LOC_Os02g44080.1	2
3811	LOC_Os02g44630.1	2
3811	LOC_Os02g51110.1	2
3811	LOC_Os02g57720.1	2
3811	LOC_Os03g05290.1	3
3811	LOC_Os03g64330.1	3
3811	LOC_Os04g16450.1	4
3811	LOC_Os04g44060.1	4
3811	LOC_Os04g44570.1	4
3811	LOC_Os04g46490.1	4
3811	LOC_Os04g47220.1	4
3811	LOC_Os05g11560.1	5
3811	LOC_Os05g14240.1	5
3811	LOC_Os06g12310.1	6
3811	LOC_Os06g22960.1	6
3811	LOC_Os06g35930.1	6
3811	LOC_Os07g26690.1	7
3811	LOC_Os07g26630.1	7
3811	LOC_Os07g26640.1	7
3811	LOC_Os08g05580.1	8
3811	LOC_Os08g05590.1	8
3811	LOC_Os08g05600.1	8
3811	LOC_Os09g36930.1	9
3811	LOC_Os10g34000.1	10
3811	LOC_Os10g35050.1	10
3811	LOC_Os10g36924.1	10
3811	LOC_Os12g10280.1	12
3812	LOC_Os01g56930.1	1
3812	LOC_Os01g64830.1	1
3812	LOC_Os01g72510.1	1
3812	LOC_Os02g49800.1	2
3812	LOC_Os02g51540.1	2
3812	LOC_Os03g20210.1	3
3812	LOC_Os03g20230.1	3
3812	LOC_Os03g20260.1	3
3812	LOC_Os04g15790.1	4
3812	LOC_Os04g24750.1	4
3812	LOC_Os04g26790.1	4
3812	LOC_Os04g26834.1	4
3812	LOC_Os04g58840.1	4
3812	LOC_Os05g31170.1	5
3812	LOC_Os05g43550.1	5
3812	LOC_Os05g51350.1	5
3812	LOC_Os06g10670.1	6
3812	LOC_Os06g11010.1	6
3812	LOC_Os06g15760.1	6
3812	LOC_Os06g50390.1	6
3812	LOC_Os07g34920.1	7
3812	LOC_Os07g40260.1	7

3812	LOC_Os07g46480.1	7
3812	LOC_Os08g10670.1	8
3812	LOC_Os09g37012.1	9
3812	LOC_Os10g39310.1	10
3812	LOC_Os10g39320.1	10
3812	LOC_Os10g39330.1	10
3812	LOC_Os10g39340.1	10
3812	LOC_Os10g39360.1	10
3812	LOC_Os10g39390.1	10
3812	LOC_Os10g39270.1	10
3812	LOC_Os11g08100.1	11
3812	LOC_Os11g08200.1	11
3812	LOC_Os12g05750.1	12
3812	LOC_Os12g39360.1	12
3812	LOC_Os12g07780.1	12
3813	LOC_Os01g17050.1	1
3813	LOC_Os01g54400.1	1
3813	LOC_Os01g59410.1	1
3813	LOC_Os02g07690.1	2
3813	LOC_Os02g15280.1	2
3813	LOC_Os02g15290.1	2
3813	LOC_Os02g33600.1	2
3813	LOC_Os02g51740.1	2
3813	LOC_Os03g20330.1	3
3813	LOC_Os03g20440.1	3
3813	LOC_Os03g26990.1	3
3813	LOC_Os03g47280.1	3
3813	LOC_Os03g57520.1	3
3813	LOC_Os04g34050.1	4
3813	LOC_Os04g55240.1	4
3813	LOC_Os04g57030.1	4
3813	LOC_Os05g12090.1	5
3813	LOC_Os05g32460.1	5
3813	LOC_Os05g41250.1	5
3813	LOC_Os05g44270.1	5
3813	LOC_Os06g33970.1	6
3813	LOC_Os06g40090.1	6
3813	LOC_Os06g41450.1	6
3813	LOC_Os06g45570.1	6
3813	LOC_Os07g06760.1	7
3813	LOC_Os07g06790.1	7
3813	LOC_Os07g06750.1	7
3813	LOC_Os07g43140.1	7
3813	LOC_Os07g48710.1	7
3813	LOC_Os07g48800.1	7
3813	LOC_Os08g01260.1	8
3813	LOC_Os08g31660.1	8
3813	LOC_Os09g20460.1	9
3813	LOC_Os10g01240.1	10
3813	LOC_Os11g03660.1	11
3813	LOC_Os11g12790.1	11
3813	LOC_Os12g03420.1	12

3814	LOC_Os01g11350.1	1
3814	LOC_Os01g36220.1	1
3814	LOC_Os01g55150.1	1
3814	LOC_Os01g58760.1	1
3814	LOC_Os01g64730.1	1
3814	LOC_Os02g03960.1	2
3814	LOC_Os02g07840.1	2
3814	LOC_Os02g09830.1	2
3814	LOC_Os02g10860.1	2
3814	LOC_Os02g16680.1	2
3814	LOC_Os02g49560.1	2
3814	LOC_Os02g58670.1	2
3814	LOC_Os03g19370.1	3
3814	LOC_Os03g47200.1	3
3814	LOC_Os03g56010.1	3
3814	LOC_Os03g58250.1	3
3814	LOC_Os05g03860.1	5
3814	LOC_Os05g34050.1	5
3814	LOC_Os05g41540.1	5
3814	LOC_Os06g39960.1	6
3814	LOC_Os06g41770.1	6
3814	LOC_Os06g42690.1	6
3814	LOC_Os06g45140.1	6
3814	LOC_Os06g50310.1	6
3814	LOC_Os06g50480.1	6
3814	LOC_Os06g50600.1	6
3814	LOC_Os06g50830.1	6
3814	LOC_Os07g03220.1	7
3814	LOC_Os07g08420.1	7
3814	LOC_Os07g44950.1	7
3814	LOC_Os08g26880.1	8
3814	LOC_Os08g38020.1	8
3814	LOC_Os08g43600.1	8
3814	LOC_Os09g13570.1	9
3814	LOC_Os09g29820.1	9
3814	LOC_Os09g36910.1	9
3814	LOC_Os12g37410.1	12
3814	LOC_Os12g40920.1	12
3815	LOC_Os01g28450.1	1
3815	LOC_Os01g28500.1	1
3815	LOC_Os02g27300.1	2
3815	LOC_Os02g54530.1	2
3815	LOC_Os02g54540.1	2
3815	LOC_Os02g54560.1	2
3815	LOC_Os02g54570.1	2
3815	LOC_Os04g22210.1	4
3815	LOC_Os04g22220.1	4
3815	LOC_Os04g22330.1	4
3815	LOC_Os04g22340.1	4
3815	LOC_Os04g22230.1	4
3815	LOC_Os05g51660.1	5
3815	LOC_Os05g51680.1	5

3815	LOC_Os06g24290.1	6
3815	LOC_Os07g03368.1	7
3815	LOC_Os07g03377.1	7
3815	LOC_Os07g03279.1	7
3815	LOC_Os07g03288.1	7
3815	LOC_Os07g03319.1	7
3815	LOC_Os07g03458.1	7
3815	LOC_Os07g03467.1	7
3815	LOC_Os07g03499.1	7
3815	LOC_Os07g03680.1	7
3815	LOC_Os07g03409.1	7
3815	LOC_Os07g03580.1	7
3815	LOC_Os07g03590.1	7
3815	LOC_Os07g03600.1	7
3815	LOC_Os07g03610.1	7
3815	LOC_Os07g03620.1	7
3815	LOC_Os07g03690.1	7
3815	LOC_Os07g03710.1	7
3815	LOC_Os07g03730.1	7
3815	LOC_Os07g03740.1	7
3815	LOC_Os07g03750.1	7
3815	LOC_Os07g14030.1	7
3815	LOC_Os07g45060.1	7
3815	LOC_Os10g11500.1	10
3816	LOC_Os01g15280.1	1
3816	LOC_Os01g24100.1	1
3816	LOC_Os01g39660.1	1
3816	LOC_Os01g58560.1	1
3816	LOC_Os02g40350.1	2
3816	LOC_Os03g08950.1	3
3816	LOC_Os04g53980.1	4
3816	LOC_Os04g55430.1	4
3816	LOC_Os05g04400.1	5
3816	LOC_Os05g07780.1	5
3816	LOC_Os05g15130.1	5
3816	LOC_Os05g46910.1	5
3816	LOC_Os06g33240.1	6
3816	LOC_Os06g36950.1	6
3816	LOC_Os06g38540.1	6
3816	LOC_Os06g45810.1	6
3816	LOC_Os06g48710.1	6
3816	LOC_Os07g06510.1	7
3816	LOC_Os07g09350.1	7
3816	LOC_Os08g12610.1	8
3816	LOC_Os08g20590.1	8
3816	LOC_Os08g23920.1	8
3816	LOC_Os09g21990.1	9
3816	LOC_Os10g10970.1	10
3816	LOC_Os10g11330.1	10
3816	LOC_Os10g17090.1	10
3816	LOC_Os10g20070.1	10
3816	LOC_Os10g26840.1	10

3816	LOC_Os10g36800.1	10
3816	LOC_Os11g04080.1	11
3816	LOC_Os11g09000.1	11
3816	LOC_Os11g38320.1	11
3816	LOC_Os11g43400.1	11
3816	LOC_Os11g43870.1	11
3816	LOC_Os12g01270.1	12
3816	LOC_Os12g12270.1	12
3816	LOC_Os12g18540.1	12
3816	LOC_Os12g38600.1	12
3817	LOC_Os01g12950.1	1
3817	LOC_Os01g16650.1	1
3817	LOC_Os01g42040.1	1
3817	LOC_Os01g48280.1	1
3817	LOC_Os01g60360.1	1
3817	LOC_Os01g60410.1	1
3817	LOC_Os01g62244.1	1
3817	LOC_Os01g46926.1	1
3817	LOC_Os02g02830.1	2
3817	LOC_Os02g16040.1	2
3817	LOC_Os02g48910.1	2
3817	LOC_Os02g48920.1	2
3817	LOC_Os02g48950.1	2
3817	LOC_Os02g42314.1	2
3817	LOC_Os03g03130.1	3
3817	LOC_Os03g19500.1	3
3817	LOC_Os03g47580.1	3
3817	LOC_Os03g47770.1	3
3817	LOC_Os03g50440.1	3
3817	LOC_Os03g57790.1	3
3817	LOC_Os04g49130.1	4
3817	LOC_Os04g57220.1	4
3817	LOC_Os04g58800.1	4
3817	LOC_Os05g08960.1	5
3817	LOC_Os05g38550.1	5
3817	LOC_Os06g09330.1	6
3817	LOC_Os06g30970.1	6
3817	LOC_Os06g45000.1	6
3817	LOC_Os07g07240.1	7
3817	LOC_Os07g38940.1	7
3817	LOC_Os08g28680.1	8
3817	LOC_Os09g12230.1	9
3817	LOC_Os09g12570.1	9
3817	LOC_Os09g15320.1	9
3817	LOC_Os10g11260.1	10
3817	LOC_Os10g31000.1	10
3817	LOC_Os10g39120.1	10
3817	LOC_Os12g41220.1	12
3817	LOC_Os12g44000.1	12
3818	LOC_Os01g51610.1	1
3818	LOC_Os01g52514.1	1
3818	LOC_Os01g52540.1	1

3818	LOC_Os01g67830.1	1
3818	LOC_Os01g68370.1	1
3818	LOC_Os02g25820.1	2
3818	LOC_Os02g38470.1	2
3818	LOC_Os02g45850.1	2
3818	LOC_Os03g02900.1	3
3818	LOC_Os03g11370.1	3
3818	LOC_Os03g06850.1	3
3818	LOC_Os03g08620.1	3
3818	LOC_Os03g42370.1	3
3818	LOC_Os03g42410.1	3
3818	LOC_Os03g42420.1	3
3818	LOC_Os03g42430.1	3
3818	LOC_Os03g42280.1	3
3818	LOC_Os04g27960.1	4
3818	LOC_Os04g27990.1	4
3818	LOC_Os04g28000.1	4
3818	LOC_Os04g49230.1	4
3818	LOC_Os04g58000.1	4
3818	LOC_Os04g58010.1	4
3818	LOC_Os05g40280.1	5
3818	LOC_Os06g02230.1	6
3818	LOC_Os06g09420.1	6
3818	LOC_Os06g42630.1	6
3818	LOC_Os08g01090.1	8
3818	LOC_Os08g06120.1	8
3818	LOC_Os08g23470.1	8
3818	LOC_Os08g23570.1	8
3818	LOC_Os10g07060.1	10
3818	LOC_Os10g17630.1	10
3818	LOC_Os10g39190.1	10
3818	LOC_Os11g05740.1	11
3818	LOC_Os11g09160.1	11
3818	LOC_Os12g40070.1	12
3818	LOC_Os12g40090.1	12
3818	LOC_Os12g40120.1	12
3819	LOC_Os01g07080.1	1
3819	LOC_Os01g08930.1	1
3819	LOC_Os01g10050.1	1
3819	LOC_Os01g36920.1	1
3819	LOC_Os01g36890.1	1
3819	LOC_Os01g45190.1	1
3819	LOC_Os01g68320.1	1
3819	LOC_Os01g73900.1	1
3819	LOC_Os02g02150.1	2
3819	LOC_Os02g05330.1	2
3819	LOC_Os02g12840.1	2
3819	LOC_Os02g42406.1	2
3819	LOC_Os02g42860.1	2
3819	LOC_Os02g54020.1	2
3819	LOC_Os02g55260.1	2
3819	LOC_Os03g01830.1	3

3819	LOC_Os03g06220.1	3
3819	LOC_Os03g12000.1	3
3819	LOC_Os03g19530.1	3
3819	LOC_Os03g36930.1	3
3819	LOC_Os03g46610.1	3
3819	LOC_Os03g51900.1	3
3819	LOC_Os03g58810.1	3
3819	LOC_Os04g43140.1	4
3819	LOC_Os04g45040.1	4
3819	LOC_Os05g01990.1	5
3819	LOC_Os05g05810.1	5
3819	LOC_Os06g48750.1	6
3819	LOC_Os07g20580.1	7
3819	LOC_Os07g33340.1	7
3819	LOC_Os07g43980.1	7
3819	LOC_Os07g46580.1	7
3819	LOC_Os07g05050.1	7
3819	LOC_Os08g05810.1	8
3819	LOC_Os08g32090.1	8
3819	LOC_Os10g35990.1	10
3819	LOC_Os11g44910.1	11
3819	LOC_Os11g48090.1	11
3819	LOC_Os11g32880.1	11
3819	LOC_Os12g29660.1	12
3820	LOC_Os01g31980.1	1
3820	LOC_Os01g49120.1	1
3820	LOC_Os01g56050.1	1
3820	LOC_Os02g57570.1	2
3820	LOC_Os03g08910.1	3
3820	LOC_Os03g12790.1	3
3820	LOC_Os03g37411.1	3
3820	LOC_Os03g37470.1	3
3820	LOC_Os03g37490.1	3
3820	LOC_Os03g37640.1	3
3820	LOC_Os03g42830.1	3
3820	LOC_Os03g62270.1	3
3820	LOC_Os03g64150.1	3
3820	LOC_Os04g30490.1	4
3820	LOC_Os04g48290.1	4
3820	LOC_Os05g48040.1	5
3820	LOC_Os06g29844.1	6
3820	LOC_Os06g29994.1	6
3820	LOC_Os06g49310.1	6
3820	LOC_Os07g01750.1	7
3820	LOC_Os07g31884.1	7
3820	LOC_Os07g33310.1	7
3820	LOC_Os08g37432.1	8
3820	LOC_Os08g43250.1	8
3820	LOC_Os08g43654.1	8
3820	LOC_Os08g44870.1	8
3820	LOC_Os09g29284.1	9
3820	LOC_Os10g11354.1	10

3820	LOC_Os10g11860.1	10
3820	LOC_Os10g20450.1	10
3820	LOC_Os10g20470.1	10
3820	LOC_Os10g20350.1	10
3820	LOC_Os10g20390.1	10
3820	LOC_Os10g37920.1	10
3820	LOC_Os11g03484.1	11
3820	LOC_Os11g03240.1	11
3820	LOC_Os12g03230.1	12
3820	LOC_Os12g03260.1	12
3820	LOC_Os12g42130.1	12
3820	LOC_Os12g03200.1	12
3821	LOC_Os01g27410.1	1
3821	LOC_Os01g39640.1	1
3821	LOC_Os03g33690.1	3
3821	LOC_Os03g34070.1	3
3821	LOC_Os03g34090.1	3
3821	LOC_Os03g49080.1	3
3821	LOC_Os04g03960.1	4
3821	LOC_Os04g07680.1	4
3821	LOC_Os04g11290.1	4
3821	LOC_Os04g14600.1	4
3821	LOC_Os04g14630.1	4
3821	LOC_Os04g17090.1	4
3821	LOC_Os04g17900.1	4
3821	LOC_Os04g18930.1	4
3821	LOC_Os04g27650.1	4
3821	LOC_Os05g20900.1	5
3821	LOC_Os05g21050.1	5
3821	LOC_Os05g27390.1	5
3821	LOC_Os06g15660.1	6
3821	LOC_Os06g26040.1	6
3821	LOC_Os06g38230.1	6
3821	LOC_Os07g02860.1	7
3821	LOC_Os07g13200.1	7
3821	LOC_Os08g11840.1	8
3821	LOC_Os08g11130.1	8
3821	LOC_Os08g11140.1	8
3821	LOC_Os08g11880.1	8
3821	LOC_Os08g28290.1	8
3821	LOC_Os09g13160.1	9
3821	LOC_Os09g15000.1	9
3821	LOC_Os09g22340.1	9
3821	LOC_Os10g11970.1	10
3821	LOC_Os10g17290.1	10
3821	LOC_Os10g17040.1	10
3821	LOC_Os11g23900.1	11
3821	LOC_Os11g40300.1	11
3821	LOC_Os12g09810.1	12
3821	LOC_Os12g11490.1	12
3821	LOC_Os12g14860.1	12
3821	LOC_Os12g23100.1	12

3821	LOC_Os12g39270.1	12
3822	LOC_Os01g14510.1	1
3822	LOC_Os01g28730.1	1
3822	LOC_Os01g40590.1	1
3822	LOC_Os01g63280.1	1
3822	LOC_Os01g67340.1	1
3822	LOC_Os02g08530.1	2
3822	LOC_Os02g35760.1	2
3822	LOC_Os02g43290.1	2
3822	LOC_Os02g57080.1	2
3822	LOC_Os02g53750.1	2
3822	LOC_Os03g05470.1	3
3822	LOC_Os03g03890.1	3
3822	LOC_Os03g12520.1	3
3822	LOC_Os03g13820.1	3
3822	LOC_Os03g24930.1	3
3822	LOC_Os03g51040.1	3
3822	LOC_Os03g58750.1	3
3822	LOC_Os03g61060.1	3
3822	LOC_Os04g45730.1	4
3822	LOC_Os04g45920.1	4
3822	LOC_Os04g41310.1	4
3822	LOC_Os05g04520.1	5
3822	LOC_Os05g11750.1	5
3822	LOC_Os05g14220.1	5
3822	LOC_Os05g33080.1	5
3822	LOC_Os06g07230.1	6
3822	LOC_Os06g45280.1	6
3822	LOC_Os06g47820.1	6
3822	LOC_Os06g50100.1	6
3822	LOC_Os08g35600.1	8
3822	LOC_Os09g07730.1	9
3822	LOC_Os09g16950.1	9
3822	LOC_Os09g19700.1	9
3822	LOC_Os09g33630.1	9
3822	LOC_Os09g33860.1	9
3822	LOC_Os09g39930.1	9
3822	LOC_Os10g25550.1	10
3822	LOC_Os10g30600.1	10
3822	LOC_Os10g34220.1	10
3822	LOC_Os11g02830.1	11
3822	LOC_Os11g25860.1	11
3822	LOC_Os12g02810.1	12
3823	LOC_Os01g14670.1	1
3823	LOC_Os01g18170.1	1
3823	LOC_Os01g50900.1	1
3823	LOC_Os01g72290.1	1
3823	LOC_Os01g72300.1	1
3823	LOC_Os02g29000.1	2
3823	LOC_Os02g29010.1	2
3823	LOC_Os02g29020.1	2
3823	LOC_Os02g32980.1	2

3823	LOC_Os03g08150.1	3
3823	LOC_Os03g44880.1	3
3823	LOC_Os03g48750.1	3
3823	LOC_Os03g48760.1	3
3823	LOC_Os03g48770.1	3
3823	LOC_Os03g48780.1	3
3823	LOC_Os03g58980.1	3
3823	LOC_Os03g59010.1	3
3823	LOC_Os04g52720.1	4
3823	LOC_Os05g19670.1	5
3823	LOC_Os05g10830.1	5
3823	LOC_Os08g08920.1	8
3823	LOC_Os08g08960.1	8
3823	LOC_Os08g08970.1	8
3823	LOC_Os08g08990.1	8
3823	LOC_Os08g09000.1	8
3823	LOC_Os08g09010.1	8
3823	LOC_Os08g08980.1	8
3823	LOC_Os08g09060.1	8
3823	LOC_Os08g09080.1	8
3823	LOC_Os08g09020.1	8
3823	LOC_Os08g09040.1	8
3823	LOC_Os08g35760.1	8
3823	LOC_Os08g35750.1	8
3823	LOC_Os09g37976.1	9
3823	LOC_Os09g39510.1	9
3823	LOC_Os09g39520.1	9
3823	LOC_Os09g39530.1	9
3823	LOC_Os11g33110.1	11
3823	LOC_Os12g05840.1	12
3823	LOC_Os12g05860.1	12
3823	LOC_Os12g05870.1	12
3823	LOC_Os12g05880.1	12
3824	LOC_Os01g13990.1	1
3824	LOC_Os01g23830.1	1
3824	LOC_Os01g27200.1	1
3824	LOC_Os01g48090.1	1
3824	LOC_Os02g29840.1	2
3824	LOC_Os02g32910.1	2
3824	LOC_Os03g22860.1	3
3824	LOC_Os03g41970.1	3
3824	LOC_Os04g11670.1	4
3824	LOC_Os04g13120.1	4
3824	LOC_Os04g23010.1	4
3824	LOC_Os04g43240.1	4
3824	LOC_Os04g49010.1	4
3824	LOC_Os04g54170.1	4
3824	LOC_Os05g17880.1	5
3824	LOC_Os05g18400.1	5
3824	LOC_Os06g01280.1	6
3824	LOC_Os06g18770.1	6
3824	LOC_Os06g25980.1	6

3824	LOC_Os06g34200.1	6
3824	LOC_Os07g13740.1	7
3824	LOC_Os07g14170.1	7
3824	LOC_Os07g16640.1	7
3824	LOC_Os07g17940.1	7
3824	LOC_Os07g49060.1	7
3824	LOC_Os08g16550.1	8
3824	LOC_Os08g17259.1	8
3824	LOC_Os08g29550.1	8
3824	LOC_Os08g32050.1	8
3824	LOC_Os08g40730.1	8
3824	LOC_Os09g03220.1	9
3824	LOC_Os09g14090.1	9
3824	LOC_Os09g19590.1	9
3824	LOC_Os09g29030.1	9
3824	LOC_Os10g11720.1	10
3824	LOC_Os10g32210.1	10
3824	LOC_Os11g18800.1	11
3824	LOC_Os11g39410.1	11
3824	LOC_Os12g13240.1	12
3824	LOC_Os12g15660.1	12
3824	LOC_Os12g22730.1	12
3824	LOC_Os12g23390.1	12
3825	LOC_Os01g06000.1	1
3825	LOC_Os01g12810.1	1
3825	LOC_Os01g15840.1	1
3825	LOC_Os01g57410.1	1
3825	LOC_Os01g63400.1	1
3825	LOC_Os01g68070.1	1
3825	LOC_Os01g73950.1	1
3825	LOC_Os02g02740.1	2
3825	LOC_Os02g21580.1	2
3825	LOC_Os02g26890.1	2
3825	LOC_Os02g38950.1	2
3825	LOC_Os02g57800.1	2
3825	LOC_Os03g06910.1	3
3825	LOC_Os03g11020.1	3
3825	LOC_Os03g10420.1	3
3825	LOC_Os03g11690.1	3
3825	LOC_Os03g12430.1	3
3825	LOC_Os03g24880.1	3
3825	LOC_Os03g40020.1	3
3825	LOC_Os03g40690.1	3
3825	LOC_Os03g51840.1	3
3825	LOC_Os03g53490.1	3
3825	LOC_Os03g55340.1	3
3825	LOC_Os04g41140.1	4
3825	LOC_Os04g57900.1	4
3825	LOC_Os05g11700.1	5
3825	LOC_Os05g19390.1	5
3825	LOC_Os05g28720.1	5
3825	LOC_Os05g24930.1	5

3825	LOC_Os06g47570.1	6
3825	LOC_Os07g08180.1	7
3825	LOC_Os07g14530.1	7
3825	LOC_Os07g32900.1	7
3825	LOC_Os07g36390.1	7
3825	LOC_Os07g48850.1	7
3825	LOC_Os08g19310.1	8
3825	LOC_Os09g20830.1	9
3825	LOC_Os10g02650.1	10
3825	LOC_Os10g21920.1	10
3825	LOC_Os10g33700.1	10
3825	LOC_Os10g34310.1	10
3825	LOC_Os12g21930.1	12
3825	LOC_Os12g42120.1	12
3826	LOC_Os01g60450.1	1
3826	LOC_Os01g72740.1	1
3826	LOC_Os02g01890.1	2
3826	LOC_Os02g09310.1	2
3826	LOC_Os02g12550.1	2
3826	LOC_Os02g29720.1	2
3826	LOC_Os02g36070.1	2
3826	LOC_Os02g36110.1	2
3826	LOC_Os02g36280.1	2
3826	LOC_Os02g38940.1	2
3826	LOC_Os03g02180.1	3
3826	LOC_Os03g39650.1	3
3826	LOC_Os03g63310.1	3
3826	LOC_Os04g27020.1	4
3826	LOC_Os05g41440.1	5
3826	LOC_Os06g19070.1	6
3826	LOC_Os06g30179.1	6
3826	LOC_Os06g30500.1	6
3826	LOC_Os06g39780.1	6
3826	LOC_Os06g43304.1	6
3826	LOC_Os06g43410.1	6
3826	LOC_Os06g43350.1	6
3826	LOC_Os06g43370.1	6
3826	LOC_Os06g45960.1	6
3826	LOC_Os07g11970.1	7
3826	LOC_Os07g26870.1	7
3826	LOC_Os08g01490.1	8
3826	LOC_Os08g03682.1	8
3826	LOC_Os08g05620.1	8
3826	LOC_Os08g36310.1	8
3826	LOC_Os08g39730.1	8
3826	LOC_Os08g39660.1	8
3826	LOC_Os08g39694.1	8
3826	LOC_Os09g10340.1	9
3826	LOC_Os09g26940.1	9
3826	LOC_Os09g26980.1	9
3826	LOC_Os09g27500.1	9
3826	LOC_Os10g05020.1	10

3826	LOC_Os10g05490.1	10
3826	LOC_Os10g08474.1	10
3826	LOC_Os10g36848.1	10
3826	LOC_Os12g09790.1	12
3826	LOC_Os12g09500.1	12
3826	LOC_Os12g44290.1	12
3827	LOC_Os01g13960.1	1
3827	LOC_Os01g37270.1	1
3827	LOC_Os02g15140.1	2
3827	LOC_Os02g20810.1	2
3827	LOC_Os02g26230.1	2
3827	LOC_Os02g45990.1	2
3827	LOC_Os02g47250.1	2
3827	LOC_Os02g49380.1	2
3827	LOC_Os03g24010.1	3
3827	LOC_Os03g30980.1	3
3827	LOC_Os03g41950.1	3
3827	LOC_Os04g01760.1	4
3827	LOC_Os04g14780.1	4
3827	LOC_Os04g15510.1	4
3827	LOC_Os04g20650.1	4
3827	LOC_Os04g24860.1	4
3827	LOC_Os04g25770.1	4
3827	LOC_Os04g43260.1	4
3827	LOC_Os04g47710.1	4
3827	LOC_Os05g19080.1	5
3827	LOC_Os05g30100.1	5
3827	LOC_Os05g31940.1	5
3827	LOC_Os05g17980.1	5
3827	LOC_Os05g25720.1	5
3827	LOC_Os05g39010.1	5
3827	LOC_Os06g24660.1	6
3827	LOC_Os06g46510.1	6
3827	LOC_Os07g30030.1	7
3827	LOC_Os07g31160.1	7
3827	LOC_Os07g49180.1	7
3827	LOC_Os08g08600.1	8
3827	LOC_Os08g15670.1	8
3827	LOC_Os08g16500.1	8
3827	LOC_Os08g20690.1	8
3827	LOC_Os08g25980.1	8
3827	LOC_Os10g01330.1	10
3827	LOC_Os10g02440.1	10
3827	LOC_Os10g32260.1	10
3827	LOC_Os10g34090.1	10
3827	LOC_Os11g13480.1	11
3827	LOC_Os11g18780.1	11
3827	LOC_Os11g19400.1	11
3827	LOC_Os11g20430.1	11
3827	LOC_Os11g23160.1	11
3827	LOC_Os11g29540.1	11
3827	LOC_Os12g11890.1	12

3827	LOC_Os12g21680.1	12
3827	LOC_Os12g30270.1	12
3828	LOC_Os01g05610.1	1
3828	LOC_Os01g05630.1	1
3828	LOC_Os01g05900.1	1
3828	LOC_Os01g05950.1	1
3828	LOC_Os01g05990.1	1
3828	LOC_Os01g06010.1	1
3828	LOC_Os01g31800.1	1
3828	LOC_Os01g31810.1	1
3828	LOC_Os01g61920.1	1
3828	LOC_Os01g62230.1	1
3828	LOC_Os01g64640.1	1
3828	LOC_Os02g25910.1	2
3828	LOC_Os02g25940.1	2
3828	LOC_Os02g45940.1	2
3828	LOC_Os03g02780.1	3
3828	LOC_Os03g06670.1	3
3828	LOC_Os03g17084.1	3
3828	LOC_Os03g17100.1	3
3828	LOC_Os03g27310.1	3
3828	LOC_Os03g51200.1	3
3828	LOC_Os03g53190.1	3
3828	LOC_Os04g13530.1	4
3828	LOC_Os04g34240.1	4
3828	LOC_Os04g37780.1	4
3828	LOC_Os04g49420.1	4
3828	LOC_Os05g02300.1	5
3828	LOC_Os05g38560.1	5
3828	LOC_Os05g38740.1	5
3828	LOC_Os05g41080.1	5
3828	LOC_Os05g36280.1	5
3828	LOC_Os05g38640.1	5
3828	LOC_Os05g38760.1	5
3828	LOC_Os05g39050.1	5
3828	LOC_Os05g49860.1	5
3828	LOC_Os06g04030.1	6
3828	LOC_Os06g06460.1	6
3828	LOC_Os06g06480.1	6
3828	LOC_Os06g06510.1	6
3828	LOC_Os07g36500.1	7
3828	LOC_Os08g38300.1	8
3828	LOC_Os09g26340.1	9
3828	LOC_Os09g38020.1	9
3828	LOC_Os09g39730.1	9
3828	LOC_Os10g28230.1	10
3828	LOC_Os10g39410.1	10
3828	LOC_Os11g05730.1	11
3828	LOC_Os12g22680.1	12
3828	LOC_Os12g22650.1	12
3828	LOC_Os12g34510.1	12
3829	LOC_Os01g04990.1	1

3829	LOC_Os01g12520.1	1
3829	LOC_Os01g12470.1	1
3829	LOC_Os01g16040.1	1
3829	LOC_Os01g22600.1	1
3829	LOC_Os01g32980.1	1
3829	LOC_Os01g38990.1	1
3829	LOC_Os01g51250.1	1
3829	LOC_Os01g70800.1	1
3829	LOC_Os01g73420.1	1
3829	LOC_Os01g74640.1	1
3829	LOC_Os02g10800.1	2
3829	LOC_Os02g13170.1	2
3829	LOC_Os02g13300.1	2
3829	LOC_Os02g44570.1	2
3829	LOC_Os02g48720.1	2
3829	LOC_Os03g11510.1	3
3829	LOC_Os03g07890.1	3
3829	LOC_Os03g09110.1	3
3829	LOC_Os03g15860.1	3
3829	LOC_Os03g16080.1	3
3829	LOC_Os03g18160.1	3
3829	LOC_Os03g18550.1	3
3829	LOC_Os03g52430.1	3
3829	LOC_Os03g54760.1	3
3829	LOC_Os04g44540.1	4
3829	LOC_Os05g07900.1	5
3829	LOC_Os05g23720.1	5
3829	LOC_Os05g32630.1	5
3829	LOC_Os05g11780.1	5
3829	LOC_Os05g28870.1	5
3829	LOC_Os05g29860.1	5
3829	LOC_Os05g46220.1	5
3829	LOC_Os05g50840.1	5
3829	LOC_Os06g40050.1	6
3829	LOC_Os07g19460.1	7
3829	LOC_Os08g29720.1	8
3829	LOC_Os08g37370.1	8
3829	LOC_Os08g40850.1	8
3829	LOC_Os09g27240.1	9
3829	LOC_Os09g29050.1	9
3829	LOC_Os09g32200.1	9
3829	LOC_Os09g33470.1	9
3829	LOC_Os10g25830.1	10
3829	LOC_Os10g42299.1	10
3829	LOC_Os11g23170.1	11
3829	LOC_Os11g24450.1	11
3829	LOC_Os11g43960.1	11
3829	LOC_Os11g48040.1	11
3829	LOC_Os12g34870.1	12
3830	LOC_Os01g10250.1	1
3830	LOC_Os01g15120.1	1
3830	LOC_Os01g15130.1	1

3830	LOC_Os01g39790.1	1
3830	LOC_Os01g41240.1	1
3830	LOC_Os01g44960.1	1
3830	LOC_Os01g45960.1	1
3830	LOC_Os01g62010.1	1
3830	LOC_Os01g65070.1	1
3830	LOC_Os01g66240.1	1
3830	LOC_Os01g69060.1	1
3830	LOC_Os02g37090.1	2
3830	LOC_Os02g43340.1	2
3830	LOC_Os02g47780.1	2
3830	LOC_Os02g19650.1	2
3830	LOC_Os02g47620.1	2
3830	LOC_Os03g10620.1	3
3830	LOC_Os03g32270.1	3
3830	LOC_Os03g51010.1	3
3830	LOC_Os03g61340.1	3
3830	LOC_Os03g61360.1	3
3830	LOC_Os04g36620.1	4
3830	LOC_Os04g55180.1	4
3830	LOC_Os05g08740.1	5
3830	LOC_Os05g34630.1	5
3830	LOC_Os05g19150.1	5
3830	LOC_Os05g35730.1	5
3830	LOC_Os05g43830.1	5
3830	LOC_Os05g46460.1	5
3830	LOC_Os05g51240.1	5
3830	LOC_Os06g04169.1	6
3830	LOC_Os06g06820.1	6
3830	LOC_Os06g24730.1	6
3830	LOC_Os07g38830.1	7
3830	LOC_Os07g41080.1	7
3830	LOC_Os07g48610.1	7
3830	LOC_Os09g23150.1	9
3830	LOC_Os09g34860.1	9
3830	LOC_Os09g24710.1	9
3830	LOC_Os10g04620.1	10
3830	LOC_Os10g08840.1	10
3830	LOC_Os10g35530.1	10
3830	LOC_Os10g35540.1	10
3830	LOC_Os10g35490.1	10
3830	LOC_Os10g35520.1	10
3830	LOC_Os10g38860.1	10
3830	LOC_Os11g01040.1	11
3830	LOC_Os11g02660.1	11
3830	LOC_Os12g01030.1	12
3830	LOC_Os12g43970.1	12
3831	LOC_Os01g01840.1	1
3831	LOC_Os01g01870.1	1
3831	LOC_Os01g09930.1	1
3831	LOC_Os01g13460.1	1
3831	LOC_Os01g18290.1	1

3831	LOC_Os01g18870.1	1
3831	LOC_Os01g33400.1	1
3831	LOC_Os01g39330.1	1
3831	LOC_Os01g50940.1	1
3831	LOC_Os01g51140.1	1
3831	LOC_Os01g56690.1	1
3831	LOC_Os01g61480.1	1
3831	LOC_Os02g02820.1	2
3831	LOC_Os02g12820.1	2
3831	LOC_Os02g13670.1	2
3831	LOC_Os02g15760.1	2
3831	LOC_Os02g23823.1	2
3831	LOC_Os02g47660.1	2
3831	LOC_Os02g48060.1	2
3831	LOC_Os02g39140.1	2
3831	LOC_Os03g03000.1	3
3831	LOC_Os03g26210.1	3
3831	LOC_Os03g39432.1	3
3831	LOC_Os03g46790.1	3
3831	LOC_Os03g53020.1	3
3831	LOC_Os04g23440.1	4
3831	LOC_Os04g31290.1	4
3831	LOC_Os04g35010.1	4
3831	LOC_Os04g51070.1	4
3831	LOC_Os04g52770.1	4
3831	LOC_Os05g06520.1	5
3831	LOC_Os05g11070.1	5
3831	LOC_Os06g16400.1	6
3831	LOC_Os06g33450.1	6
3831	LOC_Os06g37410.1	6
3831	LOC_Os07g36460.1	7
3831	LOC_Os07g43530.1	7
3831	LOC_Os08g38080.1	8
3831	LOC_Os08g43070.1	8
3831	LOC_Os09g29830.1	9
3831	LOC_Os09g34330.1	9
3831	LOC_Os10g01530.1	10
3831	LOC_Os10g40740.1	10
3831	LOC_Os11g38870.1	11
3831	LOC_Os11g41640.1	11
3831	LOC_Os12g31430.1	12
3831	LOC_Os12g40590.1	12
3831	LOC_Os12g40630.1	12
3831	LOC_Os12g40710.1	12
3831	LOC_Os12g43620.1	12
3831	LOC_Os12g41650.1	12
3832	LOC_Os01g08450.1	1
3832	LOC_Os01g12730.1	1
3832	LOC_Os01g12900.1	1
3832	LOC_Os01g35850.1	1
3832	LOC_Os01g37800.1	1
3832	LOC_Os01g42530.1	1

3832	LOC_Os01g47730.1	1
3832	LOC_Os01g51700.1	1
3832	LOC_Os01g54590.1	1
3832	LOC_Os01g62950.1	1
3832	LOC_Os02g02840.1	2
3832	LOC_Os02g20850.1	2
3832	LOC_Os02g37420.1	2
3832	LOC_Os02g50860.1	2
3832	LOC_Os02g58730.1	2
3832	LOC_Os02g43690.1	2
3832	LOC_Os03g05280.1	3
3832	LOC_Os03g05740.1	3
3832	LOC_Os03g09140.1	3
3832	LOC_Os03g46390.1	3
3832	LOC_Os03g60530.1	3
3832	LOC_Os03g60870.1	3
3832	LOC_Os03g62600.1	3
3832	LOC_Os04g39440.1	4
3832	LOC_Os05g01490.1	5
3832	LOC_Os05g01480.1	5
3832	LOC_Os05g20050.1	5
3832	LOC_Os05g38630.1	5
3832	LOC_Os05g27530.1	5
3832	LOC_Os05g44050.1	5
3832	LOC_Os05g46000.1	5
3832	LOC_Os05g43820.1	5
3832	LOC_Os05g44070.1	5
3832	LOC_Os05g48980.1	5
3832	LOC_Os05g49890.1	5
3832	LOC_Os06g12790.1	6
3832	LOC_Os06g35814.1	6
3832	LOC_Os06g47260.1	6
3832	LOC_Os06g50060.1	6
3832	LOC_Os07g09680.1	7
3832	LOC_Os07g13530.1	7
3832	LOC_Os07g31370.1	7
3832	LOC_Os07g44040.1	7
3832	LOC_Os08g41340.1	8
3832	LOC_Os09g10940.1	9
3832	LOC_Os09g15790.1	9
3832	LOC_Os09g35860.1	9
3832	LOC_Os10g14150.1	10
3832	LOC_Os10g23100.1	10
3832	LOC_Os10g30520.1	10
3832	LOC_Os10g31830.1	10
3832	LOC_Os12g43550.1	12
3833	LOC_Os01g16610.1	1
3833	LOC_Os01g17470.1	1
3833	LOC_Os01g54430.1	1
3833	LOC_Os01g57880.1	1
3833	LOC_Os02g06690.1	2
3833	LOC_Os02g15710.1	2

3833	LOC_Os02g15730.1	2
3833	LOC_Os02g48820.1	2
3833	LOC_Os02g49350.1	2
3833	LOC_Os02g49850.1	2
3833	LOC_Os02g52180.1	2
3833	LOC_Os03g02400.1	3
3833	LOC_Os03g15340.1	3
3833	LOC_Os03g44630.1	3
3833	LOC_Os03g50140.1	3
3833	LOC_Os03g50160.1	3
3833	LOC_Os03g55120.1	3
3833	LOC_Os03g57740.1	3
3833	LOC_Os03g63390.1	3
3833	LOC_Os04g34480.1	4
3833	LOC_Os04g46120.1	4
3833	LOC_Os04g46130.1	4
3833	LOC_Os04g53710.1	4
3833	LOC_Os04g57750.1	4
3833	LOC_Os05g49580.1	5
3833	LOC_Os06g11310.1	6
3833	LOC_Os06g15600.1	6
3833	LOC_Os06g17730.1	6
3833	LOC_Os06g36010.1	6
3833	LOC_Os06g50420.1	6
3833	LOC_Os06g50650.1	6
3833	LOC_Os07g01440.1	7
3833	LOC_Os07g02200.1	7
3833	LOC_Os07g07170.1	7
3833	LOC_Os07g35860.1	7
3833	LOC_Os07g38290.1	7
3833	LOC_Os08g04310.1	8
3833	LOC_Os08g04340.1	8
3833	LOC_Os08g04350.1	8
3833	LOC_Os08g04360.1	8
3833	LOC_Os08g04370.1	8
3833	LOC_Os08g17160.1	8
3833	LOC_Os08g37660.1	8
3833	LOC_Os08g37670.1	8
3833	LOC_Os09g29390.1	9
3833	LOC_Os09g36940.1	9
3833	LOC_Os09g38540.1	9
3833	LOC_Os09g39940.1	9
3833	LOC_Os11g24140.1	11
3833	LOC_Os11g29910.1	11
3833	LOC_Os12g05470.1	12
3833	LOC_Os12g26880.1	12
3834	LOC_Os01g24990.1	1
3834	LOC_Os01g28320.1	1
3834	LOC_Os01g29507.1	1
3834	LOC_Os01g31290.1	1
3834	LOC_Os01g36310.1	1
3834	LOC_Os01g40780.1	1

3834	LOC_Os02g24050.1	2
3834	LOC_Os02g25160.1	2
3834	LOC_Os02g25190.1	2
3834	LOC_Os02g25350.1	2
3834	LOC_Os03g32840.1	3
3834	LOC_Os03g35894.1	3
3834	LOC_Os03g38110.1	3
3834	LOC_Os04g10080.1	4
3834	LOC_Os04g13080.1	4
3834	LOC_Os04g18990.1	4
3834	LOC_Os05g24400.1	5
3834	LOC_Os05g27190.1	5
3834	LOC_Os05g17400.1	5
3834	LOC_Os06g23710.1	6
3834	LOC_Os06g27430.1	6
3834	LOC_Os06g28570.1	6
3834	LOC_Os06g34210.1	6
3834	LOC_Os06g35610.1	6
3834	LOC_Os07g18030.1	7
3834	LOC_Os07g21970.1	7
3834	LOC_Os07g22330.1	7
3834	LOC_Os07g25910.1	7
3834	LOC_Os08g19030.1	8
3834	LOC_Os08g21500.1	8
3834	LOC_Os08g21810.1	8
3834	LOC_Os08g21849.1	8
3834	LOC_Os08g21950.1	8
3834	LOC_Os08g26390.1	8
3834	LOC_Os09g06330.1	9
3834	LOC_Os09g06300.1	9
3834	LOC_Os10g03080.1	10
3834	LOC_Os10g14940.1	10
3834	LOC_Os10g17190.1	10
3834	LOC_Os10g23860.1	10
3834	LOC_Os11g08729.1	11
3834	LOC_Os11g08889.1	11
3834	LOC_Os11g11850.1	11
3834	LOC_Os11g22470.1	11
3834	LOC_Os11g22500.1	11
3834	LOC_Os11g26010.1	11
3834	LOC_Os11g26450.1	11
3834	LOC_Os12g08400.1	12
3834	LOC_Os12g18440.1	12
3834	LOC_Os12g19990.1	12
3834	LOC_Os12g20100.1	12
3834	LOC_Os12g24350.1	12
3835	LOC_Os01g09100.1	1
3835	LOC_Os01g09080.1	1
3835	LOC_Os01g40260.1	1
3835	LOC_Os01g43650.1	1
3835	LOC_Os01g46800.1	1
3835	LOC_Os01g51690.1	1

3835	LOC_Os01g54600.1	1
3835	LOC_Os01g60490.1	1
3835	LOC_Os01g60640.1	1
3835	LOC_Os01g60520.1	1
3835	LOC_Os01g60540.1	1
3835	LOC_Os01g60600.1	1
3835	LOC_Os01g62510.1	1
3835	LOC_Os02g08440.1	2
3835	LOC_Os02g16540.1	2
3835	LOC_Os03g20550.1	3
3835	LOC_Os03g45450.1	3
3835	LOC_Os03g58420.1	3
3835	LOC_Os03g63810.1	3
3835	LOC_Os03g33020.1	3
3835	LOC_Os05g03900.1	5
3835	LOC_Os05g09020.1	5
3835	LOC_Os05g25700.1	5
3835	LOC_Os05g14370.1	5
3835	LOC_Os05g40060.1	5
3835	LOC_Os05g40070.1	5
3835	LOC_Os05g40080.1	5
3835	LOC_Os05g46020.1	5
3835	LOC_Os05g49210.1	5
3835	LOC_Os05g49620.1	5
3835	LOC_Os05g50700.1	5
3835	LOC_Os06g05380.1	6
3835	LOC_Os06g30860.1	6
3835	LOC_Os07g27670.1	7
3835	LOC_Os07g40570.1	7
3835	LOC_Os08g17400.1	8
3835	LOC_Os08g29660.1	8
3835	LOC_Os09g09630.1	9
3835	LOC_Os09g16510.1	9
3835	LOC_Os09g25060.1	9
3835	LOC_Os10g18110.1	10
3835	LOC_Os10g42850.1	10
3835	LOC_Os11g02470.1	11
3835	LOC_Os11g02480.1	11
3835	LOC_Os11g02520.1	11
3835	LOC_Os11g02530.1	11
3835	LOC_Os11g02540.1	11
3835	LOC_Os11g45850.1	11
3835	LOC_Os12g02450.1	12
3835	LOC_Os12g02400.1	12
3835	LOC_Os12g32250.1	12
3835	LOC_Os12g02420.1	12
3835	LOC_Os12g02440.1	12
3836	LOC_Os01g06230.1	1
3836	LOC_Os01g56240.1	1
3836	LOC_Os01g70050.1	1
3836	LOC_Os02g05050.1	2
3836	LOC_Os02g05060.1	2

3836	LOC_Os02g07110.1	2
3836	LOC_Os02g20320.1	2
3836	LOC_Os02g24740.1	2
3836	LOC_Os02g24700.1	2
3836	LOC_Os02g30810.1	2
3836	LOC_Os02g42990.1	2
3836	LOC_Os02g52990.1	2
3836	LOC_Os03g18050.1	3
3836	LOC_Os03g25289.1	3
3836	LOC_Os03g45800.1	3
3836	LOC_Os03g45830.1	3
3836	LOC_Os03g45850.1	3
3836	LOC_Os03g45860.1	3
3836	LOC_Os04g43740.1	4
3836	LOC_Os04g51890.1	4
3836	LOC_Os04g52670.1	4
3836	LOC_Os04g56680.1	4
3836	LOC_Os04g56690.1	4
3836	LOC_Os06g04590.1	6
3836	LOC_Os06g45970.1	6
3836	LOC_Os06g45950.1	6
3836	LOC_Os06g48850.1	6
3836	LOC_Os06g48860.1	6
3836	LOC_Os06g50040.1	6
3836	LOC_Os07g29310.1	7
3836	LOC_Os08g02520.1	8
3836	LOC_Os08g02530.1	8
3836	LOC_Os08g35110.1	8
3836	LOC_Os08g42198.1	8
3836	LOC_Os08g42277.1	8
3836	LOC_Os08g43700.1	8
3836	LOC_Os09g26590.1	9
3836	LOC_Os09g26610.1	9
3836	LOC_Os09g32984.1	9
3836	LOC_Os09g37350.1	9
3836	LOC_Os09g37380.1	9
3836	LOC_Os09g37390.1	9
3836	LOC_Os09g37330.1	9
3836	LOC_Os09g37410.1	9
3836	LOC_Os09g37420.1	9
3836	LOC_Os09g37430.1	9
3836	LOC_Os09g37440.1	9
3836	LOC_Os09g37460.1	9
3836	LOC_Os09g37470.1	9
3836	LOC_Os09g37480.1	9
3836	LOC_Os09g37400.1	9
3836	LOC_Os09g37490.1	9
3836	LOC_Os09g37500.1	9
3836	LOC_Os12g43110.1	12
3837	LOC_Os01g14650.1	1
3837	LOC_Os01g14660.1	1
3837	LOC_Os01g16770.1	1

3837	LOC_Os01g60770.1	1
3837	LOC_Os02g16800.1	2
3837	LOC_Os02g16809.1	2
3837	LOC_Os02g16730.1	2
3837	LOC_Os02g16780.1	2
3837	LOC_Os02g16839.1	2
3837	LOC_Os02g16850.1	2
3837	LOC_Os02g42650.1	2
3837	LOC_Os02g44106.1	2
3837	LOC_Os02g44108.1	2
3837	LOC_Os02g51040.1	2
3837	LOC_Os03g01270.1	3
3837	LOC_Os03g01260.1	3
3837	LOC_Os03g01640.1	3
3837	LOC_Os03g01650.1	3
3837	LOC_Os03g01610.1	3
3837	LOC_Os03g01630.1	3
3837	LOC_Os03g04020.1	3
3837	LOC_Os03g06060.1	3
3837	LOC_Os03g06000.1	3
3837	LOC_Os03g06010.1	3
3837	LOC_Os03g06020.1	3
3837	LOC_Os03g06040.1	3
3837	LOC_Os03g06050.1	3
3837	LOC_Os03g21820.1	3
3837	LOC_Os03g25990.1	3
3837	LOC_Os03g31480.1	3
3837	LOC_Os03g60720.1	3
3837	LOC_Os03g44290.1	3
3837	LOC_Os04g15840.1	4
3837	LOC_Os04g46630.1	4
3837	LOC_Os04g46650.1	4
3837	LOC_Os04g49410.1	4
3837	LOC_Os05g15690.1	5
3837	LOC_Os05g19570.1	5
3837	LOC_Os05g19600.1	5
3837	LOC_Os05g39990.1	5
3837	LOC_Os06g01920.1	6
3837	LOC_Os06g41700.1	6
3837	LOC_Os06g50400.1	6
3837	LOC_Os07g29290.1	7
3837	LOC_Os08g44790.1	8
3837	LOC_Os10g30330.1	10
3837	LOC_Os10g30340.1	10
3837	LOC_Os10g39110.1	10
3837	LOC_Os10g39640.1	10
3837	LOC_Os10g40090.1	10
3837	LOC_Os10g40700.1	10
3837	LOC_Os10g40710.1	10
3837	LOC_Os10g40720.1	10
3837	LOC_Os10g40730.1	10
3837	LOC_Os12g36040.1	12

3838	LOC_Os01g12750.1	1
3838	LOC_Os01g12770.1	1
3838	LOC_Os01g12740.1	1
3838	LOC_Os01g27890.1	1
3838	LOC_Os01g38110.1	1
3838	LOC_Os01g50490.1	1
3838	LOC_Os02g09220.1	2
3838	LOC_Os02g09320.1	2
3838	LOC_Os02g09390.1	2
3838	LOC_Os02g09400.1	2
3838	LOC_Os02g09410.1	2
3838	LOC_Os02g26770.1	2
3838	LOC_Os02g26810.1	2
3838	LOC_Os02g29960.1	2
3838	LOC_Os02g30110.1	2
3838	LOC_Os02g30090.1	2
3838	LOC_Os02g30100.1	2
3838	LOC_Os02g36030.1	2
3838	LOC_Os02g36150.1	2
3838	LOC_Os02g36190.1	2
3838	LOC_Os03g04190.1	3
3838	LOC_Os03g14400.1	3
3838	LOC_Os03g14420.1	3
3838	LOC_Os03g14560.1	3
3838	LOC_Os03g37290.1	3
3838	LOC_Os03g39760.1	3
3838	LOC_Os03g44740.1	3
3838	LOC_Os03g55260.1	3
3838	LOC_Os03g55240.1	3
3838	LOC_Os04g40460.1	4
3838	LOC_Os04g40470.1	4
3838	LOC_Os05g35010.1	5
3838	LOC_Os05g43910.1	5
3838	LOC_Os06g01250.1	6
3838	LOC_Os06g22020.1	6
3838	LOC_Os06g30640.1	6
3838	LOC_Os06g41070.1	6
3838	LOC_Os06g43384.1	6
3838	LOC_Os06g43480.1	6
3838	LOC_Os06g43490.1	6
3838	LOC_Os06g43430.1	6
3838	LOC_Os07g11739.1	7
3838	LOC_Os08g01450.1	8
3838	LOC_Os08g35510.1	8
3838	LOC_Os08g43440.1	8
3838	LOC_Os09g26960.1	9
3838	LOC_Os10g08319.1	10
3838	LOC_Os10g16974.1	10
3838	LOC_Os10g17260.1	10
3838	LOC_Os10g30390.1	10
3838	LOC_Os10g30410.1	10
3838	LOC_Os11g27730.1	11

3838	LOC_Os11g41680.1	11
3838	LOC_Os11g41710.1	11
3838	LOC_Os12g32850.1	12
3838	LOC_Os12g39300.1	12
3839	LOC_Os01g19640.1	1
3839	LOC_Os01g35490.1	1
3839	LOC_Os02g07500.1	2
3839	LOC_Os02g22460.1	2
3839	LOC_Os02g49020.1	2
3839	LOC_Os03g32820.1	3
3839	LOC_Os03g33480.1	3
3839	LOC_Os03g39730.1	3
3839	LOC_Os04g01420.1	4
3839	LOC_Os04g13570.1	4
3839	LOC_Os04g16220.1	4
3839	LOC_Os04g27690.1	4
3839	LOC_Os05g15390.1	5
3839	LOC_Os05g15410.1	5
3839	LOC_Os05g26280.1	5
3839	LOC_Os05g21010.1	5
3839	LOC_Os06g15280.1	6
3839	LOC_Os06g25750.1	6
3839	LOC_Os06g34600.1	6
3839	LOC_Os06g35130.1	6
3839	LOC_Os06g41760.1	6
3839	LOC_Os07g22250.1	7
3839	LOC_Os07g23060.1	7
3839	LOC_Os07g24600.1	7
3839	LOC_Os07g24610.1	7
3839	LOC_Os07g24780.1	7
3839	LOC_Os08g02890.1	8
3839	LOC_Os08g12380.1	8
3839	LOC_Os08g19950.1	8
3839	LOC_Os08g21654.1	8
3839	LOC_Os08g30970.1	8
3839	LOC_Os09g05110.1	9
3839	LOC_Os09g06240.1	9
3839	LOC_Os09g06580.1	9
3839	LOC_Os09g29010.1	9
3839	LOC_Os10g11844.1	10
3839	LOC_Os10g15220.1	10
3839	LOC_Os10g18630.1	10
3839	LOC_Os10g20360.1	10
3839	LOC_Os10g24330.1	10
3839	LOC_Os10g26230.1	10
3839	LOC_Os11g18270.1	11
3839	LOC_Os11g19080.1	11
3839	LOC_Os11g24110.1	11
3839	LOC_Os11g25310.1	11
3839	LOC_Os11g25370.1	11
3839	LOC_Os11g27780.1	11
3839	LOC_Os11g41010.1	11

3839	LOC_Os11g44760.1	11
3839	LOC_Os11g45460.1	11
3839	LOC_Os12g10620.1	12
3839	LOC_Os12g22550.1	12
3839	LOC_Os12g24970.1	12
3839	LOC_Os12g30650.1	12
3839	LOC_Os12g42470.1	12
3839	LOC_Os12g43160.1	12
3840	LOC_Os01g09880.1	1
3840	LOC_Os01g12820.1	1
3840	LOC_Os01g13340.1	1
3840	LOC_Os01g39290.1	1
3840	LOC_Os01g53470.1	1
3840	LOC_Os01g64450.1	1
3840	LOC_Os01g64470.1	1
3840	LOC_Os01g64480.1	1
3840	LOC_Os01g68080.1	1
3840	LOC_Os01g68090.1	1
3840	LOC_Os02g01060.1	2
3840	LOC_Os02g16030.1	2
3840	LOC_Os02g16610.1	2
3840	LOC_Os02g30450.1	2
3840	LOC_Os02g33550.1	2
3840	LOC_Os02g44670.1	2
3840	LOC_Os02g44740.1	2
3840	LOC_Os03g15630.1	3
3840	LOC_Os03g62010.1	3
3840	LOC_Os03g62020.1	3
3840	LOC_Os04g33990.1	4
3840	LOC_Os04g53650.1	4
3840	LOC_Os04g58090.1	4
3840	LOC_Os04g58850.1	4
3840	LOC_Os04g58860.1	4
3840	LOC_Os05g24760.1	5
3840	LOC_Os05g11010.1	5
3840	LOC_Os05g30490.1	5
3840	LOC_Os05g40400.1	5
3840	LOC_Os05g45070.1	5
3840	LOC_Os05g50720.1	5
3840	LOC_Os06g06780.1	6
3840	LOC_Os06g32970.1	6
3840	LOC_Os06g49650.1	6
3840	LOC_Os07g14700.1	7
3840	LOC_Os07g14740.1	7
3840	LOC_Os07g14660.1	7
3840	LOC_Os07g34040.1	7
3840	LOC_Os07g34050.1	7
3840	LOC_Os07g34720.1	7
3840	LOC_Os08g01210.1	8
3840	LOC_Os08g01220.1	8
3840	LOC_Os08g23460.1	8
3840	LOC_Os08g38580.1	8

3840	LOC_Os08g44410.1	8
3840	LOC_Os09g09460.1	9
3840	LOC_Os09g36210.1	9
3840	LOC_Os10g39970.1	10
3840	LOC_Os11g02730.1	11
3840	LOC_Os11g03600.1	11
3840	LOC_Os11g05860.1	11
3840	LOC_Os11g05870.1	11
3840	LOC_Os12g02700.1	12
3840	LOC_Os12g03370.1	12
3840	LOC_Os12g06210.1	12
3840	LOC_Os12g06220.1	12
3840	LOC_Os12g06260.1	12
3841	LOC_Os01g19610.1	1
3841	LOC_Os01g21650.1	1
3841	LOC_Os01g21670.1	1
3841	LOC_Os01g21710.1	1
3841	LOC_Os01g38120.1	1
3841	LOC_Os01g38190.1	1
3841	LOC_Os01g38380.1	1
3841	LOC_Os01g38430.1	1
3841	LOC_Os02g15500.1	2
3841	LOC_Os03g19700.1	3
3841	LOC_Os03g39520.1	3
3841	LOC_Os03g06730.1	3
3841	LOC_Os04g42650.1	4
3841	LOC_Os04g55390.1	4
3841	LOC_Os04g55370.1	4
3841	LOC_Os05g03972.1	5
3841	LOC_Os05g04020.1	5
3841	LOC_Os05g04060.1	5
3841	LOC_Os05g10900.1	5
3841	LOC_Os05g10920.1	5
3841	LOC_Os05g15230.1	5
3841	LOC_Os06g06550.1	6
3841	LOC_Os06g08120.1	6
3841	LOC_Os06g33350.1	6
3841	LOC_Os08g02710.1	8
3841	LOC_Os08g02730.1	8
3841	LOC_Os08g02820.1	8
3841	LOC_Os08g02830.1	8
3841	LOC_Os08g07040.1	8
3841	LOC_Os08g25030.1	8
3841	LOC_Os08g26220.1	8
3841	LOC_Os08g26710.1	8
3841	LOC_Os08g26820.1	8
3841	LOC_Os08g26840.1	8
3841	LOC_Os08g26850.1	8
3841	LOC_Os08g30520.1	8
3841	LOC_Os08g42570.1	8
3841	LOC_Os09g08160.1	9
3841	LOC_Os09g09550.1	9

3841	LOC_Os09g12970.1	9
3841	LOC_Os09g13040.1	9
3841	LOC_Os09g12840.1	9
3841	LOC_Os09g13470.1	9
3841	LOC_Os09g13410.1	9
3841	LOC_Os09g36870.1	9
3841	LOC_Os10g31170.1	10
3841	LOC_Os10g31240.1	10
3841	LOC_Os11g17720.1	11
3841	LOC_Os11g33394.1	11
3841	LOC_Os11g33430.1	11
3841	LOC_Os11g33942.1	11
3841	LOC_Os11g33970.1	11
3841	LOC_Os11g34090.1	11
3841	LOC_Os11g33450.1	11
3841	LOC_Os12g29650.1	12
3841	LOC_Os12g29620.1	12
3841	LOC_Os12g32920.1	12
3841	LOC_Os12g32880.1	12
3841	LOC_Os12g32900.1	12
3842	LOC_Os01g09660.1	1
3842	LOC_Os01g14710.1	1
3842	LOC_Os01g20830.1	1
3842	LOC_Os01g32330.1	1
3842	LOC_Os01g41200.1	1
3842	LOC_Os01g48710.1	1
3842	LOC_Os01g52160.1	1
3842	LOC_Os01g55320.1	1
3842	LOC_Os01g61070.1	1
3842	LOC_Os01g70710.1	1
3842	LOC_Os01g74490.1	1
3842	LOC_Os01g70240.1	1
3842	LOC_Os02g30650.1	2
3842	LOC_Os02g37160.1	2
3842	LOC_Os02g37280.1	2
3842	LOC_Os02g37300.1	2
3842	LOC_Os02g37320.1	2
3842	LOC_Os02g37330.1	2
3842	LOC_Os02g37290.1	2
3842	LOC_Os02g57350.1	2
3842	LOC_Os02g57360.1	2
3842	LOC_Os03g02070.1	3
3842	LOC_Os03g02860.1	3
3842	LOC_Os03g03500.1	3
3842	LOC_Os03g06080.1	3
3842	LOC_Os03g22490.1	3
3842	LOC_Os03g26650.1	3
3842	LOC_Os03g27040.1	3
3842	LOC_Os03g60480.1	3
3842	LOC_Os03g64340.1	3
3842	LOC_Os04g17100.1	4
3842	LOC_Os04g32030.1	4

3842	LOC_Os04g39010.1	4
3842	LOC_Os04g39290.1	4
3842	LOC_Os04g39300.1	4
3842	LOC_Os04g39360.1	4
3842	LOC_Os04g39370.1	4
3842	LOC_Os04g39350.1	4
3842	LOC_Os04g42350.1	4
3842	LOC_Os04g45130.1	4
3842	LOC_Os04g49260.1	4
3842	LOC_Os04g52530.1	4
3842	LOC_Os04g56570.1	4
3842	LOC_Os04g57200.1	4
3842	LOC_Os05g27100.1	5
3842	LOC_Os05g45820.1	5
3842	LOC_Os06g35060.1	6
3842	LOC_Os07g20340.1	7
3842	LOC_Os07g47480.1	7
3842	LOC_Os07g43040.1	7
3842	LOC_Os08g10480.1	8
3842	LOC_Os08g10490.1	8
3842	LOC_Os08g31140.1	8
3842	LOC_Os08g40130.1	8
3842	LOC_Os09g09850.1	9
3842	LOC_Os09g09930.1	9
3842	LOC_Os09g09830.1	9
3842	LOC_Os09g20000.1	9
3842	LOC_Os10g14870.1	10
3842	LOC_Os10g30430.1	10
3842	LOC_Os10g36200.1	10
3842	LOC_Os10g38870.1	10
3842	LOC_Os10g39210.1	10
3843	LOC_Os01g04190.1	1
3843	LOC_Os01g38670.1	1
3843	LOC_Os01g38680.1	1
3843	LOC_Os01g73590.1	1
3843	LOC_Os02g06540.1	2
3843	LOC_Os02g13560.1	2
3843	LOC_Os02g17500.1	2
3843	LOC_Os02g36450.1	2
3843	LOC_Os02g36414.1	2
3843	LOC_Os02g36440.1	2
3843	LOC_Os02g58530.1	2
3843	LOC_Os03g01170.1	3
3843	LOC_Os03g03680.1	3
3843	LOC_Os03g10090.1	3
3843	LOC_Os03g10100.1	3
3843	LOC_Os03g11900.1	3
3843	LOC_Os03g24860.1	3
3843	LOC_Os03g24870.1	3
3843	LOC_Os03g39710.1	3
3843	LOC_Os03g43720.1	3
3843	LOC_Os03g60820.1	3

3843	LOC_Os04g37980.1	4
3843	LOC_Os04g37990.1	4
3843	LOC_Os04g38010.1	4
3843	LOC_Os04g38026.1	4
3843	LOC_Os04g37970.1	4
3843	LOC_Os04g38220.1	4
3843	LOC_Os04g41460.1	4
3843	LOC_Os04g43210.1	4
3843	LOC_Os04g44750.1	4
3843	LOC_Os04g58220.1	4
3843	LOC_Os04g58230.1	4
3843	LOC_Os05g37820.1	5
3843	LOC_Os05g49260.1	5
3843	LOC_Os05g49270.1	5
3843	LOC_Os05g50280.1	5
3843	LOC_Os06g04900.1	6
3843	LOC_Os07g01560.1	7
3843	LOC_Os07g03960.1	7
3843	LOC_Os07g05640.1	7
3843	LOC_Os07g10590.1	7
3843	LOC_Os07g37320.1	7
3843	LOC_Os07g38400.1	7
3843	LOC_Os07g39350.1	7
3843	LOC_Os07g39360.1	7
3843	LOC_Os08g08070.1	8
3843	LOC_Os09g09520.1	9
3843	LOC_Os09g12590.1	9
3843	LOC_Os09g15330.1	9
3843	LOC_Os09g23110.1	9
3843	LOC_Os09g24924.1	9
3843	LOC_Os09g38690.1	9
3843	LOC_Os09g39680.1	9
3843	LOC_Os10g21590.1	10
3843	LOC_Os10g39440.1	10
3843	LOC_Os10g41190.1	10
3843	LOC_Os10g42830.1	10
3843	LOC_Os11g28610.1	11
3843	LOC_Os11g38160.1	11
3843	LOC_Os11g40540.1	11
3843	LOC_Os11g41840.1	11
3843	LOC_Os11g41850.1	11
3843	LOC_Os12g32760.1	12
3843	LOC_Os12g32940.1	12
3844	LOC_Os01g01160.1	1
3844	LOC_Os01g16680.1	1
3844	LOC_Os01g17030.1	1
3844	LOC_Os01g17040.1	1
3844	LOC_Os01g32870.1	1
3844	LOC_Os01g33800.1	1
3844	LOC_Os01g37560.1	1
3844	LOC_Os01g42190.1	1
3844	LOC_Os01g70250.1	1

3844	LOC_Os01g06454.1	1
3844	LOC_Os02g10220.1	2
3844	LOC_Os02g46640.1	2
3844	LOC_Os02g50760.1	2
3844	LOC_Os02g52270.1	2
3844	LOC_Os02g54130.1	2
3844	LOC_Os03g15480.1	3
3844	LOC_Os03g18870.1	3
3844	LOC_Os03g27460.1	3
3844	LOC_Os03g51830.1	3
3844	LOC_Os03g55360.1	3
3844	LOC_Os03g56540.1	3
3844	LOC_Os03g61550.1	3
3844	LOC_Os03g61730.1	3
3844	LOC_Os03g62140.1	3
3844	LOC_Os03g62150.1	3
3844	LOC_Os03g62120.1	3
3844	LOC_Os03g62130.1	3
3844	LOC_Os03g18200.1	3
3844	LOC_Os03g60790.1	3
3844	LOC_Os04g57880.1	4
3844	LOC_Os04g59060.1	4
3844	LOC_Os05g01590.1	5
3844	LOC_Os05g30130.1	5
3844	LOC_Os05g33010.1	5
3844	LOC_Os06g09560.1	6
3844	LOC_Os06g13060.1	6
3844	LOC_Os06g30310.1	6
3844	LOC_Os06g44160.1	6
3844	LOC_Os07g03270.1	7
3844	LOC_Os07g09450.1	7
3844	LOC_Os07g28800.1	7
3844	LOC_Os07g42800.1	7
3844	LOC_Os07g43330.1	7
3844	LOC_Os07g43870.1	7
3844	LOC_Os07g44310.1	7
3844	LOC_Os08g03380.1	8
3844	LOC_Os08g32130.1	8
3844	LOC_Os08g35160.1	8
3844	LOC_Os08g37270.1	8
3844	LOC_Os08g43490.1	8
3844	LOC_Os09g20320.1	9
3844	LOC_Os10g03610.1	10
3844	LOC_Os10g33790.1	10
3844	LOC_Os10g36370.1	10
3844	LOC_Os10g42439.1	10
3844	LOC_Os11g10990.1	11
3844	LOC_Os11g36530.1	11
3844	LOC_Os11g37000.1	11
3844	LOC_Os12g15590.1	12
3844	LOC_Os12g27070.1	12
3844	LOC_Os12g31460.1	12

3844	LOC_Os12g31840.1	12
3844	LOC_Os12g41820.1	12
3844	LOC_Os12g44260.1	12
3845	LOC_Os01g04180.1	1
3845	LOC_Os01g05770.1	1
3845	LOC_Os01g26250.1	1
3845	LOC_Os01g35110.1	1
3845	LOC_Os02g08050.1	2
3845	LOC_Os02g27910.1	2
3845	LOC_Os02g35550.1	2
3845	LOC_Os02g38150.1	2
3845	LOC_Os02g42930.1	2
3845	LOC_Os02g50540.1	2
3845	LOC_Os02g55160.1	2
3845	LOC_Os03g07270.1	3
3845	LOC_Os03g19880.1	3
3845	LOC_Os03g31700.1	3
3845	LOC_Os03g32200.1	3
3845	LOC_Os04g08570.1	4
3845	LOC_Os04g28980.1	4
3845	LOC_Os04g31670.1	4
3845	LOC_Os04g45980.1	4
3845	LOC_Os04g46030.1	4
3845	LOC_Os04g56000.1	4
3845	LOC_Os04g57710.1	4
3845	LOC_Os05g13760.1	5
3845	LOC_Os05g38440.1	5
3845	LOC_Os05g41870.1	5
3845	LOC_Os05g48710.1	5
3845	LOC_Os06g02710.1	6
3845	LOC_Os06g16120.1	6
3845	LOC_Os06g22170.1	6
3845	LOC_Os06g22830.1	6
3845	LOC_Os06g26170.1	6
3845	LOC_Os06g51300.1	6
3845	LOC_Os07g20260.1	7
3845	LOC_Os07g26750.1	7
3845	LOC_Os08g09680.1	8
3845	LOC_Os08g26310.1	8
3845	LOC_Os08g27660.1	8
3845	LOC_Os09g14890.1	9
3845	LOC_Os09g16610.1	9
3845	LOC_Os09g25690.1	9
3845	LOC_Os09g25710.1	9
3845	LOC_Os09g25720.1	9
3845	LOC_Os09g27880.1	9
3845	LOC_Os09g36840.1	9
3845	LOC_Os10g04870.1	10
3845	LOC_Os10g27310.1	10
3845	LOC_Os10g28550.1	10
3845	LOC_Os10g31380.1	10
3845	LOC_Os10g31460.1	10

3845	LOC_Os10g31510.1	10
3845	LOC_Os10g31620.1	10
3845	LOC_Os10g31540.1	10
3845	LOC_Os10g31690.1	10
3845	LOC_Os10g31640.1	10
3845	LOC_Os10g31710.1	10
3845	LOC_Os10g31740.1	10
3845	LOC_Os11g07620.1	11
3845	LOC_Os11g11030.1	11
3845	LOC_Os11g15230.1	11
3845	LOC_Os11g30930.1	11
3845	LOC_Os11g37530.1	11
3845	LOC_Os11g40270.1	11
3845	LOC_Os11g14120.1	11
3845	LOC_Os12g27840.1	12
3845	LOC_Os12g41480.1	12
3845	LOC_Os12g43200.1	12
3846	LOC_Os01g21000.1	1
3846	LOC_Os01g23560.1	1
3846	LOC_Os01g66780.1	1
3846	LOC_Os02g10330.1	2
3846	LOC_Os02g17580.1	2
3846	LOC_Os02g17440.1	2
3846	LOC_Os02g18280.1	2
3846	LOC_Os02g30500.1	2
3846	LOC_Os03g33110.1	3
3846	LOC_Os03g35970.1	3
3846	LOC_Os03g52510.1	3
3846	LOC_Os04g03440.1	4
3846	LOC_Os04g11470.1	4
3846	LOC_Os04g16190.1	4
3846	LOC_Os04g29120.1	4
3846	LOC_Os04g58440.1	4
3846	LOC_Os05g20380.1	5
3846	LOC_Os05g24030.1	5
3846	LOC_Os05g28670.1	5
3846	LOC_Os05g41710.1	5
3846	LOC_Os05g34360.1	5
3846	LOC_Os05g47460.1	5
3846	LOC_Os06g18750.1	6
3846	LOC_Os06g25530.1	6
3846	LOC_Os06g29170.1	6
3846	LOC_Os06g29930.1	6
3846	LOC_Os06g38140.1	6
3846	LOC_Os06g39300.1	6
3846	LOC_Os07g14570.1	7
3846	LOC_Os07g16070.1	7
3846	LOC_Os07g17860.1	7
3846	LOC_Os07g18380.1	7
3846	LOC_Os07g27530.1	7
3846	LOC_Os07g30060.1	7
3846	LOC_Os07g37870.1	7

3846	LOC_Os08g03940.1	8
3846	LOC_Os08g08560.1	8
3846	LOC_Os08g11170.1	8
3846	LOC_Os08g11400.1	8
3846	LOC_Os08g15680.1	8
3846	LOC_Os08g16520.1	8
3846	LOC_Os08g17450.1	8
3846	LOC_Os08g22440.1	8
3846	LOC_Os08g23450.1	8
3846	LOC_Os08g39750.1	8
3846	LOC_Os08g39770.1	8
3846	LOC_Os08g40700.1	8
3846	LOC_Os09g03270.1	9
3846	LOC_Os09g28980.1	9
3846	LOC_Os10g03190.1	10
3846	LOC_Os10g03200.1	10
3846	LOC_Os10g06210.1	10
3846	LOC_Os10g15260.1	10
3846	LOC_Os10g20210.1	10
3846	LOC_Os11g11280.1	11
3846	LOC_Os11g22170.1	11
3846	LOC_Os11g22180.1	11
3846	LOC_Os11g19930.1	11
3846	LOC_Os11g20010.1	11
3846	LOC_Os11g25820.1	11
3846	LOC_Os11g27090.1	11
3846	LOC_Os11g39150.1	11
3846	LOC_Os11g41270.1	11
3846	LOC_Os11g41460.1	11
3846	LOC_Os11g47448.1	11
3846	LOC_Os11g47449.1	11
3847	LOC_Os01g19130.1	1
3847	LOC_Os01g37130.1	1
3847	LOC_Os01g40094.1	1
3847	LOC_Os01g43100.1	1
3847	LOC_Os01g46760.1	1
3847	LOC_Os01g62760.1	1
3847	LOC_Os01g74530.1	1
3847	LOC_Os02g05630.1	2
3847	LOC_Os02g13100.1	2
3847	LOC_Os02g15594.1	2
3847	LOC_Os02g27220.1	2
3847	LOC_Os02g38580.1	2
3847	LOC_Os02g38780.1	2
3847	LOC_Os02g38710.1	2
3847	LOC_Os02g39480.1	2
3847	LOC_Os02g46080.1	2
3847	LOC_Os02g46490.1	2
3847	LOC_Os02g55560.1	2
3847	LOC_Os02g39410.1	2
3847	LOC_Os03g10950.1	3
3847	LOC_Os03g04430.1	3

3847	LOC_Os03g16170.1	3
3847	LOC_Os03g16760.1	3
3847	LOC_Os03g18150.1	3
3847	LOC_Os03g25600.1	3
3847	LOC_Os03g55320.1	3
3847	LOC_Os03g60650.1	3
3847	LOC_Os03g61690.1	3
3847	LOC_Os03g09220.1	3
3847	LOC_Os04g08560.1	4
3847	LOC_Os04g25570.1	4
3847	LOC_Os04g33080.1	4
3847	LOC_Os04g37904.1	4
3847	LOC_Os04g42260.1	4
3847	LOC_Os04g49490.1	4
3847	LOC_Os04g52000.1	4
3847	LOC_Os04g56450.1	4
3847	LOC_Os05g02110.1	5
3847	LOC_Os05g04360.1	5
3847	LOC_Os05g29030.1	5
3847	LOC_Os05g38290.1	5
3847	LOC_Os05g46040.1	5
3847	LOC_Os05g49730.1	5
3847	LOC_Os05g50970.1	5
3847	LOC_Os05g51510.1	5
3847	LOC_Os06g08140.1	6
3847	LOC_Os06g33530.1	6
3847	LOC_Os06g33549.1	6
3847	LOC_Os06g39600.1	6
3847	LOC_Os06g44210.1	6
3847	LOC_Os06g48300.1	6
3847	LOC_Os06g50380.1	6
3847	LOC_Os07g02330.1	7
3847	LOC_Os07g32380.1	7
3847	LOC_Os07g33230.1	7
3847	LOC_Os07g37890.1	7
3847	LOC_Os07g45170.1	7
3847	LOC_Os08g39100.1	8
3847	LOC_Os09g14540.1	9
3847	LOC_Os09g15670.1	9
3847	LOC_Os09g38550.1	9
3847	LOC_Os10g22460.1	10
3847	LOC_Os10g39540.1	10
3847	LOC_Os10g39780.1	10
3847	LOC_Os11g01790.1	11
3847	LOC_Os11g13820.1	11
3847	LOC_Os11g22404.1	11
3847	LOC_Os12g09640.1	12
3847	LOC_Os12g39120.1	12
3848	LOC_Os01g18770.1	1
3848	LOC_Os01g22620.1	1
3848	LOC_Os01g27910.1	1
3848	LOC_Os01g38070.1	1

3848	LOC_Os01g60090.1	1
3848	LOC_Os02g24480.1	2
3848	LOC_Os02g31270.1	2
3848	LOC_Os02g35520.1	2
3848	LOC_Os02g38830.1	2
3848	LOC_Os02g42760.1	2
3848	LOC_Os02g56730.1	2
3848	LOC_Os03g38310.1	3
3848	LOC_Os03g51850.1	3
3848	LOC_Os04g05020.1	4
3848	LOC_Os04g06640.1	4
3848	LOC_Os04g18840.1	4
3848	LOC_Os04g19490.1	4
3848	LOC_Os04g18780.1	4
3848	LOC_Os04g19710.1	4
3848	LOC_Os04g25070.1	4
3848	LOC_Os04g25480.1	4
3848	LOC_Os04g26030.1	4
3848	LOC_Os04g26540.1	4
3848	LOC_Os04g56020.1	4
3848	LOC_Os05g19940.1	5
3848	LOC_Os05g30830.1	5
3848	LOC_Os05g30840.1	5
3848	LOC_Os05g20760.1	5
3848	LOC_Os05g23090.1	5
3848	LOC_Os05g26550.1	5
3848	LOC_Os06g14430.1	6
3848	LOC_Os06g14940.1	6
3848	LOC_Os06g16700.1	6
3848	LOC_Os06g21420.1	6
3848	LOC_Os06g21660.1	6
3848	LOC_Os06g29330.1	6
3848	LOC_Os07g11500.1	7
3848	LOC_Os07g14320.1	7
3848	LOC_Os07g16090.1	7
3848	LOC_Os07g18790.1	7
3848	LOC_Os07g29210.1	7
3848	LOC_Os07g40530.1	7
3848	LOC_Os08g03110.1	8
3848	LOC_Os08g03980.1	8
3848	LOC_Os08g05260.1	8
3848	LOC_Os08g05990.1	8
3848	LOC_Os08g13120.1	8
3848	LOC_Os08g13240.1	8
3848	LOC_Os08g13600.1	8
3848	LOC_Os08g14280.1	8
3848	LOC_Os08g17530.1	8
3848	LOC_Os08g22210.1	8
3848	LOC_Os08g22560.1	8
3848	LOC_Os08g32730.1	8
3848	LOC_Os08g41920.1	8
3848	LOC_Os09g14750.1	9

3848	LOC_Os09g21200.1	9
3848	LOC_Os09g22010.1	9
3848	LOC_Os09g25300.1	9
3848	LOC_Os09g27640.1	9
3848	LOC_Os10g09880.1	10
3848	LOC_Os10g11150.1	10
3848	LOC_Os10g22810.1	10
3848	LOC_Os11g12250.1	11
3848	LOC_Os11g43130.1	11
3848	LOC_Os12g11430.1	12
3848	LOC_Os12g12540.1	12
3848	LOC_Os12g19830.1	12
3848	LOC_Os12g27320.1	12
3848	LOC_Os12g31210.1	12
3848	LOC_Os12g36370.1	12
3849	LOC_Os01g05540.1	1
3849	LOC_Os01g05560.1	1
3849	LOC_Os01g12330.1	1
3849	LOC_Os01g68210.1	1
3849	LOC_Os01g70280.1	1
3849	LOC_Os01g70290.1	1
3849	LOC_Os02g01790.1	2
3849	LOC_Os02g01870.1	2
3849	LOC_Os02g01900.1	2
3849	LOC_Os02g04420.1	2
3849	LOC_Os02g04610.1	2
3849	LOC_Os02g04850.1	2
3849	LOC_Os02g05000.1	2
3849	LOC_Os02g05070.1	2
3849	LOC_Os02g05080.1	2
3849	LOC_Os02g05120.1	2
3849	LOC_Os02g05350.1	2
3849	LOC_Os02g05360.1	2
3849	LOC_Os02g05430.1	2
3849	LOC_Os02g04790.1	2
3849	LOC_Os02g04900.1	2
3849	LOC_Os02g05190.1	2
3849	LOC_Os02g05810.1	2
3849	LOC_Os02g10450.1	2
3849	LOC_Os02g10460.1	2
3849	LOC_Os02g30940.1	2
3849	LOC_Os03g01420.1	3
3849	LOC_Os03g01490.1	3
3849	LOC_Os03g05370.1	3
3849	LOC_Os03g06750.1	3
3849	LOC_Os03g08690.1	3
3849	LOC_Os03g11270.1	3
3849	LOC_Os03g13340.1	3
3849	LOC_Os03g13984.1	3
3849	LOC_Os03g43340.1	3
3849	LOC_Os03g43350.1	3
3849	LOC_Os03g56724.1	3

3849	LOC_Os03g56904.1	3
3849	LOC_Os03g60310.1	3
3849	LOC_Os04g13460.1	4
3849	LOC_Os04g34550.1	4
3849	LOC_Os04g34900.1	4
3849	LOC_Os04g58030.1	4
3849	LOC_Os04g58050.1	4
3849	LOC_Os05g03490.1	5
3849	LOC_Os05g03520.1	5
3849	LOC_Os05g23450.1	5
3849	LOC_Os06g08990.1	6
3849	LOC_Os06g09020.1	6
3849	LOC_Os07g13030.1	7
3849	LOC_Os07g13090.1	7
3849	LOC_Os07g10880.1	7
3849	LOC_Os07g32920.1	7
3849	LOC_Os07g45340.1	7
3849	LOC_Os07g49050.1	7
3849	LOC_Os08g06810.1	8
3849	LOC_Os08g06830.1	8
3849	LOC_Os09g33890.1	9
3849	LOC_Os09g38360.1	9
3849	LOC_Os10g08610.1	10
3849	LOC_Os10g19230.1	10
3849	LOC_Os11g02220.1	11
3849	LOC_Os11g02230.1	11
3849	LOC_Os11g04760.1	11
3849	LOC_Os12g01880.1	12
3849	LOC_Os12g02160.1	12
3849	LOC_Os12g02170.1	12
3849	LOC_Os12g02180.1	12
3849	LOC_Os12g04540.1	12
3849	LOC_Os12g28060.1	12
3849	LOC_Os12g28090.1	12
3849	LOC_Os12g42920.1	12
3849	LOC_Os12g42950.1	12
3850	LOC_Os01g05860.1	1
3850	LOC_Os01g07610.1	1
3850	LOC_Os01g17320.1	1
3850	LOC_Os01g32090.1	1
3850	LOC_Os01g36600.1	1
3850	LOC_Os01g37870.1	1
3850	LOC_Os01g48380.1	1
3850	LOC_Os01g54380.1	1
3850	LOC_Os01g57900.1	1
3850	LOC_Os01g60010.1	1
3850	LOC_Os01g67210.1	1
3850	LOC_Os01g55290.1	1
3850	LOC_Os02g02590.1	2
3850	LOC_Os02g02770.1	2
3850	LOC_Os02g03530.1	2
3850	LOC_Os02g03970.1	2

3850	LOC_Os02g16650.1	2
3850	LOC_Os02g17360.1	2
3850	LOC_Os02g20160.1	2
3850	LOC_Os02g21070.1	2
3850	LOC_Os02g39820.1	2
3850	LOC_Os02g43080.1	2
3850	LOC_Os02g45590.1	2
3850	LOC_Os02g47360.1	2
3850	LOC_Os02g51480.1	2
3850	LOC_Os02g52220.1	2
3850	LOC_Os02g48122.1	2
3850	LOC_Os03g02430.1	3
3850	LOC_Os03g02762.1	3
3850	LOC_Os03g06370.1	3
3850	LOC_Os03g06710.1	3
3850	LOC_Os03g11310.1	3
3850	LOC_Os03g07220.1	3
3850	LOC_Os03g17634.1	3
3850	LOC_Os03g18620.1	3
3850	LOC_Os03g50500.1	3
3850	LOC_Os03g56960.1	3
3850	LOC_Os03g58120.1	3
3850	LOC_Os03g60910.1	3
3850	LOC_Os03g63910.1	3
3850	LOC_Os04g21470.1	4
3850	LOC_Os04g40010.1	4
3850	LOC_Os04g44030.1	4
3850	LOC_Os04g49350.1	4
3850	LOC_Os04g52290.1	4
3850	LOC_Os04g55090.1	4
3850	LOC_Os05g05320.1	5
3850	LOC_Os05g22870.1	5
3850	LOC_Os05g25060.1	5
3850	LOC_Os05g40320.1	5
3850	LOC_Os05g24270.1	5
3850	LOC_Os05g33760.1	5
3850	LOC_Os06g02120.1	6
3850	LOC_Os06g08570.1	6
3850	LOC_Os06g20354.1	6
3850	LOC_Os06g31300.1	6
3850	LOC_Os06g44820.1	6
3850	LOC_Os07g28900.1	7
3850	LOC_Os07g31310.1	7
3850	LOC_Os07g36450.1	7
3850	LOC_Os07g40800.1	7
3850	LOC_Os07g40820.1	7
3850	LOC_Os07g41260.1	7
3850	LOC_Os07g42354.1	7
3850	LOC_Os07g46730.1	7
3850	LOC_Os07g47720.1	7
3850	LOC_Os07g12140.1	7
3850	LOC_Os08g06500.1	8

3850	LOC_Os08g17080.1	8
3850	LOC_Os08g28180.1	8
3850	LOC_Os08g31110.1	8
3850	LOC_Os09g09450.1	9
3850	LOC_Os09g29790.1	9
3850	LOC_Os10g23830.1	10
3850	LOC_Os10g28600.1	10
3850	LOC_Os10g28640.1	10
3850	LOC_Os10g33874.1	10
3850	LOC_Os10g36190.1	10
3850	LOC_Os10g42760.1	10
3850	LOC_Os11g03850.1	11
3850	LOC_Os11g43800.1	11
3850	LOC_Os12g07260.1	12
3850	LOC_Os12g27060.1	12
3851	LOC_Os01g01470.1	1
3851	LOC_Os01g15640.1	1
3851	LOC_Os01g29840.1	1
3851	LOC_Os01g48130.1	1
3851	LOC_Os01g48460.1	1
3851	LOC_Os01g59640.1	1
3851	LOC_Os01g64310.1	1
3851	LOC_Os01g66490.1	1
3851	LOC_Os01g09550.1	1
3851	LOC_Os02g06950.1	2
3851	LOC_Os02g12310.1	2
3851	LOC_Os02g15340.1	2
3851	LOC_Os02g34970.1	2
3851	LOC_Os02g38130.1	2
3851	LOC_Os02g41450.1	2
3851	LOC_Os02g42970.1	2
3851	LOC_Os02g51120.1	2
3851	LOC_Os02g56600.1	2
3851	LOC_Os03g01870.1	3
3851	LOC_Os03g02800.1	3
3851	LOC_Os03g03540.1	3
3851	LOC_Os03g04070.1	3
3851	LOC_Os03g12120.1	3
3851	LOC_Os03g21030.1	3
3851	LOC_Os03g56580.1	3
3851	LOC_Os03g62470.1	3
3851	LOC_Os04g35660.1	4
3851	LOC_Os04g38720.1	4
3851	LOC_Os04g43560.1	4
3851	LOC_Os04g52810.1	4
3851	LOC_Os04g59470.1	4
3851	LOC_Os05g10620.1	5
3851	LOC_Os05g34310.1	5
3851	LOC_Os05g35170.1	5
3851	LOC_Os05g34600.1	5
3851	LOC_Os05g43960.1	5
3851	LOC_Os05g48850.1	5

3851	LOC_Os06g01480.1	6
3851	LOC_Os06g04090.1	6
3851	LOC_Os06g15690.1	6
3851	LOC_Os06g23650.1	6
3851	LOC_Os06g36480.1	6
3851	LOC_Os06g46270.1	6
3851	LOC_Os06g51070.1	6
3851	LOC_Os06g01230.1	6
3851	LOC_Os07g04560.1	7
3851	LOC_Os07g37920.1	7
3851	LOC_Os07g48550.1	7
3851	LOC_Os07g48450.1	7
3851	LOC_Os08g01330.1	8
3851	LOC_Os08g02160.1	8
3851	LOC_Os08g02300.1	8
3851	LOC_Os08g10080.1	8
3851	LOC_Os08g33670.1	8
3851	LOC_Os08g33910.1	8
3851	LOC_Os08g42400.1	8
3851	LOC_Os08g44820.1	8
3851	LOC_Os08g06140.1	8
3851	LOC_Os09g12380.1	9
3851	LOC_Os09g32040.1	9
3851	LOC_Os09g32260.1	9
3851	LOC_Os09g33490.1	9
3851	LOC_Os09g38000.1	9
3851	LOC_Os09g38010.1	9
3851	LOC_Os10g09820.1	10
3851	LOC_Os10g21560.1	10
3851	LOC_Os10g25640.1	10
3851	LOC_Os10g26240.1	10
3851	LOC_Os10g27390.1	10
3851	LOC_Os10g27360.1	10
3851	LOC_Os10g38834.1	10
3851	LOC_Os10g42130.1	10
3851	LOC_Os11g03310.1	11
3851	LOC_Os11g03370.1	11
3851	LOC_Os11g04960.1	11
3851	LOC_Os11g05614.1	11
3851	LOC_Os11g08210.1	11
3851	LOC_Os12g03040.1	12
3851	LOC_Os12g03050.1	12
3851	LOC_Os12g07790.1	12
3851	LOC_Os12g22940.1	12
3851	LOC_Os12g23090.1	12
3851	LOC_Os12g29330.1	12
3851	LOC_Os12g43530.1	12
3852	LOC_Os01g01520.1	1
3852	LOC_Os01g08380.1	1
3852	LOC_Os01g09010.1	1
3852	LOC_Os01g15669.1	1
3852	LOC_Os01g15709.1	1

3852	LOC_Os01g18620.1	1
3852	LOC_Os01g18744.1	1
3852	LOC_Os01g28474.1	1
3852	LOC_Os01g42870.1	1
3852	LOC_Os01g42880.1	1
3852	LOC_Os01g63480.1	1
3852	LOC_Os02g28220.1	2
3852	LOC_Os02g28240.1	2
3852	LOC_Os02g28410.1	2
3852	LOC_Os02g28170.1	2
3852	LOC_Os02g28200.1	2
3852	LOC_Os02g28300.1	2
3852	LOC_Os02g28340.1	2
3852	LOC_Os02g28470.1	2
3852	LOC_Os02g39850.1	2
3852	LOC_Os02g43670.1	2
3852	LOC_Os02g57480.1	2
3852	LOC_Os03g08720.1	3
3852	LOC_Os03g47860.1	3
3852	LOC_Os03g53360.1	3
3852	LOC_Os04g09260.1	4
3852	LOC_Os04g09590.1	4
3852	LOC_Os04g30570.1	4
3852	LOC_Os04g42250.1	4
3852	LOC_Os04g51660.1	4
3852	LOC_Os04g52164.1	4
3852	LOC_Os04g54560.1	4
3852	LOC_Os04g54570.1	4
3852	LOC_Os04g56900.1	4
3852	LOC_Os04g56910.1	4
3852	LOC_Os05g02590.1	5
3852	LOC_Os05g04584.1	5
3852	LOC_Os05g08640.1	5
3852	LOC_Os05g19910.1	5
3852	LOC_Os05g37660.1	5
3852	LOC_Os06g01350.1	6
3852	LOC_Os06g05284.1	6
3852	LOC_Os06g05300.1	6
3852	LOC_Os06g05310.1	6
3852	LOC_Os06g05320.1	6
3852	LOC_Os06g06180.1	6
3852	LOC_Os06g08580.1	6
3852	LOC_Os06g08640.1	6
3852	LOC_Os06g39390.1	6
3852	LOC_Os06g39470.1	6
3852	LOC_Os06g48560.1	6
3852	LOC_Os07g04970.1	7
3852	LOC_Os07g23150.1	7
3852	LOC_Os08g01980.1	8
3852	LOC_Os08g01950.1	8
3852	LOC_Os08g01960.1	8
3852	LOC_Os08g02020.1	8

3852	LOC_Os08g07720.1	8
3852	LOC_Os08g07730.1	8
3852	LOC_Os08g43020.1	8
3852	LOC_Os08g43040.1	8
3852	LOC_Os08g44840.1	8
3852	LOC_Os08g02030.1	8
3852	LOC_Os09g37180.1	9
3852	LOC_Os09g37200.1	9
3852	LOC_Os10g01650.1	10
3852	LOC_Os10g01680.1	10
3852	LOC_Os10g01920.1	10
3852	LOC_Os10g01800.1	10
3852	LOC_Os10g02000.1	10
3852	LOC_Os10g01930.1	10
3852	LOC_Os10g03360.1	10
3852	LOC_Os10g03390.1	10
3852	LOC_Os10g04400.1	10
3852	LOC_Os10g23310.1	10
3852	LOC_Os10g23820.1	10
3852	LOC_Os10g26290.1	10
3852	LOC_Os11g07960.1	11
3852	LOC_Os11g13970.1	11
3852	LOC_Os11g16680.1	11
3852	LOC_Os11g31090.1	11
3852	LOC_Os11g42370.1	11
3852	LOC_Os11g42290.1	11
3852	LOC_Os12g04080.1	12
3852	LOC_Os12g27220.1	12
3852	LOC_Os12g27254.1	12
3852	LOC_Os12g27330.1	12
3853	LOC_Os01g01740.1	1
3853	LOC_Os01g02320.1	1
3853	LOC_Os01g10450.1	1
3853	LOC_Os01g19160.1	1
3853	LOC_Os01g45380.1	1
3853	LOC_Os01g48330.1	1
3853	LOC_Os01g54350.1	1
3853	LOC_Os01g64490.1	1
3853	LOC_Os01g66020.1	1
3853	LOC_Os01g09580.1	1
3853	LOC_Os02g02040.1	2
3853	LOC_Os02g12440.1	2
3853	LOC_Os02g12810.1	2
3853	LOC_Os02g14530.1	2
3853	LOC_Os02g32610.1	2
3853	LOC_Os02g35010.1	2
3853	LOC_Os02g38080.1	2
3853	LOC_Os02g43430.1	2
3853	LOC_Os02g44642.1	2
3853	LOC_Os02g45750.1	2
3853	LOC_Os02g47220.1	2
3853	LOC_Os02g50970.1	2

3853	LOC_Os02g53040.1	2
3853	LOC_Os02g54600.1	2
3853	LOC_Os03g02980.1	3
3853	LOC_Os03g06410.1	3
3853	LOC_Os03g15570.1	3
3853	LOC_Os03g16130.1	3
3853	LOC_Os03g29410.1	3
3853	LOC_Os03g39150.1	3
3853	LOC_Os03g43760.1	3
3853	LOC_Os03g47470.1	3
3853	LOC_Os03g49640.1	3
3853	LOC_Os03g50330.1	3
3853	LOC_Os03g50390.1	3
3853	LOC_Os03g53410.1	3
3853	LOC_Os03g54780.1	3
3853	LOC_Os03g55560.1	3
3853	LOC_Os03g60150.1	3
3853	LOC_Os03g63020.1	3
3853	LOC_Os04g01874.1	4
3853	LOC_Os04g03530.1	4
3853	LOC_Os04g04800.1	4
3853	LOC_Os04g13640.1	4
3853	LOC_Os04g35700.1	4
3853	LOC_Os04g47240.1	4
3853	LOC_Os04g49460.1	4
3853	LOC_Os04g52140.1	4
3853	LOC_Os04g56120.1	4
3853	LOC_Os04g56130.1	4
3853	LOC_Os04g56060.1	4
3853	LOC_Os04g56530.1	4
3853	LOC_Os04g56110.1	4
3853	LOC_Os05g25540.1	5
3853	LOC_Os05g44290.1	5
3853	LOC_Os05g50190.1	5
3853	LOC_Os06g02550.1	6
3853	LOC_Os06g05070.1	6
3853	LOC_Os06g10160.1	6
3853	LOC_Os06g12590.1	6
3853	LOC_Os06g13320.1	6
3853	LOC_Os06g18000.1	6
3853	LOC_Os06g29340.1	6
3853	LOC_Os06g43030.1	6
3853	LOC_Os06g45350.1	6
3853	LOC_Os06g48980.1	6
3853	LOC_Os07g02780.1	7
3853	LOC_Os07g04810.1	7
3853	LOC_Os07g04820.1	7
3853	LOC_Os07g31290.1	7
3853	LOC_Os07g32400.1	7
3853	LOC_Os07g42200.1	7
3853	LOC_Os07g48730.1	7
3853	LOC_Os08g18920.1	8

3853	LOC_Os08g24780.1	8
3853	LOC_Os08g32600.1	8
3853	LOC_Os08g42580.1	8
3853	LOC_Os09g27010.1	9
3853	LOC_Os09g27150.1	9
3853	LOC_Os10g29620.1	10
3853	LOC_Os10g29540.1	10
3853	LOC_Os10g37190.1	10
3853	LOC_Os11g10100.1	11
3853	LOC_Os11g10690.1	11
3853	LOC_Os11g11890.1	11
3853	LOC_Os11g25510.1	11
3853	LOC_Os12g30570.1	12
3853	LOC_Os12g41260.1	12
3853	LOC_Os12g42660.1	12
3854	LOC_Os01g04330.1	1
3854	LOC_Os01g16240.1	1
3854	LOC_Os01g17190.1	1
3854	LOC_Os01g32120.1	1
3854	LOC_Os01g41990.1	1
3854	LOC_Os01g56030.1	1
3854	LOC_Os01g57470.1	1
3854	LOC_Os01g59530.1	1
3854	LOC_Os01g72100.1	1
3854	LOC_Os01g72540.1	1
3854	LOC_Os01g72550.1	1
3854	LOC_Os01g72530.1	1
3854	LOC_Os01g39134.1	1
3854	LOC_Os02g03020.1	2
3854	LOC_Os02g10470.1	2
3854	LOC_Os02g12880.1	2
3854	LOC_Os02g39380.1	2
3854	LOC_Os02g39950.1	2
3854	LOC_Os02g50060.1	2
3854	LOC_Os02g52540.1	2
3854	LOC_Os02g55880.1	2
3854	LOC_Os03g03830.1	3
3854	LOC_Os03g19720.1	3
3854	LOC_Os03g20370.1	3
3854	LOC_Os03g21380.1	3
3854	LOC_Os03g27790.1	3
3854	LOC_Os03g29770.1	3
3854	LOC_Os03g50760.1	3
3854	LOC_Os03g53200.1	3
3854	LOC_Os03g55960.1	3
3854	LOC_Os03g59600.1	3
3854	LOC_Os03g59870.1	3
3854	LOC_Os03g59770.1	3
3854	LOC_Os03g59790.1	3
3854	LOC_Os03g14590.1	3
3854	LOC_Os04g41540.1	4
3854	LOC_Os04g42430.1	4

3854	LOC_Os04g51240.1	4
3854	LOC_Os04g58480.1	4
3854	LOC_Os05g05460.1	5
3854	LOC_Os05g05710.1	5
3854	LOC_Os05g13580.1	5
3854	LOC_Os05g22270.1	5
3854	LOC_Os05g24780.1	5
3854	LOC_Os05g41200.1	5
3854	LOC_Os05g41210.1	5
3854	LOC_Os05g31620.1	5
3854	LOC_Os05g50180.1	5
3854	LOC_Os06g07560.1	6
3854	LOC_Os06g11030.1	6
3854	LOC_Os06g40720.1	6
3854	LOC_Os06g46950.1	6
3854	LOC_Os06g47640.1	6
3854	LOC_Os06g51250.1	6
3854	LOC_Os07g12240.1	7
3854	LOC_Os07g42730.1	7
3854	LOC_Os07g42660.1	7
3854	LOC_Os07g43800.1	7
3854	LOC_Os07g48340.1	7
3854	LOC_Os07g48780.1	7
3854	LOC_Os08g02420.1	8
3854	LOC_Os08g04890.1	8
3854	LOC_Os08g34340.1	8
3854	LOC_Os08g39290.1	8
3854	LOC_Os08g44660.1	8
3854	LOC_Os08g44390.1	8
3854	LOC_Os09g24580.1	9
3854	LOC_Os09g28490.1	9
3854	LOC_Os09g28500.1	9
3854	LOC_Os09g28510.1	9
3854	LOC_Os09g30506.1	9
3854	LOC_Os09g31040.1	9
3854	LOC_Os09g30490.1	9
3854	LOC_Os09g31000.1	9
3854	LOC_Os10g09850.1	10
3854	LOC_Os10g25010.1	10
3854	LOC_Os11g01390.1	11
3854	LOC_Os11g03980.1	11
3854	LOC_Os11g04480.1	11
3854	LOC_Os11g04770.1	11
3854	LOC_Os11g04820.1	11
3854	LOC_Os11g30180.1	11
3854	LOC_Os11g37550.1	11
3854	LOC_Os11g38780.1	11
3854	LOC_Os12g01400.1	12
3854	LOC_Os12g03816.1	12
3854	LOC_Os12g04240.1	12
3854	LOC_Os12g04560.1	12
3854	LOC_Os12g04580.1	12

3854	LOC_Os12g04680.1	12
3854	LOC_Os12g12730.1	12
3854	LOC_Os12g41110.1	12
3855	LOC_Os01g11570.1	1
3855	LOC_Os01g11750.1	1
3855	LOC_Os01g11760.1	1
3855	LOC_Os01g11790.1	1
3855	LOC_Os01g11650.1	1
3855	LOC_Os01g11660.1	1
3855	LOC_Os01g11700.1	1
3855	LOC_Os01g11710.1	1
3855	LOC_Os01g11730.1	1
3855	LOC_Os01g11740.1	1
3855	LOC_Os01g12320.1	1
3855	LOC_Os01g22640.1	1
3855	LOC_Os01g22780.1	1
3855	LOC_Os01g42730.1	1
3855	LOC_Os01g46080.1	1
3855	LOC_Os01g46090.1	1
3855	LOC_Os01g46210.1	1
3855	LOC_Os01g46220.1	1
3855	LOC_Os01g46120.1	1
3855	LOC_Os01g52770.1	1
3855	LOC_Os01g54470.1	1
3855	LOC_Os01g61200.1	1
3855	LOC_Os01g61570.1	1
3855	LOC_Os01g11620.1	1
3855	LOC_Os01g22660.1	1
3855	LOC_Os01g46169.1	1
3855	LOC_Os02g01140.1	2
3855	LOC_Os02g01980.1	2
3855	LOC_Os02g18870.1	2
3855	LOC_Os02g18954.1	2
3855	LOC_Os02g19040.1	2
3855	LOC_Os02g18990.1	2
3855	LOC_Os02g39170.1	2
3855	LOC_Os02g39590.1	2
3855	LOC_Os02g40440.1	2
3855	LOC_Os02g44850.1	2
3855	LOC_Os02g44860.1	2
3855	LOC_Os02g50000.1	2
3855	LOC_Os02g50690.1	2
3855	LOC_Os02g57110.1	2
3855	LOC_Os02g15230.1	2
3855	LOC_Os03g19670.1	3
3855	LOC_Os03g25000.1	3
3855	LOC_Os03g25010.1	3
3855	LOC_Os03g25030.1	3
3855	LOC_Os03g25040.1	3
3855	LOC_Os03g38390.1	3
3855	LOC_Os03g38470.1	3
3855	LOC_Os03g47940.1	3

3855	LOC_Os03g62740.1	3
3855	LOC_Os03g64170.1	3
3855	LOC_Os04g20180.1	4
3855	LOC_Os04g42860.1	4
3855	LOC_Os04g47390.1	4
3855	LOC_Os04g55660.1	4
3855	LOC_Os04g48800.1	4
3855	LOC_Os05g04240.1	5
3855	LOC_Os05g06710.1	5
3855	LOC_Os05g11910.1	5
3855	LOC_Os05g11950.1	5
3855	LOC_Os05g11970.1	5
3855	LOC_Os05g39220.1	5
3855	LOC_Os05g43090.1	5
3855	LOC_Os05g43100.1	5
3855	LOC_Os05g43110.1	5
3855	LOC_Os05g43120.1	5
3855	LOC_Os05g33270.1	5
3855	LOC_Os05g34700.1	5
3855	LOC_Os05g44200.1	5
3855	LOC_Os05g06720.1	5
3855	LOC_Os06g05550.1	6
3855	LOC_Os06g06230.1	6
3855	LOC_Os06g06250.1	6
3855	LOC_Os06g06260.1	6
3855	LOC_Os06g06290.1	6
3855	LOC_Os06g06520.1	6
3855	LOC_Os06g12410.1	6
3855	LOC_Os06g14630.1	6
3855	LOC_Os06g24404.1	6
3855	LOC_Os06g34120.1	6
3855	LOC_Os06g34070.1	6
3855	LOC_Os06g36520.1	6
3855	LOC_Os06g47910.1	6
3855	LOC_Os06g50940.1	6
3855	LOC_Os06g50950.1	6
3855	LOC_Os07g39740.1	7
3855	LOC_Os07g39750.1	7
3855	LOC_Os07g44780.1	7
3855	LOC_Os07g47210.1	7
3855	LOC_Os08g02094.1	8
3855	LOC_Os08g45150.1	8
3855	LOC_Os09g04710.1	9
3855	LOC_Os09g07290.1	9
3855	LOC_Os09g36880.1	9
3855	LOC_Os09g39430.1	9
3855	LOC_Os10g05088.1	10
3855	LOC_Os10g25380.1	10
3855	LOC_Os10g25400.1	10
3855	LOC_Os10g25340.1	10
3855	LOC_Os10g25420.1	10
3855	LOC_Os10g30290.1	10

3855	LOC_Os10g32580.1	10
3855	LOC_Os10g33690.1	10
3855	LOC_Os11g03520.1	11
3855	LOC_Os11g31940.1	11
3855	LOC_Os11g48070.1	11
3855	LOC_Os12g17570.1	12
3855	LOC_Os12g37910.1	12
3856	LOC_Os01g12020.1	1
3856	LOC_Os01g49640.1	1
3856	LOC_Os01g49650.1	1
3856	LOC_Os01g58650.1	1
3856	LOC_Os01g58660.1	1
3856	LOC_Os01g59870.1	1
3856	LOC_Os01g62980.1	1
3856	LOC_Os01g68580.1	1
3856	LOC_Os01g68589.1	1
3856	LOC_Os01g60740.1	1
3856	LOC_Os02g24720.1	2
3856	LOC_Os02g44320.1	2
3856	LOC_Os02g44310.1	2
3856	LOC_Os02g49280.1	2
3856	LOC_Os03g01310.1	3
3856	LOC_Os03g01300.1	3
3856	LOC_Os03g02050.1	3
3856	LOC_Os03g07100.1	3
3856	LOC_Os03g09230.1	3
3856	LOC_Os03g14630.1	3
3856	LOC_Os03g20760.1	3
3856	LOC_Os03g25350.1	3
3856	LOC_Os03g26800.1	3
3856	LOC_Os03g26820.1	3
3856	LOC_Os03g46180.1	3
3856	LOC_Os03g50960.1	3
3856	LOC_Os03g55740.1	3
3856	LOC_Os03g57970.1	3
3856	LOC_Os03g57980.1	3
3856	LOC_Os03g57990.1	3
3856	LOC_Os03g58670.1	3
3856	LOC_Os03g58940.1	3
3856	LOC_Os03g59380.1	3
3856	LOC_Os03g46150.1	3
3856	LOC_Os04g33920.1	4
3856	LOC_Os04g33930.1	4
3856	LOC_Os04g38840.1	4
3856	LOC_Os04g46810.1	4
3856	LOC_Os04g46830.1	4
3856	LOC_Os04g46820.1	4
3856	LOC_Os04g52260.1	4
3856	LOC_Os04g55170.1	4
3856	LOC_Os05g06780.1	5
3856	LOC_Os05g41970.1	5
3856	LOC_Os05g40010.1	5

3856	LOC_Os05g41030.1	5
3856	LOC_Os05g47730.1	5
3856	LOC_Os05g47700.1	5
3856	LOC_Os06g01580.1	6
3856	LOC_Os06g06340.1	6
3856	LOC_Os06g12440.1	6
3856	LOC_Os06g34840.1	6
3856	LOC_Os06g46780.1	6
3856	LOC_Os06g46870.1	6
3856	LOC_Os06g49190.1	6
3856	LOC_Os06g49770.1	6
3856	LOC_Os06g47200.1	6
3856	LOC_Os07g07790.1	7
3856	LOC_Os07g07870.1	7
3856	LOC_Os07g07920.1	7
3856	LOC_Os07g07860.1	7
3856	LOC_Os07g07930.1	7
3856	LOC_Os07g09970.1	7
3856	LOC_Os07g11310.1	7
3856	LOC_Os07g11360.1	7
3856	LOC_Os07g11630.1	7
3856	LOC_Os07g11320.1	7
3856	LOC_Os07g11330.1	7
3856	LOC_Os07g11380.1	7
3856	LOC_Os07g11410.1	7
3856	LOC_Os07g11510.1	7
3856	LOC_Os07g11650.1	7
3856	LOC_Os07g12080.1	7
3856	LOC_Os07g18990.1	7
3856	LOC_Os07g18750.1	7
3856	LOC_Os07g27940.1	7
3856	LOC_Os07g29230.1	7
3856	LOC_Os07g30590.1	7
3856	LOC_Os07g43290.1	7
3856	LOC_Os08g03690.1	8
3856	LOC_Os08g42040.1	8
3856	LOC_Os08g43290.1	8
3856	LOC_Os09g35700.1	9
3856	LOC_Os10g05720.1	10
3856	LOC_Os10g09920.1	10
3856	LOC_Os10g11750.1	10
3856	LOC_Os10g20830.1	10
3856	LOC_Os10g20880.1	10
3856	LOC_Os10g20890.1	10
3856	LOC_Os10g20840.1	10
3856	LOC_Os10g36070.1	10
3856	LOC_Os10g36090.1	10
3856	LOC_Os10g36100.1	10
3856	LOC_Os10g36110.1	10
3856	LOC_Os10g36160.1	10
3856	LOC_Os10g36170.1	10
3856	LOC_Os10g40420.1	10

3856	LOC_Os10g40430.1	10
3856	LOC_Os10g40460.1	10
3856	LOC_Os10g40470.1	10
3856	LOC_Os10g40480.1	10
3856	LOC_Os10g40510.1	10
3856	LOC_Os10g40440.1	10
3856	LOC_Os10g40520.1	10
3856	LOC_Os10g40530.1	10
3856	LOC_Os10g40614.1	10
3856	LOC_Os11g02350.1	11
3856	LOC_Os11g02379.1	11
3856	LOC_Os11g02400.1	11
3856	LOC_Os11g24070.1	11
3856	LOC_Os11g29420.1	11
3856	LOC_Os11g33000.1	11
3856	LOC_Os11g34660.1	11
3856	LOC_Os11g37280.1	11
3856	LOC_Os11g40530.1	11
3856	LOC_Os12g02290.1	12
3856	LOC_Os12g02300.1	12
3856	LOC_Os12g02320.1	12
3856	LOC_Os12g02310.1	12
3856	LOC_Os12g02330.1	12
3856	LOC_Os12g02340.1	12
3856	LOC_Os12g29040.1	12
3856	LOC_Os12g28880.1	12
3857	LOC_Os01g05880.1	1
3857	LOC_Os01g05890.1	1
3857	LOC_Os01g17390.1	1
3857	LOC_Os01g28300.1	1
3857	LOC_Os01g28150.1	1
3857	LOC_Os01g28520.1	1
3857	LOC_Os01g33490.1	1
3857	LOC_Os01g34220.1	1
3857	LOC_Os01g36940.1	1
3857	LOC_Os01g39690.1	1
3857	LOC_Os01g45650.1	1
3857	LOC_Os01g45900.1	1
3857	LOC_Os01g54850.1	1
3857	LOC_Os01g55210.1	1
3857	LOC_Os01g59180.1	1
3857	LOC_Os01g60920.1	1
3857	LOC_Os01g65510.1	1
3857	LOC_Os01g69940.1	1
3857	LOC_Os01g71450.1	1
3857	LOC_Os01g59910.1	1
3857	LOC_Os02g03810.1	2
3857	LOC_Os02g28600.1	2
3857	LOC_Os02g41930.1	2
3857	LOC_Os02g46690.1	2
3857	LOC_Os02g52130.1	2
3857	LOC_Os02g54240.1	2

3857	LOC_Os02g56770.1	2
3857	LOC_Os03g10040.1	3
3857	LOC_Os03g25080.1	3
3857	LOC_Os03g26840.1	3
3857	LOC_Os03g28130.1	3
3857	LOC_Os03g30920.1	3
3857	LOC_Os03g36439.1	3
3857	LOC_Os03g43060.1	3
3857	LOC_Os03g43770.1	3
3857	LOC_Os03g44980.1	3
3857	LOC_Os03g46120.1	3
3857	LOC_Os03g46690.1	3
3857	LOC_Os03g46500.1	3
3857	LOC_Os03g46530.1	3
3857	LOC_Os03g51760.1	3
3857	LOC_Os03g56440.1	3
3857	LOC_Os03g56450.1	3
3857	LOC_Os03g64360.1	3
3857	LOC_Os04g08510.1	4
3857	LOC_Os04g24820.1	4
3857	LOC_Os04g25840.1	4
3857	LOC_Os04g31540.1	4
3857	LOC_Os04g35190.1	4
3857	LOC_Os04g36020.1	4
3857	LOC_Os04g39070.1	4
3857	LOC_Os04g39080.1	4
3857	LOC_Os04g40030.1	4
3857	LOC_Os04g40910.1	4
3857	LOC_Os04g49360.1	4
3857	LOC_Os04g50200.1	4
3857	LOC_Os04g57290.1	4
3857	LOC_Os05g03210.1	5
3857	LOC_Os05g07950.1	5
3857	LOC_Os05g30920.1	5
3857	LOC_Os05g45040.1	5
3857	LOC_Os05g49400.1	5
3857	LOC_Os05g49450.1	5
3857	LOC_Os05g49530.1	5
3857	LOC_Os05g51100.1	5
3857	LOC_Os06g06050.1	6
3857	LOC_Os06g07000.1	6
3857	LOC_Os06g07430.1	6
3857	LOC_Os06g10290.1	6
3857	LOC_Os06g11630.1	6
3857	LOC_Os06g13850.1	6
3857	LOC_Os06g19670.1	6
3857	LOC_Os06g41520.1	6
3857	LOC_Os06g45460.1	6
3857	LOC_Os06g47100.1	6
3857	LOC_Os06g49530.1	6
3857	LOC_Os07g04750.1	7
3857	LOC_Os07g07520.1	7

3857	LOC_Os07g09110.1	7
3857	LOC_Os07g13930.1	7
3857	LOC_Os07g27030.1	7
3857	LOC_Os07g36280.1	7
3857	LOC_Os07g36290.1	7
3857	LOC_Os07g36320.1	7
3857	LOC_Os07g36530.1	7
3857	LOC_Os07g37080.1	7
3857	LOC_Os07g37400.1	7
3857	LOC_Os07g42590.1	7
3857	LOC_Os07g47160.1	7
3857	LOC_Os07g47170.1	7
3857	LOC_Os08g01430.1	8
3857	LOC_Os08g09420.1	8
3857	LOC_Os08g09650.1	8
3857	LOC_Os08g10340.1	8
3857	LOC_Os08g16840.1	8
3857	LOC_Os08g20440.1	8
3857	LOC_Os08g27190.1	8
3857	LOC_Os08g29400.1	8
3857	LOC_Os08g31270.1	8
3857	LOC_Os08g33140.1	8
3857	LOC_Os08g36960.1	8
3857	LOC_Os08g38330.1	8
3857	LOC_Os08g38360.1	8
3857	LOC_Os08g41750.1	8
3857	LOC_Os08g41760.1	8
3857	LOC_Os08g43220.1	8
3857	LOC_Os08g43530.1	8
3857	LOC_Os09g06620.1	9
3857	LOC_Os09g12150.1	9
3857	LOC_Os09g20650.1	9
3857	LOC_Os09g28120.1	9
3857	LOC_Os09g32240.1	9
3857	LOC_Os10g05200.1	10
3857	LOC_Os10g04750.1	10
3857	LOC_Os10g30280.1	10
3857	LOC_Os10g35920.1	10
3857	LOC_Os10g41829.1	10
3857	LOC_Os11g09360.1	11
3857	LOC_Os11g16330.1	11
3857	LOC_Os11g41560.1	11
3857	LOC_Os11g41860.1	11
3857	LOC_Os12g30940.1	12
3857	LOC_Os12g32630.1	12
3857	LOC_Os12g41630.1	12
3858	LOC_Os01g11130.1	1
3858	LOC_Os01g26840.1	1
3858	LOC_Os01g42820.1	1
3858	LOC_Os01g56110.1	1
3858	LOC_Os01g56120.1	1
3858	LOC_Os01g65530.1	1

3858	LOC_Os01g65550.1	1
3858	LOC_Os01g68790.1	1
3858	LOC_Os01g71200.1	1
3858	LOC_Os01g71770.1	1
3858	LOC_Os01g72650.1	1
3858	LOC_Os01g72834.1	1
3858	LOC_Os01g74340.1	1
3858	LOC_Os02g04560.1	2
3858	LOC_Os02g11750.1	2
3858	LOC_Os02g33320.1	2
3858	LOC_Os02g35950.1	2
3858	LOC_Os02g39060.1	2
3858	LOC_Os02g52140.1	2
3858	LOC_Os02g54690.1	2
3858	LOC_Os02g57010.1	2
3858	LOC_Os02g54700.1	2
3858	LOC_Os02g48340.1	2
3858	LOC_Os02g51890.1	2
3858	LOC_Os03g03140.1	3
3858	LOC_Os03g04780.1	3
3858	LOC_Os03g04520.1	3
3858	LOC_Os03g07800.1	3
3858	LOC_Os03g14190.1	3
3858	LOC_Os03g17010.1	3
3858	LOC_Os03g17060.1	3
3858	LOC_Os03g17760.1	3
3858	LOC_Os03g18720.1	3
3858	LOC_Os03g21020.1	3
3858	LOC_Os03g24890.1	3
3858	LOC_Os03g25770.1	3
3858	LOC_Os03g25960.1	3
3858	LOC_Os03g40310.1	3
3858	LOC_Os03g56020.1	3
3858	LOC_Os03g58720.1	3
3858	LOC_Os03g59550.1	3
3858	LOC_Os03g59710.1	3
3858	LOC_Os03g60930.1	3
3858	LOC_Os03g63730.1	3
3858	LOC_Os03g46770.1	3
3858	LOC_Os03g37270.1	3
3858	LOC_Os04g02870.1	4
3858	LOC_Os04g33810.1	4
3858	LOC_Os04g37690.1	4
3858	LOC_Os04g39629.1	4
3858	LOC_Os04g41910.1	4
3858	LOC_Os04g49440.1	4
3858	LOC_Os04g50110.1	4
3858	LOC_Os04g52200.1	4
3858	LOC_Os04g58590.1	4
3858	LOC_Os04g53440.1	4
3858	LOC_Os04g54870.1	4
3858	LOC_Os04g24170.1	4

3858	LOC_Os05g04850.1	5
3858	LOC_Os05g13620.1	5
3858	LOC_Os05g13630.1	5
3858	LOC_Os05g13650.1	5
3858	LOC_Os05g23800.1	5
3858	LOC_Os05g30980.1	5
3858	LOC_Os06g03690.1	6
3858	LOC_Os06g05800.1	6
3858	LOC_Os06g11620.1	6
3858	LOC_Os06g11730.1	6
3858	LOC_Os06g13839.1	6
3858	LOC_Os06g13999.1	6
3858	LOC_Os06g14470.1	6
3858	LOC_Os06g37000.1	6
3858	LOC_Os06g41790.1	6
3858	LOC_Os06g47300.1	6
3858	LOC_Os06g35030.1	6
3858	LOC_Os07g03240.1	7
3858	LOC_Os07g04570.1	7
3858	LOC_Os07g06450.1	7
3858	LOC_Os07g13300.1	7
3858	LOC_Os07g33330.1	7
3858	LOC_Os07g36490.1	7
3858	LOC_Os07g41120.1	7
3858	LOC_Os07g42380.1	7
3858	LOC_Os07g43810.1	7
3858	LOC_Os07g43950.1	7
3858	LOC_Os07g46760.1	7
3858	LOC_Os08g02130.1	8
3858	LOC_Os08g02330.1	8
3858	LOC_Os08g02390.1	8
3858	LOC_Os08g04440.1	8
3858	LOC_Os08g09100.1	8
3858	LOC_Os08g09350.1	8
3858	LOC_Os08g15080.1	8
3858	LOC_Os08g31810.1	8
3858	LOC_Os08g33120.1	8
3858	LOC_Os08g33350.1	8
3858	LOC_Os08g37700.1	8
3858	LOC_Os08g38380.1	8
3858	LOC_Os08g39440.1	8
3858	LOC_Os08g40880.1	8
3858	LOC_Os08g43360.1	8
3858	LOC_Os08g44290.1	8
3858	LOC_Os08g45240.1	8
3858	LOC_Os08g38410.1	8
3858	LOC_Os09g10760.1	9
3858	LOC_Os09g20780.1	9
3858	LOC_Os09g20710.1	9
3858	LOC_Os09g28810.1	9
3858	LOC_Os09g33870.1	9
3858	LOC_Os09g35840.1	9

3858	LOC_Os09g35850.1	9
3858	LOC_Os09g39180.1	9
3858	LOC_Os10g17454.1	10
3858	LOC_Os10g30370.1	10
3858	LOC_Os10g41960.1	10
3858	LOC_Os11g01020.1	11
3858	LOC_Os11g03890.1	11
3858	LOC_Os11g07490.1	11
3858	LOC_Os11g39030.1	11
3858	LOC_Os11g40510.1	11
3858	LOC_Os12g01010.1	12
3858	LOC_Os12g01190.1	12
3858	LOC_Os12g03710.1	12
3858	LOC_Os12g38800.1	12
3858	LOC_Os12g41310.1	12
3858	LOC_Os12g41370.1	12
3859	LOC_Os01g04020.1	1
3859	LOC_Os01g10370.1	1
3859	LOC_Os01g12440.1	1
3859	LOC_Os01g21120.1	1
3859	LOC_Os01g46870.1	1
3859	LOC_Os01g58420.1	1
3859	LOC_Os01g59780.1	1
3859	LOC_Os01g64790.1	1
3859	LOC_Os01g66270.1	1
3859	LOC_Os01g07120.1	1
3859	LOC_Os02g06330.1	2
3859	LOC_Os02g09650.1	2
3859	LOC_Os02g10760.1	2
3859	LOC_Os02g29550.1	2
3859	LOC_Os02g32040.1	2
3859	LOC_Os02g32140.1	2
3859	LOC_Os02g34260.1	2
3859	LOC_Os02g34270.1	2
3859	LOC_Os02g35240.1	2
3859	LOC_Os02g38090.1	2
3859	LOC_Os02g43790.1	2
3859	LOC_Os02g43940.1	2
3859	LOC_Os02g43970.1	2
3859	LOC_Os02g45450.1	2
3859	LOC_Os02g51300.1	2
3859	LOC_Os02g51670.1	2
3859	LOC_Os02g52670.1	2
3859	LOC_Os02g54160.1	2
3859	LOC_Os02g55380.1	2
3859	LOC_Os03g07830.1	3
3859	LOC_Os03g07940.1	3
3859	LOC_Os03g08470.1	3
3859	LOC_Os03g08490.1	3
3859	LOC_Os03g05590.1	3
3859	LOC_Os03g08460.1	3
3859	LOC_Os03g08500.1	3

3859	LOC_Os03g09170.1	3
3859	LOC_Os03g12950.1	3
3859	LOC_Os03g15660.1	3
3859	LOC_Os03g19900.1	3
3859	LOC_Os03g22170.1	3
3859	LOC_Os03g60120.1	3
3859	LOC_Os03g60430.1	3
3859	LOC_Os04g18650.1	4
3859	LOC_Os04g32790.1	4
3859	LOC_Os04g34970.1	4
3859	LOC_Os04g44670.1	4
3859	LOC_Os04g46220.1	4
3859	LOC_Os04g46240.1	4
3859	LOC_Os04g46250.1	4
3859	LOC_Os04g46400.1	4
3859	LOC_Os04g46410.1	4
3859	LOC_Os04g46440.1	4
3859	LOC_Os04g48350.1	4
3859	LOC_Os04g52090.1	4
3859	LOC_Os04g55520.1	4
3859	LOC_Os04g55970.1	4
3859	LOC_Os04g56150.1	4
3859	LOC_Os04g57340.1	4
3859	LOC_Os04g55560.1	4
3859	LOC_Os05g03040.1	5
3859	LOC_Os05g25260.1	5
3859	LOC_Os05g29810.1	5
3859	LOC_Os05g36100.1	5
3859	LOC_Os05g28350.1	5
3859	LOC_Os05g41760.1	5
3859	LOC_Os05g39590.1	5
3859	LOC_Os05g41780.1	5
3859	LOC_Os05g49010.1	5
3859	LOC_Os05g49700.1	5
3859	LOC_Os05g27930.1	5
3859	LOC_Os06g03670.1	6
3859	LOC_Os06g05340.1	6
3859	LOC_Os06g06540.1	6
3859	LOC_Os06g06970.1	6
3859	LOC_Os06g07030.1	6
3859	LOC_Os06g08340.1	6
3859	LOC_Os06g09390.1	6
3859	LOC_Os06g09760.1	6
3859	LOC_Os06g10780.1	6
3859	LOC_Os06g11860.1	6
3859	LOC_Os06g23050.1	6
3859	LOC_Os06g36000.1	6
3859	LOC_Os06g40150.1	6
3859	LOC_Os06g42990.1	6
3859	LOC_Os06g43220.1	6
3859	LOC_Os06g44750.1	6
3859	LOC_Os06g47590.1	6

3859	LOC_Os07g10410.1	7
3859	LOC_Os07g13170.1	7
3859	LOC_Os07g38750.1	7
3859	LOC_Os07g42510.1	7
3859	LOC_Os07g47330.1	7
3859	LOC_Os07g47790.1	7
3859	LOC_Os08g07440.1	8
3859	LOC_Os08g07700.1	8
3859	LOC_Os08g27220.1	8
3859	LOC_Os08g31580.1	8
3859	LOC_Os08g34360.1	8
3859	LOC_Os08g35240.1	8
3859	LOC_Os08g36920.1	8
3859	LOC_Os08g41030.1	8
3859	LOC_Os08g42550.1	8
3859	LOC_Os08g43200.1	8
3859	LOC_Os08g43210.1	8
3859	LOC_Os08g44960.1	8
3859	LOC_Os08g45110.1	8
3859	LOC_Os09g11460.1	9
3859	LOC_Os09g11480.1	9
3859	LOC_Os09g13940.1	9
3859	LOC_Os09g20350.1	9
3859	LOC_Os09g25600.1	9
3859	LOC_Os09g26420.1	9
3859	LOC_Os09g28440.1	9
3859	LOC_Os09g35030.1	9
3859	LOC_Os09g35010.1	9
3859	LOC_Os09g35020.1	9
3859	LOC_Os09g39810.1	9
3859	LOC_Os10g22600.1	10
3859	LOC_Os10g25170.1	10
3859	LOC_Os10g26590.1	10
3859	LOC_Os10g30840.1	10
3859	LOC_Os10g38000.1	10
3859	LOC_Os10g41130.1	10
3859	LOC_Os10g41330.1	10
3859	LOC_Os11g03540.1	11
3859	LOC_Os11g13840.1	11
3859	LOC_Os12g03290.1	12
3859	LOC_Os12g39330.1	12
3859	LOC_Os12g40960.1	12
3859	LOC_Os12g41060.1	12
3859	LOC_Os12g41030.1	12
3860	LOC_Os01g03510.1	1
3860	LOC_Os01g04870.1	1
3860	LOC_Os01g08770.1	1
3860	LOC_Os01g09020.1	1
3860	LOC_Os01g10790.1	1
3860	LOC_Os01g13140.1	1
3860	LOC_Os01g13730.1	1
3860	LOC_Os01g21940.1	1

3860	LOC_Os01g37120.1	1
3860	LOC_Os01g41630.1	1
3860	LOC_Os01g43250.1	1
3860	LOC_Os01g44394.1	1
3860	LOC_Os01g46510.1	1
3860	LOC_Os01g50690.1	1
3860	LOC_Os01g56860.1	1
3860	LOC_Os01g63900.1	1
3860	LOC_Os01g69970.1	1
3860	LOC_Os01g71780.1	1
3860	LOC_Os01g72220.1	1
3860	LOC_Os01g51300.1	1
3860	LOC_Os01g28680.1	1
3860	LOC_Os02g03990.1	2
3860	LOC_Os02g04320.1	2
3860	LOC_Os02g04440.1	2
3860	LOC_Os02g11060.1	2
3860	LOC_Os02g13110.1	2
3860	LOC_Os02g14790.1	2
3860	LOC_Os02g18820.1	2
3860	LOC_Os02g20430.1	2
3860	LOC_Os02g21490.1	2
3860	LOC_Os02g32430.1	2
3860	LOC_Os02g33860.1	2
3860	LOC_Os02g45810.1	2
3860	LOC_Os02g50740.1	2
3860	LOC_Os02g56880.1	2
3860	LOC_Os02g57220.1	2
3860	LOC_Os02g37856.1	2
3860	LOC_Os03g02110.1	3
3860	LOC_Os03g08830.1	3
3860	LOC_Os03g05720.1	3
3860	LOC_Os03g10990.1	3
3860	LOC_Os03g17780.1	3
3860	LOC_Os03g19340.1	3
3860	LOC_Os03g21990.1	3
3860	LOC_Os03g23909.1	3
3860	LOC_Os03g23929.1	3
3860	LOC_Os03g27970.1	3
3860	LOC_Os03g33580.1	3
3860	LOC_Os03g43890.1	3
3860	LOC_Os03g46650.1	3
3860	LOC_Os03g47780.1	3
3860	LOC_Os03g48090.1	3
3860	LOC_Os03g49200.1	3
3860	LOC_Os03g53530.1	3
3860	LOC_Os03g53510.1	3
3860	LOC_Os03g58570.1	3
3860	LOC_Os03g61630.1	3
3860	LOC_Os03g63470.1	3
3860	LOC_Os03g64110.1	3
3860	LOC_Os03g64300.1	3

3860	LOC_Os03g54770.1	3
3860	LOC_Os04g11880.1	4
3860	LOC_Os04g40560.1	4
3860	LOC_Os04g42880.1	4
3860	LOC_Os04g43130.1	4
3860	LOC_Os04g47700.1	4
3860	LOC_Os04g50660.1	4
3860	LOC_Os05g05210.1	5
3860	LOC_Os05g16660.1	5
3860	LOC_Os05g23430.1	5
3860	LOC_Os05g30010.1	5
3860	LOC_Os05g33710.1	5
3860	LOC_Os05g44320.1	5
3860	LOC_Os05g46570.1	5
3860	LOC_Os05g47890.1	5
3860	LOC_Os06g04040.1	6
3860	LOC_Os06g13140.1	6
3860	LOC_Os06g30680.1	6
3860	LOC_Os06g36770.1	6
3860	LOC_Os06g39760.1	6
3860	LOC_Os06g44370.1	6
3860	LOC_Os06g50880.1	6
3860	LOC_Os07g01730.1	7
3860	LOC_Os07g03160.1	7
3860	LOC_Os07g09000.1	7
3860	LOC_Os07g12320.1	7
3860	LOC_Os07g14280.1	7
3860	LOC_Os07g14830.1	7
3860	LOC_Os07g22220.1	7
3860	LOC_Os07g22534.1	7
3860	LOC_Os07g25440.1	7
3860	LOC_Os07g38430.1	7
3860	LOC_Os07g40030.1	7
3860	LOC_Os07g39950.1	7
3860	LOC_Os07g41190.1	7
3860	LOC_Os07g46370.1	7
3860	LOC_Os07g46620.1	7
3860	LOC_Os07g49300.1	7
3860	LOC_Os07g49090.1	7
3860	LOC_Os08g01680.1	8
3860	LOC_Os08g04270.1	8
3860	LOC_Os08g04290.1	8
3860	LOC_Os08g07960.1	8
3860	LOC_Os08g18150.1	8
3860	LOC_Os08g18880.1	8
3860	LOC_Os08g21660.1	8
3860	LOC_Os08g38570.1	8
3860	LOC_Os08g44010.1	8
3860	LOC_Os09g06560.1	9
3860	LOC_Os09g09470.1	9
3860	LOC_Os09g12550.1	9
3860	LOC_Os09g12710.1	9

3860	LOC_Os09g19900.1	9
3860	LOC_Os09g24260.1	9
3860	LOC_Os09g30090.1	9
3860	LOC_Os09g36900.1	9
3860	LOC_Os09g39420.1	9
3860	LOC_Os10g32710.1	10
3860	LOC_Os10g32880.1	10
3860	LOC_Os10g39760.1	10
3860	LOC_Os11g03794.1	11
3860	LOC_Os11g03990.1	11
3860	LOC_Os11g07480.1	11
3860	LOC_Os11g10680.1	11
3860	LOC_Os11g39650.1	11
3860	LOC_Os11g43890.1	11
3860	LOC_Os12g03540.1	12
3860	LOC_Os12g06810.1	12
3860	LOC_Os12g07450.1	12
3860	LOC_Os12g41620.1	12
3860	LOC_Os12g42150.1	12
3860	LOC_Os12g19590.1	12
3860	LOC_Os12g03822.1	12
3861	LOC_Os01g31430.1	1
3861	LOC_Os01g36730.1	1
3861	LOC_Os01g37360.1	1
3861	LOC_Os01g48480.1	1
3861	LOC_Os01g54820.1	1
3861	LOC_Os01g57810.1	1
3861	LOC_Os01g58720.1	1
3861	LOC_Os01g62340.1	1
3861	LOC_Os01g64050.1	1
3861	LOC_Os02g11650.1	2
3861	LOC_Os02g19310.1	2
3861	LOC_Os02g20120.1	2
3861	LOC_Os02g20650.1	2
3861	LOC_Os02g25110.1	2
3861	LOC_Os02g26050.1	2
3861	LOC_Os02g29680.1	2
3861	LOC_Os02g29740.1	2
3861	LOC_Os02g29770.1	2
3861	LOC_Os02g36650.1	2
3861	LOC_Os02g47720.1	2
3861	LOC_Os02g52980.1	2
3861	LOC_Os03g26690.1	3
3861	LOC_Os03g26880.1	3
3861	LOC_Os03g35730.1	3
3861	LOC_Os03g35860.1	3
3861	LOC_Os03g37180.1	3
3861	LOC_Os03g39356.1	3
3861	LOC_Os03g43070.1	3
3861	LOC_Os03g43210.1	3
3861	LOC_Os03g45040.1	3
3861	LOC_Os03g52100.1	3

3861	LOC_Os04g01380.1	4
3861	LOC_Os04g01410.1	4
3861	LOC_Os04g03260.1	4
3861	LOC_Os04g09460.1	4
3861	LOC_Os04g11280.1	4
3861	LOC_Os04g11680.1	4
3861	LOC_Os04g13750.1	4
3861	LOC_Os04g16560.1	4
3861	LOC_Os04g17370.1	4
3861	LOC_Os04g19080.1	4
3861	LOC_Os04g21760.1	4
3861	LOC_Os04g22190.1	4
3861	LOC_Os04g22790.1	4
3861	LOC_Os04g26710.1	4
3861	LOC_Os05g14100.1	5
3861	LOC_Os05g26160.1	5
3861	LOC_Os05g16260.1	5
3861	LOC_Os05g20270.1	5
3861	LOC_Os05g20280.1	5
3861	LOC_Os05g22760.1	5
3861	LOC_Os05g24330.1	5
3861	LOC_Os05g39270.1	5
3861	LOC_Os06g07710.1	6
3861	LOC_Os06g13920.1	6
3861	LOC_Os06g17800.1	6
3861	LOC_Os06g18940.1	6
3861	LOC_Os06g20580.1	6
3861	LOC_Os06g20650.1	6
3861	LOC_Os06g21730.1	6
3861	LOC_Os06g22220.1	6
3861	LOC_Os06g22590.1	6
3861	LOC_Os06g25030.1	6
3861	LOC_Os06g25770.1	6
3861	LOC_Os06g27490.1	6
3861	LOC_Os06g30080.1	6
3861	LOC_Os06g34590.1	6
3861	LOC_Os06g38270.1	6
3861	LOC_Os06g48140.1	6
3861	LOC_Os07g06120.1	7
3861	LOC_Os07g13130.1	7
3861	LOC_Os07g11270.1	7
3861	LOC_Os07g12070.1	7
3861	LOC_Os07g12970.1	7
3861	LOC_Os07g13760.1	7
3861	LOC_Os07g15760.1	7
3861	LOC_Os07g18370.1	7
3861	LOC_Os07g20860.1	7
3861	LOC_Os07g22170.1	7
3861	LOC_Os07g25640.1	7
3861	LOC_Os07g26350.1	7
3861	LOC_Os07g26840.1	7
3861	LOC_Os07g27210.1	7

3861	LOC_Os07g27730.1	7
3861	LOC_Os07g28560.1	7
3861	LOC_Os07g30660.1	7
3861	LOC_Os07g45630.1	7
3861	LOC_Os07g47870.1	7
3861	LOC_Os08g08450.1	8
3861	LOC_Os08g12140.1	8
3861	LOC_Os08g12640.1	8
3861	LOC_Os08g15990.1	8
3861	LOC_Os08g17940.1	8
3861	LOC_Os08g21490.1	8
3861	LOC_Os08g21970.1	8
3861	LOC_Os08g22260.1	8
3861	LOC_Os08g22750.1	8
3861	LOC_Os08g22780.1	8
3861	LOC_Os08g28640.1	8
3861	LOC_Os09g02920.1	9
3861	LOC_Os09g07080.1	9
3861	LOC_Os09g07880.1	9
3861	LOC_Os09g18090.1	9
3861	LOC_Os09g22220.1	9
3861	LOC_Os10g02560.1	10
3861	LOC_Os10g06350.1	10
3861	LOC_Os10g06400.1	10
3861	LOC_Os10g08080.1	10
3861	LOC_Os10g10110.1	10
3861	LOC_Os10g11180.1	10
3861	LOC_Os10g13470.1	10
3861	LOC_Os10g17300.1	10
3861	LOC_Os10g17870.1	10
3861	LOC_Os10g18550.1	10
3861	LOC_Os10g24200.1	10
3861	LOC_Os10g24220.1	10
3861	LOC_Os11g08620.1	11
3861	LOC_Os11g18450.1	11
3861	LOC_Os11g18820.1	11
3861	LOC_Os11g19650.1	11
3861	LOC_Os11g20110.1	11
3861	LOC_Os11g22870.1	11
3861	LOC_Os11g22330.1	11
3861	LOC_Os11g22510.1	11
3861	LOC_Os11g22930.1	11
3861	LOC_Os11g25660.1	11
3861	LOC_Os11g35910.1	11
3861	LOC_Os11g37180.1	11
3861	LOC_Os11g41430.1	11
3861	LOC_Os11g41490.1	11
3861	LOC_Os11g42640.1	11
3861	LOC_Os12g08500.1	12
3861	LOC_Os12g09490.1	12
3861	LOC_Os12g10070.1	12
3861	LOC_Os12g14050.1	12

3861	LOC_Os12g15160.1	12
3861	LOC_Os12g16140.1	12
3861	LOC_Os12g19670.1	12
3861	LOC_Os12g22330.1	12
3861	LOC_Os12g22080.1	12
3861	LOC_Os12g22400.1	12
3861	LOC_Os12g24820.1	12
3861	LOC_Os12g25570.1	12
3861	LOC_Os12g34730.1	12
3861	LOC_Os12g42460.1	12
3862	LOC_Os01g07770.1	1
3862	LOC_Os01g10850.1	1
3862	LOC_Os01g15810.1	1
3862	LOC_Os01g15830.1	1
3862	LOC_Os01g16450.1	1
3862	LOC_Os01g18910.1	1
3862	LOC_Os01g18930.1	1
3862	LOC_Os01g18950.1	1
3862	LOC_Os01g18970.1	1
3862	LOC_Os01g18890.1	1
3862	LOC_Os01g19020.1	1
3862	LOC_Os01g22249.1	1
3862	LOC_Os01g22336.1	1
3862	LOC_Os01g22230.1	1
3862	LOC_Os01g22352.1	1
3862	LOC_Os01g22370.1	1
3862	LOC_Os01g28030.1	1
3862	LOC_Os01g36240.1	1
3862	LOC_Os01g57730.1	1
3862	LOC_Os01g73220.1	1
3862	LOC_Os01g73170.1	1
3862	LOC_Os01g73190.1	1
3862	LOC_Os01g73200.1	1
3862	LOC_Os02g06630.1	2
3862	LOC_Os02g14180.1	2
3862	LOC_Os02g14160.1	2
3862	LOC_Os02g14170.1	2
3862	LOC_Os02g14430.1	2
3862	LOC_Os02g14440.1	2
3862	LOC_Os02g14460.1	2
3862	LOC_Os02g34810.1	2
3862	LOC_Os02g50770.1	2
3862	LOC_Os02g58720.1	2
3862	LOC_Os03g02920.1	3
3862	LOC_Os03g05770.1	3
3862	LOC_Os03g13180.1	3
3862	LOC_Os03g13200.1	3
3862	LOC_Os03g13210.1	3
3862	LOC_Os03g17690.1	3
3862	LOC_Os03g22010.1	3
3862	LOC_Os03g22020.1	3
3862	LOC_Os03g25280.1	3

3862	LOC_Os03g25300.1	3
3862	LOC_Os03g25340.1	3
3862	LOC_Os03g25320.1	3
3862	LOC_Os03g25330.1	3
3862	LOC_Os03g25360.1	3
3862	LOC_Os03g25370.1	3
3862	LOC_Os03g32050.1	3
3862	LOC_Os03g32060.1	3
3862	LOC_Os03g36560.1	3
3862	LOC_Os03g55410.1	3
3862	LOC_Os03g55420.1	3
3862	LOC_Os04g01550.1	4
3862	LOC_Os04g04750.1	4
3862	LOC_Os04g14680.1	4
3862	LOC_Os04g34630.1	4
3862	LOC_Os04g35520.1	4
3862	LOC_Os04g39100.1	4
3862	LOC_Os04g51300.1	4
3862	LOC_Os04g53640.1	4
3862	LOC_Os04g55740.1	4
3862	LOC_Os04g56180.1	4
3862	LOC_Os04g59160.1	4
3862	LOC_Os04g59190.1	4
3862	LOC_Os04g59200.1	4
3862	LOC_Os04g59210.1	4
3862	LOC_Os04g59150.1	4
3862	LOC_Os04g59260.1	4
3862	LOC_Os05g04380.1	5
3862	LOC_Os05g04410.1	5
3862	LOC_Os05g04440.1	5
3862	LOC_Os05g04450.1	5
3862	LOC_Os05g04470.1	5
3862	LOC_Os05g04490.1	5
3862	LOC_Os05g04500.1	5
3862	LOC_Os05g06970.1	5
3862	LOC_Os05g14260.1	5
3862	LOC_Os05g41990.1	5
3862	LOC_Os05g42000.1	5
3862	LOC_Os06g16350.1	6
3862	LOC_Os06g20150.1	6
3862	LOC_Os06g27850.1	6
3862	LOC_Os06g29470.1	6
3862	LOC_Os06g33080.1	6
3862	LOC_Os06g33090.1	6
3862	LOC_Os06g33100.1	6
3862	LOC_Os06g32960.1	6
3862	LOC_Os06g32980.1	6
3862	LOC_Os06g32990.1	6
3862	LOC_Os06g35490.1	6
3862	LOC_Os06g35520.1	6
3862	LOC_Os06g35480.1	6
3862	LOC_Os06g46799.1	6

3862	LOC_Os06g48000.1	6
3862	LOC_Os06g48010.1	6
3862	LOC_Os06g48020.1	6
3862	LOC_Os06g48030.1	6
3862	LOC_Os07g01420.1	7
3862	LOC_Os07g01370.1	7
3862	LOC_Os07g01380.1	7
3862	LOC_Os07g01400.1	7
3862	LOC_Os07g01410.1	7
3862	LOC_Os07g02440.1	7
3862	LOC_Os07g06175.1	7
3862	LOC_Os07g06286.1	7
3862	LOC_Os07g06380.1	7
3862	LOC_Os07g31610.1	7
3862	LOC_Os07g34670.1	7
3862	LOC_Os07g34710.1	7
3862	LOC_Os07g44460.1	7
3862	LOC_Os07g44590.1	7
3862	LOC_Os07g44550.1	7
3862	LOC_Os07g48010.1	7
3862	LOC_Os07g48020.1	7
3862	LOC_Os07g48040.1	7
3862	LOC_Os07g48050.1	7
3862	LOC_Os07g48060.1	7
3862	LOC_Os07g47990.1	7
3862	LOC_Os07g48030.1	7
3862	LOC_Os07g49360.1	7
3862	LOC_Os07g49400.1	7
3862	LOC_Os08g02110.1	8
3862	LOC_Os08g20730.1	8
3862	LOC_Os08g41090.1	8
3862	LOC_Os08g42030.1	8
3862	LOC_Os08g43560.1	8
3862	LOC_Os09g15500.1	9
3862	LOC_Os09g15510.1	9
3862	LOC_Os09g29490.1	9
3862	LOC_Os09g32964.1	9
3862	LOC_Os10g01760.1	10
3862	LOC_Os10g02040.1	10
3862	LOC_Os10g02070.1	10
3862	LOC_Os10g39170.1	10
3862	LOC_Os10g39160.1	10
3862	LOC_Os10g41720.1	10
3862	LOC_Os11g02130.1	11
3862	LOC_Os11g02100.1	11
3862	LOC_Os11g10460.1	11
3862	LOC_Os11g43980.1	11
3862	LOC_Os12g02060.1	12
3862	LOC_Os12g02080.1	12
3862	LOC_Os12g07820.1	12
3862	LOC_Os12g07830.1	12
3862	LOC_Os12g08920.1	12

3862	LOC_Os12g34524.1	12
3863	LOC_Os01g03660.1	1
3863	LOC_Os01g03720.1	1
3863	LOC_Os01g04930.1	1
3863	LOC_Os01g07450.1	1
3863	LOC_Os01g08160.1	1
3863	LOC_Os01g12700.1	1
3863	LOC_Os01g12860.1	1
3863	LOC_Os01g16810.1	1
3863	LOC_Os01g18240.1	1
3863	LOC_Os01g19330.1	1
3863	LOC_Os01g19970.1	1
3863	LOC_Os01g34060.1	1
3863	LOC_Os01g36460.1	1
3863	LOC_Os01g43180.1	1
3863	LOC_Os01g44370.1	1
3863	LOC_Os01g44390.1	1
3863	LOC_Os01g45090.1	1
3863	LOC_Os01g47370.1	1
3863	LOC_Os01g49160.1	1
3863	LOC_Os01g50110.1	1
3863	LOC_Os01g50720.1	1
3863	LOC_Os01g51260.1	1
3863	LOC_Os01g59660.1	1
3863	LOC_Os01g62410.1	1
3863	LOC_Os01g62660.1	1
3863	LOC_Os01g63160.1	1
3863	LOC_Os01g63680.1	1
3863	LOC_Os01g63460.1	1
3863	LOC_Os01g64360.1	1
3863	LOC_Os01g65370.1	1
3863	LOC_Os01g67770.1	1
3863	LOC_Os01g74020.1	1
3863	LOC_Os01g74590.1	1
3863	LOC_Os01g74410.1	1
3863	LOC_Os02g07170.1	2
3863	LOC_Os02g14490.1	2
3863	LOC_Os02g17190.1	2
3863	LOC_Os02g22020.1	2
3863	LOC_Os02g30700.1	2
3863	LOC_Os02g34630.1	2
3863	LOC_Os02g36890.1	2
3863	LOC_Os02g40530.1	2
3863	LOC_Os02g41510.1	2
3863	LOC_Os02g42850.1	2
3863	LOC_Os02g42870.1	2
3863	LOC_Os02g45080.1	2
3863	LOC_Os02g46030.1	2
3863	LOC_Os02g46780.1	2
3863	LOC_Os02g46940.1	2
3863	LOC_Os02g47190.1	2
3863	LOC_Os02g47744.1	2

3863	LOC_Os02g53670.1	2
3863	LOC_Os02g54520.1	2
3863	LOC_Os02g57270.1	2
3863	LOC_Os02g49986.1	2
3863	LOC_Os03g03760.1	3
3863	LOC_Os03g04900.1	3
3863	LOC_Os03g13310.1	3
3863	LOC_Os03g18480.1	3
3863	LOC_Os03g19120.1	3
3863	LOC_Os03g20090.1	3
3863	LOC_Os03g22560.1	3
3863	LOC_Os03g26130.1	3
3863	LOC_Os03g27090.1	3
3863	LOC_Os03g31230.1	3
3863	LOC_Os03g38210.1	3
3863	LOC_Os03g51110.1	3
3863	LOC_Os03g51220.1	3
3863	LOC_Os03g55590.1	3
3863	LOC_Os03g55760.1	3
3863	LOC_Os03g56090.1	3
3863	LOC_Os03g62100.1	3
3863	LOC_Os03g62379.1	3
3863	LOC_Os03g63890.1	3
3863	LOC_Os04g27410.1	4
3863	LOC_Os04g28090.1	4
3863	LOC_Os04g30890.1	4
3863	LOC_Os04g38740.1	4
3863	LOC_Os04g39470.1	4
3863	LOC_Os04g42950.1	4
3863	LOC_Os04g43680.1	4
3863	LOC_Os04g45020.1	4
3863	LOC_Os04g45060.1	4
3863	LOC_Os04g47890.1	4
3863	LOC_Os04g49450.1	4
3863	LOC_Os04g50680.1	4
3863	LOC_Os04g50770.1	4
3863	LOC_Os05g03550.1	5
3863	LOC_Os05g04210.1	5
3863	LOC_Os05g04820.1	5
3863	LOC_Os05g07010.1	5
3863	LOC_Os05g34110.1	5
3863	LOC_Os05g37040.1	5
3863	LOC_Os05g37050.1	5
3863	LOC_Os05g37060.1	5
3863	LOC_Os05g35500.1	5
3863	LOC_Os05g37730.1	5
3863	LOC_Os05g38460.1	5
3863	LOC_Os05g46330.1	5
3863	LOC_Os05g48010.1	5
3863	LOC_Os05g49310.1	5
3863	LOC_Os05g50340.1	5
3863	LOC_Os05g50350.1	5

3863	LOC_Os05g51160.1	5
3863	LOC_Os05g41166.1	5
3863	LOC_Os06g02250.1	6
3863	LOC_Os06g06740.1	6
3863	LOC_Os06g08290.1	6
3863	LOC_Os06g10350.1	6
3863	LOC_Os06g11780.1	6
3863	LOC_Os06g19980.1	6
3863	LOC_Os06g35140.1	6
3863	LOC_Os06g45410.1	6
3863	LOC_Os06g45890.1	6
3863	LOC_Os06g46560.1	6
3863	LOC_Os06g51260.1	6
3863	LOC_Os07g12130.1	7
3863	LOC_Os07g14110.1	7
3863	LOC_Os07g26150.1	7
3863	LOC_Os07g30130.1	7
3863	LOC_Os07g31470.1	7
3863	LOC_Os07g37210.1	7
3863	LOC_Os07g43420.1	7
3863	LOC_Os07g43580.1	7
3863	LOC_Os07g44090.1	7
3863	LOC_Os07g48870.1	7
3863	LOC_Os07g49530.1	7
3863	LOC_Os07g04700.1	7
3863	LOC_Os07g02800.1	7
3863	LOC_Os08g04840.1	8
3863	LOC_Os08g05510.1	8
3863	LOC_Os08g06110.1	8
3863	LOC_Os08g06240.1	8
3863	LOC_Os08g06370.1	8
3863	LOC_Os08g15020.1	8
3863	LOC_Os08g33050.1	8
3863	LOC_Os08g33150.1	8
3863	LOC_Os08g33800.1	8
3863	LOC_Os08g33660.1	8
3863	LOC_Os08g33940.1	8
3863	LOC_Os08g34960.1	8
3863	LOC_Os08g37970.1	8
3863	LOC_Os08g43550.1	8
3863	LOC_Os09g01960.1	9
3863	LOC_Os09g23620.1	9
3863	LOC_Os09g24800.1	9
3863	LOC_Os09g31454.1	9
3863	LOC_Os09g36250.1	9
3863	LOC_Os09g36730.1	9
3863	LOC_Os10g30719.1	10
3863	LOC_Os10g32600.1	10
3863	LOC_Os10g35660.1	10
3863	LOC_Os10g39550.1	10
3863	LOC_Os10g41260.1	10
3863	LOC_Os10g41200.1	10

3863	LOC_Os11g01480.1	11
3863	LOC_Os11g10130.1	11
3863	LOC_Os11g35390.1	11
3863	LOC_Os11g45740.1	11
3863	LOC_Os11g47460.1	11
3863	LOC_Os12g01490.1	12
3863	LOC_Os12g07610.1	12
3863	LOC_Os12g07640.1	12
3863	LOC_Os12g13570.1	12
3863	LOC_Os12g33950.1	12
3863	LOC_Os12g37690.1	12
3863	LOC_Os12g38400.1	12
3863	LOC_Os12g39640.1	12
3863	LOC_Os12g37970.1	12
3864	LOC_Os01g01410.1	1
3864	LOC_Os01g02460.1	1
3864	LOC_Os01g04550.1	1
3864	LOC_Os01g07940.1	1
3864	LOC_Os01g10430.1	1
3864	LOC_Os01g10840.1	1
3864	LOC_Os01g13060.1	1
3864	LOC_Os01g13270.1	1
3864	LOC_Os01g16230.1	1
3864	LOC_Os01g14860.1	1
3864	LOC_Os01g18150.1	1
3864	LOC_Os01g19150.1	1
3864	LOC_Os01g32660.1	1
3864	LOC_Os01g38840.1	1
3864	LOC_Os01g40840.1	1
3864	LOC_Os01g42400.1	1
3864	LOC_Os01g46030.1	1
3864	LOC_Os01g46720.1	1
3864	LOC_Os01g60700.1	1
3864	LOC_Os01g61620.1	1
3864	LOC_Os01g62080.1	1
3864	LOC_Os01g65230.1	1
3864	LOC_Os01g67160.1	1
3864	LOC_Os01g70130.1	1
3864	LOC_Os01g70260.1	1
3864	LOC_Os01g20900.1	1
3864	LOC_Os02g01730.1	2
3864	LOC_Os02g03060.1	2
3864	LOC_Os02g04240.1	2
3864	LOC_Os02g05480.1	2
3864	LOC_Os02g12130.1	2
3864	LOC_Os02g14130.1	2
3864	LOC_Os02g17910.1	2
3864	LOC_Os02g18430.1	2
3864	LOC_Os02g20140.1	2
3864	LOC_Os02g29070.1	2
3864	LOC_Os02g37830.1	2
3864	LOC_Os02g37880.1	2

3864	LOC_Os02g39010.1	2
3864	LOC_Os02g39090.1	2
3864	LOC_Os02g41580.1	2
3864	LOC_Os02g43870.1	2
3864	LOC_Os02g44610.1	2
3864	LOC_Os02g46760.1	2
3864	LOC_Os02g47410.1	2
3864	LOC_Os02g49040.1	2
3864	LOC_Os02g49310.1	2
3864	LOC_Os02g56310.1	2
3864	LOC_Os02g57440.1	2
3864	LOC_Os03g01850.1	3
3864	LOC_Os03g02190.1	3
3864	LOC_Os03g02680.1	3
3864	LOC_Os03g10940.1	3
3864	LOC_Os03g12390.1	3
3864	LOC_Os03g14840.1	3
3864	LOC_Os03g14710.1	3
3864	LOC_Os03g17700.1	3
3864	LOC_Os03g18170.1	3
3864	LOC_Os03g18430.1	3
3864	LOC_Os03g21770.1	3
3864	LOC_Os03g22700.1	3
3864	LOC_Os03g40400.1	3
3864	LOC_Os03g43590.1	3
3864	LOC_Os03g44020.1	3
3864	LOC_Os03g49750.1	3
3864	LOC_Os03g50230.1	3
3864	LOC_Os03g50550.1	3
3864	LOC_Os03g51020.1	3
3864	LOC_Os03g53250.1	3
3864	LOC_Os03g53880.1	3
3864	LOC_Os03g55389.1	3
3864	LOC_Os03g55490.1	3
3864	LOC_Os03g62500.1	3
3864	LOC_Os03g55620.1	3
3864	LOC_Os03g53720.1	3
3864	LOC_Os04g03370.1	4
3864	LOC_Os04g04670.1	4
3864	LOC_Os04g06210.1	4
3864	LOC_Os04g12180.1	4
3864	LOC_Os04g27100.1	4
3864	LOC_Os04g35114.1	4
3864	LOC_Os04g39180.1	4
3864	LOC_Os04g41100.1	4
3864	LOC_Os04g41160.1	4
3864	LOC_Os04g43710.1	4
3864	LOC_Os04g46320.1	4
3864	LOC_Os04g47570.1	4
3864	LOC_Os04g51370.1	4
3864	LOC_Os04g52630.1	4
3864	LOC_Os04g58990.1	4

3864	LOC_Os04g54190.1	4
3864	LOC_Os05g04340.1	5
3864	LOC_Os05g11730.1	5
3864	LOC_Os05g14750.1	5
3864	LOC_Os05g16360.1	5
3864	LOC_Os05g32360.1	5
3864	LOC_Os05g34950.1	5
3864	LOC_Os05g26940.1	5
3864	LOC_Os05g32170.1	5
3864	LOC_Os05g32600.1	5
3864	LOC_Os05g35770.1	5
3864	LOC_Os05g36960.1	5
3864	LOC_Os05g39080.1	5
3864	LOC_Os05g40180.1	5
3864	LOC_Os05g47560.1	5
3864	LOC_Os05g51400.1	5
3864	LOC_Os06g05520.1	6
3864	LOC_Os06g06090.1	6
3864	LOC_Os06g09180.1	6
3864	LOC_Os06g18830.1	6
3864	LOC_Os06g22820.1	6
3864	LOC_Os06g35530.1	6
3864	LOC_Os06g38100.1	6
3864	LOC_Os06g41980.1	6
3864	LOC_Os06g43270.1	6
3864	LOC_Os06g47880.1	6
3864	LOC_Os06g48590.1	6
3864	LOC_Os06g03682.1	6
3864	LOC_Os07g02350.1	7
3864	LOC_Os07g06570.1	7
3864	LOC_Os07g08000.1	7
3864	LOC_Os07g14490.1	7
3864	LOC_Os07g20610.1	7
3864	LOC_Os07g28820.1	7
3864	LOC_Os07g28930.1	7
3864	LOC_Os07g32480.1	7
3864	LOC_Os07g35770.1	7
3864	LOC_Os07g36980.1	7
3864	LOC_Os07g39520.1	7
3864	LOC_Os07g40550.1	7
3864	LOC_Os07g47950.1	7
3864	LOC_Os07g35730.1	7
3864	LOC_Os08g06060.1	8
3864	LOC_Os08g17320.1	8
3864	LOC_Os08g35220.1	8
3864	LOC_Os08g39460.1	8
3864	LOC_Os08g40170.1	8
3864	LOC_Os08g02050.1	8
3864	LOC_Os09g06230.1	9
3864	LOC_Os09g10720.1	9
3864	LOC_Os09g20640.1	9
3864	LOC_Os09g24340.1	9

3864	LOC_Os09g29170.1	9
3864	LOC_Os09g31210.1	9
3864	LOC_Os09g37949.1	9
3864	LOC_Os10g01390.1	10
3864	LOC_Os10g03480.1	10
3864	LOC_Os10g03490.1	10
3864	LOC_Os10g03990.1	10
3864	LOC_Os10g06510.1	10
3864	LOC_Os10g06930.1	10
3864	LOC_Os10g06950.1	10
3864	LOC_Os10g06560.1	10
3864	LOC_Os10g06580.1	10
3864	LOC_Os10g06770.1	10
3864	LOC_Os10g06780.1	10
3864	LOC_Os10g06800.1	10
3864	LOC_Os10g21090.1	10
3864	LOC_Os10g37740.1	10
3864	LOC_Os10g38950.1	10
3864	LOC_Os10g41290.1	10
3864	LOC_Os10g41390.1	10
3864	LOC_Os10g42950.1	10
3864	LOC_Os11g05320.1	11
3864	LOC_Os11g19490.1	11
3864	LOC_Os11g26140.1	11
3864	LOC_Os11g29510.1	11
3864	LOC_Os11g39450.1	11
3864	LOC_Os11g40240.1	11
3864	LOC_Os11g40430.1	11
3864	LOC_Os11g40370.1	11
3864	LOC_Os11g42440.1	11
3864	LOC_Os11g44260.1	11
3864	LOC_Os11g44550.1	11
3864	LOC_Os12g05394.1	12
3864	LOC_Os12g13310.1	12
3864	LOC_Os12g14480.1	12
3864	LOC_Os12g14610.1	12
3864	LOC_Os12g23930.1	12
3864	LOC_Os12g23700.1	12
3864	LOC_Os12g23940.1	12
3864	LOC_Os12g23950.1	12
3864	LOC_Os12g24140.1	12
3864	LOC_Os12g24420.1	12
3864	LOC_Os12g27520.1	12
3864	LOC_Os12g30300.1	12
3864	LOC_Os12g31610.1	12
3864	LOC_Os12g38860.1	12
3864	LOC_Os12g41180.1	12
3864	LOC_Os12g42020.1	12
3864	LOC_Os12g40419.1	12
3864	LOC_Os12g29580.1	12
3865	LOC_Os01g06590.1	1
3865	LOC_Os01g07390.1	1

3865	LOC_Os01g11460.1	1
3865	LOC_Os01g11480.1	1
3865	LOC_Os01g11490.1	1
3865	LOC_Os01g11500.1	1
3865	LOC_Os01g16120.1	1
3865	LOC_Os01g15630.1	1
3865	LOC_Os01g16950.1	1
3865	LOC_Os01g20910.1	1
3865	LOC_Os01g35100.1	1
3865	LOC_Os01g35120.1	1
3865	LOC_Os01g44240.1	1
3865	LOC_Os01g50750.1	1
3865	LOC_Os01g53130.1	1
3865	LOC_Os01g58780.1	1
3865	LOC_Os01g61470.1	1
3865	LOC_Os01g62640.1	1
3865	LOC_Os01g64620.1	1
3865	LOC_Os01g69040.1	1
3865	LOC_Os01g70160.1	1
3865	LOC_Os01g72480.1	1
3865	LOC_Os01g74610.1	1
3865	LOC_Os01g47740.1	1
3865	LOC_Os01g66970.1	1
3865	LOC_Os01g49770.1	1
3865	LOC_Os02g08200.1	2
3865	LOC_Os02g09820.1	2
3865	LOC_Os02g14990.1	2
3865	LOC_Os02g15000.1	2
3865	LOC_Os02g15010.1	2
3865	LOC_Os02g15020.1	2
3865	LOC_Os02g15060.1	2
3865	LOC_Os02g15100.1	2
3865	LOC_Os02g15080.1	2
3865	LOC_Os02g15110.1	2
3865	LOC_Os02g31150.1	2
3865	LOC_Os02g33720.1	2
3865	LOC_Os02g42690.1	2
3865	LOC_Os02g43120.1	2
3865	LOC_Os02g44700.1	2
3865	LOC_Os02g45710.1	2
3865	LOC_Os02g45780.1	2
3865	LOC_Os02g46100.1	2
3865	LOC_Os02g49550.1	2
3865	LOC_Os02g49710.1	2
3865	LOC_Os02g52870.1	2
3865	LOC_Os02g55520.1	2
3865	LOC_Os02g55480.1	2
3865	LOC_Os02g58540.1	2
3865	LOC_Os02g35144.1	2
3865	LOC_Os02g05692.1	2
3865	LOC_Os03g01790.1	3
3865	LOC_Os03g10890.1	3

3865	LOC_Os03g05560.1	3
3865	LOC_Os03g07790.1	3
3865	LOC_Os03g17170.1	3
3865	LOC_Os03g20870.1	3
3865	LOC_Os03g22080.1	3
3865	LOC_Os03g22110.1	3
3865	LOC_Os03g22830.1	3
3865	LOC_Os03g26300.1	3
3865	LOC_Os03g26370.1	3
3865	LOC_Os03g26420.1	3
3865	LOC_Os03g28040.1	3
3865	LOC_Os03g40170.1	3
3865	LOC_Os03g43360.1	3
3865	LOC_Os03g44636.1	3
3865	LOC_Os03g44642.1	3
3865	LOC_Os03g47500.1	3
3865	LOC_Os03g53080.1	3
3865	LOC_Os03g57500.1	3
3865	LOC_Os03g58390.1	3
3865	LOC_Os03g16570.1	3
3865	LOC_Os03g62250.1	3
3865	LOC_Os04g01160.1	4
3865	LOC_Os04g10680.1	4
3865	LOC_Os04g16970.1	4
3865	LOC_Os04g34230.1	4
3865	LOC_Os04g37740.1	4
3865	LOC_Os04g41050.1	4
3865	LOC_Os04g41070.1	4
3865	LOC_Os04g41080.1	4
3865	LOC_Os04g43220.1	4
3865	LOC_Os04g44820.1	4
3865	LOC_Os04g46450.1	4
3865	LOC_Os04g49000.1	4
3865	LOC_Os04g49160.1	4
3865	LOC_Os04g49170.1	4
3865	LOC_Os04g49700.1	4
3865	LOC_Os04g51400.1	4
3865	LOC_Os04g55510.1	4
3865	LOC_Os04g59580.1	4
3865	LOC_Os05g06270.1	5
3865	LOC_Os05g07140.1	5
3865	LOC_Os05g11720.1	5
3865	LOC_Os05g11860.1	5
3865	LOC_Os05g25180.1	5
3865	LOC_Os05g28730.1	5
3865	LOC_Os05g32350.1	5
3865	LOC_Os05g15170.1	5
3865	LOC_Os05g41520.1	5
3865	LOC_Os05g33830.1	5
3865	LOC_Os05g36310.1	5
3865	LOC_Os05g37900.1	5
3865	LOC_Os05g39260.1	5

3865	LOC_Os05g39380.1	5
3865	LOC_Os05g39940.1	5
3865	LOC_Os05g40020.1	5
3865	LOC_Os05g40980.1	5
3865	LOC_Os05g47670.1	5
3865	LOC_Os05g48970.1	5
3865	LOC_Os05g51780.1	5
3865	LOC_Os05g07070.1	5
3865	LOC_Os06g01200.1	6
3865	LOC_Os06g05200.1	6
3865	LOC_Os06g06150.1	6
3865	LOC_Os06g09310.1	6
3865	LOC_Os06g14200.1	6
3865	LOC_Os06g14640.1	6
3865	LOC_Os06g14650.1	6
3865	LOC_Os06g16060.1	6
3865	LOC_Os06g19680.1	6
3865	LOC_Os06g34360.1	6
3865	LOC_Os06g34390.1	6
3865	LOC_Os06g34450.1	6
3865	LOC_Os06g34640.1	6
3865	LOC_Os06g34650.1	6
3865	LOC_Os06g34880.1	6
3865	LOC_Os06g34400.1	6
3865	LOC_Os06g34430.1	6
3865	LOC_Os06g34470.1	6
3865	LOC_Os06g34530.1	6
3865	LOC_Os06g34560.1	6
3865	LOC_Os06g42700.1	6
3865	LOC_Os06g45580.1	6
3865	LOC_Os06g47270.1	6
3865	LOC_Os06g48040.1	6
3865	LOC_Os06g43210.1	6
3865	LOC_Os07g06560.1	7
3865	LOC_Os07g06540.1	7
3865	LOC_Os07g22840.1	7
3865	LOC_Os07g31850.1	7
3865	LOC_Os07g43380.1	7
3865	LOC_Os07g43740.1	7
3865	LOC_Os07g48680.1	7
3865	LOC_Os08g01040.1	8
3865	LOC_Os08g05560.1	8
3865	LOC_Os08g06090.1	8
3865	LOC_Os08g06510.1	8
3865	LOC_Os08g14320.1	8
3865	LOC_Os08g31720.1	8
3865	LOC_Os08g38460.1	8
3865	LOC_Os08g41520.1	8
3865	LOC_Os08g42640.1	8
3865	LOC_Os08g44950.1	8
3865	LOC_Os09g06740.1	9
3865	LOC_Os09g12720.1	9

3865	LOC_Os09g17610.1	9
3865	LOC_Os09g26400.1	9
3865	LOC_Os09g27380.1	9
3865	LOC_Os09g30160.1	9
3865	LOC_Os09g32690.1	9
3865	LOC_Os09g32730.1	9
3865	LOC_Os09g33670.1	9
3865	LOC_Os09g36500.1	9
3865	LOC_Os09g38110.1	9
3865	LOC_Os10g05230.1	10
3865	LOC_Os10g20600.1	10
3865	LOC_Os10g30310.1	10
3865	LOC_Os10g34030.1	10
3865	LOC_Os10g34590.1	10
3865	LOC_Os10g39450.1	10
3865	LOC_Os10g39936.1	10
3865	LOC_Os10g39850.1	10
3865	LOC_Os10g41590.1	10
3865	LOC_Os10g41660.1	10
3865	LOC_Os10g42390.1	10
3865	LOC_Os11g02250.1	11
3865	LOC_Os11g02260.1	11
3865	LOC_Os11g04280.1	11
3865	LOC_Os11g04281.1	11
3865	LOC_Os11g04680.1	11
3865	LOC_Os11g04690.1	11
3865	LOC_Os11g05200.1	11
3865	LOC_Os11g05230.1	11
3865	LOC_Os11g07450.1	11
3865	LOC_Os11g36430.1	11
3865	LOC_Os11g36970.1	11
3865	LOC_Os11g37230.1	11
3865	LOC_Os11g39640.1	11
3865	LOC_Os11g41140.1	11
3865	LOC_Os11g47690.1	11
3865	LOC_Os11g47700.1	11
3865	LOC_Os11g02670.1	11
3865	LOC_Os11g18947.1	11
3865	LOC_Os12g01750.1	12
3865	LOC_Os12g02220.1	12
3865	LOC_Os12g02350.1	12
3865	LOC_Os12g04090.1	12
3865	LOC_Os12g04450.1	12
3865	LOC_Os12g04460.1	12
3865	LOC_Os12g04590.1	12
3865	LOC_Os12g04650.1	12
3865	LOC_Os12g04660.1	12
3865	LOC_Os12g05270.1	12
3865	LOC_Os12g16690.1	12
3865	LOC_Os12g24490.1	12
3865	LOC_Os12g24530.1	12
3865	LOC_Os12g39110.1	12

3865	LOC_Os12g40460.1	12
3865	LOC_Os12g42540.1	12
3865	LOC_Os12g43930.1	12
3865	LOC_Os12g02620.1	12
