# Supplementary material for: Characterization of paralogous protein families in rice
Source: BMC Plant Biol. 2008 Feb 19;8:18. doi: 10.1186/1471-2229-8-18 (PMC2275729; doi:10.1186/1471-2229-8-18)
Supplement: Additional File 2 — Rice paralogous protein families with more than one hundred member proteins. [file 1471-2229-8-18-S2.pdf]

Additional file 2. Rice paralogous protein families with more than a hundred member proteins

Family_ID	Family_size	MPSS-qualifying proteins	Putative Function
3865	214	121	Zinc finger, C3HC4 type family protein
3864	192	102	Protein kinase domain containing protein
3863	169	90	Myb-like DNA-binding domain containing protein
3862	147	82	Peroxidase
3861	145	2	Hypothetical protein
3860	133	101	Transducin family protein
3859	132	66	AP2 domain containing protein
3858	126	79	RNA recognition motif family protein
3857	124	35	F-box domain containing protein
3856	123	60	Protease inhibitor/seed storage/LTP family protein
3855	108	73	GDSL-like lipase/acylhydrolase family protein
