# Supplementary figures and images for: Characterization of paralogous protein families in rice
Source: BMC Plant Biol. 2008 Feb 19;8:18. doi: 10.1186/1471-2229-8-18 (PMC2275729; doi:10.1186/1471-2229-8-18)

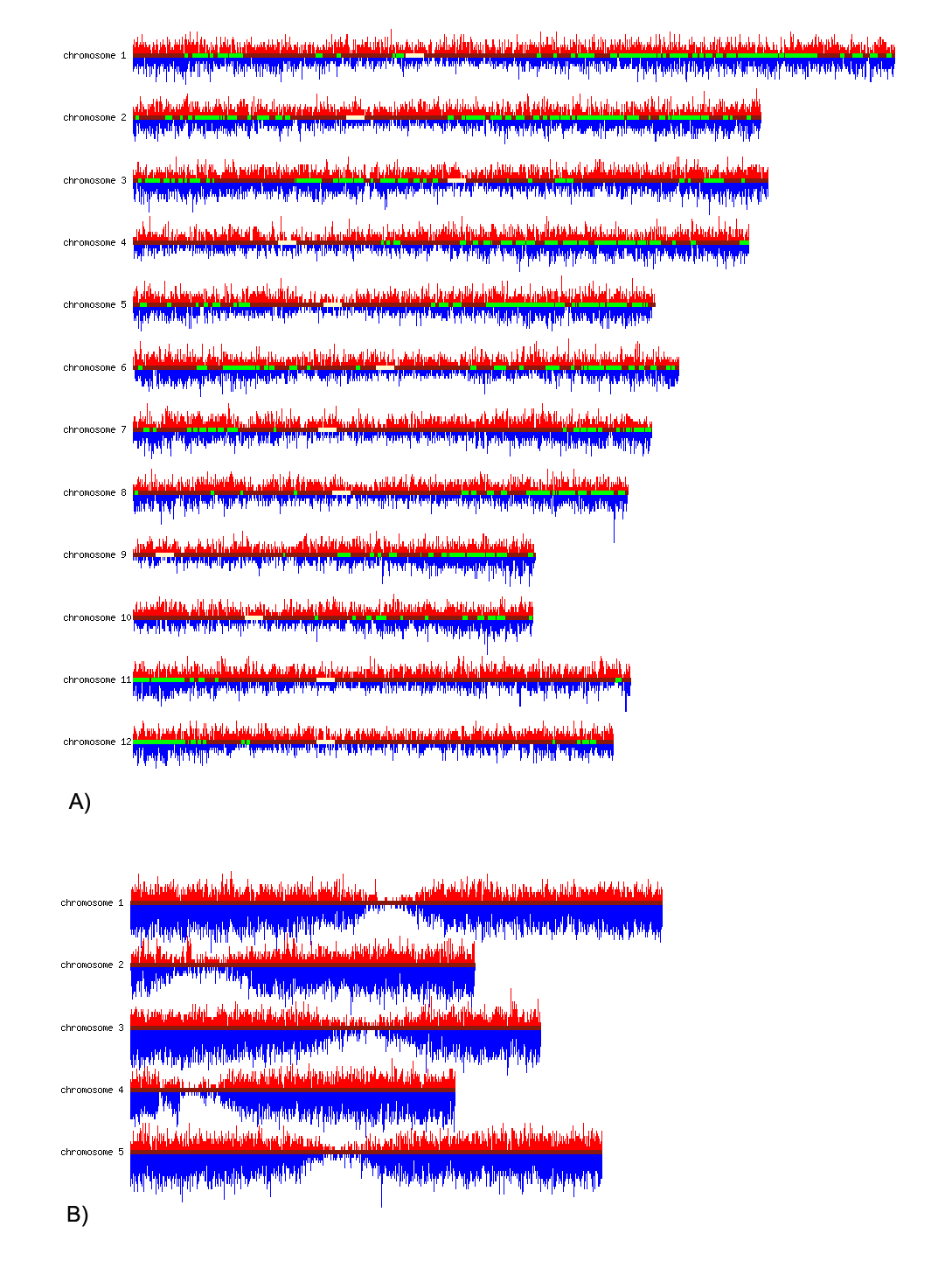

Supplement: Additional File 3 — Distribution of non-transposable element-related genes in rice and Arabidopsis. In panel A, the 12 rice chromosomes are shown with paralogous gene family members plotted in blue while single copy genes are plotted in red. Segmental duplicated blocks are indicated in green and centromeres are denoted by a white box. In panel B, the five Arabidopsis chromosomes are shown with paralogous gene family members plotted in blue while single copy genes are plotted in red. [file 1471-2229-8-18-S3.tiff]
