# Supplementary material for: Characterization of paralogous protein families in rice
Source: BMC Plant Biol. 2008 Feb 19;8:18. doi: 10.1186/1471-2229-8-18 (PMC2275729; doi:10.1186/1471-2229-8-18)

Additional file 4. The age distribution of rice paralogous protein families. **A)** an expanded view of the age distribution. **B)** an enlarged distribution of rice paralogous protein families with largest *d*s ≤ 1.5.

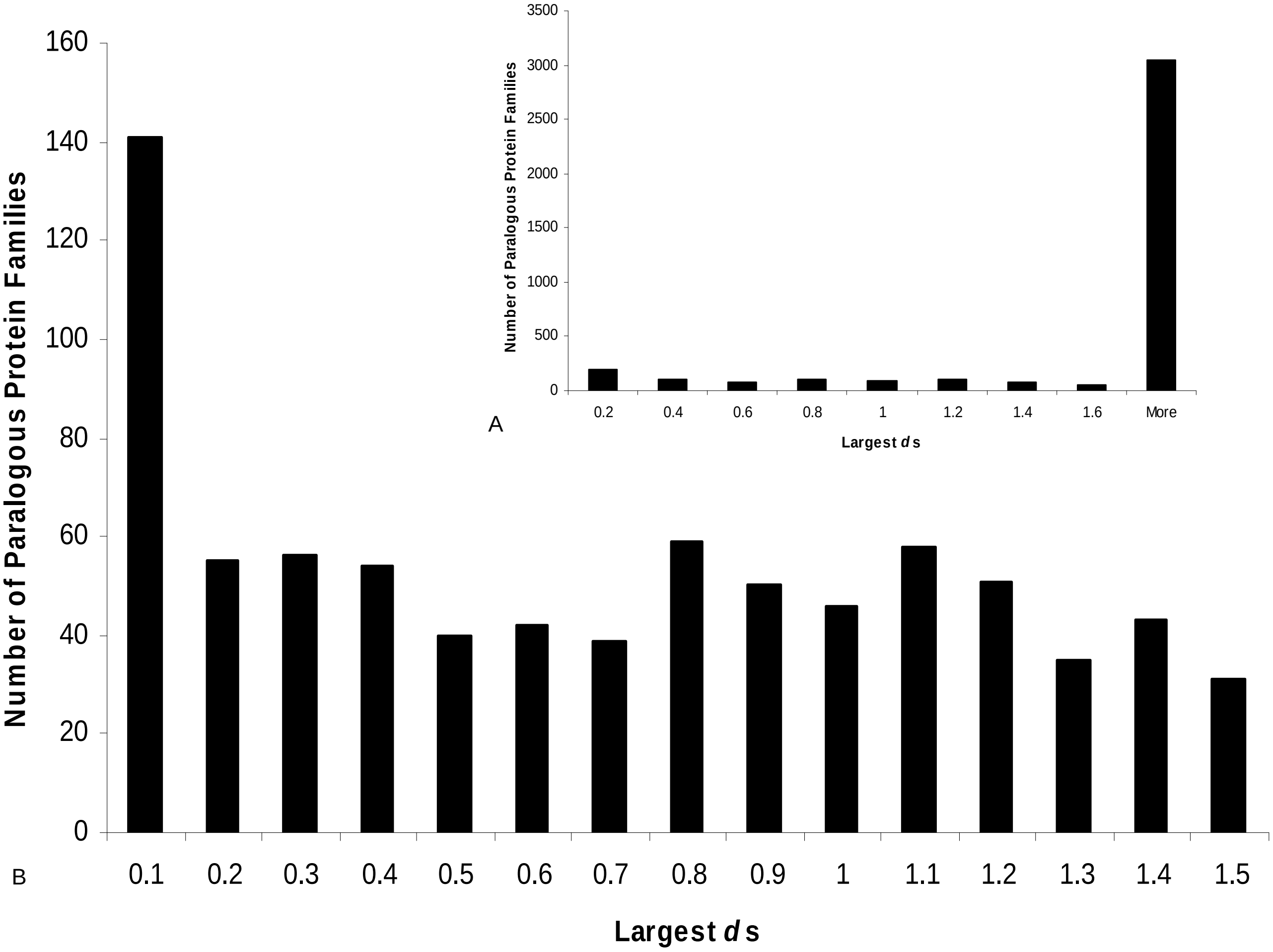

Supplement: Additional File 4 — The age distribution of rice paralogous protein families. A) an expanded view of the age distribution. B) the enlarged distribution of rice paralogous protein families with largest ds ≤ 1.5. [file 1471-2229-8-18-S4.pdf]
