# Supplementary material for: Characterization of paralogous protein families in rice
Source: BMC Plant Biol. 2008 Feb 19;8:18. doi: 10.1186/1471-2229-8-18 (PMC2275729; doi:10.1186/1471-2229-8-18)

**Additional file 5**. Distribution of modal values under $d_S$ = 1.5 across rice paralogous protein families. Of all 3,865 paralogous protein families, 2,388 showed a peak under 1.5 in the distribution of all pairwise $d_S$ values and are plotted.

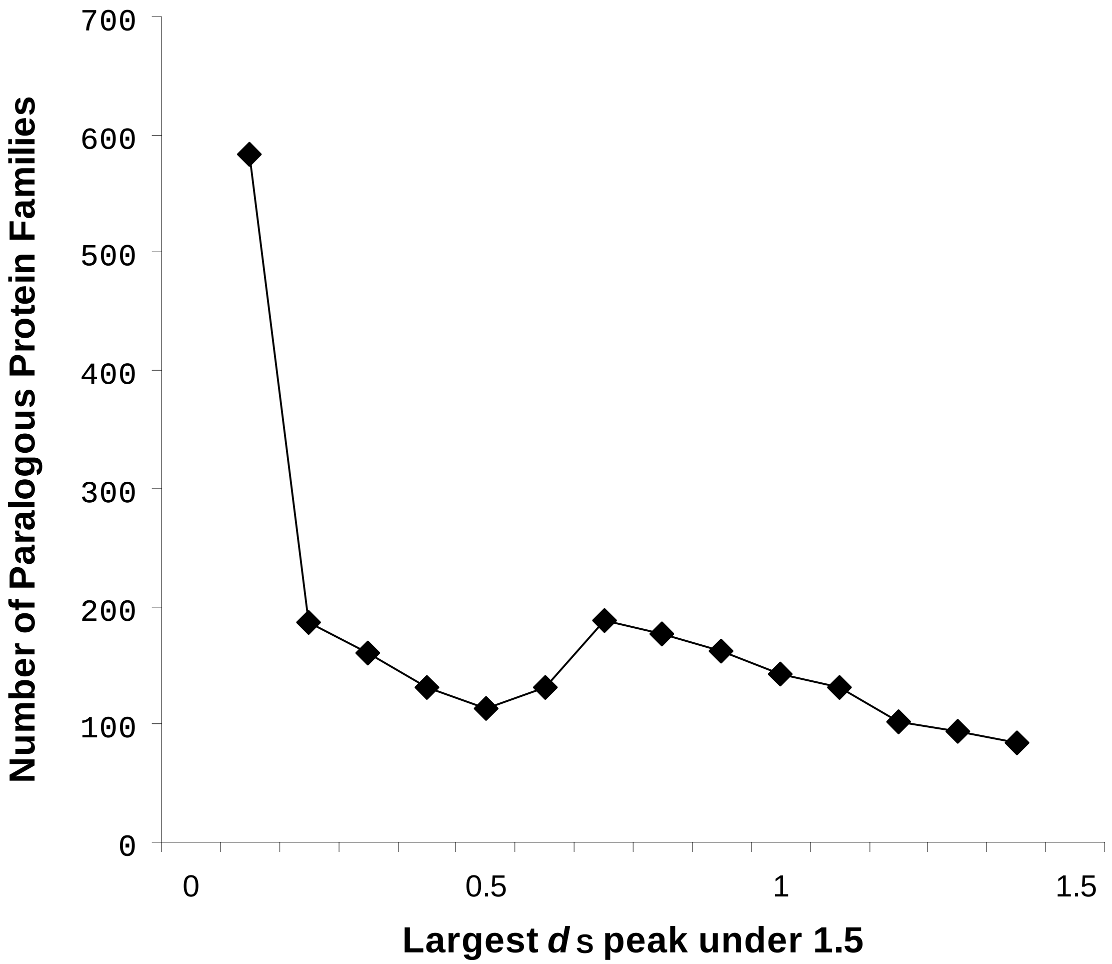

Supplement: Additional File 5 — Distribution of modal values under dS ≤ 1.5 across rice paralogous protein families. Of all 3,865 paralogous protein families, 2,388 showed a peak under 1.5 in the distribution of all pairwise dS values and are plotted. [file 1471-2229-8-18-S5.pdf]
