# Supplementary material for: Characterization of paralogous protein families in rice
Source: BMC Plant Biol. 2008 Feb 19;8:18. doi: 10.1186/1471-2229-8-18 (PMC2275729; doi:10.1186/1471-2229-8-18)
Supplement: Additional File 6 — Pearson's correlation coefficient (r) versus ds values. A) 0 <dS ≤ 0.1; B) 0.4 <dS ≤ 0.5; C) 1.0 <dS ≤ 1.1; D) 1.4 <dS ≤ 1.5. [file 1471-2229-8-18-S6.pdf]

Additional file 6. Pearson's correlation coefficient (*r*) versus *ds* values. **A)** $0 < d_S \leq 0.1$; **B)** $0.4 < d_S \leq 0.5$; **C)** $1.0 < d_S \leq 1.1$; **D)** $1.4 < d_S \leq 1.5$. Only very young genes showed correlation between correlation of expression and *ds* values.

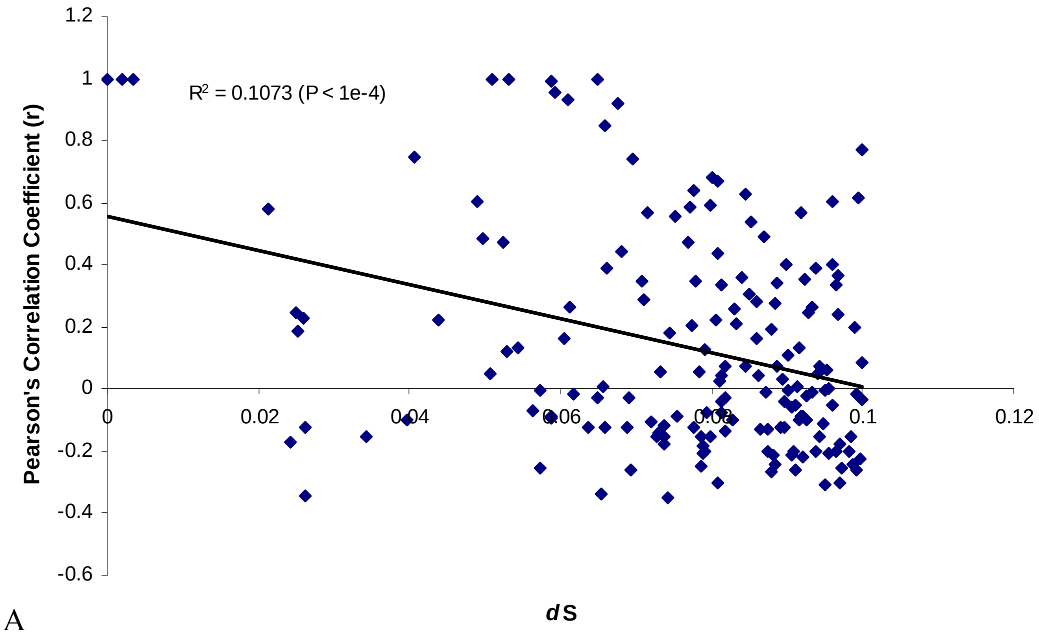

A

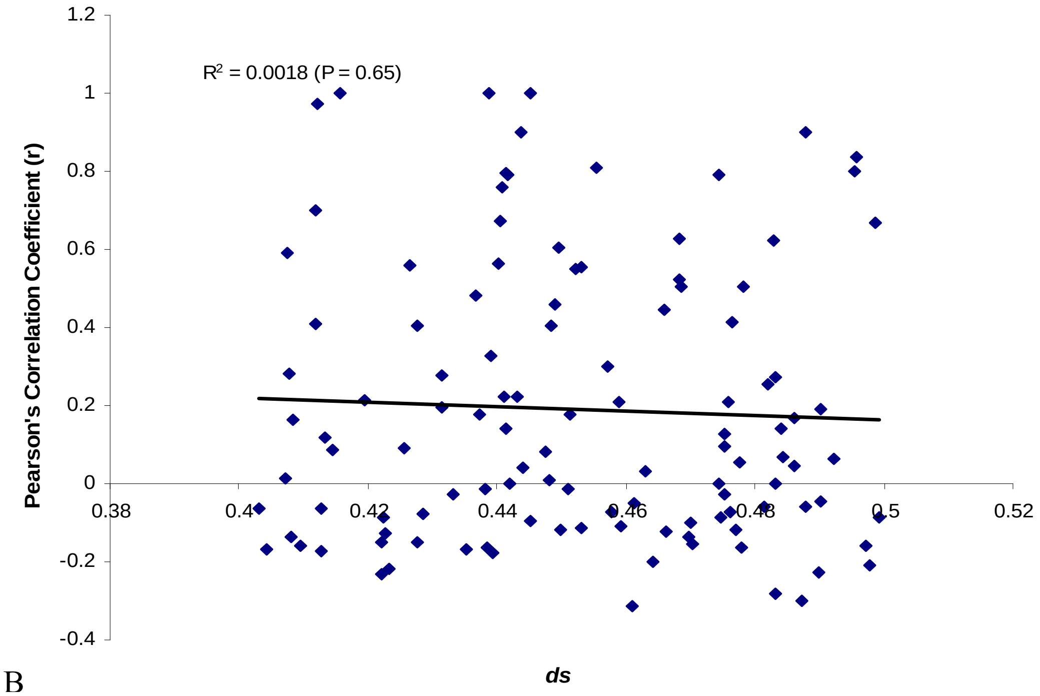

B

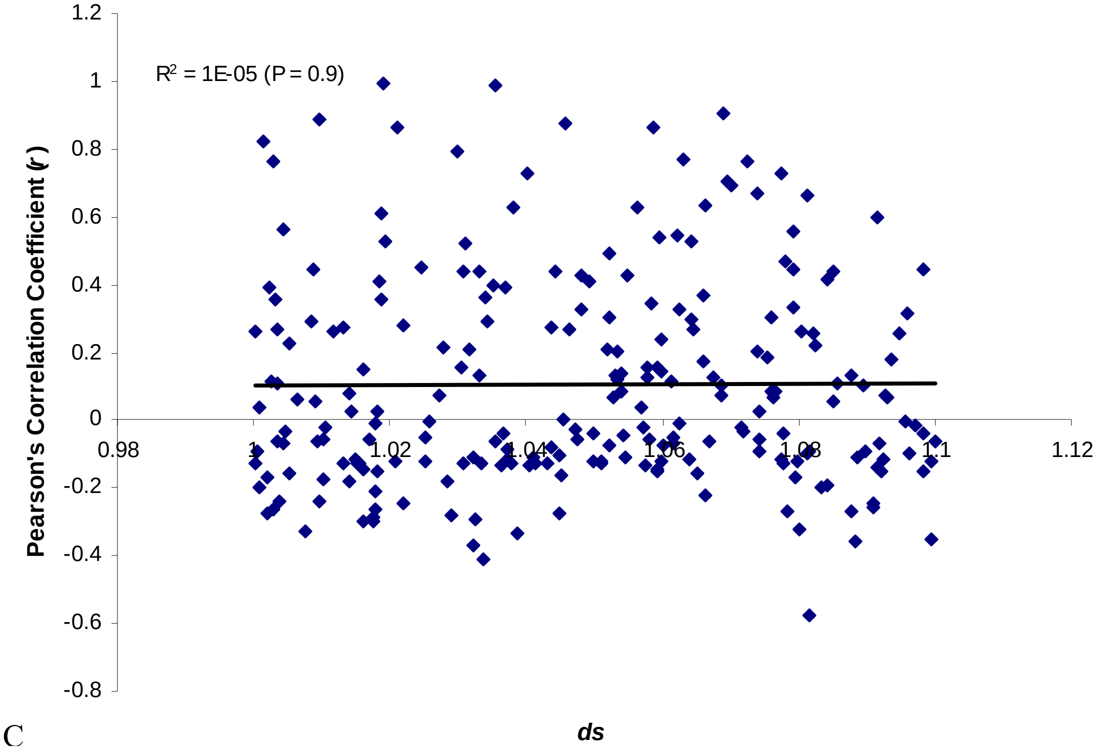

R^2 = 1E-05 (P = 0.9)
Pearson's Correlation Coefficient (r)
ds
1.2
1
0.8
0.6
0.4
0.2
0
-0.2
-0.4
-0.6
-0.8
0.98
1
1.02
1.04
1.06
1.08
1.1
1.12

C

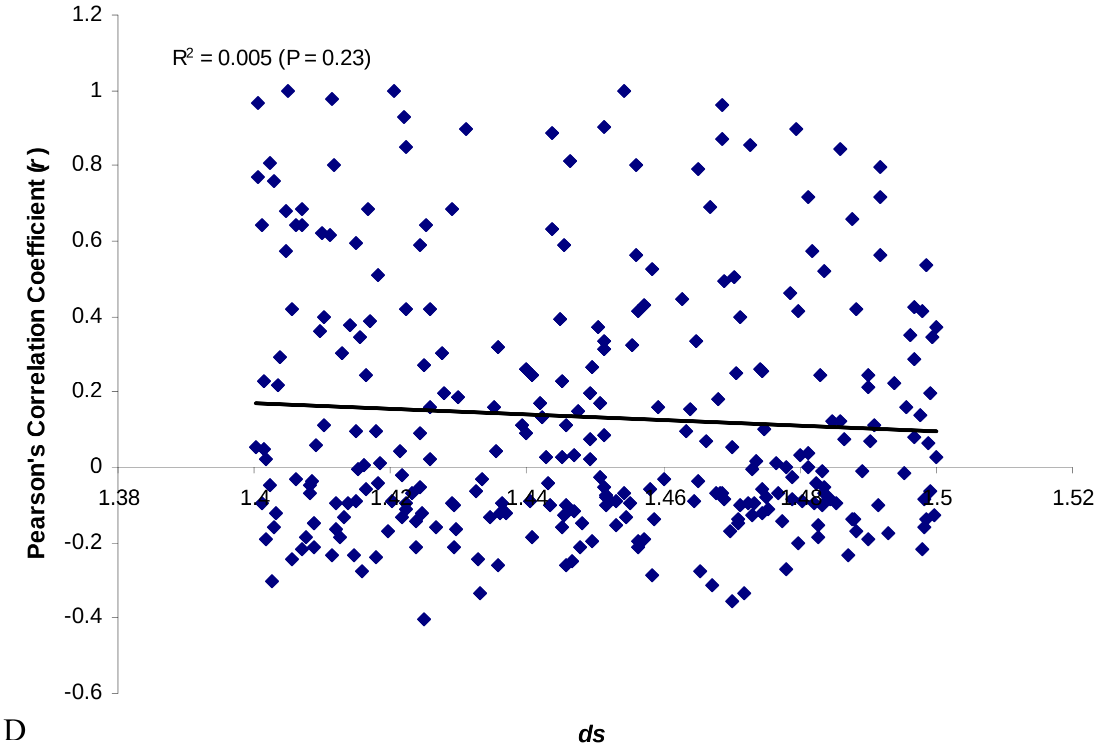

R^2 = 0.005 (P = 0.23)
Pearson's Correlation Coefficient (r)
ds
1.2
1
0.8
0.6
0.4
0.2
0
-0.2
-0.4
-0.6
1.38
1.4
1.42
1.44
1.46
1.48
1.5
1.52

D
