# Supplementary material for: Characterization of paralogous protein families in rice
Source: BMC Plant Biol. 2008 Feb 19;8:18. doi: 10.1186/1471-2229-8-18 (PMC2275729; doi:10.1186/1471-2229-8-18)

Additional file 7. Schematic illustration of the domain composition of three related rice paralogous protein families: Family 3722, Family 3193, and Family 3856.

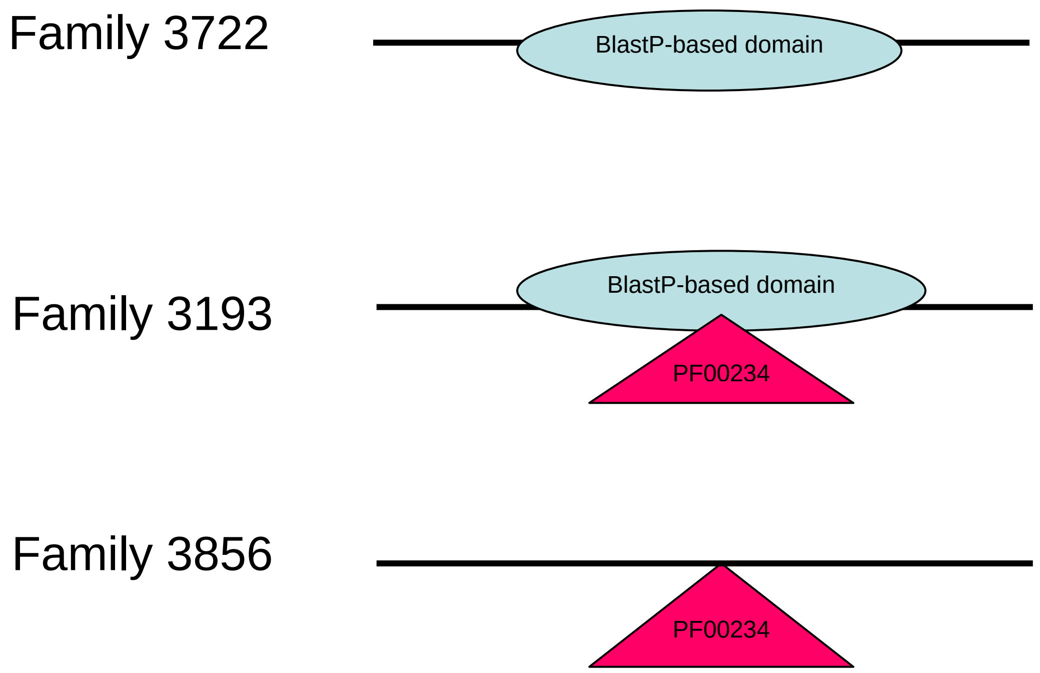

Supplement: Additional File 7 — Schematic illustration of the domain composition of three related rice paralogous protein families: Family 3722, Family 3193, and Family 3856. [file 1471-2229-8-18-S7.pdf]
