# Supplementary material for: Characterization of paralogous protein families in rice
Source: BMC Plant Biol. 2008 Feb 19;8:18. doi: 10.1186/1471-2229-8-18 (PMC2275729; doi:10.1186/1471-2229-8-18)
Supplement: Additional File 8 — Expression abundance of the rice prolamin genes from Family 3722 and Family 3193 in 18 libraries which were associated with unique, reliable, and significant MPSS tags. [file 1471-2229-8-18-S8.pdf]

Additional file 8. Expression abundance of the rice prolamin genes from Family 3722 and Family 3193 in 18 libraries which were associated with unique, reliable, and significant MPSS tags.

genes	MPSS tags	NYR	NR2	NYL	NL4	NST	NGS	NGD	NME	NPO	NSO	NIP	NCA	NSR	NSL	NDR	NDL	NCR	NCL
LOC_Os05g26720.1	GATCTCTACTTTGGTCG	0	0	0	0	0	198	0	0	0	0	0	0	0	0	0	0	0	0
LOC_Os05g26770.1	GATCGGAATCTGGGTCA	0	0	0	0	0	562	0	0	0	0	0	0	0	0	0	0	0	0
LOC_Os06g31070.1	GATCATGAACCCGTGCA	0	0	0	0	0	1042	0	0	0	0	0	0	0	0	0	0	0	0
LOC_Os12g16880.1	GATCAGACTCAAGCTCT	0	0	0	0	0	148	0	0	0	0	0	0	0	0	0	0	0	0
LOC_Os12g16890.1	GATCAGAGTCAAGCTCA	0	0	0	0	0	670	0	0	0	0	0	0	0	0	0	0	0	0

Library	Description
NCA	35 days - Callus
NCL	14 days - Young leaves stressed in 4C cold for 24h
NCR	14 days - Young roots stressed in 4C cold for 24h
NDL	14 days - Young leaves stressed in drought for 5 days
NDR	14 days - Young roots stressed in drought for 5 days
NGD	10 days - Germinating seedlings grown in dark
NGS	3 days - Germinating seed
NIP	90 days - Immature panicle
NL4	Leaves combined
NME	60 days - Crown vegetative meristematic tissue
NOS	Ovary and mature stigma
NPO	Mature Pollen
NR2	Root combined
NSL	14 days - Young leaves stressed in 250 mM NaCl for 24h
NSR	14 days - Young roots stressed in 250 mM NaCl for 24h
NST	60 days - Stem
NYL	14 days - Young leaves
NYR	14 days - Young Roots
