# Supplementary material for: Characterization of paralogous protein families in rice
Source: BMC Plant Biol. 2008 Feb 19;8:18. doi: 10.1186/1471-2229-8-18 (PMC2275729; doi:10.1186/1471-2229-8-18)
Supplement: Additional File 9 — Expression abundance of genes from rice paralogous protein family Family 3856 (contained PF00234) in 18 libraries which were associated with unique, reliable, and significant MPSS tags. [file 1471-2229-8-18-S9.pdf]

Addtional file 9. Expression abundance of rice genes from paralogous protein family Family 3856 (contained PF00234) in 18 libraries which were associated with unique, reliable, and significant MPSS tags. Genes which were exclusively expressed in the 3-day seeds were highlighted.

gene_model	tag	NYR	NR2	NYL	NL4	NST	NGS	NGD	NME	NPO	NSO	NIP	NCA	NSR	NSL	NDR	NDL	NCR	NCL
LOC_Os01g49640.1	GATCTTTTGTTCTCCTG	0	0	0	0	0	20	0	0	0	0	0	193	0	0	36	0	0	0
LOC_Os01g49650.1	GATCGCCATCGCCGCGC	0	0	0	0	0	0	0	0	0	0	1280	0	0	0	0	0	0	0
LOC_Os01g59870.1	GATCGGCCGTAAGGCGC	0	0	0	0	0	0	0	0	0	0	5	0	0	0	0	0	0	0
LOC_Os01g62980.1	GATCGCAGCACGGCTCA	0	0	0	11	0	0	0	0	0	0	0	0	0	0	0	0	0	0
LOC_Os01g68589.1	GATCTTATTTTGTTCTG	6	0	0	0	0	6	0	0	0	14	0	3	0	0	0	0	0	0
LOC_Os01g60740.1	GATCGTATGTCTCCACA	0	0	0	0	218	78	0	113	0	568	1626	4543	0	0	59	0	0	0
LOC_Os02g24720.1	GATCGACGGCAGCAACA	0	0	0	0	0	0	16	0	0	0	0	0	0	0	0	0	0	0
LOC_Os02g44310.1	GATCGTGCATGCTGGCT	463	1008	0	0	0	284	0	64	0	0	0	36	405	0	502	0	47	0
LOC_Os03g01300.1	GATCTCTGTATCTTGTT	7	59	0	0	0	84	0	0	0	0	0	10	0	0	39	0	0	0
LOC_Os03g02050.1	GATCGACCGATGTTATG	0	0	0	0	0	154	0	0	0	0	0	149	0	19	0	0	0	0
LOC_Os03g07100.1	GATCAATTAGTGGGCGA	0	5	0	0	0	55	0	3	0	62	237	0	0	0	10	0	0	0
LOC_Os03g09230.1	GATCGTCATCGCTATCG	0	2	42	0	17	0	6	28	0	0	12	0	9	13	2	0	0	13
LOC_Os03g26820.1	GATCGTGATTAGACTCG	9	79	406	190	228	73	54	327	15	470	383	14	0	755	85	216	17	376
LOC_Os03g46180.1	GATCGAGCGCTGGTTCA	0	0	0	0	0	0	0	0	0	0	0	0	0	0	11	0	0	0
LOC_Os03g50960.1	GATCCCCGTCAACCTGC	0	0	0	0	0	0	0	2	0	0	0	0	0	0	0	0	0	4
LOC_Os03g57970.1	GATCAACGGCGGCGCGT	1757	304	5	0	281	156	404	388	0	0	225	0	942	10	456	196	246	0
LOC_Os03g57980.1	GATCATCCAGGGGCAGA	179	108	1	0	0	10	5	8	0	0	56	0	86	0	194	2	0	5
LOC_Os03g57990.1	GATCACTTTTGAGTTCT	13	1	0	0	0	2	0	0	0	0	0	0	9	0	4	0	0	0
LOC_Os03g59380.1	GATCTCAGATTTGAGAT	0	0	0	0	0	0	0	0	7	29	319	0	0	0	0	0	0	0
LOC_Os04g46810.1	GATCCAGCGGCAGCAAT	0	5	0	0	0	0	0	0	0	0	0	0	0	0	0	0	0	0
LOC_Os04g46830.1	GATCGTTTGTTCTGTTT	0	0	0	0	90	123	49	3	0	0	47	0	0	0	0	22	0	0
LOC_Os05g06780.1	GATCAAGCCGGAGGTCG	420	38	0	0	499	0	377	760	0	0	595	3	0	0	481	0	56	0
LOC_Os05g40010.1	GATCACGCTGTATGTTC	0	0	0	0	0	0	0	0	0	0	0	0	0	0	0	0	0	10
LOC_Os05g41970.1	GATCAAAACAAATAATG	0	0	0	0	0	80	0	0	0	0	0	0	0	0	0	0	0	0
LOC_Os05g47700.1	GATCGTGCCGTGTATTG	845	959	7	2	8	92	102	814	12	4	139	0	759	0	1002	10	3	0
LOC_Os05g47730.1	GATCGTGGGGAACTCGC	0	0	0	0	2	978	70	0	0	0	0	124	0	34	0	0	6	0
LOC_Os06g49190.1	GATCATTGTGTGCATGT	0	0	0	0	2	20	495	0	2	0	0	0	0	0	0	0	0	0
LOC_Os06g49770.1	GATCAGTTCCTCGGTTG	31	3	0	0	367	165	0	132	0	10	827	0	35	0	53	2	0	0
LOC_Os06g47200.1	GATCAAAGCGTAATGGA	100	36	26	0	88	46	2	0	141	189	9	103	56	80	81	18	0	28
LOC_Os07g07790.1	GATCCTCGCGGCGAGCC	75	0	0	0	0	0	0	0	0	0	0	0	0	0	5	0	0	0
LOC_Os07g07860.1	GATCGTGTCGGCTGCGT	964	173	0	0	13	5	11	0	0	0	8	0	661	0	487	0	567	0
LOC_Os07g07920.1	GATCAACACGAGCGCCA	60	0	0	0	0	0	1	0	0	0	0	0	0	0	0	0	0	0
LOC_Os07g07930.1	GATCAGAGGAGCTTGTT	694	28	79	0	44	20	4	61	0	0	34	27	404	59	203	76	0	1
LOC_Os07g09970.1	GATCAGCTAGCTCTAGT	0	0	0	0	112	0	0	0	0	0	0	0	0	0	29	389	0	0
LOC_Os07g11310.1	GATCGCGCCGTTGCCAC	1	0	0	0	0	3479	0	11	0	0	0	0	0	0	0	0	0	0
LOC_Os07g11330.1	GATCACCGTGGCCAGGT	0	0	0	0	0	2431	0	0	0	0	0	0	0	0	0	0	0	0
LOC_Os07g11360.1	GATCACATGCTGTCAGG	0	0	0	0	0	2118	0	0	0	0	0	0	0	0	0	0	0	0
LOC_Os07g11410.1	GATCATCTACAGGGAGA	6	0	0	0	0	6489	0	44	0	0	0	0	31	0	0	0	6	0
LOC_Os07g11510.1	GATCTGGCTACTAGCTA	14	0	0	0	0	2816	0	0	0	0	0	0	0	0	0	0	0	0
LOC_Os07g12080.1	GATCATCACATGAATGC	0	0	0	0	0	45	0	0	0	0	0	0	0	0	0	0	0	0
LOC_Os07g18750.1	GATCGCATGCCTAATAT	0	6	0	0	1	21	0	3	0	2	0	0	0	0	83	0	0	0
LOC_Os07g18990.1	GATCGATGAGCCCGGCA	0	59	0	53	0	0	0	38	0	0	0	0	0	0	32	0	4	0

LOC_Os07g43290.1	GATCTGCTAATGTTGAC	20	10	2	1	2	79	1	0	23	43	32	73	14	25	37	11	0	8
LOC_Os08g42040.1	GATCTCTACGTCGTTCA	0	0	0	0	322	430	18	13	0	0	196	0	0	0	0	0	0	0
LOC_Os08g43290.1	GATCAACTGCTCTGCCT	40	0	0	0	0	0	0	1	0	0	3116	0	14	0	19	0	0	0
LOC_Os09g35700.1	GATCGTTCAATGAACGA	0	0	0	0	0	0	0	0	0	0	923	0	0	0	0	0	0	0
LOC_Os10g05720.1	GATCCAAGGTCTAAAAC	0	0	0	0	0	0	0	0	0	0	60	0	0	0	0	0	0	0
LOC_Os10g11750.1	GATCTTGTTCCTGCTTC	0	0	0	0	0	0	0	0	94	0	0	0	0	0	0	0	0	0
LOC_Os10g20890.1	GATCGGTGTGAAGATTG	0	0	0	0	26	0	0	0	0	0	0	0	0	0	0	0	0	0
LOC_Os10g36160.1	GATCGATGATGTGCCTT	0	0	0	0	11	4	0	0	0	0	0	0	0	0	0	0	0	0
LOC_Os10g36170.1	GATCATCGGCGGGTCGA	0	0	0	0	0	24	154	0	87	0	5	0	0	0	0	36	0	0
LOC_Os10g40420.1	GATCGTGTAGCGCATGC	0	0	0	0	226	10	75	0	0	0	57	0	1	0	0	65	0	0
LOC_Os10g40440.1	GATCTGTCCCTCCGATT	0	0	0	0	0	260	0	0	0	0	0	66	0	0	0	0	0	0
LOC_Os10g40480.1	GATCTGCCCCTCCGACT	0	0	0	0	12	7	14	0	0	0	0	0	0	0	0	0	0	0
LOC_Os10g40510.1	GATCTGTTATGGTTCAG	129	157	0	0	54	276	1	4	0	0	0	31	132	0	106	0	8	0
LOC_Os10g40520.1	GATCTATCTCTTGGTTC	87	54	37	7	0	309	6	0	0	0	0	10	94	3	74	0	0	0
LOC_Os10g40530.1	GATCATTGTATTTTTTT	0	0	0	0	0	0	0	0	0	0	0	0	0	0	11	0	0	0
LOC_Os11g02379.1	GATCGGCCCGGCCGGTC	0	0	458	430	165	2349	42939	221	161	371	13	324	0	668	0	663	0	444
LOC_Os11g02400.1	GATCACGTGCGGGCAGG	0	0	0	21	0	0	19	0	0	0	0	0	0	0	0	35	0	0
LOC_Os11g24070.1	GATCGGCCCGGTCAGTC	0	0	0	0	0	281	20078	0	15	126	5	0	0	0	0	0	0	0
LOC_Os12g02290.1	GATCTGTCTCCTTTACG	0	0	0	0	6	0	0	2	2	748	644	3673	0	0	0	0	10	0
LOC_Os12g02310.1	GATCGCCCGGCCGGTCA	0	0	32	6	228	0	4245	99	0	8	91	43	2	0	0	269	6	0
LOC_Os12g02320.1	GATCGGCCCGGTCACGC	0	0	641	185	37	656	27699	0	17	344	0	831	0	539	0	600	0	345
LOC_Os12g02340.1	GATCTGTGCGTACGTGT	0	0	915	189	164	198	336	0	2897	26	64	0	0	202	0	678	0	74

Library	Description
NCA	35 days - Callus
NCL	14 days - Young leaves stressed in 4C cold for 24h
NCR	14 days - Young roots stressed in 4C cold for 24h
NDL	14 days - Young leaves stressed in drought for 5 days
NDR	14 days - Young roots stressed in drought for 5 days
NGD	10 days - Germinating seedlings grown in dark
NGS	3 days - Germinating seed
NIP	90 days - Immature panicle
NL4	Leaves combined
NME	60 days - Crown vegetative meristematic tissue
NOS	Ovary and mature stigma
NPO	Mature Pollen
NR2	Root combined
NSL	14 days - Young leaves stressed in 250 mM NaCl for 24h
NSR	14 days - Young roots stressed in 250 mM NaCl for 24h
NST	60 days - Stem
NYL	14 days - Young leaves
NYR	14 days - Young Roots
