# Supplementary material for: Characterization of paralogous protein families in rice
Source: BMC Plant Biol. 2008 Feb 19;8:18. doi: 10.1186/1471-2229-8-18 (PMC2275729; doi:10.1186/1471-2229-8-18)

Additional file 11. Schematic illustration of the domain composition of two rice BBI-related paralogous protein families which have Pfam domain PF00228: Family 3328 and Family 1493.

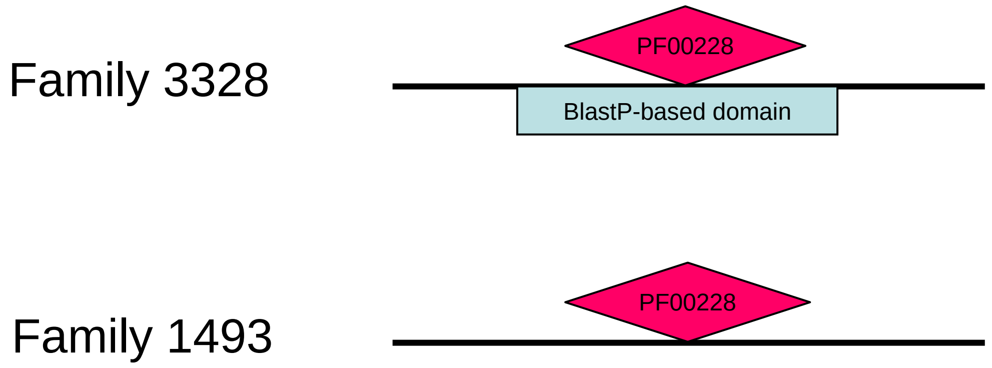

Supplement: Additional File 11 — Schematic illustration of the domain composition of two rice BBI-related paralogous protein families which have Pfam domain PF00228: Family 3328 and Family 1493. [file 1471-2229-8-18-S11.pdf]
