# Supplementary material for: Characterization of paralogous protein families in rice
Source: BMC Plant Biol. 2008 Feb 19;8:18. doi: 10.1186/1471-2229-8-18 (PMC2275729; doi:10.1186/1471-2229-8-18)

Additional file 12. Neighbor-Joining tree of the rice Bowman-Birk inhibitor protein family Family 3328.

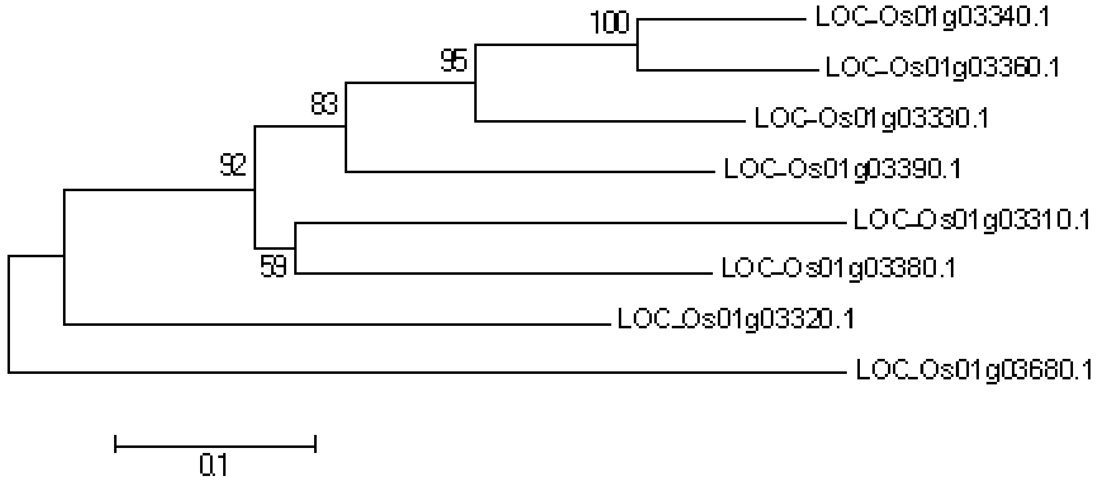

Supplement: Additional File 12 — Neighbor-Joining tree of the rice Bowman-Birk inhibitor protein family Family 3328. [file 1471-2229-8-18-S12.pdf]
