# Supplementary material for: Characterization of paralogous protein families in rice
Source: BMC Plant Biol. 2008 Feb 19;8:18. doi: 10.1186/1471-2229-8-18 (PMC2275729; doi:10.1186/1471-2229-8-18)

Additional file 13. Genome Browser view of the rice genes encoding BBI proteins on chromosome 1. Two tracks, TIGR Rice Loci and TIGR Rice Gene Models, are displayed.

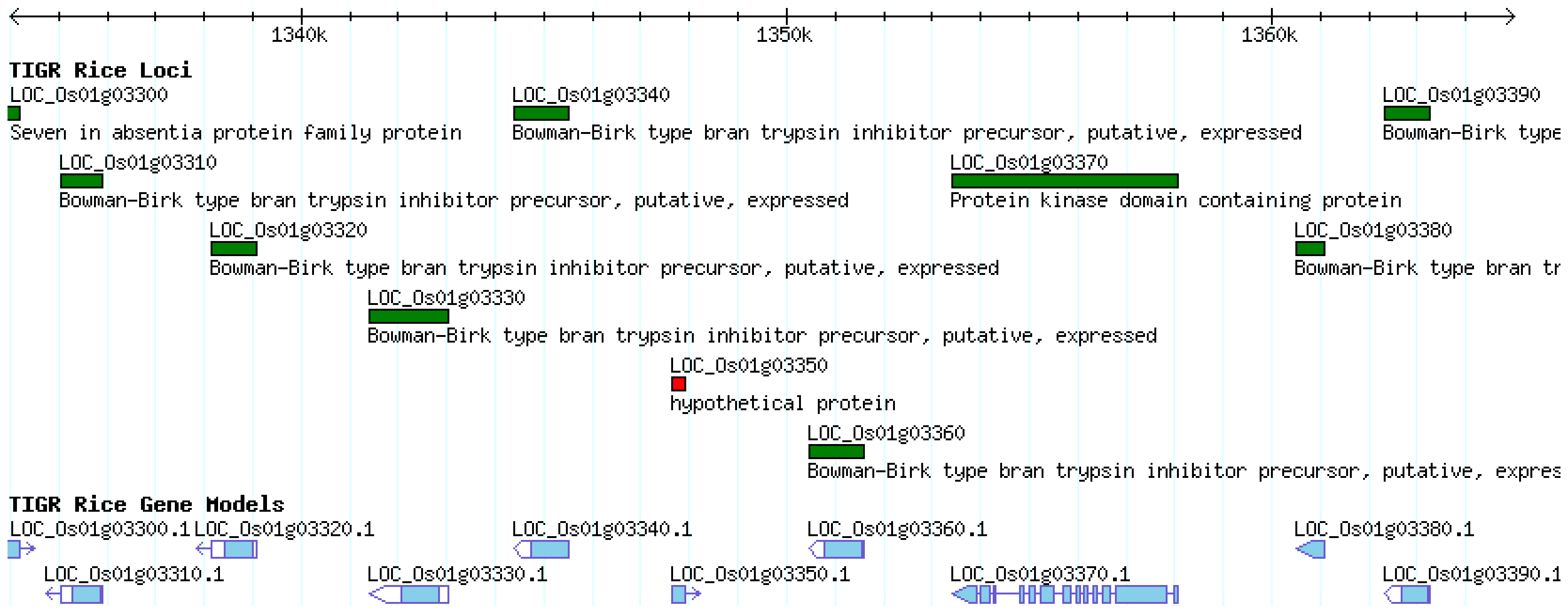

Supplement: Additional File 13 — Genome Browser view of the rice genes encoding BBI proteins on chromosome 1. [file 1471-2229-8-18-S13.pdf]
